# In situ targeted base editing of bacteria in the mouse gut

**DOI:** 10.1038/s41586-024-07681-w

**Published:** 2024-07-10

**Authors:** Andreas K. Brödel, Loïc H. Charpenay, Matthieu Galtier, Fabien J. Fuche, Rémi Terrasse, Chloé Poquet, Jan Havránek, Simone Pignotti, Antonina Krawczyk, Marion Arraou, Gautier Prevot, Dalila Spadoni, Matthew T. N. Yarnall, Edith M. Hessel, Jesus Fernandez-Rodriguez, Xavier Duportet, David Bikard

**Affiliations:** 1 Eligo Bioscience, Paris, France; 2 Institut Pasteur, Université Paris Cité, Synthetic Biology, Paris, France

**Keywords:** Bacteria, Genetic engineering, Gene delivery, Bacteriophages

## Abstract

Microbiome research is now demonstrating a growing number of bacterial strains and genes that affect our health^1^. Although CRISPR-derived tools have shown great success in editing disease-driving genes in human cells^2^, we currently lack the tools to achieve comparable success for bacterial targets in situ. Here we engineer a phage-derived particle to deliver a base editor and modify *Escherichia coli* colonizing the mouse gut. Editing of a β-lactamase gene in a model *E. coli* strain resulted in a median editing efficiency of 93% of the target bacterial population with a single dose. Edited bacteria were stably maintained in the mouse gut for at least 42 days following treatment. This was achieved using a non-replicative DNA vector, preventing maintenance and dissemination of the payload. We then leveraged this approach to edit several genes of therapeutic relevance in *E. coli* and *Klebsiella pneumoniae* strains in vitro and demonstrate in situ editing of a gene involved in the production of curli in a pathogenic *E. coli* strain. Our work demonstrates the feasibility of modifying bacteria directly in the gut, offering a new avenue to investigate the function of bacterial genes and opening the door to the design of new microbiome-targeted therapies.

## Abstract (web summary)

Edited bacteria were stably maintained in mouse gut for at least 42 days following the delivery of a base editor using an engineered phage-derived particle to modify *Escherichia coli* colonizing the gut.

## Main

In recent years microbiome research has unravelled an increasing number of mechanisms by which the expression of genes from commensal bacteria can affect our health. Bacteria can affect the success of immunotherapies^3,4^, bacterial proteins are involved in neurodegenerative^5^ and autoimmune diseases^6–8^, bacterial toxins can drive a range of acute and chronic diseases including cancer^9,10^ and bacteria can modify or sequester drugs affecting the effectiveness of therapies^11–13^. This growing list is driving interest in manipulating the microbiome, both to better understand it and develop new therapies.

Here we propose a strategy to perform in situ, precise and stable genetic modifications of target bacterial populations. Achieving this goal in the complex gut environment requires efficient DNA delivery combined with an efficient targeted mutagenesis strategy. We explore the use of phage-derived particles as delivery vehicles, in combination with base editors. Base editors convert one base pair to another at a target locus without the introduction of a double-strand DNA break^14^ and have been successfully used in a broad range of bacterial species^15–19^.

Several studies have explored the idea of delivering a CRISPR–Cas system to target bacteria with the purpose of killing them or curing plasmids^20^. This has been achieved using different delivery modes, including the conjugation of a plasmid from donor bacteria to the target strain^21–23^, the transduction of a plasmid packaged into phage capsids^23,24^ or the addition of CRISPR–Cas systems to a phage genome^25–27^. Phages are an attractive vector choice because of their DNA delivery efficiency. Whereas lytic phages cannot easily be used when the goal is to modify a target population without killing it, this can be achieved using phagemids or temperate phages^26^.

Previous works on DNA delivery to *Escherichia* *coli* in the mouse gut have relied on either M13 phagemids^28^ or genetically modified bacteriophage λ (ref. ^29^). M13 uses the F pilus as a receptor, which limits its range, and M13 virions were shown to be unstable during passage through the mouse gastrointestinal tract^28^. Other studies have relied on temperate bacteriophage λ. When infecting an *E. coli* cell, λ will either enter its lytic cycle and produce more virions or enter lysogeny and integrate into the chromosome, thereby enabling the maintenance of transgenes such as a type I CRISPR–Cas system^26^ or dead Cas9 (ref. ^29^). More recently a base editor has also been introduced in the genome of bacteriophage λ (ref. ^30^). Despite its ability to reproduce in the gut environment, previous work showed how λ fails to lysogenize the whole target population^29,31^. To improve delivery efficiency in the gut environment we engineer multiple chimeric variants of the λ phage tail to target different receptors.

Another desired feature of an in situ targeted mutagenesis strategy is that it should not spread transgenes. To achieve this we developed a DNA payload that harnesses the replication machinery of a phage-inducible chromosomal island^32^. Our design ensures that the delivered DNA will not be replicated in recipient bacteria, while still allowing for efficient expression of the base editor. This strategy enables the introduction of stable genetic perturbations to the majority of an *E. coli* population colonizing the mouse gut without the need for selection pressure or maintenance of a transgene.

## Engineering of chimeric λ delivery vehicles

**Fig. 1:**
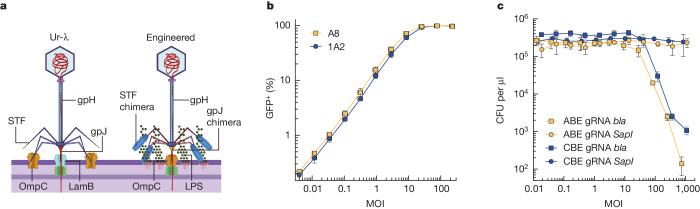
Engineering of an efficient and selective DNA delivery vector for *E. coli* colonizing the mouse gut. **a**, Schematic of the Ur-λ phage injection mechanism. Left, adsorption of phage Ur-λ to *E.* *coli* cells. Gene *stf* encodes for long appendages anchored at the base plate, which promote adsorption of the phage to target bacteria through interaction with the OmpC outer membrane porin. The tail tip protein gpJ recognizes the LamB outer membrane porin and, following binding of gpJ to its receptor, the gpH protein allows for injection of DNA through the periplasm and into the bacterial cytoplasm. Right, engineered λ-derived particles with λ-P2 STF chimeras recognizing LPS and with gpJ chimeras recognizing OmpC. **b**, Delivery efficiency of gpJ cosmid variants with a payload encoding *sfGFP* (plasmid p513) into *E. coli* s14269, measured by flow cytometry (excitation, 488 nm; emission, 530/30 BP). On the *x* axis, MOI represents the ratio of packaged cosmids to bacteria; the *y* axis represents the percentage of GFP^+^ population following incubation for 45 min. The graph shows the average and standard deviation of an experiment performed in triplicate. **c**, MOI-dependent adenine (ABE gRNA *bla*, p1396) and cytosine base editing (CBE gRNA *bla*, p2327) of β-lactamase on strain MG1655*-bla* (gpJ A8, λ-P2 STF chimera). Control samples with a non-targeting *SapI* spacer are shown (ABE gRNA *SapI*, p2771; CBE gRNA *SapI*, p2770). The *y* axis represents colony-forming units (CFU) per microlitre on carbenicillin plates. The graph shows the average and standard deviation of an experiment performed in triplicate. Panel **a** created with BioRender.com. Source Data

The adsorption of phage Ur-λ (ref. ^33^) to *E.* *coli* cells is determined by two main components of the capsid. First, the side tail fibre (*stf*) gene encodes for long appendages anchored at the base plate, promoting reversible adsorption of the phage to target bacteria through interaction with the OmpC outer membrane porin^33,34^ (Fig. 1a). These side tail fibres were lost from the laboratory strain of phage λ (λ PaPa) but are known to be important for efficient adsorption^33^. Second, the tail tip protein gpJ recognizes the LamB outer membrane porin and results in an irreversible binding of the phage to the cell surface^35,36^.

The adaptation of *E. coli* K-12 to the mouse gut selects for mutants that downregulate the expression of the maltose operon, which includes *lamB*^31,37^. These mutants are resistant to bacteriophage λ, which might explain why previous attempts to use this phage for DNA delivery to *E. coli* in the gut failed to reach the whole population^29^. To circumvent this issue and ensure high and consistent delivery efficiency, we engineered a variant of Ur-λ gpJ that uses a different receptor, the outer membrane porin OmpC. The expression of OmpC is upregulated under high-osmotic conditions typical of the gut and is known to be important for growth in this environment^38,39^.

We constructed different gpJ chimeras by fusing the C-terminal portion of naturally occurring gpJ variants found in *E. coli* phages to the λ *gpJ* gene. In addition, to avoid competition between new gpJ variants and natural Ur-λ STF for binding to the same receptor, OmpC, we further constructed a chimera between the N-terminal part of Ur-λ STF and the C-terminal part of the phage P2 tail fibre, known to recognize the lipopolysaccharides of *E.* *coli* K-12 strains^40^. Chimeric gpJ variants were integrated in the Ur-λ prophage genome while the *stf* gene was removed and complemented by the λ-P2 STF chimera encoded on a plasmid (p938) (Fig. 1a). To produce cosmids we used an Ur-λ prophage that carries the cI857 mutation, making it heat inducible and, with its *cos* site deleted, preventing packaging of phage DNA and enabling the packaging of cosmids^41^.

**Extended Data Fig. 1:**
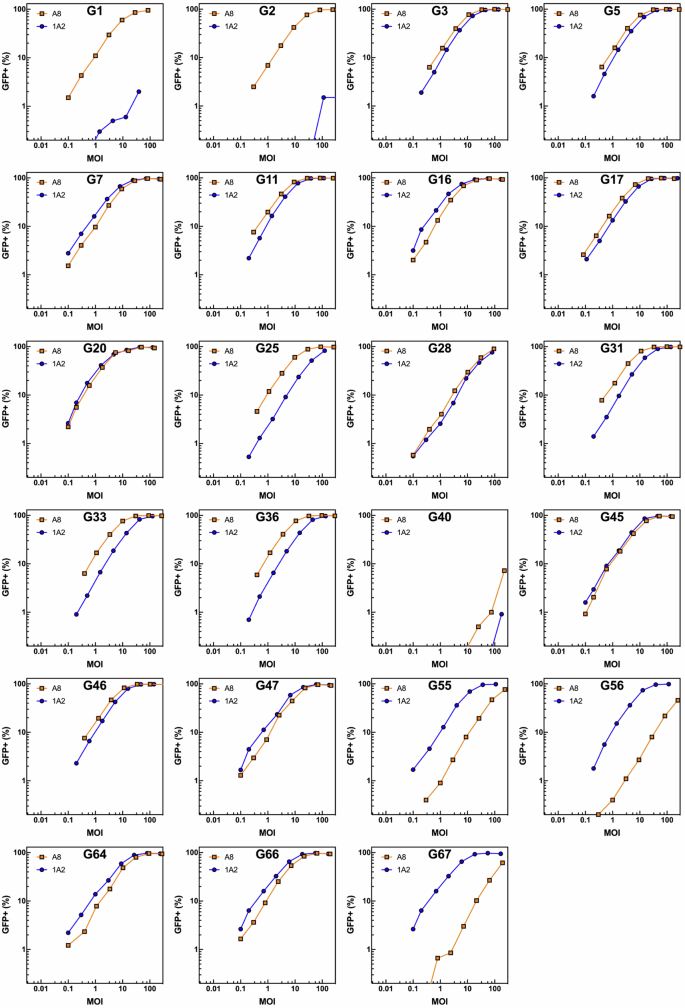
Delivery efficiency of a DNA payload into a MG1655 strain deleted for both *lamB* and *ompC* and complemented with a set of 23 different ompC variants *in trans.* Delivery efficiencies of a λ cosmid harboring P2-STF chimera and gpJ variant A8 (orange lines) or 1A2 (blue lines). Cosmids package a DNA payload encoding *sfGFP* and delivery into *E.* *coli* was measured by flow cytometry (excitation: 488 nm, emission: 530/30 BP). The multiple sequence alignment of the tested OmpC variants is provided in Supplementary Data 10. A phylogenetic tree is provided in Supplementary Fig. 1. Source Data

**Extended Data Fig. 2:**
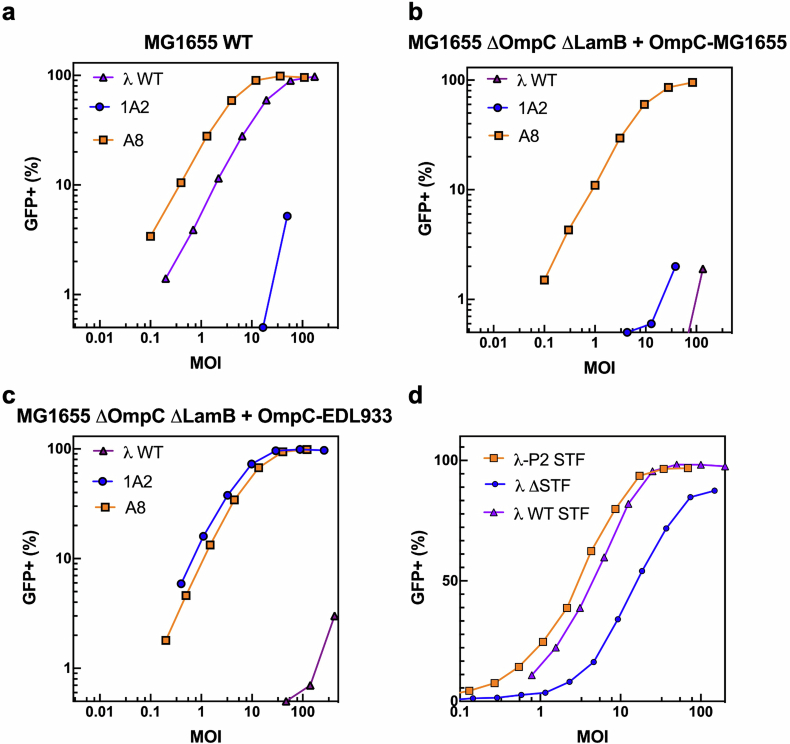
Cell receptor analysis for efficient cosmid delivery. Delivery efficiency assays of cosmids harboring Ur-λ wild-type (WT) gpJ and stf, or harboring gpJ chimeras (1A2 or A8) and P2 STF. Transduction of a payload encoding a *sfGFP* fluorescent protein gene was measured in a flow cytometer (excitation: 488 nm, emission: 530/30 BP; Attune NxT Thermo Scientific) at different MOIs. **a)** Delivery efficiency into MG1655 WT, carrying the endogenous OmpC variant in the genome. **b)** and **c)** Delivery efficiency into a MG1655 strain deleted for both *lamB* and *ompC* genes, complemented with two different *ompC* variants on a plasmid (p1471 or p1472). **d)** Delivery efficiencies in *E. coli* MG1655 of λ particles carrying the WT λ STF (purple line), no STF (blue line) and λ-P2 STF chimera (p938) (orange line). Source Data

To evaluate capsid variants we packaged cosmids designed to express a *sfGFP* gene (plasmid p513) and measured delivery efficiency in different strains by flow cytometry following transduction. A strain deleted for the *lamB* gene was used as recipient to identify gpJ variants that recognize another receptor. In addition, to readily evaluate gpJ chimeras against different natural OmpC variants, we deleted *ompC* and expressed it from a plasmid. We evaluated two different gpJ chimeras (A8 and 1A2) against a panel of 23 OmpC variants, representative of the phylogenetic diversity of this protein in *E. coli* (Supplementary Fig. 1 and Extended Data Fig. 1). Efficient delivery could be observed with chimeras A8, 1A2 or both for 21 out of 23 OmpC variants, showing their broad applicability. We characterized in more detail the delivery efficiencies against the OmpC proteins of *E.* *coli* MG1655 and EDL933, showing that A8 enables delivery to 90% of the population at a multiplicity of infection (MOI) of roughly 20 for both OmpC variants whereas 1A2 efficiently delivers DNA to the EDL933 variant only (Fig. 1b and Extended Data Fig. 2a–c). The functionality of the chimeric λ-P2 STF was further confirmed by the demonstration of markedly improved delivery efficiency compared with capsids lacking an STF (Extended Data Fig. 2d).

## Base editing of a target population in vitro

**Extended Data Fig. 3:**
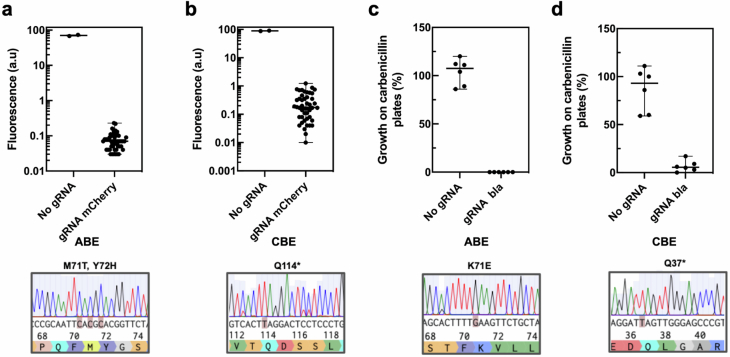
Targeted base editing of the *E. coli* genome *in vitro* after DNA payload transformation and antibiotic selection. **a)** Adenine base editing (ABE8e, plasmid p2325) of MG1655-*mCherry*. The experiment was performed in the presence (48 colonies analyzed) or absence (2 colonies analyzed) of a guide RNA targeting the amino acid triad forming the fluorophore of mCherry (tripeptide: M71, Y72, G73). **b)** Cytosine base editing (CBE = evoAPOBEC1-nCas9-UGI, plasmid p2326) of mCherry on MG1655-*mCherry* genome. The experiment was performed in presence (48 colonies analyzed) or absence (2 colonies analyzed) of a guide RNA inserting a stop codon at position Q114* into mCherry. Fluorescence of individual colonies was measured by flow cytometry (excitation: 561 nm, emission: 620/15 BP; Attune NxT Thermo Scientific). Dots represent single colonies after overnight incubation on chloramphenicol plates. **c)** Adenine base editing of β-lactamase (*bla*) in MG1655-*bla in vitro* (plasmid p1396). The experiment was performed in presence or absence of a guide RNA targeting the active site of β-lactamase (K71E or K71R). **d)** Cytosine base editing of β-lactamase in MG1655-*bla in vitro* (plasmid p2327). The CBE inserts a premature stop codon (Q37*) into the target gene, resulting in the re-sensitization of the bacterial population to β-lactam carbenicillin. The no gRNA controls carried a *SapI* spacer in the ABE or CBE plasmids. Adenine and cytosine base editing of β-lactamase was analyzed by colony counting after overnight incubation on chloramphenicol (Cm) and Cm/carbenicillin agar plates at 30 °C. The percentage growth was obtained by dividing the number of colonies on Cm/carbenicillin plates by the number on Cm plates. One dot represents one transformation after overnight incubation on plates. Graphs show individual values (dots) and average plus standard deviation. Sequencing data at the target site are shown exemplarily for a single base-edited colony. Source Data

**Extended Data Fig. 4:**
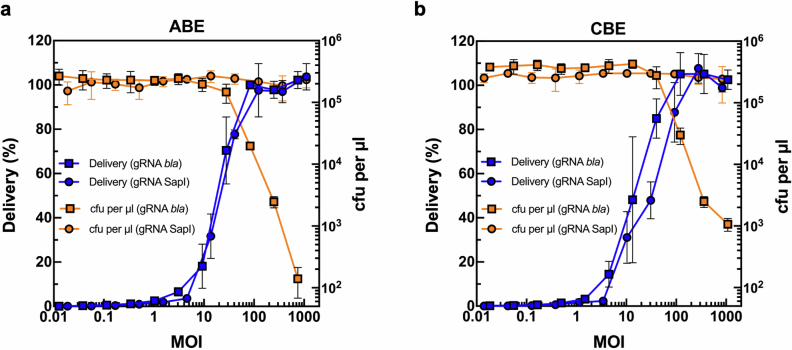
Multiplicity of injection (MOI)-dependent delivery efficiency and corresponding adenine base editing (ABE) and cytosine base editing (CBE). Delivery efficiency was obtained by colony counting after overnight incubation on LB and LB (chloramphenicol) agar plates. Left y axis: The percentage delivery was obtained by dividing the number of colonies on chloramphenicol plates by the number on LB plates (blue line). Right y axis: Base editing of β-lactamase in strain MG1655-*bla* diminishes cell growth on carbenicillin plates (colony-forming units cfu per µl; orange line). For the control samples, the gRNA targeting *bla* was replaced by a non-targeting *SapI* spacer. The graphs show the average and standard deviation of an experiment performed in triplicate. Source Data

Subsequently we constructed cosmids carrying either an adenine base editor (ABE = ABE8e)^42^ or a cytosine base editor (CBE = evoAPOBEC1-nCas9-UGI)^43^. We first established that these cosmids could efficiently edit targets in mCherry or β-lactamase (*bla*) genes following transformation of *E. coli* MG1655 derivatives carrying these genes (MG1655-*mCherry* and MG1655-*bla*). Adenine and cytosine edits were obtained for both targets with over 99% efficiency (Extended Data Fig. 3). We then investigated the feasibility of delivering the base editors encoded in plasmids p1396 (ABE) and p2327 (CBE) with our engineered λ particles to efficiently edit the *bla* gene without selecting for transduction of the cosmid (Fig. 1c). The λ-derived particles were incubated with MG1655-*bla* for 2 h at varying MOI. Because editing the *bla* gene results in its inactivation, base-editing efficiency was measured by colony counting following overnight incubation on carbenicillin plates. ABE or CBE resulted in a reduction of around 10^4^- and 10^3^-fold in cell growth on carbenicillin plates at high MOI, respectively, showing that up to 99.99% of the bacterial population was edited. This reduction in plating efficiency at increasing MOI is consistent with the transduction rates observed, with plating efficiency on selective media starting to drop when most cells received the cosmid (Extended Data Fig. 4).

## Engineering of a non-replicative DNA cosmid

**Fig. 2:**
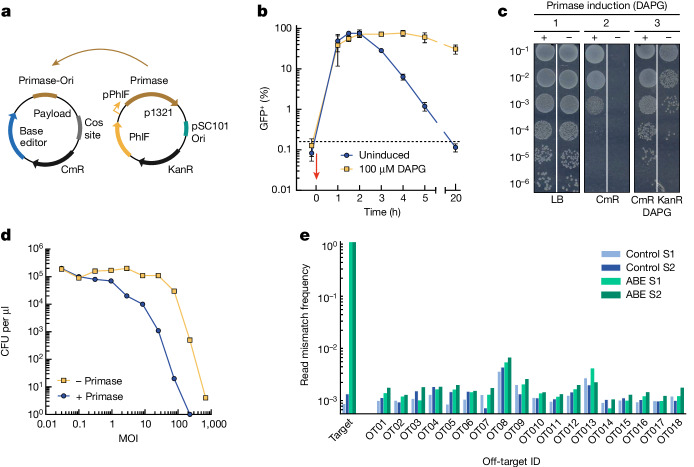
A non-replicative DNA payload can efficiently edit a target bacterial population. **a**, Schematics for the conditional replication of a cosmid. Replication requires both the primase protein and the primase origin of replication (Primase-Ori). **b**, In vitro plasmid stability time-course assay. Bacteria carrying an inducible primase plasmid were grown either without inducer (blue) or with 100 µM DAPG (yellow). A cosmid harbouring a *sfGFP* gene (p1324) was delivered at an approximate MOI of 40 (red arrow). Samples from different time points were analysed in a flow cytometer. The graph shows the average and standard deviation of an experiment performed in triplicate. Dashed line indicates background fluorescence of cells before transduction. **c**, Cells carrying an induced (+) or uninduced (−) primase plasmid were transduced with a payload carrying the conditional origin of replication, incubated for 5 h and plated on either (1) lysogeny broth agar, (2) lysogeny broth agar with chloramphenicol 25 µg ml^−1^ or (3) lysogeny broth agar with kanamycin 50 µg ml^−1^, chloramphenicol 25 µg ml^−1^ and DAPG 100 µM. **d**, In vitro adenine base editing of *bla* using a non-replicative payload (p2328). MG1655-*bla* was transduced in either the presence (blue) or absence (yellow) of primase expression (plasmid p1321). After 2 h, cells transduced at varying MOI were plated on lysogeny broth with carbenicillin. **e**, Analysis of on- and off-target editing in MG1655-*bla* by next-generation sequencing. The frequency of mismatches in Illumina sequencing data is shown for all 20 base pair subsequences followed by a NGG PAM in the reference genome with up to seven mismatches to the target sequence, including up to two in the ten PAM-proximal nucleotides. Bars represent the mismatch frequency of the base with the highest frequency in each off-target. The sequence, coordinates and neighbouring genes of each off-target are listed in Supplementary Data 1. Data shown for two biological replicates of samples treated with either ABE or control. Source Data

When considering the delivery of a DNA payload in patients, it is desirable to avoid the dissemination of transgenes. To this end we developed a cosmid that replicates only in the production strain and not in recipient bacteria. We modified our cosmid by replacing the p15A origin of replication with that of a phage-inducible chromosomal island that requires a specific primase gene for replication^44^. We constructed a production strain expressing the primase gene on an additional plasmid under the control of the 2,4-diacetylphloroglucinol (DAPG)-inducible PhlF promoter^45^ (plasmid p1321) (Fig. 2a).

A time-course experiment was performed to investigate plasmid stability in the presence or absence of the primase protein (Fig. 2b). Stability was measured by flow cytometry over time following transduction in *E. coli* MG1655 + p1321 using packaged Ur-λ cosmids encoding a *sfGFP* reporter gene (p1324) in the presence or absence of the inducer DAPG. After 1–2 h, high delivery efficiency could be measured under both conditions whereas following 5 h of incubation only around 1% of the cells were positive for green fluorescent protein (GFP) in the absence of primase, compared with roughly 75% of induced cells. The absence of plasmid replication in the absence of primase was further confirmed by plating on lysogeny broth agar supplemented with chloramphenicol 5 h after transduction. No colonies appeared in the uninduced primase sample, indicating that the payload was indeed lost (Fig. 2c).

Our next focus was on determining whether the transient expression of a base editor by a non-replicative DNA payload would be sufficient to edit an entire target bacterial population. We constructed a conditionally replicative cosmid with ABE programmed to target *bla* (p2328) and packaged it in the engineered λ capsid with 1A2 gpJ and λ-P2 STF chimeras. The packaged cosmid was used to transduce *E. coli* MG1655 carrying the EDL933 OmpC receptor and *bla* (s14269-*bla*) in the presence or absence of the primase protein. Cells were plated with or without carbenicillin, and base-editing efficiency was analysed via colony counting the following day (Fig. 2d). Whereas editing efficiency was slightly reduced in the absence of plasmid replication, the ABE still resulted in a reduction of about 10^4^-fold in cell growth on carbenicillin at MOI of over 100 using the non-replicative payload. This result demonstrates that up to 99.99% of the bacterial population was edited.

We further investigated the occurrence of any off-target base edits in strain MG1655-*bla* following the delivery of the non-replicative ABE payload at an approximate MOI of either 227 (sample ABE S1) or 195 (sample ABE S2). For this we extracted DNA from the bacterial population and performed Illumina sequencing at an average coverage of over 6,000. An on-target base-editing efficiency of around 98% was measured for both MOIs, with a base edit at position 7A in the editing window for all ABE samples as well as bystander mutations at positions 1A, 8A and 9A (Supplementary Fig. 2). For detection of off-target mutations we compared the frequency of mismatched reads in the treated sample to that of a control sample in which the phagemid solution was replaced by lysogeny broth medium. We did not detect off-target mutations at candidate off-target sites based on their similarity to the guide sequence (Fig. 2e and Supplementary Fig. 2). Beyond guide-dependent off-target effects, the expression of an adenosine deaminase could result in an overall elevated mutation rate, in particular at adenine positions. Whereas some positions appeared to be mutated at a higher rate in the treated samples, a similar number of positions showed an elevated mutation rate in the control samples. Our experiment thus did not show an increased mutation rate above the error rate of Illumina sequencing (Supplementary Fig. 3).

## Targeted base editing of non-pathogenic *E. coli* in the mouse gut

**Fig. 3:**
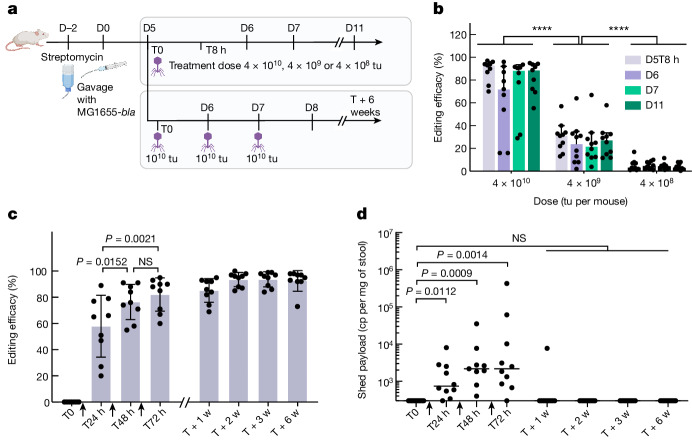
Targeted adenine base editing of *E. coli* MG1655-*bla* in the gut of BALB/c mice. **a**, Summary of the experimental set-up. Five days following colonization, mice were treated with packaged cosmids equipped with gpJ 1A2 and the λ-P2 STF chimera, and an ABE targeting the *bla* gene. In one arm we investigated dose–response and in the other the impact of multiple doses on treatment efficacy, following animals up to 6 weeks (w). Dose titres were measured as transducing units (tu). **b**, Editing efficiency at different time points for a single dose with decreasing concentrations. Points show individual mice (*n* = 10 per group), bars indicate median ± 95% confidence interval (****P < 0.0001 by one-way analysis of variance (ANOVA) with Tukey’s multiple-comparisons test). **c**, Editing efficiency following multiple treatments (*P* values indicated on the graph; not significant (NS), *P* > 0.05 by repeated-measures ANOVA with Tukey’s multiple-comparisons test; treatments are indicated by black arrowheads on the *x* axis; *n* = 9 animals). Bars represent mean ± s.d. **d**, Number of copies of payload (cp) recovered in the stool and quantified by ddPCR. Bars represent the group median (*P* values indicated on the graph; NS *P* > 0.05 by Kruskal–Wallis test with Dunn’s multiple-comparisons test; *n* = 10 animals). D0, day 0; T0, time 0. Panel **a** created with BioRender.com. Source Data

We generated a streptomycin-resistant variant (*rpsL K42R*) of the s14269-*bla* strain (s21052) that could be used in an in vivo mouse colonization model. BALB/c mice were treated with streptomycin to allow for the intestinal engraftment of orally administered *E. coli* s21052. Five days following colonization, mice were orally gavaged with packaged cosmids harbouring gpJ 1A2 and the λ-P2 STF chimera, and an ABE targeting the *bla* gene (p2328) (Fig. 3a). For quantitative monitoring of base-editing efficiency in mouse stool samples we established a digital droplet PCR (ddPCR) assay based on two competing probes, enabling the relative quantification of both genotypes (Supplementary Fig. 4 and Methods).

**Extended Data Fig. 5:**
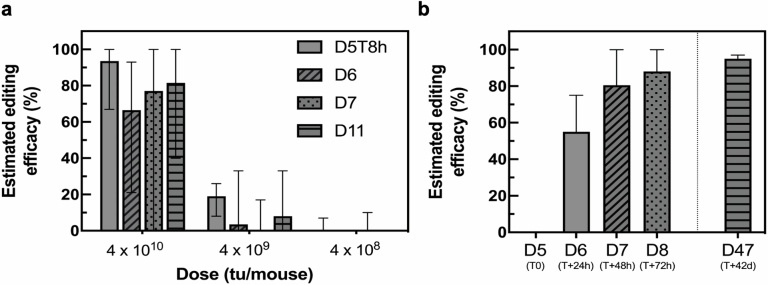
Estimated efficacy of targeted adenine base editing on the *E. coli* genome in the gut of BALB/c mice after cosmid treatment using a non-replicative payload. **a)** Estimation of the editing efficacy as measured by repatching individual colonies onto agar plate with or without carbenicillin, for the initial dose-response experiment, and **b)** for the treatment with multiple doses. Bars represent the group median, with 95% confidence interval. Source Data

A dose-dependent increase in base-editing efficacy was observed, reaching a median efficacy of 93% at the highest dose (4 × 10^10^ particles) 8 h following treatment (Fig. 3b). Colonies recovered from stool were further restreaked onto selective medium to corroborate ddPCR data (Extended Data Fig. 5). The edited population remained stable for at least 6 days following treatment, suggesting no obvious fitness cost of the targeted genetic modification.

**Extended Data Fig. 6:**
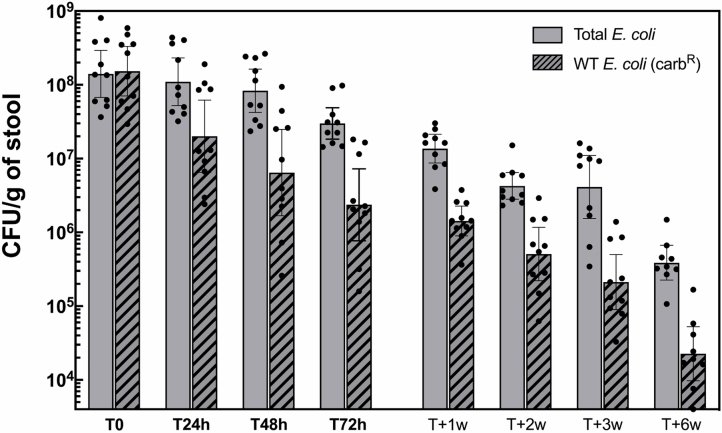
Colonization levels of *E. coli* s21052 in the streptomycin-treated BALB/c mice during treatment with base-editing cosmid particles. **a)** Total *E. coli* bacteria were quantified by plating resuspended stool samples onto Drigalski plates supplemented with streptomycin. Wild-type WT (ie, non-edited) *E. coli* s21052 were quantified by plating samples onto Drigalski plates with carbenicillin. Source Data

We then assessed whether the administration of several doses would increase the relative abundance of the edited population. We selected an intermediate dose (1 × 10^10^ particles) and administered one dose per day for three consecutive days to the mice. Each dose increased median editing efficacy—from 65 to 76% and finally to 88% of the target bacterial population (Fig. 3c). Note that in this animal model, although the colonization level of *E. coli* s21052 decreased over the 6-week experiment, the average proportion of edited versus unedited *E. coli* remained stable (Extended Data Fig. 6). Over time individual mice showed slight fluctuations in the proportion of edited bacteria, with the highest ratio measured at the 3-week time point in a mouse in which 99.7% of the *E. coli* population carried the desired modification as measured by ddPCR.

We did not detect payload in the stool of treated animals 11 days after the last treatment, and detected it in only one out of ten animals 4 days following the last treatment (Fig. 3d). This showed that the non-replicative payload was not maintained in target bacteria but still allowed for efficient editing.

**Extended Data Fig. 7:**
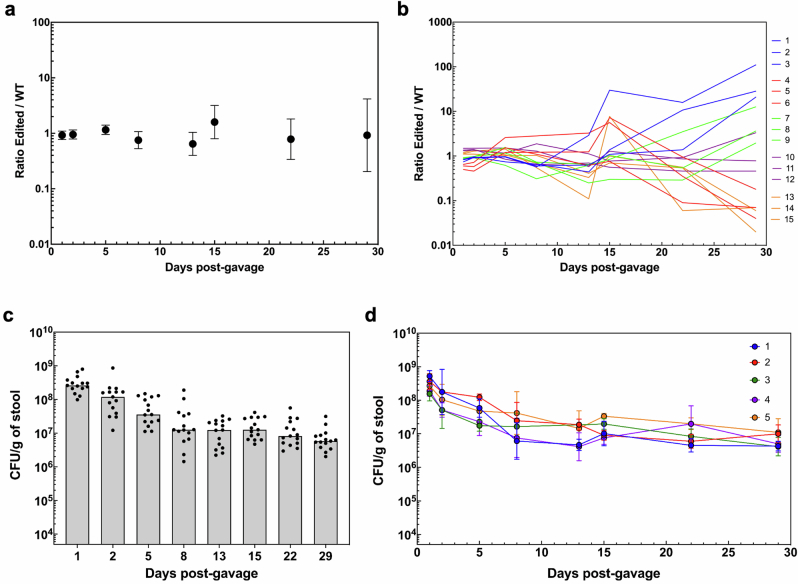
Fitness experiment of the *bla*-edited versus non-edited strain in the gut of BALB/c mice. **a)** Time-course of the relative abundance of the *bla*-edited compared to the WT *E. coli* s21052 strain in 15 mice. Five groups of three mice were treated with independent pairs of clones at an approximate ratio of 1:1 (from 0.92 to 0.96). Plots represent geometric mean and error bars the 95% confidence interval. **b)** Individual mice data from the same experiment. Colors represent the group of each animal. **c)** Time-course of the *E. coli* s21052 colonization level in the streptomycin-treated BALB/c model. Total *E.* *coli* bacteria were quantified by plating resuspended stool samples onto Drigalski plates supplemented with streptomycin. Bars represent the median and each dot individual values. **d)** *E. coli* s21052 colonization level in the streptomycin-treated BALB/c model for each group. Dots represent the geometric mean, and error bars the geometric standard deviation. Source Data

A slight increase in the frequency of the edit can be seen following our last treatment (Fig. 3c). This raises the question of whether modification of the *bla* gene could provide a fitness advantage to *E. coli* in the mouse gut. To investigate this we performed a competition experiment in which *bla*-edited and unedited strains were administered to mice at a ratio of 1:1 (Extended Data Fig. 7). Although individual mice showed large fluctuations in the proportion of the two strains starting from the 2-week time point, on average the *bla*-edited strain did not show a fitness advantage over the unedited strain. *E. coli* is known to adapt to the mouse gut environment through the acquisition of diverse mutations^37,46^. Such mutations being rare, they are more likely to occur in edited cells if editing frequency is higher than 50%. Our edits can then probably hitchhike on beneficial mutations, explaining the continued increase in the frequency of the edited allele following the last base-editor treatment.

## Targeted base editing of pathogenic strains in vitro

We then evaluated the feasibility of engineering delivery vectors and obtaining efficient base editing in natural pathogenic isolates. To this end we selected the uropathogenic K1 *E. coli* strain UTI89 (ref. ^47^), an ST131 strain associated with extra-intestinal infections (TN03)^48,49^ and a *Klebsiella pneumoniae* strain belonging to sequence type 258 (ST258)^50^.

**Extended Data Fig. 8:**
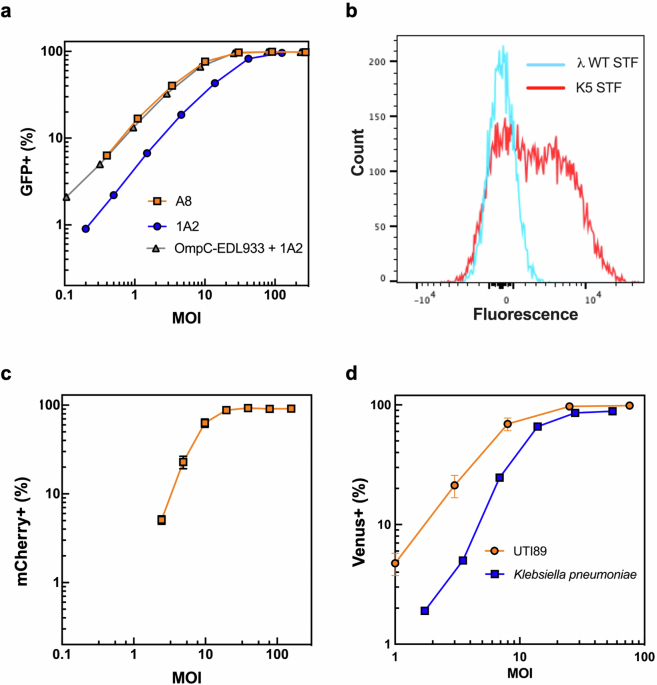
Delivery efficiencies into pathogenic strains of *E. coli* and *K. pneumoniae*. **a)** Delivery efficiency assays in MG1655 carrying TN03-OmpC of cosmids harboring A8 (orange line) or 1A2 (blue line) gpJ chimeras and P2 STF. Grey line shows a comparison of the delivery efficiency of gpJ chimera 1A2 into MG1655 carrying OmpC-EDL933. Transduction of a payload encoding a *sfGFP* fluorescent protein gene was measured in a flow cytometer (excitation: 488 nm, emission: 530/30 BP) at different MOIs. **b)** Flow cytometry histogram of *E. coli* TN03 after delivery with a cosmid harboring λ-A8 gpJ chimera and λ-WT STF (blue line) or λ-K5 STF (red line) at an estimated MOI of ~10. Transduction of a payload encoding a *mCherry* fluorescent protein gene was measured in a flow cytometer (excitation: 561 nm, emission: 620/15 BP). **c)** Delivery efficiency into *E. coli* TN03 of λ particles carrying the λ-K5 STF chimera and payload from panel *b* at different MOIs. **d)** Delivery efficiencies of a DNA payload encoding the fluorescent gene *venus* (plasmid p2075) into UTI89 (gpJ 1A2, λ-K1F STF chimera), or a DNA payload encoding the fluorescent gene *mCherry* (plasmid p2074) into TN03 (gpJ A8, λ-K5 STF chimera) or *Klebsiella pneumoniae* (gpJ A8, λ-KL106 STF chimera), measured by flow cytometry (excitation: 488 nm, emission: 530/30 BP). Source Data

We first evaluated the ability of chimeras 1A2 and A8 gpJ to target the OmpC variant of *E. coli* TN03 by expressing it from a plasmid in an MG1655-*ΔlamBΔompC* strain. We obtained good delivery efficiency with gpJ A8 (Extended Data Fig. 8a). In silico analysis further showed that TN03 probably produces a K5 capsule^51^. We therefore engineered a chimeric STF using the tail fibre from a phage known to infect K5 strains^52^. Capsids carrying the λ-K5 STF chimera and A8 gpJ enabled delivery into over 90% of a TN03 population at an approximate MOI of 30 in vitro (Extended Data Fig. 8b,c).

Because UTI89 is known to express the K1 capsule^53^, we followed the same approach as for TN03 but this time using a tail fibre from a phage known to infect K1 strains^54^. Delivery into over 99% of the UTI89 population was obtained using a capsid carrying the λ-K1F STF chimera and A8 gpJ at an approximate MOI of 76 (Extended Data Fig. 8d).

Based on in silico analysis of *K. pneumoniae*, we hypothesized that our ST258 strain shows a KL106 capsule^51^. We were able to deliver a reporter payload to over 90% of the target population at an approximate MOI of 56 with a capsid carrying the A8 gpJ and a chimera between the N-terminal part of the Ur-λ STF and C-terminal part of a prophage STF found in a *K.* *pneumoniae* KL106 strain (Extended Data Fig. 8d).

We further investigated the specificity of our chimeric particles. We first evaluated the ability of eight vectors harbouring all possible combinations of the A8 or 1A2 gpJ variant and STF chimeras P2, K1F, K5 or KL106 into eight different *Enterobacteria* species and five distant species. Whereas some vectors showed efficient delivery only to their target strain, other vectors showed delivery to multiple *Enterobacteria* and no delivery could be detected for any of the other species (Supplementary Fig. 5). For instance, vectors able to deliver into *E. coli* UTI89 also showed delivery into the *Escherichia fergusonii* strain of our panel, suggesting that both harbour a K1 capsule. In addition, particles harbouring the chimeric P2 STF were able to inject at varying efficiencies in diverse *Enterobacteria*. Using four of the chimeric particles we then evaluated the potential delivery and maintenance of a replicative payload carrying a chloramphenicol resistance gene to bacteria present in mouse faeces. We could not detect delivery into any bacteria other than the targeted *E.* *coli* strain, as measured by plating under anaerobic conditions on rich medium containing chloramphenicol (Supplementary Fig. 6).

**Fig. 4:**
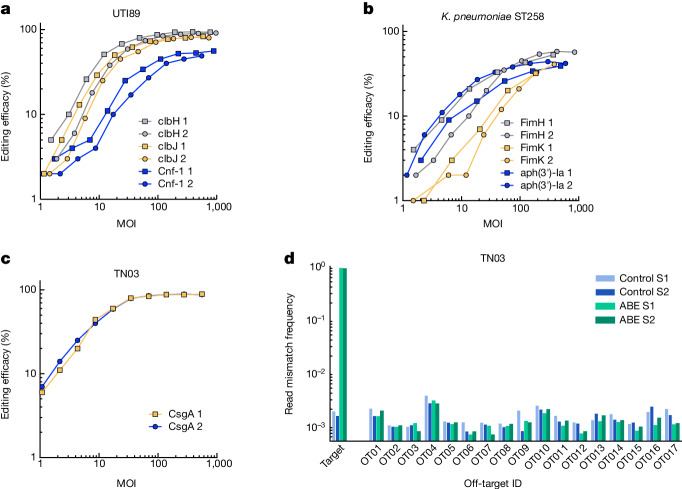
Targeted base editing of pathogenic *E. coli* and *K. pneumoniae* strains in vitro using a non-replicative DNA payload. **a**, MOI-dependent cytosine base editing of target genes *clbH*, *clbJ* and *cnf1* in strain UTI89 using a vector harbouring the λ-K1F STF chimera and gpJ 1A2. The CBE inserts a premature stop codon into the target genes. **b**, MOI-dependent cytosine base editing of target genes *fimH*, *fimK* and *aph(3*′*)-Ia* in *K. pneumoniae* ST258 using a vector harbouring the λ-KL106 STF chimera and gpJ A8. The CBE inserts a premature stop codon into the target genes. **c**, MOI-dependent adenine base editing of the start codon of *csgA* (ATG to ACG) in strain TN03 using a vector harbouring the λ-K5 STF chimera and gpJ A8. **d**, Next-generation sequencing analysis of on- and off-target adenine base editing (ABE8e, plasmid p2515) in TN03-*csgA* in vitro. The frequency of mismatches in Illumina sequencing data is shown for all 20 base pair subsequences with an NGG PAM in the reference genome with up to seven mismatches to the target sequence, including up to two in the ten PAM-proximal nucleotides. Bars represent mismatch frequency of the base with the highest frequency in each off-target. The sequence, coordinates and neighbouring genes of each off-target are listed in Supplementary Data 2. Data shown for two biological replicates of samples treated with either ABE or control. Base-editing experiments shown in **a**–**c** were performed in duplicate (1 and 2). Source Data

For evaluation of editing efficiency we selected several therapeutically relevant targets for *E. coli* UTI89, TN03 and *K. pneumoniae ST258*. In *E. coli* strain UTI89 we targeted *clbH* and *clbJ*, two genes of the colibactin cluster associated with colorectal cancer^55^. In addition we selected *cnf1*, a virulence factor^56^. The gRNA of the non-replicative CBE payload was modified to insert stop codons at the target sites and delivered using chimera λ-K1F STF and A8 gpJ. On-target cytosine base editing efficiency was roughly 92% (*clbH*), 83% (*clbJ*) and 53% (*cnf1*) at an MOI of over 100 as measured by sequencing (Fig. 4a).

As target genes for *K. pneumoniae ST258* we chose the virulence factors *fimH* and *fimK*^57,58^, as well as *aph(3*′*)-Ia*, an aminoglycoside resistance gene. The gRNA of the non-replicative CBE payload was modified to insert stop codons in *fimH*, *fimK* *or aph(3*′*)-Ia* and the cosmid was delivered using chimera λ-KL106 STF and A8 gpJ (Fig. 4b). We obtained approximate on-target cytosine base-editing efficiencies of 56% for *fimH*, 37% for *fimK and* 42% for *aph(3*′*)-Ia*, demonstrating that this technology can be applied to species other than *E. coli* while still using λ as a chassis for our delivery vector.

As a target gene for TN03 we chose *csgA*, the major subunit of curli, an amyloid bacterial appendage hypothesized as having a role in several neurodegenerative^5,59^, inflammatory and autoimmune diseases^60^. The gRNA of the non-replicative ABE payload was modified to target the start codon of the *csgA* gene (ATG to ACG) in TN03 and the cosmid was delivered using chimera λ-K5 STF and A8 gpJ. On-target base editing efficiency was about 90% at an approximate MOI of 51 as measured by sequencing (Fig. 4c). We observed a base edit at the desired target site (6A) in the editing window, as well as bystander mutations at positions 10A and 11A located upstream of the *csgA* start codon. Dot-blot analysis revealed that the CsgA protein was undetectable in base-edited strains (Supplementary Fig. 7). In accordance with the results for MG1655-*bla*, Illumina sequencing showed no detectable off-target mutations at candidate sites based on their similarity to the guide sequence (Fig 4d and Supplementary Fig. 8). We also did not detect any increase in overall mutation rate above the error rate of Illumina sequencing (Supplementary Fig. 9).

## Targeted base editing of *E. coli* TN03 in the mouse gut

**Fig. 5:**
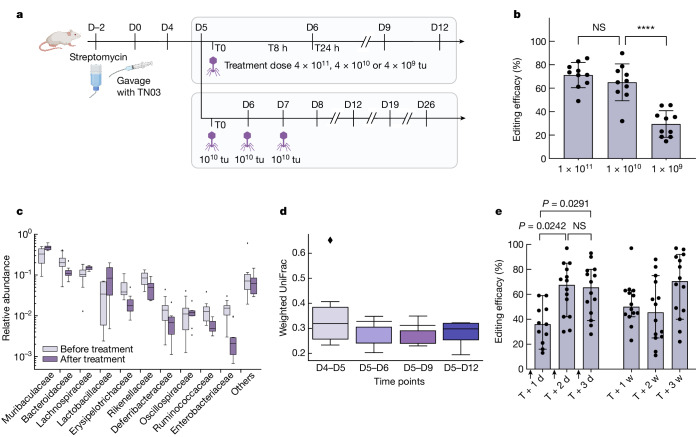
Targeted adenine base editing of gene *csgA* on the *E. coli* TN03 genome in the gut of BALB/c mice following packaged λ cosmid treatment using a non-replicative payload. **a**, Summary of the experimental set-up. Five days following colonization, mice were treated with packaged cosmids equipped with gpJ A8 and λ-K5 STF chimera encoding an ABE targeting the *csgA* gene. In one arm we investigated the dose–response and in the other the impact of multiple doses on treatment efficacy. **b**, Editing efficacy 24 h following a single dose at decreasing concentration (*n* = 10 independent animals per group, ****P < 0.0001 by one-way ANOVA with Tukey’s multiple-comparisons test). Bars represent mean ± s.d. **c**, Family-level bacterial composition quantified by metagenomic 16S sequencing before (D4) and after treatment (D12) with 1 × 10^10^ particles (*n* = 10 animals); data for all mice and time points are provided in Extended Data Fig. 10. Boxes are drawn from the first to third quartile, with the midline representing the median; whiskers extend to the minimum and maximum in each category, excluding outliers shown as black diamonds. **d**, Changes in intestinal microbiome composition of individual mice over time, represented as weighted UniFrac distance from the D5 sample. Stool samples of mice treated with 1 × 10^10^ particles were analysed (*n* = 9 animals). The plot is drawn as in **c**. **e**, Editing efficacy following multiple treatments (*P* values indicated on the graph; NS, *P* > 0.05 by one-way ANOVA with Tukey’s multiple-comparisons test; *n* = 14 animals); treatments are indicated by black arrowheads on the *x* axis). Points show individual mice and bars represent the group median, with 95% confidence interval when applicable. Panel **a** created with BioRender.com. Source Data

**Extended Data Fig. 9:**
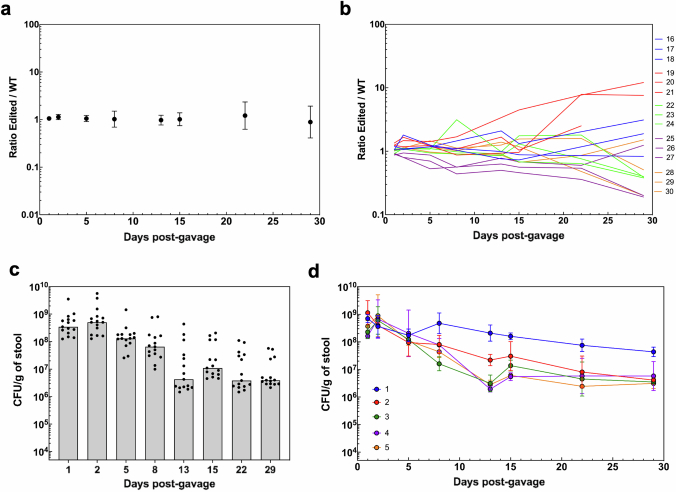
Fitness experiment of the edited versus WT *E. coli* TN03 strain in the gut of BALB/c mice. **a)** Time-course of the relative ratio of the edited compared to the WT TN03 strain in 15 mice. Five groups of three mice were treated with independent pairs of clones at an approximate ratio of 1:1 (from 1.06 to 1.07). Plots represent geometric mean and error bars with a 95% confidence interval. **b)** Individual mice data from the same experiment (mouse number 16 to 30). Colors represent the group of origin for each animal. **c)** *E. coli* TN03 colonization level in the streptomycin-treated BALB/c model. Total *E.* *coli* bacteria were quantified by plating resuspended stool samples onto Drigalski plates supplemented with streptomycin. Bars represent the median and each dot individual values. **d)** *E. coli* TN03 colonization levels in the streptomycin-treated BALB/c model for each group. Dots represent the geometric mean, and error bars the geometric standard deviation. Source Data

Motivated by the high editing efficiencies obtained in vitro, we selected the ST131 strain (TN03) and *csgA* as a target to demonstrate base editing of a pathogenic strain in the mouse gut. We obtained a streptomycin-resistant variant (*rpsL K42Q*) of strain TN03 (s21476) to enable colonization of the mouse gut during streptomycin treatment. BALB/c mice were treated with streptomycin to allow for the intestinal engraftment of orally administered TN03 (s21476). Five days following colonization, mice were gavaged with three different doses of packaged cosmids encoding an ABE targeting the *csgA* gene (p2515) (Fig. 5a). Base editing efficacy was dose dependent, reaching 64–79% (median 71%) in the target population 24 h following treatment at the highest dose (1 × 10^11^ particles) (Fig. 5b). To determine whether editing of *csgA* could affect the fitness of strain TN03 and affect the measured efficiencies, we performed a competition experiment between edited and unedited strains; this showed that our edit is probably neutral (Extended Data Fig. 9).

**Extended Data Fig. 10:**
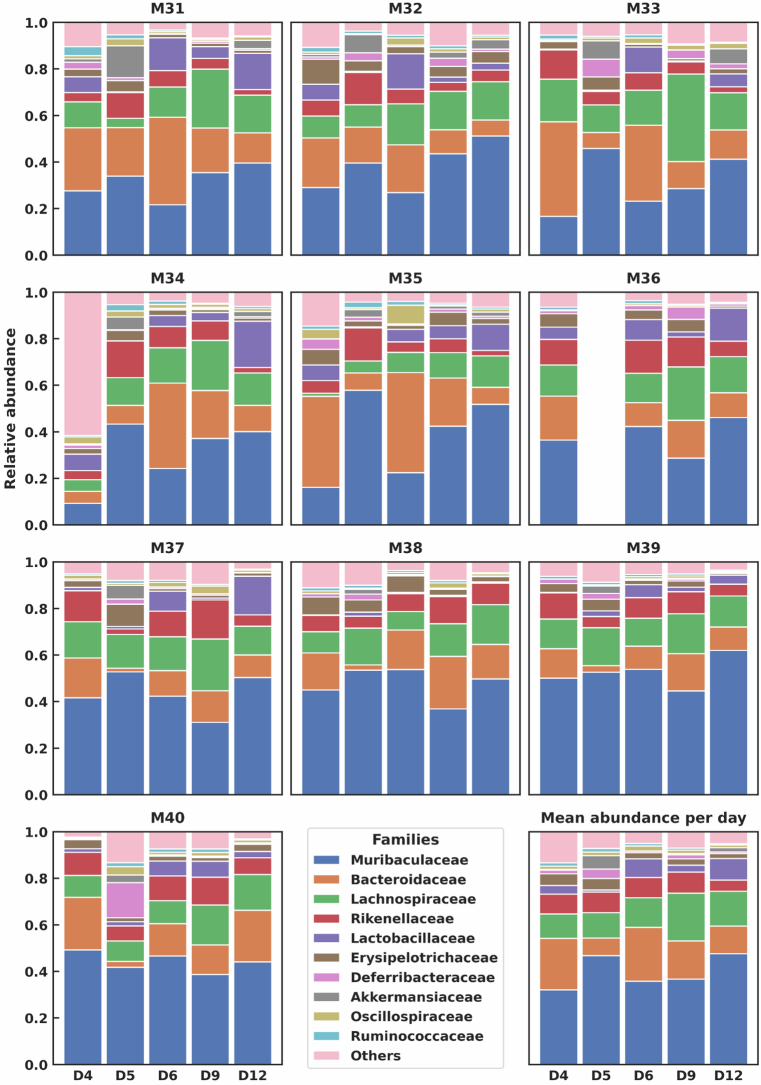
Family-level bacterial composition quantified by metagenomic 16S sequencing for ten mice treated with 1 x 10^10^ particles per mouse. Metagenomic 16S sequencing of stool samples was performed at two time points before treatment (D4 and D5) as well as three time points after treatment (D6, D9, and D12). The sample D5 M36 had to be excluded from the analysis due to low sequencing depth. The mean abundance per day is the average across the ten mice sequenced. The raw microbiome composition data are included in Supplementary Data. Source Data

**Extended Data Fig. 11:**
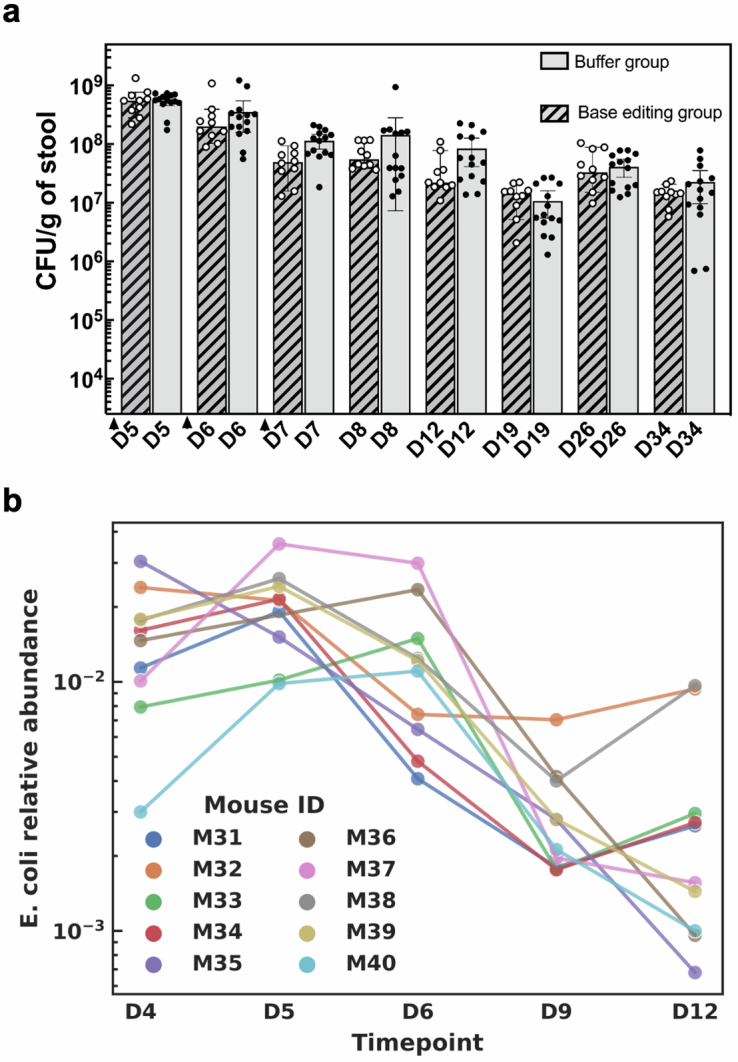
Colonization levels of *E. coli* s21476. **a)** Colonization levels in streptomycin-treated BALB/c mice after multiple treatment. Total *E.* *coli* bacteria were quantified by plating resuspended stool samples onto Drigalski plates supplemented with streptomycin (treatments are indicated with black arrowheads on the x-axis). **b)** Relative abundance of *E.* *coli* quantified by metagenomic 16S sequencing for ten mice treated with 1 x 10^10^ particles per mouse. Metagenomic 16S sequencing of stool samples was performed at two time points before treatment (D4 and D5) as well as three time points after treatment (D6, D9, and D12) for ten mice gavaged with 1 x 10^10^ particles per mouse. Source Data

**Extended Data Fig. 12:**
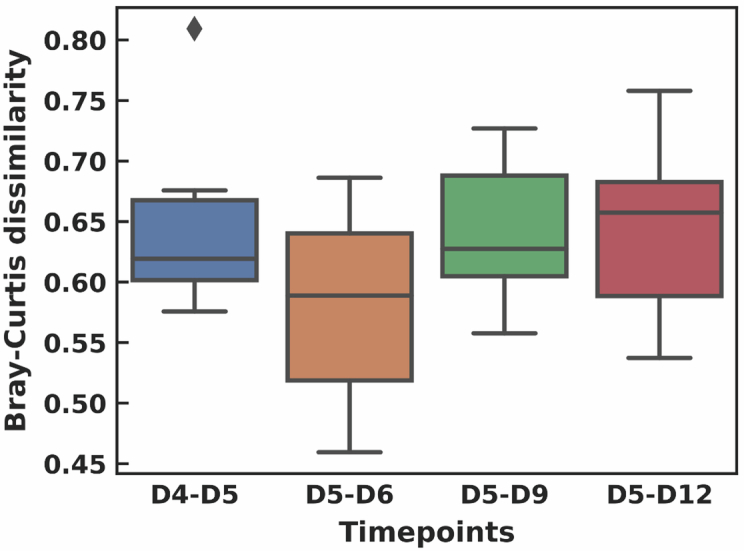
Bray-Curtis dissimilarity analysis of microbial communities. Metagenomic 16S sequencing of stool samples was performed at two time points before treatment (D4 and D5) as well as three time points after treatment (D6, D9, and D12) for ten mice gavaged with 1 x 10^10^ particles per mouse (n = 9 independent animals for each pair of timepoints due to a failed sequencing in one case). The boxes are drawn from the 1st to 3rd quartile with the middle line representing the median. Whiskers extend to the minimum and maximum in each category, excluding outliers shown as black diamonds. Only distances between samples from the same individual at different time points are shown. Source Data

To investigate the potential effect of our treatment on overall microbiome composition, we performed metagenomic 16S sequencing at two time points before treatment (D4 and D5), as well as at three time points following treatment (D6, D9 and D12) with the intermediate dose (1 × 10^10^ particles) (Fig. 5c,d and Extended Data Fig. 10). The data showed some fluctuation in microbiome composition between mice, and over the course of the experiment, which could be explained by the response to streptomycin treatment. The drop observed for Enterobacteriaceae is driven by the *E. coli* population, which naturally drops in this model (Fig. 5c and Extended Data Fig. 11). However, changes in microbiome composition following treatment with the base editor (weighted UniFrac distance or Bray–Curtis dissimilarity between D5 samples and post-treatment samples) are no greater than those that occurred before treatment (distance between D4 and D5 samples; Fig. 5d and Extended Data Fig. 12). This suggests that our treatment did not have a noticeable effect on microbiome composition.

Finally, we administered one dose of 1 × 10^10^ particles per day for three consecutive days (Fig. 5e). The first two doses increased median editing efficacy from 36 to 68%, whereas the third did not further affect efficacy. The average proportion of edited TN03 cells fluctuated over the 3 weeks of the experiments and showed greater mouse-to-mouse variation than the previous experiment on strain s14269-*bla*. Nonetheless, the median proportion of the edited bacterial population was still roughly 70% 3 weeks after treatment.

## Discussion

We demonstrate the efficient and durable genetic modification of a bacterial population in the gut environment using engineered, non-replicative, phage-derived, delivery particles equipped with base editors. We engineered the λ tail to ensure binding to surface determinants consistently expressed by *E. coli* in the gut environment, thereby enabling delivery to most of a target population without resorting to selection. Editing a gene in situ bypasses the need to remove the target bacterium and replace it by a genetically modified strain, something challenging to achieve without imposing strong perturbation of the environment. Previous work aiming to deliver a transgene to bacteria colonizing the mouse gut using phages achieved delivery to only a fraction of the target population^29^ or necessitated the use of antibiotics^28^ to kill bacteria that did not receive the payload. Our strategy avoids the perturbations imposed by such methods, which is desirable for both research and potential therapeutic applications.

Our engineering efforts focused on the construction of λ particles with chimeric STF and tail tip gpJ proteins. Because no single λ tail variant was found that allows delivery to all *E. coli* strains, targeting of new strains thus requires the identification of suitable receptor-binding domains. Here we engineered four STF and two gpJ chimeras, achieving efficient delivery to three *E. coli* strains and one *K. pneumoniae*. Although these species are known pathobionts, they typically colonize the healthy human gut at low relative abundance and more work is needed to adapt our strategy to target other species of the human microbiome. A first step towards this goal will be to engineer suitable delivery vectors. Our work builds on numerous previous demonstrations that chimeric variants of phage receptor-binding proteins can be engineered to modify phage host range^61–64^. We anticipate that such modular swapping of domains will allow targeting of various Enterobacteriaceae with engineered λ-derived particles. Reaching more distant bacterial species will probably require the identification of an adequate phage chassis, as well as the availability of basic genetic elements to replicate the engineering efforts described here. Several recent studies have established genetic engineering tools for diverse microbiome species, opening the possibility of adapting our strategy to a broad range of bacteria^65–68^.

An important consideration for the future translation of our approach is the MOI needed to achieve efficient delivery to bacteria in the gut environment. The production of a single dose of 10^10^ particles, suitable for our mouse model, required an initial culture of around 5 ml (Supplementary Table 1). This can be extrapolated to a culture of roughly 5 l for a single human dose of 10^13^ particles, showing the viability of our approach (Supplementary Table 2).

We demonstrated the efficient modification of eight target genes in two species and four strains. Whereas not all mutations of interest are achievable with base editors, our delivery strategy can be readily adapted to any gene editor, such as prime editors or RNA-guided transposons^69^, with the potential to achieve different types of modification. The natural capacity of λ virions to package 48.5 kb ensures that payload size limitations are not a concern^70^.

The effect of edits on bacterial fitness will be a critical consideration for many applications. Our strategy leaves a small fraction of bacteria unedited even when high efficiencies are achieved. If an edit is costly, unedited strains will outcompete the edited ones. This phenomenon could be exploited by researchers to investigate bacterial genes important for fitness in the gut environment. However, for applications necessitating the maintenance of the edited population it will be important to introduce mutations that are neutral or beneficial. Future research will also focus on developing relevant animal models to demonstrate that in situ bacterial editing can achieve a beneficial outcome for the host. Such advancements will pave the way for new microbiome-targeted therapies and deepen our understanding of bacterial gene functions in both health and disease.

## Methods

### Strains and media

The *E. coli* and λ prophage genomes were engineered using a strategy derived from λ red homologous recombination coupled to Cas9 targeting^71^. Packaged cosmids were produced using an engineered *E. coli* K-12 strain carrying the thermosensitive cI857 λ prophage with its *cos* site deleted. When needed, a constitutively expressed SrpR repressor^45^ was inserted in the *lacZ* locus to repress the expression of genes in the production strain. The chimeric *stf* genes (λ-P2, λ-K1F, λ-K5 and λ-KL106) were expressed on a plasmid in *trans* (p938, p2292, p2058 and p1806), with the *stf* gene deleted from the prophage. All experiments were performed with cells grown in lysogeny broth plus 5 mM CaCl_2_, supplemented with antibiotics when necessary (chloramphenicol 12.5–25 µg ml^−1^, kanamycin 25–50 µg ml^−1^, trimethoprim 5–10 µg ml^−1^, streptomycin 50–100 µg ml^−1^, ampicillin 50–100 µg ml^−1^ and carbenicillin 50–100 µg ml^−1^). DAPG (100 µM, Santa Cruz Biotechnology) was added to medium or plates to induce the pPhlF promoter^45^. The *csgA-StrepTag* translation levels were analysed using YESCA agar plates (1 g l^−1^ yeast extract, 10 g l^−1^ casamino acids and 20 g l^−1^ agar in water) + 4% DMSO (v/v).

All titration assays were carried out with an *E. coli* MG1655 strain (NCBI, no. NC_000913) modified to encode the EDL933 OmpC variant, if necessary (s14269), and carrying a plasmid expressing the primase gene (p1321) if needed. mCherry (GenBank, no. QQM12952) and β-lactamase (GenBank, no. ANG10794) were used as reporter proteins encoded on the *E. coli* MG1655 genome (MG1655-*mCherry* and MG1655-*bla*). For chimeric gpJ/receptor analysis, plasmid stability assays and delivery efficiency assays, superfolder GFP (GenBank, no. AYN72676) was used as a reporter. *E. coli* MG1655 strains carrying the OmpC EDL933 receptor and *bla* gene were used for the primase experiments (s14269 and MG1655-*bla*). The clinical *K. pneumoniae* ST258 isolate was obtained from the International Health Management Associates (no. 1445327). Strain UTI89 is an extra-intestinal pathogenic strain isolated from a patient suffering from a urinary tract infection^53^; strain TN03 is a human pathogenic strain isolated from a human stool sample at Hôpital Tenon (Paris, France), and the genomic sequence was obtained by shotgun sequencing (NCBI, BioSample no. SAMN12782170)^48^ and PacBio sequencing (Sequel II, Centre for Genomic Research Liverpool, UK).

Streptomycin-resistant mutants were selected on M9 minimal medium plates supplemented with streptomycin overnight at 37 °C, and the *rpsL* gene was sequenced. *E. coli* strains s14269 (rpsL K42R) and TN03 (rpsL K42Q) were used for in vivo experiments (s21052 and s21476). Strain genotypes are listed in Supplementary Table 3.

### Cloning and plasmid construction

Chemically competent DH10B cells (Thermo Scientific) or a modified K-12 strain carrying the SrpR repressor^45^ were used in cloning procedures. Molecular cloning was carried out using Gibson Assembly^72^. Base editors ABE8e^42^ and evoAPOBEC1-nCas9-UGI^43^ were codon optimized for *E. coli* and synthesized (Twist Bioscience). The obtained DNA fragments were assembled and cloned downstream of the pSrpR promoter for all constructs.

For generation of gpJ variants the insertion point was defined as one of the last stretches of amino acid identity between naturally occurring gpJ proteins and λ gpJ (amino acid position 964 in the λ gpJ protein, Uniprot accession no. P03749). A8 and 1A2 gpJ were amplified from the genome of *E. coli* strains in a private collection (see sequence in Supplementary Information). Sequences for the λ-P2 STF chimera were amplified from phage P2 protein H (amino acid position 251 onwards, Uniprot accession no. P26700) and phage λ genomic DNA (λ STF, amino acid positions 1–393, Uniprot accession no. P03764) and cloned downstream of the inducible pPhlF promoter. The sequence for the λ-K5 chimera was obtained by fusion of λ STF (positions 1–528, Uniprot accession no. P03764) to Genbank no. YP_009787294.1 (amino acid positions 211–272), followed by GenBank no. CAA71133.2 (amino acid position 62 onwards). The latter was codon optimized to remove rare codons and synthesized (Twist Bioscience). The λ-KL106 chimera was obtained by fusion of λ STF with the STF from a prophage identified in a KL106 *K. pneumoniae ST258* strain. The K1F STF sequence was codon optimized to remove rare codons and synthesized based on gene 17 of GenBank entry no. NC_007636.1. K1F, K5 and KL106 STFs were all fused at amino acid position S528 of the λ STF; the STF P2 was fused at amino acid position R393 of the λ STF. The DNA sequences of chimeric gpJ and stf genes are provided in Supplementary Information.

The primase gene from the *E. coli* CFT073 strain (NCBI, no. AAN79964, locus AE016759_238) was amplified from the CFT073 genome and cloned into plasmid p2076 or p1321 (constitutive and DAPG-inducible promoter, respectively). The cohesive end site (*cos*) of the λ genome was cloned onto plasmids designed to be packed into λ cosmid particles (Supplementary Table 4). The 23 OmpC variants were amplified from bacterial genomes and cloned onto a pSC101 vector carrying a kanamycin resistance cassette. All plasmids were purified using the Plasmid DNA Miniprep Kit (Omega Bio-Tek) and sequence verified by Sanger sequencing (Eurofins Genomics). Plasmids, gRNAs and oligonucleotide sequences are listed in Supplementary Tables 4–6. A plasmid map of the non-replicative cosmid encoding the adenine base editor and a guide RNA is depicted in Supplementary Fig. 10.

### Packaged cosmid production

Packaged cosmid production was performed with an engineered *E. coli* K-12 strain derived from CY2120 (ref. ^41^) carrying a modified λ prophage (CY-1A2, CY-A8 or CY-Ur-λ; Supplementary Table 3). For the packaged cosmids shown in Fig. 1b and Extended Data Figs. 1 and 8a, the production strains contained plasmids p513 and p938; for the packaged cosmids in Fig. 1c and Extended Data Fig. 4, the production strains carried plasmids p1396 and p938 or p2327 and p938; for the packaged cosmids in Fig. 2b,c, the production strains contained plasmids p1324 and p1321; for the packaged cosmids in Figs. 2d and 3, the production strains carried plasmids p2328, p938 and p2076; for the packaged cosmids in Extended Data Fig. 2, the production strains contained plasmid p513, as well as the plasmid p938 where indicated; for the packaged cosmids in Extended Data Fig. 8b,c, the production strains contained plasmids p2074 and p2058; for the packaged cosmids in Extended Data Fig. 8d, the production strains contained plasmids p2075 and p2292 or p1806. All plasmids are listed in Supplementary Table 4.

Production strains were cultured in lysogeny broth medium supplemented with 5 mM CaCl_2_, and appropriate antibiotics and 100 µM DAPG if necessary, to induce λ-P2 STF or λ-K5 STF expression or primase expression. Production strains were grown overnight at 30 °C in liquid medium in an orbital shaker, diluted 1:6 the following day in fresh medium supplemented with antibiotics and DAPG when needed, and grown for 30 min at 30 °C. Packaged cosmid production was heat induced at 42 °C for 45 min. Next, cell cultures were shifted to 37 °C for 3–6 h in an orbital shaker (New Brunswick Innova 44) at 180 rpm. Samples were centrifuged for 10 min at 4,500*g* and cell pellets were resuspended and lysed with B-PER reagent (Thermo Scientific; 1/10 of the initial volume of cosmid production for in vitro assays and 1/50 for in vivo assays) and lysozyme (100 µg ml^−1^, Applichem Lifescience). DENARASE (×10,000, c-LEcta) was added to the reaction to degrade residual DNA and RNA at a dilution of 1:10,000. Bio-Beads (SM-2 resin, Bio-Rad) were added to the lysis reaction and samples incubated for 1 h in a mini-shaker (PS-3D, Grant-Bio) at room temperature. Samples were centrifuged for 5 min at 16,000*g* and supernatants sterile filtered (0.22 µm pore size, Sartorius Minisart). For in vivo administration, packaged cosmids were concentrated and buffer exchanged against PBS by tangential flow filtration (MWCO 100 kDa, Sartorius Vivaflow 200). Packaged cosmid concentration was analysed by *E. coli* transduction with diluted cosmid stocks (1:10 dilution) and consecutive colony counting on chloramphenicol plates following overnight incubation at 37 °C. An initial culture volume of roughly 4.8 ml was needed to obtain an intermediate dose of 10^10^ particles used per mouse, as shown in Fig. 5 (see calculation in Supplementary Table 1).

### Analysis of OmpC variants in *E. coli*

Seventy-six unique OmpC protein sequences were extracted from 525 *E. coli* genome assemblies using TBLASTN^73^ v.2.11.0 with a curated set of OmpC sequences as query. The sequences were aligned using MAFFT^74^ v.7.520 with default parameters and with the option --treeout to export the guide tree. The multiple sequence alignment and corresponding guide tree are provided in Supplementary Data 10 and Supplementary Fig. 1.

### Packaged cosmid delivery efficiency

Delivery efficiency was analysed using cosmid particles equipped with λ-P2 STF, λ-K1F STF, λ-K5 STF or λ-KL106 STF chimeras and gpJ 1A2 or A8 into strain s14269, UTI89, TN03 or *K. pneumoniae ST258*, respectively. Cosmids carrying λ-P2 STF and gpJ A8 or 1A2 were also used for analysis of delivery efficiency into a series of MG1655-*ΔLamB-ΔompC* strains carrying different OmpC variants on a plasmid. The cosmid encodes a *sfGFP*, *mCherry* or *venus* gene under a constitutive promoter. Cells were grown in lysogeny broth supplemented with 5 mM CaCl_2_ to an optical density (OD_600_) of 0.2–0.6. Cell density was adjusted to OD_600_ = 0.025 in fresh lysogeny broth supplemented with 5 mM CaCl_2_, and 90 µl of cell culture was mixed with 10 µl of each cosmid serially diluted in lysogeny broth plus 5 mM CaCl_2_ (1:3 dilution) to reach different MOIs. The samples were incubated for 45 min at 37 °C, and 8 µl was added to 250 µl of ice-cold PBS plus 1 mg ml^−1^ kanamycin before analysis by flow cytometry (*sfGFP* and *venus*: excitation, 488 nm; emission, 530/30 BP; *mCherry*: excitation, 561 nm; emission, 620/15 BP; Attune NxT, Thermo Scientific). The gating strategy is described in Supplementary Fig. 11.

### Plasmid stability assay

Plasmid stability was investigated in vitro with a time-course assay. *E. coli* MG1655 carrying a DAPG-inducible primase plasmid (p1321) with or without 100 µM DAPG was grown to an OD_600_ of 0.2–0.6 in lysogeny broth plus 5 mM CaCl_2_ and 50 µg ml^−1^ kanamycin. Samples were then diluted to an OD_600_ of 0.01 in fresh lysogeny broth plus 5 mM CaCl_2_ and 50 µg ml^−1^ kanamycin plus/minus 100 µM DAPG, treated with a packaged cosmid harbouring the *sfGFP* gene and the conditional primase origin of replication (p1324) at an approximate MOI of 40, and subsequently incubated in an orbital shaker at 37 °C. Samples (1–5 µl) were taken at different time points, mixed with 250 µl of ice-cold PBS supplemented with 1 mg ml^−1^ kanamycin and analysed in a flow cytometer (excitation, 488 nm; emission, 530/30 BP; Attune NxT, Thermo Scientific). To maintain cells in the exponential growth phase, samples were diluted 1:5 into fresh lysogeny broth medium supplemented with 50 µg ml^−1^ kanamycin plus/minus 100 µM DAPG every 2 h.

### Base editing in vitro

The *E. coli* MG1655-*mCherry* strain was transformed with base editor payload p2316 or p2326, grown for 2 h in SOC medium (30 °C, 180 rpm) and selected on chloramphenicol plates overnight at 30 °C. Forty-eight individual colonies were resuspended in 250 µl of PBS supplemented with 1 mg ml^−1^ kanamycin in a 96-well plate, and mCherry fluorescence was measured by flow cytometry (excitation, 561 nm; emission, 620/15 BP; Attune NxT, Thermo Scientific). As a control, base editors were transformed with a non-targeting gRNA (*SapI* spacer) and the mCherry fluorescence of two colonies was analysed. The target region of mCherry was amplified from a minimum of three individual colonies per experiment, and PCR products were sequenced to confirm base editing.

The *E. coli* strain MG1655-*bla* transformed with base editor payload p1396 or p2327 was grown for 2 h in SOC medium (30 °C, 180 rpm) before spotting of 10 µl of individual cell dilutions on chloramphenicol/carbenicillin plates, as well as on chloramphenicol plates. As a control, base editors were transformed with a non-targeting gRNA (*SapI* spacer) on the payload. Editing efficiency was analysed by colony counting on plates following overnight incubation at 30 °C. The target site of *bla* was amplified from a minimum of three individual colonies, and PCR products were sequenced to confirm targeted base editing.

Base editing using packaged cosmids in *E. coli* strains MG1655-*bla*, UTI89 and TN03 or *K. pneumoniae* ST258 was performed similarly to transformation assays. The target strain was cultured to mid-log phase, diluted to an OD_600_ of either 0.025 (Figs. 1c and 4) or 0.005 (Fig. 2d) and transduced with serial dilutions (1:1 or 1:2) of the packaged cosmid yielded in a 96-well plate. Cells were grown for 2 h in lysogeny broth medium supplemented with 5 mM CaCl_2_ (30 or 37 °C, 180 rpm) before spotting of individual dilutions on either lysogeny broth plates or lysogeny broth + carbenicillin plates. As a control, cells were treated with lysogeny broth medium rather than packaged cosmid solution. For all experiments the target gene was amplified via PCR from the genome of individual colonies and base editing was confirmed by Sanger sequencing. For the UTI89, TN03 and *K. pneumoniae* ST258 experiments, the target gene was amplified via PCR from the genome of cell populations (more than 10^5^ cells) and base-editing efficiencies from Sanger sequencing data were analysed using the tool EditR^75^.

### In situ editing of *E. coli* in the mouse gut

Specific-pathogen-free 5–9-week-old female BALB/cYJ mice were supplied by Charles River Laboratories and housed in an animal facility in accordance with Institut Pasteur’s guidelines and European recommendations. Animal procedures were approved by Institut Pasteur (approval ID: 20040) and the French Research Ministry (APAFIS ID: 28717), and animal experiments were performed in compliance with applicable ethical regulations. Water and food were provided ad libitum. Animals were randomly assigned into cages upon reception. After acclimatation, cages where randomly assigned to treatment groups.

Animals were acclimated for 5 days before the addition of streptomycin sulfate (5 mg ml^−1^, Sigma-Aldrich, no. S9137) to autoclaved drinking water to decrease the number of facultative aerobic/anaerobic resident bacteria^76^. Drinking water containing streptomycin was prepared fresh weekly. Three days later (D0), mice were orally gavaged with approximately 1 × 10^8 ^CFU of strain s21052 or s21476, grown overnight in lysogeny broth and resuspended in 200 µl of sterile gavage buffer (20% sucrose, 2.6% sodium bicarbonate, pH 8.0). Starting at D5, mice were orally administered (200 µl per mouse) with either gavage buffer or packaged cosmids diluted 1:1 in buffer. The appropriate dose was achieved by diluting the packaged cosmid suspension in PBS before formulation in buffer, and checked by *E. coli* transduction.

### Metagenomic analysis

For 16S analysis, faecal samples were collected from individual mice at D4, D5 (immediately before oral gavage with packaged cosmids), D6, D9 and D12 of colonization for all mice that received 1 × 10^10^ tu per animal, and samples were immediately frozen at −80 °C to preserve microbial diversity. Total microbial faecal DNA was extracted using a QiaAMP Fast DNA Stool Mini Kit (Qiagen) following the manufacturer’s instructions and sent for sequencing (Illumina NovaSeq, 250 base pair, paired-end reads, Novogene).

The obtained paired-end reads were filtered by length (cutadapt v.3.3 (ref. ^73^); --minimum-length parameter set to 20), merged (FLASH^77^ v.1.2.11, parameters --min-overlap 10 --max-mismatch-density 0.2) and filtered by quality (fastp^78^ v.0.23.1; parameters -q 19 -u 15). Subsequently chimeric sequences were removed using vsearch^79^ v.2.16.0 with the Silva reference database^80^. At this point, one sample (D5 M36) had to be excluded from subsequent analysis due to low sequencing depth (11,499 non-chimeric reads). Sequences were clustered into operational taxonomic units at a 97% similarity threshold with Uparse^81^ v.7.0.1001 and annotated with taxonomy information using QIIME^82^ v.1.9.1 and the Silva database. The QIIME toolkit was also used for both taxonomic profiling of samples and obtaining beta-diversity values. The phylogenetic tree supplied to UniFrac distance computation^83^ was generated using MUSCLE^84^ 3.8.1551 (-maxiters parameter set to 2). Microbiome composition at different phylogenetic levels is provided in Supplementary Data 3–9.

### Evaluation of base-editing efficiency from mouse faeces by direct plating

Fresh faecal samples were collected at D0 and subsequent relevant time points as a proxy for assessment of intestinal colonization levels of s21052. In brief, samples were weighed on an analytical balance and 1 ml of PBS added. Samples were incubated for 2 min at room temperature and suspended by manual mixing and vortexing. Serial dilutions were performed in PBS, 5 μl of each dilution was spotted onto Drigalski agar plates (Bio-Rad) supplemented with 100 µg ml^−1^ streptomycin and plates were incubated overnight at 37 °C. Estimation of editing efficacy was performed the following day by repatching individual colonies (up to 12 colonies per mouse and per time point) onto agar plates, with or without 50 µg ml^−1^ carbenicillin, to investigate loss of resistance to β-lactams subsequent to editing of the *bla* gene (Extended Data Fig. 5). In addition, separate faecal samples were collected and frozen at −80 °C within 1 h of collection to assess editing efficacy by ddPCR.

### Evaluation of base-editing efficiency by ddPCR

Primers F3 (5′-GGATCTCAACAGCGGTAAG-3′) and R3 (5′-GGCATCAACACGGGATAATA-3′), both with a melting temperature of 61 °C, were designed to amplify a 112-base-pair region of the *bla* gene in *E. coli* s21052 spanning the target site for base editing. Two Taqman probes were designed to bind this amplicon with the target site towards the middle of the probes, before or after successful editing (**A** to **G**): P1 (5′-FAM-CT+TT+T**+A**+AA+GTT+C+T+GC-3′) and P2 (5′-HEX-CT+TT+T**+G**+AAGTT+CT+GC-3′). Each probe contained a different fluorophore (FAM or HEX), as well as carefully positioned locked nucleic acid bases (symbolized by base A, T, C or G preceded by a + sign in the sequences above). Locked nucleic acid nucleotides allow for a greater melting temperature (Tm) difference between matching and mismatched probes while retaining small probe size, further improving discrimination^82^. Tm for either probe matching its specific sequence was predicted to be 66 °C, compared with 55 °C in the case of binding to the non-matching sequence (OligoAnalyzer Tool, IDT).

Reactions were conducted in a final volume of 8 µl with PerfeCTa Multiplex qPCR Toughmix, 100 nM fluorescein, 250 nM of each primer and 250 nM of each probe using a Naica ddPCR system (Stilla Technologies). The following two-step cycling programme was applied: initial denaturation for 3 min at 95 °C, followed by 50 cycles at 95 °C for 10 s and 57 °C for 30 s. This Taqman assay was validated for specificity using purified gDNA from overnight bacterial cultures of either wild-type s21052 or in vitro-edited s21052 (Supplementary Fig. 4), and fluorescence spillover compensation was carried out using the appropriate control reactions following the manufacturer’s recommendations.

A similar strategy was used to quantify the editing of the *csgA* gene in TN03, with primers F5 (5′-GCGTGACACAACGTTAATTTCCATTC-3′) and R5 (5′-AGAGCGCTACCGGAGAATACG-3′) and probes P3 (5′-FAM-AC+A+T+GAA+A+CT+T+TTAAAA+G+T+A+GC-3′) and P4 (5′-HEX-AC+A+C+GAA+A+CT+TT+TAAAA+GT+A+GC-3′).

Stool samples collected from mice were weighed, resuspended at 100 mg ml^−1^ in ultrapure water, homogenized and heat treated at 98 °C for 10 min. Following brief vortexing and 1 min cooling at room temperature, supernatant was pipetted from the top of the suspension to avoid major debris, diluted at least ten times in ultrapure water and analysed immediately by ddPCR without further processing.

### Base-editing off-target analysis

Target strain MG1655-*bla* or TN03 was cultured to mid-log phase, diluted to an OD_600_ of 0.005 and mixed with serial dilutions (1:2) of the yielded packaged cosmid in a 96-well plate. Cells were incubated for 2 h (30 °C, 180 rpm), serially diluted and 10 µl spotted on lysogeny broth plates followed by incubation overnight at 30 °C. As a control, cells were treated with lysogeny broth medium supplemented with 5 mM CaCl_2_ rather than packaged cosmid solution. The following day, cells were recovered from different spots (over 10^5^ cells per spot) corresponding to different MOIs and the *csgA* gene was amplified by PCR from the genome. Base editing of *csgA* was analysed by Sanger sequencing; meanwhile, plates and samples were stored at 4 °C. For downstream next-generation sequencing analysis we selected samples based on our Sanger sequencing results. We selected samples that were treated with the lowest MOI (roughly 227 (S1) and roughly 195 (S2) for the MG1655-*bla* experiment, and with MOI of about 51 (S1 and S2) for the TN03-*csgA* experiment) in which no wild-type peak at the target site was detectable on the chromatogram. Both treated and control samples were grown for 2–3 h from these two independent experiments (S1 and S2) and gDNA was extracted (Wizard Genomic DNA Purification Kit, Promega). Sample concentrations were measured and dilutions sent for next-generation sequencing (Illumina PE150, Eurofins). Samples were sequenced at an average genome-wide coverage greater than 6,000.

The program fastp v.0.23.2 (ref. ^75^) was used for quality control of raw reads. Because multiple libraries were sequenced to obtain the desired depth, all reads passing quality control from the same sample were merged into a single file. The *E.* *coli* MG1655 complete reference genome was downloaded from NCBI (nuccore accession no. NC_000913.3), manually modified to reflect genetic modifications to the wild-type strain and further corrected using breseq^85^ v.0.37.1 with reads from control sample S1. For TN03 (NCBI assembly accession no. GCA_015186165.1), the complete reference genome obtained from a previous PacBio sequencing run of the same strain was used. The versions of both reference genomes used in the following analyses are available at https://github.com/Eligo-Bioscience/in-situ-targeted-base-editing-of-gut-bacteria-in-mice. The four readsets (two repeats for base-edited samples ABE S1 and ABE S2 and two for controls S1 and S2) corresponding to each of the two strains were aligned against their respective reference genome using bwa^86^ v.0.7.17-r1188 (mem algorithm, default parameters). For each position of the reference genome, the frequency of each nucleotide was computed with a custom Python script using the pile-up function of pysam v.0.20.0 (ref. ^87^). The ‘read mismatch frequency’ was calculated as the ratio between the number of aligned reads differing from the reference and total alignment coverage at each position.

Figures 2e and 4d and Supplementary Figs. 2, 3, 8 and 9 were generated using the libraries Matplotlib and Seaborn. The merged readsets passing quality control for each sample are available on NCBI SRA (Bioproject, no. PRJNA944658). The predicted off-target sites (positions in the reference genomes with up to seven mismatches in the target sequence, including up to two in the ten PAM-proximal nucleotides) corresponding to individual off-target IDs for MG1655-*bla* or TN03 are listed in Supplementary Data 1 and 2. The code used for the analysis and generation of the figures is available at https://github.com/Eligo-Bioscience/in-situ-targeted-base-editing-of-gut-bacteria-in-mice.

### In vivo competition assay

To ensure that both edited and unedited clones used in the competition assay were as close as possible to each other, we performed an in vitro base-editing experiment using *E. coli* strains s21052 and s21476 and selected sequence-confirmed edited and unedited clones treated in the same manner. For each *E. coli* strain, five groups of three mice were conditioned with streptomycin and orally gavaged with independent pairs of clones (wild type and edited) at an approximate ratio of 1:1. Stool samples were collected regularly for up to 29 days, and bacterial colonization levels were measured by plating resuspended stool samples onto selective agar plates. Duplicate stool samples were also collected and frozen, and the ratio of edited to wild-type clones was later estimated by ddPCR using the assays described above.

### Protein translation analysis of the base-edited *csgA* gene

A StrepTag (NWSHPQFEK) was fused to the C-terminal site of the *csgA* gene (roughly 16 kDa) in strain TN03 and the genomic insertion was confirmed by sequencing. Base editing of this strain was performed as described in ‘Base editing in vitro’. Twelve single colonies from the highest MOI were picked, resuspended in 40 µl of PBS and serial dilutions were spotted on lysogeny broth plates. The following day, the target gene was amplified from single colonies by PCR from the genome of individual colonies to confirm targeted base editing by Sanger sequencing. Strain *BE* *1bp* carried a base edit at the target site 6A (start codon) in the editing window, whereas strain *BE 2bp* carried the target mutation 6A as well as a bystander mutation at position 10A located upstream of the *csgA* start codon in the editing window. Base-edited strains carrying one or two base pair mutations at the target region were grown in lysogeny broth and stored at −80 °C. The two base-edited strains, as well as the unedited strain (TN03-*csgA-StrepTag*), were plated on YESCA agar plates + 4% DMSO (v/v) and incubated at 26 °C for 72 h (curli-inducing conditions^88^).

Translation of the cell-associated curli protein CsgA was investigated by dot-blot analysis. The lawn from different strains was scooped and resuspended in water and OD_600_ was adjusted to 2.0. Cells were pelleted and the supernatant removed. Each pellet was treated with 100 µl of hexafluoroisopropanol and incubated for 1 h at room temperature to dissociate curli subunits. Hexafluoroisopropanol was removed by overnight incubation of open Eppendorf tubes under a fume hood at room temperature. Samples were resuspended in 75 µl of 1× Bolt LDS Sample buffer (Thermo Scientific) supplemented with 2% beta-mercaptoethanol. Samples were incubated for 10 min at 95 °C, and 5 µl of each sample was blotted on a nitrocellulose membrane (0.2 µm, Thermo Scientific). Dots were allowed to dry under a laminar flow hood for 20 min at room temperature. The blot was blocked by incubation with 5% skimmed milk in 0.1% PBS-Tween 20. Either mouse anti-StrepTag (BioLegend) at 0.5 µg ml^−1^ or mouse anti-GAPDH (Thermo Scientific) at 1 µg ml^−1^ was used as primary antibody, with incubation overnight at 4 °C. As a secondary antibody, horseradish peroxidase-conjugated anti-mouse antibody (Invitrogen) was used at 0.2 µg ml^−1^ and the blot was incubated for 1.5 h at room temperature. SuperSignal West PICO PLUS Chemiluminescent Substrate (Thermo Scientific) was used for detection.

### Payload delivery into faecal samples

To investigate the ability of our vectors to deliver to bacteria in the mouse gut, faeces were collected from mice that had either (1) undergone streptomycin conditioning only, (2) undergone streptomycin conditioning followed by colonization with a streptomycin-resistant MG1655 strain or (3) been left untreated. Faeces were resuspended in PBS at 1 mg ml^−1^, and 180 µl aliquots were treated with 20 µl of four different λ-derived vectors carrying a payload with a chloramphenicol resistance marker and harbouring different gpJ and STF chimeras (A8-P2, A8-K5, 1A2-P2 or 1A2-K5). Following a 3 h incubation at 37 °C, the samples were diluted in series and 150 µl (undiluted, 1:100 or 1:10,000) plated on either BHI medium or BHI medium supplemented with 24 µg ml^−1^ chloramphenicol. Plates were then incubated in an anaerobic chamber for 24 h at 37 °C (Supplementary Fig. 6).

### Delivery of DNA payloads to other bacterial species

Eight λ-derived vectors harbouring different versions of gpJ and STFs (combinations of gpJ A8 or 1A2 with STF chimeras P2, K1, K5 or KL106) were used to transduce ten different Enterobacteria species and strains and six non-enterobacterial species (Supplementary Table 3). Bacteria were grown to an OD_600_ of 0.1 and 90 µl of cells treated with 10 µl of various λ-derived vectors carrying an mCherry payload. Following 2 h of incubation, 2 µl of these reactions was resuspended in 250 µl of PBS supplemented with 1 mg ml^−1^ kanamycin in a 96-well plate, and mCherry fluorescence measured by flow cytometry (excitation, 561 nm; emission, 620/15 BP; Attune NxT, Thermo Scientific). To test for background fluorescence events, untreated bacterial samples were processed and measured as for treated samples.

### Reporting summary

Further information on research design is available in the Nature Portfolio Reporting Summary linked to this article.

## Online content

Any methods, additional references, Nature Portfolio reporting summaries, source data, extended data, supplementary information, acknowledgements, peer review information; details of author contributions and competing interests; and statements of data and code availability are available at 10.1038/s41586-024-07681-w.

### Supplementary information

Supplementary Information This file contains Supplementary Figs. 1–11, Tables 1–6, references, a list of gene sequences used in this study, DNA sequences on plasmids and genomes and a list of plasmid sequences used in this study.

Reporting Summary

Supplementary Data

### Source data

Source Data Fig. 1

Source Data Fig. 2

Source Data Fig. 3

Source Data Fig. 4

Source Data Fig. 5

Source Data Extended Data Fig. 1

Source Data Extended Data Fig. 2

Source Data Extended Data Fig. 3

Source Data Extended Data Fig. 4

Source Data Extended Data Fig. 5

Source Data Extended Data Fig. 6

Source Data Extended Data Fig. 7

Source Data Extended Data Fig. 8

Source Data Extended Data Fig. 9

Source Data Extended Data Fig. 10

Source Data Extended Data Fig. 11

Source Data Extended Data Fig. 12

## Data Availability

Sequencing reads are available on NCBI SRA (Bioproject no. PRJNA944658). Reference genomes for *E. coli* strains were downloaded from NCBI: MG1655 (nuccore accession no. NC_000913.3) and TN03 (NCBI assembly accession no. GCA_015186165.1). Source data are provided with this paper.

## Code availability

Custom scripts used for data analysis are available at https://github.com/Eligo-Bioscience/in-situ-targeted-base-editing-of-gut-bacteria-in-mice and were deposited at Zenodo (10.5281/zenodo.11198996)^89^.
