# Supplementary material for: In situ targeted base editing of bacteria in the mouse gut
Source: Nature. 2024 Jul 10;632(8026):877–84. doi: 10.1038/s41586-024-07681-w (PMC11338833; doi:10.1038/s41586-024-07681-w)
Supplement: Supplementary file 1 — This file contains Supplementary Figs. 1–11, Tables 1–6, references, a list of gene sequences used in this study, DNA sequences on plasmids and genomes and a list of plasmid sequences used in this study. [file 41586_2024_7681_MOESM1_ESM.pdf]

nature portfolio

https://doi.org/10.1038/s41586-024-07681-w

Supplementary information

In situ targeted base editing of bacteria in the mouse gut

In the format provided by the authors and unedited

Supplementary Information

In situ targeted base editing of bacteria in the mouse gut

Authors

Andreas K Brödel[1], Loïc H Charpenay[1], Matthieu Galtier[1], Fabien J Fuche[1], Rémi Terrasse[1], Chloé Poquet[1], Jan Havránek[1], Simone Pignotti[1], Matthew T N Yarnall[1], Antonina Krawczyk[1], Marion Arraou[1], Gautier Prevot[1], Dalila Spadoni[1], Edith M Hessel[1], Jesus Fernandez-Rodriguez[1], Xavier Duportet[1], David Bikard[1,2]

Shared first authorship: Andreas K Brödel, Loïc H Charpenay

Shared last authorship: Jesus Fernandez-Rodriguez, Xavier Duportet, David Bikard

Affiliation

[1] Eligo Bioscience, 75013 Paris, France.

[2] Institut Pasteur, Université Paris Cité, Synthetic Biology, 75015 Paris, France

Corresponding authors

Jesus Fernandez-Rodriguez, Xavier Duportet & David Bikard

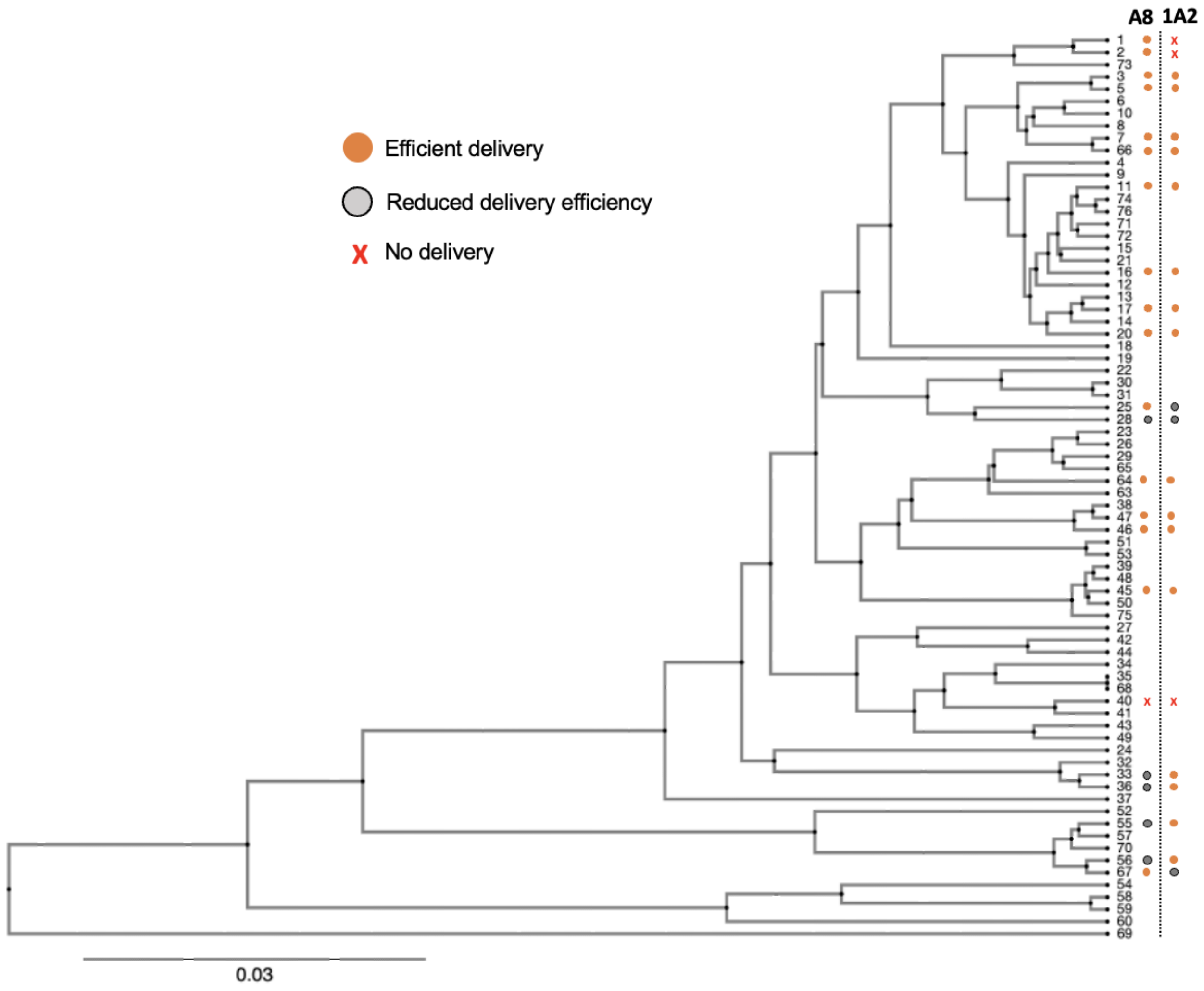

Supplementary Figure 1: Correlation of ompC variance with DNA payload delivery efficiency in *E. coli*. We identified 74 OmpC variants and experimentally tested the delivery into 23 of them with two different gpJ chimera, A8 and 1A2 (see Fig. S2). Most OmpC variants were efficiently targeted by at least one of the gpJ chimeras, demonstrating that gpJ has a broad host range. The multiple sequence alignment is provided in **Supplementary Data 10**.

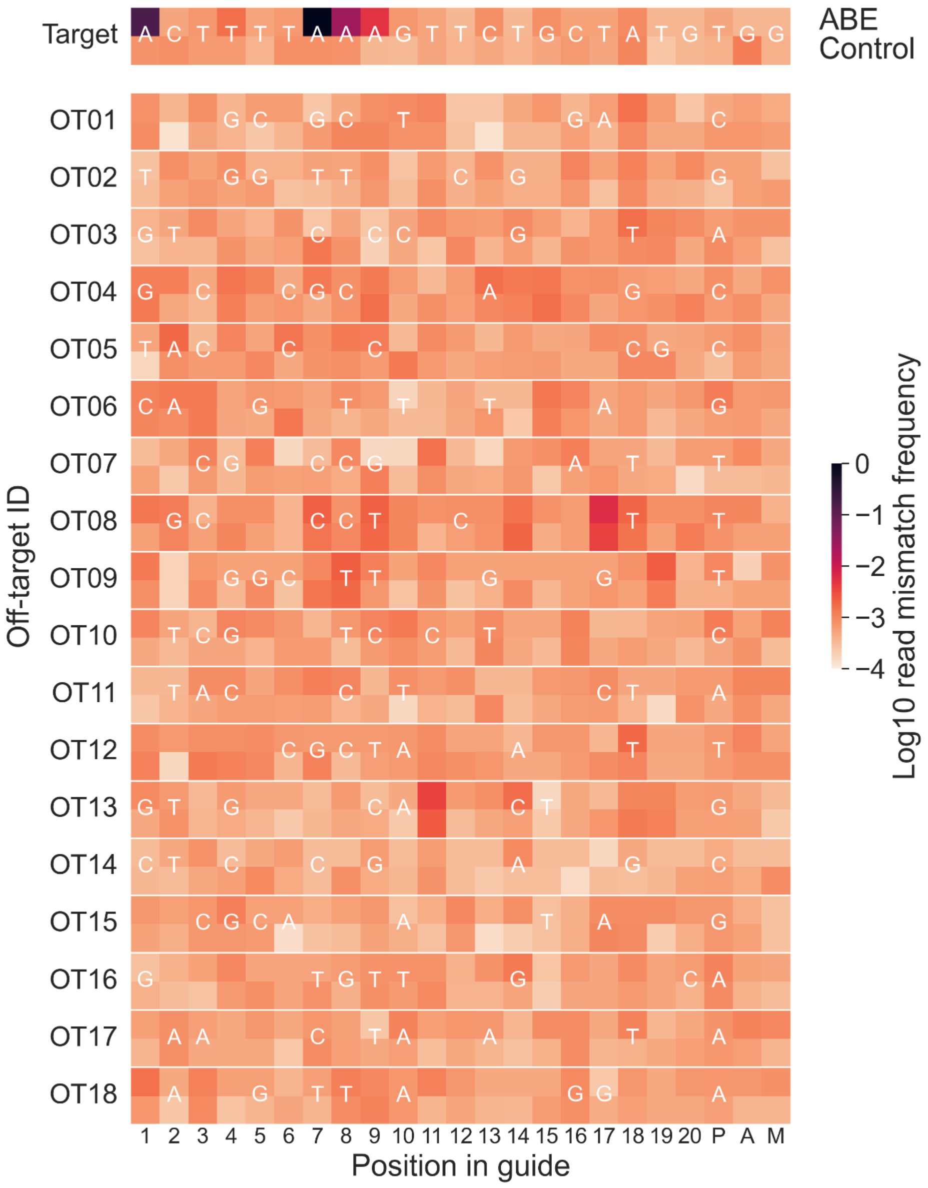

Supplementary Figure 2: Off-target analysis in MG1655-*bla*. Positions in the reference genome with up to 7 mismatches to the target sequence, up to 2 of which in the 10 PAM-proximal nucleotides, were analyzed for off-target mutations. Each row in the heatmap is a potential off-target region, and the positions with a mismatch to the target are overlaid with the mismatching nucleotide. The values correspond to the maximum Log10 of the read mismatch frequency (as defined in the Methods section) across the two controls and the two adenine base-edited samples (ABE). For comparison, the top row shows the same values but for the intended target. The off-target sites corresponding to individual off-target IDs are listed in **Supplementary Data 1**.

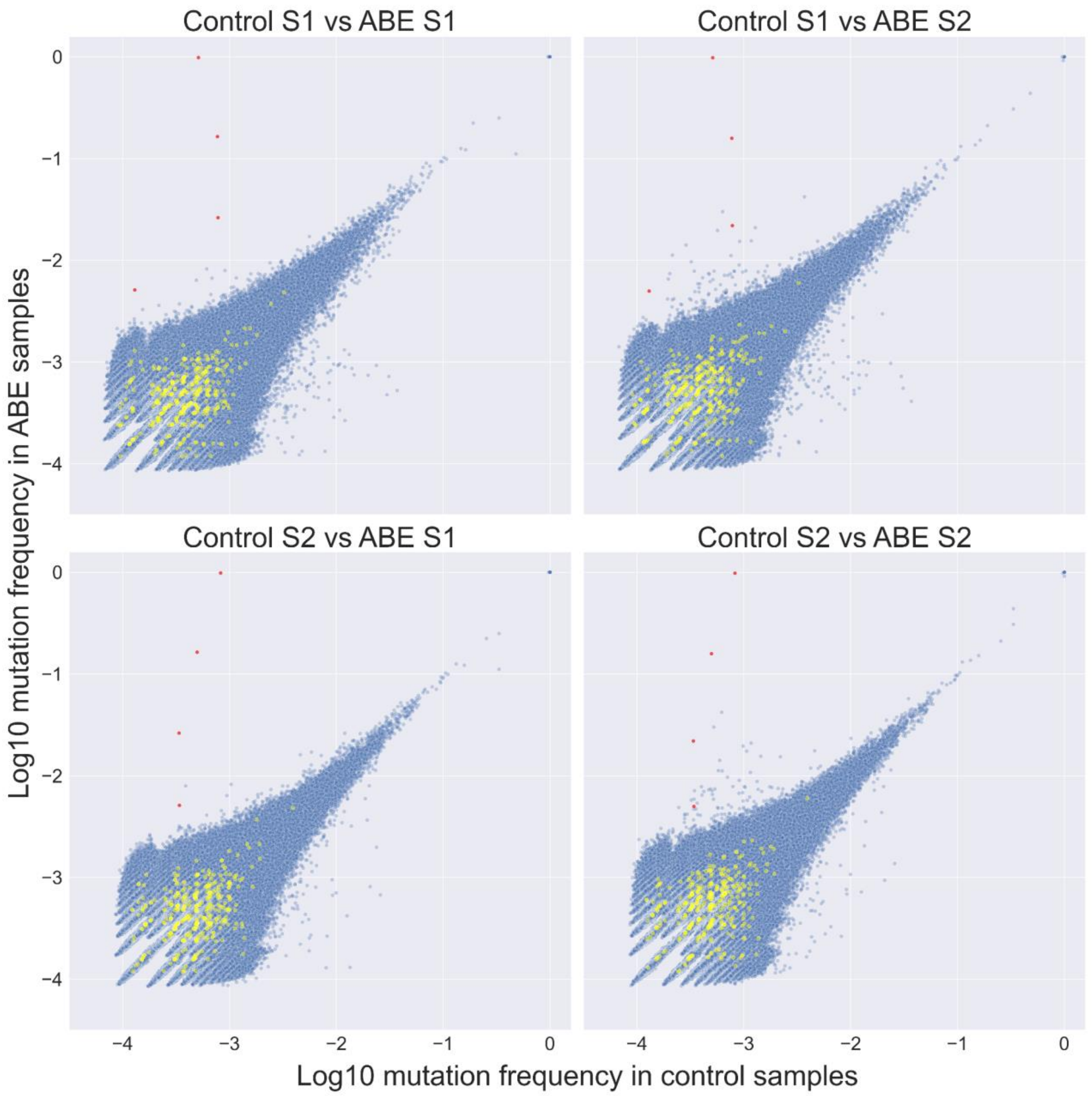

Supplementary Figure 3: Correlation of read mismatch frequency between adenine base-edited (ABE) and non-transduced control samples in MG1655-*bla*. Analysis was performed for two independent experiments (S1 and S2) for ABE-treated or control samples. On-target base editing as well as bystander mutations in the editing window are highlighted in red, while the potential off-target sites from Figure S4 are highlighted in yellow. Unlike Figure S6, all positions in the genome are included. While some positions show an increased mutation rate in ABE-treated samples, a similar effect is observed for other positions in control samples, suggesting that this is due to spurious mutations or sequencing errors rather than a consequence of the ABE treatment.

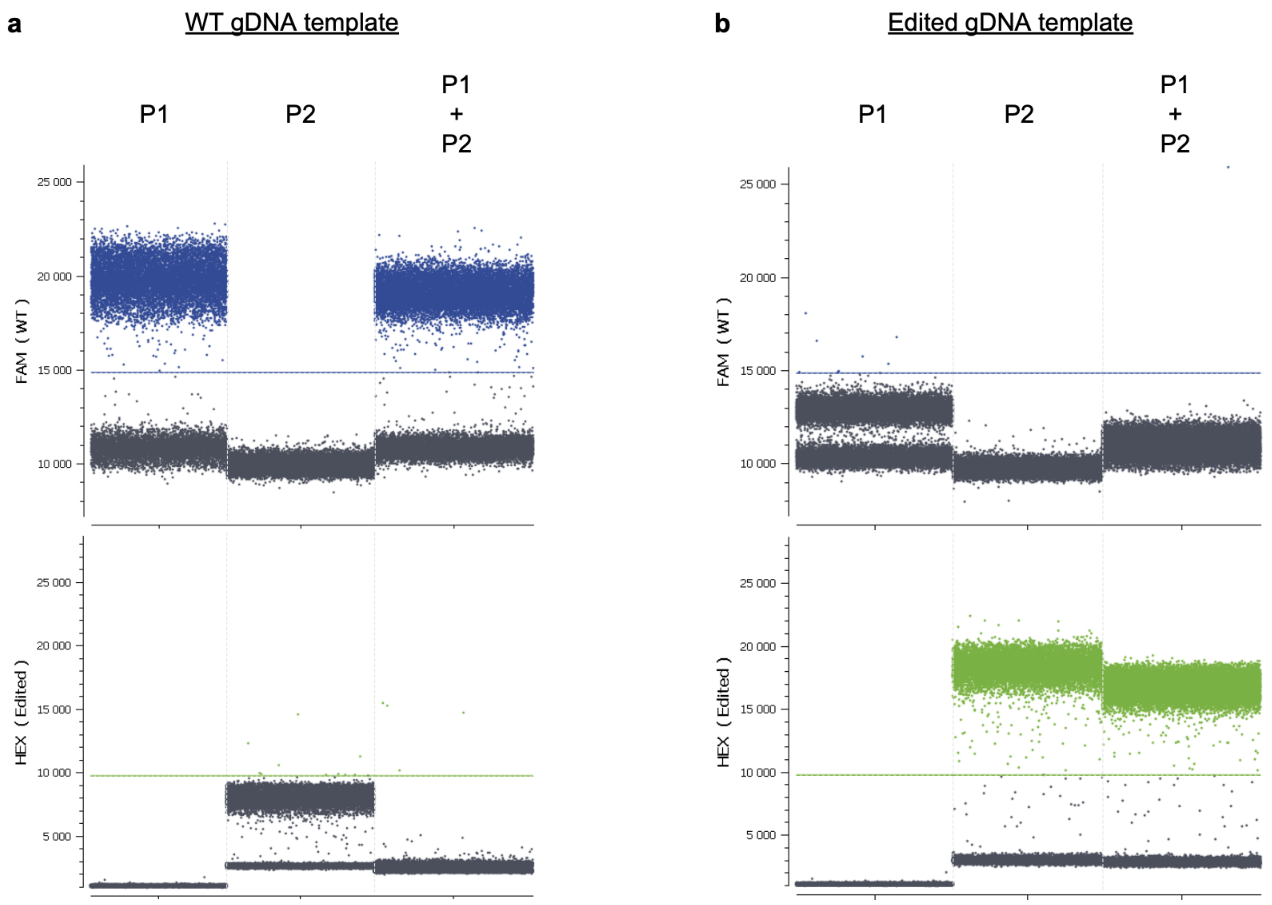

Supplementary Figure 4: Validation of ddPCR Taqman assay. a) Probes P1 (WT target) and P2 (base-edited target) were assessed for their specificity, either individually or when mixed together at a 1:1 molar ratio, using purified genomic DNA (gDNA) from wild-type (WT) and **b)** *in vitro*-edited MG1655-*bla* as template. Probe 1 contained the fluorophore FAM whereas Probe 2 carried the fluorophore HEX.. Each dot represents an individual droplet. Positive events for the WT gDNA template or the edited gDNA template are highlighted as blue or green dots, respectively.

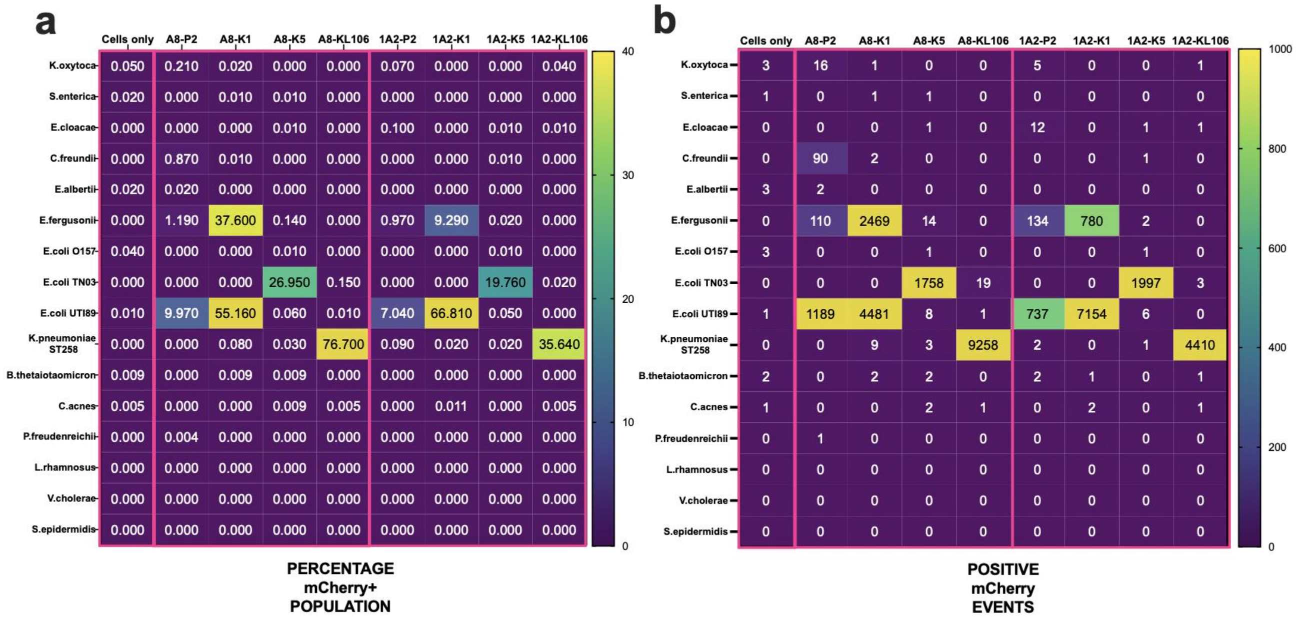

Supplementary Figure 5: λ-derived vectors are specific to *Enterobacteria* and transduction depends on bacterial surface determinants. Eight λ-derived vectors harboring different versions of gpJ and STFs (combinations of gpJ A8 or 1A2 with STF chimeras P2, K1, K5, or KL106) were used to transduce ten different *Enterobacteria* species and strains and six non-Enterobacterial species. Bacteria were grown to an OD_{600} of 0.1 and 90 µl treated with 10 µl of the different λ-derived vectors carrying an mCherry payload at an MOI range of 5-30. After 1 hour incubation, 2 µl of these reactions were resuspended in 250 µl Phosphate Buffered Saline supplemented with 1 mg ml^{-1} kanamycin in a 96-well plate and mCherry fluorescence was measured by flow cytometry (excitation: 561 nm, emission: 620/15 BP; Attune NxT Thermo Scientific. Left column "Cells only" refers to untreated bacterial samples to verify basal fluorescent levels in the absence of transduction. **a)** Percentage of mCherry-positive population after treatment. **b)** Absolute number of positive mCherry events measured. Total measured events range from 3,500 to 25,000 depending on the strain.

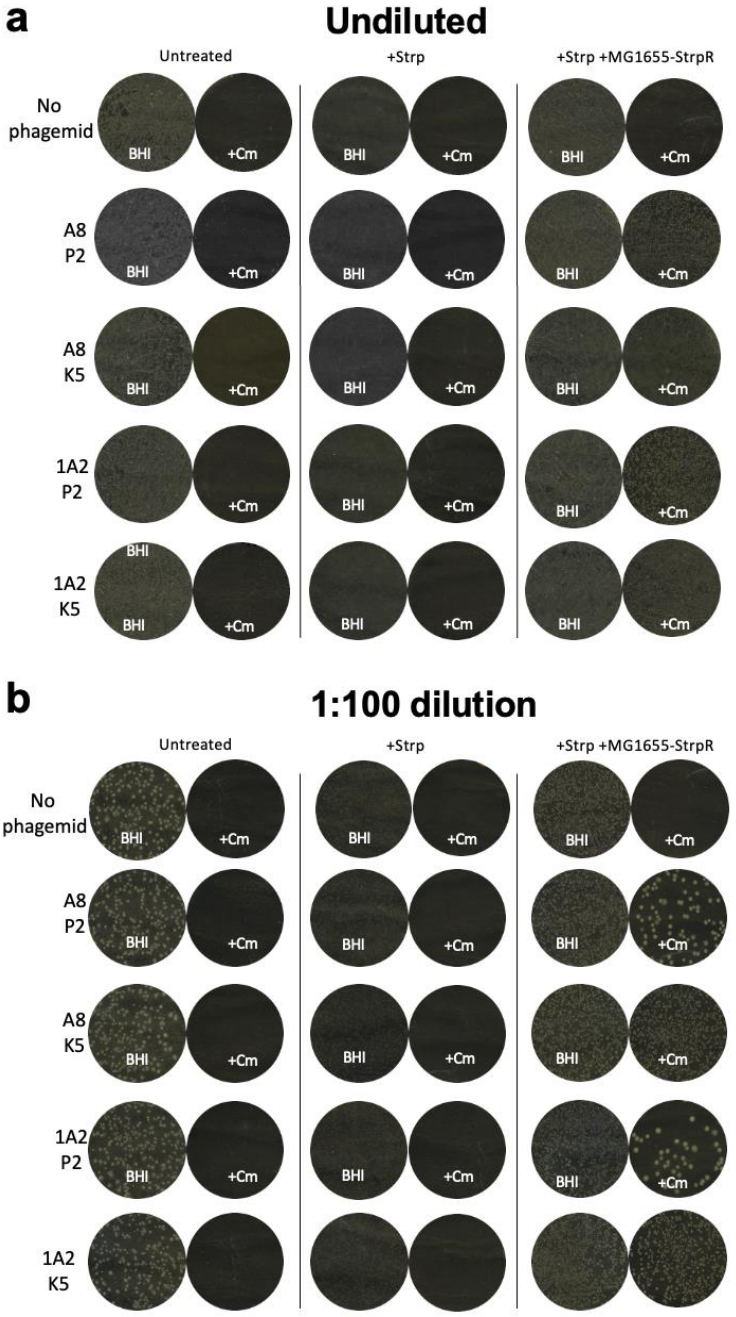

Supplementary Figure 6: Payload transduction is only detectable in mice colonized with the target *E. coli* strain. Samples were plated **a)** undiluted and **b)** after a 100-fold dilution. Mouse feces were resuspended in PBS at 1 mg ml^{-1}. 180 µl of this suspension was treated with 20 µl of λ-derived particles harboring different gpJ and STF variants (left labels: A8-P2, A8-K5 STF, 1A2-P2 STF, and 1A2-K5 STF) and a payload carrying a chloramphenicol resistance marker. After 3 hours of incubation at 37ºC in an anaerobic chamber, 150 µl were plated, at a 1:100 dilution, on BHI media or BHI media supplemented with 24 µg ml^{-1} chloramphenicol (+Cm) and incubated in an anaerobic chamber for 24 hours at 37ºC. Left column: feces from untreated mice; middle column: feces from mice treated with streptomycin; right column: feces from mice treated with streptomycin and colonized with streptomycin-resistant MG1655. Some plates show debris present in the feces samples as these samples were not filtered prior to plating.

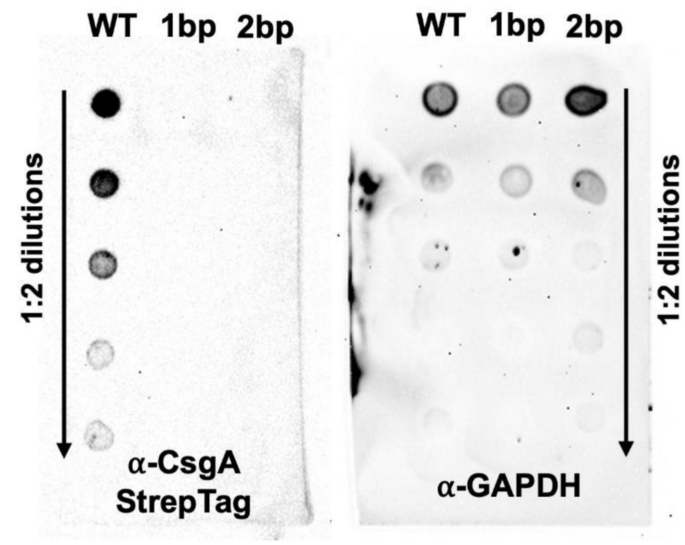

Supplementary Figure 7: Dot blot analysis of the *csgA-StrepTag* protein produced in the strain TN03-*csgA StrepTag* compared to the base-edited strains. A StrepTag was fused to the C-terminal site of the *csgA* gene on the strain TN03 (WT) and the start codon base edited using our technology. Clones obtained showed either a single mutation at the target site 6A in the editing window converting the ATG start codon to ACG (1bp), or the 6A mutation together with a bystander mutation at position 10A located upstream the *csgA* start codon in the editing window (2bp). Clones were grown on YESCA agar plates supplemented with 4% DMSO prior to sample preparation. A mouse anti-StrepTag primary antibody (left panel) or a mouse anti-glyceraldehyde-3-phosphate dehydrogenase (GAPDH) primary antibody (right panel) and a horseradish peroxidase (HRP)-conjugated anti-mouse secondary antibody were used for detection.

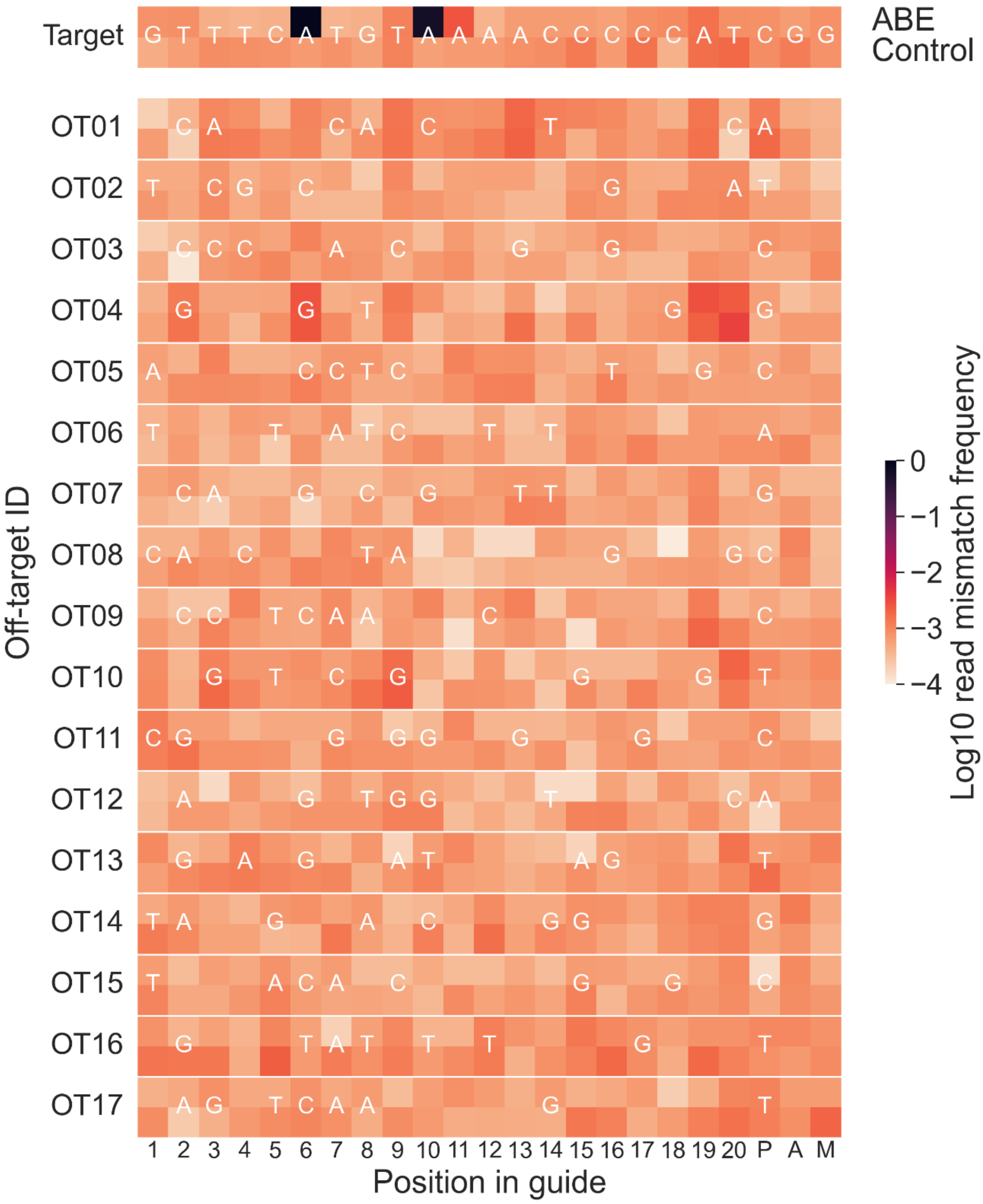

Supplementary Figure 8: Off-target analysis in *E. coli* TN03. Positions in the reference genome with up to 7 mismatches to the target sequence, up to 2 of which in the 10 PAM-proximal nucleotides, were analyzed for off-targets. Each row in the heatmap is a potential off-target region, and the positions with a mismatch to the target are overlaid with the mismatching nucleotide. The values correspond to the maximum Log10 of the read mismatch frequency (as defined in the Methods section) across the two controls and the two adenine base-edited samples (ABE). For comparison, the top row shows the same values but for the intended target. The off-target sites corresponding to individual off-target IDs are listed in **Supplementary Data 2**.

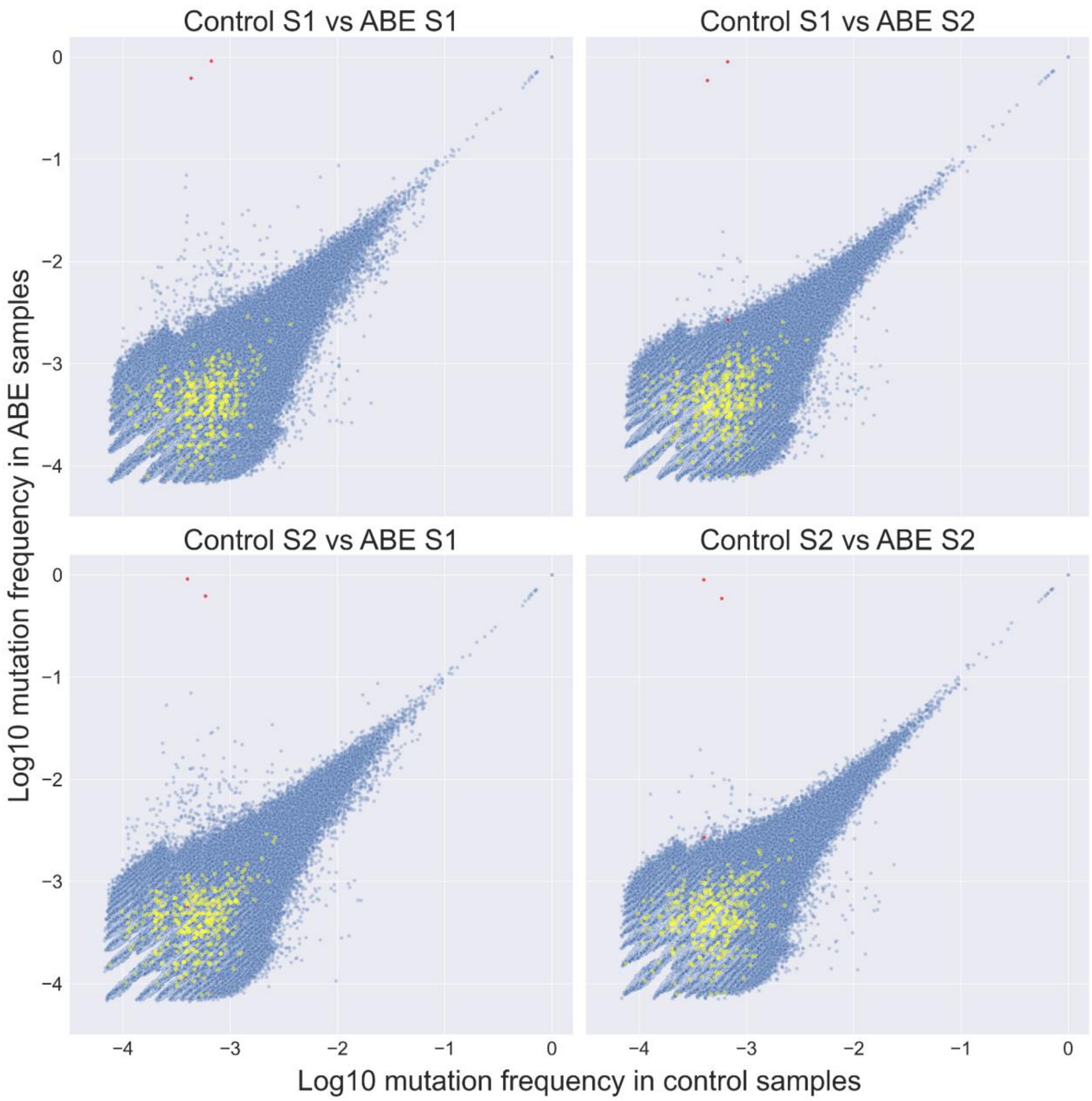

Supplementary Figure 9: Correlation of read mismatch frequency between adenine base-edited (ABE) and non-transduced control samples in TN03-*csgA*. Analysis was performed for two independent experiments (S1 and S2) for ABE-treated or control samples. On-target base editing as well as bystander mutations in the editing window are highlighted in red, while the potential off-target sites from Figure S17 are highlighted in yellow. Unlike Figure S17, all positions in the genome are included. While some positions show an increased mutation rate in ABE-treated samples, a similar effect is observed for other positions in control samples, suggesting that this is due to spurious mutations rather than a consequence of the ABE treatment.

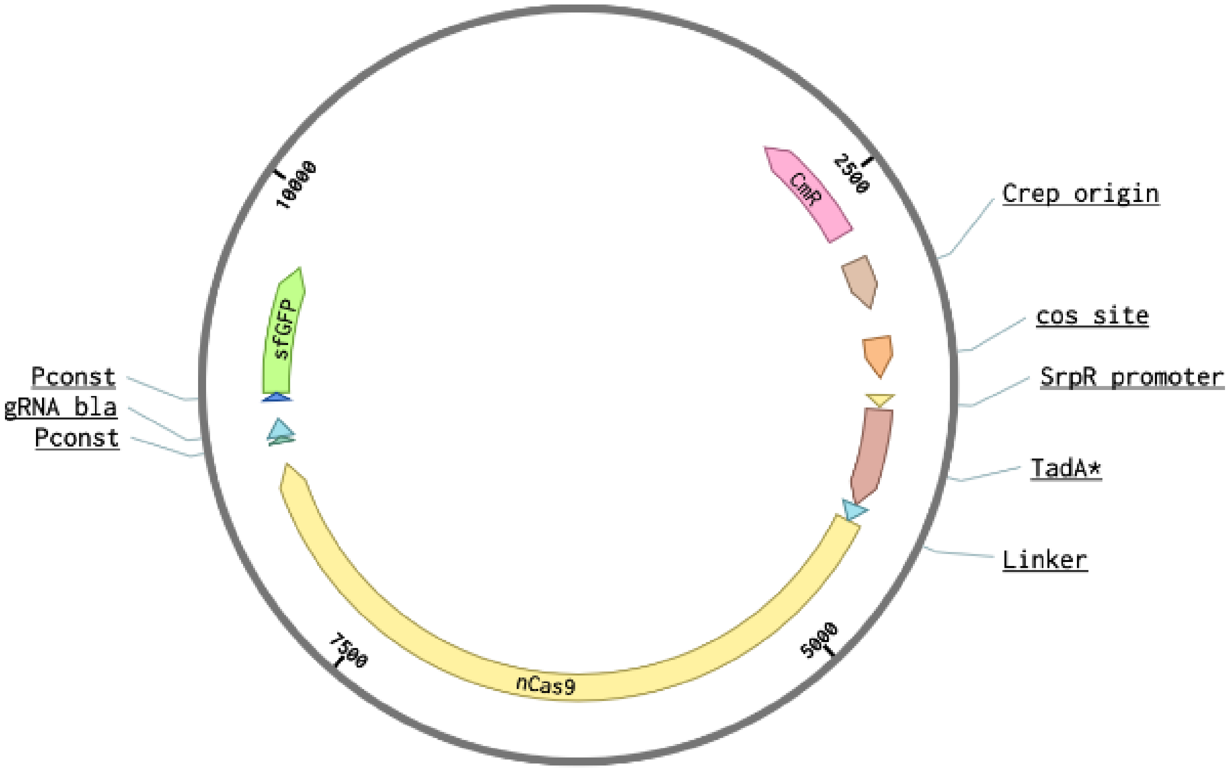

Supplementary Figure 10: Plasmid map of the non-replicative cosmid encoding the adenine base editor and a guide RNA targeting the active site of β-lactamase (*bla*). Packaging into λ phage particles is enabled by the cohesive end site (*cos)* of the λ genome. The adenine base editor is a fusion gene of *TadA** and *nCas9* under the SrpR promoter[1]. The guide RNA targeting the *bla* gene is under a constitutive promoter. The plasmid carries a constitutively-expressed chloramphenicol resistance gene (CmR) and the non-replicative primase origin of replication (Crep). In addition, the plasmid carries the *sfGFP* gene under a constitutive promoter to investigate cosmid delivery efficiencies. The total plasmid size is 10,747 bp.

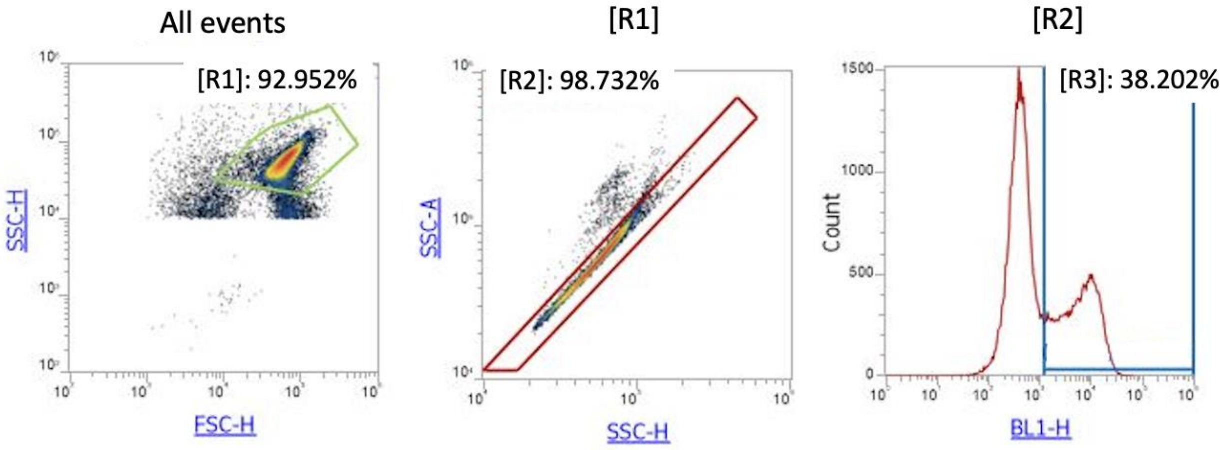

Supplementary Figure 11: Gating strategy used to measure *in vitro* delivery efficiencies. R1 gate on dot density plot of all events (bacterial population), SSC-H/FSC-H. R2 gate on dot density plot of [R1], SSC-A/SSC-H (single cells), R3 gate on histogram plot of [R2] (Count/BL1-H for GFP or Count/YL2-H for mCherry). GFP+ or mCherry+ population gated in R3 and its percentage are plotted in all figures reporting delivery efficiencies measured by flow cytometry.

Table S1: Culture volume required to obtain an intermediate dose of 10^{10} particles. This calculation is based on an eight liter batch fermentation and the standard purification process described in the methods section.

Batch volume [ml]	Final volume after purification [µl]	Titer [tu $µl^{-1}$]	Volume needed per dose of 10^{10} tu [µl]	Number of doses per 8 liter batch	Culture volume per dose [ml]
8000	5500	3 x 10e9	3.3	1650	4.8

Table S2: Extrapolation for the number of particles [tu] and culture volume needed for a human dose. The calculation is based on an intermediate dose of 10^{10} particles for an average BALB/c mouse weight of 18 gram. The GI tract volume conversion factor of 0.21 was obtained from data by McConnell *et al*[2]. The human dose was calculated for a human with a body weight of 70 kg. *The culture volume needed for 1 dose was calculated in Table S1.

Total dose for 1 mouse [tu]	Mouse dose [tu/kg bodyweight]	Mouse to human GI tract volume factor	Human equivalent dose [tu/kg bodyweight]	Total dose for 1 human [tu]	Culture volume needed for 1 mouse dose [ml]*	Culture volume needed for 1 human dose [ml]
1 x 10e10	5.6 x 10e11	0.21	1.2 x 10e11	8.2 x 10e12	4.8	3922

Table S3: Strains used in this study.

Strain	Genotype	Supplier
CY2120	*BW25113 Δ9 λcI857 PaPa Sam7 Δcos lamB-*	John Cronan
CY-1A2	*BW25113 Δ9 λcI857 Sam7 Δcos gpJ-1A2 Δstf lacZ::SrpR lamB-*	This study
CY-A8	*BW25113 Δ9 λcI857 Sam7 Δcos gpJ-A8 Δstf lacZ::SrpR lamB- ΔOmpC*	This study
CY-Ur-λ	*BW25113 Δ9 λcI857 orf401::orf314 Sam7 Δcos lamB-*	This study
DH10B	*F– mcrA Δ(mrr-hsdRMS-mcrBC) φ80lacZΔM15 ΔlacX74 recA1 endA1 araD139 Δ (ara-leu)7697 galU galK λ– rpsL(StrR) nupG*	Thermo Scientific
E. coli CFT073	*O6:K2:H1*	Pasteur Institute
Klebsiella pneumoniae (IHMA_1445327)	*ST258*	IHMA
MG1655	*K-12 F- λ- ilvG- rfb-50 rph-1*	Pasteur Institute
MG1655-*ΔLamB*	*K-12 F- λ- ilvG- rfb-50 rph-1 ΔLamB*	This study
MG1655-*ΔLamB-ΔompC*	*K-12 F- λ- ilvG- rfb-50 rph-1 ΔLamB ΔompC*	This study

MG1655-*mCherry*	*K-12 F- λ- ilvG- rfb-50 rph-1 mCherry*	Pasteur Institute
MG1655-*bla*	*K-12 F- λ- ilvG- rfb-50 rph-1 rpsLK42R wbbL::bla-rfp*	This study
s14269	*K-12 F- λ- ilvG- rfb-50 rph-1 rpsLK42R ompC-EDL933*	This study
s14269-*bla*	*K-12 F- λ- ilvG- rfb-50 rph-1 rpsLK42R wbbL::bla-rfp ompC-EDL933*	This study
s21052	*K-12 F- λ- ilvG- rfb-50 rph-1 rpsLK42R wbbL::bla-rfp ompC-EDL933 rpslK42R*	This study
s21476	TN03 (*O25b:H4) rpslK42R*	This study
TN03	*O25b:H4*	Erick Denamur
TN03-*csgA StrepTag*	*O25b:H4 csgA StrepTag*	This study
E. coli UTI89	*O18:K1:H7*	Erick Denamur
Klebsiella oxytoca CIP107218		Institut Pasteur Collection (CIP); s030
Salmonella enterica subsp. enterica CIP105150		Institut Pasteur Collection (CIP); s038
Enterobacter cloacae LMR_3639		Erick Denamur
Citrobacter freundii LMR_3656		Erick Denamur
Escherichia albertii B156 (s1051)		Erick Denamur
Escherichia fergusonii CIP 104947T (s1014)		Erick Denamur
E.coli O157-Δstx (s17465)		This study
Bacteroides thetaiotaomicron VPI 5482 (s20823; ATCC 29148)		ATCC
Cutibacterium acnes (ATCC 11828)		ATCC
Propionibacterium freudenreichii s77		INRA
Lacticaseibacillus rhamnosus (s11721)		This study
Vibrio cholerae O:1 CIP105882		Institut Pasteur Collection (CIP); s033
Staphylococcus epidermidis SK135 (s12005)		BEI

Table S4: List of plasmids used in this study.

Name	Plasmid	Class	Resistance	Source	Notes
p2325	p15A-cos-pSrpR-RBS-ABE8e gRNA1(mCherry)	Cosmid	CmR	This study	Encodes ABE targeting mCherry in a constitutive origin of replication
p2326	p15A-cos-pSrpR-RBS-CBE gRNA2(mCherry)	Cosmid	CmR	This study	Encodes CBE targeting mCherry in a constitutive origin of replication
p1396	p15A-cos-pSrpR-RBS-ABE8e gRNA3(*bla*)	Cosmid	CmR	This study	Encodes ABE targeting bla in a constitutive origin of replication
p2327	p15A-cos-pSrpR-RBS-CBE gRNA4(*bla*)	Cosmid	CmR	This study	Encodes CBE targeting bla in a constitutive origin of replication
p2771	p15A-cos-pSrpR-RBS-ABE8e gRNA6(*SapI*)	Cosmid	CmR	This study	Encodes ABE and a non-targeting *SapI* site in a constitutive origin of replication
p2770	p15A-cos-pSrpR-RBS-CBE gRNA6(*SapI*)	Cosmid	CmR	This study	Encodes CBE and a non-targeting *SapI* site in a constitutive origin of replication
p513	p15A-cos-sfGFP	Cosmid	CmR	This study	Encodes sfGFP in a constitutive origin of replication
p2328	pCrep-cos-pSrpR-RBS-ABE8e gRNA3(*bla*)	Cosmid	CmR	This study	Encodes ABE targeting bla in a conditional origin of replication
p2515	pCrep-cos-pSrpR-RBS-ABE8e gRNA5(*csgA*)	Cosmid	CmR	This study	Encodes ABE targeting csgA in a conditional origin of replication
p2798	pCrep-cos-pSrpR-RBS-CBE gRNA7(*clbH*)	Cosmid	CmR	This study	Encodes CBE targeting clbH in a conditional origin of replication
p2800	pCrep-cos-pSrpR-RBS-CBE gRNA8(*clbJ*)	Cosmid	CmR	This study	Encodes CBE targeting clbJ in a conditional origin of replication
p2802	pCrep-cos-pSrpR-RBS-CBE gRNA9(*cnf1*)	Cosmid	CmR	This study	Encodes CBE targeting cnf1 in a conditional origin of replication
p2777	pCrep-cos-pSrpR-RBS-ABE8e gRNA10(*fimH*)	Cosmid	CmR	This study	Encodes CBE targeting fimH in a conditional origin of replication
p2779	pCrep-cos-pSrpR-RBS-ABE8e gRNA11(*fimK*)	Cosmid	CmR	This study	Encodes CBE targeting fimK in a conditional origin of replication

p2781	pCrep-cos-pSrpR-RBS-ABE8e gRNA12(*aph(3')-Ia*)	Cosmid	CmR	This study	Encodes CBE targeting aph(3')-Ia in a conditional origin of replication
p1324	pCrep-cos-sfGFP	Cosmid	CmR	This study	Encodes sfGFP in a conditional origin of replication
p2074	pCrep-cos-mCherry	Cosmid	CmR	This study	Encodes mCherry in a conditional origin of replication
p2075	pCrep-cos-Venus	Cosmid	CmR	This study	Encodes venus in a constitutive origin of replication
p938	pSC101-pPhlF-RBS-stf P2	Stf	KanR	This study	Encodes an inducible λ-P2 STF chimera
p2292	pSC101-pPhlF-RBS-stf K1F	Stf	KanR	This study	Encodes an inducible λ-K1F STF chimera
p2058	pSC101-pPhlF-RBS-stf K5	Stf	KanR	This study	Encodes an inducible λ-K5 STF chimera
p1806	pSC101-pPhlF-RBS-stf KL106	Stf	KanR	This study	Encodes an inducible λ-KL106 STF chimera
p1471	pEco-OmpC G1	Receptor	KanR	This study	Encodes OmpC from MG1655
p1472	pEco-OmpC G17	Receptor	KanR	This study	Encodes OmpC from EDL933
p2826	CsgA-StrepTag	Genomic engineering		This study	Encodes a CRISPR-Cas9 system and the StrepTag sequence to genetically fuse the StrepTag to the C-terminal site of the csgA gene
p2076	pIncW-RARE7-RBS-primase	Crep system	TpR	This study	Encodes a constitutive primase gene
p2564	pSC101-J23110-RBS-primase	Crep system	KanR	This study	Encodes a constitutive primase gene
p1321	pSC101-pPhlF-RBS-primase	Crep system	KanR	This study	Encodes an inducible primase gene

Table S5: List of gRNAs used in this study. Edited positions highlight the target mutation site as well as bystander mutations (in brackets) obtained by sequencing.

Name	Editor	gRNA	Position	Target
gRNA1	ABE	CGTACATAAATTGCGGGCTC	(4A), 6A, (8A)	mCherry (M71T, Y72H)
gRNA2	CBE	CACTCAGGACTCCTCCCTGC	5C	mCherry (Q114*)
gRNA3	ABE	ACTTTTAAAGTTCTGCTATG	(1A), 7A, (8A)	β-lactamase (K71E or K71R)
gRNA4	CBE	GATCAGTTGGGAGCCCGTGT	4C	β-lactamase (Q37*)
gRNA5	ABE	GTTTCATGTAAAACCCCCAT	6A, (10A)	CsgA (M1T)
gRNA6	ABE/CBE	TGAAGAGCTTTCGCTCTTCA	-	-
gRNA7	CBE	ATTTTCAGATTAAAATTAGA	6C	ClbH (Q418*)
gRNA8	CBE	TTCAGCAGGTGATAAACACC	3C, 6C	ClbJ (Q140*, Q141*)
gRNA9	CBE	AACTTTCAGAAATTAAGTCG	(3C), 7C	Cnf1 (Q731*)
gRNA10	CBE	GCGCGCAAAGCCAGCAGCTG	(2C), (4C), 6C	FimH(Q212*)
gRNA11	CBE	GTGGCAAACGCTGCTCTATC	5C	FimK (Q91*)
gRNA12	CBE	GCCGCGATTAAATTCCAACA	(2C), (3C), 5C	Aph(3')-Ia (R14*)

Table S6: Selection of oligonucleotides used for plasmid sequencing, genome amplification, and ddPCR. Probes P1 and P2 contained a different fluorophore (FAM or HEX), as well as carefully positioned Locked Nucleic Acid bases (LNA; symbolized by the base A, T, C, or G preceded by a "+" sign in the sequences above).

Name	Oligonucleotide sequence	Gene
F1	ATGGTTTCCAAGGGCGAGG	mCherry
R1	TTATTTGTACAGCTCATCCATGCC	mCherry
F2	ATGAGTATTCAACATTTCCGTGTCGC	*Bla*
R2	TTACCAATGCTTAATCAGTGATGC	*Bla*
F3	GGATCTCAACAGCGGTAAG	*Bla* (ddPCR, primer)
R3	GGCATCAACACGGGATAATA	*Bla* (ddPCR, primer)
F4	GACACAACGTTAATTTCCATTCGAC	*CsgA*
R4	AGTACTGATGAGCGGTCGC	*CsgA*
F5	GCGTGACACAACGTTAATTTCCATTC	*CsgA* (ddPCR, primer)
R5	AGAGCGCTACCGGAGAATACG	*CsgA* (ddPCR, primer)
F6	GAGCTGAATATTTACCAGTACGGTGGC	*CsgA-StrepTag*
R6	GAAGTGACTGTCCATCAGAAACAGTAACAAC	*CsgA-StrepTag*
F7	GCGCGGGTGATTTATGATTCACGG	*ClbH*
R7	TCGACGCGACTGGCTGAGTACGC	*ClbH*
F8	CGGATCCGGCGCAGGCAATC	*ClbJ*
R8	CAGCAGAGGCGCGTCTTGCAAAC	*ClbJ*
F9	CAGGGCCCACAGTCAAGCTTAG	*Cnf1*
R9	CCACGAGCAGAATTTGACACACG	*Cnf1*
F10	GCGCGGGTGATTTATGATTCACGG	*FimH*
R10	TTCAGCATCAGCTGGCTCAACCC	*FimH*
F11	AGGTTACCGCCGGCAACGTGC	*FimK*
R11	AGCCAGCCGCTGGGTGTAGAGC	*FimK*
F12	GCAACAGTGCCCTCTGATGTTACATTG	*Aph(3')-Ia*
R12	GGAACACTGCCAGCGCATCAAC	*Aph(3')-Ia*

P1	FAM-CT+TT+T+A+AA+GTT+C+T+GC	*Bla* (ddPCR, probe 1)
P2	HEX-CT+TT+T+G+AAGTT+CT+GC	*Bla* (ddPCR, probe 2)
P3	FAM-AC+A+T+GAA+A+CT+T+TTAAAA+G+T+A+GC	*CsgA (ddPCR, probe 1)*
P4	HEX-AC+A+C+GAA+A+CT+TT+TAAAA+GT+A+GC	*CsgA (ddPCR, probe 2)*

Supplementary References

1. Stanton, B. C. *et al.* Genomic Mining of Prokaryotic Repressors for Orthogonal Logic Gates. *Nat. Chem. Biol.* **10**, 99–105 (2014).
2. McConnell, E. L., Basit, A. W. & Murdan, S. Measurements of rat and mouse gastrointestinal pH, fluid and lymphoid tissue, and implications for in-vivo experiments. *J. Pharm. Pharmacol.* **60**, 63–70 (2008).

List of gene sequences used in this study

Base Editor target genes

mCherry

ATGGTTTCCAAGGGCGAGGAGGATAACATGGCTATCATTAAAGAGTTCATGCGCTTCAAAGTTCACATGGAGGGTTCTGTTAA
CGGTCACGAGTTCGAGATCGAAGGCGAAGGCGAGGGCCGTCCGTATGAAGGCACCCAGACCGCCAAACTGAAAGTGACTAAAG
GCGGCCCGCTGCCTTTTGCGTGGGACATCCTGAGCCCGCAATTTATGTACGGTTCTAAAGCTTATGTTAAACACCCAGCGGAT
ATCCCGGACTATCTGAAGCTGTCTTTTCCGGAAGGTTTCAAGTGGGAACGCGTAATGAATTTTGAAGATGGTGGTGTCGTGAC
CGTCACTCAGGACTCCTCCCTGCAGGATGGCGAGTTCATCTATAAAGTTAAACTGCGTGGTACTAATTTTCCATCTGATGGCC
CGGTGATGCAGAAGAAGACGATGGGTTGGGAGGCGTCTAGCGAACGCATGTATCCGGAAGATGGTGCGCTGAAAGGCGAAATT
AAACAGCGCCTGAAACTGAAAGATGGCGGCCATTATGACGCTGAAGTGAAAACCACGTACAAAGCCAAGAAACCTGTGCAGCT
GCCTGGCGCGTACAATGTGAATATTAAACTGGACATCACCTCTCATAATGAAGATTATACGATCGTAGAGCAATATGAGCGCG
CGGAGGGTCGTCATTCTACCGGTGGCATGGATGAGCTGTACAAATAA

Beta-lactamase (*bla*)

ATGAGTATTCAACATTTCCGTGTCGCCCTTATTCCCTTTTTTGCGGCATTTTGCCTTCCTGTTTTTGCTCACCCAGAAACGCT
GGTGAAAGTAAAAGACGCTGAGGATCAGTTGGGAGCCCGTGTGGGTTACATCGAGCTGGATCTCAACAGCGGTAAGATCCTTG
AGAGTTTTCGCCCCGAAGAACGTTTTCCAATGATGAGCACTTTTAAAGTTCTGCTATGTGGCGCGGTATTATCCCGTGTTGAT
GCCGGACAAGAGCAACTTGGTCGCCGTATACACTATTCTCAGAATGACTTGGTTGAGTACTCACCAGTTACCGAAAAGCATCT
TACGGATGGCATGACAGTAAGAGAATTATGCAGTGCTGCCATAACCATGAGTGATAACACGGCAGCCAACTTACTTCTGACAA
CGATCGGAGGGCCGAAGGAGCTAACCGCTTTTTTGCACAACATGGGGGATCATGTAACTCGCCTTGATCGTTGGGAGCCGGAG
CTGAATGAAGCCATACCAAACGACGAGCGTGACACCACGATGCCCGCAGCAATGGCAACAACGTTGCGCAAACTATTAACTGG
CGAACTACTTACTCTAGCTTCTCGTCAACAATTAATAGACTGGATGGAGGCGGATAAAGTTGCAGGCCCACTTCTGCGTTCGG
CCCTTCCGGCTGGCTGGTTTATTGCTGATAAATCTGGAGCAGGCGAGCGTGGATCTCGCGGTATCATTGCAGCACTGGGGCCA
GATGGTAAGCCCTCCCGTATCGTAGTTATCTACACGACGGGGAGTCAGGCAACTATGGATGAACGAAATAGACAGATCGCTGA
GATAGGTGCATCACTGATTAAGCATTGGTAA

CsgA

*CCGATGGGGGTTTTAC*ATGAAACTTTTAAAAGTAGCAGCAATTGCAGCAATCGTATTCTCCGGTAGCGCTCTGGCAGGTGTTG
TTCCTCAGTACGGCGGCGGTGGCGGCAACCACGGTGGTGGCGGTAATAACAGCGGCCCGAATTCAGAGCTGAATATTTACCAG
TACGGTGGCGGTAACTCTGCTCTTGCTCTGCAAGCTGACGCCCGCAACTCTGATCTGACCATTACCCAGCACGGCGGCGGTAA
TGGTGCAGATGTTGGCCAAGGTTCTGATGACAGCTCAATCGATCTGACTCAGCGTGGTTTCGGCAACAGCGCTACTCTTGATC
AGTGGAATGGTAAAGATTCTACTATGACTGTTAAACAGTTCGGTGGCGGTAACGGTGCTGCTGTTGACCAGACTGCATCTAAC
TCCAGCGTTAACGTCACTCAGGTTGGCTTTGGTAACAACGCGACCGCTCATCAGTACTAA

Italics: Sequence upstream start codon where gRNA binds

ClbH

ATGGAACAGCAAGGGATTATGAGACAGTTGCCTACCGACGACCAAACGATAGTCGACTATTTGTATCGTATCGCCGGAGAATA
TGGGGAAAAAGCCGCTGTATTGATGGGGGACGCGGCGCTGAGCTATCACGATCTTAATGCACGCTCTAACCAACTGGCGCATT
ATCTGCGTGGGCTGGGGATCGGCGAGGATCGTGTGGTAGCTATCCGCCTGCCGCGCGGCATGGCAATGCTGATTGCCATTTTC
GCTATTGTAAAAGCTGGTGGTGCCTATTTACCGCTGGCGTACAATGCACCGCGCAGCCGCATTGAAAATATACTGAGCAACAG
CGGCGCTGTTTGTCTGATCGGTACTGACGATGGTGATCGCTGGCCGATTCCTCGCGTCGAAATCGACAGCGCGGCGGTCAGTG

CCATGCCAACGACGGATCTGCGTTACCGACCACATGCGCGGCAACTGGCGTATATTATTTACACCTCCGGTTCCACCGGTGTC
CCTAAAGGGGTGGCGACGGAACATGCAGCGCTGCTAAACCGAATTGTCTGGATGCAGAACGCTTATCCTATCAGTTCTCAGGA
CGTGCTGTTTCAGAAGACGGTGTACACCTTTGACGTCTCTGTCTGGGAGATGTTCTGGTGGGCGATGTACGGCGCATCTGTAG
TGCTGTTACCGTCCGGACTGGAGAGCGATCCGCGAACCTTGGCTCGACTGATTCAGCGTCACCGCGTGTCGGTTGTGCACTTT
GTCCCTTCGATGCTGAACCTGTTTGTCGAGTATCTGGAGATGAAACAGGATCCTCGTTTGACCGCCTCATTGCGATTAGTGTT
TTCCAGCGGTGAGAAACTCACGGTCCACAGTGTGGCTCGCTTTTATCAATCGGTGGCGCAGGGTGATCTTATAAATCTCTATG
GCCCGACCGAAGCAGCAATCGATGTCAGTCATCACCGCTGCCTGCGCGGGTACGACTACGACGATATCCCTATCGGTCAAGCG
ATTGACGGTTGCCGACTCTATGTGCTGGATGACCATGGTAATCCGGTAGCAGACGGCGAAGAGGGCGAACTGTATCTCGCTGG
TATCGGGCTGGCACGTGGCTATCTCAACAACGTGGCGTTAACTGATCGCTGTTTTACTATACATCCAACTTTGCGCCATTTAG
GGAAACCGGAGCGGCTGTATAAAACTGGAGATCTGGTGTGGCGCGACGGGGAAAGCCAACAAATTCATTACATTGGCCGTAAT
GATTTTCAGATTAAAATTAGAGGGTTGCGCGTTGAATTGGGAGAAATCGAAGCCCATGCGATGCGTTTCCCGGGGGTACAGCA
GGCAGTCGTGGTGGCGGATCAGGATGATCCCGACAATCAGTTGATTTACGCTTTTGTCGTCAGTAGCGTGCCGCTCAATTTGG
CGGCCTTAATGGACGCACTGTCCAAAAACTTGCCTGCCTACATGCTGCCGAACCGTTTGTTGGCAATGTCAGAGTTACCACTC
TCCGACAATGGCAAGTGCTGTCGTAAAACGTTGCTCGACTTGGCGCGGGCGTACTCAGCCAGTCGCGTCGATTTACGTGAAAC
TCCCGCCGTGCGCTACCTACCGTTGTCATCGGCTCAATCGTCGATGTGGTTTATGCAACAATTGGCGCCGCATACTGCACTAT
ACAATAACCCCACCGCCTTGCTGCTTGAAGGAGAACTGGATCGCACGCGGATGGACGGCGCGATTCGTCAATTGATGAGTCGG
CATACTCTGTTACGTGCTATGGCGGAAACCCACAATGGACAACCAGTATTGGCGGTGCCTCAGTGCGTATCGTCGCAGGCGTT
GCTGACCATAGTGCCACTCCCCTCGGTGAGTGATGATAACGCGCTACAGGCGATGATCAACCAGCGTGCGGCACACCCCATGC
CCTTAACGTCAGGCACACCGCTGTGCCGGTTTGAACTGTTGACGCTCGATGACGATCGTAGCGTATTGTTGATTCATCTGCAC
CACATCATCAGTGATGGCTGGTCGAAAGGCGTTTTGTTACGCGAATTGCAGGCCGCATATAACGGTGAATCGCTGACGCCAGA
ACCGTTGTTGGAGTATGCCGACTACATGGAGTACCAAGAAGAGTGGCGTCAGAGTGACGCCTATCAGGACGCGATGCGTTACT
GGCAAAATACCCTGGCGGGGACGTTGCCGATTCTTGATATCCCCACCGATCAACCGCGTCAGAAGGTGGCACGTTACCAAGGC
GCGTTTGTTGCCTTCGCATTGTCTGCCAACACATGCGAGCGAGTGTTGGCGGCGGCGCGTGCGCAGCGCGTGTCGTTGTATAA
CTATCTGCTGACCGCCTTCGTCCTCCTGCTGCATCGTAATGCGCGTCAACAGGAATACATCGTCGGTATGCCGATTGCTGCGC
GGCTGACGAAAGAACAGGAGCATATGATCGCGCCGCTGGTCAACGTACTACCGCTGCGCTTACCTCTTGACGAAGCGGCATCG
TTTTCCGAGTTAGTACAGACGATCAGAGGTATTCTGTTTGCCGCTTTCAGGCACCAGCGTCTCGAATTTACCGACATAGTGCG
CGCAGTGAATGTGGATCGCAGCGCCGGACATTTCCCAATATATCAGTGCATGTTCCAACTCGACAATATGCCTCTGGCGAGCC
CGACACTAAACGGTGTCAACGTTACACCATTATTGTTGGATACCAGTGCATCTCAGGTGGATATTTCGCTAAGTATGCAGCAT
ATCGATGGGCGCATCACCGGCACTTTCGAATACGACGCTGGGTTATACAGTGCGGATCGTATTCAGCACCTGGTGGCGCAGTG
GAGAGAACTGCTAGATGAGGCCAGCAGCCAACCCACGCAGTTAGTCCGCGATTTGATTCGTTTCACCCCTCGAGAGCACGCTT
GGCTGGCGCGGCACAACGCCACTGAAGTTGCTCTGCCGCCCGTAGATAACCTGCTGGCGCTAGTGTTACCGCACTGCCAGCAG
CGCCCTACACAGGTGGCTTTGCGCCACGCTGACGACGCCATGACCTATGGCGAATTGCAACAGGCCACGATGCAGATGTGTAC
TTGGCTGCGTGCGCAAGGCGTAAAACGCGGAGAATCTGTCGCACTGCAACTGCCTTTTTGTTTCGAATTGATTATCGCACAAT
TGGCGATCCTCTCTTTGGGGGCGAGCTATGTGCCGCTGGATGGTAACGCGCCAGCGGCCCGCAATGCCCTGATCTTGGCGCAG
GCAACGCCGTGCATGCTGCTGGTGGCGCAACCGCTGGAATCCCCTCATGGGCTGACGATACCTTGGGTGCTAGTGCCTGACTG
GCGTAGCCTGCTGACAGAAATACCGAACCTGCCAGTTAGTGTTGCGCCAGATGCTTTAGATTGCGATGCGGTGGTGATCTTTA
CCTCCGGTACTACCGGGCAGCCCAAAGGCGTCCGACTCAGCCAACGTAATCTGGTCAACTTAACCGCATCCTTTATATCCAGC
TATCAAGTGACGCATCAGGATGTTTTGCTACCCATTACCTCAGTCGCGTCTGCTAGCTTTGTCGGGGAGGTACTGCCGCTGCT
CGCCGCTGGCGGTACCTTAGTATTGGCGCAAAAAGCGCAGAGTTTGGATAGTGATGCGCTCATTGCGCTGCTGGCATCTCAGC
GGGTGACTATCCTGAGCACCACCCCATCGCTTTCTGCCAGCCTCTCTGTGCTGGCTCAGTCGATGGGATCGCTGCGCTTGTTT
CTGTGCGGTGGTGAAGCGTTAGAGTATGAGCAGATAGCGCCGCTTCTGCCGCACATGGCAGTGGTTAACGGTTATGGCCTGAC
CGAAAGCGGTATCTGCTCGACCTACTTTCCTGTCGCAAAGCGTAGGGAGCAAGAAACGGGAGCATTGCCCATTGGCCGCCCGA
TTCAGAACACCCAAGCTTATGTGGTGGATGCATATAATCGTTTGGTACCGCCAGGTGCCTGTGGAGAACTCTGTTTCTCTGGT
CTGGGCATTTCGCCCGGCTACCTTGATGCACGACAGGATCCCGAGCGCTTTGTCGAGTTGCCGGAATACCCTGGCGTTCGGGT
GCTGAAAACTGGCGATCGTGCTCGTTGGGCAACCGACGGGATGTTGTTCTATCTCGGCAGACAAGATCGCCAAGTGCAGATCC
GTGGATACCGAGTTGAACTGGGCGACATCGAAAGCCTACTGAAACAGCATCCGGATATTGCTGATGCGTGGGTCGATGTGCGA
CGCAATGCGGCGGCAACGCCGCTACTAGTGGCCTTCTATTGCAGCGTCAACGGCGTTGCGCTGGATGCTCAGCAATTGCGCGT
ATGGCTCAGCCTGCGGCTTCCGTTGCATATGCTGCCGTTGCTCTACGTGCCGCTGAGCGCCATGCCGCTAGGTGTAAACGGCA
AAATCGATCCCCAGTGCCTGCCGCTGGTCGATCTGCGGCAGTTGGAAGGGCCGGGCGAGTATGTCCCTCCGGCAACCGAACTG
GAACAGCGTCTGGCGGAGATCTGGCAGCAGTTGCTTGGCCTGGAGCGTGTCGGCACCACCACAAATTTCTTCGATCTCGGTGG
ACACTCACTTCTGCTAGTACAGATGCAGCAATACATCGGGCAGCAGTGCGGTCAGCACGTGGCGTTGGTTGACCTGCTGCGCT
TCACTACCATCAAACGCTTGGCGGAATTTCTGCTGGCCCCGGACGCAGCGCAAGGGACCACAGGAGATCAGACACAACTGCGT
GCGGCGAAGCAGCGTTTGGCCTTTGGTCACACGCGCTGGGCAGCCACAACCGACAGTCATCACTGA

ClbJ

ATGACGATACATCATGCCGCATTGGCGCGAATGTTACCGGCGGAAAAAAAGGAAAAGCTATTACGACAGTTGGCGCAATCAGG
CGTGTCGCCCAGCCGCATTCCCATCATCAAAGCGGATCCGGCGCAGGCAATCCCGCTGTCGTTTAATCAGGAGCGGTTGTGGT
TTTTGCAAAAATACGACAGTACCGCCACTAACTACAACCTTTATGTGGTGTATCGTTTGCATGGCGTGGTGGATATGCCGATG
CTCACGGAGGCATTGCGCCATGTGCAAGCACGCCACGCCATCTTGCGTACCCGTATCATAGTACGTAACGATCGGCCTTGCCA

GGTGATAGATGATGCGTCTTCGTTAGTTCTCGATACGGTCACGCTGGCAGCGCAGGCCCCAACTTCAGCGCTAGATGCGGTAA
TTCAGCAGGTGATAAACACCCGGTTCGATCTGGCACGCGGTCCTCTGTGGGGGGTAACTCAGATTATTCAACCCGATCAAGGT
TGTCATTTGGTGTTTTGCGCGCACCATATCATCATCGACGGTATCTCGTTACGGCTATTGTTCGATGAGTTACAGCAGCAGTA
TGCTCGATTGCATGCTGGTAATGAAACGTCGCTGCCTCCGCCGCCGCTGCAATATGCCGATTATGCCTTCTGGCAGCGCGAAT
GGTTCCAGGATACCTTGCTGGCGAACGAACTTGCCTACTGGCGTGCGCGTTTGCAAGACGCGCCTCTGCTGTCGACATTTCCA
TCGCTGCACCCGCGCCCGGCACAACCCTCTACACATGGTTCCCGGTTCAGCATCACCCTGGATGAAACATTAAGCCTCGCGTT
GAAACACGTAGCCCGCACGCAGGAAACAACGCCGTTCGTGCTGATGCTGACTGCCTTCCAACTGGTGCTGATGCGTTACGCTC
AGCAACAGCGATTGGTGATAGGCATGCCAGTATCGGGGCGCATTCGCCCGGAATTGCAGAGTAGCATTGGTTATTATGCCAGC
ACTGCGGTTATCTACACCGATTTTAATGGGGTTGAGGTAGGGCGCGAGGCGCTCCAGCGTGTGAAGGCCAGCGTGAAAGAGAC
GCAGGGGCGCCAGCAACTGCCGTTTGAAAACTTGGTGAATATGCTGGATCTCCCTCGCAGCCTTTCCCATTCACCGTTGTTCC
AGATCCTCTATATCTACCATAACCACGTGACCCCACGCGCTTTTACCTTGGCTGGTGCGTATTGGGAGCAGGTGACGTATCAC
AATCAGACCGTCAAATACGACATGACAGTCGAGGTGTTCCAAAACGACGCCACGTTCGACGTCTCCTTTGAGTACGATTTGGG
GTTATACGATGCTGATGTGGTGAAGCAGATTGCCGAGGCGCTGCGTCAGCACTGCTTATCGTTGACATCATCACTGGAGACCC
CGATAGGGGCGATCCCTCTGCATGCACCGGAGACCGCAACGCCGCGGCGTGATCCGCTCAACGCCACTAACGTCCCGTGGCTC
GGGCCGCAGGATGTGCTGCGTATCATTGAACAGCGCTGTGTGCAGCACCCAAAGCAACTGGCAATACAGCAGCATGACGGCAC
ACTGACCTACGCTGAGCTCTGGGCGCGTGTGCAGTTCATCGCGATGCGTTTTCGAGCGCATGGCATACAACCGGGCGATCGTA
TTGGCGTGCTGTTGCCACGTCACAGGGATGTGATTGCAACCATGTTGGCGACGTGGTTTGTGGGGGCGTGCTACGTGCCGTTC
GATATTCATCAGCCTGCCGCGCGTTTGCAACGCCTTATGCAACGCGCGCGTTTGGTCTGTCTGGTGGTCCGTCAGCCGGGAGA
GTGGGGTGAAATTGTACAACTGTCGTTGCCGGAATTGATGCAGGACATGTCGAATGCGATCCGGTATTCTACACCTTGCGCGC
TGTTGCCGGATATGCAGGCCTACCTGTTGTTTACCTCCGGCAGTACCGGTGAACCTAAAGGCGTGTGCGTTGTTCACCGCGGG
TTGCTGAACTTGTTGTTGGATATGCAGCGTACCTTTGCGGTTGGCTCGCAAGACCGGCTGCTCTCGGTGACGACGCCAACATT
TGATATCTCATTCTTGGAGTATCTGTTGCCGCTGATCTCCGGTGCGAGTCTCTATCTGACAGAGGCGGAACGCGCCGCAGACA
GCTTCCGTATGATTCCGCTGATTGCCGACTATCGACCAACGCTGATGCAGGCGACGCCCTCGTTCTGGCACGGGCTGTTGATG
GCGGGTTGGCGTGGCGACCCGGAACTATGTGTGCTGGCAGGCGGTGAAGCGCTGCCAACGAAAGTGGCGGAAGAACTGTTGCG
CTGTTGTGGTTCATTGTGGAACCTCTACGGTCCCACCGAAACAACCATTTGGTCGCTGAAATCGCAGATAACCCAAGCGGAAA
ACATCACCCTCGGCGCTCCCATTGCCAATACCCGTATATACATTCTGGACAATGAGGGCCATCCAGTGCCGCAAGGCGTTGAC
GGCGAGCTTTACATCGCCGGGGATGGTGTGGCGCAAGGGTATGATGGGCAGCCTGAGTTGAACGCACAGTTCTTCTTGTCAGA
ACCAGGGGTCCCCGGTGGCAGGATGTTCCGCACTGGCGATCTGGTCAGGAGTGATGCGCAGGGGCAGTTGTTTTTTGTTGGGC
GCAAAGATAGCCAGATCAAACTGCGCGGTTATCGTATTGAGTTAGGCGAAATTGAACGGACGTTGGCACGGCATCCGCATGTG
GACGCCGCGGTGGTGGCCTGTATTGAAAGAGCACCGTTACACAAAGCACTGGCAGCATTCATCATCACCAGTGAGCCTCCCTC
GCTATTCGAGCAACTGAAAAACGAGCTTCGGCAGCAACTGCCAGACTATATGGTGCCAACGCTGTGGCAGAGGGTGGCCGACT
TCCCGAACACTGACAACGGTAAAATCGATCGTAAGCGGTTAGCGGAAAATTTCGTTGCCGATAGCTCCCTTGTGTCGCCGCAG
ACGCAAGCGCTGAGCGACACGGAACAGATGTTGCTGGCGCTGTGGATGCGCTATTTGCCGATAAAAAACGTCGATCCTGAGTG
CGATTTCTTTCGCTTGGGGGGACATTCGCTGTTGGCGGTAACGTTGGTAGCAGAGATCAACCGCACTTTTCACTGCGCCTTGA
CCCTGAAGGACATTTTCCACTACTCCACACTACGGGCACTGAGTGCGCGTATTGCACAGCAATCTATCACGGACGCTGCCGCG
TCTCAGGATGACTGGGTGATAGTGCACGACCCTGAGCATCGTCATCAACCGTTCCCATTGACGGATGTTCAGCGCGCCTACTG
GCTTGGACGACAGACGGGTGCTACCTCGATCGCGACCCACATCTACCATGAATTTGACGTAGAACACTTTAATGTTACGCGTT
TTACCCATGCGGTGAATGCGCTGATCGCTCGCCATGAAATGCTACGTGCGCGGGTACTCCCCGACGGTACTCAGCAGATTCTG
GCGCAAGTGCCGGCGTATCAGTTAGAGCAGCGCGATCTGAGTGCTTTGTCCCCTAACGCACGAAACGATGCCTTGATGGCGAT
CCGCGATCGGCTGTCGCATCATGTGCATCCCGCAGATCGTTGGCCGCTGTTTGATTTCAGTTATTCGGCTTGCACGGCGCAAC
ATGGCCGCTTGCATTTCAGTCTCGATCTGCTGATTGCCGATGCTCTGAGTATGCGCACGCTACAGCAGGAGTTGATGATGCTG
TACCGTGAGCCCCATGTGTCACTGCCGTTGCTACCGTTCTCTTTTCGTGACTACGTGCAGGCGCTGTTGGTAGAGCAGGCGAG
TGAAGCCTATGCACGCGATCAGGCCTATTGGCAACGGGCGCTGCCGCAGCTGTATGGCCCACCAACGCTGCCCGTACAGGGCG
ATTTGGCGCAACTGTCTGCGATCAGGTTCGTACGTCGCCGTCATCGGCTGTCAGCCCACAACTGGGGAGTGCTGAGCGCGCTG
GCCCAACGCACACGTATCACCAAGACGGCATTGTTGTTGACAGTCTTTAGCCAAGTGCTGGCACGTTGGAGCCTTAGCCCGAC
GTTTACGCTCAATCTGACGTTGTTCAACCGCCCGCAGGGTTACCCCAACGCAGAGGCAGTTATTGGTGATTTTACCGCTGTCA
GCTTGCTGAATGTTTGTTACGACAGCCAGCACTCTTATGCCCACAACGCTCAGCGTATTCAGGTGCAACTGTGGGAAGATCTC
GAACATCGTCGTTTCAGTGGGATCCGCGCCAGCGAGGCGCTGATCCATAGCGGTCGTTTCCATGCGCCGATGCCGGTGGTATT
CACTAGTATGTTGGATATCGACGGGGAGACGACTGCGCAAGACCCTCGGGACACAACCCGTTTTACTCTGTGTCCGGACGCCA
ATATTACCCAAACACCGCAGGTGTGGCTCGATCACCAGGTGATCGAGTTGGCTGGGGAGTTGCATTTCAACTGGGACGCGGTC
GAGCAACTGTTTGATACCACGCTGCTGGATCAGATGTTTGGTGCTTATTGTCATGCGCTGCAGGCGCTGGTTGCCATGCCGCA
AAGTTGGTGGGGGGTAAATAGTTCTCTGGCGCTGCCCACCGTTAGTGCACCGGTCACGCAGGCTCCTGCACCTACGGCCTTGT
TGCACCATGGATTACTGCGTCAGGCAGCACTGACGCCACAGGAAACTGCGCTGATCAGTCCTATCCGTGAATTGACCTATCGC
CAACTGTCGACGGCGGCGGATCATGTGGCCCGCGCCCTGTTAGCGCTGGGCGTGCAGCATGGCGACCGCGTGGCGGTGGTGAT
GGAAAAAGGCTGGCAGCAGATTGCCGCCGTACACGGCATTTTACGACTGGGTGCGGTCTATCTGCCAGTGGATCCGGTGCTAC
CGCCACAGCGTCGCCAGCTTTTGCTGACGGTGGGCGAGGTGCGGGTACAAGTAACGCAGCCGGGTCTCACGCAATTGGAGCCG
TCGCTGCCCGTGCTGATCATCGACGACGGAATGCTGGACACGCCTGCTGCGCCGTTGCCTGAAGTGGCTGGGGATGTCACGGA
TCTGGCCTATATCATTTTCACTTCCGGCTCCACCGGTACCCCGAAAGGAGTGATGATCGACCACCGTGCGGCCATGAACACGC
TGGAAGACATCAACGAACGCTTTGGCCTCAATGCGCAGGATAGGGTGTTCGGGCTGTCATCATTGAGCTTTGACCTGTCGGTT
TACGATGCCTTTGCGCCTTTTATGGTGGGTGCAGCGCTGGTACTGCCGGAAGCAGGACGGGAAAAAGATCCGCGTCATTGGCA
GACAGTTATGGCACACGGTCATGTAAGCGTCTGGAATGCAGTGCCCGCACTGATGCAGATGCTGTGCGAATACCACAGCGGCG
ATCGGATGAGTTATCCGACGTTGCGTCTGGCACTGTTGAGCGGCGACTGGATCCCGCTAACGTTACCGGAGCAGATGCGCGAG
CGGCTCAATGAAACGATGGACATCATCAGTCTGGGTGGAGCGACCGAGTGCGCCATCTGGTCGGTCTACTACCCGATAGGTGA

GGTGGAATCGACGTGGACCAGTATTCCCTACGGTCGGGGCCTGCGCAACCAGCCAGTATACGTGCTAAATGCGCAACTGGAGG
AATGTCCGGTCGGGGTGGAAGGAGAGATTTGCATTGGCGGGATGGGGCTGGCACAAGGCTACCTGAACGACGCAGAGAAAACG
GCGGCGAGCTTTGTCTGGCGCGAAGCGAGTGGTGAGCGAATTTACCGCACTGGGGATCGCGGGCGCTACTTTGCTGACGGGCA
AGTCGCCTTTTTGGGGCGCAACGATACCCAAGTGAAGGTGAATGGTTACCGTATCGAACTGGGGGAAGTCAAAAGCCACCTTG
AACAGCTCGACAGCGTAGGGAGTGCCGCCGTGGTGTGCCACCAGGGACAGCTGTATGCCTTTATCACTGCCGCAGAAAACCTG
CATCCTGACGATACTGACGCCCTGTTAGCGCGTGTTCGTGCTCAGTTAGCCGTGCAGTTGCCGTATTACCTGCTGCCCCAACA
TTTCTTTCTTCTCAAGGTGCTGCCGATGACAGGCAACGGCAAAATTGATCAGGCGGCAATGGTTCAAGAGGTTATCCAACGTA
TGTCGCAATCTACATCACAGAAGTCGAGGGCCCTCGCGCACGCCTCGCCCTATGAACAGCAGGTGGCCGCTCTCTGGTGCGAG
GTACTACAACGAGAACAGATCGGACTGAATGACAACTTTTTTGAAGCGGGGGGCGGCTCAATCCAGATCGTGCTGTTGCATCG
CCGTATTGAGGAGATTTTTAAGGTTACGGTACCTATTGCTGAGCTATTTCGCTTAACCACCGTGAAAAGAATTGCCGGTTATT
TGCAGGCTATGCAGGACAATGCACGGGCGGTGAATCAAACACAGCAGCGTGATGCTTCCCGATCCCGCGCCCAGCAACGACTT
GTACGTCGTCACCAGCGTCAGCGTTAA

Cnf1

ATGGGTAACCAATGGCAACAAAAATATCTTCTTGAGTACAATGAGTTGGTATCAAATTTCCCTTCACCTGAAAGAGTTGTCAG
CGATTACATTAAGAATTGTTTTAAAACTGACTTGCCGTGGTTTAGTCGGATTGATCCTGATAATGCTTATTTCATCTGCTTTT
CTCAAAACCGGAGTAATAGCAGATCTTATACTGGATGGGATCATCTTGGGAAATATAAAACAGAAGTACTGACACTCACTCAA
GCCGCTCTTATTAATATTGGTTATCGTTTTGATGTTTTTGATGATGCAAATTCAAGCACAGGAATTTATAAAACAAAGAGTGC
AGATGTGTTTAACGAAGAAAATGAAGAAAAAATGCTCCCGTCGGAATACCTGCATTTTTTACAAAAGTGTGATTTTGCAGGTG
TTTATGGAAAAACTCTGTCAGATTACTGGTCGAAATACTATGATAAATTTAAGCTTTTACTAAAAAATTATTATATTTCTTCT
GCTTTGTATCTTTATAAAAATGGAGAGCTTGATGAGCGTGAATATAATTTCTCCATGAACGCCTTAAATCGCAGTGATAATAT
ATCACTATTATTCTTTGATATTTATGGATATTACGCATCTGATATTTTTGTAGCCAAAAATAATGATAAGGTAATGCTTTTCA
TTCCTGGTGCAAAAAAACCTTTTTTATTCAAGAAGAATATCGCTGATTTGCGGCTTACCCTTAAAGAACTTATTAAGGATAGT
GACAAACAACAATTACTTTCCCAACATTTTTCATTATATAGTCGTCAAGATGGAGTTTCCTATGCAGGAGTAAATTCTGTTCT
ACATGCAATAGAAAATGATGGTAATTTTAATGAGTCTTACTTTCTGTATTCCAATAAGACACTTAGCAATAAAGATGTTTTTG
ATGCTATAGCTATTTCTGTTAAGAAACGCAGTTTCAGTGATGGTGATATCGTTATAAAATCAAACAGTGAAGCTCAACGAGAC
TATGCTCTGACTATACTCCAGACGATTTTATCAATGACCCCTATATTTGATATCGTAGTCCCGGAGGTATCTGTTCCGCTTGG
ACTGGGGATTATTACTTCCAGTATGGGGATCAGTTTTGATCAACTGATTAATGGTGATACTTATGAAGAACGTCGTTCTGCTA
TACCTGGTTTGGCGACAAATGCAGTATTGCTTGGTCTGTCTTTTGCAATTCCACTCTTGATTAGTAAGGCAGGAATAAACCAG
GAGGTACTTAGCAGCGTTATAAATAATGAGGGCAGGACTCTGAATGAAACAAATATCGATATATTTTTGAAGGAATATGGAAT
TGCTGAAGATAGTATATCCTCAACTAATTTGTTAGACGTTAAGCTTAAAAGTTCCGGGCAGCATGTCAATATTGTAAAGCTTA
GTGATGAAGATAATCAAATTGTCGCTGTAAAAGGGAGTTCTCTGAGCGGCATCTACTATGAAGTGGACATTGAAACAGGATAT
GAGATTTTATCCCGAAGAATTTATCGTACCGAATATAATAATGAAATTCTCTGGACTCGAGGTGGTGGTCTAAAAGGGGGGCA
GCCATTTGATTTTGAAAGTCTCAATATTCCTGTATTTTTTAAAGATGAACCCTATTCTGCAGTGACCGGATCTCCGTTATCAT
TTATTAATGATGACAGCTCACTTTTATATCCTGATACAAACCCAAAATTACCGCAACCAACGTCAGAAATGGATATTGTTAAT
TATGTTAAGGGTTCTGGAAGCTTTGGGGATAGATTTGTAACTTTGATGAGAGGAGCTACTGAGGAAGAAGCATGGAATATTGC
CTCTTATCATACGGCTGGGGGAAGTACAGAAGAATTACACGAAATTTTGTTAGGTCAGGGCCCACAGTCAAGCTTAGGTTTTA
CTGAATATACCTCAAATGTTAACAGTGCAGATGCAGCAAGCAGACGACACTTTCTGGTAGTTATAAAAGTGCACGTAAAATAT
ATCACCAATAATAATGTTTCATATGTTAATCATTGGGCAATTCCTGATGAAGCCCCGGTTGAAGTACTGGCTGTGGTTGACAG
GAGATTTAATTTTCCTGAGCCATCAACGCCTCCTGATATATCAACCATACGTAAATTGTTATCTCTACGATATTTTAAAGAAA
GTATCGAAAGCACCTCCAAATCTAACTTTCAGAAATTAAGTCGCGGTAATATTGATGTGCTTAAAGGACGGGGAAGTATTTCA
TCGACACGTCAGCGTGCAATCTATCCGTATTTTGAAGCCGCTAATGCTGATGAGCAACAACCTCTCTTTTTCTACATCAAAAA
AGATCGCTTTGATAACCATGGCTATGATCAGTATTTCTATGATAATACAGTGGGGCTAAATGGTATTCCAACATTGAACACCT
ATACTGGGGAAATTCCATCAGACTCATCTTCACTCGGCTCAACTTATTGGAAGAAGTATAATCTTACTAATGAAACAAGCATA
ATTCGTGTGTCAAATTCTGCTCGTGGGGCGAATGGTATTAAAATAGCACTTGAGGAAGTCCAGGAGGGTAAACCAGTAATCAT
TACAAGCGGAAATCTAAGTGGTTGTACGACAATTGTTGCCCGAAAAGAAGGATATATTTATAAGGTACATACTGGTACAACAA
AATCTTTGGCTGGATTTACCAGTACTACCGGGGTGAAAAAAGCAGTTGAAGTACTTGAGCTACTTACAAAAGAACCAATACCT
CGCGTGGAGGGAATAATGAGCAATGATTTCTTAGTCGATTATCTGTCGGAAAATTTTGAAGATTCATTAATAACTTACTCATC
ATCTGAAAAAAAACCAGATAGTCAAATCACTATTATTCGTGATAATGTTTCTGTTTTCCCTTACTTCCTTGATAATATACCTG
AACATGGCTTTGGTACATCGGCGACTGTACTGGTGAGAGTGGACGGCAATGTTGTCGTAAGGTCTCTGTCTGAGAGTTATTCT
CTGAATGCAGATGCCTCCGAAATATCGGTATTGAAGGTATTTTCAAAAAAATTTTGA

FimH (*Klebsiella Pneumoniae*)

ATGATGAAAAAAATAATCCCCCTGTTCACCACCCTGCTGCTGCTGGGCTGGTCGATGAACGCCTGGTCCTTTGCCTGCAAAAC
GGCCACCGGGGCGACGATTCCCATCGGCGGCGGGTCAGCCAACGTCTACGTTAACCTGACCCCGGCGGTGAACGTCGGGCAAA

ACCTGGTGGTCGACCTCTCCACGCAGATTTTTTGCCATAACGACTATCCGGAAACGATCACCGACTACGTGACCCTGCAGCGC
GGATCCGCCTATGGCGGTGTGCTGTCGAGTTTTTCAGGCACCGTGAAATATAACGGCACCTCTTACCCGTTCCCGACCACCAC
GGAAACCGCGCGGGTGATTTATGATTCACGGACCGATAAACCCTGGCCGGCCGTCCTGTATCTGACGCCGGTGAGCACTGCCG
GTGGAGTGGCCATCACCGCAGGATCGTTAATCGCGGTGCTGATCCTGCATCAGACCAACAACTACAATAGCGACTCCTTCCAG
TTCATCTGGAACATTTACGCCAACAACGACGTGGTGGTCCCCACCGGCGGCTGCGATGTCTCCGCCCGCGATGTCACCGTCAC
CCTCCCCGACTACCCGGGATCGATGGCCGTGCCGCTCACCGTCCACTGCGCGCAAAGCCAGCAGCTGGGGTATTACCTCTCCG
GCACCACCGCGGACAGCGCCAACGCGATCTTCACCAATACCGCCTCCGCCTCGCCGGCGCAGGGGATAGGCGTTCAGCTGACG
CGCAACGGCAGCGCCGTCCCGGCGAACAGCACGGTCTCGCTGGGCACCGTCGGCACCTCGCCGGTCAACCTCGGCCTGACGGC
CACCTATGCCCGGACCACAGGCCAGGTTACCGCCGGCAACGTGCAGTCGATCATCGGCATCACCTTTGTCTATCAATGA

<u>FimK (*Klebsiella Pneumoniae*)</u>

ATGCTCACCACGCTGCGCCAGGCGGCAAGGCTACTCGACGAGGCCTTGCCGCCGTTACCGATGTTGATTCTGAGCCGCAGCCC
GGCCATCTGGCTGTGGCAAACGCTGCTCTATCAGGTGAGTCATCCGGATCGTCTGCGCAACGTCCATACTGCCCCCGCCGATC
TGTCCTGCGCGGAGCTGGCCCATCGGCTGGAGAATGCGCCGCGGCTTGAGCGGCTTGCCAGCGAAGCCGCCCTGATCCACGGA
AAACGGGTCGTCGGGTTGACCCACGCCGAGCTCAAGGTGATCCTCGCCCTGCTGCAAGGGCAGACGATAGGCGAGCAGGCCCA
ACGTCTCGGATTGAGCCAGAAAACGCTCTACACCCAGCGGCTGGCTGGGGTGAAAAAGCTGGTGGAATGTCATCCGCATCTGG
CCCCCCGCTTTCCGCGCACGCTGCTGCCGCGCTCACCCGCAAACGCACTGACGGCGTTTGAACAGGAATGGGTACAAGCGATT
CACGATCGCCAGGTCTTCCCGGTTTTTCAACCTATCGTCGATAGTCGCTCACAGCTACAGGGGGTGGAGATCCTGATCCGCTG
GCGCCACCGCGGCCAGGTACTTCACCCCCAGACCTTTCTACCGCACTTCCGCGCCGACTACACCTGGCTACTGCTTACGGCCT
TTGTTCTGCAGGAGGCCGTGCAGAATATTAATGAGTATCCAGGCACCTTCTATTTTTCGGTCAACATACCCTCCTCACTCGCC
GACAGTGACAGCCTGCTGCGGATGGTGGAAGCCGCTCGCCAGCAGCTTCGGCAGCCAGAGGGCGTGGCAAGGCTGGTGCTGGA
ATATGCTGAAACTATCGATTTTCGCCATCAGAGCCGCTCCGCCGCCCACGTGGCGCAGCTGCAACGTGCTGGCGTGCGCGTGA
TGCTGGACGACTGCTTTTCTCAGGGGAGCGTTATCTTTCCGGCGCGTCGACTGCACTTCAATGCGTATAAACTGGATATGAGC
ATCGTCAACGACGCCCAGCACGATCCGAAGGCGCTTGCGCTAATAAAAAGCCTGGCCTACTACTGCCAGTTAAGCGACAGTCG
CTGTGTGGCGGAAGGGGTGGATTCACTGGCGAAATTTACGCAGTTAAAATCGCTGGGGATTGATCGCTTCTAGGGCTATCTGT
TTTCACCACCGATGCGGCGTGAACATCTCCCGGATCTGATCCGCCGCTTTTCCCACCAGCGCGATCCGGCGGATCGTTGA

<u>aph(3')-Ia (*Klebsiella Pneumoniae*)</u>

ATGAGCCATATTCAACGGGAAACGTCTTGCTCGAGGCCGCGATTAAATTCCAACATGGATGCTGATTTATATGGGTATAAATG
GGCTCGCGATAATGTCGGGCAATCAGGTGCGACAATCTATCGATTGTATGGGAAGCCCGATGCGCCAGAGTTGTTTCTGAAAC
ATGGCAAAGGTAGCGTTGCCAATGATGTTACAGATGAGATGGTCAGACTAAACTGGCTGACGGAATTTATGCCTCTTCCGACC
ATCAAGCATTTTATCCGTACTCCTGATGATGCATGGTTACTCACCACTGCGATCCCCGGGAAAACAGCATTCCAGGTATTAGA
AGAATATCCTGATTCAGGTGAAAATATTGTTGATGCGCTGGCAGTGTTCCTGCGCCGGTTGCATTCGATTCCTGTTTGTAATT
GTCCTTTTAACAGCGATCGCGTATTTCGTCTCGCTCAGGCGCAATCACGAATGAATAACGGTTTGGTTGATGCGAGTGATTTT
GATGACGAGCGTAATGGCTGGCCTGTTGAACAAGTCTGGAAAGAAATGCATAAGCTTTTGCCATTCTCACCGGATTCAGTCGT
CACTCATGGTGATTTCTCACTTGATAACCTTATTTTTGACGAGGGGAAATTAATAGGTTGTATTGATGTTGGACGAGTCGGAA
TCGCAGACCGATACCAGGATCTTGCCATCCTATGGAACTGCCTCGGTGAGTTTTCTCCTTCATTACAGAAACGGCTTTTTCAA
AAATATGGTATTGATAATCCTGATATGAATAAATTGCAGTTTCATTTGATGCTCGATGAGTTTTTCTAA

DNA sequences on plasmids and genome

Adenine Base Editor: ABE8e (TadA*-Linker-nCas9)

ATGAAGCGTACCGCCGATGGCAGCGAGTTTGAATCTCCGAAGAAAAAGCGTAAGGTCAGCGAAGTTGAGTTCAGCCACGAATA
CTGGATGCGTCACGCTTTAACCCTGGCTAAACGCGCGCGCGACGAGCGCGAAGTACCAGTGGGGGCGGTGCTGGTGTTAAACA
ACCGCGTAATCGGCGAAGGCTGGAACCGTGCAATCGGGTTACATGACCCGACCGCCCATGCCGAGATCATGGCCCTGCGCCAG
GGGGGGCTGGTCATGCAGAATTACCGTCTGATCGACGCGACGTTGTATGTCACATTCGAGCCATGCGTGATGTGTGCGGGGGC
AATGATTCACTCTCGCATTGGTCGCGTCGTGTTTGGCGTTCGTAATAGTAAACGCGGCGCTGCTGGCTCCTTAATGAATGTTC
TGAATTATCCGGGTATGAACCACCGTGTCGAAATTACAGAAGGTATCTTAGCAGATGAATGTGCCGCACTGCTGTGTGACTTC
TACCGCATGCCGCGCCAAGTATTCAACGCCCAAAAAAAAGCTCAGTCCTCAATTAACTCTGGTGGTAGTAGTGGCGGCTCTAG
CGGCTCCGAGACGCCTGGTACGTCGGAATCGGCTACGCCTGAGTCGAGCGGTGGGTCCTCTGGCGGCTCTGACAAGAAATATA
GCATCGGCCTGGCCATCGGCACAAATAGCGTCGGATGGGCGGTGATCACTGATGAATATAAAGTTCCGTCTAAAAAGTTCAAG
GTACTGGGTAATACAGATCGCCATAGTATCAAAAAGAACTTAATCGGTGCGCTTCTGTTCGATTCCGGCGAAACCGCAGAAGC
AACACGTCTGAAACGCACCGCTCGTCGCCGTTACACCCGTCGTAAAAACCGCATCTGCTACCTGCAAGAAATCTTCTCTAACG
AAATGGCTAAAGTAGATGACAGCTTTTTTCACCGTCTGGAAGAATCATTTCTGGTGGAAGAAGATAAAAAGCACGAACGTCAT
CCAATCTTCGGCAACATTGTGGACGAAGTAGCGTATCACGAAAAATACCCGACTATCTATCACCTGCGCAAAAAGCTGGTCGA
TTCGACGGATAAGGCCGATCTGCGTCTGATCTATCTGGCCTTAGCGCATATGATTAAGTTCCGTGGTCATTTCCTGATCGAAG
GCGACCTGAATCCAGACAACAGCGATGTAGACAAACTGTTCATCCAGCTGGTGCAAACCTATAACCAGCTGTTTGAAGAAAAC
CCAATTAATGCTAGCGGTGTTGACGCGAAAGCGATCTTGTCCGCACGCCTGTCCAAATCCCGTCGTCTGGAAAACTTAATTGC
GCAACTGCCGGGTGAGAAGAAAAACGGACTGTTCGGCAATCTGATCGCTCTTAGCTTGGGACTGACCCCGAACTTCAAAAGCA
ACTTCGATCTGGCAGAGGACGCAAAACTTCAACTTAGCAAAGATACGTATGACGATGACTTGGATAACTTACTGGCCCAGATC
GGAGATCAGTACGCTGATCTGTTTCTGGCGGCAAAGAACTTATCAGACGCTATTCTCCTGTCTGATATTCTTCGTGTGAATAC
CGAAATCACCAAAGCACCGCTTTCTGCATCCATGATTAAACGCTATGACGAACATCACCAAGATCTGACTCTTCTGAAAGCGC
TGGTACGGCAACAACTGCCGGAGAAGTACAAGGAGATCTTCTTTGACCAATCCAAAAACGGCTACGCGGGTTATATTGACGGG
GGTGCAAGCCAAGAGGAGTTCTACAAATTCATCAAGCCAATCTTAGAAAAAATGGATGGCACGGAAGAATTACTTGTTAAACT
GAATCGTGAGGATCTGCTTCGTAAACAGCGTACCTTCGACAACGGTAGCATTCCGCACCAGATCCACTTAGGTGAACTGCACG
CTATCCTGCGTCGCCAAGAGGATTTTTACCCGTTCCTGAAAGATAATCGTGAAAAAATCGAAAAAATCCTGACCTTTCGTATC
CCGTATTATGTCGGCCCGCTGGCGCGTGGCAACTCCCGTTTCGCGTGGATGACTCGCAAATCCGAAGAAACTATTACCCCGTG
GAACTTCGAGGAAGTGGTTGACAAAGGCGCAAGCGCCCAATCCTTCATCGAGCGCATGACTAACTTTGATAAAAACCTGCCGA
ACGAAAAGGTACTGCCGAAACACTCCCTTCTGTACGAATACTTCACCGTGTACAACGAGCTGACTAAAGTAAAGTATGTGACT
GAGGGCATGCGTAAACCTGCATTCCTGAGCGGTGAACAGAAAAAAGCAATTGTTGATTTACTGTTTAAAACCAACCGTAAAGT
AACCGTTAAACAGCTGAAAGAGGACTACTTCAAGAAAATCGAATGCTTCGACTCCGTCGAGATTAGTGGAGTTGAAGATCGTT
TTAATGCAAGTTTAGGCACGTATCACGATTTATTAAAGATCATTAAAGACAAAGATTTCTTGGACAACGAAGAAAATGAGGAC
ATCTTAGAGGACATCGTCCTGACCCTGACTCTGTTCGAAGATCGTGAAATGATTGAAGAACGCCTTAAGACGTATGCTCACCT
GTTTGACGATAAAGTAATGAAACAACTGAAACGTCGCCGTTATACTGGCTGGGGCCGTCTGAGCCGTAAACTGATTAACGGTA
TCCGTGACAAACAGTCCGGTAAAACTATTCTGGACTTCCTGAAATCTGACGGCTTCGCAAACCGTAACTTCATGCAACTGATT
CACGACGATTCCCTGACCTTCAAAGAGGACATCCAGAAAGCTCAGGTTTCTGGTCAAGGTGATTCTCTGCACGAGCATATCGC
CAATTTAGCAGGTAGTCCGGCGATCAAAAAAGGTATCCTGCAAACCGTGAAAGTGGTGGATGAGCTTGTGAAAGTTATGGGTC
GTCACAAACCGGAAAACATTGTTATCGAGATGGCTCGTGAAAACCAAACGACCCAGAAGGGACAGAAAAACTCCCGCGAACGC
ATGAAACGTATCGAGGAGGGTATTAAAGAACTTGGCTCTCAGATTCTGAAAGAACACCCTGTTGAAAATACCCAACTGCAAAA
TGAAAAACTGTACCTGTACTACCTGCAAAATGGTCGTGACATGTATGTAGATCAGGAGCTGGACATCAACCGCCTCTCCGATT
ACGACGTTGACCACATTGTTCCGCAGTCTTTTCTGAAAGATGATTCCATTGATAACAAAGTACTCACCCGTAGCGATAAAAAC
CGTGGGAAGAGTGACAACGTTCCATCGGAAGAAGTAGTTAAGAAAATGAAGAACTATTGGCGTCAACTGCTTAACGCGAAACT
GATTACTCAACGTAAATTTGATAACCTGACCAAAGCTGAACGTGGCGGTTTGTCTGAGCTGGATAAGGCGGGTTTTATTAAAC
GTCAACTGGTAGAAACTCGCCAGATTACAAAACATGTTGCTCAGATTCTGGACTCTCGTATGAACACTAAATACGATGAAAAT
GACAAACTGATCCGCGAAGTTAAGGTTATTACCCTGAAATCTAAGCTGGTTTCCGACTTCCGTAAAGATTTCCAATTCTATAA
AGTGCGCGAGATTAACAACTATCACCACGCGCACGACGCATATCTGAATGCAGTTGTTGGCACGGCACTGATCAAAAAATATC
CGAAACTGGAAAGCGAATTTGTGTACGGCGATTATAAAGTTTACGACGTGCGCAAAATGATCGCCAAATCTGAACAGGAAATT
GGCAAAGCAACCGCTAAATACTTTTTCTACTCAAACATTATGAATTTCTTCAAAACCGAAATCACCTTAGCGAATGGCGAAAT
TCGTAAACGCCCTCTGATCGAAACCAACGGCGAAACGGGTGAGATCGTGTGGGACAAAGGTCGTGATTTCGCTACTGTCCGCA
AAGTTCTGTCCATGCCTCAAGTAAACATCGTTAAAAAGACTGAGGTACAGACTGGCGGTTTCAGCAAGGAATCCATTCTGCCG
AAACGCAACTCCGACAAACTGATCGCGCGTAAGAAAGACTGGGATCCGAAGAAATACGGTGGCTTCGATTCTCCAACCGTGGC
ATACAGCGTTCTGGTAGTCGCCAAAGTCGAAAAGGGTAAATCAAAAAAACTGAAATCAGTGAAAGAACTTTTAGGCATCACCA
TTATGGAACGTAGCTCTTTCGAAAAAAACCCGATTGACTTCCTCGAAGCGAAGGGGTACAAGGAAGTAAAGAAAGATCTGATT
ATCAAACTGCCGAAGTATTCCCTGTTCGAACTGGAAAATGGTCGTAAACGTATGTTAGCGTCTGCGGGTGAACTGCAAAAAGG
GAACGAATTGGCCCTTCCGTCCAAGTACGTGAACTTCCTGTATCTGGCCTCGCACTACGAGAAACTGAAAGGTAGTCCGGAAG
ATAATGAGCAGAAACAGCTGTTCGTGGAACAGCACAAACACTATCTGGACGAGATTATTGAACAGATTTCTGAGTTTAGCAAA
CGCGTAATTCTGGCGGACGCGAATCTGGATAAAGTCCTGAGCGCCTACAATAAACACCGTGATAAACCGATCCGTGAACAGGC
AGAAAACATCATTCACCTGTTCACGCTGACTAATCTTGGTGCTCCGGCAGCCTTCAAATACTTCGACACCACGATCGATCGTA

AACGTTACACCTCCACTAAAGAAGTCTTAGATGCAACTCTTATTCACCAGAGCATCACTGGCCTGTATGAAACTCGTATTGAT
CTGAGTCAGTTGGGCGGTGACTAA

Cytosine Base Editor: CBE (evoAPOBEC1-Linker-nCas9-Linker-UGI)

ATGAAACGTACTGCCGACGGTTCAGAGTTCGAGTCGCCCAAGAAGAAACGTAAAGTGTCATCCAAAACCGGCCCAGTTGCGGT
GGACCCAACGCTGCGCCGTCGTATCGAACCGCACGAGTTCGAAGTATTTTTCGATCCTCGCGAACTGCGTAAAGAGACCTGTT
TACTTTACGAAATCAATTGGGGTGGCCGCCACTCGATTTGGCGCCACACGTCTCAGAATACAAACAAACATGTCGAAGTAAAT
TTCATCGAAAAATTTACAACGGAGCGTTATTTCTGTCCCAATACTCGTTGTTCTATTACATGGTTTTTATCATGGAGTCCCTG
CGGGGAATGCTCTCGTGCGATCACGGAGTTTCTGTCGCGTTATCCGAACGTAACGCTTTTTATCTATATTGCTCGTCTTTACC
ACTTGGCTAACCCTCGTAACCGCCAAGGACTTCGCGACCTGATTTCGAGTGGAGTAACCATCCAGATCATGACTGAGCAAGAA
TCTGGATACTGTTGGCACAATTTCGTAAACTACTCGCCATCGAATGAGAGCCATTGGCCTCGCTACCCCCATCTTTGGGTACG
TTTGTATGTCTTGGAACTTTATTGTATCATTTTAGGGCTGCCACCTTGTCTTAATATTTTGCGCCGCAAACAGAGTCAGCTGA
CATCTTTCACAATTGCTTTACAGTCATGCCACTACCAACGTCTTCCACCGCACATTCTGTGGGCCACGGGCTTAAAATCGGGC
GGTAGCTCAGGCGGGAGTTCAGGCTCAGAAACGCCGGGAACTAGCGAATCCGCAACGCCAGAGTCTTCAGGTGGTTCGTCAGG
TGGTTCAGACAAGAAATATAGCATCGGCCTGGCCATCGGCACAAATAGCGTCGGATGGGCGGTGATCACTGATGAATATAAAG
TTCCGTCTAAAAAGTTCAAGGTACTGGGTAATACAGATCGCCATAGTATCAAAAAGAACTTAATCGGTGCGCTTCTGTTCGAT
TCCGGCGAAACCGCAGAAGCAACACGTCTGAAACGCACCGCTCGTCGCCGTTACACCCGTCGTAAAAACCGCATCTGCTACCT
GCAAGAAATCTTCTCTAACGAAATGGCTAAAGTAGATGACAGCTTTTTCACCGTCTGGAAGAATCATTTCTGGTGGAAGAAG
ATAAAAAGCACGAACGTCATCCAATCTTCGGCAACATTGTGGACGAAGTAGCGTATCACGAAAAATACCCGACTATCTATCAC
CTGCGCAAAAAGCTGGTCGATTCGACGGATAAGGCCGATCTGCGTCTGATCTATCTGGCCTTAGCGCATATGATTAAGTTCCG
TGGTCATTTCCTGATCGAAGGCGACCTGAATCCAGACAACAGCGATGTAGACAAACTGTTCATCCAGCTGGTGCAAACCTATA
ACCAGCTGTTTGAAGAAAACCCAATTAATGCTAGCGGTGTTGACGCGAAAGCGATCTTGTCCGCACGCCTGTCCAAATCCCGT
CGTCTGGAAAACTTAATTGCGCAACTGCCGGGTGAGAAGAAAAACGGACTGTTCGGCAATCTGATCGCTCTTAGCTTGGGACT
GACCCCGAACTTCAAAAGCAACTTCGATCTGGCAGAGGACGCAAAACTTCAACTTAGCAAAGATACGTATGACGATGACTTGG
ATAACTTACTGGCCCAGATCGGAGATCAGTACGCTGATCTGTTTCTGGCGGCAAAGAACTTATCAGACGCTATTCTCCTGTCT
GATATTCTTCGTGTGAATACCGAAATCACCAAAGCACCGCTTTCTGCATCCATGATTAAACGCTATGACGAACATCACCAAGA
TCTGACTCTTCTGAAAGCGCTGGTACGGCAACAACTGCCGGAGAAGTACAAGGAGATCTTCTTTGACCAATCCAAAAACGGCT
ACGCGGGTTATATTGACGGGGGTGCAAGCCAAGAGGAGTTCTACAAATTCATCAAGCCAATCTTAGAAAAAATGGATGGCACG
GAAGAATTACTTGTTAAACTGAATCGTGAGGATCTGCTTCGTAAACAGCGTACCTTCGACAACGGTAGCATTCCGCACCAGAT
CCACTTAGGTGAACTGCACGCTATCCTGCGTCGCCAAGAGGATTTTTACCCGTTCCTGAAAGATAATCGTGAAAAAATCGAAA
AAATCCTGACCTTTCGTATCCCGTATTATGTCGGCCCGCTGGCGCGTGGCAACTCCCGTTTCGCGTGGATGACTCGCAAATCC
GAAGAAACTATTACCCCGTGGAACTTCGAGGAAGTGGTTGACAAAGGCGCAAGCGCCCAATCCTTCATCGAGCGCATGACTAA
CTTTGATAAAAACCTGCCGAACGAAAAGGTACTGCCGAAACACTCCCTTCTGTACGAATACTTCACCGTGTACAACGAGCTGA
CTAAAGTAAAGTATGTGACTGAGGGCATGCGTAAACCTGCATTCCTGAGCGGTGAACAGAAAAAAGCAATTGTTGATTTACTG
TTTAAAACCAACCGTAAAGTAACCGTTAAACAGCTGAAAGAGGACTACTTCAAGAAAATCGAATGCTTCGACTCCGTCGAGAT
TAGTGGAGTTGAAGATCGTTTTAATGCAAGTTTAGGCACGTATCACGATTTATTAAAGATCATTAAAGACAAAGATTTCTTGG
ACAACGAAGAAAATGAGGACATCTTAGAGGACATCGTCCTGACCCTGACTCTGTTCGAAGATCGTGAAATGATTGAAGAACGC
CTTAAGACGTATGCTCACCTGTTTGACGATAAAGTAATGAAACAACTGAAACGTCGCCGTTATACTGGCTGGGGCCGTCTGAG
CCGTAAACTGATTAACGGTATCCGTGACAAACAGTCCGGTAAAACTATTCTGGACTTCCTGAAATCTGACGGCTTCGCAAACC
GTAACTTCATGCAACTGATTCACGACGATTCCCTGACCTTCAAAGAGGACATCCAGAAAGCTCAGGTTTCTGGTCAAGGTGAT
TCTCTGCACGAGCATATCGCCAATTTAGCAGGTAGTCCGGCGATCAAAAAAGGTATCCTGCAAACCGTGAAAGTGGTGGATGA
GCTTGTGAAAGTTATGGGTCGTCACAAACCGGAAAACATTGTTATCGAGATGGCTCGTGAAAACCAAACGACCCAGAAGGGAC
AGAAAAACTCCCGCGAACGCATGAAACGTATCGAGGAGGGTATTAAAGAACTTGGCTCTCAGATTCTGAAAGAACACCCTGTT
GAAAATACCCAACTGCAAAATGAAAAACTGTACCTGTACTACCTGCAAAATGGTCGTGACATGTATGTAGATCAGGAGCTGGA
CATCAACCGCCTCTCCGATTACGACGTTGACCACATTGTTCCGCAGTCTTTTCTGAAAGATGATTCCATTGATAACAAAGTAC
TCACCCGTAGCGATAAAAACCGTGGGAAGAGTGACAACGTTCCATCGGAAGAAGTAGTTAAGAAAATGAAGAACTATTGGCGT
CAACTGCTTAACGCGAAACTGATTACTCAACGTAAATTTGATAACCTGACCAAAGCTGAACGTGGCGGTTTGTCTGAGCTGGA
TAAGGCGGGTTTTATTAAACGTCAACTGGTAGAAACTCGCCAGATTACAAAACATGTTGCTCAGATTCTGGACTCTCGTATGA
ACACTAAATACGATGAAAATGACAAACTGATCCGCGAAGTTAAGGTTATTACCCTGAAATCTAAGCTGGTTTCCGACTTCCGT
AAAGATTTCCAATTCTATAAAGTGCGCGAGATTAACAACTATCACCACGCGCACGACGCATATCTGAATGCAGTTGTTGGCAC
GGCACTGATCAAAAAATATCCGAAACTGGAAAGCGAATTTGTGTACGGCGATTATAAAGTTTACGACGTGCGCAAAATGATCG
CCAAATCTGAACAGGAAATTGGCAAAGCAACCGCTAAATACTTTTTCTACTCAAACATTATGAATTTCTTCAAAACCGAAATC
ACCTTAGCGAATGGCGAAATTCGTAAACGCCCTCTGATCGAAACCAACGGCGAAACGGGTGAGATCGTGTGGGACAAAGGTCG
TGATTTCGCTACTGTCCGCAAAGTTCTGTCCATGCCTCAAGTAAACATCGTTAAAAAGACTGAGGTACAGACTGGCGGTTTCA
GCAAGGAATCCATTCTGCCGAAACGCAACTCCGACAAACTGATCGCGCGTAAGAAAGACTGGGATCCGAAGAAATACGGTGGC
TTCGATTCTCCAACCGTGGCATACAGCGTTCTGGTAGTCGCCAAAGTCGAAAAGGGTAAATCAAAAAAACTGAAATCAGTGAA
AGAACTTTTAGGCATCACCATTATGGAACGTAGCTCTTTCGAAAAAAACCCGATTGACTTCCTCGAAGCGAAGGGGTACAAGG
AAGTAAAGAAAGATCTGATTATCAAACTGCCGAAGTATTCCCTGTTCGAACTGGAAAATGGTCGTAAACGTATGTTAGCGTCT
GCGGGTGAACTGCAAAAAGGGAACGAATTGGCCCTTCCGTCCAAGTACGTGAACTTCCTGTATCTGGCCTCGCACTACGAGAA

ACTGAAAGGTAGTCCGGAAGATAATGAGCAGAAACAGCTGTTCGTGGAACAGCACAAACACTATCTGGACGAGATTATTGAAC
AGATTTCTGAGTTTAGCAAACGCGTAATTCTGGCGGACGCGAATCTGGATAAAGTCCTGAGCGCCTACAATAAACACCGTGAT
AAACCGATCCGTGAACAGGCAGAAAACATCATTCACCTGTTCACGCTGACTAATCTTGGTGCTCCGGCAGCCTTCAAATACTT
CGACACCACGATCGATCGTAAACGTTACACCTCCACTAAAGAAGTCTTAGATGCAACTCTTATTCACCAGAGCATCACTGGCC
TGTATGAAACTCGTATTGATCTGAGTCAGTTGGGCGGTGACAGTGGGGGCAGCGGAGGTAGCGGAGGCAGCACTAATTTGTCG
GATATTATTGAGAAGGAAACAGGAAAGCAATTGGTTATCCAGGAATCCATTTTAATGCTTCCGGAAGAAGTGGAAGAGGTGAT
CGGAAATAAGCCAGAGAGCGATATCTTAGTTCATACGGCCTATGATGAAAGCACTGACGAGAACGTTATGCTGTTAACATCGG
ACGCTCCCGAGTACAAGCCGTGGGCCCTGGTTATCCAGGACAGTAATGGCGAAAATAAAATTAAAATGTTATAA

<u>Superfolder Green Fluorescent Protein (*sfGFP*)</u>

ATGAGTAAAGGTGAGGAATTATTTACTGGTGTTGTTCCGATCTTAGTTGAACTGGACGGCGATGTTAACGGTCATAAATTCAG
TGTTCGTGGTGAAGGTGAAGGTGATGCAACCAACGGTAAGCTGACCCTGAAATTCATCTGCACTACTGGAAAATTACCAGTAC
CGTGGCCTACTCTGGTGACTACCCTGACCTATGGTGTTCAGTGTTTTTCTCGTTACCCTGACCACATGAAGCAACATGATTTC
TTCAAATCTGCAATGCCGGAAGGTTATGTACAGGAGCGCACCATTTCTTTCAAAGACGATGGCACGTATAAAACCCGTGCAGA
GGTTAAATTTGAAGGTGACACTCTGGTGAATCGTATTGAACTGAAAGGCATTGATTTCAAAGAGGACGGCAATATTTTAGGCC
ACAAACTGGAATATAACTTCAACTCCCATAACGTTTACATCACCGCAGACAAACAGAAGAACGGTATCAAAGCTAACTTCAAA
ATTCGCCATAACGTTGAAGATGGTAGCGTACAGCTGGCGGATCATTACCAACAGAACACTCCGATTGGAGATGCTCCTGTTTT
ACTGCCGGATAACCACTACCTGTCCACCCAGTCTAAACTGTCGAAGGATCCGAACGAAAAGCGCGACCACATGGTGTTATTAG
AGTTCGTTACCGCTAGTGGTATCACGCACGGTATGGATGAACTCTACAAATAA

<u>Primase</u>

ATGAAACTGGCACCGAACGTAAAACAGCAGTCACGCGGCATAAAACACAAAGAAACAGAAGTCATTATTTTTGCGGGTAGTGA
TGCCTGGTCACACGCAAAACAATGGCAGGAACATGACGCGCGTATGGCCGGAGATAATGAGCCTCCTGTGTGGCTTGGGGAGC
AGCAGTTATCCGAACTGGATAAGCTGCAAATTGTGCCGGAAGGCAGAAAATCCGTGCGCATATTCAGGGCCGGATATCTTGCG
CCAGTAATGATAAAGGCGATTGGTCAGAAGCTGGCGGCGGCAGGCGTACAGGATGCAAATTTTTACCCTGATGGTATGCACGG
TCAGAAGGTGGAGAACTGGCGCGAATATCTGGCCCGTGAGCGCCAGAATCTTTCTGATGGTCTGGTCATTGAGCTTCCGGTAA
AGCAAAAGGCGCAACTTTCGCAGATGGCGGACAGTGAGCGCGCGCAGCTGCTTGCCGATCGCTTTGATGGCGTTTGCGTACAT
CCTGAAAGTGAAATCGTTCACGTATGGCGCGGCGGGGTATGGTGTCCGGTCAGCACAATGGAGCTGAGCCGCGAAATGGTGGC
GATCTATTCAGAGCACAGGGCCACTTTCAGCAAGCGCGTAATCAATAACGCCGTGGAAGCGTTAAAAGTTATTGCCGAACCAA
TGGGCGAGCCGTCCGGCGATTTGCTGCCGTTCGCCAATGGTGCGCTTGACCTGAAAACGGGGGAATTTTCCCCGCACACGCCG
GAGAACTGGATCACCACGCACAACGGCATTGAGTACACGCCACCAGCACCCGGGGAGAACATCCGCGATAACGCGCCAAACTT
TCATAAATGGCTTGAGCACGCAGCCGGAAAAGACCCGCGCAAGATGATGCGTATATGTGCCGCGCTGTACATGATTATGGCGA
ACCGGTACGACTGGCAGATGTTTATTGAGGCCACCGGAGACGGCGGGAGCGGTAAAAGTACATTCACACACATAGCCAGCCTT
CTGGCAGGGAAACAAAACACGGTAAGCGCTGAAATGACATCGCTTGATGATGCTGGTGGGCGTGCGCAGGTTGTCGGGAGTCG
TCTTATCGTCCTGGCAGACCAGCCGAAATATACAGGCGAAGGAACGGGCATCAAGAAAATCACGGGCGGCGACCCCGTGGAAA
TTAACCCGAAATATGAAAAGCGTTTTACGGCGGTAATCAGGGCGGTGGTGCTGGCAACCAATAACAATCCGATGATATTCACC
GAACGGGCCGGAGGTGTGGCACGTCGTCGGGTGATATTCCGGTTCGATAACATCGTAAGCGAGGCAGAAAAAGACAGGGAGCT
ACCGGAAAAGATCGCGGCTGAAATCCCTGTCATTATCCGCCGCTTGCTGGCGAACTTTGCCGACCCTGAAAAGGCACGGGCTT
TACTCATTGAACAGCGTGACGGTGATGAAGCACTGGCAATAAAGCAACAGACGGATCCGGTTATTGAGTTTTGCCAGTTCCTG
AATTTTCTGGAGGAAGCACGCGGCCTGATGATGGGCGGCGGTGGCGATTCAGTGAAGTACACGACCAGAAACAGCCTTTACCG
CGTCTATCTGGCGTTTATGGCGTACGCAGGCAGGAGCAAACCGCTAAACGTAAATGACTTTGGCAAGGCTATGAAGCCAGCCG
CGAAAGTTTACGGACATGAATATATTACGCGGAAAGTTAAAGGAGTAACGCAGACTAACGCAATAACAACAGACGATTGCGAC
GCGTTTTTATAA

<u>Origin of replication (Primase)</u>

TTTGTTGCAATGGCTGTCTACCCTGTCTACCTGAGTAAAGAAAAATACATTTAATTCAGTATATTAACTTGGGTAGACAGCCT
TTTTTTACTGTCTACCTTCTGTCTACCCTCTCTACCTGATTTTACCTGAATCAGACAGGGAGGTAGACACGGGGTAGACAGTG
GATAAAAGCACTCTACCCCACTGAAAGCAGTGCCATTACTGGCATGGTTGCCAGTAAGGTTGATAAGGTAGACAAGGGGAGGG
ACAACTCAAAACTTTTTAAACGAGGGGGTAAAA

GpJ 1A2

ATGGGTAAAGGAAGCAGTAAGGGGCATACCCCGCGCGAAGCGAAGGACAACCTGAAGTCCACGCAGTTGCTGAGTGTGATCGA
TGCCATCAGCGAAGGGCCGATTGAAGGTCCGGTGGATGGCTTAAAAAGCGTGCTGCTGAACAGTACGCCGGTGCTGGACACTG
AGGGGAATACCAACATATCCGGTGTCACGGTGGTGTTCCGGGCTGGTGAGCAGGAGCAGACTCCGCCGGAGGGATTTGAATCC
TCCGGCTCCGAGACGGTGCTGGGTACGGAAGTGAAATATGACACGCCGATCACCCGCACCATTACGTCTGCAAACATCGACCG
TCTGCGCTTTACCTTCGGTGTACAGGCACTGGTGGAAACCACCTCAAAGGGTGACAGGAATCCGTCGGAAGTCCGCCTGCTGG
TTCAGATACAACGTAACGGTGGCTGGGTGACGGAAAAAGACATCACCATTAAGGGCAAAACCACCTCGCAGTATCTGGCCTCG
GTGGTGATGGGTAACCTGCCGCCGCGCCCGTTTAATATCCGGATGCGCAGGATGACGCCGGACAGCACCACAGACCAGCTGCA
GAACAAAACGCTCTGGTCGTCATACACTGAAATCATCGATGTGAAACAGTGCTACCCGAACACGGCACTGGTCGGCGTGCAGG
TGGACTCGGAGCAGTTCGGCAGCCAGCAGGTGAGCCGTAATTATCATCTGCGCGGGCGTATTCTGCAGGTGCCGTCGAACTAT
AACCCGCAGACGCGGCAATACAGCGGTATCTGGGACGGAACGTTTAAACCGGCATACAGCAACAACATGGCCTGGTGTCTGTG
GGATATGCTGACCCATCCGCGCTACGGCATGGGGAAACGTCTTGGTGCGGCGGATGTGGATAAATGGGCGCTGTATGTCATCG
GCCAGTACTGCGACCAGTCAGTGCCGGACGGCTTTGGCGGCACGGAGCCGCGCATCACCTGTAATGCGTACCTGACCACACAG
CGTAAGGCGTGGGATGTGCTCAGCGATTTCTGCTCGGCGATGCGCTGTATGCCGGTATGGAACGGGCAGACGCTGACGTTCGT
GCAGGACCGACCGTCGGATAAGACGTGGACCTATAACCGCAGTAATGTGGTGATGCCGGATGATGGCGCGCCGTTCCGCTACA
GCTTCAGCGCCCTGAAGGACCGCCATAATGCCGTTGAGGTGAACTGGATTGACCCGAACAACGGCTGGGAGACGGCGACAGAG
CTTGTTGAAGATACGCAGGCCATTGCCCGTTACGGTCGTAATGTTACGAAGATGGATGCCTTTGGCTGTACCAGCCGGGGGCA
GGCACACCGCGCCGGGCTGTGGCTGATTAAAACAGAACTGCTGGAAACGCAGACCGTGGATTTCAGCGTCGGCGCAGAAGGGC
TTCGCCATGTACCGGGCGATGTTATTGAAATCTGCGATGATGACTATGCCGGTATCAGCACCGGTGGTCGTGTGCTGGCGGTG
AACAGCCAGACCCGGACGCTGACGCTCGACCGTGAAATCACGCTGCCATCCTCCGGTACCGCGCTGATAAGCCTGGTTGACGG
AAGTGGCAATCCGGTCAGCGTGGAGGTTCAGTCCGTCACCGACGGCGTGAAGGTAAAAGTGAGCCGTGTTCCTGACGGTGTTG
CTGAATACAGCGTATGGGAGCTGAAGCTGCCGACGCTGCGCCAGCGACTGTTCCGCTGCGTGAGTATCCGTGAGAACGACGAC
GGCACGTATGCCATCACCGCCGTGCAGCATGTGCCGGAAAAAGAGGCCATCGTGGATAACGGGGCGCACTTTGACGGCGAACA
GAGTGGCACGGTGAATGGTGTCACGCCGCCAGCGGTGCAGCACCTGACCGCAGAAGTCACTGCAGACAGCGGGGAATATCAGG
TGCTGGCGCGATGGGACACACCGAAGGTGGTGAAGGGCGTGAGTTTCCTGCTCCGTCTGACCGTAACAGCGGACGACGGCAGT
GAGCGGCTGGTCAGCACGGCCCGGACGACGGAAACCACATACCGCTTCACGCAACTGGCGCTGGGGAACTACAGGCTGACAGT
CCGGGCGGTAAATGCGTGGGGGCAGCAGGGCGATCCGGCGTCGGTATCGTTCCGGATTGCCGCACCGGCAGCACCGTCGAGGA
TTGAGCTGACGCCGGGCTATTTTCAGATAACCGCCACGCCGCATCTTGCCGTTTATGACCCGACGGTACAGTTTGAGTTCTGG
TTCTCGGAAAAGCAGATTGCGGATATCAGACAGGTTGAAACCAGCACGCGTTATCTTGGTACGGCGCTGTACTGGATAGCCGC
CAGTATCAATATCAAACCGGGCCATGATTATTACTTTTATATCCGCAGTGTGAACACCGTTGGCAAATCGGCATTCGTGGAGG
CCGTCGGTCGGGCGAGCGATGATGCGGAAGGTTACCTGGATTTTTTCAAAGGCAAGATAACCGAATCCCATCTCGGCAAGGAG
CTGCTGGAAAAAGTCGAGCTGACGGAGGATAACGCCAGCAGACTGGAGGAGTTTTCGAAAGAGTGGAAGGATGCCAGTGATAA
GTGGAATGCCATGTGGGCTGTCAAAATTGAGCAGACCAAAGACGGCAAACATTATGTCGCGGGTATTGGCCTCAGCATGGAGG
ACACGGAGGAAGGCAAACTGAGCCAGTTTCTGGTTGCCGCCAATCGTATCGCATTTATTGACCCGGCAAACGGGAATGAAACG
CCGATGTTTGTGGCGCAGGGCAACCAGATATTCATGAACGACGTGTTCCTGAAGCGCCTGACGGCCCCCACCATTACCAGCGG
CGGCAATCCTCCGGCCTTTTCCCTGACACCGGACGGAAAGCTGACCGCTAAAAATGCGGATATCAGTGGCAACGTAAACGCCA
ATAGCGGTACTTTGAATAATGTGACTATCAATGAAAATTGCCGCGTGCTTGGCAAGCTCAGCGCAAATCAAATCGAGGGTGAC
CTGGTCAAGACCGTTGGGAAGGCATTTCCGCGCGATAGCCGCGCCCCTGAACGTTGGCCTAGCGGCACTATCACGGTTCGTGT
GTACGATGACCAACCTTTCGATCGTCAAATCGTGATCCCAGCAGTTGCCTTTAGTGGAGCGAAGCACGAAAAGGAACACACCG
ACATCTATTCTTCGTGTCGCTTAATCGTCCGTAAGAATGGCGCGGAGATCTACAATCGCACGGCCCTTGACAACACATTGATC
TATTCAGGAGTGATCGACATGCCCGCAGGCCATGGACATATGACCCTTGAATTCAGCGTTAGTGCCTGGTTGGTCAACAATTG
GTACCCAACTGCGTCCATTAGTGACTTATTAGTGGTAGTTATGAAGAAGGCTACAGCGGGAATTACAATCTCGTGA

GpJ A8

ATGGGTAAAGGAAGCAGTAAGGGGCATACCCCGCGCGAAGCGAAGGACAACCTGAAGTCCACGCAGTTGCTGAGTGTGATCGA
TGCCATCAGCGAAGGGCCGATTGAAGGTCCGGTGGATGGCTTAAAAAGCGTGCTGCTGAACAGTACGCCGGTGCTGGACACTG
AGGGGAATACCAACATATCCGGTGTCACGGTGGTGTTCCGGGCTGGTGAGCAGGAGCAGACTCCGCCGGAGGGATTTGAATCC
TCCGGCTCCGAGACGGTGCTGGGTACGGAAGTGAAATATGACACGCCGATCACCCGCACCATTACGTCTGCAAACATCGACCG
TCTGCGCTTTACCTTCGGTGTACAGGCACTGGTGGAAACCACCTCAAAGGGTGACAGGAATCCGTCGGAAGTCCGCCTGCTGG
TTCAGATACAACGTAACGGTGGCTGGGTGACGGAAAAAGACATCACCATTAAGGGCAAAACCACCTCGCAGTATCTGGCCTCG
GTGGTGATGGGTAACCTGCCGCCGCGCCCGTTTAATATCCGGATGCGCAGGATGACGCCGGACAGCACCACAGACCAGCTGCA
GAACAAAACGCTCTGGTCGTCATACACTGAAATCATCGATGTGAAACAGTGCTACCCGAACACGGCACTGGTCGGCGTGCAGG
TGGACTCGGAGCAGTTCGGCAGCCAGCAGGTGAGCCGTAATTATCATCTGCGCGGGCGTATTCTGCAGGTGCCGTCGAACTAT
AACCCGCAGACGCGGCAATACAGCGGTATCTGGGACGGAACGTTTAAACCGGCATACAGCAACAACATGGCCTGGTGTCTGTG

GGATATGCTGACCCATCCGCGCTACGGCATGGGGAAACGTCTTGGTGCGGCGGATGTGGATAAATGGGCGCTGTATGTCATCG
GCCAGTACTGCGACCAGTCAGTGCCGGACGGCTTTGGCGGCACGGAGCCGCGCATCACCTGTAATGCGTACCTGACCACACAG
CGTAAGGCGTGGGATGTGCTCAGCGATTTCTGCTCGGCGATGCGCTGTATGCCGGTATGGAACGGGCAGACGCTGACGTTCGT
GCAGGACCGACCGTCGGATAAGACGTGGACCTATAACCGCAGTAATGTGGTGATGCCGGATGATGGCGCGCCGTTCCGCTACA
GCTTCAGCGCCCTGAAGGACCGCCATAATGCCGTTGAGGTGAACTGGATTGACCCGAACAACGGCTGGGAGACGGCGACAGAG
CTTGTTGAAGATACGCAGGCCATTGCCCGTTACGGTCGTAATGTTACGAAGATGGATGCCTTTGGCTGTACCAGCCGGGGGCA
GGCACACCGCGCCGGGCTGTGGCTGATTAAAACAGAACTGCTGGAAACGCAGACCGTGGATTTCAGCGTCGGCGCAGAAGGGC
TTCGCCATGTACCGGGCGATGTTATTGAAATCTGCGATGATGACTATGCCGGTATCAGCACCGGTGGTCGTGTGCTGGCGGTG
AACAGCCAGACCCGGACGCTGACGCTCGACCGTGAAATCACGCTGCCATCCTCCGGTACCGCGCTGATAAGCCTGGTTGACGG
AAGTGGCAATCCGGTCAGCGTGGAGGTTCAGTCCGTCACCGACGGCGTGAAGGTAAAAGTGAGCCGTGTTCCTGACGGTGTTG
CTGAATACAGCGTATGGGAGCTGAAGCTGCCGACGCTGCGCCAGCGACTGTTCCGCTGCGTGAGTATCCGTGAGAACGACGAC
GGCACGTATGCCATCACCGCCGTGCAGCATGTGCCGGAAAAAGAGGCCATCGTGGATAACGGGGCGCACTTTGACGGCGAACA
GAGTGGCACGGTGAATGGTGTCACGCCGCCAGCGGTGCAGCACCTGACCGCAGAAGTCACTGCAGACAGCGGGGAATATCAGG
TGCTGGCGCGATGGGACACACCGAAGGTGGTGAAGGGCGTGAGTTTCCTGCTCCGTCTGACCGTAACAGCGGACGACGGCAGT
GAGCGGCTGGTCAGCACGGCCCGGACGACGGAAACCACATACCGCTTCACGCAACTGGCGCTGGGGAACTACAGGCTGACAGT
CCGGGCGGTAAATGCGTGGGGGCAGCAGGGCGATCCGGCGTCGGTATCGTTCCGGATTGCCGCACCGGCAGCACCGTCGAGGA
TTGAGCTGACGCCGGGCTATTTTCAGATAACCGCCACGCCGCATCTTGCCGTTTATGACCCGACGGTACAGTTTGAGTTCTGG
TTCTCGGAAAAGCAGATTGCGGATATCAGACAGGTTGAAACCAGCACGCGTTATCTTGGTACGGCGCTGTACTGGATAGCCGC
CAGTATCAATATCAAACCGGGCCATGATTATTACTTTTATATCCGCAGTGTGAACACCGTTGGCAAATCGGCATTCGTGGAGG
CCGTCGGTCGGGCGAGCGATGATGCGGAAGGTTACCTGGATTTTTTCAAAGGCAAGATAACCGAATCCCATCTCGGCAAGGAG
CTGCTGGAAAAAGTCGAGCTGACGGAGGATAACGCCAGCAGACTGGAGGAGTTTTCGAAAGAGTGGAAGGATGCCAGTGATAA
GTGGAATGCCATGTGGGCTGTCAAAATTGAGCAGACCAAAGACGGCAAACATTATGTCGCGGGTATTGGCCTCAGCATGGAGG
ACACGGAGGAAGGCAAACTGAGCCAGTTTCTGGTTGCCGCCAATCGTATCGCATTTATTGACCCGGCAAACGGGAATGAAACG
CCGATGTTTGTGGCGCAGGGCAACCAGATATTCATGAACGACGTGTTCCTGAAGCGCCTGACGGCCCCCACCATTACCAGCGG
CGGCAATCCTCCGGCCTTTTCCCTGACACCGGACGGAAAGCTGACCGCTAAAAATGCGGATATCAGTGGCAGTGTGAATGCGA
ACTCAGGGACGCTCAACAACGTCACGATTAATGAGAACTGTCAGATTAAGGGGAAACTGTCAGCCAACCAGATTGAAGGTGAT
ATTGTCAAAACGGTCAGCAAGTCTTTCCCCCGCACGAACAGTTATGCCAGCGGCACCATCACGGTAAGAATCAGTGATGATCA
GAAGTTTGACCGGCAGGTCATGATACCGCCAGTGTTATTCCGCGGTGGTAAGCATGAGAATTTCAACAGTAATAACCAACAGT
CATACTGGTATTCAACCTGCCGGTTAAGAGTGACCCGCAATGGTCAGGAGATTTTTAATCAGTCCACGACGGATGCTCAGGGC
GTATTTTCCTCAGTTATAGATATGCCTGCCGGACAGGGGACACTGACACTGACATTCACCGTATCTTCATCAGGAGCGAATAA
CTGGACACCAACAACCAGTATCAGCGATCTGCTGGTTGTGGTGATGAAGAAATCCACAGCAGGTATCAGTATCAGCTGA

<u>Stf λ-P2 chimera</u>

ATGGCAGTAAAGATTTCAGGAGTCCTGAAAGACGGCACAGGAAAACCGGTACAGAACTGCACCATTCAGCTGAAAGCCAGACG
TAACAGCACCACGGTGGTGGTGAACACGGTGGGCTCAGAGAATCCGGATGAAGCCGGGCGTTACAGCATGGATGTGGAGTACG
GTCAGTACAGTGTCATCCTGCAGGTTGACGGTTTTCCACCATCGCACGCCGGGACCATCACCGTGTATGAAGATTCACAACCG
GGGACGCTGAATGATTTTCTCTGTGCCATGACGGAGGATGATGCCCGGCCGGAGGTGCTGCGTCGTCTTGAACTGATGGTGGA
AGAGGTGGCGCGTAACGCGTCCGTGGTGGCACAGAGTACGGCAGACGCGAAGAAATCAGCCGGCGATGCCAGTGCATCAGCTG
CTCAGGTCGCGGCCCTTGTGACTGATGCAACTGACTCAGCACGCGCCGCCAGCACGTCCGCCGGACAGGCTGCATCGTCAGCT
CAGGAAGCGTCCTCCGGCGCAGAAGCGGCATCAGCAAAGGCCACTGAAGCGGAAAAAAGTGCCGCAGCCGCAGAGTCCTCAAA
AAACGCGGCGGCCACCAGTGCCGGTGCGGCGAAAACGTCAGAAACGAATGCTGCAGCGTCACAACAATCAGCCGCCACGTCTG
CCTCCACCGCGGCCACGAAAGCGTCAGAGGCCGCCACTTCAGCACGAGATGCGGTGGCCTCAAAAGAGGCAGCAAAATCATCA
GAAACGAACGCATCATCAAGTGCCGGTCGTGCAGCTTCCTCGGCAACGGCGGCAGAAAATTCTGCCAGGGCGGCAAAAACGTC
CGAGACGAATGCCAGGTCATCTGAAACAGCAGCGGAACGGAGCGCCTCTGCCGCGGCAGACGCAAAAACAGCGGCGGCGGGGA
GTGCGTCAACGGCATCCACGAAGGCGACAGAGGCTGCGGGAAGTGCGGTATCAGCATCGCAGAGCAAAAGTGCGGCAGAAGCG
GCGGCAATACGTGCAAAAAATTCGGCAAAACGTGCAGAAGATATAGCTTCAGCTGTCGCGCTTGAGGATGCGGACACAACGAG
AAAGGGGATAGTGCAGCTCAGCAGTGCAACCAACAGCACGTCTGAAACGCTTGCTGCAACGCCAAAGGCGGTTAAGGTGGTAA
TGGATGAGACTAATCGTAAAGCGCCATTAAACAGCCCTGCACTGACCGGAACGCCAACGACGCCAACTGCGCGACAGGGAACG
AATAATACTCAGATCGCAAACACGGCTTTCGTTATGGCCGCGATTGCCGCCCTTGTAGACTCGTCGCCTGACGCACTGAATAC
GCTGAACGAGCTGGCGGCGGCGCTGGGCAATGACCCGAATTTTGCTACCACCATGACTAATGCGCTTGCGGGTAAGCAACCGA
AAGATGCTACCCTGACGGCGCTGGCGGGGCTTGCTACTGCGGCAGACAGGTTTCCGTATTTTACGGGGAATGATGTTGCCAGC
CTGGCGACCCTGACAAAAGTCGGGCGGGATATTCTGGCTAAATCGACCGTTGCCGCCGTTATCGAATATCTCGGTTTACAGGA
AACGGTAAACCGAGCCGGGAACGCCGTGCAAAAAAATGGCGATACCTTGTCCGGTGGACTTACTTTTGAAAACGACTCAATCC
TTGCCTGGATTCGAAATACTGACTGGGCGAAGATTGGATTTAAAAATGATGCCGATGGTGACACTGATTCATACATGTGGTTT
GAAACGGGGGATAACGGCAATGAATATTTCAAATGGAGAAGCCGCCAGAGTACCACAACAAAAGACCTGATGACGTTGAAATG
GGATGCACTAAATATTCTTGTTAATGCCGTCATTAATGGCTGTTTTGGAGTTGGTACGACGAATGCACTAGGTGGTAGCTCTA
TTGTTCTTGGTGATAATGATACCGGATTTAAACAGAATGGAGACGGTATTCTTGATGTTTATGCTAACAGTCAGCGTGTATTC
CGTTTTCAGAATGGAGTGGCTATTGCTTTTAAAAATATTCAGGCAGGTGATAGTAAAAAGTTCTCGCTATCCAGCTCTAATAC
ATCCACGAAGAATATTACCTTTAATTTATGGGGTGCTTCCACCCGTCCAGTGGTTGCAGAGTTAGGCGATGAGGCCGGATGGC

ATTTCTATAGCCAGCGAAATACAGATAACTCGGTAATATTTGCTGTTAACGGTCAGATGCAACCCAGCAACTGGGGAAATTTT
GATTCCCGCTATGTGAAAGATGTTCGCCTGGGTACGCGAGTTGTTCAATTGATGGCGCGAGGTGGTCGTTATGAAAAAGCCGG
ACACACGATTACCGGATTAAGAATCATTGGTGAAGTAGATGGCGATGATGAAGCCATCTTCAGGCCGATACAAAAATACATCA
ATGGCACATGGTATAACGTTGCGCAGGTGTAA

<u>Stf λ-K5 chimera</u>

ATGGCAGTAAAGATTTCAGGAGTCCTGAAAGACGGCACAGGAAAACCGGTACAGAACTGCACCATTCAGCTGAAAGCCAGACG
TAACAGCACCACGGTGGTGGTGAACACGGTGGGCTCAGAGAATCCGGATGAAGCCGGGCGTTACAGCATGGATGTGGAGTACG
GTCAGTACAGTGTCATCCTGCAGGTTGACGGTTTTCCACCATCGCACGCCGGGACCATCACCGTGTATGAAGATTCACAACCG
GGGACGCTGAATGATTTTCTCTGTGCCATGACGGAGGATGATGCCCGGCCGGAGGTGCTGCGTCGTCTTGAACTGATGGTGGA
AGAGGTGGCGCGTAACGCGTCCGTGGTGGCACAGAGTACGGCAGACGCGAAGAAATCAGCCGGCGATGCCAGTGCATCAGCTG
CTCAGGTCGCGGCCCTTGTGACTGATGCAACTGACTCAGCACGCGCCGCCAGCACGTCCGCCGGACAGGCTGCATCGTCAGCT
CAGGAAGCGTCCTCCGGCGCAGAAGCGGCATCAGCAAAGGCCACTGAAGCGGAAAAAAGTGCCGCAGCCGCAGAGTCCTCAAA
AAACGCGGCGGCCACCAGTGCCGGTGCGGCGAAAACGTCAGAAACGAATGCTGCAGCGTCACAACAATCAGCCGCCACGTCTG
CCTCCACCGCGGCCACGAAAGCGTCAGAGGCCGCCACTTCAGCACGAGATGCGGTGGCCTCAAAAGAGGCAGCAAAATCATCA
GAAACGAACGCATCATCAAGTGCCGGTCGTGCAGCTTCCTCGGCAACGGCGGCAGAAAATTCTGCCAGGGCGGCAAAAACGTC
CGAGACGAATGCCAGGTCATCTGAAACAGCAGCGGAACGGAGCGCCTCTGCCGCGGCAGACGCAAAAACAGCGGCGGCGGGGA
GTGCGTCAACGGCATCCACGAAGGCGACAGAGGCTGCGGGAAGTGCGGTATCAGCATCGCAGAGCAAAAGTGCGGCAGAAGCG
GCGGCAATACGTGCAAAAAATTCGGCAAAACGTGCAGAAGATATAGCTTCAGCTGTCGCGCTTGAGGATGCGGACACAACGAG
AAAGGGGATAGTGCAGCTCAGCAGTGCAACCAACAGCACGTCTGAAACGCTTGCTGCAACGCCAAAGGCGGTTAAGGTGGTAA
TGGATGAGACTAATCGTAAGGCACCTCTGGACAGTCCGGCACTGACCGGAACGCCAACAGCACCAACCGCGCTCAGGGGAACA
AACAATACCCAGATTGCGAACACCGCTTTTGTACTGGCCGCGATTGCAGATGTTATCGACGCGTCACCTGACGCACTGAATAC
GCTGAATGAACTGGCCGCAGCGCTCGGGAATGATCCAGATTTTGCTACCACCATGACTAACGCGCTTGCGGGTAAACAACCGA
AGAATGCGACACTGACGGCGCTGGCAGGGCTTTCCACGGCGAAAAATAAATTACCGTATTTTGCGGAAAATGATGCCGCCAGC
CTGACTGAACTGACTCAGGTTGGCAGGGATATTCTGGCAAAAAATTCCGTTGCAGATGTTCTTGAATACCTTGGGGCCGGTGA
GAATTCGGACGCAGCACTGCGCTCTCAAATCAGCAACCCAGAAGGCGCGATTCTGTACCCGGAACTGCAGATGGCGCGCTGGC
GTGATGAAGGCGACGTTCGTGGTTGGGGTGCCAAAGGTGATGGTGTAACCGACTCCACTGAAAACATCGCAGCATCCTTGAAC
TCTCAGAAAGCAGTTGTTGCCAGCGAAGGCGTTTTCAGTTCTTCTGGCATCAACTCCAACTACTGTAACCTGGATGGTCGCGG
ATCCGGTGTGCTCAGCCACCGTAGCTCTACTGGTAATTACCTGGTGTTTAACAATCCGCGTACTGGTCGTCTGAGCAATATCA
CTGTTGAATCTAACAAAGCGACCGATACCACTCAGGGCCAACAGGTGTCCCTGGCAGGTGGCAGTGACGTGACCGTGTCAGAT
GTCAACTTCTCCAACGTGAAAGGCACTGGTTTTAGCCTGATTGCCTACCCAAACGATGCTCCGCCGGATGGCCTGATGATCAA
AGGCATTCGCGGATCTTACAGCGGTTACGCGACCAACAAAGCAGCTGGTTGCGTCCTGGCGGATAGCTCCGTTAACAGCCTGA
TCGACAATGTGATCGCTAAGAATTACCCGCAATTCGGTGCTGTTGAATTAAAGGGCACTGCAAGCTACAACATTGTATCGAAC
GTTATCGGTGCGGATTGTCAGCACGTGACTTACAACGGCACTGAGGGACCGATCGCTCCTAGTAACAATCTGATCAAGGGCGT
TATGGCGAACAACCCGAAATACGCGGCAGTTGTGGCGGGTAAAGGCTCGACGAATCTGATCTCTGATGTACTGGTAGACTATT
CTACCAGCGATGCTCGTCAGGCGCATGGTGTTACCGTCGAAGGATCTGATAACGTGATTAACAACGTACTGATGTCCGGTTGC
GACGGAACTAATTCCCTGGGTCAGCGTCAAACCGCAACTATCGCGCGTTTCATCGGTACTGCAAATAACAACTATGCTAGCGT
GTTCCCATCCTATTCTGCCACTGGTGTGATCACGTTTGAGTCTGGCAGTACCCGTAACTTCGTCGAGGTTAAGCATCCGGGCC
GTCGCAACGATCTTCTGTCATCGGCAAGCACGATTGACGGCGCTGCGACCATCGACGGGACTTCTAACTCTAACGTAGTACAC
GCGCCTGCTCTGGGCCAATACATTGGCTCCATGAGTGGTCGCTTTGAATGGCGTATTAAGTCAATGAGCCTGCCGTCCGGCGT
ACTCACTAGCGCGGATAAATACCGTATGCTGGGTGACGGTGCTGTTAGCCTTGCTGTTGGCGGAGGAACTAGCAGTCAGGTGC
GCTTGTTCACCTCAGACGGTACTTCTCGCACTGTTTCTCTGACCAATGGTAACGTGCGCCTGAGCACGTCCTCTACTGGCTAT
TTACAGCTGGGTGCAGACGCAATGACTCCGGACTCCACTGGTACTTACGCGTTAGGCTCCGCATCTCGTGCTTGGAGTGGCGG
ATTCACTCAGGCAGCATTCACCGTTACTTCTGACGCACGTTGCAAAACTGAGCCTTTAACCATCTCTGACGCTTTACTGGATG
CTTGGAGTGAAGTGGACTTTGTCCAGTTCCAGTATCTGGATCGTGTTGAAGAGAAAGGTGCTGACTCCGCGCGTTGGCATTTC
GGAATCATCGCCCAGCGTGCTAAAGAGGCATTCGAACGTCACGGCATCGATGCGCATCGTTACGGTTTCTTATGCTTTGACTC
TTGGGACGATGTGTACGAAGAGGATGCAAATGGATCTCGCAAACTGATCACTCCGGCGGGTAGTCGCTATGGTATTCGCTATG
AGGAAGTTCTGATCCTCGAAGCAGCGCTGATGCGTCGCACGATCAAGCGCATGCAGGAAGCACTGGCTGCGTTACCGAAGTAA

<u>Stf λ-K1F chimera</u>

ATGGCAGTAAAGATTTCAGGAGTCCTGAAAGACGGCACAGGAAAACCGGTACAGAACTGCACCATTCAGCTGAAAGCCAGACG
TAACAGCACCACGGTGGTGGTGAACACGGTGGGCTCAGAGAATCCGGATGAAGCCGGGCGTTACAGCATGGATGTGGAGTACG
GTCAGTACAGTGTCATCCTGCAGGTTGACGGTTTTCCACCATCGCACGCCGGGACCATCACCGTGTATGAAGATTCACAACCG
GGGACGCTGAATGATTTTCTCTGTGCCATGACGGAGGATGATGCCCGGCCGGAGGTGCTGCGTCGTCTTGAACTGATGGTGGA
AGAGGTGGCtCGTAACGCGTCCGTGGTGGCACAGAGTACGGCAGACGCGAAGAAATCAGCCGGCGATGCCAGTGCATCAGCTG

CTCAGGTCGCGGCCCTTGTGACTGATGCAACTGACTCAGCACGCGCCGCCAGCACGTCCGCCGGACAGGCTGCATCGTCAGCT
CAGGAAGCGTCCTCCGGCGCAGAAGCGGCATCAGCAAAGGCCACTGAAGCGGAAAAAAGTGCCGCAGCCGCAGAGTCCTCAAA
AAACGCGGCGGCCACCAGTGCCGGTGCGGCGAAAACGTCAGAAACGAATGCTGCAGCGTCACAACAATCAGCCGCCACGTCTG
CCTCCACCGCGGCCACGAAAGCGTCAGAGGCCGCCACTTCAGCACGAGATGCGGTGGCCTCAAAAGAGGCAGCAAAATCATCA
GAAACGAACGCATCATCAAGTGCCGGTCGTGCAGCTTCCTCGGCAACGGCGGCAGAAAATTCTGCCAGGGCGGCAAAAACGTC
CGAGACGAATGCCAGGTCATCTGAAACAGCAGCGGAACGGAGCGCCTCTGCCGCGGCAGACGCAAAAACAGCGGCGGCGGGGA
GTGCGTCAACGGCATCCACGAAGGCGACAGAGGCTGCGGGAAGTGCGGTATCAGCATCGCAGAGCAAAAGTGCGGCAGAAGCG
GCGGCAATACGTGCAAAAAATTCGGCAAAACGTGCAGAAGATATAGCTTCAGCTGTCGCGCTTGAGGATGCGGACACAACGAG
AAAGGGGATAGTGCAGCTCAGCAGTGCAACCAACAGCACGTCTGAAACGCTTGCTGCAACGCCAAAGGCGGTTAAGGTGGTAA
TGGATGAGACTAATCGTAAGGCACCTCTGGACAGTCCGGCACTGACCGGAACGCCAACAGCACCAACCGCGCTCAGGGGAACA
AACAATACCCAGATTGCGAACACCGCTTTTGTACTGGCCGCGATTGCAGATGTTATCGACGCGTCACCTGACGCACTGAATAC
GCTGAATGAACTGGCCGCAGCGCTCGGGAATGATCCAGATTTTGCTACCACCATGACTAACGCGCTTGCGGGTAAACAACCGA
AGAATGCGACACTGACGGCGCTGGCAGGGCTTTCCACGGCGAAAAATAAATTACCGTATTTTGCGGAAAATGATGCCGCCAGC
CTGACTGAACTGACTCAGGTTGGCAGGGATATTCTGGCAAAAAATTCCGTTGCAGATGTTCTTGAATACCTTGGGGCCGGTGA
GAATTCGGACGCAGCACTGCGTTCCCAGATTTCTAACCCGGAAGGCGCAATCCTCTATCCGGAACTTCACCGCGCGCGTTGGT
TAGATGAGAAGGATGCTCGCGGCTGGGGTGCGAAGGGCGATGGCGTTACCGACGACACTGCAGCGCTGACTTCCGCCCTGAAC
GATACTCCGGTGGGTCAGAAAATCAACGGTAACGGTAAAACTTATAAAGTTACGTCCCTGCCGGACATCTCCCGCTTTATCAA
CACCCGTTTCGTGTATGAACGTATCCCAGGCCAGCCGCTGTACTACGCATCGGAAGAGTTCGTTCAGGGTGAGCTTTTTAAAA
TCACCGACACTCCGTATTATAACGCCTGGCCACAGGATAAGGCTTTCGTGTACGAAAACGTTATCTATGCTCCGTACATGGGT
TCCGACCGTCACGGTGTCAGCCGACTGCACGTAAGCTGGGTGAAATCGGGCGACGATGGTCAGACCTGGAGCACGCCTGAGTG
GCTGACCGACCTTCATCCGGACTATCCGACCGTTAACTATCACTGCATGAGCATGGGCGTCTGTCGCAACCGTCTGTTCGCAA
TGATCGAAACCCGTACGCTGGCAAAAAACGCTCTGACTAACTGCGCCCTGTGGGATCGTCCAATGAGCCGCTCTCTGCACCTG
ACGGGTGGTATTACCAAAGCAGCGAACCAGCGTTACGCCACCATTCACGTACCGGATCATGGTCTGTTCGTTGGTGACTTTGT
AAATTTCTCTAATTCTGCAGTTACCGGTGTGTCTGGCGACATGACCGTTGCGACCGTAATCGATAAGGACAATTTCACCGTCC
TGACCCCGAACCAGCAAACCTCTGATCTTAACAACGCTGGCAAGAACTGGCACATGGGCACTAGCTTTCACAAATCTCCGTGG
CGTAAAACCGATCTGGGCCTGATCCCGTCTGTAACTGAAGTGCACTCCTTCGCGACCATTGATAACAACGGTTTCGCTATGGG
TTATCACCAAGGTGATGTTGCACCGCGTGAAGTCGGCCTCTTTTATTTTCCGGACGCATTCAACAGCCCGTCCAACTACGTGC
GCCGTCAGATTCCGTCTGAATATGAACCGGACGCCTCCGAGCCGTGCATTAAGTACTATGACGGTGTGCTGTACCTGATTACC
CGTGGCACCCGTGGTGATCGTCTGGGTTCATCTCTGCATCGCTCCCGCGACATTGGTCAGACGTGGGAAAGTCTGCGCTTCCC
GCACAATGTTCATCACACCACCCTGCCGTTCGCGAAAGTCGGCGATGACCTGATCATGTTTGGCTCCGAACGTGCTGAAAACG
AATGGGAAGCGGGCGCCCCAGACGATCGCTACAAGGCATCTTACCCGCGCACCTTCTACGCGCGTCTGAACGTGAACAACTGG
AACGCAGACGATATCGAATGGGTAAACATCACCGACCAGATCTACCAGGGTGGTATCGTGAACTCTGGTGTGGGCGTTGGTTC
CGTTGTAGTTAAAGATAACTACATCTATTATATGTTCGGCGGCGAAGACCACTTCAACCCGTGGACTTACGGCGATAACTCCG
CGAAAGACCCGTTCAAATCCGATGGTCACCCTTCTGACCTCTATTGTTACAAAATGAAAATCGGTCCGGACAACCGTGTTTCC
CGCGATTTTCGCTACGGCGCTGTTCCAAACCGTGCAGTTCCGGTATTCTTCGACACGAACGGCGTGCGTACCGTTCCGGCTCC
GATGGAATTCACCGGCGACCTGGGTCTGGGCCACGTAACCATTCGTGCCTCCACCAGCTCTAACATCCGTTCCGAAGTACTCA
TGGAAGGTGAATACGGCTTTATCGGTAAGTCTATCCCGACGGACAACCCGGCAGGTCAGCGTATCATCTTCTGCGGCGGTGAG
GGTACCTCTAGCACCACCGGCGCGCAAATCACCCTGTACGGCGCTAACAACACCGACTCTCGTCGTATCGTATACAACGGTGA
TGAACATCTGTTCCAGTCCGCAGACGTGAAACCGTACAACGACAACGTCACCGCACTGGGTGGTCCATCCAACCGTTTCACCA
CTGCGTACCTGGGTTCCAACCCGATCGTTACTAGCAATGGTGAACGCAAAACTGAACCGGTAGTGTTTGACGACGCTTTTCTG
GACGCATGGGGCGATGTTCATTACATCATGTATCAGTGGCTGGATGCCGTGCAGCTGAAAGGTAACGACGCGCGTATCCACTT
TGGTGTGATCGCACAGCAGATTCGCGATGTCTTCATCGCACACGGTCTGATGGATGAAAATAGTACTAACTGTCGCTATGCGG
TGCTGTGCTATGACAAATACCCGCGTATGACCGACACCGTGTTCTCGCACAATGAGATTGTTGAACATACCGATGAAGAAGGT
AACGTGACTACTACCGAAGAACCGGTTTATACCGAAGTGGTTATTCACGAAGAAGGTGAAGAATGGGGCGTGCGTCCTGATGG
TATCTTTTTCGCGGAGGCAGCGTACCAGCGTCGCAAACTGGAACGCATCGAAGCTCGTCTGTCGGCACTGGAACAGAAATAA

Stf λ-KL106 chimera

ATGGCAGTAAAGATTTCAGGAGTCCTGAAAGACGGCACAGGAAAACCGGTACAGAACTGCACCATTCAGCTGAAAGCCAGACG
TAACAGCACCACGGTGGTGGTGAACACGGTGGGCTCAGAGAATCCGGATGAAGCCGGGCGTTACAGCATGGATGTGGAGTACG
GTCAGTACAGTGTCATCCTGCAGGTTGACGGTTTTCCACCATCGCACGCCGGGACCATCACCGTGTATGAAGATTCACAACCG
GGGACGCTGAATGATTTTCTCTGTGCCATGACGGAGGATGATGCCCGGCCGGAGGTGCTGCGTCGTCTTGAACTGATGGTGGA
AGAGGTGGCGCGTAACGCGTCCGTGGTGGCACAGAGTACGGCAGACGCGAAGAAATCAGCCGGCGATGCCAGTGCATCAGCTG
CTCAGGTCGCGGCCCTTGTGACTGATGCAACTGACTCAGCACGCGCCGCCAGCACGTCCGCCGGACAGGCTGCATCGTCAGCT
CAGGAAGCGTCCTCCGGCGCAGAAGCGGCATCAGCAAAGGCCACTGAAGCGGAAAAAAGTGCCGCAGCCGCAGAGTCCTCAAA
AAACGCGGCGGCCACCAGTGCCGGTGCGGCGAAAACGTCAGAAACGAATGCTGCAGCGTCACAACAATCAGCCGCCACGTCTG
CCTCCACCGCGGCCACGAAAGCGTCAGAGGCCGCCACTTCAGCACGAGATGCGGTGGCCTCAAAAGAGGCAGCAAAATCATCA
GAAACGAACGCATCATCAAGTGCCGGTCGTGCAGCTTCCTCGGCAACGGCGGCAGAAAATTCTGCCAGGGCGGCAAAAACGTC
CGAGACGAATGCCAGGTCATCTGAAACAGCAGCGGAACGGAGCGCCTCTGCCGCGGCAGACGCAAAAACAGCGGCGGCGGGGA
GTGCGTCAACGGCATCCACGAAGGCGACAGAGGCTGCGGGAAGTGCGGTATCAGCATCGCAGAGCAAAAGTGCGGCAGAAGCG

GCGGCAATACGTGCAAAAAATTCGGCAAAACGTGCAGAAGATATAGCTTCAGCTGTCGCGCTTGAGGATGCGGACACAACGAG
AAAGGGGATAGTGCAGCTCAGCAGTGCAACCAACAGCACGTCTGAAACGCTTGCTGCAACGCCAAAGGCGGTTAAGGTGGTAA
TGGATGAGACTAATCGTAAGGCACCTCTGGACAGTCCGGCACTGACCGGAACGCCAACAGCACCAACCGCGCTCAGGGGAACA
AACAATACCCAGATTGCGAACACCGCTTTTGTACTGGCCGCGATTGCAGATGTTATCGACGCGTCACCTGACGCACTGAATAC
GCTGAATGAACTGGCCGCAGCGCTCGGGAATGATCCAGATTTTGCTACCACCATGACTAACGCGCTTGCGGGTAAACAACCGA
AGAATGCGACACTGACGGCGCTGGCAGGGCTTTCCACGGCGAAAAATAAATTACCGTATTTTGCGGAAAATGATGCCGCCAGC
CTGACTGAACTGACTCAGGTTGGCAGGGATATTCTGGCAAAAAATTCCGTTGCAGATGTTCTTGAATACCTTGGGGCCGGTGA
GAATTCGGGATCAGCAGCTGATGTATTAATAGAATTAGCGAAACCAACAGGTGCCGATTTAGTTTATTGTGGAAATTCTCCTG
TATCTTTAATAATTAGAGGTAGTATATTTAAATACTTAAATGAAGTAGACAGGAGTACGCTGTTAAATGTAGTTGGGGCAGAA
GTTGTTGCGGATTATGCTCTACAAGCTGCTATCGATGATGGTGTTACCATTCTTGAATGGCATGCAGTTCCTGGGGTATATGT
TTTGGGTAAGGATTTAGTTACGTTACCTGTTGGTTTCTCATTTGAGGGAGAATCGAGGAGAACGTATACAGCATCATCAGATG
CTTCATTTAATAATGTTGGCACGGTTCTCCGTTTATTTAATGGTGCATCAGCAATTTTCAAAATGACATCAAGACACTCATTT
AGAAGGGTGGTTTTCGATGGTCGCAACAAGTCCGTGCGCTTTATGCAAGGGTATGACCAAACACAATGGTGCAGGTTTTATGA
CTGCGGAGTACACCGTTGGTATATTGGTATTGGTGGCTCAAGTCCTAATGGTTACTCTGCTACTTTAATCTTTTCTGGTGGCA
CCATTTCCAGTAATACGATTGGCGTCAAAAACGTCATTGATTCACTGTTTTTAGGGGCCACAATAAATGCCAATGACACTGAT
GGTGTTCAGCTTCTGACCGGAGCGAATAACAATGCGTTTATCGGCGTCAGAAACGAATGGAATAATGGTGATAACTATTATGG
CTATGGGTGTAAGAGAATACTGATTCAGGGTGAACTGATAGACAGGGCTGGCAAACGAGCGGTTGCCGCCGTTGGTGGAGCCC
AGTTTGTTCTTTCTGGTGTAGCTTTACAACGTTCGGGTAGACTTGCTACTGAGGGTACTGTAGATGACTCTCATTTTTATCTG
GAAGGGGATACGTCTTCAATAGTAGTAACCCCAACCTACACTACTACTGGTGCTAACGATGATGGGTCTGGTAGATCTTCCCC
AACCTATATATTAGCCACAGGTGGTAGCAACTCTGATGCGAAAAGTTTTATTGCTTCAGCATCCAATCTTTCAGGTTATACAG
GCACCTCATGGCTGCGTTCTGGTGTAATTGCCTCACTTTCAGTGCAAGGCTGCCTGGGAGTTGAGGATGTTAAAAACTTCGGA
CTTAGACGTATTAGTAACGGTGTACAGTATCTTGGCGATGCTGTGTCTGGGCTGGCTTTGTCTGGTGCAGGTAATACTGCAAC
TATGGTATTTACAACTACTCCACAAGAGCTTTCTCGATACTCGTCAGAGCTTCTTGTGCGTACTCTTGAAATTACGGCAAGAA
ATAATACCTCTACAGGGTCGGTTGCATATTACTCTGTTAACTTAATTATCTCACGTGAATATGCATCGGCTGCTATTGCGGTG
GATACGGCTTCTGTTAGAACTTTTGCCACTGTTAGTGGTGGAACGTGGGGCATTACCTCTGCTAGTCCAACCGGGGTTTCGCT
TTCTTTTGCTATCTCTTCTGATGGTAAGACCTTAACCGTAACATTAACAGCGATAGACTCTGCCAGCAGAGTTATCAGTGCAA
AACTAAGGGCATAA

List of plasmid sequences used in this study

p513

CCTTTAGGGAAATATGCTAAGTTTTCACCGTAACACGCCACATCTTGACTATATATGTGTAGAAACTGCCGGAAATCGTCGTG
GTATTCTGACCAGAGCGATGAAAACGTTTCAGTTTGCTCATGGAAAACGGTGTAACAAGGGTGAACACTATCCCATATCACCA
GCTCACCGTCTTTCATTGCCATACGAAACTCCGGATGTGCATTCATCAGGCGGGCAAGAATGTGAATAAAGGCCGGATAAAAC
TTGTGCTTATTTTTCTTTACGGTTTTTAAAAAGGCCGTAATATCCAGCTGAACGGTTTGGTTATAGGTGCACTGAGCAACTGA
CTGGAATGCCTCAAAATGTTCTTTACGATGCCATTGACTTATATCAACTGTAGTATATCCAGTGATTTTTTTCTCCATTTTAG
CTTCCTTAGCTTGCGAAATCTCGATAACTCAAAAAATAGTAGTGATCTTATTTCATTATGGTGAAAGTTGTCTTACGTGCAAC
ATTTTCGCAAAAAGTTGGCGCTTTATCAACACTGTCCCTCCTGTTCAGCTACTGACGGTACTGCGGAACTGACTAAAGTAGTG
CGTAACGGCAAAAGCACCGCCGGACATCTGCGCTAGCGGAGTGTATACTGGCTTACTATGTTGGCACTGATGAGGGTGTAAGT
GAAGTGCTTCATGTGGCAGGAGAAAAAAGGCTGCATCGGTGCGTCAGCAGAATATGTGATACAGGATATATTCCGCTTCCTCG
CTCACTGACTCGCTACGCTCGGTCGTTCGACTGTGGCGAGCGGAAATGGCTTACGAACGGGGCGGAGATTTCCTGGAAGATGC
CAGGAAGATACTTAACAGGGAAGTGAGAGGGTCGCGGCAAAGCCGTTTTTCCATAGGCTCCGCCCCCCTGACAAGCATCACGA
AATCTGACGCTCAAATCAGTGGTGGCGAAACCTGACAGGACTATAAAGATACCAGGCGTTTCCCCCTGGCGGCTCCCTCGTGC
GCTCTCCTGTTCCTGCCTTTCGGTTTGCCGGTGTCATTCCTCTGTTACGGCCGAGTTTGTCTCATTCCACGCCTGACACTCAG
TTCCGGGTAGGCAGTTCGCTCCAAGCTGGACTGTATGCACGAACCCCCCGTTCAGTCCGACCGCTGCGCCTTATCCGGTAACT
ATCGTCTTGAGTCCAACCCGGAAAGACATGCAAAAGCACCACTGGCAGCAGCCACTGGTAATTGATTTAGAGGAGTTAGTCTT
GAAGTCATGCGCCGGATAAGGCTAAACTGAAAGGACAAGTTTTGGCGACTGCGCTCCTCCAAGCCAGTTACCTCGGTTCAAAG
AGTTGGTAGCTCAGAGAACCTTCGAAAAACCGCCCTGCAAGGCGGTTTTTTCGTTTTCAGAGCAAGAGATTACGCGCAGACCA
AAACGATCTCAAGAAGATCATCTTATTAATCAGATAAAATATTTCTAGATTTCAGTGCAATTTATCTCTTCAAATGTAGCACT
TTATAGCTAGCTCAGCCCTTGGTACAATGCTAGCGTTTTCATTAAAGAGGAGAAAGGAAGCCATGAGTAAAGGTGAGGAATTA
TTTACTGGTGTTGTTCCGATCTTAGTTGAACTGGACGGCGATGTTAACGGTCATAAATTCAGTGTTCGTGGTGAAGGTGAAGG
TGATGCAACCAACGGTAAGCTGACCCTGAAATTCATCTGCACTACTGGAAAATTACCAGTACCGTGGCCTACTCTGGTGACTA
CCCTGACCTATGGTGTTCAGTGTTTTTCTCGTTACCCTGACCACATGAAGCAACATGATTTCTTCAAATCTGCAATGCCGGAA
GGTTATGTACAGGAGCGCACCATTTCTTTCAAAGACGATGGCACGTATAAAACCCGTGCAGAGGTTAAATTTGAAGGTGACAC
TCTGGTGAATCGTATTGAACTGAAAGGCATTGATTTCAAAGAGGACGGCAATATTTTAGGCCACAAACTGGAATATAACTTCA
ACTCCCATAACGTTTACATCACCGCAGACAAACAAAAGAACGGTATCAAAGCTAACTTCAAAATTCGCCATAACGTTGAAGAC
GGTAGCGTACAGCTGGCGGATCATTACCAACAGAACACTCCGATTGGAGATGCTCCTGTTTTACTGCCGGATAACCACTACCT

GTCCACCCAGTCTAAACTGTCGAAGGATCCGAACGAAAAGCGCGACCACATGGTGTTATTAGAGTTCGTTACCGCTAGTGGTA
TCACGCACGGTATGGATGAACTCTACAAATAAGTCAGTTTCACCTGTTTTACGTTAAAACCCGCTTCGGCGGGTTTTTACTTT
TGGGTTTAGCCGAACGCCCCAAAAAGCCTCGCTTTCAGCACCTGTCGTTTCCTTTCTTTTCAGAGGGTATTTTAAATAAAAAC
ATTAAGTTATGACGAAGAAGAACGGAAACGCCTTAAACCGGAAAATTTTCATAAATAGCGAAAACCCGCGAGGTCGCCGCCCC
GTAACCTGTCGGATCACCGGAAAGAACCTGTAAAGTGATAATGATTATCATCTACATATCACAACGTGCGTAAAGGGACTATA
ACAAGACGCAAACGGAGGTAGGCTCACTCCTACTTCGGAAACTTAACCGAAGAACTAGGACGGTATTGTTTGCGCTTGGAATT
GGCCTTGAAGTAAGTCAGGTTTTGACGGAACGATTAGTTACAGGGGGGGAACAGTCGTTGGTCGCCACCAAGTCGATTTTTGG
CTTACCTCTTATCTCGTAGTTGGTGAGGGTTGGGATTCACGGACGAGATCCAGCCTAAGTATATTGTCACTTCTGATTCGTT
CGATCACTTACTCCCCTTACTTATCCTGCGGCTACTGTTTCCGCTGGCTCGTAAGCTCTACGTTCGGCAATCTACCCGCGAGG
TCAGACGTGACACTCTTAAACTAAAAATTGGTAGCTTCTTTGGCTGAATTGCTGGATCTTATTCGTTCACCCAATAAAACGGT
ACAGCTTCAAGCAATATCCTCAGTAAGTTAATACCCGTTGTACTATTACTTTCACGACCGTTCGACGTTCCCGCTCTATTTAT
TAAGAGCTGTCACTTCGAGTCTTTAGCTCACTTAGGAATTAGCTGAGTTTAGGCTCAGCCCTCTTGGGTTGCTTGTACTTTCA
GAGTTATTCGCACGGCTGGTTTTGTCGAGTGGGGAATTGTGGTTGACCGAAAGTCCGCTATCCTTCAACGCCGAATCAGCTCT
TGCCCTTTACTATCTTCAATCTCTTGGAGGCTATTACGGGCGGGGGCAAGAGATTAGAACTGCAAGACACCCGTTGATAATCG
AGTCGCTCGATAGATTGTCGAGAGCCGGAGAGATTAGTACGTTATTCAAGGCAATACGTGCAGGGTTAATCTGGGCGCGTTGT
AGTCTACGCTGGCGTAAGTCCCCAATAACACGCTCGTCCGGCGAGTCACGATCCTCTAGGCGGTGTTCAACGCGTACGCCAGC
TATTTGGGATACTTAGCTACGTTACACGTAAGAATATCTTAGCGGAGGATCGCCCTGCTTCCGCTTGGACGGATAAACGGGAG
AGTGGGCGCGTATAGCGCAGGCGGTGTGAAGGCTTTTAAGTAATTCTAGCCCTCTTTGAACGGTATTTCCCAATTTGGAGATT
ACCGGATAGCGCGTTTAAATGAGTGTCAGAGAAACGGAAGCCGAAGTCTTTCCATTCCGGATGTTTGGAAATGCTCTGTTTAT
AGAAGTCGATGAACTTACGGCAGTCCTCTATATTAAATTCGAATTTTTCATACCCTTTCTGCGGGCTACCGTTTTTAGTGTGC
GTGCTATGATTGCGGATGCGCAGAATATCCTCAGACGGGTTGTAGAATTTGATGCTTTTCGCGGAAAAGAACACTTTCGGTAA
CATTTTATTCGCACCCGGCAGGAGCTTGTACACGATTTTCTTATAGCCTTCACCCTTGTTTTCTTTGATCGCTTTATCGTCGA
AGATCTTGTTGTTCTTCTTGTTCATTACGCCCAGATAGTATTTGTCGTCTTTGATGAACAGGATTGCGGTGTTGTCCGGCTCT
TTGTTCTTATCCCAGCCGTTCGCCAGCGTGCTGTTTTCGAAGTTCAGTTTGAATTTCTCGTCAGAGTAAGGCTTCTGCGTGAT
GTAGTTGCGGATTTTATTGTAGAGAGGGACGATGTTTGCCAGTTCGAAGTAACATTCTTCGAACACCAGATAGAAGTGTTCAT
CTTTATCCAGAATGTTCGCCTTGTCCTCGCTCTGGCTGATGTGGAAGATTTTGAGCTTGTGTAATAAGTTATTCGTCTGATCT
AATAAGTCTTTAATTGCTTTCACATCGTCCTCCGCAGATGCTTGAAGCAGATCTTTCTTACCCTGATTCTGGTACTTGATAGA
GATCTGCGCCAGATTGTCTTTGTTTTGAGCAATTTCGTCGAAGATCATCGGGATTGCCGCAAAGTTCGCCAGAATTTCCTCAA
AACGACACTGTTTATCAATATCACGATGTTTATTAAATTCCTCAAGTGCCAGTTTGATAGTTTCTAAGCTCAGGTATTTAGCT
TTTTCTGTTTTCTTTGCAATCAGTTCCTGTTCCTTCTTGGACGGGTTGTCCAGATTTTTCGGCGCGATTTGTTGGGTGATGTA
TTCCAAAACTGCCGTGCCGATCACGCTATAGTCATCGAAAACTTGTTGACTGAGATCGGTCAGAGATTTGTCGTTTTTAAAGT
AAATCTTAGACAGATCTAGTTTCTGCGCTTTGAGGTCGTCAAAGAGCAGGGACAGAGTTTCTTTAATAGATTTCTCTTCCACG
GTTTTGAACGCCGCAATCTGCTCATAAAAGCTCTGCATCGTGGTGACAACGTCGCTATCATCTTCCAGTTTATCAATTACGAA
GGATTTAGATTCGGTGTCCGATAAAATCTGTTTAAACAGAACGGACATTTTATACTTTTTCAGGGTTTTGTCGTTGATTTGTT
GGCTATACAGGTTAATGTATTCGTTGATGCCCTTACGCTTGGTGTTTTCGCCGTTAACAAATTTGCCACCAATAATGGTGTTG
AATTTGGTGATGCCAGATTGATTCAGGTAATTGTTGAAATTAGCGATTTCGAAAACCTCGTCCAGTGAGAAAACACGCTGGTT
AACTTCGGAGGTTTTATAGTCGATGTCGAAGGTCAGTTCTTCCGCCAGATCTTTCTTGATCTGTTCATAGTTAATAGCTTCCG
GTGCTTTGTCTTTCAGAGATTCATATTTCGCTTTGTTTTCCAGAAACTTCGGCAGGTTGTCGTCCACGATACGATAAATAATA
GAGGTCGGAATATCGTTGCTCGAATATACATTCTTACGGTTTTCATGAAAACCTTTGAAATACGTCGTCCAGCCTTTGAAAGA
CTTGATGATTTCGGTATTCGAATATGACCTGATCAAAGATAAACGTTTCACCGAAGATAAGTTCTTTTTCCACTGTCCGATTA
CCATCAACTTCAAATCTAGCGGTGCGAACAAGTTCAACGATGAAATTAACTTATTACTGAAAGAGAAAGCTAATGACGTACAC
ATCTTATCTATTGATCGCGGTGAACGTCATTTAGCATACTATACACTGGTAGATGGTAAAGGTAATATTATTAAACAGGATAC
TTTCAATATTATCGGTAATGACCGTATGAAAACCAACTATCACGATAAGCTGGCGGCGATCGAAAAAGATCGTGATTCTGCGC
GTAAAGATTGGAAGAAAATTAACAATATCAAAGAAATGAAAGAAGGCTATCTGAGCCAAGTGGTGCACGAGATCGCAAAACTG
GTGATTGAATATAACGCTATCGTGGTTTTCGAAGATCTGAACTTTGGTTTTAAACGTGGTCGCTTCAAAGTAGAAAAACAGGT
GTACCAAAAACTGGAAAAAATGCTGATTGAAAAACTGAACTATCTGGTTTTTAAAGACAACGAATTTGACAAAACGGGTGGCG
TACTCCGTGCCTATCAGCTGACCGCTCCGTTCGAAACGTTCAAGAAAATGGGTAAACAAACGGGGATTATCTATTATGTGCCA
GCTGGTTTCACCTCCAAGATTTGTCCAGTTACGGGCTTCGTTAACCAGCTGTACCCGAAATACGAGAGCGTTAGCAAATCTCA
AGAATTTTTCAGCAAATTCGACAAGATCTGCTATAATCTGGATAAAGGCTATTTCGAGTTCAGCTTCGATTACAAAAACTTCG
GCGATAAAGCGGCTAAAGGTAAGTGGACTATTGCTAGCTTTGGTAGCCGTCTGATTAACTTTCGCAACTCCGACAAAAACCAT
AATTGGGACACGCGTGAAGTGTATCCGACCAAAGAACTGGAAAAATTACTGAAAGACTATTCCGGACACTCAGAAGGGTTATA
GGAATAGTCACTACTGGGGTAAGCACTTCGGAAATTATATTATTCTCGCTTCTTATTGCGGTAACGTGATCCTGAACGATACT
TATTACTTTGTAATTTACTTAACGTCGGAGTCCCTGCAATCTTCTAGTACCCGCTTCCCGAATACAGGAGATAACTTTTTAAC
ACTCAAGAGTTGCTTCGTGCTTAGCCAGTCTTGGATTTGATTGCTCTAATCCTTCAACGTGTCAAAGACAGTGTATCTGGTCA
AGTAAAGTCTAGAGAAAGGCGTAGTCAGTTACGGAGTTATCCCACCTTAGTGTTACTCCGATTTAATTTCTGCTTTCTTTGAT
TTCTACCCGACTTTCGCCGTGACTTCAATAGAGAGGCAGGCTCTTGCTATTTCTTTCAAGGGCTTGTCCAACTACCTAATTAA
GATAAAGATACGGCAGTTGACGCACTGCCGATAATTTCTTTACGTCAGCGAAATTAAATCGAGCACCAGTCGTAGAGTCGCGG
TTGCCTAGCAGTTTATCTCGCGTACGGCCTTCGCTACTTACACGATACCTAGTACGTGGATTCGGGTAGCACCAGAAGTCTA
TAGCATGTGCATACCTTTGGTCGAAAAAAAAAGCCCGCACTGTCAGGTGCGGGCTTTTTTCAGTGTTTCCTTGCCGGATTACG
CCCCGCCCTGCCACTCATCGCAGTATTGTTGTAATTCATTAAGCATTCTGCCGACATGGAAGCCATCACAAACGGCATGATGA
ACTTGGATCGCCAGTGGCATTAACACCTTGTCGCCTTGCGTATAATATTTTCCCATAGTGAAAACGGGGGCGAAGAAGTTGTC
CATATTTGCTACGTTTAAATCAAAACTGGTGAAACTCACCCAGGGATTGGCACTGACGAAAAACATATTTCGATAAAC

p938

GGACAGTTTTCCCTTTGATATGTAACGGTGAACAGTTGTTCTACTTTTGTTTGTTAGTCTTGATGCTTCACTGATAGATACAA
GAGCCATAAGAACCTCAGATCCTTCCGTATTTAGCCAGTATGTTCTCTAGTGTGGTTCGTTGTTTTTGCGTGAGCCATGAGAA
CGAACCATTGAGATCATACTTACTTTGCATGTCACTCAAAAATTTTGCCTCAAAACTGGTGAGCTGAATTTTTGCAGTTAAAG
CATCGTGTAGTGTTTTTCTTAGTCCGTTACGTAGGTAGGAATCTGATGTAATGGTTGTTGGTATTTTGTCACCATTCATTTTT
ATCTGGTTGTTCTCAAGTTCGGTTACGAGATCCATTTGTCTATCTAGTTCAACTTGGAAAATCAACGTATCAGTCGGGCGGCC
TCGCTTATCAACCACCAATTTCATATTGCTGTAAGTGTTTAAATCTTTACTTATTGGTTTCAAAACCCATTGGTTAAGCCTTT
TAAACTCATGGTAGTTATTTTCAAGCATTAACATGAACTTAAATTCATCAAGGCTAATCTCTATATTTGCCTTGTGAGTTTTC
TTTTGTGTTAGTTCTTTTAATAACCACTCATAAATCCTCATAGAGTATTTGTTTTCAAAAGACTTAACATGTTCCAGATTATA
TTTTATGAATTTTTTTAACTGGAAAAGATAAGGCAATATCTCTTCACTAAAAACTAATTCTAATTTTTCGCTTGAGAACTTGG
CATAGTTTGTCCACTGGAAAATCTCAAAGCCTTTAACCAAAGGATTCCTGATTTCCACAGTTCTCGTCATCAGCTCTCTGGTT
GCTTTAGCTAATACACCATAAGCATTTTCCCTACTGATGTTCATCATCTGAGCGTATTGGTTATAAGTGAACGATACCGTCCG
TTCTTTCCTTGTAGGGTTTTCAATCGTGGGGTTGAGTAGTGCCACACAGCATAAAATTAGCTTGGTTTCATGCTCCGTTAAGT
CATAGCGACTAATCGCTAGTTCATTTGCTTTGAAAACAACTAATTCAGACATACATCTCAATTGGTCTAGGTGATTTTAATCA
CTATACCAATTGAGATGGGCTAGTCAATGATAATTACTAGTCCTTTTCCTTTGAGTTGTGGGTATCTGTAAATTCTGCTAGAC
CTTTGCTGGAAAACTTGTAAATTCTGCTAGACCCTCTGTAAATTCCGCTAGACCTTTGTGTGTTTTTTTTGTTTATATTCAAG
TGGTTATAATTTATAGAATAAAGAAAGAATAAAAAAAGATAAAAAGAATAGATCCCAGCCCTGTGTATAACTCACTACTTTAG
TCAGTTCCGCAGTATTACAAAAGGATGTCGCAAACGCTGTTTGCTCCTCTACAAAACAGACCTTAAAACCCTAAAGGCTTAAG
TAGCACCCTCGCAAGCTCGGTTGCGGCCGCAATCGGGCAAATCGCTGAATATTCCTTTTGTCTCCGACCATCAGGCACCTGAG
TCGCTGTCTTTTTCGTGACATTCAGTTCGCTGCGCTCACGGCTCTGGCAGTGAATGGGGGTAAATGGCACTACAGGCGCCTTT
TATGGATTCATGCAAGGAAACTACCCATAATACAAGAAAAGCCCGTCACGGGCTTCTCAGGGCGTTTTATGGCGGGTCTGCTA
TGTGGTGCTATCTGACTTTTTGCTGTTCAGCAGTTCCTGCCCTCTGATTTTCCAGTCTGACCACTTCGGATTATCCCGTGACA
GGTCATTCAGACTGGCTAATGCACCCAGTAAGGCAGCGGTATCATCAACGGGGTCTGACGCTCAGTGGAACGAAAACTCACGT
TAAGGGATTTTGGTCATGAGATTATCAAAAAGGATCTTCACCTAGATCCTTTTAAATTAAAAATGAAGTTTTAAATCAATCTA
AAGTATATATGAGTAAACTTGGTCTGACAGTTACGTTTCCACAACCAATTAACCAATTCTGATTTAGAAAAACTCATCGAGCA
TCAAATGAAACTGCAATTTATTCATATCAGGATTATCAATACCATATTTTTGAAAAAGCCGTTTCTGTAATGAAGGAGAAAAC
TCACCGAGGCAGTTCCATAGGATGGCAAGATCCTGGTATCGGTCTGCGATTCCGACTCGTCCAACATCAATACAACCTATTAA
TTTCCCCTCGTCAAAAATAAGGTTATCAAGTGAGAAATCACCATGAGTGACGACTGAATCCGGTGAGAATGGCAAAAGCTTAT
GCATTTCTTTCCAGACTTGTTCAACAGGCCAGCCATTACGCTCGTCATCAAAATCACTCGCATCAACCAAACCGTTATTCATT
CGTGATTGCGCCTGAGCGAGACGAAATACGCGATCGCTGTTAAAAGGACAATTACAAACAGGAATCGAATGCAACCGGCGCAG
GAACACTGCCAGCGCATCAACAATATTTTCACCTGAATCAGGATATTCTTCTAATACCTGGAATGCTGTTTTCCCGGGGATCG
CAGTGGTGAGTAACCATGCATCATCAGGAGTACGGATAAAATGCTTGATGGTCGGAAGAGGCATAAATTCCGTCAGCCAGTTT
AGTCTGACCATCTCATCTGTAACATCATTGGCAACGCTACCTTTGCCATGTTTCAGAAACAACTCTGGCGCATCGGGCTTCCC
ATACAATCGATAGATTGTCGCACCTGATTGCCCGACATTATCGCGAGCCCATTTATACCCATATAAATCAGCATCCATGTTGG
AATTTAATCGCGGCCTCGAGCAAGACGTTTCCCGTTGAATATGGCTCATAACACCCCTTGTATTACTGTTTATGTAAGCAGAC
AGTTTTATTGTTCATGATGATATATTTTTATCTTGTGCAATGTAACATCAGAGATTTTGAGACACAACGTGGCTTTCCCTGCA
GGATTTCGGAGGCCTGCGTTATCCCCTGATTCTGTGGATAACCGTATTACCGCCTTTGAGTGAGCTGATACCGCTCGCCGCAG
CCGAACGCCGACTAGTGGATTTTACGGCTAGCTCAGTCCTAGGTACAATGCTAGCGAATTCATTAAAGAGGAGAAAGGTACCC
ATGGCACGTACCCCGAGCCGTAGCAGCATTGGTAGCCTGCGTAGTCCGCATACCCATAAAGCAATTCTGACCAGCACCATTGA
AATCCTGAAAGAATGTGGTTATAGCGGTCTGAGCATTGAAAGCGTTGCACGTCGTGCCGGTGCAAGCAAACCGACCATTTATC
GTTGGTGGACCAATAAAGCAGCACTGATTGCCGAAGTGTATGAAAATGAAAGCGAACAGGTGCGTAAATTTCCGGATCTGGGT
AGCTTTAAAGCCGATCTGGATTTTCTGCTGCGTAATCTGTGGAAAGTTTGGCGTGAAACCATTTGTGGTGAAGCATTTCGTTG
TGTTATTGCAGAAGCACAGCTGGACCCTGCAACCCTGACCCAGCTGAAAGATCAGTTTATGGAACGTCGTCGTGAGATGCCGA
AAAAACTGGTTGAAAATGCCATTAGCAATGGTGAACTGCCGAAAGATACCAATCGTGAACTGCTGCTGGATATGATTTTTGGT
TTTTGTTGGTATCGCCTGCTGACCGAACAGCTGACCGTTGAACAGGATATTGAAGAATTTACCTTCCTGCTAATTAATGGTGT
TTGTCCGGGTACACAGCGTTAACTAGGGCCCATACCCCCAATTATTGAAGGCCGCTAACGCGGCCTTTTTTTGTTTCTGGTCT
GCCCGACGTACGGTGAATCTGATTCGTTACCAATTGACATGATACGAAACGTACCGTATCGTTAAGGTTACTAGATTAAAGAG
GAGAAATACTAGATGGCAGTAAAGATTTCAGGAGTCCTGAAAGACGGCACAGGAAAACCGGTACAGAACTGCACCATTCAGCT
GAAAGCCAGACGTAACAGCACCACGGTGGTGGTGAACACGGTGGGCTCAGAGAATCCGGATGAAGCCGGGCGTTACAGCATGG
ATGTGGAGTACGGTCAGTACAGTGTCATCCTGCAGGTTGACGGTTTTCCACCATCGCACGCCGGGACCATCACCGTGTATGAA
GATTCACAACCGGGGACGCTGAATGATTTTCTCTGTGCCATGACGGAGGATGATGCCCGGCCGGAGGTGCTGCGTCGTCTTGA
ACTGATGGTGGAAGAGGTGGCGCGTAACGCGTCCGTGGTGGCACAGAGTACGGCAGACGCGAAGAAATCAGCCGGCGATGCCA
GTGCATCAGCTGCTCAGGTCGCGGCCCTTGTGACTGATGCAACTGACTCAGCACGCGCCGCCAGCACGTCCGCCGGACAGGCT
GCATCGTCAGCTCAGGAAGCGTCCTCCGGCGCAGAAGCGGCATCAGCAAAGGCCACTGAAGCGGAAAAAAGTGCCGCAGCCGC
AGAGTCCTCAAAAAACGCGGCGGCCACCAGTGCCGGTGCGGCGAAAACGTCAGAAACGAATGCTGCAGCGTCACAACAATCAG
CCGCCACGTCTGCCTCCACCGCGGCCACGAAAGCGTCAGAGGCCGCCACTTCAGCACGAGATGCGGTGGCCTCAAAAGAGGCA
GCAAAATCATCAGAAACGAACGCATCATCAAGTGCCGGTCGTGCAGCTTCCTCGGCAACGGCGGCAGAAAATTCTGCCAGGGC
GGCAAAAACGTCCGAGACGAATGCCAGGTCATCTGAAACAGCAGCGGAACGGAGCGCCTCTGCCGCGGCAGACGCAAAAACAG

CGGCGGCGGGGAGTGCGTCAACGGCATCCACGAAGGCGACAGAGGCTGCGGGAAGTGCGGTATCAGCATCGCAGAGCAAAAGT
GCGGCAGAAGCGGCGGCAATACGTGCAAAAAATTCGGCAAAACGTGCAGAAGATATAGCTTCAGCTGTCGCGCTTGAGGATGC
GGACACAACGAGAAAGGGGATAGTGCAGCTCAGCAGTGCAACCAACAGCACGTCTGAAACGCTTGCTGCAACGCCAAAGGCGG
TTAAGGTGGTAATGGATGAGACTAATCGTAAAGCGCCATTAAACAGCCCTGCACTGACCGGAACGCCAACGACGCCAACTGCG
CGACAGGGAACGAATAATACTCAGATCGCAAACACGGCTTTCGTTATGGCCGCGATTGCCGCCCTTGTAGACTCGTCGCCTGA
CGCACTGAATACGCTGAACGAGCTGGCGGCGGCGCTGGGCAATGACCCGAATTTTGCTACCACCATGACTAATGCGCTTGCGG
GTAAGCAACCGAAAGATGCTACCCTGACGGCGCTGGCGGGGCTTGCTACTGCGGCAGACAGGTTTCCGTATTTTACGGGGAAT
GATGTTGCCAGCCTGGCGACCCTGACAAAAGTCGGGCGGGATATTCTGGCTAAATCGACCGTTGCCGCCGTTATCGAATATCT
CGGTTTACAGGAAACGGTAAACCGAGCCGGGAACGCCGTGCAAAAAAATGGCGATACCTTGTCCGGTGGACTTACTTTTGAAA
ACGACTCAATCCTTGCCTGGATTCGAAATACTGACTGGGCGAAGATTGGATTTAAAAATGATGCCGATGGTGACACTGATTCA
TACATGTGGTTTGAAACGGGGGATAACGGCAATGAATATTTCAAATGGAGAAGCCGCCAGAGTACCACAACAAAAGACCTGAT
GACGTTGAAATGGGATGCACTAAATATTCTTGTTAATGCCGTCATTAATGGCTGTTTTGGAGTTGGTACGACGAATGCACTAG
GTGGTAGCTCTATTGTTCTTGGTGATAATGATACCGGATTTAAACAGAATGGAGACGGTATTCTTGATGTTTATGCTAACAGT
CAGCGTGTATTCCGTTTTCAGAATGGAGTGGCTATTGCTTTTAAAAATATTCAGGCAGGTGATAGTAAAAAGTTCTCGCTATC
CAGCTCTAATACATCCACGAAGAATATTACCTTTAATTTATGGGGTGCTTCCACCCGTCCAGTGGTTGCAGAGTTAGGCGATG
AGGCCGGATGGCATTTCTATAGCCAGCGAAATACAGATAACTCGGTAATATTTGCTGTTAACGGTCAGATGCAACCCAGCAAC
TGGGGAAATTTTGATTCCCGCTATGTGAAAGATGTTCGCCTGGGTACGCGAGTTGTTCAATTGATGGCGCGAGGTGGTCGTTA
TGAAAAAGCCGGACACACGATTACCGGATTAAGAATCATTGGTGAAGTAGATGGCGATGATGAAGCCATCTTCAGGCCGATAC
AAAAATACATCAATGGCACATGGTATAACGTTGCGCAGGTGTAAGTTATGCAGCATTTAAAGAACATTAAGTCAGGTAATCCA
AAAACAAAAGAGCAATATCAGCTAACAAAGAATTTTGATGTTATCTGGTTATGGTCCGAAGACGGAAAAAACTGGTATGAGGA
AGTGAAGAACTTTCAGCCAGACACAATAAAGATTGTTTACGATGAAAATAATATTATTGTCGCTATCACCAGAGATGCTTCAA
CGCTTAATCCTGAAGGTTTTAGCGTTGTTGAGGTTCCTGATATTACCTCCAACCGACGTGCTGACGACTCAGGTAAATGGATG
TTTAAGGATGGTGCTGTGGTTAAACGGATTTATACGGCAGATGAACAGCAACAACAGGCAGAATCACAAAAGGCCGCGTTACT
TTCCGAAGCGGAAAACGTTATTCAGCCACTGGAACGCGCTGTCAGGCTGAATATGGCGACGGATGAGGAACGTGCACGACTGG
AGTCATGGGAACGTTACAGCGTTCTGGTCAGCCGTGTGGATCCTGCAAATCCTGAATGGCCGGAAATGCCGCAATAAAACTAT
ATTGTGAGGCTTGCATAATGGCATTCAGAATGAGTGAACAACCACGGACCATAAAAATTTATAATCTGCTGGCCGGAACTAAT
GAATTTATTGGTGAAGGTGACGCATATATTCCGCCTCATACCGGTCTGCCTGCAAACAGTACCGATATTGCACCGCCAGATAT
TCCGGCTGGCTTTGTGGCTGTTTTCAACAGTGATGAGGCATCGTGGCATCTCGTTGAAGACCATCGGGGTAAAACCGTCTATG
ACGTGGCTTCCGGCGACGCGTTATTTATTTCTGAACTCGGTCCGTTACCGGAAAATTTTACCTGGTTATCGCCGGGAGGGGAA
TATCAGAAGTGGAACGGCACAGCCTGGGTGAAGGATACGGAAGCAGAAAAACTGTTCCGGATCCGGGAGGCGGAAGAAACAAA
AAAAAGCCTGATGCAGGTAGCCAGTGAGCATATTGCGCCGCTTCAGGATGCTGCAGATCTGGAAATTGCAACGAAGGAAGAAA
CCTCGTTGCTGGAAGCCTGGAAGAAGTATCGGGTGTTGCTGAACCGTGTTGATACATCAACTGCACCTGATATTGAGTGGCCT
GCTGTCCCTGTTATGGAGTAATGACGCATCCTCACGATAATATCCGGGTAGGACGAACAATAAGGCCGCAAATCGCGGCCTTT
TTTATTGATAACAAAA

p1321

GGACGAACAATAAGGCCGCAAATCGCGGCCTTTTTTATTGATAACAAAAGGACAGTTTTCCCTTTGATATGTAACGGTGAACA
GTTGTTCTACTTTTGTTTGTTAGTCTTGATGCTTCACTGATAGATACAAGAGCCATAAGAACCTCAGATCCTTCCGTATTTAG
CCAGTATGTTCTCTAGTGTGGTTCGTTGTTTTTGCGTGAGCCATGAGAACGAACCATTGAGATCATACTTACTTTGCATGTCA
CTCAAAAATTTTGCCTCAAAACTGGTGAGCTGAATTTTTGCAGTTAAAGCATCGTGTAGTGTTTTTCTTAGTCCGTTACGTAG
GTAGGAATCTGATGTAATGGTTGTTGGTATTTTGTCACCATTCATTTTTATCTGGTTGTTCTCAAGTTCGGTTACGAGATCCA
TTTGTCTATCTAGTTCAACTTGGAAAATCAACGTATCAGTCGGGCGGCCTCGCTTATCAACCACCAATTTCATATTGCTGTAA
GTGTTTAAATCTTTACTTATTGGTTTCAAAACCCATTGGTTAAGCCTTTTAAACTCATGGTAGTTATTTTCAAGCATTAACAT
GAACTTAAATTCATCAAGGCTAATCTCTATATTTGCCTTGTGAGTTTTCTTTTGTGTTAGTTCTTTTAATAACCACTCATAAA
TCCTCATAGAGTATTTGTTTTCAAAAGACTTAACATGTTCCAGATTATATTTTATGAATTTTTTTAACTGGAAAAGATAAGGC
AATATCTCTTCACTAAAAACTAATTCTAATTTTTCGCTTGAGAACTTGGCATAGTTTGTCCACTGGAAAATCTCAAAGCCTTT
AACCAAAGGATTCCTGATTTCCACAGTTCTCGTCATCAGCTCTCTGGTTGCTTTAGCTAATACACCATAAGCATTTTCCCTAC
TGATGTTCATCATCTGAGCGTATTGGTTATAAGTGAACGATACCGTCCGTTCTTTCCTTGTAGGGTTTTCAATCGTGGGGTTG
AGTAGTGCCACACAGCATAAAATTAGCTTGGTTTCATGCTCCGTTAAGTCATAGCGACTAATCGCTAGTTCATTTGCTTTGAA
AACAACTAATTCAGACATACATCTCAATTGGTCTAGGTGATTTTAATCACTATACCAATTGAGATGGGCTAGTCAATGATAAT
TACTAGTCCTTTTCCTTTGAGTTGTGGGTATCTGTAAATTCTGCTAGACCTTTGCTGGAAAACTTGTAAATTCTGCTAGACCC
TCTGTAAATTCCGCTAGACCTTTGTGTGTTTTTTTTGTTTATATTCAAGTGGTTATAATTTATAGAATAAAGAAAGAATAAAA
AAAGATAAAAAGAATAGATCCCAGCCCTGTGTATAACTCACTACTTTAGTCAGTTCCGCAGTATTACAAAAGGATGTCGCAAA
CGCTGTTTGCTCCTCTACAAAACAGACCTTAAAACCCTAAAGGCTTAAGTAGCACCCTCGCAAGCTCGGTTGCGGCCGCAATC
GGGCAAATCGCTGAATATTCCTTTTGTCTCCGACCATCAGGCACCTGAGTCGCTGTCTTTTTCGTGACATTCAGTTCGCTGCG
CTCACGGCTCTGGCAGTGAATGGGGGTAAATGGCACTACAGGCGCCTTTTATGGATTCATGCAAGGAAACTACCCATAATACA
AGAAAAGCCCGTCACGGGCTTCTCAGGGCGTTTTATGGCGGGTCTGCTATGTGGTGCTATCTGACTTTTTGCTGTTCAGCAGT
TCCTGCCCTCTGATTTTCCAGTCTGACCACTTCGGATTATCCCGTGACAGGTCATTCAGACTGGCTAATGCACCCAGTAAGGC
AGCGGTATCATCAACGGGGTCTGACGCTCAGTGGAACGAAAACTCACGTTAAGGGATTTTGGTCATGAGATTATCAAAAAGGA
TCTTCACCTAGATCCTTTTAAATTAAAAATGAAGTTTTAAATCAATCTAAAGTATATATGAGTAAACTTGGTCTGACAGTTAC
GTTTCCACAACCAATTAACCAATTCTGATTTAGAAAAACTCATCGAGCATCAAATGAAACTGCAATTTATTCATATCAGGATT

ATCAATACCATATTTTTGAAAAAGCCGTTTCTGTAATGAAGGAGAAAACTCACCGAGGCAGTTCCATAGGATGGCAAGATCCT
GGTATCGGTCTGCGATTCCGACTCGTCCAACATCAATACAACCTATTAATTTCCCCTCGTCAAAAATAAGGTTATCAAGTGAG
AAATCACCATGAGTGACGACTGAATCCGGTGAGAATGGCAAAAGCTTATGCATTTCTTTCCAGACTTGTTCAACAGGCCAGCC
ATTACGCTCGTCATCAAAATCACTCGCATCAACCAAACCGTTATTCATTCGTGATTGCGCCTGAGCGAGACGAAATACGCGAT
CGCTGTTAAAAGGACAATTACAAACAGGAATCGAATGCAACCGGCGCAGGAACACTGCCAGCGCATCAACAATATTTTCACCT
GAATCAGGATATTCTTCTAATACCTGGAATGCTGTTTTCCCGGGGATCGCAGTGGTGAGTAACCATGCATCATCAGGAGTACG
GATAAAATGCTTGATGGTCGGAAGAGGCATAAATTCCGTCAGCCAGTTTAGTCTGACCATCTCATCTGTAACATCATTGGCAA
CGCTACCTTTGCCATGTTTCAGAAACAACTCTGGCGCATCGGGCTTCCCATACAATCGATAGATTGTCGCACCTGATTGCCCG
ACATTATCGCGAGCCCATTTATACCCATATAAATCAGCATCCATGTTGGAATTTAATCGCGGCCTCGAGCAAGACGTTTCCCG
TTGAATATGGCTCATAACACCCCTTGTATTACTGTTTATGTAAGCAGACAGTTTTATTGTTCATGATGATATATTTTTATCTT
GTGCAATGTAACATCAGAGATTTTGAGACACAACGTGGCTTTCCCTGCAGGATTTCGGAGGCCTGCGTTATCCCCTGATTCTG
TGGATAACCGTATTACCGCCTTTGAGTGAGCTGATACCGCTCGCCGCAGCCGAACGCCGACTAGTGGATTTTACGGCTAGCTC
AGTCCTAGGTACAATGCTAGCGAATTCATTAAAGAGGAGAAAGGTACCCATGGCACGTACCCCGAGCCGTAGCAGCATTGGTA
GCCTGCGTAGTCCGCATACCCATAAAGCAATTCTGACCAGCACCATTGAAATCCTGAAAGAATGTGGTTATAGCGGTCTGAGC
ATTGAAAGCGTTGCACGTCGTGCCGGTGCAAGCAAACCGACCATTTATCGTTGGTGGACCAATAAAGCAGCACTGATTGCCGA
AGTGTATGAAAATGAAAGCGAACAGGTGCGTAAATTTCCGGATCTGGGTAGCTTTAAAGCCGATCTGGATTTTCTGCTGCGTA
ATCTGTGGAAAGTTTGGCGTGAAACCATTTGTGGTGAAGCATTTCGTTGTGTTATTGCAGAAGCACAGCTGGACCCTGCAACC
CTGACCCAGCTGAAAGATCAGTTTATGGAACGTCGTCGTGAGATGCCGAAAAAACTGGTTGAAAATGCCATTAGCAATGGTGA
ACTGCCGAAAGATACCAATCGTGAACTGCTGCTGGATATGATTTTTGGTTTTTGTTGGTATCGCCTGCTGACCGAACAGCTGA
CCGTTGAACAGGATATTGAAGAATTTACCTTCCTGCTAATTAATGGTGTTTGTCCGGGTACACAGCGTTAACTAGGGCCCATA
CCCCCAATTATTGAAGGCCGCTAACGCGGCCTTTTTTTGTTTCTGGTCTGCCCGACGTACGGTGAATCTGATTCGTTACCAAT
TGACATGATACGAAACGTACCGTATCGTTAAGGTCGTGATTAAACGATCCCGTTTTCAAAAATGAAACTGGCACCGAACGTAA
AACAGCAGTCACGCGGCATAAAACACAAAGAAACAGAAGTCATTATTTTTGCGGGTAGTGATGCCTGGTCACACGCAAAACAA
TGGCAGGAACATGACGCGCGTATGGCCGGAGATAATGAGCCTCCTGTGTGGCTTGGGGAGCAGCAGTTATCCGAACTGGATAA
GCTGCAAATTGTGCCGGAAGGCAGAAAATCCGTGCGCATATTCAGGGCCGGATATCTTGCGCCAGTAATGATAAAGGCGATTG
GTCAGAAGCTGGCGGCGGCAGGCGTACAGGATGCAAATTTTTACCCTGATGGTATGCACGGTCAGAAGGTGGAGAACTGGCGC
GAATATCTGGCCCGTGAGCGCCAGAATCTTTCTGATGGTCTGGTCATTGAGCTTCCGGTAAAGCAAAAGGCGCAACTTTCGCA
GATGGCGGACAGTGAGCGCGCGCAGCTGCTTGCCGATCGCTTTGATGGCGTTTGCGTACATCCTGAAAGTGAAATCGTTCACG
TATGGCGCGGCGGGGTATGGTGTCCGGTCAGCACAATGGAGCTGAGCCGCGAAATGGTGGCGATCTATTCAGAGCACAGGGCC
ACTTTCAGCAAGCGCGTAATCAATAACGCCGTGGAAGCGTTAAAAGTTATTGCCGAACCAATGGGCGAGCCGTCCGGCGATTT
GCTGCCGTTCGCCAATGGTGCGCTTGACCTGAAAACGGGGGAATTTTCCCCGCACACGCCGGAGAACTGGATCACCACGCACA
ACGGCATTGAGTACACGCCACCAGCACCCGGGGAGAACATCCGCGATAACGCGCCAAACTTTCATAAATGGCTTGAGCACGCA
GCCGGAAAAGACCCGCGCAAGATGATGCGTATATGTGCCGCGCTGTACATGATTATGGCGAACCGGTACGACTGGCAGATGTT
TATTGAGGCCACCGGAGACGGCGGGAGCGGTAAAAGTACATTCACACACATAGCCAGCCTTCTGGCAGGGAAACAAAACACGG
TAAGCGCTGAAATGACATCGCTTGATGATGCTGGTGGGCGTGCGCAGGTTGTCGGGAGTCGTCTTATCGTCCTGGCAGACCAG
CCGAAATATACAGGCGAAGGAACGGGCATCAAGAAAATCACGGGCGGCGACCCCGTGGAAATTAACCCGAAATATGAAAAGCG
TTTTACGGCGGTAATCAGGGCGGTGGTGCTGGCAACCAATAACAATCCGATGATATTCACCGAACGGGCCGGAGGTGTGGCAC
GTCGTCGGGTGATATTCCGGTTCGATAACATCGTAAGCGAGGCAGAAAAAGACAGGGAGCTACCGGAAAAGATCGCGGCTGAA
ATCCCTGTCATTATCCGCCGCTTGCTGGCGAACTTTGCCGACCCTGAAAAGGCACGGGCTTTACTCATTGAACAGCGTGACGG
TGATGAAGCACTGGCAATAAAGCAACAGACGGATCCGGTTATTGAGTTTTGCCAGTTCCTGAATTTTCTGGAGGAAGCACGCG
GCCTGATGATGGGCGGCGGTGGCGATTCAGTGAAGTACACGACCAGAAACAGCCTTTACCGCGTCTATCTGGCGTTTATGGCG
TACGCAGGCAGGAGCAAACCGCTAAACGTAAATGACTTTGGCAAGGCTATGAAGCCAGCCGCGAAAGTTTACGGACATGAATA
TATTACGCGGAAAGTTAAAGGAGTAACGCAGACTAACGCAATAACAACAGACGATTGCGACGCGTTTTTATAATGACGCATCC
TCACGATAATATCCGGGTA

p1324

CTAATCTCTTGCCCCCGCCCGTAATAGCCTCCAAGAGATTGAAGATAGTAAAGGGCAAGAGCTGATTCGGCGTTGAAGGATAG
CGGACTTTCGGTCAACCACAATTCCCCACTCGACAAAACCAGCCGTGCGAATAACTCTGAAAGTACAAGCAACCCAAGAGGGC
TGAGCCTAAACTCAGCTAATTCCTAAGTGAGCTAAAGACTCGAAGTGACAGCTCTTAATAAATAGAGCGGGAACGTCGAACGG
TCGTGAAAGTAATAGTACAACGGGTATTAACTTACTGAGGATATTGCTTGAAGCTGTACCGTTTTATTGGGTGAACGAATAAG
ATCCAGCAATTCAGCCAAAGAAGCTACCAATTTTTAGTTTAAGAGTGTCACGTCTGACCTCGCGGGTAGATTGCCGAACGTAG
AGCTTACGAGCCAGCGGAAACAGTAGCCGCAGGATAAGTAAGGGGAGTAAGTGATCGAACGAATCAGAAGTGACAATATACTT
AGGCTGGATCTCGTCCCGTGAATCCCAACCCTCACCAACTACGAGATAAGAGGTAAGCCAAAAATCGACTTGGTGGCGACCAA
CGACTGTTCCCCCCCTGTAACTAATCGTTCCGTCAAAACCTGACTTACTTCAAGGCCAATTCCAAGCGCAAACAATACCGTCC
TAGTTCTTCGGTTAAGTTTCCGAAGTAGGAGTGAGCCTACCTCCGTTTGCGTCTTGTTACCACTGACCCAGCTATTTACTTTG
TATTGCCTGCAATCGAATTTCTGAACTCTCAGATAGTGGGGATAACGGGAAAGTTCCTATATTTGCGAACTAACTTAGCCGTC
CACCTCGAAGCTACCTACTCACACCCACCCCGCGCGGGGTAAATAAGGCACTAATCCCAGCTGAGAGCTGGCGTAGCACTTAG
CCACAAGTTAATTAACAGTTGTCTGGTAGTTTGGCGGTATTAGGAAGATCCTAGAAGCAAGGCAGAGTTAGTTCTAACCTAAA
GCCACAAATAAGACAGGTTGCCAAAGCCCGCCGGAAATTAAATCTTGCTCAGTTCGGTAACGGAGTTTCCCTCCCGCGTACTT
AATTCCCAATAAGAAACGCGCCCAAGTCCTATCAGGCAAAATTCAGCCCCTTCCCGTGTTAGAACGAGGGTAAAAATACAAGC
CGATTGAACAAGGGTTGGGGGCTTCAAATCGTCGTTTACCCCACTTTACAACGGAGATTAAGTAGTTCACCCTATAGTACGAA

GCAGAACTATTTCGAGGGGCGTGCAATAATCGAATCTTCTGCGGTTGACTTAACACGCTAGGGACGTGCCCTCGATTCAATCG
AAGGTACTCCTACTCAGACTGCCTCACACCCAGCTAGTCACTGAGCGATAAAATTGACCCGCCCTCTAGGGAAGCGAGTACGT
CCCAAAGGGCTCCGGACAGGGCTATATAGGAGAGTTTGATCTCGCCCCGACAACTGCAACCCTCAACTCCCTTAGATAATATT
GTTAGCCGAAGTTGCACGACCCGCCGTCCACGGACTGCTCTTAGGGTGTGGCTCCTTAATCTGACAACGTGCAACCCCTATCG
AAGTCGATTGTTTCTGCGAAAGGTGTTGTCCTAATAGTCCCGAAATTTGGCCCTTGTAGGTGTGAAACCACTTAGCTTCGCGC
CGTAGTCCTAAAGGCCCACCTATTGACTTTGTTTCGGGTAGCACTAGGAATCTTAACAATTTGAATTTGGACGTGGAACGCGT
ACACCTTAATCTCCGAATAATTCTAGGGATTTGGAAGTCCTCTACGTTGACACACCTACACTGCTCGAAGTAAATATACGAAT
AACGCGGGCCTCGCGGAGCCGTTCCGAATCGTCACGTGTTCGTTTACTGTTAATTGGTGGCAAATAAGCAATATCGTAGTCCG
TCAGGCCCAGCCCTGTTATCCACGGCGTTATTTGTCAAATTGCGTAGAACTGGATTGACTGCCTGACAATACCTAATTATCGG
TACGAAGTCCCCGAATCTGTCGGGCTATTTCACTAATACTTTCCAAACGCCCCGTATCCAAGAAGAACGAATTTATCCACGCT
CCCGTCTTTGGGACGAATACCGCTACAAGTGGACAGAGGATCGGTACGGGCCTCTAATAAATCCAACACTCTACGCCCTCTTC
AAGAGCTAGAAGAACAGGGTGCAGTTGGAAAGGGAATTATTTCGTAAGGCGAGCCAATACCGTAATTAATTCGGAAGAGTTAA
CACGATTGGAAGTAGGAATAGTTTCTAACCACGGTTACTAATCCTAATAACGGAACGCTGTCTGATAGATTAGTGTCAGCGCT
CGGTACCAAAGAAAAATAAAAAGACGCTGAAAAGCGTCTTTTTATTTTTCGGTCCAGTGTAACTCAGGCAAAAGCACGTAATA
TTCGTACTTTCTTCCTCCGTAAGCGTCACCCACATTCCTTAAAGAGTGCATGTGCATATTTTGTTATCAATAAAAAAGGCCGC
GATTTGCGGCCTTATTGTTCGTCTTGCCGGATTACGCCCCGCCCTGCCACTCATCGCAGTATTGTTGTAATTCATTAAGCATT
CTGCCGACATGGAAGCCATCACAAACGGCATGATGAACTTGGATCGCCAGTGGCATTAACACCTTGTCGCCTTGCGTATAATA
TTTTCCCATAGTGAAAACGGGGGCGAAGAAGTTGTCCATATTTGCTACGTTTAAATCAAAACTGGTGAAACTCACCCACGGAT
TGGCACTGACGAAAAACATATTTTCGATAAACCCTTTAGGGAAATATGCTAAGTTTTCACCGTAACACGCCACATCTTGACTA
TATATGTGTAGAAACTGCCGGAAATCGTCGTGGTATTCTGACCAGAGCGATGAAAACGTTTCAGTTTGCTCATGGAAAACGGT
GTAACAAGGGTGAACACTATCCCATATCACCAGCTCACCGTCTTTCATTGCCATACGAAACTCCGGATGTGCATTCATCAGGC
GGGCAAGAATGTGAATAAAGGCCGGATAAAACTTGTGCTTATTTTTCTTTACGGTTTTTAAAAAGGCCGTAATATCCAGCTGA
ACGGTTTGGTTATAGGTGCACTGAGCAACTGACTGGAATGCCTCAAAATGTTCTTTACGATGCCATTGACTTATATCAACTGT
AGTATATCCAGTGATTTTTTTCTCCATTTTAGCTTCCTTAGCTTGCGAAATCTCGATAACTCAAAAAATAGTAGTGATCTTAT
TTCATTATGGTGAAAGTTGTCTTACGTGCAACATTTTCGCAAAAAGTTGGCGCTTTATCAACACTGTCGGAATGACAAATGGT
TCCAATTATTGAACACCCTTCGGGGTGTTTTTTTGTTTCTGGTTTCCCGAGGCCGGCCTTTTGTTGCAATGGCTGTCTACCCT
GTCTACCTGAGTAAAGAAAAATACATTTAATTCAGTACATTAACTTGGGTAGACAGCCTTTTTTTACTGTCTACCTACTATCT
ACCCTCTCTACCTGATTTTACCTGAATCAGACAGGGAGGTAGATACGGGGTAGATAGTGGATAAAAGCACTCTACCCCACTGA
AAGCCGCGCCATTACTGGCATGGTGGCCAGTAAGGTAGATAAGGTAGACAAGGGGAGGCACAACTCAAAACTTTTTAAACGAG
GGGGTAAAACGCAGACCAAAACGATCTCAAGAAGATCATCTTATTAATCAGATAAAATATTTCTAGATTTCAGTGCAATTTAT
CTCTTCAAATGTAGCACCGGCGCGCCGTGACCAATTATTGAAGGCCGCTAACGCGGCCTTTTTTTGTTTCTGGTATCCCGAAT
GGAGCGACTTCTCCCCAAAAAGCCTCGCTTTCAGCACCTGTCGTTTCCTTTCTTTTCAGAGGGTATTTTAAATAAAAACATTA
AGTTATGACGAAGAAGAACGGAAACGCCTTAAACCGGAAAATTTTCATAAATAGCGAAAACCCGCGAGGTCGCCGCCCCGTAA
CCTGTCGGATCACCGGAAAGGACCCGTAAAGTGATAATGATTATCATCTACATATCACAACGTGCGTAAAGGGTAAGTATGAA
GGTCGTGTACTCCATCGCTACCAAATTCCAGAAAACAGACGCTTTCGAGCGTCTTTTTTCGTTTTGGTCACGACGTACGGTGG
AAATGTCCATCTATCAGGAGTTTGTTAACAAGTATTCCCTGTCTAAAACCCTGCGTTTTGAACTGATCCCGCAGGGCAAAACT
TTGGAAAACATTAAAGCGCGTGGCCTGATTCTGGATGACGAAAAACGTGCAAAGGATTACAAGAAAGCTAAACAGATCATCGA
CAAATATCACCAGTTCTTTATCGAAGAAATTCTGTCGTCGGTGTGCATCAGTGAGGATCTGTTACAGAATTATTCTGATGTAT
ACTTTAAACTTAAAAAGTCCGATGACGATAATCTGCAAAAAGATTTCAAGTCAGCCAAAGATACCATCAAGAAACAGATCTCA
GAATATATTAAAGATAGCGAAAAGTTCAAAAACCTGTTTAACCAAAACCTCATTGATGCTAAGAAAGGCCAAGAATCTGACCT
GATCTTATGGCTGAAACAGAGCAAAGATAACGGCATTGAACTGTTCAAAGCTAATAGCGACATCACCGATATTGATGAAGCGC
TCGAAATCATCAAGTCTTTCAAAGGCTGGACGACGTATTTCAAAGGTTTTCATGAAAACCGTAAGAATGTATATTCGAGCAAC
GATATTCCGACCTCTATTATTTATCGTATCGTGGACGACAACCTGCCGAAGTTTCTGGAAAACAAAGCGAAATATGAATCTCT
GAAAGACAAAGCACCGGAAGCTATTAACTATGAACAGATCAAGAAAGATCTGGCGGAAGAACTGACCTTCGACATCGACTATA
AAACCTCCGAAGTTAACCAGCGTGTTTTCTCACTGGACGAGGTTTTCGAAATCGCTAATTTCAACAATTACCTGAATCAATCT
GGCATCACCAAATTCAACACCATTATTGGTGGCAAATTTGTTAACGGCGAAAACACCAAGCGTAAGGGCATCAACGAATACAT
TAACCTGTATAGCCAACAAATCAACGACAAAACCCTGAAAAAGTATAAAATGTCCGTTCTGTTTAAACAGATTTTATCGGACA
CCGAATCTAAATCCTTCGTAATTGATAAACTGGAAGATGATAGCGACGTTGTCACCACGATGCAGAGCTTTTATGAGCAGATT
GCGGCGTTCAAAACCGTGGAAGAGAAATCTATTAAAGAAACTCTGTCCCTGCTCTTTGACGACCTCAAAGCGCAGAAACTAGA
TCTGTCTAAGATTTACTTTAAAAACGACAAATCTCTGACCGATCTCAGTCAACAAGTTTTCGATGACTATAGCGTGATCGGCA
CGGCAGTTTTGGAATACATCACCCAACAAATCGCGCCGAAAAATCTGGACAACCCGTCCAAGAAGGAACAGGAACTGATTGCA
AAGAAAACAGAAAAAGCTAAATACCTGAGCTTAGAAACTATCAAACTGGCACTTGAGGAATTTAATAAACATCGTGATATTGA
TAAACAGTGTCGTTTTGAGGAAATTCTGGCGAACTTTGCGGCAATCCCGATGATCTTCGACGAAATTGCTCAAAACAAAGACA
ATCTGGCGCAGATCTCTATCAAGTACCAGAATCAGGGTAAGAAAGATCTGCTTCAAGCATCTGCGGAGGACGATGTGAAAGCA
ATTAAAGACTTATTAGATCAGACGAATAACTTATTACACAAGCTCAAAATCTTCCACATCAGCCAGAGCGAGGACAAGGCGAA
CATTCTGGATAAAGATGAACACTTCTATCTGGTGTTCGAAGAATGTTACTTCGAACTGGCAAACATCGTCCCTCTCTACAATA
AAATCCGCAACTACATCACGCAGAAGCCTTACTCTGACGAGAAATTCAAACTGAACTTCGAAAACAGCACGCTGGCGAACGGC
TGGGATAAGAACAAAGAGCCGGACAACACCGCAATCCTGTTCATCAAAGACGACAAATACTATCTGGGCGTAATGAACAAGAA
GAACAACAAGATCTTCGACGATAAAGCGATCAAAGAAAACAAGGGTGAAGGCTATAAGAAAATCGTGTACAAGCTCCTGCCGG
GTGCGAATAAAATGTTACCGAAAGTGTTCTTTTCCGCGAAAAGCATCAAATTCTACAACCCGTCTGAGGATATTCTGCGCATC
CGCAATCATAGCACGCACACTAAAAACGGTAGCCCGCAGAAAGGGTATGAAAAATTCGAATTTAATATAGAGGACTGCCGTAA
GTTCATCGACTTCTATAAACAGAGCATTTCCAAACATCCGGAATGGAAAGACTTCGGCTTCCGTTTCTCTGACACTCAGCGCT
ATAATAGCATCGACGAGTTCTACCGCGAAGTGGAGAATCAGGGCTATAAACTGACCTTCGAGAACATTAGTGAGTCGTACATC

GACTCCGTTGTGAATCAGGGTAAACTGTACCTGTTTCAGATCTATAATAAAGACTTTAGCGCGTACAGCAAAGGCCGTCCGAA
TCTGCACACCCTTTACTGGAAAGCATTATTTGACGAACGTAACCTGCAAGATGTGGTGTATAAACTGAACGGTGAGGCGGAAC
TTTTCTACCGTAAACAGAGTATCCCGAAGAAAATCACGCATCCGGCAAAAGAAGCTATTGCCAACAAAAACAAAGACAACCCG
AAGAAAGAATCAGTATTCGAATATGACCTGATCAAAGATAAACGTTTCACCGAAGATAAGTTCTTTTTCCACTGTCCGATTAC
CATCAACTTCAAATCTAGCGGTGCGAACAAGTTCAACGATGAAATTAACTTATTACTGAAAGAGAAAGCTAATGACGTACACA
TCTTATCTATTGATCGCGGTGAACGTCATTTAGCATACTATACACTGGTAGATGGTAAAGGTAATATTATTAAACAGGATACT
TTCAATATTATCGGTAATGACCGTATGAAAACCAACTATCACGATAAGCTGGCGGCGATCGAAAAAGATCGTGATTCTGCGCG
TAAAGATTGGAAGAAAATTAACAATATCAAAGAAATGAAAGAAGGCTATCTGAGCCAAGTGGTGCACGAGATCGCAAAACTGG
TGATTGAATATAACGCTATCGTGGTTTTCGAAGATCTGAACTTTGGTTTTAAACGTGGTCGCTTCAAAGTAGAAAAACAGGTG
TACCAAAAACTGGAAAAAATGCTGATTGAAAAACTGAACTATCTGGTTTTTAAAGACAACGAATTTGACAAAACGGGTGGCGT
ACTCCGTGCCTATCAGCTGACCGCTCCGTTCGAAACGTTCAAGAAAATGGGTAAACAAACGGGGATTATCTATTATGTGCCAG
CTGGTTTCACCTCCAAGATTTGTCCAGTTACGGGCTTCGTTAACCAGCTGTACCCGAAATACGAGAGCGTTAGCAAATCTCAA
GAATTTTTCAGCAAATTCGACAAGATCTGCTATAATCTGGATAAAGGCTATTTCGAGTTCAGCTTCGATTACAAAAACTTCGG
CGATAAAGCGGCTAAAGGTAAGTGGACTATTGCTAGCTTTGGTAGCCGTCTGATTAACTTTCGCAACTCCGACAAAAACCATA
ATTGGGACACGCGTGAAGTGTATCCGACCAAAGAACTGGAAAAATTACTGAAAGACTATTCCATCGAATATGGTCATGGGGAG
TGCATTAAAGCGGCGATTTGCGGTGAATCCGATAAGAAATTTTTCGCCAAACTGACCAGCGTGCTTAACACCATTCTGCAAAT
GCGTAATTCTAAAACGGGTACGGAGCTGGACTACCTGATTTCTCCGGTAGCCGACGTTAACGGCAACTTCTTCGATTCTCGTC
AAGCACCGAAAAATATGCCACAAGACGCGGATGCCAACGGTGCATACCATATCGGCTTAAAAGGCTTAATGTTATTAGGCCGT
ATCAAGAATAATCAGGAGGGCAAGAAATTAAATCTGGTTATCAAAAACGAAGAATACTTCGAGTTCGTTCAGAATCGTAACAA
TTAATGTATGCTTAAGCAGCTCGGTACCAAAGACGAACAATAAGACGCTGAAAAGCGTCTTTTTTCGTTTTGGTCCTGTTGCG
GCGCGATAGTGTGAACATGCTATAGACTTCTGGTGCTACCCGACTGACAATTAATCATCCGGCTCGTATAATGCTAGCAATTT
CTACTGTTGTAGATACTGAGATGACATCTCCTGCGCGTTTCGAGACGAACAATAAGGCCTCCCTAACGGGGGGCCTTTTTTAT
TGATAACAAAAGTAACTTCGAGCTTGTCTACCTCCTAGCACTTTATGGCTAGCTCAGTCCTAGGTACAATGCTAGCGTTTTCA
TTAAAGAGGAGAAAGGAAGCCATGAGTAAAGGTGAGGAATTATTTACTGGTGTTGTTCCGATCTTAGTTGAACTGGACGGCGA
TGTTAACGGTCATAAATTCAGTGTTCGTGGTGAAGGTGAAGGTGATGCAACCAACGGTAAGCTGACCCTGAAATTCATCTGCA
CTACTGGAAAATTACCAGTACCGTGGCCTACTCTGGTGACTACCCTGACCTATGGTGTTCAGTGTTTTTCTCGTTACCCTGAC
CACATGAAGCAACATGATTTCTTCAAATCTGCAATGCCGGAAGGTTATGTACAGGAGCGCACCATTTCTTTCAAAGACGATGG
CACGTATAAAACCCGTGCAGAGGTTAAATTTGAAGGTGACACTCTGGTGAATCGTATTGAACTGAAAGGCATTGATTTCAAAG
AGGACGGCAATATTTTAGGCCACAAACTGGAATATAACTTCAACTCCCATAACGTTTACATCACCGCAGACAAACAGAAGAAC
GGTATCAAAGCTAACTTCAAAATTCGCCATAACGTTGAAGATGGTAGCGTACAGCTGGCGGATCATTACCAACAGAACACTCC
GATTGGAGATGCTCCTGTTTTACTGCCGGATAACCACTACCTGTCCACCCAGTCTAAACTGTCGAAGGATCCGAACGAAAAGC
GCGACCACATGGTGTTATTAGAGTTCGTTACCGCTAGTGGTATCACGCACGGTATGGATGAACTCTACAAATAAGACGAACAA
TAAGGGGAGCGGGAAACCGCTCCCCTTTTTTATTGATAACAAAAGTAAATTGCACGCTGATAGTCTCCCAATTGCGAAGGACC
AAAACGAAAAAACACCCTTTCGGGTGTCTTTTCTGGAATTTGGTACCGAGTACTAGGTATCGTGTAAGTAGCGAAGGCCCGTA
CGCGAGATAAACTGCTAGGCAACCGCGACTCTACGACTGGTGCTCGATTTAATTTCGCTGACGTAAAGAAATTATCGGCAGTG
CGTCAACTGCCGTATCTTTATCTTAATTAGGTAGTTGGACAAGCCCTTGAAAGAAATAGCAAGAGCCTGCCTCTCTATTGAAG
TCACGGCGAAAGTCGGGTAGAAATCAAAGAAAGCAGAAATTAAATCGGAGTAACACTAAGGTGGGATAACTCCGTAACTGACT
ACGCCTTTCTCTAGACTTTACTTGACCAGATACACTGTCTTTGACACGTTGAAGGATTAGAGCAATCAAATCCAAGACTGGCT
AAGCACGAAGCAACTCTTGAGTGTTAAAAAGTTATCTCCTGTATTCGGGAAGCGGGTACTAGAAGATTGCAGGGACTCCGACG
TTAAGTAAATTACAAAGTAATAAGTATCGTTCAGGATCACGTTACCGCAATAAGAAGCGAGAATAATATAATTTCCGAAGTGC
TTACCCCAGTAGTGACTATTCCTATAACCCTTCTGAGTGTCCGGAGGCGGAAATTTGCCACGAAAGAGAAAGTATTTCCCCGA
CAATAATAAAGGGGCGCTCCTCAGCTTTTCCACTTGGTTGGGTAAGCTAGGCAACTCTGAAAGGAGTTTCGGCGAATTGAAGC
CGACAGCTTTGAATTGTTTTAGGGGCGTTATTCGAGGGCAATCGGAGCTAACTTCAAGACTACTTCTTTGTTGAATACTAAAT
AGTGCAAAGGTCGTGTTTCCTCAAGGATACTCCGCTAACAATATAGGATTCCAATCAGATTCAGCACTGGCGGTACGGGTGTT
GCGGTGAGGCGTTCGGGTTTACGGCTCGAAGCTAGCACGGTAGGAAGCCTGACAATCACCAAGCAAAAGGGCCGTCGAAGGCC
CACAAGATACGAAAGCTCTCGAAGCCTTATCCTTGACCGATCCACCTATTTAGGCAGTTACGCACAAAAGCTACCCAATAATC
CGTGACAGGCACAATATCACGGAACAAAACCGAAAACTCTCGTACACGGTTAGGTTTTCGCTAGGAAGAATAAACCTCTATCT
TGATTATAAGAAGGCTCCCCAAGCACCCCCAAAACCGAAATAGCGGTTTGCAATAAGGGACAAGTTACGAGTGTAGACACGCA
GAATTATCCAGCCTTTAGTCTTTAGGAAGGCAAAGCTATTGTACGCGGTAGCCGTCGTAGCAATTTACCAACTGTAGAATTAT
TGGACACACGTAGGAAGGGCTTACAGTTGAAGTTTAATAAGGTCACACGCAAAACCGCTAAGGAATAATCGCACCGTTAGCGA
AAGAATATTTCAGAGCGGTTAGTAAAGGTTGAGTAAAGTGAGATTCCAAAGTGAGCCTTTATAAAAAGTAAAGAGCTATAATA
AAACCGTCGAGCAGAAAACAATCGCCTGAAATCTCAAGCACGTTGCCCTTTCTAACGTCGCTAAGGTTTCGTAAACCCGTTTG
ATTAGGAAGAAGAATAAGTAACCCGATTAGGTTTGAGATCGCGGGTTATCGGTTTGGATTAAAAGTGGATACCAGCGGAGTCA
ACGCCGACGCAAACGTACAGTGATCCAATCCTGTTGCACGGTCAAGCACAATCAGCTCGCAAGATCTTGGAATAGTGTGCCCA
ACAGTTTAGTTGAGGGCCACGTTCCGACTACAAGTTGCTTCAAGAGGGGAATTTGGATTTGGCAATAGCCCCCCGTTTCTACC
TCAAGAGGCGACGAGTATTAACCGCGCCAGCTGTCGGCACAAGGGCCAAAGAAGATTCCAATTTCTTATTCCCGAATAACCTC
CGAATCCCTGCGGGAAAATCACCGACCGAATAGCCTAGAAGCAAGGGGGAACAGATAGGTATAATTAGCTTAAGAGAGTACCA
GCCGTGACAACAGCGTAGTAACCACAAACTTACGCTGGGGCTTCTTTGGCGGATTTTTACAGATACTAACAAGGTGATTTGAA
GTACCTTAGTTGAGGATTTAAACGCGCTATCCGGTAATCTCCAAATTGGGAAATACCGTTCAAAGAGGGCTAGAATTACTTAA
AAGCCTTCACACCGCCTGCGCTATACGCGCCCACTCTCCCGTTTATCCGTCCAAGCGGAAGCAGGGCGATCCTCCGCTAAGAT
ATTCTTACGTGTAACGTAGCTAAGTATCCCAAATAGCTGGCGTACGCGTTGAACACCGCCTAGAGGATCGTGACTCGCCGGAC
GAGCGTGTTATTGGGGACTTACGCCAGCGTAGACTACAACGCGCCCAGATTAACCCTGCACGTATTGCCTTGAATAACGTACT
AATCTCTCCGGCTCTCGACAATCTATCGAGCGACTCGATTATCAACGGGTGTCTTGCAGTT

p1396

CGCAGGATAAGTAAGGGGAGTAAGTGATCGAACGAATCAGAAGTGACAATATACTTAGGCTGGATCTCGTCCCGTGAATCCCA
ACCCTCACCAACTACGAGATAAGAGGTAAGCCAAAAATCGACTTGGTGGCGACCAACGACTGTTCCCCCCCTGTAACTAATCG
TTCCGTCAAAACCTGACTTACTTCAAGGCCAATTCCAAGCGCAAACAATACCGTCCTAGTTCTTCGGTTAAGTTTCCGAAGTA
GGAGTGAGCCTACCTCCGTTTGCGTCTTGTTACCACTGACCCAGCTATTTACTTTGTATTGCCTGCAATCGAATTTCTGAACT
CTCAGATAGTGGGGATAACGGGAAAGTTCCTATATTTGCGAACTAACTTAGCCGTCCACCTCGAAGCTACCTACTCACACCCA
CCCCGCGCGGGGTAAATAAGGCACTAATCCCAGCTGAGAGCTGGCGTAGCACTTAGCCACAAGTTAATTAACAGTTGTCTGGT
AGTTTGGCGGTATTAGGAAGATCCTAGAAGCAAGGCAGAGTTAGTTCTAACCTAAAGCCACAAATAAGACAGGTTGCCAAAGC
CCGCCGGAAATTAAATCTTGCTCAGTTCGGTAACGGAGTTTCCCTCCCGCGTACTTAATTCCCAATAAGAAACGCGCCCAAGT
CCTATCAGGCAAAATTCAGCCCCTTCCCGTGTTAGAACGAGGGTAAAAATACAAGCCGATTGAACAAGGGTTGGGGGCTTCAA
ATCGTCGTTTACCCCACTTTACAACGGAGATTAAGTAGTTCACCCTATAGTACGAAGCAGAACTATTTCGAGGGGCGTGCAAT
AATCGAATCTTCTGCGGTTGACTTAACACGCTAGGGACGTGCCCTCGATTCAATCGAAGGTACTCCTACTCAGACTGCCTCAC
ACCCAGCTAGTCACTGAGCGATAAAATTGACCCGCCCTCTAGGGAAGCGAGTACGTCCCAAAGGGCTCCGGACAGGGCTATAT
AGGAGAGTTTGATCTCGCCCCGACAACTGCAACCCTCAACTCCCTTAGATAATATTGTTAGCCGAAGTTGCACGACCCGCCGT
CCACGGACTGCTCTTAGGGTGTGGCTCCTTAATCTGACAACGTGCAACCCCTATCGAAGTCGATTGTTTCTGCGAAAGGTGTT
GTCCTAATAGTCCCGAAATTTGGCCCTTGTAGGTGTGAAACCACTTAGCTTCGCGCCGTAGTCCTAAAGGCCCACCTATTGAC
TTTGTTTCGGGTAGCACTAGGAATCTTAACAATTTGAATTTGGACGTGGAACGCGTACACCTTAATCTCCGAATAATTCTAGG
GATTTGGAAGTCCTCTACGTTGACACACCTACACTGCTCGAAGTAAATATACGAATAACGCGGGCCTCGCGGAGCCGTTCCGA
ATCGTCACGTGTTCGTTTACTGTTAATTGGTGGCAAATAAGCAATATCGTAGTCCGTCAGGCCCAGCCCTGTTATCCACGGCG
TTATTTGTCAAATTGCGTAGAACTGGATTGACTGCCTGACAATACCTAATTATCGGTACGAAGTCCCCGAATCTGTCGGGCTA
TTTCACTAATACTTTCCAAACGCCCCGTATCCAAGAAGAACGAATTTATCCACGCTCCCGTCTTTGGGACGAATACCGCTACA
AGTGGACAGAGGATCGGTACGGGCCTCTAATAAATCCAACACTCTACGCCCTCTTCAAGAGCTAGAAGAACAGGGTGCAGTTG
GAAAGGGAATTATTTCGTAAGGCGAGCCAATACCGTAATTAATTCGGAAGAGTTAACACGATTGGAAGTAGGAATAGTTTCTA
ACCACGGTTACTAATCCTAATAACGGAACGCTGTCTGATAGATTAGTGTCAGCGCTCGGTACCAAAGAAAAATAAAAAGACGC
TGAAAAGCGTCTTTTTATTTTTCGGTCCAGTGTAACTCAGGCAAAAGCACGTAATATTCGTACTTTCTTCCTCCGTAAGCGTC
ACCCACATTCCTTAAAGAGTGCATGTGCATATTTTGTTATCAATAAAAAAGGCCGCGATTTGCGGCCTTATTGTTCGTCTTGC
CGGATTACGCCCCGCCCTGCCACTCATCGCAGTATTGTTGTAATTCATTAAGCATTCTGCCGACATGGAAGCCATCACAAACG
GCATGATGAACTTGGATCGCCAGTGGCATTAACACCTTGTCGCCTTGCGTATAATATTTCCCATAGTGAAAACGGGGGCGAA
GAAGTTGTCCATATTTGCTACGTTTAAATCAAAACTGGTGAAACTCACCCACGGATTGGCACTGACGAAAAACATATTTTCGA
TAAACCCTTTAGGGAAATATGCTAAGTTTTCACCGTAACACGCCACATCTTGACTATATATGTGTAGAAACTGCCGGAAATCG
TCGTGGTATTCTGACCAGAGCGATGAAAACGTTTCAGTTTGCTCATGGAAAACGGTGTAACAAGGGTGAACACTATCCCATAT
CACCAGCTCACCGTCTTTCATTGCCATACGAAACTCCGGATGTGCATTCATCAGGCGGGCAAGAATGTGAATAAAGGCCGGAT
AAAACTTGTGCTTATTTTTCTTTACGGTTTTTAAAAAGGCCGTAATATCCAGCTGAACGGTTTGGTTATAGGTGCACTGAGCA
ACTGACTGGAATGCCTCAAAATGTTCTTTACGATGCCATTGACTTATATCAACTGTAGTATATCCAGTGATTTTTTTCTCCAT
TTTAGCTTCCTTAGCTTGCGAAATCTCGATAACTCAAAAAATAGTAGTGATCTTATTTCATTATGGTGAAAGTTGTCTTACGT
GCAACATTTTCGCAAAAAGTTGGCGCTTTATCAACACTGTCGGAATGACAAATGGTTCCAATTATTGAACACCCTTCGGGGTG
TTTTTTTGTTTCTGGTTTCCCGAGGCCGGCCTGCGCTAGCGGAGTGTATACTGGCTTACTATGTTGGCACTGATGAGGGTGTA
AGTGAAGTGCTTCATGTGGCAGGAGAAAAAAGGCTGCATCGGTGCGTCAGCAGAATATGTGATACAGGATATATTCCGCTTCC
TCGCTCACTGACTCGCTACGCTCGGTCGTTCGACTGTGGCGAGCGGAAATGGCTTACGAACGGGGCGGAGATTTCCTGGAAGA
TGCCAGGAAGATACTTAACAGGGAAGTGAGAGGGTCGCGGCAAAGCCGTTTTTCCATAGGCTCCGCCCCCCTGACAAGCATCA
CGAAATCTGACGCTCAAATCAGTGGTGGCGAAACCTGACAGGACTATAAAGATACCAGGCGTTTCCCCCTGGCGGCTCCCTCG
TGCGCTCTCCTGTTCCTGCCTTTCGGTTTGCCGGTGTCATTCCTCTGTTACGGCCGAGTTTGTCTCATTCCACGCCTGACACT
CAGTTCCGGGTAGGCAGTTCGCTCCAAGCTGGACTGTATGCACGAACCCCCCGTTCAGTCCGACCGCTGCGCCTTATCCGGTA
ACTATCGTCTTGAGTCCAACCCGGAAAGACATGCAAAAGCACCACTGGCAGCAGCCACTGGTAATTGATTTAGAGGAGTTAGT
CTTGAAGTCATGCGCCGGATAAGGCTAAACTGAAAGGACAAGTTTTGGCGACTGCGCTCCTCCAAGCCAGTTACCTCGGTTCA
AAGAGTTGGTAGCTCAGAGAACCTTCGAAAAACCGCCCTGCAAGGCGGTTTTTTCGTTTTCAGAGCAAGAGATTACGCGCAGA
CCAAAACGATCTCAAGAAGATCATCTTATTAATCAGATAAAATATTTCTAGATTTCAGTGCAATTTATCTCTTCAAATGTAGC
ACCGGCGCGCCGTGACCAATTATTGAAGGCCGCTAACGCGGCCTTTTTTTGTTTCTGGTATCCCGAATGGAGCGACTTCTCCC
CAAAAAGCCTCGCTTTCAGCACCTGTCGTTTCCTTTCTTTTCAGAGGGTATTTTAAATAAAAACATTAAGTTATGACGAAGAA
GAACGGAAACGCCTTAAACCGGAAAATTTTCATAAATAGCGAAAACCCGCGAGGTCGCCGCCCCGTAACCTGTCGGATCACCG
GAAAGGACCCGTAAAGTGATAATGATTATCATCTACATATCACAACGTGCGTAAAGGGTAAGTATGAAGGTCGTGTACTCCAT
CGCTACCAAATTCCAGAAAACAGACGCTTTCGAGCGTCTTTTTTCGTTTTGGTCACGACGTACGGTGGAAGATTCGTTACCAA
TTGACAGCTAGCTCAGTCCTAGGTATATACATACATGCTTGTTTGTTTGTAAACGGAGATAAATGGTGAATGCGAGATGAAGC
GTACCGCCGATGGCAGCGAGTTTGAATCTCCGAAGAAAAAGCGTAAGGTCAGCGAAGTTGAGTTCAGCCACGAATACTGGATG
CGTCACGCTTTAACCCTGGCTAAACGCGCGCGCGACGAGCGCGAAGTACCAGTGGGGGCGGTGCTGGTGTTAAACAACCGCGT
AATCGGCGAAGGCTGGAACCGTGCAATCGGGTTACATGACCCGACCGCCCATGCCGAGATCATGGCCCTGCGCCAGGGGGGGC
TGGTCATGCAGAATTACCGTCTGATCGACGCGACGTTGTATGTCACATTCGAGCCATGCGTGATGTGTGCGGGGGCAATGATT
CACTCTCGCATTGGTCGCGTCGTGTTTGGCGTTCGTAATAGTAAACGCGGCGCTGCTGGCTCCTTAATGAATGTTCTGAATTA
TCCGGGTATGAACCACCGTGTCGAAATTACAGAAGGTATCTTAGCAGATGAATGTGCCGCACTGCTGTGTGACTTCTACCGCA
TGCCGCGCCAAGTATTCAACGCCCAAAAAAAAGCTCAGTCCTCAATTAACTCTGGTGGTAGTAGTGGCGGCTCTAGCGGCTCC

GAGACGCCTGGTACGTCGGAATCGGCTACGCCTGAGTCGAGCGGTGGGTCCTCTGGCGGCTCTGACAAGAAATATAGCATCGG
CCTGGCCATCGGCACAAATAGCGTCGGATGGGCGGTGATCACTGATGAATATAAAGTTCCGTCTAAAAAGTTCAAGGTACTGG
GTAATACAGATCGCCATAGTATCAAAAAGAACTTAATCGGTGCGCTTCTGTTCGATTCCGGCGAAACCGCAGAAGCAACACGT
CTGAAACGCACCGCTCGTCGCCGTTACACCCGTCGTAAAAACCGCATCTGCTACCTGCAAGAAATCTTCTCTAACGAAATGGC
TAAAGTAGATGACAGCTTTTTTCACCGTCTGGAAGAATCATTTCTGGTGGAAGAAGATAAAAAGCACGAACGTCATCCAATCT
TCGGCAACATTGTGGACGAAGTAGCGTATCACGAAAAATACCCGACTATCTATCACCTGCGCAAAAAGCTGGTCGATTCGACG
GATAAGGCCGATCTGCGTCTGATCTATCTGGCCTTAGCGCATATGATTAAGTTCCGTGGTCATTTCCTGATCGAAGGCGACCT
GAATCCAGACAACAGCGATGTAGACAAACTGTTCATCCAGCTGGTGCAAACCTATAACCAGCTGTTTGAAGAAAACCCAATTA
ATGCTAGCGGTGTTGACGCGAAAGCGATCTTGTCCGCACGCCTGTCCAAATCCCGTCGTCTGGAAAACTTAATTGCGCAACTG
CCGGGTGAGAAGAAAAACGGACTGTTCGGCAATCTGATCGCTCTTAGCTTGGGACTGACCCCGAACTTCAAAAGCAACTTCGA
TCTGGCAGAGGACGCAAAACTTCAACTTAGCAAAGATACGTATGACGATGACTTGGATAACTTACTGGCCCAGATCGGAGATC
AGTACGCTGATCTGTTTCTGGCGGCAAAGAACTTATCAGACGCTATTCTCCTGTCTGATATTCTTCGTGTGAATACCGAAATC
ACCAAAGCACCGCTTTCTGCATCCATGATTAAACGCTATGACGAACATCACCAAGATCTGACTCTTCTGAAAGCGCTGGTACG
GCAACAACTGCCGGAGAAGTACAAGGAGATCTTCTTTGACCAATCCAAAAACGGCTACGCGGGTTATATTGACGGGGGTGCAA
GCCAAGAGGAGTTCTACAAATTCATCAAGCCAATCTTAGAAAAAATGGATGGCACGGAAGAATTACTTGTTAAACTGAATCGT
GAGGATCTGCTTCGTAAACAGCGTACCTTCGACAACGGTAGCATTCCGCACCAGATCCACTTAGGTGAACTGCACGCTATCCT
GCGTCGCCAAGAGGATTTTTACCCGTTCCTGAAAGATAATCGTGAAAAAATCGAAAAAATCCTGACCTTTCGTATCCCGTATT
ATGTCGGCCCGCTGGCGCGTGGCAACTCCCGTTTCGCGTGGATGACTCGCAAATCCGAAGAAACTATTACCCCGTGGAACTTC
GAGGAAGTGGTTGACAAAGGCGCAAGCGCCCAATCCTTCATCGAGCGCATGACTAACTTTGATAAAAACCTGCCGAACGAAAA
GGTACTGCCGAAACACTCCCTTCTGTACGAATACTTCACCGTGTACAACGAGCTGACTAAAGTAAAGTATGTGACTGAGGGCA
TGCGTAAACCTGCATTCCTGAGCGGTGAACAGAAAAAAGCAATTGTTGATTTACTGTTTAAAACCAACCGTAAAGTAACCGTT
AAACAGCTGAAAGAGGACTACTTCAAGAAAATCGAATGCTTCGACTCCGTCGAGATTAGTGGAGTTGAAGATCGTTTTAATGC
AAGTTTAGGCACGTATCACGATTTATTAAAGATCATTAAAGACAAAGATTTCTTGGACAACGAAGAAAATGAGGACATCTTAG
AGGACATCGTCCTGACCCTGACTCTGTTCGAAGATCGTGAAATGATTGAAGAACGCCTTAAGACGTATGCTCACCTGTTTGAC
GATAAAGTAATGAAACAACTGAAACGTCGCCGTTATACTGGCTGGGGCCGTCTGAGCCGTAAACTGATTAACGGTATCCGTGA
CAAACAGTCCGGTAAAACTATTCTGGACTTCCTGAAATCTGACGGCTTCGCAAACCGTAACTTCATGCAACTGATTCACGACG
ATTCCCTGACCTTCAAAGAGGACATCCAGAAAGCTCAGGTTTCTGGTCAAGGTGATTCTCTGCACGAGCATATCGCCAATTTA
GCAGGTAGTCCGGCGATCAAAAAAGGTATCCTGCAAACCGTGAAAGTGGTGGATGAGCTTGTGAAAGTTATGGGTCGTCACAA
ACCGGAAAACATTGTTATCGAGATGGCTCGTGAAAACCAAACGACCCAGAAGGGACAGAAAAACTCCCGCGAACGCATGAAAC
GTATCGAGGAGGGTATTAAAGAACTTGGCTCTCAGATTCTGAAAGAACACCCTGTTGAAAATACCCAACTGCAAAATGAAAAA
CTGTACCTGTACTACCTGCAAAATGGTCGTGACATGTATGTAGATCAGGAGCTGGACATCAACCGCCTCTCCGATTACGACGT
TGACCACATTGTTCCGCAGTCTTTTCTGAAAGATGATTCCATTGATAACAAAGTACTCACCCGTAGCGATAAAAACCGTGGGA
AGAGTGACAACGTTCCATCGGAAGAAGTAGTTAAGAAAATGAAGAACTATTGGCGTCAACTGCTTAACGCGAAACTGATTACT
CAACGTAAATTTGATAACCTGACCAAAGCTGAACGTGGCGGTTTGTCTGAGCTGGATAAGGCGGGTTTTATTAAACGTCAACT
GGTAGAAACTCGCCAGATTACAAAACATGTTGCTCAGATTCTGGACTCTCGTATGAACACTAAATACGATGAAAATGACAAAC
TGATCCGCGAAGTTAAGGTTATTACCCTGAAATCTAAGCTGGTTTCCGACTTCCGTAAAGATTTCCAATTCTATAAAGTGCGC
GAGATTAACAACTATCACCACGCGCACGACGCATATCTGAATGCAGTTGTTGGCACGGCACTGATCAAAAAATATCCGAAACT
GGAAAGCGAATTTGTGTACGGCGATTATAAAGTTTACGACGTGCGCAAAATGATCGCCAAATCTGAACAGGAAATTGGCAAAG
CAACCGCTAAATACTTTTTCTACTCAAACATTATGAATTTCTTCAAAACCGAAATCACCTTAGCGAATGGCGAAATTCGTAAA
CGCCCTCTGATCGAAACCAACGGCGAAACGGGTGAGATCGTGTGGGACAAAGGTCGTGATTTCGCTACTGTCCGCAAAGTTCT
GTCCATGCCTCAAGTAAACATCGTTAAAAAGACTGAGGTACAGACTGGCGGTTTCAGCAAGGAATCCATTCTGCCGAAACGCA
ACTCCGACAAACTGATCGCGCGTAAGAAAGACTGGGATCCGAAGAAATACGGTGGCTTCGATTCTCCAACCGTGGCATACAGC
GTTCTGGTAGTCGCCAAAGTCGAAAAGGGTAAATCAAAAAAACTGAAATCAGTGAAAGAACTTTTAGGCATCACCATTATGGA
ACGTAGCTCTTTCGAAAAAAACCCGATTGACTTCCTCGAAGCGAAGGGGTACAAGGAAGTAAAGAAAGATCTGATTATCAAAC
TGCCGAAGTATTCCCTGTTCGAACTGGAAAATGGTCGTAAACGTATGTTAGCGTCTGCGGGTGAACTGCAAAAAGGAACGAA
TTGGCCCTTCCGTCCAAGTACGTGAACTTCCTGTATCTGGCCTCGCACTACGAGAAACTGAAAGGTAGTCCGGAAGATAATGA
GCAGAAACAGCTGTTCGTGGAACAGCACAAACACTATCTGGACGAGATTATTGAACAGATTTCTGAGTTTAGCAAACGCGTAA
TTCTGGCGGACGCGAATCTGGATAAAGTCCTGAGCGCCTACAATAAACACCGTGATAAACCGATCCGTGAACAGGCAGAAAAC
ATCATTCACCTGTTCACGCTGACTAATCTTGGTGCTCCGGCAGCCTTCAAATACTTCGACACCACGATCGATCGTAAACGTTA
CACCTCCACTAAAGAAGTCTTAGATGCAACTCTTATTCACCAGAGCATCACTGGCCTGTATGAAACTCGTATTGATCTGAGTC
AGTTGGGCGGTGACTAATGTATGCTTAAGCAGCTCGGTACCAAAGACGAACAATAAGACGCTGAAAAGCGTCTTTTTTCGTTT
TGGTCCTGTTGCGGCGCGATAGTGTGAACATGCTATAGACTTCTGGTGCTACCCGACTGACAATTAATCATCCGGCTCGTATA
ATGCTAGCACTTTTAAAGTTCTGCTATGGTTTTAGAGCTAGAAATAGCAAGTTAAAATAAGGCTAGTCCGTTATCAACTTGAA
AAAGTGGCACCGAGTCGGTGCTTTTTTTAATCCATTCGAATCACGTCCGACGAACAATAAGGCCTCCCTAACGGGGGGCCTTT
TTTATTGATAACAAAAGTAACTTCGAGCTTGTCTACCTCCTAGCACCATTATTGCAATTAATAAACAACTAACGGACAATTCT
ACCTAACAGTTTTCATATATGACGAGCAGTTAAGTGATGAGTAAAGGTGAGGAATTATTTACTGGTGTTGTTCCGATCTTAGT
TGAACTGGACGGCGATGTTAACGGTCATAAATTCAGTGTTCGTGGTGAAGGTGAAGGTGATGCAACCAACGGTAAGCTGACCC
TGAAATTCATCTGCACTACTGGAAAATTACCAGTACCGTGGCCTACTCTGGTGACTACCCTGACCTATGGTGTTCAGTGTTTT
TCTCGTTACCCTGACCACATGAAGCAACATGATTTCTTCAAATCTGCAATGCCGGAAGGTTATGTACAGGAGCGCACCATTTC
TTTCAAAGACGATGGCACGTATAAAACCCGTGCAGAGGTTAAATTTGAAGGTGACACTCTGGTGAATCGTATTGAACTGAAAG
GCATTGATTTCAAAGAGGACGGCAATATTTTAGGCCACAAACTGGAATATAACTTCAACTCCCATAACGTTTACATCACCGCA
GACAAACAGAAGAACGGTATCAAAGCTAACTTCAAAATTCGCCATAACGTTGAAGATGGTAGCGTACAGCTGGCGGATCATTA
CCAACAGAACACTCCGATTGGAGATGCTCCTGTTTTACTGCCGGATAACCACTACCTGTCCACCCAGTCTAAACTGTCGAAGG

ATCCGAACGAAAAGCGCGACCACATGGTGTTATTAGAGTTCGTTACCGCTAGTGGTATCACGCACGGTATGGATGAACTCTAC
AAATAAGACGAACAATAAGGGGAGCGGGAAACCGCTCCCCTTTTTTATTGATAACAAAAGTAAATTGCACGCTGATAGTCTCC
CAATTGCGAAGGACCAAAACGAAAAAACACCCTTTCGGGTGTCTTTTCTGGAATTTGGTACCGAGTACTAGGTATCGTGTAAG
TAGCGAAGGCCCGTACGCGAGATAAACTGCTAGGCAACCGCGACTCTACGACTGGTGCTCGATTTAATTTCGCTGACGTAAAG
AAATTATCGGCAGTGCGTCAACTGCCGTATCTTTATCTTAATTAGGTAGTTGGACAAGCCCTTGAAAGAAATAGCAAGAGCCT
GCCTCTCTATTGAAGTCACGGCGAAAGTCGGGTAGAAATCAAAGAAAGCAGAAATTAAATCGGAGTAACACTAAGGTGGGATA
ACTCCGTAACTGACTACGCCTTTCTCTAGACTTTACTTGACCAGATACACTGTCTTTGACACGTTGAAGGATTAGAGCAATCA
AATCCAAGACTGGCTAAGCACGAAGCAACTCTTGAGTGTTAAAAAGTTATCTCCTGTATTCGGGAAGCGGGTACTAGAAGATT
GCAGGGACTCCGACGTTAAGTAAATTACAAAGTAATAAGTATCGTTCAGGATCACGTTACCGCAATAAGAAGCGAGAATAATA
TAATTTCCGAAGTGCTTACCCCAGTAGTGACTATTCCTATAACCCTTCTGAGTGTCCGGAGGCGGAAATTTGCCACGAAAGAG
AAAGTATTTCCCCGACAATAATAAAGGGGCGCTCCTCAGCTTTTCCACTTGGTTGGGTAAGCTAGGCAACTCTGAAAGGAGTT
TCGGCGAATTGAAGCCGACAGCTTTGAATTGTTTTAGGGGCGTTATTCGAGGGCAATCGGAGCTAACTTCAAGACTACTTCTT
TGTTGAATACTAAATAGTGCAAAGGTCGTGTTTCCTCAAGGATACTCCGCTAACAATATAGGATTCCAATCAGATTCAGCACT
GGCGGTACGGGTGTTGCGGTGAGGCGTTCGGGTTTACGGCTCGAAGCTAGCACGGTAGG

p1471

TCAGATCCTTCCGTATTTAGCCAGTATGTTCTCTAGTGTGGTTCGTTGTTTTTGCGTGAGCCATGAGAACGAACCATTGAGAT
CATACTTACTTTGCATGTCACTCAAAAATTTTGCCTCAAAACTGGTGAGCTGAATTTTTGCAGTTAAAGCATCGTGTAGTGTT
TTTCTTAGTCCGTTACGTAGGTAGGAATCTGATGTAATGGTTGTTGGTATTTTGTCACCATTCATTTTTATCTGGTTGTTCTC
AAGTTCGGTTACGAGATCCATTTGTCTATCTAGTTCAACTTGGAAAATCAACGTATCAGTCGGGCGGCCTCGCTTATCAACCA
CCAATTTCATATTGCTGTAAGTGTTTAAATCTTTACTTATTGGTTTCAAAACCCATTGGTTAAGCCTTTTAAACTCATGGTAG
TTATTTTCAAGCATTAACATGAACTTAAATTCATCAAGGCTAATCTCTATATTTGCCTTGTGAGTTTTCTTTTGTGTTAGTTC
TTTTAATAACCACTCATAAATCCTCATAGAGTATTTGTTTTCAAAAGACTTAACATGTTCCAGATTATATTTTATGAATTTTT
TTAACTGGAAAAGATAAGGCAATATCTCTTCACTAAAAACTAATTCTAATTTTTCGCTTGAGAACTTGGCATAGTTTGTCCAC
TGGAAAATCTCAAAGCCTTTAACCAAAGGATTCCTGATTTCCACAGTTCTCGTCATCAGCTCTCTGGTTGCTTTAGCTAATAC
ACCATAAGCATTTTCCCTACTGATGTTCATCATCTGAGCGTATTGGTTATAAGTGAACGATACCGTCCGTTCTTTCCTTGTAG
GGTTTTCAATCGTGGGGTTGAGTAGTGCCACACAGCATAAAATTAGCTTGGTTTCATGCTCCGTTAAGTCATAGCGACTAATC
GCTAGTTCATTTGCTTTGAAAACAACTAATTCAGACATACATCTCAATTGGTCTAGGTGATTTTAATCACTATACCAATTGAG
ATGGGCTAGTCAATGATAATTACTAGTCCTTTTCCTTTGAGTTGTGGGTATCTGTAAATTCTGCTAGACCTTTGCTGGAAAAC
TTGTAAATTCTGCTAGACCCTCTGTAAATTCCGCTAGACCTTTGTGTGTTTTTTTTGTTTATATTCAAGTGGTTATAATTTAT
AGAATAAAGAAAGAATAAAAAAAGATAAAAAGAATAGATCCCAGCCCTGTGTATAACTCACTACTTTAGTCAGTTCCGCAGTA
TTACAAAAGGATGTCGCAAACGCTGTTTGCTCCTCTACAAAACAGACCTTAAAACCCTAAAGGCTTAAGTAGCACCCTCGCAA
GCTCGGTTGCGGCCGCAATCGGGCAAATCGCTGAATATTCCTTTTGTCTCCGACCATCAGGCACCTGAGTCGCTGTCTTTTTC
GTGACATTCAGTTCGCTGCGCTCACGGCTCTGGCAGTGAATGGGGGTAAATGGCACTACAGGCGCCTTTTATGGATTCATGCA
AGGAAACTACCCATAATACAAGAAAAGCCCGTCACGGGCTTCTCAGGGCGTTTTATGGCGGGTCTGCTATGTGGTGCTATCTG
ACTTTTTGCTGTTCAGCAGTTCCTGCCCTCTGATTTTCCAGTCTGACCACTTCGGATTATCCCGTGACAGGTCATTCAGACTG
GCTAATGCACCCAGTAAGGCAGCGGTATCATCAACGGGGTCTGACGCTCAGTGGAACGAAAACTCACGTTAAGGGATTTTGGT
CATGAGATTATCAAAAAGGATCTTCACCTAGATCCTTTTAAATTAAAAATGAAGTTTTAAATCAATCTAAAGTATATATGAGT
AAACTTGGTCTGACAGTTACGTTTCCACAACCAATTAACCAATTCTGATTTAGAAAAACTCATCGAGCATCAAATGAAACTGC
AATTTATTCATATCAGGATTATCAATACCATATTTTTGAAAAAGCCGTTTCTGTAATGAAGGAGAAAACTCACCGAGGCAGTT
CCATAGGATGGCAAGATCCTGGTATCGGTCTGCGATTCCGACTCGTCCAACATCAATACAACCTATTAATTTCCCCTCGTCAA
AAATAAGGTTATCAAGTGAGAAATCACCATGAGTGACGACTGAATCCGGTGAGAATGGCAAAAGCTTATGCATTTCTTTCCAG
ACTTGTTCAACAGGCCAGCCATTACGCTCGTCATCAAAATCACTCGCATCAACCAAACCGTTATTCATTCGTGATTGCGCCTG
AGCGAGACGAAATACGCGATCGCTGTTAAAAGGACAATTACAAACAGGAATCGAATGCAACCGGCGCAGGAACACTGCCAGCG
CATCAACAATATTTTCACCTGAATCAGGATATTCTTCTAATACCTGGAATGCTGTTTTCCCGGGGATCGCAGTGGTGAGTAAC
CATGCATCATCAGGAGTACGGATAAAATGCTTGATGGTCGGAAGAGGCATAAATTCCGTCAGCCAGTTTAGTCTGACCATCTC
ATCTGTAACATCATTGGCAACGCTACCTTTGCCATGTTTCAGAAACAACTCTGGCGCATCGGGCTTCCCATACAATCGATAGA
TTGTCGCACCTGATTGCCCGACATTATCGCGAGCCCATTTATACCCATATAAATCAGCATCCATGTTGGAATTTAATCGCGGC
CTCGAGCAAGACGTTTCCCGTTGAATATGGCTCATAACACCCCTTGTATTACTGTTTATGTAAGCAGACAGTTTTATTGTTCA
TGATGATATATTTTTATCTTGTGCAATGTAACATCAGAGATTTTGAGACACAACGTGGCTTTCCCTGCAGGATTTCGGAGGCC
TGCGTTATCCCCTGATTCTGTGGATAACCGTATTACCGCCTTTGAGTGAGCTGATACCGCTCGCCGCAGCCGAACGCCGACTA
GTGGATTTTATGGCTAGCTCAGTCCTAGGTACAATGCTAGCAGGCATATAACAGAGGGTTAATAACATGAAAGTTAAAGTACT
GTCCCTCCTGGTCCCAGCTCTGCTGGTAGCAGGCGCAGCAAACGCTGCTGAAGTTTACAACAAAGACGGCAACAAATTAGATC
TGTACGGTAAAGTAGACGGCCTGCACTATTTCTCTGACAACAAAGATGTAGATGGCGACCAGACCTACATGCGTCTTGGCTTC
AAAGGTGAAACTCAGGTTACTGACCAGCTGACCGGTTACGGCCAGTGGGAATATCAGATCCAGGGCAACAGCGCTGAAAACGA
AAACAACTCCTGGACCCGTGTGGCATTCGCAGGTCTGAAATTCCAGGATGTGGGTTCTTTCGACTACGGTCGTAACTACGGCG
TTGTTTATGACGTAACTTCCTGGACCGACGTACTGCCAGAATTCGGTGGTGACACCTACGGTTCTGACAACTTCATGCAGCAG
CGTGGTAACGGCTTCGCGACCTACCGTAACACTGACTTCTTCGGTCTGGTTGACGGCCTGAACTTTGCTGTTCAGTACCAGGG
TAAAAACGGCAACCCATCTGGTGAAGGCTTTACTAGTGGCGTAACTAACAACGGTCGTGACGCACTGCGTCAAAACGGCGACG
GCGTCGGCGGTTCTATCACTTATGATTACGAAGGTTTCGGTATCGGTGGTGCGATCTCCAGCTCCAAACGTACTGATGCTCAG
AACACCGCTGCTTACATCGGTAACGGCGACCGTGCTGAAACCTACACTGGTGGTCTGAAATACGACGCTAACAACATCTACCT

GGCTGCTCAGTACACCCAGACCTACAACGCAACTCGCGTAGGTTCCCTGGGTTGGGCGAACAAAGCACAGAACTTCGAAGCTG
TTGCTCAGTACCAGTTCGACTTCGGTCTGCGTCCGTCCCTGGCTTACCTGCAGTCTAAAGGTAAAAACCTGGGTCGTGGCTAC
GACGACGAAGATATCCTGAAATATGTTGATGTTGGTGCTACCTACTACTTCAACAAAAACATGTCCACCTACGTTGACTACAA
AATCAACCTGCTGGACGACAACCAGTTCACTCGTGACGCTGGCATCAACACTGATAACATCGTAGCTCTGGGTCTGGTTTACC
AGTTCTAATGACGCATCCTCACGATAATATCCGGGTAGGACGAACAATAAGGCCGCAAATCGCGGCCTTTTTTATTGATAACA
AAAGGACAGTTTTCCCTTTGATATGTAACGGTGAACAGTTGTTCTACTTTTGTTTGTTAGTCTTGATGCTTCACTGATAGATA
CAAGAGCCATAAGAACC

p1472

TCAGATCCTTCCGTATTTAGCCAGTATGTTCTCTAGTGTGGTTCGTTGTTTTTGCGTGAGCCATGAGAACGAACCATTGAGAT
CATACTTACTTTGCATGTCACTCAAAAATTTTGCCTCAAAACTGGTGAGCTGAATTTTTGCAGTTAAAGCATCGTGTAGTGTT
TTTCTTAGTCCGTTACGTAGGTAGGAATCTGATGTAATGGTTGTTGGTATTTTGTCACCATTCATTTTTATCTGGTTGTTCTC
AAGTTCGGTTACGAGATCCATTTGTCTATCTAGTTCAACTTGGAAAATCAACGTATCAGTCGGGCGGCCTCGCTTATCAACCA
CCAATTTCATATTGCTGTAAGTGTTTAAATCTTTACTTATTGGTTTCAAAACCCATTGGTTAAGCCTTTTAAACTCATGGTAG
TTATTTTCAAGCATTAACATGAACTTAAATTCATCAAGGCTAATCTCTATATTTGCCTTGTGAGTTTTCTTTTGTGTTAGTTC
TTTTAATAACCACTCATAAATCCTCATAGAGTATTTGTTTTCAAAAGACTTAACATGTTCCAGATTATATTTTATGAATTTTT
TTAACTGGAAAAGATAAGGCAATATCTCTTCACTAAAAACTAATTCTAATTTTTCGCTTGAGAACTTGGCATAGTTTGTCCAC
TGGAAAATCTCAAAGCCTTTAACCAAAGGATTCCTGATTTCCACAGTTCTCGTCATCAGCTCTCTGGTTGCTTTAGCTAATAC
ACCATAAGCATTTTCCCTACTGATGTTCATCATCTGAGCGTATTGGTTATAAGTGAACGATACCGTCCGTTCTTTCCTTGTAG
GGTTTTCAATCGTGGGGTTGAGTAGTGCCACACAGCATAAAATTAGCTTGGTTTCATGCTCCGTTAAGTCATAGCGACTAATC
GCTAGTTCATTTGCTTTGAAAACAACTAATTCAGACATACATCTCAATTGGTCTAGGTGATTTTAATCACTATACCAATTGAG
ATGGGCTAGTCAATGATAATTACTAGTCCTTTTCCTTTGAGTTGTGGGTATCTGTAAATTCTGCTAGACCTTTGCTGGAAAAC
TTGTAAATTCTGCTAGACCCTCTGTAAATTCCGCTAGACCTTTGTGTGTTTTTTTTGTTTATATTCAAGTGGTTATAATTTAT
AGAATAAAGAAAGAATAAAAAAAGATAAAAAGAATAGATCCCAGCCCTGTGTATAACTCACTACTTTAGTCAGTTCCGCAGTA
TTACAAAAGGATGTCGCAAACGCTGTTTGCTCCTCTACAAAACAGACCTTAAAACCCTAAAGGCTTAAGTAGCACCCTCGCAA
GCTCGGTTGCGGCCGCAATCGGGCAAATCGCTGAATATTCCTTTTGTCTCCGACCATCAGGCACCTGAGTCGCTGTCTTTTTC
GTGACATTCAGTTCGCTGCGCTCACGGCTCTGGCAGTGAATGGGGGTAAATGGCACTACAGGCGCCTTTTATGGATTCATGCA
AGGAAACTACCCATAATACAAGAAAAGCCCGTCACGGGCTTCTCAGGGCGTTTTATGGCGGGTCTGCTATGTGGTGCTATCTG
ACTTTTTGCTGTTCAGCAGTTCCTGCCCTCTGATTTTCCAGTCTGACCACTTCGGATTATCCCGTGACAGGTCATTCAGACTG
GCTAATGCACCCAGTAAGGCAGCGGTATCATCAACGGGGTCTGACGCTCAGTGGAACGAAAACTCACGTTAAGGGATTTTGGT
CATGAGATTATCAAAAAGGATCTTCACCTAGATCCTTTTAAATTAAAAATGAAGTTTTAAATCAATCTAAAGTATATATGAGT
AAACTTGGTCTGACAGTTACGTTTCCACAACCAATTAACCAATTCTGATTTAGAAAAACTCATCGAGCATCAAATGAAACTGC
AATTTATTCATATCAGGATTATCAATACCATATTTTTGAAAAAGCCGTTTCTGTAATGAAGGAGAAAACTCACCGAGGCAGTT
CCATAGGATGGCAAGATCCTGGTATCGGTCTGCGATTCCGACTCGTCCAACATCAATACAACCTATTAATTTCCCCTCGTCAA
AAATAAGGTTATCAAGTGAGAAATCACCATGAGTGACGACTGAATCCGGTGAGAATGGCAAAAGCTTATGCATTTCTTTCCAG
ACTTGTTCAACAGGCCAGCCATTACGCTCGTCATCAAAATCACTCGCATCAACCAAACCGTTATTCATTCGTGATTGCGCCTG
AGCGAGACGAAATACGCGATCGCTGTTAAAAGGACAATTACAAACAGGAATCGAATGCAACCGGCGCAGGAACACTGCCAGCG
CATCAACAATATTTTCACCTGAATCAGGATATTCTTCTAATACCTGGAATGCTGTTTTCCCGGGGATCGCAGTGGTGAGTAAC
CATGCATCATCAGGAGTACGGATAAAATGCTTGATGGTCGGAAGAGGCATAAATTCCGTCAGCCAGTTTAGTCTGACCATCTC
ATCTGTAACATCATTGGCAACGCTACCTTTGCCATGTTTCAGAAACAACTCTGGCGCATCGGGCTTCCCATACAATCGATAGA
TTGTCGCACCTGATTGCCCGACATTATCGCGAGCCCATTTATACCCATATAAATCAGCATCCATGTTGGAATTTAATCGCGGC
CTCGAGCAAGACGTTTCCCGTTGAATATGGCTCATAACACCCCTTGTATTACTGTTTATGTAAGCAGACAGTTTTATTGTTCA
TGATGATATATTTTTATCTTGTGCAATGTAACATCAGAGATTTTGAGACACAACGTGGCTTTCCCTGCAGGATTTCGGAGGCC
TGCGTTATCCCCTGATTCTGTGGATAACCGTATTACCGCCTTTGAGTGAGCTGATACCGCTCGCCGCAGCCGAACGCCGACTA
GTGGATTTTATGGCTAGCTCAGTCCTAGGTACAATGCTAGCAGGCATATAACAGAGGGTTAATAACATGAAAGTTAAAGTACT
GTCCCTCCTGGTCCCAGCTCTGCTGGTAGCAGGCGCAGCAAACGCTGCTGAAGTTTACAACAAAGACGGCAACAAATTAGATC
TGTACGGTAAAGTAGACGGCCTGCACTATTTCTCTGACGACAAGTCTGTAGATGGCGACCAGACCTACATGCGTCTTGGCTTC
AAAGGTGAAACTCAGGTTACTGACCAGCTGACCGGTTACGGCCAGTGGGAATATCAGATCCAGGGCAACAGCGCTGAAAACGA
AAACAACTCCTGGACCCGTGTGGCATTCGCAGGTCTGAAATTCCAGGATGTAGGTTCTTTCGACTACGGTCGTAACTACGGCG
TAGTTTACGACGTAACTTCCTGGACCGACGTTCTGCCAGAATTCGGTGGTGACACCTACGGTTCTGACAACTTCATGCAGCAG
CGTGGTAACGGTTTCGCGACCTACCGTAACACTGACTTCTTCGGTCTGGTTGACGGCCTGAACTTTGCTGTTCAGTACCAGGG
CAAAAACGGTAGCGTAAGCGGCGAAGGCATGACTAACAACGGTCGTGAAGCACTGCGTCAGAACGGCGACGGCGTTGGCGGAT
CTATCACTTATGATTACGAAGGCTTCGGTATCGGTGCTGCAGTTTCCAGCTCCAAACGTACTGATGATCAGAACAGCCCGCTG
TACATCGGTAACGGCGACCGTGCTGAAACCTACACTGGTGGTCTGAAATACGACGCTAACAACATCTATCTGGCTGCTCAGTA
CACCCAGACCTACAACGCAACTCGCGTAGGTTCCCTGGGTTGGGCGAACAAAGCACAGAACTTCGAAGCTGTTGCTCAGTACC
AGTTCGACTTCGGTCTGCGTCCGTCCCTGGCTTACCTGCAGTCTAAAGGTAAAAACCTGGGTGTCATCAATGGTCGTAACTAC
GACGACGAAGATATCCTGAAATATGTTGATGTTGGCGCGACCTACTACTTCAACAAAAACATGTCCACCTATGTTGACTACAA
AATCAACCTGCTGGACGACAACCAGTTCACTCGTGACGCTGGCATCAACACTGATAACATCGTAGCTCTGGGTCTGGTTTACC
AGTTCTAATGACGCATCCTCACGATAATATCCGGGTAGGACGAACAATAAGGCCGCAAATCGCGGCCTTTTTTATTGATAACA

AAAGGACAGTTTTCCCTTTGATATGTAACGGTGAACAGTTGTTCTACTTTTGTTTGTTAGTCTTGATGCTTCACTGATAGATA
CAAGAGCCATAAGAACC

p1806

TCAGATCCTTCCGTATTTAGCCAGTATGTTCTCTAGTGTGGTTCGTTGTTTTTGCGTGAGCCATGAGAACGAACCATTGAGAT
CATACTTACTTTGCATGTCACTCAAAAATTTTGCCTCAAAACTGGTGAGCTGAATTTTTGCAGTTAAAGCATCGTGTAGTGTT
TTTCTTAGTCCGTTACGTAGGTAGGAATCTGATGTAATGGTTGTTGGTATTTTGTCACCATTCATTTTTATCTGGTTGTTCTC
AAGTTCGGTTACGAGATCCATTTGTCTATCTAGTTCAACTTGGAAAATCAACGTATCAGTCGGGCGGCCTCGCTTATCAACCA
CCAATTTCATATTGCTGTAAGTGTTTAAATCTTTACTTATTGGTTTCAAAACCCATTGGTTAAGCCTTTTAAACTCATGGTAG
TTATTTTCAAGCATTAACATGAACTTAAATTCATCAAGGCTAATCTCTATATTTGCCTTGTGAGTTTTCTTTTGTGTTAGTTC
TTTTAATAACCACTCATAAATCCTCATAGAGTATTTGTTTTCAAAAGACTTAACATGTTCCAGATTATATTTTATGAATTTTT
TTAACTGGAAAAGATAAGGCAATATCTCTTCACTAAAAACTAATTCTAATTTTTCGCTTGAGAACTTGGCATAGTTTGTCCAC
TGGAAAATCTCAAAGCCTTTAACCAAAGGATTCCTGATTTCCACAGTTCTCGTCATCAGCTCTCTGGTTGCTTTAGCTAATAC
ACCATAAGCATTTTCCCTACTGATGTTCATCATCTGAGCGTATTGGTTATAAGTGAACGATACCGTCCGTTCTTTCCTTGTAG
GGTTTTCAATCGTGGGGTTGAGTAGTGCCACACAGCATAAAATTAGCTTGGTTTCATGCTCCGTTAAGTCATAGCGACTAATC
GCTAGTTCATTTGCTTTGAAAACAACTAATTCAGACATACATCTCAATTGGTCTAGGTGATTTTAATCACTATACCAATTGAG
ATGGGCTAGTCAATGATAATTACTAGTCCTTTTCCTTTGAGTTGTGGGTATCTGTAAATTCTGCTAGACCTTTGCTGGAAAAC
TTGTAAATTCTGCTAGACCCTCTGTAAATTCCGCTAGACCTTTGTGTGTTTTTTTTGTTTATATTCAAGTGGTTATAATTTAT
AGAATAAAGAAAGAATAAAAAAAGATAAAAAGAATAGATCCCAGCCCTGTGTATAACTCACTACTTTAGTCAGTTCCGCAGTA
TTACAAAAGGATGTCGCAAACGCTGTTTGCTCCTCTACAAAACAGACCTTAAAACCCTAAAGGCTTAAGTAGCACCCTCGCAA
GCTCGGTTGCGGCCGCAATCGGGCAAATCGCTGAATATTCCTTTTGTCTCCGACCATCAGGCACCTGAGTCGCTGTCTTTTTC
GTGACATTCAGTTCGCTGCGCTCACGGCTCTGGCAGTGAATGGGGGTAAATGGCACTACAGGCGCCTTTTATGGATTCATGCA
AGGAAACTACCCATAATACAAGAAAAGCCCGTCACGGGCTTCTCAGGGCGTTTTATGGCGGGTCTGCTATGTGGTGCTATCTG
ACTTTTTGCTGTTCAGCAGTTCCTGCCCTCTGATTTTCCAGTCTGACCACTTCGGATTATCCCGTGACAGGTCATTCAGACTG
GCTAATGCACCCAGTAAGGCAGCGGTATCATCAACGGGGTCTGACGCTCAGTGGAACGAAAACTCACGTTAAGGGATTTTGGT
CATGAGATTATCAAAAAGGATCTTCACCTAGATCCTTTTAAATTAAAAATGAAGTTTTAAATCAATCTAAAGTATATATGAGT
AAACTTGGTCTGACAGTTACGTTTCCACAACCAATTAACCAATTCTGATTTAGAAAAACTCATCGAGCATCAAATGAAACTGC
AATTTATTCATATCAGGATTATCAATACCATATTTTTGAAAAAGCCGTTTCTGTAATGAAGGAGAAAACTCACCGAGGCAGTT
CCATAGGATGGCAAGATCCTGGTATCGGTCTGCGATTCCGACTCGTCCAACATCAATACAACCTATTAATTTCCCCTCGTCAA
AAATAAGGTTATCAAGTGAGAAATCACCATGAGTGACGACTGAATCCGGTGAGAATGGCAAAAGCTTATGCATTTCTTTCCAG
ACTTGTTCAACAGGCCAGCCATTACGCTCGTCATCAAAATCACTCGCATCAACCAAACCGTTATTCATTCGTGATTGCGCCTG
AGCGAGACGAAATACGCGATCGCTGTTAAAAGGACAATTACAAACAGGAATCGAATGCAACCGGCGCAGGAACACTGCCAGCG
CATCAACAATATTTTCACCTGAATCAGGATATTCTTCTAATACCTGGAATGCTGTTTTCCCGGGGATCGCAGTGGTGAGTAAC
CATGCATCATCAGGAGTACGGATAAAATGCTTGATGGTCGGAAGAGGCATAAATTCCGTCAGCCAGTTTAGTCTGACCATCTC
ATCTGTAACATCATTGGCAACGCTACCTTTGCCATGTTTCAGAAACAACTCTGGCGCATCGGGCTTCCCATACAATCGATAGA
TTGTCGCACCTGATTGCCCGACATTATCGCGAGCCCATTTATACCCATATAAATCAGCATCCATGTTGGAATTTAATCGCGGC
CTCGAGCAAGACGTTTCCCGTTGAATATGGCTCATAACACCCCTTGTATTACTGTTTATGTAAGCAGACAGTTTTATTGTTCA
TGATGATATATTTTTATCTTGTGCAATGTAACATCAGAGATTTTGAGACACAACGTGGCTTTCCCTGCAGGATTTCGGAGGCC
TGCGTTATCCCCTGATTCTGTGGATAACCGTATTACCGCCTTTGAGTGAGCTGATACCGCTCGCCGCAGCCGAACGCCGACTA
GTGGATTTTACGGCTAGCTCAGTCCTAGGTACAATGCTAGCgaattcattaaagaggagaaaggtacccATGGCACGTACCCC
GAGCCGTAGCAGCATTGGTAGCCTGCGTAGTCCGCATACCCATAAAGCAATTCTGACCAGCACCATTGAAATCCTGAAAGAAT
GTGGTTATAGCGGTCTGAGCATTGAAAGCGTTGCACGTCGTGCCGGTGCAAGCAAACCGACCATTTATCGTTGGTGGACCAAT
AAAGCAGCACTGATTGCCGAAGTGTATGAAAATGAAAGCGAACAGGTGCGTAAATTTCCGGATCTGGGTAGCTTTAAAGCCGA
TCTGGATTTTCTGCTGCGTAATCTGTGGAAAGTTTGGCGTGAAACCATTTGTGGTGAAGCATTTCGTTGTGTTATTGCAGAAG
CACAGCTGGACCCTGCAACCCTGACCCAGCTGAAAGATCAGTTTATGGAACGTCGTCGTGAGATGCCGAAAAAACTGGTTGAA
AATGCCATTAGCAATGGTGAACTGCCGAAAGATACCAATCGTGAACTGCTGCTGGATATGATTTTTGGTTTTTGTTGGTATCG
CCTGCTGACCGAACAGCTGACCGTTGAACAGGATATTGAAGAATTTACCTTCCTGCTaATTAATGGTGTTTGTCCGGGTACAC
AGCGTTAACTAGGGCCCATACCCCCAATTATTGAAGGCCGCTAACGCGGCCTTTTTTTGTTTCTGGTCTGCCCGACGTACGGT
GAAtctgattcgttaccaattgacATGATACGAAACGTACCGTATCGTTAAGGTTACTAGattaaagaggagaaatactagAT
GGCAGTAAAGATTTCAGGAGTCCTGAAAGACGGCACAGGAAAACCGGTACAGAACTGCACCATTCAGCTGAAAGCCAGACGTA
ACAGCACCACGGTGGTGGTGAACACGGTGGGCTCAGAGAATCCGGATGAAGCCGGGCGTTACAGCATGGATGTGGAGTACGGT
CAGTACAGTGTCATCCTGCAGGTTGACGGTTTTCCACCATCGCACGCCGGGACCATCACCGTGTATGAAGATTCACAACCGGG
GACGCTGAATGATTTTCTCTGTGCCATGACGGAGGATGATGCCCGGCCGGAGGTGCTGCGTCGTCTTGAACTGATGGTGGAAG
AGGTGGCGCGTAACGCGTCCGTGGTGGCACAGAGTACGGCAGACGCGAAGAAATCAGCCGGCGATGCCAGTGCATCAGCTGCT
CAGGTCGCGGCCCTTGTGACTGATGCAACTGACTCAGCACGCGCCGCCAGCACGTCCGCCGGACAGGCTGCATCGTCAGCTCA
GGAAGCGTCCTCCGGCGCAGAAGCGGCATCAGCAAAGGCCACTGAAGCGGAAAAAAGTGCCGCAGCCGCAGAGTCCTCAAAAA
ACGCGGCGGCCACCAGTGCCGGTGCGGCGAAAACGTCAGAAACGAATGCTGCAGCGTCACAACAATCAGCCGCCACGTCTGCC
TCCACCGCGGCCACGAAAGCGTCAGAGGCCGCCACTTCAGCACGAGATGCGGTGGCCTCAAAAGAGGCAGCAAAATCATCAGA
AACGAACGCATCATCAAGTGCCGGTCGTGCAGCTTCCTCGGCAACGGCGGCAGAAAATTCTGCCAGGGCGGCAAAAACGTCCG
AGACGAATGCCAGGTCATCTGAAACAGCAGCGGAACGGAGCGCCTCTGCCGCGGCAGACGCAAAAACAGCGGCGGCGGGGAGT
GCGTCAACGGCATCCACGAAGGCGACAGAGGCTGCGGGAAGTGCGGTATCAGCATCGCAGAGCAAAAGTGCGGCAGAAGCGGC

GGCAATACGTGCAAAAAATTCGGCAAAACGTGCAGAAGATATAGCTTCAGCTGTCGCGCTTGAGGATGCGGACACAACGAGAA
AGGGGATAGTGCAGCTCAGCAGTGCAACCAACAGCACGTCTGAAACGCTTGCTGCAACGCCAAAGGCGGTTAAGGTGGTAATG
GATGAGACTAATCGTAAGGCACCTCTGGACAGTCCGGCACTGACCGGAACGCCAACAGCACCAACCGCGCTCAGGGGAACAAA
CAATACCCAGATTGCGAACACCGCTTTTGTACTGGCCGCGATTGCAGATGTTATCGACGCGTCACCTGACGCACTGAATACGC
TGAATGAACTGGCCGCAGCGCTCGGGAATGATCCAGATTTTGCTACCACCATGACTAACGCGCTTGCGGGTAAACAACCGAAG
AATGCGACACTGACGGCGCTGGCAGGGCTTTCCACGGCGAAAAATAAATTACCGTATTTTGCGGAAAATGATGCCGCCAGCCT
GACTGAACTGACTCAGGTTGGCAGGGATATTCTGGCAAAAAATTCCGTTGCAGATGTTCTTGAATACCTTGGGGCCGGTGAGA
ATTCGGGATCAGCAGCTGATGTATTAATAGAATTAGCGAAACCAACAGGTGCCGATTTAGTTTATTGTGGAAATTCTCCTGTA
TCTTTAATAATTAGAGGTAGTATATTTAAATACTTAAATGAAGTAGACAGGAGTACGCTGTTAAATGTAGTTGGGGCAGAAGT
TGTTGCGGATTATGCTCTACAAGCTGCTATCGATGATGGTGTTACCATTCTTGAATGGCATGCAGTTCCTGGGGTATATGTTT
TGGGTAAGGATTTAGTTACGTTACCTGTTGGTTTCTCATTTGAGGGAGAATCGAGGAGAACGTATACAGCATCATCAGATGCT
TCATTTAATAATGTTGGCACGGTTCTCCGTTTATTTAATGGTGCATCAGCAATTTTCAAAATGACATCAAGACACTCATTTAG
AAGGGTGGTTTTCGATGGTCGCAACAAGTCCGTGCGCTTTATGCAAGGGTATGACCAAACACAATGGTGCAGGTTTTATGACT
GCGGAGTACACCGTTGGTATATTGGTATTGGTGGCTCAAGTCCTAATGGTTACTCTGCTACTTTAATCTTTTCTGGTGGCACC
ATTTCCAGTAATACGATTGGCGTCAAAAACGTCATTGATTCACTGTTTTTAGGGGCCACAATAAATGCCAATGACACTGATGG
TGTTCAGCTTCTGACCGGAGCGAATAACAATGCGTTTATCGGCGTCAGAAACGAATGGAATAATGGTGATAACTATTATGGCT
ATGGGTGTAAGAGAATACTGATTCAGGGTGAACTGATAGACAGGGCTGGCAAACGAGCGGTTGCCGCCGTTGGTGGAGCCCAG
TTTGTTCTTTCTGGTGTAGCTTTACAACGTTCGGGTAGACTTGCTACTGAGGGTACTGTAGATGACTCTCATTTTTATCTGGA
AGGGGATACGTCTTCAATAGTAGTAACCCCAACCTACACTACTACTGGTGCTAACGATGATGGGTCTGGTAGATCTTCCCCAA
CCTATATATTAGCCACAGGTGGTAGCAACTCTGATGCGAAAAGTTTTATTGCTTCAGCATCCAATCTTTCAGGTTATACAGGC
ACCTCATGGCTGCGTTCTGGTGTAATTGCCTCACTTTCAGTGCAAGGCTGCCTGGGAGTTGAGGATGTTAAAAACTTCGGACT
TAGACGTATTAGTAACGGTGTACAGTATCTTGGCGATGCTGTGTCTGGGCTGGCTTTGTCTGGTGCAGGTAATACTGCAACTA
TGGTATTTACAACTACTCCACAAGAGCTTTCTCGATACTCGTCAGAGCTTCTTGTGCGTACTCTTGAAATTACGGCAAGAAAT
AATACCTCTACAGGGTCGGTTGCATATTACTCTGTTAACTTAATTATCTCACGTGAATATGCATCGGCTGCTATTGCGGTGGA
TACGGCTTCTGTTAGAACTTTTGCCACTGTTAGTGGTGGAACGTGGGGCATTACCTCTGCTAGTCCAACCGGGGTTTCGCTTT
CTTTTGCTATCTCTTCTGATGGTAAGACCTTAACCGTAACATTAACAGCGATAGACTCTGCCAGCAGAGTTATCAGTGCAAAA
CTAAGGGCATAAtATGGAACAAAGTGAAAATGATGCATTTGATGCATGGTATCAAGAATTATGTATTATTCTGGCACATAATG
GAAAATGTGCACCCTACAAAATGGCATGGTATGAATTTTATGAGCAAGGACTATCTGTGCAAGATGCAGCGAATCAAGGACCT
AGTGAAATAATTAATTAGtaaAACTATATTGTGAGGCTTGCATAATGGCATTCAGAATGAGTGAACAACCACGGACCATAAAA
ATTTATAATCTGCTGGCCGGAACTAATGAATTTATTGGTGAAGGTGACGCATATATTCCGCCTCATACCGGTCTGCCTGCAAA
CAGTACCGATATTGCACCGCCAGATATTCCGGCTGGCTTTGTGGCTGTTTTCAACAGTGATGAGGCATCGTGGCATCTCGTTG
AAGACCATCGGGGTAAAACCGTCTATGACGTGGCTTCCGGCGACGCGTTATTTATTTCTGAACTCGGTCCGTTACCGGAAAAT
TTTACCTGGTTATCGCCGGGAGGGGAATATCAGAAGTGGAACGGCACAGCCTGGGTGAAGGATACGGAAGCAGAAAAACTGTT
CCGGATCCGGGAGGCGGAAGAAACAAAAAAAAGCCTGATGCAGGTAGCCAGTGAGCATATTGCGCCGCTTCAGGATGCTGCAG
ATCTGGAAATTGCAACGAAGGAAGAAACCTCGTTGCTGGAAGCCTGGAAGAAGTATCGGGTGTTGCTGAACCGTGTTGATACA
TCAACTGCACCTGATATTGAGTGGCCTGCTGTCCCTGTTATGGAGTAAtgacgcatcctcacgataatatccgggtagGACGA
ACAATAAGGCCGCAAATCGCGGCCTTTTTTATTGATAACAAAAGGACAGTTTTCCCTTTGATATGTAACGGTGAACAGTTGTT
CTACTTTTGTTTGTTAGTCTTGATGCTTCACTGATAGATACAAGAGCCATAAGAACC

p2058

TCAGATCCTTCCGTATTTAGCCAGTATGTTCTCTAGTGTGGTTCGTTGTTTTTGCGTGAGCCATGAGAACGAACCATTGAGAT
CATACTTACTTTGCATGTCACTCAAAAATTTTGCCTCAAAACTGGTGAGCTGAATTTTTGCAGTTAAAGCATCGTGTAGTGTT
TTTCTTAGTCCGTTACGTAGGTAGGAATCTGATGTAATGGTTGTTGGTATTTTGTCACCATTCATTTTTATCTGGTTGTTCTC
AAGTTCGGTTACGAGATCCATTTGTCTATCTAGTTCAACTTGGAAAATCAACGTATCAGTCGGGCGGCCTCGCTTATCAACCA
CCAATTTCATATTGCTGTAAGTGTTTAAATCTTTACTTATTGGTTTCAAAACCCATTGGTTAAGCCTTTTAAACTCATGGTAG
TTATTTTCAAGCATTAACATGAACTTAAATTCATCAAGGCTAATCTCTATATTTGCCTTGTGAGTTTTCTTTTGTGTTAGTTC
TTTTAATAACCACTCATAAATCCTCATAGAGTATTTGTTTTCAAAAGACTTAACATGTTCCAGATTATATTTTATGAATTTTT
TTAACTGGAAAAGATAAGGCAATATCTCTTCACTAAAAACTAATTCTAATTTTTCGCTTGAGAACTTGGCATAGTTTGTCCAC
TGGAAAATCTCAAAGCCTTTAACCAAAGGATTCCTGATTTCCACAGTTCTCGTCATCAGCTCTCTGGTTGCTTTAGCTAATAC
ACCATAAGCATTTTCCCTACTGATGTTCATCATCTGAGCGTATTGGTTATAAGTGAACGATACCGTCCGTTCTTTCCTTGTAG
GGTTTTCAATCGTGGGGTTGAGTAGTGCCACACAGCATAAAATTAGCTTGGTTTCATGCTCCGTTAAGTCATAGCGACTAATC
GCTAGTTCATTTGCTTTGAAAACAACTAATTCAGACATACATCTCAATTGGTCTAGGTGATTTTAATCACTATACCAATTGAG
ATGGGCTAGTCAATGATAATTACTAGTCCTTTTCCTTTGAGTTGTGGGTATCTGTAAATTCTGCTAGACCTTTGCTGGAAAAC
TTGTAAATTCTGCTAGACCCTCTGTAAATTCCGCTAGACCTTTGTGTGTTTTTTTTGTTTATATTCAAGTGGTTATAATTTAT
AGAATAAAGAAAGAATAAAAAAAGATAAAAAGAATAGATCCCAGCCCTGTGTATAACTCACTACTTTAGTCAGTTCCGCAGTA
TTACAAAAGGATGTCGCAAACGCTGTTTGCTCCTCTACAAAACAGACCTTAAAACCCTAAAGGCTTAAGTAGCACCCTCGCAA
GCTCGGTTGCGGCCGCAATCGGGCAAATCGCTGAATATTCCTTTTGTCTCCGACCATCAGGCACCTGAGTCGCTGTCTTTTTC
GTGACATTCAGTTCGCTGCGCTCACGGCTCTGGCAGTGAATGGGGGTAAATGGCACTACAGGCGCCTTTTATGGATTCATGCA
AGGAAACTACCCATAATACAAGAAAAGCCCGTCACGGGCTTCTCAGGGCGTTTTATGGCGGGTCTGCTATGTGGTGCTATCTG
ACTTTTTGCTGTTCAGCAGTTCCTGCCCTCTGATTTTCCAGTCTGACCACTTCGGATTATCCCGTGACAGGTCATTCAGACTG
GCTAATGCACCCAGTAAGGCAGCGGTATCATCAACGGGGTCTGACGCTCAGTGGAACGAAAACTCACGTTAAGGGATTTTGGT

CATGAGATTATCAAAAAGGATCTTCACCTAGATCCTTTTAAATTAAAAATGAAGTTTTAAATCAATCTAAAGTATATATGAGT
AAACTTGGTCTGACAGTTACGTTTCCACAACCAATTAACCAATTCTGATTTAGAAAAACTCATCGAGCATCAAATGAAACTGC
AATTTATTCATATCAGGATTATCAATACCATATTTTTGAAAAAGCCGTTTCTGTAATGAAGGAGAAAACTCACCGAGGCAGTT
CCATAGGATGGCAAGATCCTGGTATCGGTCTGCGATTCCGACTCGTCCAACATCAATACAACCTATTAATTTCCCCTCGTCAA
AAATAAGGTTATCAAGTGAGAAATCACCATGAGTGACGACTGAATCCGGTGAGAATGGCAAAAGCTTATGCATTTCTTTCCAG
ACTTGTTCAACAGGCCAGCCATTACGCTCGTCATCAAAATCACTCGCATCAACCAAACCGTTATTCATTCGTGATTGCGCCTG
AGCGAGACGAAATACGCGATCGCTGTTAAAAGGACAATTACAAACAGGAATCGAATGCAACCGGCGCAGGAACACTGCCAGCG
CATCAACAATATTTTCACCTGAATCAGGATATTCTTCTAATACCTGGAATGCTGTTTTCCCGGGGATCGCAGTGGTGAGTAAC
CATGCATCATCAGGAGTACGGATAAAATGCTTGATGGTCGGAAGAGGCATAAATTCCGTCAGCCAGTTTAGTCTGACCATCTC
ATCTGTAACATCATTGGCAACGCTACCTTTGCCATGTTTCAGAAACAACTCTGGCGCATCGGGCTTCCCATACAATCGATAGA
TTGTCGCACCTGATTGCCCGACATTATCGCGAGCCCATTTATACCCATATAAATCAGCATCCATGTTGGAATTTAATCGCGGC
CTCGAGCAAGACGTTTCCCGTTGAATATGGCTCATAACACCCCTTGTATTACTGTTTATGTAAGCAGACAGTTTTATTGTTCA
TGATGATATATTTTTATCTTGTGCAATGTAACATCAGAGATTTTGAGACACAACGTGGCTTTCCCTGCAGGATTTCGGAGGCC
TGCGTTATCCCCTGATTCTGTGGATAACCGTATTACCGCCTTTGAGTGAGCTGATACCGCTCGCCGCAGCCGAACGCCGACTA
GTGGATTTTACGGCTAGCTCAGTCCTAGGTACAATGCTAGCGAATTCATTAAAGAGGAGAAAGGTACCCATGGCACGTACCCC
GAGCCGTAGCAGCATTGGTAGCCTGCGTAGTCCGCATACCCATAAAGCAATTCTGACCAGCACCATTGAAATCCTGAAAGAAT
GTGGTTATAGCGGTCTGAGCATTGAAAGCGTTGCACGTCGTGCCGGTGCAAGCAAACCGACCATTTATCGTTGGTGGACCAAT
AAAGCAGCACTGATTGCCGAAGTGTATGAAAATGAAAGCGAACAGGTGCGTAAATTTCCGGATCTGGGTAGCTTTAAAGCCGA
TCTGGATTTTCTGCTGCGTAATCTGTGGAAAGTTTGGCGTGAAACCATTTGTGGTGAAGCATTTCGTTGTGTTATTGCAGAAG
CACAGCTGGACCCTGCAACCCTGACCCAGCTGAAAGATCAGTTTATGGAACGTCGTCGTGAGATGCCGAAAAAACTGGTTGAA
AATGCCATTAGCAATGGTGAACTGCCGAAAGATACCAATCGTGAACTGCTGCTGGATATGATTTTTGGTTTTTGTTGGTATCG
CCTGCTGACCGAACAGCTGACCGTTGAACAGGATATTGAAGAATTTACCTTCCTGCTAATTAATGGTGTTTGTCCGGGTACAC
AGCGTTAACTAGGGCCCATACCCCCAATTATTGAAGGCCGCTAACGCGGCCTTTTTTTGTTTCTGGTCTGCCCGACGTACGGT
GAATCTGATTCGTTACCAATTGACATGATACGAAACGTACCGTATCGTTAAGGTTACTAGATTAAAGAGGAGAAATACTAGAT
GGCAGTAAAGATTTCAGGAGTCCTGAAAGACGGCACAGGAAAACCGGTACAGAACTGCACCATTCAGCTGAAAGCCAGACGTA
ACAGCACCACGGTGGTGGTGAACACGGTGGGCTCAGAGAATCCGGATGAAGCCGGGCGTTACAGCATGGATGTGGAGTACGGT
CAGTACAGTGTCATCCTGCAGGTTGACGGTTTTCCACCATCGCACGCCGGGACCATCACCGTGTATGAAGATTCACAACCGGG
GACGCTGAATGATTTTCTCTGTGCCATGACGGAGGATGATGCCCGGCCGGAGGTGCTGCGTCGTCTTGAACTGATGGTGGAAG
AGGTGGCGCGTAACGCGTCCGTGGTGGCACAGAGTACGGCAGACGCGAAGAAATCAGCCGGCGATGCCAGTGCATCAGCTGCT
CAGGTCGCGGCCCTTGTGACTGATGCAACTGACTCAGCACGCGCCGCCAGCACGTCCGCCGGACAGGCTGCATCGTCAGCTCA
GGAAGCGTCCTCCGGCGCAGAAGCGGCATCAGCAAAGGCCACTGAAGCGGAAAAAAGTGCCGCAGCCGCAGAGTCCTCAAAAA
ACGCGGCGGCCACCAGTGCCGGTGCGGCGAAAACGTCAGAAACGAATGCTGCAGCGTCACAACAATCAGCCGCCACGTCTGCC
TCCACCGCGGCCACGAAAGCGTCAGAGGCCGCCACTTCAGCACGAGATGCGGTGGCCTCAAAAGAGGCAGCAAAATCATCAGA
AACGAACGCATCATCAAGTGCCGGTCGTGCAGCTTCCTCGGCAACGGCGGCAGAAAATTCTGCCAGGGCGGCAAAAACGTCCG
AGACGAATGCCAGGTCATCTGAAACAGCAGCGGAACGGAGCGCCTCTGCCGCGGCAGACGCAAAAACAGCGGCGGCGGGGAGT
GCGTCAACGGCATCCACGAAGGCGACAGAGGCTGCGGGAAGTGCGGTATCAGCATCGCAGAGCAAAAGTGCGGCAGAAGCGGC
GGCAATACGTGCAAAAAATTCGGCAAAACGTGCAGAAGATATAGCTTCAGCTGTCGCGCTTGAGGATGCGGACACAACGAGAA
AGGGGATAGTGCAGCTCAGCAGTGCAACCAACAGCACGTCTGAAACGCTTGCTGCAACGCCAAAGGCGGTTAAGGTGGTAATG
GATGAGACTAATCGTAAGGCACCTCTGGACAGTCCGGCACTGACCGGAACGCCAACAGCACCAACCGCGCTCAGGGGAACAAA
CAATACCCAGATTGCGAACACCGCTTTTGTACTGGCCGCGATTGCAGATGTTATCGACGCGTCACCTGACGCACTGAATACGC
TGAATGAACTGGCCGCAGCGCTCGGGAATGATCCAGATTTTGCTACCACCATGACTAACGCGCTTGCGGGTAAACAACCGAAG
AATGCGACACTGACGGCGCTGGCAGGGCTTTCCACGGCGAAAAATAAATTACCGTATTTTGCGGAAAATGATGCCGCCAGCCT
GACTGAACTGACTCAGGTTGGCAGGGATATTCTGGCAAAAAATTCCGTTGCAGATGTTCTTGAATACCTTGGGGCCGGTGAGA
ATTCGGACGCAGCACTGCGCTCTCAAATCAGCAACCCAGAAGGCGCGATTCTGTACCCGGAACTGCAGATGGCGCGCTGGCGT
GATGAAGGCGACGTTCGTGGTTGGGGTGCCAAAGGTGATGGTGTAACCGACTCCACTGAAAACATCGCAGCATCCTTGAACTC
TCAGAAAGCAGTTGTTGCCAGCGAAGGCGTTTTCAGTTCTTCTGGCATCAACTCCAACTACTGTAACCTGGATGGTCGCGGAT
CCGGTGTGCTCAGCCACCGTAGCTCTACTGGTAATTACCTGGTGTTTAACAATCCGCGTACTGGTCGTCTGAGCAATATCACT
GTTGAATCTAACAAAGCGACCGATACCACTCAGGGCCAACAGGTGTCCCTGGCAGGTGGCAGTGACGTGACCGTGTCAGATGT
CAACTTCTCCAACGTGAAAGGCACTGGTTTTAGCCTGATTGCCTACCCAAACGATGCTCCGCCGGATGGCCTGATGATCAAAG
GCATTCGCGGATCTTACAGCGGTTACGCGACCAACAAAGCAGCTGGTTGCGTCCTGGCGGATAGCTCCGTTAACAGCCTGATC
GACAATGTGATCGCTAAGAATTACCCGCAATTCGGTGCTGTTGAATTAAAGGGCACTGCAAGCTACAACATTGTATCGAACGT
TATCGGTGCGGATTGTCAGCACGTGACTTACAACGGCACTGAGGGACCGATCGCTCCTAGTAACAATCTGATCAAGGGCGTTA
TGGCGAACAACCCGAAATACGCGGCAGTTGTGGCGGGTAAAGGCTCGACGAATCTGATCTCTGATGTACTGGTAGACTATTCT
ACCAGCGATGCTCGTCAGGCGCATGGTGTTACCGTCGAAGGATCTGATAACGTGATTAACAACGTACTGATGTCCGGTTGCGA
CGGAACTAATTCCCTGGGTCAGCGTCAAACCGCAACTATCGCGCGTTTCATCGGTACTGCAAATAACAACTATGCTAGCGTGT
TCCCATCCTATTCTGCCACTGGTGTGATCACGTTTGAGTCTGGCAGTACCCGTAACTTCGTCGAGGTTAAGCATCCGGGCCGT
CGCAACGATCTTCTGTCATCGGCAAGCACGATTGACGGCGCTGCGACCATCGACGGGACTTCTAACTCTAACGTAGTACACGC
GCCTGCTCTGGGCCAATACATTGGCTCCATGAGTGGTCGCTTTGAATGGCGTATTAAGTCAATGAGCCTGCCGTCCGGCGTAC
TCACTAGCGCGGATAAATACCGTATGCTGGGTGACGGTGCTGTTAGCCTTGCTGTTGGCGGAGGAACTAGCAGTCAGGTGCGC
TTGTTCACCTCAGACGGTACTTCTCGCACTGTTTCTCTGACCAATGGTAACGTGCGCCTGAGCACGTCCTCTACTGGCTATTT
ACAGCTGGGTGCAGACGCAATGACTCCGGACTCCACTGGTACTTACGCGTTAGGCTCCGCATCTCGTGCTTGGAGTGGCGGAT
TCACTCAGGCAGCATTCACCGTTACTTCTGACGCACGTTGCAAAACTGAGCCTTTAACCATCTCTGACGCTTTACTGGATGCT
TGGAGTGAAGTGGACTTTGTCCAGTTCCAGTATCTGGATCGTGTTGAAGAGAAAGGTGCTGACTCCGCGCGTTGGCATTTCGG

AATCATCGCCCAGCGTGCTAAAGAGGCATTCGAACGTCACGGCATCGATGCGCATCGTTACGGTTTCTTATGCTTTGACTCTT
GGGACGATGTGTACGAAGAGGATGCAAATGGATCTCGCAAACTGATCACTCCGGCGGGTAGTCGCTATGGTATTCGCTATGAG
GAAGTTCTGATCCTCGAAGCAGCGCTGATGCGTCGCACGATCAAGCGCATGCAGGAAGCACTGGCTGCGTTACCGAAGTAAAA
CTATATTGTGAGGCTTGCATAATGGCATTCAGAATGAGTGAACAACCACGGACCATAAAAATTTATAATCTGCTGGCCGGAAC
TAATGAATTTATTGGTGAAGGTGACGCATATATTCCGCCTCATACCGGTCTGCCTGCAAACAGTACCGATATTGCACCGCCAG
ATATTCCGGCTGGCTTTGTGGCTGTTTTCAACAGTGATGAGGCATCGTGGCATCTCGTTGAAGACCATCGGGGTAAAACCGTC
TATGACGTGGCTTCCGGCGACGCGTTATTTATTTCTGAACTCGGTCCGTTACCGGAAAATTTTACCTGGTTATCGCCGGGAGG
GGAATATCAGAAGTGGAACGGCACAGCCTGGGTGAAGGATACGGAAGCAGAAAAACTGTTCCGGATCCGGGAGGCGGAAGAAA
CAAAAAAAAGCCTGATGCAGGTAGCCAGTGAGCATATTGCGCCGCTTCAGGATGCTGCAGATCTGGAAATTGCAACGAAGGAA
GAAACCTCGTTGCTGGAAGCCTGGAAGAAGTATCGGGTGTTGCTGAACCGTGTTGATACATCAACTGCACCTGATATTGAGTG
GCCTGCTGTCCCTGTTATGGAGTAATGACGCATCCTCACGATAATATCCGGGTAGGACGAACAATAAGGCCGCAAATCGCGGC
CTTTTTTATTGATAACAAAAGGACAGTTTTCCCTTTGATATGTAACGGTGAACAGTTGTTCTACTTTTGTTTGTTAGTCTTGA
TGCTTCACTGATAGATACAAGAGCCATAAGAACC

p2074

GATGCCTTACTTTTGTTGCAATGGCTGTCTACCCTGTCTACCTGAGTAAAGAAAAATACATTTAATTCAGTATATTAACTTGG
GTAGACAGCCTTTTTTTACTGTCTACCTTCTGTCTACCCTCTCTACCTGATTTTACCTGAATCAGACAGGGAGGTAGACACGG
GGTAGACAGTGGATAAAAGCACTCTACCCCACTGAAAGCAGTGCCATTACTGGCATGGTTGCCAGTAAGGTTGATAAGGTAGA
CAAGGGGAGGGACAACTCAAAACTTTTTAAACGAGGGGGTAAAACGCAGATCAAAACGATTTCACCAAAAAGCCTCGCTTTCA
GCACCTGCCGTTTCCTTTCTTTTCAGAGGGTATTTTAAATAAAAACATTAAGTTATGACGAAAAAAAACGGAAACGCCTTAAA
CCGGAAAATTTTCATAAATAGCGAAAACCCGCGAGGTCGCCGCCCCGTAACCAGTCGGAAAGCGGAAAGTACCTGTAAAGTGA
TAATGATTATCTCAAACATATCACAACGTGCGTAAAGGGTATCTATGAACGTCGTGTGATTCGTTATCAATTGACAGCTAGCT
TAGTGCTACGTATATACATACATGCTTGTTTGTTTGTAAACTACCGAGTAAAGGTTAAGGAGGTACTTAATGGTTAGTAAAGG
TGAAGAGGATAACATGGCTATCATCAAAGAGTTTATGCGCTTCAAGGTACATATGGAGGGCAGTGTAAATGGTCATGAGTTTG
AGATCGAAGGCGAAGGCGAAGGCCGTCCGTACGAGGGAACACAAACTGCGAAACTGAAAGTAACTAAAGGTGGCCCGTTACCT
TTTGCGTGGGATATTTTATCGCCTCAATTTATGTACGGATCTAAGGCTTATGTTAAGCACCCAGCGGATATCCCGGACTATTT
AAAACTGTCTTTCCCGGAGGGTTTTAAATGGGAGCGTGTTATGAATTTTGAAGATGGCGGTGTCGTAACCGTTACTCAAGATA
GCTCCCTGCAAGATGGCGAGTTTATTTACAAAGTTAAACTGCGTGGTACTAATTTTCCATCTGATGGCCCGGTAATGCAAAAA
AAGACGATGGGCTGGGAGGGTAGTAGCGAGCGCATGTACCCGGAAGACGGTGCGCTGAAAGGCGAAATTAAGCAACGCTTAAA
ATTAAAAGATGGCGGGCATTATGACGCAGAGGTTAAAACCACGTACAAAGCGAAGAAGCCAGTGCAGTTACCTGGCGCGTACA
ATGTGAATATTAAACTGGATATTACCTCTCATAATGAGGACTATACGATCGTAGAACAATATGAGCGTGCAGAAGGCCGTCAC
TCAACTGGGGGAATGGATGAACTGTACAAATAAGTAAGTAGGAAACACTGAAAAAAGCCCGCACCTGACAGTGCGGGCTTTTT
TTTTCGACCAAAGGTATTACGTATCGTGTAAGTAGCGAAGGCCCGTGAGCGATATACAGTTTACGTTAATCTCTGCTCAACGA
CTGGTGCTGGATTTAATTTCGCTGACGTAAAGAAATTATCGGCAGTGCGTCAACTGCCGTATCTTTATCTTAATTAGGTAGTT
GGACAAGCCCTTGAAAGAAATAGCAAGAACATGCCCCTATATTGAAGTCACGGCTAAAGTCGGGTAGAAATCAAAGAAAGCAG
AAATTAAATCGGAGTAATACTAAGTTGGGATAACTCCGTAACTGACTACTCGTTTCTCTAGACTTTACTTGAACATATACACT
GTCTTTGACACGTTGAAGGATTAGAGCAATCCTATCCAAGACTGGCTAACCACGAAGCAAGTCTAGAGGGTTAAAAAGTTACT
TCCTGTATTCGGTACGAGGGTACTAGAAGATTGCAGGTACTCCGACGTTAAGTAAATTACAAAGTAATAAGTATCGTTCAGGA
TCACGTTACCGCAATAAGAAGCGACAATAATATCATTTCCGAAGTGCTTACACCTGTAGTAACTATTCCTATAACCATTACAG
GTGTCCTTCGTCGGAAATTTGCCACGAAAGAGAAAGTAACTCCTCGACAATAATACAGGGGCGCTCTTCACCTTTTCTACTTG
GTTGGCTAATCTAGGTAACTCTTAAAGGAGTTACGGCGAAGTGAAGCCGACACCTTTGAATTGTTTTAGGGGCGTTATTCGAG
GGCAATCGGAGCTAACTTCAAGACTACTTCTTTGTTGAATACTAAATAGTGCTAATGTAGTGTTTCCTCAAGGATACTCCGCT
AACAATATAGGATTCCAATCAGAGTTAGCACTGGTACTACGGGTGTAACGGTGAGGTTTTCGGGTTTACGGCTGGAAGCTAGC
ACGGTAGGAAGCCTTTCAATCACAAAGCAAAAGGGCCGTCGAAGGCCCACAAGATACGAAAGCTCTCGAAGCCTTATCCTTGA
ACGATCCACCTATTTAGGCAGTTACGCACAAAAGCTACCCAATAATCCGTGACAGGCACAATATCACGGAACAAAGGCGAAAA
CTCTCGTACACGGTTAGGTTTTCGCTAGGAAGAATAAACCTCTATCTTGATTATGAGTACGTGCCCCAAGCACCCCCATTAAC
GAAATAGCGGTTTACAATAAGTGACAAGTTACGAGTGTAGACACGCAGAATTATCCAGCCTTTAGTCTTTAGACAGGTAAAGC
TATTGTACGCGGTTGCAGTCGTAGCAATTTACCAACTGTAGAATTATTGCACACACGTAACAAGGGCTTACAGTTGAAGTTTA
ATAAGGTCACACGAAAAAACGCTAACGAATAATCGCACCGTTAGCGAAACACTATTGCAGAGAGAATAGTAAAGGTTGAGTAA
AATGATATTCCAAAGTGAGCATTTATAAAAAGTAAAGAGTAATAATAAAACCGTCGATCGGAAAACAATCGCCTGAAATCTCA
AGCACGTTGCCCTTTCTAACGTCGCTAACGTTTCGTAAACCCCTTTAATTAGGAAGAAGAATAAGTAACCCGATTATGTTTGA
GATGGCGCGTTATCGGTTTAGATTAAGAGTGGATACCAGCGCAGTCAACGCTGACGCAAATGTACAGTGATTCAATCCTGTTC
CACGGTTAACCACAATCAGTTAGCAAGATCTTGGAATAGAGTCGTTGCACCGCTTTGATTTACATGCTCTCCATTGCACAACA
TACCGGAATGAATGCCTTATCTGCCATGTTCGGATAATGAAAAACATCAGTATGCCCTGTCATTTTTCTTTGGGTGTCCTCAA
ATAATTGCCCTCACGTTATCGTATGTGACGCGTACATCTATGCTCGAAGTATTCCTAGTTCTCCCATCTTTTAATATAAAGTC
TTTAATGAACGTGTCGTTACGCAGTGTATGAACTATTTTTTTATAGGGCAGACTTTGGCGTGGCTTAAGTGTGTTCGATAAGA
AGGCAAGGACAACTAGCTGACGCGCTGTAATACGGATATTATGGCACGTATTTGTACAAACTTAGATATCCTGGTTTTCTAAT
GTGCCTAACACGTTAGTTGAGTGCCACGTACCGACTACAAGTTGCTTCAAGACTGGAATTAGGATTAGGCAATAGCCCCCCGT
TTCTACCTCAAGACGCGACGAGTATTAACCGCGCCAGCTTTCGGCACAAGTGCCAAAGAAGATTCCTAATTCTTATTCCCGAA
TAACCTCCGAATCCCTGCGGTAAAATCACGTACCGAATAGTCTAGATGAAAGGGGAACAGTTAGGTATAATTAGCTAAAGGGA
GTACCAGCCGTGACCACACCGTAGTAACCACAAACTTACGCTGGGGCTTCTTTAGCGGATTTTTACAGATACTAACAAGGTGA

GTTGAAGTACCTTAGTTGAGGATTTAAACGCGCTATCCGGTAGTCTACAAATTGGGAAATACCGTTCAAAGAGGGCTAGAATT
ACTTAAAAGCCTTCACACCGCCTGCGTTATACGCGCCTACTCTCCCGTTTATCCGTCCAAGCGGAAGCAGGGCGTACTACCGC
TAACATATTCTTACGTGTAACGTAGCTAACTATCTCAAATAGCTGGCGTACGCGTTGTACACCGCCTACAGGATCGGGAGTCG
CCGGACGAGCGTGTTATTGTGGACTTACGCCAGCGTAGACTACAACGCGCCCAGATTAACCCTGCACGTATTGCCTTGAATAA
CGTACTAATCTCTCCGGCTATCGACCATTTATCGAGCGACTCGATTATCAACGGGTGTCTTGCAGTTCTAATCTCTTCCCCCC
GCCCGTAATAGCCTCCAAGTGATTCAAGATACGAAAGGGCAAATGCTTATTCGGCGTTGAAGGATAGCGTACTTTCGGTCAAC
CACGATTCCCCACTCTACAAATCTAGCCGTGCGAACAACGATGAAAGGACAAGCAACCCAAGACGGCTGATCATAAACTCACC
TAATTCCTAAGTGAGCTAAAGAATCGAGGTGACAGCTATTAATAAATAGAGCGTTAACGTCTTACGGTCGTGAAAGTAATAGT
ACAACGGGTATTAACTTACTGACGATATTGCTTGAAGCTGTACCGTTTTATTGGGTGAACGAATAAGATCCAGCAATTCAGCC
AAAGAAGCTAACAATTGCTAGTTTAAGTTAGACACGTTTGATCTCGCGCGTGGTTAGCAGATCGTAGAGCTTACGAGCAAGCG
GAAACAGTAGCAGAAGGATAAGTAAGGGGAGTAAGTGATCGAACGAATCAGAAGCGACAATATAATTGGGATGGATATCGTAC
CGTGAATCCCAACCCTCACCAACTACGAGATAAGAGTTAATCCAGAAATCGGCATGGTGGCGACTAACGACTGTTCCCCCCCT
GTAACTAATCGTTGCATCAATAACTGACTTACTTCAAGGCCAATTCCAAGCGCAAAGAATACCTTACTAATTCTTCGGTTAAG
TTTCCGAAGTAGGATGAGCATACCTCTCTTTGCGTCTTATTACCACTGTTATAGCTATTTACTTTGTATTGCCTGCAATGGAA
TTTAATGAACTATCATATAGTGGGGATAACGGGAAAGTTACTATATTTGCTAACTAACTTATTCGTCCACCTCGAAGCTACCT
AATCACACCCAACCCGCGCGGGGTAAATAAGGCACTAATTCGAGCTTAGAGCTTGCGTACCACTTAGACACAAGTTAATTATC
AATTGTCTGGTAGTTTGACGGTATTAGTGAGATCCCAGACGCAAGGCAGAGTTAATTTTAACCTAAAGCCACAAATAAGACAG
GTTGCACAAGCCCGCCGGAAATTAAATCTTGATCACTTCGGTAACGGAGTTTACCTCCCGCGTACTTGATTCCTAATAAGAAA
CGCGCCCAAGTCCTATCAGGCAAAATTCAGCCCCTTCACGTCTTAGAACGAGGGTAAAAATACAAGCCGATTGTACAAGGGTT
GGGCTTCAAATCGTCGTTTACCCCACTTTACAACGGAGGGTAATTAGTTCACCCTATAGTACGAAGCAGAACTATTACGAGGG
GCGTGCAATAATCGAATCTTCTGCGGTTGACTTAACACGCTAGTGACGTGCCCTCGATATAGTCGCAGGTATTCCTACTAACA
CTGCCTTACACTCATCTAGTCATTCACCGATAAAATTGACTCGCCCTCTAATGTAGCGAGTACGTCCTAAAAGGCTTCGGACA
GGGCTATATAGGAGAGTTTGATCTCGCCCCGACAACTGCAACCCTCGACTCCCTTAGATAATATTGATAGCCGAAGTAGCACT
ACCCGCCGTCCACGGACTTCACTTAGGGTGACGCTTCTTAATCTGACAACGTGCAACCCCTATCGAGGGCGATTGTTTCTGCG
AAAGGGGTTGCCCTAACAGACGCGACAATTGGCCCTTGTAGGGGTGCAACCACTTAGTTTCGCGCCGTAGTCCTAAAGGCCCA
CCTATTGACTTTGTTTCGGGTAGCACTAGGAATCTTAACAATTTGAGTTTGCACGTGGAACGCGTACACCTTGATCTTCGAAT
AATTCTAGGTATTACTAGTTCACCTATGTTGACTCACCTAAAATGCTACCAGTAAAAATTTACAGACGCGGGCTTCTTGGAGC
AGATCCGAATCGTCACGTGTTCGTTTACTGTTAATTGGTGGCAAATAAGCAATATCGTATTCCGTCAGGCCCAGCCCTGTTAT
CCACGGCGTTATTTGCTAAACTGCGTAGAACTGGATTGACTGCCTGGCAATACGTAATTATCGGTACGTAGTTCCCGAATCTG
TCCGGCTATTTCACTAATACTTTACAAACGCCCCGTATCCAAGAAAAAGGAATTTATCCACGCTCCCGTCTTTGGAACGAATA
CCGCTACAAGTGGACAGAGGATCGGTACGGCCCTCAAATAAAGCCAATACTCTACGCCCTCTTCAAGAAGTAGCAGAACTGGG
TGCAGTTGGAAAGGGAATAATTTCGTAAGGCGAGCAAATACCGTAATTAATTCGGAAGAGTTAACACGATTGGAAGTAGGAAT
AGTTTCTAACCACGGTTACTAATCCTAATAACTGAACACTGTCTGATAGATTAGTGTCAGCGTTTTGTTATCAATAAAAAAGG
CCGCGATTTGCGGCCTTATTGTTCGTCAATGTTACATTACGCCCCGCCTTGCCACTCATCGCAATATTGTTGAAGCTCATTAA
GCATACGGCCTACATGAAAACCATCACATACCGCATGGTGTACCTGGATCGCCAGAGGCATTAACACTTTGTCGCCTTGCGTA
TAATATTTACCCATAGTGAAAACAGGAGCAAAAAAGTTGTCCATATTTGCTACGTTTAAATCAAAACTGGTGAATGATACCCA
GGGATTAGCACTGACGAAAAACATATTTTCGATAAACCCTTTAGGGAAATATGCTAAATTTTCACCGTAACATGCCACATCCT
GACTATAAATGTGCAGAAACTGACGGAAATCATCATGGTATTCTGACCATAAACTACTAAACGTTTCAGTCTGTTCATGGAAA
ACGGTGTAACAAGGATGGACACTATCCCAGATCACTAATTCACCGTCTTTCATTGCCATCCTAAATTCAGGATGTGCATTCAT
CAGGCGGGCAAGAATGTGAATAAAGGCCGGATAAAACTTGTGCTTATTTTTCTTTACGGTTTTTAAAAAGGCCGTAATATCCA
GTTGAACAGTTTGATTATAGGTGCACTGCGCAACTGACTGAAAGGCCTCAAAATGTTCTTTACGATGCCATTGACTAATATCA
ACTGTAGTATAACCTGTAATTTTCTTCTCCATTTTAGATTCCTTAGGTTGCGAAATCTGTTTCATTATGGTGAAAGTTGGAAC
GTCTTACGTGCCTTCGCAACAAGTACATTACTC

p2075

GATGCCTTACTTTTGTTGCAATGGCTGTCTACCCTGTCTACCTGAGTAAAGAAAAATACATTTAATTCAGTATATTAACTTGG
GTAGACAGCCTTTTTTTACTGTCTACCTTCTGTCTACCCTCTCTACCTGATTTTACCTGAATCAGACAGGGAGGTAGACACGG
GGTAGACAGTGGATAAAAGCACTCTACCCCACTGAAAGCAGTGCCATTACTGGCATGGTTGCCAGTAAGGTTGATAAGGTAGA
CAAGGGGAGGGACAACTCAAAACTTTTTAAACGAGGGGGTAAAACGCAGATCAAAACGATTTCACCAAAAAGCCTCGCTTTCA
GCACCTGCCGTTTCCTTTCTTTTCAGAGGGTATTTTAAATAAAAACATTAAGTTATGACGAAAAAAAACGGAAACGCCTTAAA
CCGGAAAATTTTCATAAATAGCGAAAACCCGCGAGGTCGCCGCCCCGTAACCAGTCGGAAAGCGGAAAGTACCTGTAAAGTGA
TAATGATTATCTCAAACATATCACAACGTGCGTAAAGGGTATCTATGAACGTCGTGTGTATCTATGAACGTCGTGTGATTCGT
TATCAATTGACAGCTAGCTTAGTGCTACGTATATACATACATGCTTGTTTGTTTGTAAACGTCTAAATCGATAAGAGGGGGAA
ACAATGGTTAGTAAAGGTGAAGAATTATTTACAGGTGTAGTTCCGATCTTAGTTGAACTGGACGGCGATGTTAATGGTCATAA
ATTCAGTGTATCCGGTGAGGGTGAAGGTGACGCAACCTATGGCAAGTTAACCCTGAAACTGATCTGCACTACTGGAAAATTAC
CAGTACCGTGGCTTACTCTGGTAACTACCTTAGGTTACGGCTTACAGTGTTTTGCGCGTTACCCTGACCACATGAAACAACAT
GATTTCTTCAAATCTGCAATGCCGGAAGGTTATGTACAGGAGCGCACCATATTCTTTAAAGACGATGGCAACTATAAAACCCG
TGCAGAAGTTAAATTTGAAGGTGACACCCTGGTGAATCGTATTGAACTGAAAGGCATTGATTTCAAAGAGGACGGCAATATTT
TAGGCCATAAACTGGAATATAACTATAACAGTCATAACGTTTACATCACGGCAGACAAGCAAAAAAACGGTATCAAAGCTAAC
TTTAAGATTCGTCATAACATCGAAGACGGCGGGGTACAGCTTGCGGATCATTACCAACAAAACACTCCGATTGGCGATGGTCC
TGTTTTACTGCCGGATAACCACTACTTAAGCTACCAAAGTGCGCTGTCTAAGGATCCGAATGAAAAGCGCGACCATATGGTAT

TATTAGAGTTTGTAACCGCTGGGGGTATTACGCTGGGTATGGATGAGTTATACAAATAAGTAAGTAGGAAACACTGAAAAAAG
CCCGCACCTGACAGTGCGGGCTTTTTTTTTCGACCAAAGGTATTACGTATCGTGTAAGTAGCGAAGGCCCGTGAGCGATATAC
AGTTTACGTTAATCTCTGCTCAACGACTGGTGCTGGATTTAATTTCGCTGACGTAAAGAAATTATCGGCAGTGCGTCAACTGC
CGTATCTTTATCTTAATTAGGTAGTTGGACAAGCCCTTGAAAGAAATAGCAAGAACATGCCCCTATATTGAAGTCACGGCTAA
AGTCGGGTAGAAATCAAAGAAAGCAGAAATTAAATCGGAGTAATACTAAGTTGGGATAACTCCGTAACTGACTACTCGTTTCT
CTAGACTTTACTTGAACATATACACTGTCTTTGACACGTTGAAGGATTAGAGCAATCCTATCCAAGACTGGCTAACCACGAAG
CAAGTCTAGAGGGTTAAAAAGTTACTTCCTGTATTCGGTACGAGGGTACTAGAAGATTGCAGGTACTCCGACGTTAAGTAAAT
TACAAAGTAATAAGTATCGTTCAGGATCACGTTACCGCAATAAGAAGCGACAATAATATCATTTCCGAAGTGCTTACACCTGT
AGTAACTATTCCTATAACCATTACAGGTGTCCTTCGTCGGAAATTTGCCACGAAAGAGAAAGTAACTCCTCGACAATAATACA
GGGGCGCTCTTCACCTTTTCTACTTGGTTGGCTAATCTAGGTAACTCTTAAAGGAGTTACGGCGAAGTGAAGCCGACACCTTT
GAATTGTTTTAGGGGCGTTATTCGAGGGCAATCGGAGCTAACTTCAAGACTACTTCTTTGTTGAATACTAAATAGTGCTAATG
TAGTGTTTCCTCAAGGATACTCCGCTAACAATATAGGATTCCAATCAGAGTTAGCACTGGTACTACGGGTGTAACGGTGAGGT
TTTCGGGTTTACGGCTGGAAGCTAGCACGGTAGGAAGCCTTTCAATCACAAAGCAAAAGGGCCGTCGAAGGCCCACAAGATAC
GAAAGCTCTCGAAGCCTTATCCTTGAACGATCCACCTATTTAGGCAGTTACGCACAAAAGCTACCCAATAATCCGTGACAGGC
ACAATATCACGGAACAAAGGCGAAAACTCTCGTACACGGTTAGGTTTTCGCTAGGAAGAATAAACCTCTATCTTGATTATGAG
TACGTGCCCCAAGCACCCCCATTAACGAAATAGCGGTTTACAATAAGTGACAAGTTACGAGTGTAGACACGCAGAATTATCCA
GCCTTTAGTCTTTAGACAGGTAAAGCTATTGTACGCGGTTGCAGTCGTAGCAATTTACCAACTGTAGAATTATTGCACACACG
TAACAAGGGCTTACAGTTGAAGTTTAATAAGGTCACACGAAAAAACGCTAACGAATAATCGCACCGTTAGCGAAACACTATTG
CAGAGAGAATAGTAAAGGTTGAGTAAAATGATATTCCAAAGTGAGCATTTATAAAAAGTAAAGAGTAATAATAAAACCGTCGA
TCGGAAAACAATCGCCTGAAATCTCAAGCACGTTGCCCTTTCTAACGTCGCTAACGTTTCGTAAACCCCTTTAATTAGGAAGA
AGAATAAGTAACCCGATTATGTTTGAGATGGCGCGTTATCGGTTTAGATTAAGAGTGGATACCAGCGCAGTCAACGCTGACGC
AAATGTACAGTGATTCAATCCTGTTCCACGGTTAACCACAATCAGTTAGCAAGATCTTGGAATAGAGTCGTTGCACCGCTTTG
ATTTACATGCTCTCCATTGCACAACATACCGGAATGAATGCCTTATCTGCCATGTTCGGATAATGAAAAACATCAGTATGCCC
TGTCATTTTTCTTTGGGTGTCCTCAAATAATTGCCCTCACGTTATCGTATGTGACGCGTACATCTATGCTCGAAGTATTCCTA
GTTCTCCCATCTTTTAATATAAAGTCTTTAATGAACGTGTCGTTACGCAGTGTATGAACTATTTTTTTATAGGGCAGACTTTG
GCGTGGCTTAAGTGTGTTCGATAAGAAGGCAAGGACAACTAGCTGACGCGCTGTAATACGGATATTATGGCACGTATTTGTAC
AAACTTAGATATCCTGGTTTTCTAATGTGCCTAACACGTTAGTTGAGTGCCACGTACCGACTACAAGTTGCTTCAAGACTGGA
ATTAGGATTAGGCAATAGCCCCCCGTTTCTACCTCAAGACGCGACGAGTATTAACCGCGCCAGCTTTCGGCACAAGTGCCAAA
GAAGATTCCTAATTCTTATTCCCGAATAACCTCCGAATCCCTGCGGTAAAATCACGTACCGAATAGTCTAGATGAAAGGGGAA
CAGTTAGGTATAATTAGCTAAAGGGAGTACCAGCCGTGACCACACCGTAGTAACCACAAACTTACGCTGGGGCTTCTTTAGCG
GATTTTTACAGATACTAACAAGGTGAGTTGAAGTACCTTAGTTGAGGATTTAAACGCGCTATCCGGTAGTCTACAAATTGGGA
AATACCGTTCAAAGAGGGCTAGAATTACTTAAAAGCCTTCACACCGCCTGCGTTATACGCGCCTACTCTCCCGTTTATCCGTC
CAAGCGGAAGCAGGGCGTACTACCGCTAACATATTCTTACGTGTAACGTAGCTAACTATCTCAAATAGCTGGCGTACGCGTTG
TACACCGCCTACAGGATCGGGAGTCGCCGGACGAGCGTGTTATTGTGGACTTACGCCAGCGTAGACTACAACGCGCCCAGATT
AACCCTGCACGTATTGCCTTGAATAACGTACTAATCTCTCCGGCTATCGACCATTTATCGAGCGACTCGATTATCAACGGGTG
TCTTGCAGTTCTAATCTCTTCCCCCCGCCCGTAATAGCCTCCAAGTGATTCAAGATACGAAAGGGCAAATGCTTATTCGGCGT
TGAAGGATAGCGTACTTTCGGTCAACCACGATTCCCCACTCTACAAATCTAGCCGTGCGAACAACGATGAAAGGACAAGCAAC
CCAAGACGGCTGATCATAAACTCACCTAATTCCTAAGTGAGCTAAAGAATCGAGGTGACAGCTATTAATAAATAGAGCGTTAA
CGTCTTACGGTCGTGAAAGTAATAGTACAACGGGTATTAACTTACTGACGATATTGCTTGAAGCTGTACCGTTTTATTGGGTG
AACGAATAAGATCCAGCAATTCAGCCAAAGAAGCTAACAATTGCTAGTTTAAGTTAGACACGTTTGATCTCGCGCGTGGTTAG
CAGATCGTAGAGCTTACGAGCAAGCGGAAACAGTAGCAGAAGGATAAGTAAGGGGAGTAAGTGATCGAACGAATCAGAAGCGA
CAATATAATTGGGATGGATATCGTACCGTGAATCCCAACCCTCACCAACTACGAGATAAGAGTTAATCCAGAAATCGGCATGG
TGGCGACTAACGACTGTTCCCCCCCTGTAACTAATCGTTGCATCAATAACTGACTTACTTCAAGGCCAATTCCAAGCGCAAAG
AATACCTTACTAATTCTTCGGTTAAGTTTCCGAAGTAGGAGTGAGCATACCTCTCTTTGCGTCTTATTACCACTGTTATAGCT
ATTTACTTTGTATTGCCTGCAATGGAATTTAATGAACTATCATATAGTGGGGATAACGGGAAAGTTACTATATTTGCTAACTA
ACTTATTCGTCCACCTCGAAGCTACCTAATCACACCCAACCCGCGCGGGGTAAATAAGGCACTAATTCGAGCTTAGAGCTTGC
GTACCACTTAGACACAAGTTAATTATCAATTGTCTGGTAGTTTGACGGTATTAGTGAGATCCCAGACGCAAGGCAGAGTTAAT
TTTAACCTAAAGCCACAAATAAGACAGGTTGCACAAGCCCGCCGGAAATTAAATCTTGATCACTTCGGTAACGGAGTTTACCT
CCCGCGTACTTGATTCCTAATAAGAAACGCGCCCAAGTCCTATCAGGCAAAATTCAGCCCCTTCACGTCTTAGAACGAGGGTA
AAAATACAAGCCGATTGTACAAGGGTTGGGCTTCAAATCGTCGTTTACCCCACTTTACAACGGAGGGTAATTAGTTCACCCTA
TAGTACGAAGCAGAACTATTACGAGGGGCGTGCAATAATCGAATCTTCTGCGGTTGACTTAACACGCTAGTGACGTGCCCTCG
ATATAGTCGCAGGTATTCCTACTAACACTGCCTTACACTCATCTAGTCATTCACCGATAAAATTGACTCGCCCTCTAATGTAG
CGAGTACGTCCTAAAAGGCTTCGGACAGGGCTATATAGGAGAGTTTGATCTCGCCCCGACAACTGCAACCCTCGACTCCCTTA
GATAATATTGATAGCCGAAGTAGCACTACCCGCCGTCCACGGACTTCACTTAGGGTGACGCTTCTTAATCTGACAACGTGCAA
CCCCTATCGAGGGCGATTGTTTCTGCGAAAGGGGTTGCCCTAACAGACGCGACAATTGGCCCTTGTAGGGGTGCAACCACTTA
GTTTCGCGCCGTAGTCCTAAAGGCCCACCTATTGACTTTGTTTCGGGTAGCACTAGGAATCTTAACAATTTGAGTTTGCACGT
GGAACGCGTACACCTTGATCTTCGAATAATTCTAGGTATTACTAGTTCACCTATGTTGACTCACCTAAAATGCTACCAGTAAA
AATTTACAGACGCGGGCTTCTTGGAGCAGATCCGAATCGTCACGTGTTCGTTTACTGTTAATTGGTGGCAAATAAGCAATATC
GTATTCCGTCAGGCCCAGCCCTGTTATCCACGGCGTTATTTGCTAAACTGCGTAGAACTGGATTGACTGCCTGGCAATACGTA
ATTATCGGTACGTAGTTCCCGAATCTGTCCGGCTATTTCACTAATACTTTACAAACGCCCCGTATCCAAGAAAAAGGAATTTA
TCCACGCTCCCGTCTTTGGAACGAATACCGCTACAAGTGGACAGAGGATCGGTACGGCCCTCAAATAAAGCCAATACTCTACG
CCCTCTTCAAGAAGTAGCAGAACTGGGTGCAGTTGGAAAGGGAATAATTTCGTAAGGCGAGCAAATACCGTAATTAATTCGGA
AGAGTTAACACGATTGGAAGTAGGAATAGTTTCTAACCACGGTTACTAATCCTAATAACTGAACACTGTCTGATAGATTAGTG

TCAGCGTTTTGTTATCAATAAAAAAGGCCGCGATTTGCGGCCTTATTGTTCGTCAATGTTACATTACGCCCCGCCTTGCCACT
CATCGCAATATTGTTGAAGCTCATTAAGCATACGGCCTACATGAAAACCATCACATACCGCATGGTGTACCTGGATCGCCAGA
GGCATTAACACTTTGTCGCCTTGCGTATAATATTTACCCATAGTGAAAACAGGAGCAAAAAAGTTGTCCATATTTGCTACGTT
TAAATCAAAACTGGTGAATGATACCCAGGGATTAGCACTGACGAAAAACATATTTTCGATAAACCCTTTAGGGAAATATGCTA
AATTTTCACCGTAACATGCCACATCCTGACTATAAATGTGCAGAAACTGACGGAAATCATCATGGTATTCTGACCATAAACTA
CTAAACGTTTCAGTCTGTTCATGGAAAACGGTGTAACAAGGATGGACACTATCCCAGATCACTAATTCACCGTCTTTCATTGC
CATCCTAAATTCAGGATGTGCATTCATCAGGCGGGCAAGAATGTGAATAAAGGCCGGATAAAACTTGTGCTTATTTTTCTTTA
CGGTTTTTAAAAAGGCCGTAATATCCAGTTGAACAGTTTGATTATAGGTGCACTGCGCAACTGACTGAAAGGCCTCAAAATGT
TCTTTACGATGCCATTGACTAATATCAACTGTAGTATAACCTGTAATTTTCTTCTCCATTTTAGATTCCTTAGGTTGCGAAAT
CTGTTTCATTATGGTGAAAGTTGGAACGTCTTACGTGCCTTCGCAACAAGTACATTACTC

p2076

TCTTGCGGGCCTCTGTACTGTAGTATGTTGTATGATACTACATACTACAACAATTTAACAGAGCCATCTTGGAATCTGGTGTC
TCTGCGCCTATAATTCTGGAACAGCTACTTTCCGAACGACTCCTGCGTTGATCGGAAATCCAGAAGCCCGAGAGGTTGCCGCC
TTTCGGGCTTTTTCTTTTTCAAAAAAAAAAATTTATAAAACGATCTGTTGCGGCCGCCGGGTTGTGGGCAAAGGCGCTGGCGC
TCGACGGTGGGCAACCGCTTGCGGTTGTCCACGGGCGGAGCCGGTGCGCGTAGCGCATTGTCCACAAGCCAAGGGCGACCAAT
AATTGATATATATATTCATAATTGAAAAGCTAATTGAACATACTACTTGCTGTAACTACTTGCCGGAGCGAGGGGTGTTTGCA
AGCTGTTGATCTGAAAGGGCTATTAGCGTTCTCACGTGCCTTTTTGATTAGCGATTTCACGTGACCTTATTAGCGATTTCACG
TACTCCGATTAGCGATTTCACGTACCCTGATTAGCGATTTCACGTGGATAGTTTTTGGAGCGGGCCGGAAAGCCCCGTGAATC
AAGGCTTTGCGGGGCATTAGCGGTTTCACGTGGATAACTACCCTCTATCCACAGGCTTCCGGGGATAAAAAAGCCCGCTCGAC
GGCGGGCTGTTGGATGGGAAGGCTTGACCAAGCCAAGCGTAGCGTTGGCCTGGTCAAGTCGGAGGGGGGCCGATGCGAGCGCC
CTTGCCGGGTGCGCGGGTGACATGCAGGCGTGTGGATTTGATGCGCAGGCATTCGCCGTCATCTTCGATGCAGTCGCTTGCCT
CGGGATAGACAATCAACACTTCGCGTAGGCGCTTTTTGAAGTTGTATTTGAAGCTGGCGAGTGCTGCCCGCTCTGCCCGCTCT
CGGGCCTTATCGTCCAGTTCGGGCGAGTTGCGTGCGCGGCTGCCATAGGATGAGCCGAATTGCGCTTGCAGGGCGACCCAAGG
GATTTGCACGAAGGGGCGGCCCTTGGCCCGCAACAGGAACACGCGATAGGTCAGCCACGTGTAAATGTCCATCGCAAGCGGAG
ACTGCCGCAAGGCATGCAGGTAGTCGATTCGGATAGGAACCGGTGAGCGGGTGACTTCCTCGAAGAAATCGCCTGTGAGGGTG
AGGGTGCTATCCCATAGCGCCCGATCTTCTGGCCGCTTGGGATTCCAGAATAGAAAAGCGCGCTTGGCAATGACGACGTTCTC
AATGCCGAAGTCATTGCCTTGCTCGCCGGCAAGCGAAATCATGGATGAAAACAGGCGTTGCGCCTGATTGCGAAGGGTGGCCG
TGTAACGGCCATCGGTGTGCATTCCGAGCCTTTGTAGAAATTCCGATTGCGACCGGCCAAGGTTCAACACGGGGTCTTTCGTT
CGCACGGCCTCGGTGCATATCCAAGCAAGCAAGGTGCGCGGCATAGAACCGTAGGGCAGGCCGATGCTCGGCTTGCCCATGAT
CGACAAGGTGACGATGCCATTGGTGCGCTCAAAGTAGCTGGTCTTGGGGTCGGTGTGGGGCATGGTCGCTTGCACAAGGCAAC
GGGCCATGTAGCCGACTAAGCCAGCTTCGCGGGCATCCTCCATTTCGAGCGCGAGGCTCGTCTTGATGATCTCGTTGATACGA
TGGCCGGGGGCTTTGTTGTTCTTAGGCATGTTGTTCCCTCCCCGGCATGGTGATGGTTGGTCTAGTGTTTGTGGGTTTGATGT
TCCGGCGTTTGATGAACAGGCGCAAGGTGTGAGGGCTGACGCCTAACAACTCGGCTGCGCGACTTTGCGGCAAGCCAAGGTTC
ACGTATGCCTGTACTTCATCAATACGGCTGTCCAGCTTCAAGGCGCTCGATTTGCTGCCCTTGGGTCGCCCGAGCGTCTTGCC
GCGCTCTCTGGCGACTTGTAGCGCCTCGGTGGTACGTGCCTGAATGAAATGCCGCTCGATCTGTGCAGCCAAGCCAAGCACGG
TTGCCATGATGTCGCTTTGTAGGCTGCCGTCCATGATGATCTTCTGTTTGGTCACATGGACGATTAGGCCGCGCTCGCTCGCC
GCTTTGAGAATTTCCAAGGCGGCGAGGGCGGAACCGGCAATGCGCGTAATCTCCGGCGTCAGTAGCACGTCGCCACGCTCGGC
CTTTTCGATGATTGCTCCGAGCTTGCGCTTGCGCCAGTCCTTTGCTCTGCTGGCAATTTCTTCCTCGATCTGTAGCGGCGCGA
AGCCTTTGGCGTTCGCGTATTCGAGCAAACCGTATTTTTGGTTTTCCGGGTCTTGGCCGTCACGCGAAACCCGGAGATAGGCA
TAGTATTTTGGCATTTGCAGGGAAAACGTCAGATTCGGTTAAACATGCCTCATTCTAGCGCAGATTAAATAGGAATTAAATAC
CCTGTAGCGGTATAGATAAAACGTTGGTTTGGAGCTCTGGTCTGACAGCTCTAGCAGAAATCATCCTTAGCGAAAGCTAAGGA
TTTTTTTTATCTGGTAACGGCCGCCAGTGTGCTGGAATTCGGCTTAAAGCTTAAAAAGGCTGACGATTTCTCGTCAGCCTTTG
CGGCCGCTGGTGGCCCCTGCTGGACTTGAACCAGCGACCAAGCGATTATGAGTCGCCTGCTCTAACCACTGAGCTAAGGGGCC
AATCGATTGGATTATAAAGTAACTCCGTGTCGCAATCCAAGCTTGGCTGCAGGTCACTTTGATACATGAAAATACGGGTTTTC
TTGATTCAGACGCGCAGCGGTGTGCGTTTGTTTGCCGCTATAGCGAAATAAATCAGAAAATCAGACGCGGTCGTTCACTTGTT
CAGCAACCAGATCAAAAGCCATTGACTCAGCAAGGGTTGACCGTATAATTCACGCGATTACACCGCATTGCGGTATCAACGCG
CCCTTAGCTCAGTTGGATAGAGCAACGACCTTCTAAGTCGTGGGCCGCAGGTTCGAATCCTGCAGGGCGCGCCATTACAATTC
AATCAGTTACGCCTTCTTTATATCCTCCATAATTTCAGAGTGGGACATATTTGGGACATTATCACCAAAAATGTCGTCTATTT
TCCTCGCATGCTCTGTCAAATGATTAGGCGCAAGGTGAGCATACCTACGAACCATTTCTATGGACTCCCATCCGCCCATTTCC
TGAAGCACTGATAATGGGACGCCTGACTGAATCAGCCAGCTTGCCCAGGTGTGTCTGAGGTCATGGAAAACGGAAAATCTTCA
ATTCCTGCACGACGACAGCTGATAGCCATGATGTCTTGCTGTCGATGCGCATCTTCCTGACCGCAGGCGTTGATGTTCCATCT
GCTCGCTTAGCCGCCTTGGTATGTGACGGGATCCTCTACGCGGACGCATCGTGTCGGCATCACCGGCGCCACAAGTGCGATGC
TGGCGCCTATATCGCCGACATCACCGATGGGGAAGATCGGGCTCGCCACTTCGGGCTCATGAGGTACCTAGGCTGTTTCTTCA
TCGTGTCGCATAAAATGTGACCAATAAAACAAATTATGCAATTTTTTAGTTGCATGAACTCGCATGTCTCCATAGAGTGCGCG
CTACTTGATGCCGACTTAGCTCAGTAGGTAGAGCAACTGACTTGTAATCAGTAGGTCACCAGTTCGATTCCGGTAGTCGGCAC
CATCAAGTCCGGTGGGGTTCCCGAGCGGCCAAAGGGAGCAGACTGTAAATCTGCCGTCACAGACTTCGAAGGTTCGAATCCTT
CCCCCACCACCAATTTCGGCCACGCGATGGCGTAGCCCGAGACGATAAGTTCGCTTACCGGCTCGAATAAAGAGAGCTTCTCT
CGATATTCAGTGCAGAATGAAAATCAGGTAGCCGAGTTCCAGGATGCGGGCATCGTATAATGGCTATTACCTCAGCCTTCCAA
GCTGATGATGCGGGTTCGATTCCCGCTGCCCGCTCCAAGATGTGCTGATATAGCTCAGTTGGTAGAGCGCACCCTTGGTAAGG
GTGAGGTCGGCAGTTCGAATCTGCCTATCAGCACCACTTCTTTTCTCCTCCCTGTTTTTTCATTCTGTTATTGCATTCAACAA

GTCGGGCATGTTGCCTGGTTGATGTGGTGATATCACCGATTTATCCGTGTCTTAGAGGGACAATCGATGACGTCCAAAAAGCA
GAACGTGCGGAAAACATTAAGAAAAATTATAAAAACCCGGCATAAATGGCGAGGGTTTAAGCAATCGAGCGGCAGCGTACTTA
CCCCGCACTCCATTAGCGGGTATACTCATGCCGCATTGTCCTCTTAGTTAAATGGATATAACGAGCCCCTCCTAAGGGCTAAT
TGCAGGTTCGATTCCTGCAGGGGACACCATTTATCAGTTCGCTCCCATCCGTACCAGTCCGCAAAATCCCCTGAATATCAAGC
ATTCCGTAGATTTACAGTTCGTCATGGTTCGCTGTTCGCAACCTAACCAAACAGTCACTTTCGAGCAATTTTCCTTGAAAAAG
AGGTTGACGCTGCAAGGCTCTATACGCATAATGCGCCCCGCAACGCCGATAAGGTATCGCGAAAAAAAAGATGGCTACGTAGC
TCAGTTGGTTAGAGCACATCACTCATAATGATGGGGTCACAGGTTCGAATCCCGTCGTAGCCACCATCTTTTTTTGCGGGAGT
GGCGAAATTGGTAGACGCACCAGATTTAGGTTCTGGCGCCGCAAGGTGTGCGAGTTCAAGTCTCGCCTCCCGCACCATTCACC
AGAAAGCGTTGTACGAGAAGCGTTTTATCGCTAACTGATTAATTATAAATCAGTTAGCGAAATATCTTACTTGCAATCGGTGT
GGAAAACGGTAGTATTAGCAGCCACGAGTCGGCACGTAGCGCAGCCTGGTAGCGCACCGTCATGGGGTGTCGGGGGTCGGAGG
TTCAAATCCTCTCGTGCCGACCAAAAATCCCAAGAAAAAACCAACCCTTACGGTTGGTTTTTTTATATCTGCAATTAATTCGA
TAAACAGACCTCAGGCATTTGAGAAGCACACGGTCACACTGCTTCCGGTAGTCAATAAACCGGTAAACCAGCAATAGACATAA
GCGGCTATTTAACGACCCTGCCCTGAACCGACGACCGCGCAATTTATTTAAAAAGGGACTAGACAGAGGGGTGGGAAGTCCGT
ATTATCCACCCCCGCAACGGCGCTAAGCGCCCGTAGCTCAGCTGGATAGAGCGCTGCCCTCCGGAGGCAGAGGTCTCAGGTTC
GAATCCTGTCGGGCGCGCCATCACATACCGCCATTAGCTCATCGGGATAGAACGCCAGCCTTCGAAGCTGGTTTCGCGGGGTT
CGAGTCTCCGATGGCGGTCCAATTTTGAACCCCGCTTCGGCGGGGTTTTTTGACGACCGGGTCGAATTTGCTTTCGAATTTCT
GCCATTCATCCGCTTATTATCACTTCTGTTGTTTGTCGGACCCTAGGTCTAGGGCGGCGGGAGTCACTAAGGGCTAACTAACT
AATTACGTAGCAATCAACTCACTGGCTCACCTTCACGGGTGGGCCTTTCTTCGGCACGGGCAAATTGCTGAATATTCCTTTTC
TTAGACGTCAGGTGGCAAACGCGAAGTAATCTTTTCGGTTTTAAAGAAAAAGGGCAGGGTGGTGACACCTTGCCCGTTTTTTT
GCCGGAGGGTACCTGTTGTTATAAAAACGCGTCGCAATCGTCTGTTGTTATTGCGTTAGTCTGCGTTACTCCTTTAACTTTCC
GCGTAATATATTCATGTCCGTAAACTTTCGCGGCTGGCTTCATAGCCTTGCCAAAGTCATTTACGTTTAGCGGTTTGCTCCTG
CCTGCGTACGCCATAAACGCCAGATAGACGCGGTAAAGGCTGTTTCTGGTCGTGTACTTCACTGAATCGCCACCGCCGCCCAT
CATCAGGCCGCGTGCTTCCTCCAGAAAATTCAGGAACTGGCAAAACTCAATAACCGGATCCGTCTGTTGCTTTATTGCCAGTG
CTTCATCACCGTCACGCTGTTCAATGAGTAAAGCCCGTGCCTTTTCAGGGTCGGCAAAGTTCGCCAGCAAGCGGCGGATAATG
ACAGGGATCTCAGCCGCGATCTTTTCCGGTAGCTCCCTGTCTTTTTCTGCCTCGCTTACGATGTTATCGAACCGGAATATCAC
CCGACGACGTGCCACACCTCCGGCCCGTTCGGTGAATATCATCGGATTGTTATTGGTTGCCAGCACCACCGCCCTGATTACCG
CCGTAAAACGCTTTTCATATTTCGGGTTAATTTCCACGGGGTCGCCGCCCGTGATTTTCTTGATGCCCGTTCCTTCGCCTGTA
TATTTCGGCTGGTCTGCCAGGACGATAAGACGACTCCCGACAACCTGCGCACGCCCACCAGCATCATCAAGCGATGTCATTTC
AGCGCTTACCGTGTTTTGTTTCCCTGCCAGAAGGCTGGCTATGTGTGTGAATGTACTTTTACCGCTCCCGCCGTCTCCGGTGG
CCTCAATAAACATCTGCCAGTCGTACCGGTTCGCCATAATCATGTACAGCGCGGCACATATACGCATCATCTTGCGCGGGTCT
TTTCCGGCTGCGTGCTCAAGCCATTTATGAAAGTTTGGCGCGTTATCGCGGATGTTCTCCCCGGGTGCTGGTGGCGTGTACTC
AATGCCGTTGTGCGTGGTGATCCAGTTCTCCGGCGTGTGCGGGGAAAATTCCCCCGTTTTCAGGTCAAGCGCACCATTGGCGA
ACGGCAGCAAATCGCCGGACGGCTCGCCCATTGGTTCGGCAATAACTTTTAACGCTTCCACGGCGTTATTGATTACGCGCTTG
CTGAAAGTGGCCCTGTGCTCTGAATAGATCGCCACCATTTCGCGGCTCAGCTCCATTGTGCTGACCGGACACCATACCCCGCC
GCGCCATACGTGAACGATTTCACTTTCAGGATGTACGCAAACGCCATCAAAGCGATCGGCAAGCAGCTGCGCGCGCTCACTGT
CCGCCATCTGCGAAAGTTGCGCCTTTTGCTTTACCGGAAGCTCAATGACCAGACCATCAGAAAGATTCTGGCGCTCACGGGCC
AGATATTCGCGCCAGTTCTCCACCTTCTGACCGTGCATACCATCAGGGTAAAAATTTGCATCCTGTACGCCTGCCGCCGCCAG
CTTCTGACCAATCGCCTTTATCATTACTGGCGCAAGATATCCGGCCCTGAATATGCGCACGGATTTTCTGCCTTCCGGCACAA
TTTGCAGCTTATCCAGTTCGGATAACTGCTGCTCCCCAAGCCACACAGGAGGCTCATTATCTCCGGCCATACGCGCGTCATGT
TCCTGCCATTGTTTTGCGTGTGACCAGGCATCACTACCCGCAAAAATAATGACTTCTGTTTCTTTGTGTTTTATGCCGCGTGA
CTGCTGTTTTACGTTCGGTGCCAGTTTCATTTTTTGAAACCGAATCGTTTAGTCAAGAGAGATCCTTAGGCCACACGTTCAAG
TGCAGCCACAGGATAAATTTGCACTGAGCCTGGGTGGGATTCGGACTCGACCGCATAGCCTTCAGGAGTGAGTTTTGTGCAAT
ACCAACCGACGACTTGACCCTGCCAAGCGGCACCAGATTTCTTGCGTACGCGATCCCCTAAGCCAAAGGTGGCACTCAGGGGA
AGCGCAAACTGCCCTGCAACGGGAGCGTTGGCTTCATCGCTACTTTGACCCATACTTTTCCTTCTCCTTATTAAAGATCCGCT
AGCATTATACCTAGGACTGAGCTAGCTGTCAAAAAACCGCCACTGCGCCGTTACCACCGCTCGAGGCGCTGTGCGCTATGGCG
ATCTGGAAGTGTCGGGACGAGGGGCGTCCCGGCGGTAAATTCGACGTGCCTGCGGCGCGTCGAACAAGGGGCGTGCCCGGTGT
CAGTTCGTGGCATTTTTCGAGGCGCGACGCCATTTCCAAGGCTCCTGAGCATTCGGGTCTGACCAAAGGCCGAGCCGTTGGCG
GCGGGCCTCTTTTTCATACTCGTACATCTGCGCGTCGGTTGGTTTGCCGTAATAACGGTAAGCCCAGGCCATGCCTTCTTGAA
CCATGATCGCATTGATGTTGGTGAGTTGTGTTTGGCCGCCGGGGTATTGCAACGGCGCGTAAACGACCCCAAGAGTGCGGCCA
TACCGATCAACCTCTTTTTCGGTCACTTGAACCTCTTGGCGAAAGGTCAAGTCGGCGAGCCGTTGGCGAGCACGGGAGCCGAA
GGCTTGGCCGCTTTCCGGTGCGTCAATATCGGCCAATCTCACGCGGATGGTCTGACGGTTCACCAAAACGTCGATAGTGTCAC
CGTCAAGGATTCGGACGACTTCACCCCGGAAGTCGGCCCAAGCGGGCACACTGACGATTAGGACGACAGCGGCCGCGACCGCG
CGAAGGGCGGCAAGGGCGCTTTTCATTGTTTGCCTCCTGTTTTCAAGACGGCTGTGAGATTGGCGACCTGCTCTTTGAGGGCT
TCCACCTGACCTTGCAGACTGGCGGCGCGCTCGATGGCCTCCTTGGCCTGTTTGCGGGCCTCGATAGCCTCGTTATCGCGTTG
GGTGAGCTTTTCCATGCAGCGGTTTAGTTCTTCGCCGCTGCGGCGTTTCACTTCGGCAAGCTGGTCGGCCAGCTTGTCGCGCT
CGCGTTCCATAGGTTCGAGCTGATTCACTCGTTCGCGGAGCTGGTCGTTTTCGCGGGTGAAGGTGTCGGCTAGTTCGATTGCT
TCGGCAAGCTGCTGGCTGATGGCCGCTTTGTCGGCCTCGATCTGTTTCCGATCTTCGTCAAACCGGGCGTTGGCGTGCGCCAG
GGCGATAGCCCATAGCGCATTGCCAAGCTCGGCAAGATGCTCGTTGACTGCAACCGGCAATGGGTCTGATGAGGGCAGGGTGG
CGGTCTTGCGGTTTTTCCATTCAGCCATTGCATCGGAAATGGTTGTGAAGCTACCGCTTCCGAGTTTCTTGCGCACGGCGGCC
AAAGTGGGCCGGATGCCTTCGGCGTCCAGTTCGTCGGCTGCTCGCCAAATGTCTTGTTTAGTGATTGCCAT

p2292

TCAGATCCTTCCGTATTTAGCCAGTATGTTCTCTAGTGTGGTTCGTTGTTTTTGCGTGAGCCATGAGAACGAACCATTGAGAT
CATACTTACTTTGCATGTCACTCAAAAATTTTGCCTCAAAACTGGTGAGCTGAATTTTTGCAGTTAAAGCATCGTGTAGTGTT
TTTCTTAGTCCGTTACGTAGGTAGGAATCTGATGTAATGGTTGTTGGTATTTTGTCACCATTCATTTTTATCTGGTTGTTCTC
AAGTTCGGTTACGAGATCCATTTGTCTATCTAGTTCAACTTGGAAAATCAACGTATCAGTCGGGCGGCCTCGCTTATCAACCA
CCAATTTCATATTGCTGTAAGTGTTTAAATCTTTACTTATTGGTTTCAAAACCCATTGGTTAAGCCTTTTAAACTCATGGTAG
TTATTTTCAAGCATTAACATGAACTTAAATTCATCAAGGCTAATCTCTATATTTGCCTTGTGAGTTTTCTTTTGTGTTAGTTC
TTTTAATAACCACTCATAAATCCTCATAGAGTATTTGTTTTCAAAAGACTTAACATGTTCCAGATTATATTTTATGAATTTTT
TTAACTGGAAAAGATAAGGCAATATCTCTTCACTAAAAACTAATTCTAATTTTTCGCTTGAGAACTTGGCATAGTTTGTCCAC
TGGAAAATCTCAAAGCCTTTAACCAAAGGATTCCTGATTTCCACAGTTCTCGTCATCAGCTCTCTGGTTGCTTTAGCTAATAC
ACCATAAGCATTTTCCCTACTGATGTTCATCATCTGAGCGTATTGGTTATAAGTGAACGATACCGTCCGTTCTTTCCTTGTAG
GGTTTTCAATCGTGGGGTTGAGTAGTGCCACACAGCATAAAATTAGCTTGGTTTCATGCTCCGTTAAGTCATAGCGACTAATC
GCTAGTTCATTTGCTTTGAAAACAACTAATTCAGACATACATCTCAATTGGTCTAGGTGATTTTAATCACTATACCAATTGAG
ATGGGCTAGTCAATGATAATTACTAGTCCTTTTCCTTTGAGTTGTGGGTATCTGTAAATTCTGCTAGACCTTTGCTGGAAAAC
TTGTAAATTCTGCTAGACCCTCTGTAAATTCCGCTAGACCTTTGTGTGTTTTTTTTGTTTATATTCAAGTGGTTATAATTTAT
AGAATAAAGAAAGAATAAAAAAAGATAAAAAGAATAGATCCCAGCCCTGTGTATAACTCACTACTTTAGTCAGTTCCGCAGTA
TTACAAAAGGATGTCGCAAACGCTGTTTGCTCCTCTACAAAACAGACCTTAAAACCCTAAAGGCTTAAGTAGCACCCTCGCAA
GCTCGGTTGCGGCCGCAATCGGGCAAATCGCTGAATATTCCTTTTGTCTCCGACCATCAGGCACCTGAGTCGCTGTCTTTTTC
GTGACATTCAGTTCGCTGCGCTCACGGCTCTGGCAGTGAATGGGGGTAAATGGCACTACAGGCGCCTTTTATGGATTCATGCA
AGGAAACTACCCATAATACAAGAAAAGCCCGTCACGGGCTTCTCAGGGCGTTTTATGGCGGGTCTGCTATGTGGTGCTATCTG
ACTTTTTGCTGTTCAGCAGTTCCTGCCCTCTGATTTTCCAGTCTGACCACTTCGGATTATCCCGTGACAGGTCATTCAGACTG
GCTAATGCACCCAGTAAGGCAGCGGTATCATCAACGGGGTCTGACGCTCAGTGGAACGAAAACTCACGTTAAGGGATTTTGGT
CATGAGATTATCAAAAAGGATCTTCACCTAGATCCTTTTAAATTAAAAATGAAGTTTTAAATCAATCTAAAGTATATATGAGT
AAACTTGGTCTGACAGTTACGTTTCCACAACCAATTAACCAATTCTGATTTAGAAAAACTCATCGAGCATCAAATGAAACTGC
AATTTATTCATATCAGGATTATCAATACCATATTTTTGAAAAAGCCGTTTCTGTAATGAAGGAGAAAACTCACCGAGGCAGTT
CCATAGGATGGCAAGATCCTGGTATCGGTCTGCGATTCCGACTCGTCCAACATCAATACAACCTATTAATTTCCCCTCGTCAA
AAATAAGGTTATCAAGTGAGAAATCACCATGAGTGACGACTGAATCCGGTGAGAATGGCAAAAGCTTATGCATTTCTTTCCAG
ACTTGTTCAACAGGCCAGCCATTACGCTCGTCATCAAAATCACTCGCATCAACCAAACCGTTATTCATTCGTGATTGCGCCTG
AGCGAGACGAAATACGCGATCGCTGTTAAAAGGACAATTACAAACAGGAATCGAATGCAACCGGCGCAGGAACACTGCCAGCG
CATCAACAATATTTTCACCTGAATCAGGATATTCTTCTAATACCTGGAATGCTGTTTTCCCGGGGATCGCAGTGGTGAGTAAC
CATGCATCATCAGGAGTACGGATAAAATGCTTGATGGTCGGAAGAGGCATAAATTCCGTCAGCCAGTTTAGTCTGACCATCTC
ATCTGTAACATCATTGGCAACGCTACCTTTGCCATGTTTCAGAAACAACTCTGGCGCATCGGGCTTCCCATACAATCGATAGA
TTGTCGCACCTGATTGCCCGACATTATCGCGAGCCCATTTATACCCATATAAATCAGCATCCATGTTGGAATTTAATCGCGGC
CTCGAGCAAGACGTTTCCCGTTGAATATGGCTCATAACACCCCTTGTATTACTGTTTATGTAAGCAGACAGTTTTATTGTTCA
TGATGATATATTTTTATCTTGTGCAATGTAACATCAGAGATTTTGAGACACAACGTGGCTTTCCCTGCAGGATTTCGGAGGCC
TGCGTTATCCCCTGATTCTGTGGATAACCGTATTACCGCCTTTGAGTGAGCTGATACCGCTCGCCGCAGCCGAACGCCGACTA
GTGGATTTTACGGCTAGCTCAGTCCTAGGTACAATGCTAGCgaattcattaaagaggagaaaggtacccATGGCACGTACCCC
GAGCCGTAGCAGCATTGGTAGCCTGCGTAGTCCGCATACCCATAAAGCAATTCTGACCAGCACCATTGAAATCCTGAAAGAAT
GTGGTTATAGCGGTCTGAGCATTGAAAGCGTTGCACGTCGTGCCGGTGCAAGCAAACCGACCATTTATCGTTGGTGGACCAAT
AAAGCAGCACTGATTGCCGAAGTGTATGAAAATGAAAGCGAACAGGTGCGTAAATTTCCGGATCTGGGTAGCTTTAAAGCCGA
TCTGGATTTTCTGCTGCGTAATCTGTGGAAAGTTTGGCGTGAAACCATTTGTGGTGAAGCATTTCGTTGTGTTATTGCAGAAG
CACAGCTGGACCCTGCAACCCTGACCCAGCTGAAAGATCAGTTTATGGAACGTCGTCGTGAGATGCCGAAAAAACTGGTTGAA
AATGCCATTAGCAATGGTGAACTGCCGAAAGATACCAATCGTGAACTGCTGCTGGATATGATTTTTGGTTTTTGTTGGTATCG
CCTGCTGACCGAACAGCTGACCGTTGAACAGGATATTGAAGAATTTACCTTCCTGCTaATTAATGGTGTTTGTCCGGGTACAC
AGCGTTAACTAGGGCCCATACCCCCAATTATTGAAGGCCGCTAACGCGGCCTTTTTTTGTTTCTGGTCTGCCCGACGTACGGT
GAAtctgattcgttaccaattgacATGATACGAAACGTACCGTATCGTTAAGGTTACTAGattaaagaggagaaatactagAT
GGCAGTAAAGATTTCAGGAGTCCTGAAAGACGGCACAGGAAAACCGGTACAGAACTGCACCATTCAGCTGAAAGCCAGACGTA
ACAGCACCACGGTGGTGGTGAACACGGTGGGCTCAGAGAATCCGGATGAAGCCGGGCGTTACAGCATGGATGTGGAGTACGGT
CAGTACAGTGTCATCCTGCAGGTTGACGGTTTTCCACCATCGCACGCCGGGACCATCACCGTGTATGAAGATTCACAACCGGG
GACGCTGAATGATTTTCTCTGTGCCATGACGGAGGATGATGCCCGGCCGGAGGTGCTGCGTCGTCTTGAACTGATGGTGGAAG
AGGTGGCtCGTAACGCGTCCGTGGTGGCACAGAGTACGGCAGACGCGAAGAAATCAGCCGGCGATGCCAGTGCATCAGCTGCT
CAGGTCGCGGCCCTTGTGACTGATGCAACTGACTCAGCACGCGCCGCCAGCACGTCCGCCGGACAGGCTGCATCGTCAGCTCA
GGAAGCGTCCTCCGGCGCAGAAGCGGCATCAGCAAAGGCCACTGAAGCGGAAAAAAGTGCCGCAGCCGCAGAGTCCTCAAAAA
ACGCGGCGGCCACCAGTGCCGGTGCGGCGAAAACGTCAGAAACGAATGCTGCAGCGTCACAACAATCAGCCGCCACGTCTGCC
TCCACCGCGGCCACGAAAGCGTCAGAGGCCGCCACTTCAGCACGAGATGCGGTGGCCTCAAAAGAGGCAGCAAAATCATCAGA
AACGAACGCATCATCAAGTGCCGGTCGTGCAGCTTCCTCGGCAACGGCGGCAGAAAATTCTGCCAGGGCGGCAAAAACGTCCG
AGACGAATGCCAGGTCATCTGAAACAGCAGCGGAACGGAGCGCCTCTGCCGCGGCAGACGCAAAAACAGCGGCGGCGGGGAGT
GCGTCAACGGCATCCACGAAGGCGACAGAGGCTGCGGGAAGTGCGGTATCAGCATCGCAGAGCAAAAGTGCGGCAGAAGCGGC
GGCAATACGTGCAAAAAATTCGGCAAAACGTGCAGAAGATATAGCTTCAGCTGTCGCGCTTGAGGATGCGGACACAACGAGAA
AGGGGATAGTGCAGCTCAGCAGTGCAACCAACAGCACGTCTGAAACGCTTGCTGCAACGCCAAAGGCGGTTAAGGTGGTAATG
GATGAGACTAATCGTAAGGCACCTCTGGACAGTCCGGCACTGACCGGAACGCCAACAGCACCAACCGCGCTCAGGGGAACAAA
CAATACCCAGATTGCGAACACCGCTTTTGTACTGGCCGCGATTGCAGATGTTATCGACGCGTCACCTGACGCACTGAATACGC
TGAATGAACTGGCCGCAGCGCTCGGGAATGATCCAGATTTTGCTACCACCATGACTAACGCGCTTGCGGGTAAACAACCGAAG
AATGCGACACTGACGGCGCTGGCAGGGCTTTCCACGGCGAAAAATAAATTACCGTATTTTGCGGAAAATGATGCCGCCAGCCT

GACTGAACTGACTCAGGTTGGCAGGGATATTCTGGCAAAAAATTCCGTTGCAGATGTTCTTGAATACCTTGGGGCCGGTGAGA
ATTCGGACGCAGCACTGCGTTCCCAGATTTCTAACCCGGAAGGCGCAATCCTCTATCCGGAACTTCACCGCGCGCGTTGGTTA
GATGAGAAGGATGCTCGCGGCTGGGGTGCGAAGGGCGATGGCGTTACCGACGACACTGCAGCGCTGACTTCCGCCCTGAACGA
TACTCCGGTGGGTCAGAAAATCAACGGTAACGGTAAAACTTATAAAGTTACGTCCCTGCCGGACATCTCCCGCTTTATCAACA
CCCGTTTCGTGTATGAACGTATCCCAGGCCAGCCGCTGTACTACGCATCGGAAGAGTTCGTTCAGGGTGAGCTTTTTAAAATC
ACCGACACTCCGTATTATAACGCCTGGCCACAGGATAAGGCTTTCGTGTACGAAAACGTTATCTATGCTCCGTACATGGGTTC
CGACCGTCACGGTGTCAGCCGACTGCACGTAAGCTGGGTGAAATCGGGCGACGATGGTCAGACCTGGAGCACGCCTGAGTGGC
TGACCGACCTTCATCCGGACTATCCGACCGTTAACTATCACTGCATGAGCATGGGCGTCTGTCGCAACCGTCTGTTCGCAATG
ATCGAAACCCGTACGCTGGCAAAAAACGCTCTGACTAACTGCGCCCTGTGGGATCGTCCAATGAGCCGCTCTCTGCACCTGAC
GGGTGGTATTACCAAAGCAGCGAACCAGCGTTACGCCACCATTCACGTACCGGATCATGGTCTGTTCGTTGGTGACTTTGTAA
ATTTCTCTAATTCTGCAGTTACCGGTGTGTCTGGCGACATGACCGTTGCGACCGTAATCGATAAGGACAATTTCACCGTCCTG
ACCCCGAACCAGCAAACCTCTGATCTTAACAACGCTGGCAAGAACTGGCACATGGGCACTAGCTTTCACAAATCTCCGTGGCG
TAAAACCGATCTGGGCCTGATCCCGTCTGTAACTGAAGTGCACTCCTTCGCGACCATTGATAACAACGGTTTCGCTATGGGTT
ATCACCAAGGTGATGTTGCACCGCGTGAAGTCGGCCTCTTTTATTTTCCGGACGCATTCAACAGCCCGTCCAACTACGTGCGC
CGTCAGATTCCGTCTGAATATGAACCGGACGCCTCCGAGCCGTGCATTAAGTACTATGACGGTGTGCTGTACCTGATTACCCG
TGGCACCCGTGGTGATCGTCTGGGTTCATCTCTGCATCGCTCCCGCGACATTGGTCAGACGTGGGAAAGTCTGCGCTTCCCGC
ACAATGTTCATCACACCACCCTGCCGTTCGCGAAAGTCGGCGATGACCTGATCATGTTTGGCTCCGAACGTGCTGAAAACGAA
TGGGAAGCGGGCGCCCCAGACGATCGCTACAAGGCATCTTACCCGCGCACCTTCTACGCGCGTCTGAACGTGAACAACTGGAA
CGCAGACGATATCGAATGGGTAAACATCACCGACCAGATCTACCAGGGTGGTATCGTGAACTCTGGTGTGGGCGTTGGTTCCG
TTGTAGTTAAAGATAACTACATCTATTATATGTTCGGCGGCGAAGACCACTTCAACCCGTGGACTTACGGCGATAACTCCGCG
AAAGACCCGTTCAAATCCGATGGTCACCCTTCTGACCTCTATTGTTACAAAATGAAAATCGGTCCGGACAACCGTGTTTCCCG
CGATTTTCGCTACGGCGCTGTTCCAAACCGTGCAGTTCCGGTATTCTTCGACACGAACGGCGTGCGTACCGTTCCGGCTCCGA
TGGAATTCACCGGCGACCTGGGTCTGGGCCACGTAACCATTCGTGCCTCCACCAGCTCTAACATCCGTTCCGAAGTACTCATG
GAAGGTGAATACGGCTTTATCGGTAAGTCTATCCCGACGGACAACCCGGCAGGTCAGCGTATCATCTTCTGCGGCGGTGAGGG
TACCTCTAGCACCACCGGCGCGCAAATCACCCTGTACGGCGCTAACAACACCGACTCTCGTCGTATCGTATACAACGGTGATG
AACATCTGTTCCAGTCCGCAGACGTGAAACCGTACAACGACAACGTCACCGCACTGGGTGGTCCATCCAACCGTTTCACCACT
GCGTACCTGGGTTCCAACCCGATCGTTACTAGCAATGGTGAACGCAAAACTGAACCGGTAGTGTTTGACGACGCTTTTCTGGA
CGCATGGGGCGATGTTCATTACATCATGTATCAGTGGCTGGATGCCGTGCAGCTGAAAGGTAACGACGCGCGTATCCACTTTG
GTGTGATCGCACAGCAGATTCGCGATGTCTTCATCGCACACGGTCTGATGGATGAAAATAGTACTAACTGTCGCTATGCGGTG
CTGTGCTATGACAAATACCCGCGTATGACCGACACCGTGTTCTCGCACAATGAGATTGTTGAACATACCGATGAAGAAGGTAA
CGTGACTACTACCGAAGAACCGGTTTATACCGAAGTGGTTATTCACGAAGAAGGTGAAGAATGGGGCGTGCGTCCTGATGGTA
TCTTTTTCGCGGAGGCAGCGTACCAGCGTCGCAAACTGGAACGCATCGAAGCTCGTCTGTCGGCACTGGAACAGAAATAAAAC
TATATTGTGAGGCTTGCATAATGGCATTCAGAATGAGTGAACAACCACGGACCATAAAAATTTATAATCTGCTGGCCGGAACT
AATGAATTTATTGGTGAAGGTGACGCATATATTCCGCCTCATACCGGTCTGCCTGCAAACAGTACCGATATTGCACCGCCAGA
TATTCCGGCTGGCTTTGTGGCTGTTTTCAACAGTGATGAGGCATCGTGGCATCTCGTTGAAGACCATCGGGGTAAAACCGTCT
ATGACGTGGCTTCCGGCGACGCGTTATTTATTTCTGAACTCGGTCCGTTACCGGAAAATTTTACCTGGTTATCGCCGGGAGGG
GAATATCAGAAGTGGAACGGCACAGCCTGGGTGAAGGATACGGAAGCAGAAAAACTGTTCCGGATCCGGGAGGCGGAAGAAAC
AAAAAAAAGCCTGATGCAGGTAGCCAGTGAGCATATTGCGCCGCTTCAGGATGCTGCAGATCTGGAAATTGCAACGAAGGAAG
AAACCTCGTTGCTGGAAGCCTGGAAGAAGTATCGGGTGTTGCTGAACCGTGTTGATACATCAACTGCACCTGATATTGAGTGG
CCTGCTGTCCCTGTTATGGAGTAAtgacgcatcctcacgataatatccgggtagGACGAACAATAAGGCCGCAAATCGCGGCC
TTTTTTATTGATAACAAAAGGACAGTTTTCCCTTTGATATGTAACGGTGAACAGTTGTTCTACTTTTGTTTGTTAGTCTTGAT
GCTTCACTGATAGATACAAGAGCCATAAGAACC

p2325

CGCAGGATAAGTAAGGGGAGTAAGTGATCGAACGAATCAGAAGTGACAATATACTTAGGCTGGATCTCGTCCCGTGAATCCCA
ACCCTCACCAACTACGAGATAAGAGGTAAGCCAAAAATCGACTTGGTGGCGACCAACGACTGTTCCCCCCCTGTAACTAATCG
TTCCGTCAAAACCTGACTTACTTCAAGGCCAATTCCAAGCGCAAACAATACCGTCCTAGTTCTTCGGTTAAGTTTCCGAAGTA
GGAGTGAGCCTACCTCCGTTTGCGTCTTGTTACCACTGACCCAGCTATTTACTTTGTATTGCCTGCAATCGAATTTCTGAACT
CTCAGATAGTGGGGATAACGGGAAAGTTCCTATATTTGCGAACTAACTTAGCCGTCCACCTCGAAGCTACCTACTCACACCCA
CCCCGCGCGGGGTAAATAAGGCACTAATCCCAGCTGAGAGCTGGCGTAGCACTTAGCCACAAGTTAATTAACAGTTGTCTGGT
AGTTTGGCGGTATTAGGAAGATCCTAGAAGCAAGGCAGAGTTAGTTCTAACCTAAAGCCACAAATAAGACAGGTTGCCAAAGC
CCGCCGGAAATTAAATCTTGCTCAGTTCGGTAACGGAGTTTCCCTCCCGCGTACTTAATTCCCAATAAGAAACGCGCCCAAGT
CCTATCAGGCAAAATTCAGCCCCTTCCCGTGTTAGAACGAGGGTAAAAATACAAGCCGATTGAACAAGGGTTGGGGGCTTCAA
ATCGTCGTTTACCCCACTTTACAACGGAGATTAAGTAGTTCACCCTATAGTACGAAGCAGAACTATTTCGAGGGGCGTGCAAT
AATCGAATCTTCTGCGGTTGACTTAACACGCTAGGGACGTGCCCTCGATTCAATCGAAGGTACTCCTACTCAGACTGCCTCAC
ACCCAGCTAGTCACTGAGCGATAAAATTGACCCGCCCTCTAGGGAAGCGAGTACGTCCCAAAGGGCTCCGGACAGGGCTATAT
AGGAGAGTTTGATCTCGCCCCGACAACTGCAACCCTCAACTCCCTTAGATAATATTGTTAGCCGAAGTTGCACGACCCGCCGT
CCACGGACTGCTCTTAGGGTGTGGCTCCTTAATCTGACAACGTGCAACCCCTATCGAAGTCGATTGTTTCTGCGAAAGGTGTT
GTCCTAATAGTCCCGAAATTTGGCCCTTGTAGGTGTGAAACCACTTAGCTTCGCGCCGTAGTCCTAAAGGCCCACCTATTGAC
TTTGTTTCGGGTAGCACTAGGAATCTTAACAATTTGAATTTGGACGTGGAACGCGTACACCTTAATCTCCGAATAATTCTAGG
GATTTGGAAGTCCTCTACGTTGACACACCTACACTGCTCGAAGTAAATATACGAATAACGCGGGCCTCGCGGAGCCGTTCCGA

ATCGTCACGTGTTCGTTTACTGTTAATTGGTGGCAAATAAGCAATATCGTAGTCCGTCAGGCCCAGCCCTGTTATCCACGGCG
TTATTTGTCAAATTGCGTAGAACTGGATTGACTGCCTGACAATACCTAATTATCGGTACGAAGTCCCCGAATCTGTCGGGCTA
TTTCACTAATACTTTCCAAACGCCCCGTATCCAAGAAGAACGAATTTATCCACGCTCCCGTCTTTGGGACGAATACCGCTACA
AGTGGACAGAGGATCGGTACGGGCCTCTAATAAATCCAACACTCTACGCCCTCTTCAAGAGCTAGAAGAACAGGGTGCAGTTG
GAAAGGGAATTATTTCGTAAGGCGAGCCAATACCGTAATTAATTCGGAAGAGTTAACACGATTGGAAGTAGGAATAGTTTCTA
ACCACGGTTACTAATCCTAATAACGGAACGCTGTCTGATAGATTAGTGTCAGCGCTCGGTACCAAAGAAAAATAAAAAGACGC
TGAAAAGCGTCTTTTTATTTTTCGGTCCAGTGTAACTCAGGCAAAAGCACGTAATATTCGTACTTTCTTCCTCCGTAAGCGTC
ACCCACATTCCTTAAAGAGTGCATGTGCATATTTTGTTATCAATAAAAAAGGCCGCGATTTGCGGCCTTATTGTTCGTCTTGC
CGGATTACGCCCCGCCCTGCCACTCATCGCAGTATTGTTGTAATTCATTAAGCATTCTGCCGACATGGAAGCCATCACAAACG
GCATGATGAACTTGGATCGCCAGTGGCATTAACACCTTGTCGCCTTGCGTATAATATTTTCCCATAGTGAAAACGGGGGCGAA
GAAGTTGTCCATATTTGCTACGTTTAAATCAAAACTGGTGAAACTCACCCACGGATTGGCACTGACGAAAAACATATTTTCGA
TAAACCCTTTAGGGAAATATGCTAAGTTTTCACCGTAACACGCCACATCTTGACTATATATGTGTAGAAACTGCCGGAAATCG
TCGTGGTATTCTGACCAGAGCGATGAAAACGTTTCAGTTTGCTCATGGAAAACGGTGTAACAAGGGTGAACACTATCCCATAT
CACCAGCTCACCGTCTTTCATTGCCATACGAAACTCCGGATGTGCATTCATCAGGCGGGCAAGAATGTGAATAAAGGCCGGAT
AAAACTTGTGCTTATTTTTCTTTACGGTTTTTAAAAAGGCCGTAATATCCAGCTGAACGGTTTGGTTATAGGTGCACTGAGCA
ACTGACTGGAATGCCTCAAAATGTTCTTTACGATGCCATTGACTTATATCAACTGTAGTATATCCAGTGATTTTTTTCTCCAT
TTTAGCTTCCTTAGCTTGCGAAATCTCGATAACTCAAAAAATAGTAGTGATCTTATTTCATTATGGTGAAAGTTGTCTTACGT
GCAACATTTTCGCAAAAAGTTGGCGCTTTATCAACACTGTCGGAATGACAAATGGTTCCAATTATTGAACACCCTTCGGGGTG
TTTTTTTGTTTCTGGTTTCCCGAGGCCGGCCTGCGCTAGCGGAGTGTATACTGGCTTACTATGTTGGCACTGATGAGGGTGTA
AGTGAAGTGCTTCATGTGGCAGGAGAAAAAAGGCTGCATCGGTGCGTCAGCAGAATATGTGATACAGGATATATTCCGCTTCC
TCGCTCACTGACTCGCTACGCTCGGTCGTTCGACTGTGGCGAGCGGAAATGGCTTACGAACGGGGCGGAGATTTCCTGGAAGA
TGCCAGGAAGATACTTAACAGGGAAGTGAGAGGGTCGCGGCAAAGCCGTTTTTCCATAGGCTCCGCCCCCCTGACAAGCATCA
CGAAATCTGACGCTCAAATCAGTGGTGGCGAAACCTGACAGGACTATAAAGATACCAGGCGTTTCCCCCTGGCGGCTCCCTCG
TGCGCTCTCCTGTTCCTGCCTTTCGGTTTGCCGGTGTCATTCCTCTGTTACGGCCGAGTTTGTCTCATTCCACGCCTGACACT
CAGTTCCGGGTAGGCAGTTCGCTCCAAGCTGGACTGTATGCACGAACCCCCCGTTCAGTCCGACCGCTGCGCCTTATCCGGTA
ACTATCGTCTTGAGTCCAACCCGGAAAGACATGCAAAAGCACCACTGGCAGCAGCCACTGGTAATTGATTTAGAGGAGTTAGT
CTTGAAGTCATGCGCCGGATAAGGCTAAACTGAAAGGACAAGTTTTGGCGACTGCGCTCCTCCAAGCCAGTTACCTCGGTTCA
AAGAGTTGGTAGCTCAGAGAACCTTCGAAAAACCGCCCTGCAAGGCGGTTTTTTCGTTTTCAGAGCAAGAGATTACGCGCAGA
CCAAAACGATCTCAAGAAGATCATCTTATTAATCAGATAAAATATTTCTAGATTTCAGTGCAATTTATCTCTTCAAATGTAGC
ACCGGCGCGCCGTGACCAATTATTGAAGGCCGCTAACGCGGCCTTTTTTTGTTTCTGGTATCCCGAATGGAGCGACTTCTCCC
CAAAAAGCCTCGCTTTCAGCACCTGTCGTTTCCTTTCTTTTCAGAGGGTATTTTAAATAAAAACATTAAGTTATGACGAAGAA
GAACGGAAACGCCTTAAACCGGAAAATTTTCATAAATAGCGAAAACCCGCGAGGTCGCCGCCCCGTAACCTGTCGGATCACCG
GAAAGGACCCGTAAAGTGATAATGATTATCATCTACATATCACAACGTGCGTAAAGGGTAAGTATGAAGGTCGTGTACTCCAT
CGCTACCAAATTCCAGAAAACAGACGCTTTCGAGCGTCTTTTTTCGTTTTGGTCACGACGTACGGTGGAAGATTCGTTACCAA
TTGACAGCTAGCTCAGTCCTAGGTATATACATACATGCTTGTTTGTTTGTAAACTACTAGAAGAGCGATTGGGCTCTTCAATG
AAGCGTACCGCCGATGGCAGCGAGTTTGAATCTCCGAAGAAAAAGCGTAAGGTCAGCGAAGTTGAGTTCAGCCACGAATACTG
GATGCGTCACGCTTTAACCCTGGCTAAACGCGCGCGCGACGAGCGCGAAGTACCAGTGGGGGCGGTGCTGGTGTTAAACAACC
GCGTAATCGGCGAAGGCTGGAACCGTGCAATCGGGTTACATGACCCGACCGCCCATGCCGAGATCATGGCCCTGCGCCAGGGG
GGGCTGGTCATGCAGAATTACCGTCTGATCGACGCGACGTTGTATGTCACATTCGAGCCATGCGTGATGTGTGCGGGGGCAAT
GATTCACTCTCGCATTGGTCGCGTCGTGTTTGGCGTTCGTAATAGTAAACGCGGCGCTGCTGGCTCCTTAATGAATGTTCTGA
ATTATCCGGGTATGAACCACCGTGTCGAAATTACAGAAGGTATCTTAGCAGATGAATGTGCCGCACTGCTGTGTGACTTCTAC
CGCATGCCGCGCCAAGTATTCAACGCCCAAAAAAAAGCTCAGTCCTCAATTAACTCTGGTGGTAGTAGTGGCGGCTCTAGCGG
CTCCGAGACGCCTGGTACGTCGGAATCGGCTACGCCTGAGTCGAGCGGTGGGTCCTCTGGCGGCTCTGACAAGAAATATAGCA
TCGGCCTGGCCATCGGCACAAATAGCGTCGGATGGGCGGTGATCACTGATGAATATAAAGTTCCGTCTAAAAAGTTCAAGGTA
CTGGGTAATACAGATCGCCATAGTATCAAAAAGAACTTAATCGGTGCGCTTCTGTTCGATTCCGGCGAAACCGCAGAAGCAAC
ACGTCTGAAACGCACCGCTCGTCGCCGTTACACCCGTCGTAAAAACCGCATCTGCTACCTGCAAGAAATCTTCTCTAACGAAA
TGGCTAAAGTAGATGACAGCTTTTTTCACCGTCTGGAAGAATCATTTCTGGTGGAAGAAGATAAAAAGCACGAACGTCATCCA
ATCTTCGGCAACATTGTGGACGAAGTAGCGTATCACGAAAAATACCCGACTATCTATCACCTGCGCAAAAAGCTGGTCGATTC
GACGGATAAGGCCGATCTGCGTCTGATCTATCTGGCCTTAGCGCATATGATTAAGTTCCGTGGTCATTTCCTGATCGAAGGCG
ACCTGAATCCAGACAACAGCGATGTAGACAAACTGTTCATCCAGCTGGTGCAAACCTATAACCAGCTGTTTGAAGAAAACCCA
ATTAATGCTAGCGGTGTTGACGCGAAAGCGATCTTGTCCGCACGCCTGTCCAAATCCCGTCGTCTGGAAAACTTAATTGCGCA
ACTGCCGGGTGAGAAGAAAAACGGACTGTTCGGCAATCTGATCGCTCTTAGCTTGGGACTGACCCCGAACTTCAAAAGCAACT
TCGATCTGGCAGAGGACGCAAAACTTCAACTTAGCAAAGATACGTATGACGATGACTTGGATAACTTACTGGCCCAGATCGGA
GATCAGTACGCTGATCTGTTTCTGGCGGCAAAGAACTTATCAGACGCTATTCTCCTGTCTGATATTCTTCGTGTGAATACCGA
AATCACCAAAGCACCGCTTTCTGCATCCATGATTAAACGCTATGACGAACATCACCAAGATCTGACTCTTCTGAAAGCGCTGG
TACGGCAACAACTGCCGGAGAAGTACAAGGAGATCTTCTTTGACCAATCCAAAAACGGCTACGCGGGTTATATTGACGGGGGT
GCAAGCCAAGAGGAGTTCTACAAATTCATCAAGCCAATCTTAGAAAAAATGGATGGCACGGAAGAATTACTTGTTAAACTGAA
TCGTGAGGATCTGCTTCGTAAACAGCGTACCTTCGACAACGGTAGCATTCCGCACCAGATCCACTTAGGTGAACTGCACGCTA
TCCTGCGTCGCCAAGAGGATTTTTACCCGTTCCTGAAAGATAATCGTGAAAAAATCGAAAAAATCCTGACCTTTCGTATCCCG
TATTATGTCGGCCCGCTGGCGCGTGGCAACTCCCGTTTCGCGTGGATGACTCGCAAATCCGAAGAAACTATTACCCCGTGGAA
CTTCGAGGAAGTGGTTGACAAAGGCGCAAGCGCCCAATCCTTCATCGAGCGCATGACTAACTTTGATAAAAACCTGCCGAACG
AAAAGGTACTGCCGAAACACTCCCTTCTGTACGAATACTTCACCGTGTACAACGAGCTGACTAAAGTAAAGTATGTGACTGAG
GGCATGCGTAAACCTGCATTCCTGAGCGGTGAACAGAAAAAAGCAATTGTTGATTTACTGTTTAAAACCAACCGTAAAGTAAC

CGTTAAACAGCTGAAAGAGGACTACTTCAAGAAAATCGAATGCTTCGACTCCGTCGAGATTAGTGGAGTTGAAGATCGTTTTA
ATGCAAGTTTAGGCACGTATCACGATTTATTAAAGATCATTAAAGACAAAGATTTCTTGGACAACGAAGAAAATGAGGACATC
TTAGAGGACATCGTCCTGACCCTGACTCTGTTCGAAGATCGTGAAATGATTGAAGAACGCCTTAAGACGTATGCTCACCTGTT
TGACGATAAAGTAATGAAACAACTGAAACGTCGCCGTTATACTGGCTGGGGCCGTCTGAGCCGTAAACTGATTAACGGTATCC
GTGACAAACAGTCCGGTAAAACTATTCTGGACTTCCTGAAATCTGACGGCTTCGCAAACCGTAACTTCATGCAACTGATTCAC
GACGATTCCCTGACCTTCAAAGAGGACATCCAGAAAGCTCAGGTTTCTGGTCAAGGTGATTCTCTGCACGAGCATATCGCCAA
TTTAGCAGGTAGTCCGGCGATCAAAAAAGGTATCCTGCAAACCGTGAAAGTGGTGGATGAGCTTGTGAAAGTTATGGGTCGTC
ACAAACCGGAAAACATTGTTATCGAGATGGCTCGTGAAAACCAAACGACCCAGAAGGGACAGAAAAACTCCCGCGAACGCATG
AAACGTATCGAGGAGGGTATTAAAGAACTTGGCTCTCAGATTCTGAAAGAACACCCTGTTGAAAATACCCAACTGCAAAATGA
AAAACTGTACCTGTACTACCTGCAAAATGGTCGTGACATGTATGTAGATCAGGAGCTGGACATCAACCGCCTCTCCGATTACG
ACGTTGACCACATTGTTCCGCAGTCTTTTCTGAAAGATGATTCCATTGATAACAAAGTACTCACCCGTAGCGATAAAAACCGT
GGGAAGAGTGACAACGTTCCATCGGAAGAAGTAGTTAAGAAAATGAAGAACTATTGGCGTCAACTGCTTAACGCGAAACTGAT
TACTCAACGTAAATTTGATAACCTGACCAAAGCTGAACGTGGCGGTTTGTCTGAGCTGGATAAGGCGGGTTTTATTAAACGTC
AACTGGTAGAAACTCGCCAGATTACAAAACATGTTGCTCAGATTCTGGACTCTCGTATGAACACTAAATACGATGAAAATGAC
AAACTGATCCGCGAAGTTAAGGTTATTACCCTGAAATCTAAGCTGGTTTCCGACTTCCGTAAAGATTTCCAATTCTATAAAGT
GCGCGAGATTAACAACTATCACCACGCGCACGACGCATATCTGAATGCAGTTGTTGGCACGGCACTGATCAAAAAATATCCGA
AACTGGAAAGCGAATTTGTGTACGGCGATTATAAAGTTTACGACGTGCGCAAAATGATCGCCAAATCTGAACAGGAAATTGGC
AAAGCAACCGCTAAATACTTTTTCTACTCAAACATTATGAATTTCTTCAAAACCGAAATCACCTTAGCGAATGGCGAAATTCG
TAAACGCCCTCTGATCGAAACCAACGGCGAAACGGGTGAGATCGTGTGGGACAAAGGTCGTGATTTCGCTACTGTCCGCAAAG
TTCTGTCCATGCCTCAAGTAAACATCGTTAAAAAGACTGAGGTACAGACTGGCGGTTTCAGCAAGGAATCCATTCTGCCGAAA
CGCAACTCCGACAAACTGATCGCGCGTAAGAAAGACTGGGATCCGAAGAAATACGGTGGCTTCGATTCTCCAACCGTGGCATA
CAGCGTTCTGGTAGTCGCCAAAGTCGAAAAGGGTAAATCAAAAAAACTGAAATCAGTGAAAGAACTTTTAGGCATCACCATTA
TGGAACGTAGCTCTTTCGAAAAAAACCCGATTGACTTCCTCGAAGCGAAGGGGTACAAGGAAGTAAAGAAAGATCTGATTATC
AAACTGCCGAAGTATTCCCTGTTCGAACTGGAAAATGGTCGTAAACGTATGTTAGCGTCTGCGGGTGAACTGCAAAAAGGGAA
CGAATTGGCCCTTCCGTCCAAGTACGTGAACTTCCTGTATCTGGCCTCGCACTACGAGAAACTGAAAGGTAGTCCGGAAGATA
ATGAGCAGAAACAGCTGTTCGTGGAACAGCACAAACACTATCTGGACGAGATTATTGAACAGATTTCTGAGTTTAGCAAACGC
GTAATTCTGGCGGACGCGAATCTGGATAAAGTCCTGAGCGCCTACAATAAACACCGTGATAAACCGATCCGTGAACAGGCAGA
AAACATCATTCACCTGTTCACGCTGACTAATCTTGGTGCTCCGGCAGCCTTCAAATACTTCGACACCACGATCGATCGTAAAC
GTTACACCTCCACTAAAGAAGTCTTAGATGCAACTCTTATTCACCAGAGCATCACTGGCCTGTATGAAACTCGTATTGATCTG
AGTCAGTTGGGCGGTGACTAATGTATGCTTAAGCAGCTCGGTACCAAAGACGAACAATAAGACGCTGAAAAGCGTCTTTTTTC
GTTTTGGTCCTGTTGCGGCGCGATAGTGTGAACATGCTATAGACTTCTGGTGCTACCCGACTGACAATTAATCATCCGGCTCG
TATAATGCTAGCCGTACATAAATTGCGGGCTCGTTTTAGAGCTAGAAATAGCAAGTTAAAATAAGGCTAGTCCGTTATCAACT
TGAAAAAGTGGCACCGAGTCGGTGCTTTTTTTAATCCATTCGAATCACGTCCGACGAACAATAAGGCCTCCCTAACGGGGGGC
CTTTTTTATTGATAACAAAAGTAACTTCGAGCTTGTCTACCTCCTAGCACCATTATTGCAATTAATAAACAACTAACGGACAA
TTCTACCTAACAGTTTTCATATATGACGAGCAGTTAAGTGATGAGTAAAGGTGAGGAATTATTTACTGGTGTTGTTCCGATCT
TAGTTGAACTGGACGGCGATGTTAACGGTCATAAATTCAGTGTTCGTGGTGAAGGTGAAGGTGATGCAACCAACGGTAAGCTG
ACCCTGAAATTCATCTGCACTACTGGAAAATTACCAGTACCGTGGCCTACTCTGGTGACTACCCTGACCTATGGTGTTCAGTG
TTTTTCTCGTTACCCTGACCACATGAAGCAACATGATTTCTTCAAATCTGCAATGCCGGAAGGTTATGTACAGGAGCGCACCA
TTTCTTTCAAAGACGATGGCACGTATAAAACCCGTGCAGAGGTTAAATTTGAAGGTGACACTCTGGTGAATCGTATTGAACTG
AAAGGCATTGATTTCAAAGAGGACGGCAATATTTTAGGCCACAAACTGGAATATAACTTCAACTCCCATAACGTTTACATCAC
CGCAGACAAACAGAAGAACGGTATCAAAGCTAACTTCAAAATTCGCCATAACGTTGAAGATGGTAGCGTACAGCTGGCGGATC
ATTACCAACAGAACACTCCGATTGGAGATGCTCCTGTTTTACTGCCGGATAACCACTACCTGTCCACCCAGTCTAAACTGTCG
AAGGATCCGAACGAAAAGCGCGACCACATGGTGTTATTAGAGTTCGTTACCGCTAGTGGTATCACGCACGGTATGGATGAACT
CTACAAATAAGACGAACAATAAGGGGAGCGGGAAACCGCTCCCCTTTTTTATTGATAACAAAAGTAAATTGCACGCTGATAGT
CTCCCAATTGCGAAGGACCAAAACGAAAAAACACCCTTTCGGGTGTCTTTTCTGGAATTTGGTACCGAGTACTAGGTATCGTG
TAAGTAGCGAAGGCCCGTACGCGAGATAAACTGCTAGGCAACCGCGACTCTACGACTGGTGCTCGATTTAATTTCGCTGACGT
AAAGAAATTATCGGCAGTGCGTCAACTGCCGTATCTTTATCTTAATTAGGTAGTTGGACAAGCCCTTGAAAGAAATAGCAAGA
GCCTGCCTCTCTATTGAAGTCACGGCGAAAGTCGGGTAGAAATCAAAGAAAGCAGAAATTAAATCGGAGTAACACTAAGGTGG
GATAACTCCGTAACTGACTACGCCTTTCTCTAGACTTTACTTGACCAGATACACTGTCTTTGACACGTTGAAGGATTAGAGCA
ATCAAATCCAAGACTGGCTAAGCACGAAGCAACTCTTGAGTGTTAAAAAGTTATCTCCTGTATTCGGGAAGCGGGTACTAGAA
GATTGCAGGGACTCCGACGTTAAGTAAATTACAAAGTAATAAGTATCGTTCAGGATCACGTTACCGCAATAAGAAGCGAGAAT
AATATAATTTCCGAAGTGCTTACCCCAGTAGTGACTATTCCTATAACCCTTCTGAGTGTCCGGAGGCGGAAATTTGCCACGAA
AGAGAAAGTATTTCCCCGACAATAATAAAGGGGCGCTCCTCAGCTTTTCCACTTGGTTGGGTAAGCTAGGCAACTCTGAAAGG
AGTTTCGGCGAATTGAAGCCGACAGCTTTGAATTGTTTTAGGGGCGTTATTCGAGGGCAATCGGAGCTAACTTCAAGACTACT
TCTTTGTTGAATACTAAATAGTGCAAAGGTCGTGTTTCCTCAAGGATACTCCGCTAACAATATAGGATTCCAATCAGATTCAG
CACTGGCGGTACGGGTGTTGCGGTGAGGCGTTCGGGTTTACGGCTCGAAGCTAGCACGGTAGG

p2326

CGCAGGATAAGTAAGGGGAGTAAGTGATCGAACGAATCAGAAGTGACAATATACTTAGGCTGGATCTCGTCCCGTGAATCCCA
ACCCTCACCAACTACGAGATAAGAGGTAAGCCAAAAATCGACTTGGTGGCGACCAACGACTGTTCCCCCCCTGTAACTAATCG
TTCCGTCAAAACCTGACTTACTTCAAGGCCAATTCCAAGCGCAAACAATACCGTCCTAGTTCTTCGGTTAAGTTTCCGAAGTA

GGAGTGAGCCTACCTCCGTTTGCGTCTTGTTACCACTGACCCAGCTATTTACTTTGTATTGCCTGCAATCGAATTTCTGAACT
CTCAGATAGTGGGGATAACGGGAAAGTTCCTATATTTGCGAACTAACTTAGCCGTCCACCTCGAAGCTACCTACTCACACCCA
CCCCGCGCGGGGTAAATAAGGCACTAATCCCAGCTGAGAGCTGGCGTAGCACTTAGCCACAAGTTAATTAACAGTTGTCTGGT
AGTTTGGCGGTATTAGGAAGATCCTAGAAGCAAGGCAGAGTTAGTTCTAACCTAAAGCCACAAATAAGACAGGTTGCCAAAGC
CCGCCGGAAATTAAATCTTGCTCAGTTCGGTAACGGAGTTTCCCTCCCGCGTACTTAATTCCCAATAAGAAACGCGCCCAAGT
CCTATCAGGCAAAATTCAGCCCCTTCCCGTGTTAGAACGAGGGTAAAAATACAAGCCGATTGAACAAGGGTTGGGGGCTTCAA
ATCGTCGTTTACCCCACTTTACAACGGAGATTAAGTAGTTCACCCTATAGTACGAAGCAGAACTATTTCGAGGGGCGTGCAAT
AATCGAATCTTCTGCGGTTGACTTAACACGCTAGGGACGTGCCCTCGATTCAATCGAAGGTACTCCTACTCAGACTGCCTCAC
ACCCAGCTAGTCACTGAGCGATAAAATTGACCCGCCCTCTAGGGAAGCGAGTACGTCCCAAAGGGCTCCGGACAGGGCTATAT
AGGAGAGTTTGATCTCGCCCCGACAACTGCAACCCTCAACTCCCTTAGATAATATTGTTAGCCGAAGTTGCACGACCCGCCGT
CCACGGACTGCTCTTAGGGTGTGGCTCCTTAATCTGACAACGTGCAACCCCTATCGAAGTCGATTGTTTCTGCGAAAGGTGTT
GTCCTAATAGTCCCGAAATTTGGCCCTTGTAGGTGTGAAACCACTTAGCTTCGCGCCGTAGTCCTAAAGGCCCACCTATTGAC
TTTGTTTCGGGTAGCACTAGGAATCTTAACAATTTGAATTTGGACGTGGAACGCGTACACCTTAATCTCCGAATAATTCTAGG
GATTTGGAAGTCCTCTACGTTGACACACCTACACTGCTCGAAGTAAATATACGAATAACGCGGGCCTCGCGGAGCCGTTCCGA
ATCGTCACGTGTTCGTTTACTGTTAATTGGTGGCAAATAAGCAATATCGTAGTCCGTCAGGCCCAGCCCTGTTATCCACGGCG
TTATTTGTCAAATTGCGTAGAACTGGATTGACTGCCTGACAATACCTAATTATCGGTACGAAGTCCCCGAATCTGTCGGGCTA
TTTCACTAATACTTTCCAAACGCCCCGTATCCAAGAAGAACGAATTTATCCACGCTCCCGTCTTTGGGACGAATACCGCTACA
AGTGGACAGAGGATCGGTACGGGCCTCTAATAAATCCAACACTCTACGCCCTCTTCAAGAGCTAGAAGAACAGGGTGCAGTTG
GAAAGGGAATTATTTCGTAAGGCGAGCCAATACCGTAATTAATTCGGAAGAGTTAACACGATTGGAAGTAGGAATAGTTTCTA
ACCACGGTTACTAATCCTAATAACGGAACGCTGTCTGATAGATTAGTGTCAGCGCTCGGTACCAAAGAAAAATAAAAAGACGC
TGAAAAGCGTCTTTTTATTTTTCGGTCCAGTGTAACTCAGGCAAAAGCACGTAATATTCGTACTTTCTTCCTCCGTAAGCGTC
ACCCACATTCCTTAAAGAGTGCATGTGCATATTTTGTTATCAATAAAAAAGGCCGCGATTTGCGGCCTTATTGTTCGTCTTGC
CGGATTACGCCCCGCCCTGCCACTCATCGCAGTATTGTTGTAATTCATTAAGCATTCTGCCGACATGGAAGCCATCACAAACG
GCATGATGAACTTGGATCGCCAGTGGCATTAACACCTTGTCGCCTTGCGTATAATATTTTCCCATAGTGAAAACGGGGGCGAA
GAAGTTGTCCATATTTGCTACGTTTAAATCAAAACTGGTGAAACTCACCCACGGATTGGCACTGACGAAAAACATATTTTCGA
TAAACCCTTTAGGGAAATATGCTAAGTTTTCACCGTAACACGCCACATCTTGACTATATATGTGTAGAAACTGCCGGAAATCG
TCGTGGTATTCTGACCAGAGCGATGAAAACGTTTCAGTTTGCTCATGGAAAACGGTGTAACAAGGGTGAACACTATCCCATAT
CACCAGCTCACCGTCTTTCATTGCCATACGAAACTCCGGATGTGCATTCATCAGGCGGGCAAGAATGTGAATAAAGGCCGGAT
AAAACTTGTGCTTATTTTTCTTTACGGTTTTTAAAAAGGCCGTAATATCCAGCTGAACGGTTTGGTTATAGGTGCACTGAGCA
ACTGACTGGAATGCCTCAAAATGTTCTTTACGATGCCATTGACTTATATCAACTGTAGTATATCCAGTGATTTTTTTCTCCAT
TTTAGCTTCCTTAGCTTGCGAAATCTCGATAACTCAAAAAATAGTAGTGATCTTATTTCATTATGGTGAAAGTTGTCTTACGT
GCAACATTTTCGCAAAAAGTTGGCGCTTTATCAACACTGTCGGAATGACAAATGGTTCCAATTATTGAACACCCTTCGGGGTG
TTTTTTTGTTTCTGGTTTCCCGAGGCCGGCCTGCGCTAGCGGAGTGTATACTGGCTTACTATGTTGGCACTGATGAGGGTGTA
AGTGAAGTGCTTCATGTGGCAGGAGAAAAAAGGCTGCATCGGTGCGTCAGCAGAATATGTGATACAGGATATATTCCGCTTCC
TCGCTCACTGACTCGCTACGCTCGGTCGTTCGACTGTGGCGAGCGGAAATGGCTTACGAACGGGGCGGAGATTTCCTGGAAGA
TGCCAGGAAGATACTTAACAGGGAAGTGAGAGGGTCGCGGCAAAGCCGTTTTTCCATAGGCTCCGCCCCCCTGACAAGCATCA
CGAAATCTGACGCTCAAATCAGTGGTGGCGAAACCTGACAGGACTATAAAGATACCAGGCGTTTCCCCCTGGCGGCTCCCTCG
TGCGCTCTCCTGTTCCTGCCTTTCGGTTTGCCGGTGTCATTCCTCTGTTACGGCCGAGTTTGTCTCATTCCACGCCTGACACT
CAGTTCCGGGTAGGCAGTTCGCTCCAAGCTGGACTGTATGCACGAACCCCCCGTTCAGTCCGACCGCTGCGCCTTATCCGGTA
ACTATCGTCTTGAGTCCAACCCGGAAAGACATGCAAAAGCACCACTGGCAGCAGCCACTGGTAATTGATTTAGAGGAGTTAGT
CTTGAAGTCATGCGCCGGATAAGGCTAAACTGAAAGGACAAGTTTTGGCGACTGCGCTCCTCCAAGCCAGTTACCTCGGTTCA
AAGAGTTGGTAGCTCAGAGAACCTTCGAAAAACCGCCCTGCAAGGCGGTTTTTTCGTTTTCAGAGCAAGAGATTACGCGCAGA
CCAAAACGATCTCAAGAAGATCATCTTATTAATCAGATAAAATATTTCTAGATTTCAGTGCAATTTATCTCTTCAAATGTAGC
ACCGGCGCGCCGTGACCAATTATTGAAGGCCGCTAACGCGGCCTTTTTTTGTTTCTGGTATCCCGAATGGAGCGACTTCTCCC
CAAAAAGCCTCGCTTTCAGCACCTGTCGTTTCCTTTCTTTTCAGAGGGTATTTTAAATAAAAACATTAAGTTATGACGAAGAA
GAACGGAAACGCCTTAAACCGGAAAATTTTCATAAATAGCGAAAACCCGCGAGGTCGCCGCCCCGTAACCTGTCGGATCACCG
GAAAGGACCCGTAAAGTGATAATGATTATCATCTACATATCACAACGTGCGTAAAGGGTAAGTATGAAGGTCGTGTACTCCAT
CGCTACCAAATTCCAGAAAACAGACGCTTTCGAGCGTCTTTTTTCGTTTTGGTCACGACGTACGGTGGAAGATTCGTTACCAA
TTGACAGCTAGCTCAGTCCTAGGTATATACATACATGCTTGTTTGTTTGTAAACTACTAGAAGAGCGATTGGGCTCTTCAATG
AAACGTACTGCCGACGGTTCAGAGTTCGAGTCGCCCAAGAAGAAACGTAAAGTGTCATCCAAAACCGGCCCAGTTGCGGTGGA
CCCAACGCTGCGCCGTCGTATCGAACCGCACGAGTTCGAAGTATTTTTCGATCCTCGCGAACTGCGTAAAGAGACCTGTTTAC
TTTACGAAATCAATTGGGGTGGCCGCCACTCGATTTGGCGCCACACGTCTCAGAATACAAACAAACATGTCGAAGTAAATTTC
ATCGAAAAATTTACAACGGAGCGTTATTTCTGTCCCAATACTCGTTGTTCTATTACATGGTTTTTATCATGGAGTCCCTGCGG
GGAATGCTCTCGTGCGATCACGGAGTTTCTGTCGCGTTATCCGAACGTAACGCTTTTTATCTATATTGCTCGTCTTTACCACT
TGGCTAACCCTCGTAACCGCCAAGGACTTCGCGACCTGATTTCGAGTGGAGTAACCATCCAGATCATGACTGAGCAAGAATCT
GGATACTGTTGGCACAATTTCGTAAACTACTCGCCATCGAATGAGAGCCATTGGCCTCGCTACCCCCATCTTTGGGTACGTTT
GTATGTCTTGGAACTTTATTGTATCATTTTAGGGCTGCCACCTTGTCTTAATATTTTGCGCCGCAAACAGAGTCAGCTGACAT
CTTTCACAATTGCTTTACAGTCATGCCACTACCAACGTCTTCCACCGCACATTCTGTGGGCCACGGGCTTAAAATCGGGCGGT
AGCTCAGGCGGGAGTTCAGGCTCAGAAACGCCGGGAACTAGCGAATCCGCAACGCCAGAGTCTTCAGGTGGTTCGTCAGGTGG
TTCAGACAAGAAATATAGCATCGGCCTGGCCATCGGCACAAATAGCGTCGGATGGGCGGTGATCACTGATGAATATAAAGTTC
CGTCTAAAAAGTTCAAGGTACTGGGTAATACAGATCGCCATAGTATCAAAAAGAACTTAATCGGTGCGCTTCTGTTCGATTCC
GGCGAAACCGCAGAAGCAACACGTCTGAAACGCACCGCTCGTCGCCGTTACACCCGTCGTAAAAACCGCATCTGCTACCTGCA
AGAAATCTTCTCTAACGAAATGGCTAAAGTAGATGACAGCTTTTTTCACCGTCTGGAAGAATCATTTCTGGTGGAAGAAGATA

AAAAGCACGAACGTCATCCAATCTTCGGCAACATTGTGGACGAAGTAGCGTATCACGAAAAATACCCGACTATCTATCACCTG
CGCAAAAAGCTGGTCGATTCGACGGATAAGGCCGATCTGCGTCTGATCTATCTGGCCTTAGCGCATATGATTAAGTTCCGTGG
TCATTTCCTGATCGAAGGCGACCTGAATCCAGACAACAGCGATGTAGACAAACTGTTCATCCAGCTGGTGCAAACCTATAACC
AGCTGTTTGAAGAAAACCCAATTAATGCTAGCGGTGTTGACGCGAAAGCGATCTTGTCCGCACGCCTGTCCAAATCCCGTCGT
CTGGAAAACTTAATTGCGCAACTGCCGGGTGAGAAGAAAAACGGACTGTTCGGCAATCTGATCGCTCTTAGCTTGGGACTGAC
CCCGAACTTCAAAAGCAACTTCGATCTGGCAGAGGACGCAAAACTTCAACTTAGCAAAGATACGTATGACGATGACTTGGATA
ACTTACTGGCCCAGATCGGAGATCAGTACGCTGATCTGTTTCTGGCGGCAAAGAACTTATCAGACGCTATTCTCCTGTCTGAT
ATTCTTCGTGTGAATACCGAAATCACCAAAGCACCGCTTTCTGCATCCATGATTAAACGCTATGACGAACATCACCAAGATCT
GACTCTTCTGAAAGCGCTGGTACGGCAACAACTGCCGGAGAAGTACAAGGAGATCTTCTTTGACCAATCCAAAAACGGCTACG
CGGGTTATATTGACGGGGGTGCAAGCCAAGAGGAGTTCTACAAATTCATCAAGCCAATCTTAGAAAAAATGGATGGCACGGAA
GAATTACTTGTTAAACTGAATCGTGAGGATCTGCTTCGTAAACAGCGTACCTTCGACAACGGTAGCATTCCGCACCAGATCCA
CTTAGGTGAACTGCACGCTATCCTGCGTCGCCAAGAGGATTTTTACCCGTTCCTGAAAGATAATCGTGAAAAAATCGAAAAAA
TCCTGACCTTTCGTATCCCGTATTATGTCGGCCCGCTGGCGCGTGGCAACTCCCGTTTCGCGTGGATGACTCGCAAATCCGAA
GAAACTATTACCCCGTGGAACTTCGAGGAAGTGGTTGACAAAGGCGCAAGCGCCCAATCCTTCATCGAGCGCATGACTAACTT
TGATAAAAACCTGCCGAACGAAAAGGTACTGCCGAAACACTCCCTTCTGTACGAATACTTCACCGTGTACAACGAGCTGACTA
AAGTAAAGTATGTGACTGAGGGCATGCGTAAACCTGCATTCCTGAGCGGTGAACAGAAAAAAGCAATTGTTGATTTACTGTTT
AAAACCAACCGTAAAGTAACCGTTAAACAGCTGAAAGAGGACTACTTCAAGAAAATCGAATGCTTCGACTCCGTCGAGATTAG
TGGAGTTGAAGATCGTTTTAATGCAAGTTTAGGCACGTATCACGATTTATTAAAGATCATTAAAGACAAAGATTTCTTGGACA
ACGAAGAAAATGAGGACATCTTAGAGGACATCGTCCTGACCCTGACTCTGTTCGAAGATCGTGAAATGATTGAAGAACGCCTT
AAGACGTATGCTCACCTGTTTGACGATAAAGTAATGAAACAACTGAAACGTCGCCGTTATACTGGCTGGGGCCGTCTGAGCCG
TAAACTGATTAACGGTATCCGTGACAAACAGTCCGGTAAAACTATTCTGGACTTCCTGAAATCTGACGGCTTCGCAAACCGTA
ACTTCATGCAACTGATTCACGACGATTCCCTGACCTTCAAAGAGGACATCCAGAAAGCTCAGGTTTCTGGTCAAGGTGATTCT
CTGCACGAGCATATCGCCAATTTAGCAGGTAGTCCGGCGATCAAAAAAGGTATCCTGCAAACCGTGAAAGTGGTGGATGAGCT
TGTGAAAGTTATGGGTCGTCACAAACCGGAAAACATTGTTATCGAGATGGCTCGTGAAAACCAAACGACCCAGAAGGGACAGA
AAAACTCCCGCGAACGCATGAAACGTATCGAGGAGGGTATTAAAGAACTTGGCTCTCAGATTCTGAAAGAACACCCTGTTGAA
AATACCCAACTGCAAAATGAAAAACTGTACCTGTACTACCTGCAAAATGGTCGTGACATGTATGTAGATCAGGAGCTGGACAT
CAACCGCCTCTCCGATTACGACGTTGACCACATTGTTCCGCAGTCTTTTCTGAAAGATGATTCCATTGATAACAAAGTACTCA
CCCGTAGCGATAAAAACCGTGGGAAGAGTGACAACGTTCCATCGGAAGAAGTAGTTAAGAAAATGAAGAACTATTGGCGTCAA
CTGCTTAACGCGAAACTGATTACTCAACGTAAATTTGATAACCTGACCAAAGCTGAACGTGGCGGTTTGTCTGAGCTGGATAA
GGCGGGTTTTATTAAACGTCAACTGGTAGAAACTCGCCAGATTACAAAACATGTTGCTCAGATTCTGGACTCTCGTATGAACA
CTAAATACGATGAAAATGACAAACTGATCCGCGAAGTTAAGGTTATTACCCTGAAATCTAAGCTGGTTTCCGACTTCCGTAAA
GATTTCCAATTCTATAAAGTGCGCGAGATTAACAACTATCACCACGCGCACGACGCATATCTGAATGCAGTTGTTGGCACGGC
ACTGATCAAAAAATATCCGAAACTGGAAAGCGAATTTGTGTACGGCGATTATAAAGTTTACGACGTGCGCAAAATGATCGCCA
AATCTGAACAGGAAATTGGCAAAGCAACCGCTAAATACTTTTTCTACTCAAACATTATGAATTTCTTCAAAACCGAAATCACC
TTAGCGAATGGCGAAATTCGTAAACGCCCTCTGATCGAAACCAACGGCGAAACGGGTGAGATCGTGTGGGACAAAGGTCGTGA
TTTCGCTACTGTCCGCAAAGTTCTGTCCATGCCTCAAGTAAACATCGTTAAAAAGACTGAGGTACAGACTGGCGGTTTCAGCA
AGGAATCCATTCTGCCGAAACGCAACTCCGACAAACTGATCGCGCGTAAGAAAGACTGGGATCCGAAGAAATACGGTGGCTTC
GATTCTCCAACCGTGGCATACAGCGTTCTGGTAGTCGCCAAAGTCGAAAAGGGTAAATCAAAAAAACTGAAATCAGTGAAAGA
ACTTTTAGGCATCACCATTATGGAACGTAGCTCTTTCGAAAAAAACCCGATTGACTTCCTCGAAGCGAAGGGGTACAAGGAAG
TAAAGAAAGATCTGATTATCAAACTGCCGAAGTATTCCCTGTTCGAACTGGAAAATGGTCGTAAACGTATGTTAGCGTCTGCG
GGTGAACTGCAAAAAGGGAACGAATTGGCCCTTCCGTCCAAGTACGTGAACTTCCTGTATCTGGCCTCGCACTACGAGAAACT
GAAAGGTAGTCCGGAAGATAATGAGCAGAAACAGCTGTTCGTGGAACAGCACAAACACTATCTGGACGAGATTATTGAACAGA
TTTCTGAGTTTAGCAAACGCGTAATTCTGGCGGACGCGAATCTGGATAAAGTCCTGAGCGCCTACAATAAACACCGTGATAAA
CCGATCCGTGAACAGGCAGAAAACATCATTCACCTGTTCACGCTGACTAATCTTGGTGCTCCGGCAGCCTTCAAATACTTCGA
CACCACGATCGATCGTAAACGTTACACCTCCACTAAAGAAGTCTTAGATGCAACTCTTATTCACCAGAGCATCACTGGCCTGT
ATGAAACTCGTATTGATCTGAGTCAGTTGGGCGGTGACAGTGGGGGCAGCGGAGGTAGCGGAGGCAGCACTAATTTGTCGGAT
ATTATTGAGAAGGAAACAGGAAAGCAATTGGTTATCCAGGAATCCATTTTAATGCTTCCGGAAGAAGTGGAAGAGGTGATCGG
AAATAAGCCAGAGAGCGATATCTTAGTTCATACGGCCTATGATGAAAGCACTGACGAGAACGTTATGCTGTTAACATCGGACG
CTCCCGAGTACAAGCCGTGGGCCCTGGTTATCCAGGACAGTAATGGCGAAAATAAAATTAAAATGTTATAATGTATGCTTAAG
CAGCTCGGTACCAAAGACGAACAATAAGACGCTGAAAAGCGTCTTTTTTCGTTTTGGTCCTGTTGCGGCGCGATAGTGTGAAC
ATGCTATAGACTTCTGGTGCTACCCGACTGACAATTAATCATCCGGCTCGTATAATGCTAGCTGAAGAGCTTCATCGCTCTTC
AGTTTTAGAGCTAGAAATAGCAAGTTAAAATAAGGCTAGTCCGTTATCAACTTGAAAAAGTGGCACCGAGTCGGTGCTTTTTT
TAATCCATTCGAATCACGTCCGACGAACAATAAGGCCTCCCTAACGGGGGGCCTTTTTTATTGATAACAAAAGTAACTTCGAG
CTTGTCTACCTCCTAGCACCATTATTGCAATTAATAAACAACTAACGGACAATTCTACCTAACAGTTTTCATATATGACGAGC
AGTTAAGTGATGAGTAAAGGTGAGGAATTATTTACTGGTGTTGTTCCGATCTTAGTTGAACTGGACGGCGATGTTAACGGTCA
TAAATTCAGTGTTCGTGGTGAAGGTGAAGGTGATGCAACCAACGGTAAGCTGACCCTGAAATTCATCTGCACTACTGGAAAAT
TACCAGTACCGTGGCCTACTCTGGTGACTACCCTGACCTATGGTGTTCAGTGTTTTTCTCGTTACCCTGACCACATGAAGCAA
CATGATTTCTTCAAATCTGCAATGCCGGAAGGTTATGTACAGGAGCGCACCATTTCTTTCAAAGACGATGGCACGTATAAAAC
CCGTGCAGAGGTTAAATTTGAAGGTGACACTCTGGTGAATCGTATTGAACTGAAAGGCATTGATTTCAAAGAGGACGGCAATA
TTTTAGGCCACAAACTGGAATATAACTTCAACTCCCATAACGTTTACATCACCGCAGACAAACAGAAGAACGGTATCAAAGCT
AACTTCAAAATTCGCCATAACGTTGAAGATGGTAGCGTACAGCTGGCGGATCATTACCAACAGAACACTCCGATTGGAGATGC
TCCTGTTTTACTGCCGGATAACCACTACCTGTCCACCCAGTCTAAACTGTCGAAGGATCCGAACGAAAAGCGCGACCACATGG
TGTTATTAGAGTTCGTTACCGCTAGTGGTATCACGCACGGTATGGATGAACTCTACAAATAAGACGAACAATAAGGGGAGCGG

GAAACCGCTCCCCTTTTTTATTGATAACAAAAGTAAATTGCACGCTGATAGTCTCCCAATTGCGAAGGACCAAAACGAAAAAA
CACCCTTTCGGGTGTCTTTTCTGGAATTTGGTACCGAGTACTAGGTATCGTGTAAGTAGCGAAGGCCCGTACGCGAGATAAAC
TGCTAGGCAACCGCGACTCTACGACTGGTGCTCGATTTAATTTCGCTGACGTAAAGAAATTATCGGCAGTGCGTCAACTGCCG
TATCTTTATCTTAATTAGGTAGTTGGACAAGCCCTTGAAAGAAATAGCAAGAGCCTGCCTCTCTATTGAAGTCACGGCGAAAG
TCGGGTAGAAATCAAAGAAAGCAGAAATTAAATCGGAGTAACACTAAGGTGGGATAACTCCGTAACTGACTACGCCTTTCTCT
AGACTTTACTTGACCAGATACACTGTCTTTGACACGTTGAAGGATTAGAGCAATCAAATCCAAGACTGGCTAAGCACGAAGCA
ACTCTTGAGTGTTAAAAAGTTATCTCCTGTATTCGGGAAGCGGGTACTAGAAGATTGCAGGGACTCCGACGTTAAGTAAATTA
CAAAGTAATAAGTATCGTTCAGGATCACGTTACCGCAATAAGAAGCGAGAATAATATAATTTCCGAAGTGCTTACCCCAGTAG
TGACTATTCCTATAACCCTTCTGAGTGTCCGGAGGCGGAAATTTGCCACGAAAGAGAAAGTATTTCCCCGACAATAATAAAGG
GGCGCTCCTCAGCTTTTCCACTTGGTTGGGTAAGCTAGGCAACTCTGAAAGGAGTTTCGGCGAATTGAAGCCGACAGCTTTGA
ATTGTTTTAGGGGCGTTATTCGAGGGCAATCGGAGCTAACTTCAAGACTACTTCTTTGTTGAATACTAAATAGTGCAAAGGTC
GTGTTTCCTCAAGGATACTCCGCTAACAATATAGGATTCCAATCAGATTCAGCACTGGCGGTACGGGTGTTGCGGTGAGGCGT
TCGGGTTTACGGCTCGAAGCTAGCACGGTAGG

p2327

CGCAGGATAAGTAAGGGGAGTAAGTGATCGAACGAATCAGAAGTGACAATATACTTAGGCTGGATCTCGTCCCGTGAATCCCA
ACCCTCACCAACTACGAGATAAGAGGTAAGCCAAAAATCGACTTGGTGGCGACCAACGACTGTTCCCCCCCTGTAACTAATCG
TTCCGTCAAAACCTGACTTACTTCAAGGCCAATTCCAAGCGCAAACAATACCGTCCTAGTTCTTCGGTTAAGTTTCCGAAGTA
GGAGTGAGCCTACCTCCGTTTGCGTCTTGTTACCACTGACCCAGCTATTTACTTTGTATTGCCTGCAATCGAATTTCTGAACT
CTCAGATAGTGGGGATAACGGGAAAGTTCCTATATTTGCGAACTAACTTAGCCGTCCACCTCGAAGCTACCTACTCACACCCA
CCCCGCGCGGGGTAAATAAGGCACTAATCCCAGCTGAGAGCTGGCGTAGCACTTAGCCACAAGTTAATTAACAGTTGTCTGGT
AGTTTGGCGGTATTAGGAAGATCCTAGAAGCAAGGCAGAGTTAGTTCTAACCTAAAGCCACAAATAAGACAGGTTGCCAAAGC
CCGCCGGAAATTAAATCTTGCTCAGTTCGGTAACGGAGTTTCCCTCCCGCGTACTTAATTCCCAATAAGAAACGCGCCCAAGT
CCTATCAGGCAAAATTCAGCCCCTTCCCGTGTTAGAACGAGGGTAAAAATACAAGCCGATTGAACAAGGGTTGGGGGCTTCAA
ATCGTCGTTTACCCCACTTTACAACGGAGATTAAGTAGTTCACCCTATAGTACGAAGCAGAACTATTTCGAGGGGCGTGCAAT
AATCGAATCTTCTGCGGTTGACTTAACACGCTAGGGACGTGCCCTCGATTCAATCGAAGGTACTCCTACTCAGACTGCCTCAC
ACCCAGCTAGTCACTGAGCGATAAAATTGACCCGCCCTCTAGGGAAGCGAGTACGTCCCAAAGGGCTCCGGACAGGGCTATAT
AGGAGAGTTTGATCTCGCCCCGACAACTGCAACCCTCAACTCCCTTAGATAATATTGTTAGCCGAAGTTGCACGACCCGCCGT
CCACGGACTGCTCTTAGGGTGTGGCTCCTTAATCTGACAACGTGCAACCCCTATCGAAGTCGATTGTTTCTGCGAAAGGTGTT
GTCCTAATAGTCCCGAAATTTGGCCCTTGTAGGTGTGAAACCACTTAGCTTCGCGCCGTAGTCCTAAAGGCCCACCTATTGAC
TTTGTTTCGGGTAGCACTAGGAATCTTAACAATTTGAATTTGGACGTGGAACGCGTACACCTTAATCTCCGAATAATTCTAGG
GATTTGGAAGTCCTCTACGTTGACACACCTACACTGCTCGAAGTAAATATACGAATAACGCGGGCCTCGCGGAGCCGTTCCGA
ATCGTCACGTGTTCGTTTACTGTTAATTGGTGGCAAATAAGCAATATCGTAGTCCGTCAGGCCCAGCCCTGTTATCCACGGCG
TTATTTGTCAAATTGCGTAGAACTGGATTGACTGCCTGACAATACCTAATTATCGGTACGAAGTCCCCGAATCTGTCGGGCTA
TTTCACTAATACTTTCCAAACGCCCCGTATCCAAGAAGAACGAATTTATCCACGCTCCCGTCTTTGGGACGAATACCGCTACA
AGTGGACAGAGGATCGGTACGGGCCTCTAATAAATCCAACACTCTACGCCCTCTTCAAGAGCTAGAAGAACAGGGTGCAGTTG
GAAAGGGAATTATTTCGTAAGGCGAGCCAATACCGTAATTAATTCGGAAGAGTTAACACGATTGGAAGTAGGAATAGTTTCTA
ACCACGGTTACTAATCCTAATAACGGAACGCTGTCTGATAGATTAGTGTCAGCGCTCGGTACCAAAGAAAAATAAAAAGACGC
TGAAAAGCGTCTTTTTATTTTTCGGTCCAGTGTAACTCAGGCAAAAGCACGTAATATTCGTACTTTCTTCCTCCGTAAGCGTC
ACCCACATTCCTTAAAGAGTGCATGTGCATATTTTGTTATCAATAAAAAAGGCCGCGATTTGCGGCCTTATTGTTCGTCTTGC
CGGATTACGCCCCGCCCTGCCACTCATCGCAGTATTGTTGTAATTCATTAAGCATTCTGCCGACATGGAAGCCATCACAAACG
GCATGATGAACTTGGATCGCCAGTGGCATTAACACCTTGTCGCCTTGCGTATAATATTTTCCCATAGTGAAAACGGGGGCGAA
GAAGTTGTCCATATTTGCTACGTTTAAATCAAAACTGGTGAAACTCACCCACGGATTGGCACTGACGAAAAACATATTTTCGA
TAAACCCTTTAGGGAAATATGCTAAGTTTTCACCGTAACACGCCACATCTTGACTATATATGTGTAGAAACTGCCGGAAATCG
TCGTGGTATTCTGACCAGAGCGATGAAAACGTTTCAGTTTGCTCATGGAAAACGGTGTAACAAGGGTGAACACTATCCCATAT
CACCAGCTCACCGTCTTTCATTGCCATACGAAACTCCGGATGTGCATTCATCAGGCGGGCAAGAATGTGAATAAAGGCCGGAT
AAAACTTGTGCTTATTTTTCTTTACGGTTTTTAAAAAGGCCGTAATATCCAGCTGAACGGTTTGGTTATAGGTGCACTGAGCA
ACTGACTGGAATGCCTCAAAATGTTCTTTACGATGCCATTGACTTATATCAACTGTAGTATATCCAGTGATTTTTTTCTCCAT
TTTAGCTTCCTTAGCTTGCGAAATCTCGATAACTCAAAAAATAGTAGTGATCTTATTTCATTATGGTGAAAGTTGTCTTACGT
GCAACATTTTCGCAAAAAGTTGGCGCTTTATCAACACTGTCGGAATGACAAATGGTTCCAATTATTGAACACCCTTCGGGGTG
TTTTTTTGTTTCTGGTTTCCCGAGGCCGGCCTGCGCTAGCGGAGTGTATACTGGCTTACTATGTTGGCACTGATGAGGGTGTA
AGTGAAGTGCTTCATGTGGCAGGAGAAAAAAGGCTGCATCGGTGCGTCAGCAGAATATGTGATACAGGATATATTCCGCTTCC
TCGCTCACTGACTCGCTACGCTCGGTCGTTCGACTGTGGCGAGCGGAAATGGCTTACGAACGGGGCGGAGATTTCCTGGAAGA
TGCCAGGAAGATACTTAACAGGGAAGTGAGAGGGTCGCGGCAAAGCCGTTTTTCCATAGGCTCCGCCCCCCTGACAAGCATCA
CGAAATCTGACGCTCAAATCAGTGGTGGCGAAACCTGACAGGACTATAAAGATACCAGGCGTTTCCCCCTGGCGGCTCCCTCG
TGCGCTCTCCTGTTCCTGCCTTTCGGTTTGCCGGTGTCATTCCTCTGTTACGGCCGAGTTTGTCTCATTCCACGCCTGACACT
CAGTTCCGGGTAGGCAGTTCGCTCCAAGCTGGACTGTATGCACGAACCCCCCGTTCAGTCCGACCGCTGCGCCTTATCCGGTA
ACTATCGTCTTGAGTCCAACCCGGAAAGACATGCAAAAGCACCACTGGCAGCAGCCACTGGTAATTGATTTAGAGGAGTTAGT
CTTGAAGTCATGCGCCGGATAAGGCTAAACTGAAAGGACAAGTTTTGGCGACTGCGCTCCTCCAAGCCAGTTACCTCGGTTCA
AAGAGTTGGTAGCTCAGAGAACCTTCGAAAAACCGCCCTGCAAGGCGGTTTTTTCGTTTTCAGAGCAAGAGATTACGCGCAGA
CCAAAACGATCTCAAGAAGATCATCTTATTAATCAGATAAAATATTTCTAGATTTCAGTGCAATTTATCTCTTCAAATGTAGC

ACCGGCGCGCCGTGACCAATTATTGAAGGCCGCTAACGCGGCCTTTTTTTGTTTCTGGTATCCCGAATGGAGCGACTTCTCCC
CAAAAAGCCTCGCTTTCAGCACCTGTCGTTTCCTTTCTTTTCAGAGGGTATTTTAAATAAAAACATTAAGTTATGACGAAGAA
GAACGGAAACGCCTTAAACCGGAAAATTTTCATAAATAGCGAAAACCCGCGAGGTCGCCGCCCCGTAACCTGTCGGATCACCG
GAAAGGACCCGTAAAGTGATAATGATTATCATCTACATATCACAACGTGCGTAAAGGGTAAGTATGAAGGTCGTGTACTCCAT
CGCTACCAAATTCCAGAAAACAGACGCTTTCGAGCGTCTTTTTTCGTTTTGGTCACGACGTACGGTGGAAGATTCGTTACCAA
TTGACAGCTAGCTCAGTCCTAGGTATATACATACATGCTTGTTTGTTTGTAAACCAGGATAAAGAGAGAGCATACTCGATGAA
ACGTACTGCCGACGGTTCAGAGTTCGAGTCGCCCAAGAAGAAACGTAAAGTGTCATCCAAAACCGGCCCAGTTGCGGTGGACC
CAACGCTGCGCCGTCGTATCGAACCGCACGAGTTCGAAGTATTTTTCGATCCTCGCGAACTGCGTAAAGAGACCTGTTTACTT
TACGAAATCAATTGGGGTGGCCGCCACTCGATTTGGCGCCACACGTCTCAGAATACAAACAAACATGTCGAAGTAAATTTCAT
CGAAAAATTTACAACGGAGCGTTATTTCTGTCCCAATACTCGTTGTTCTATTACATGGTTTTTATCATGGAGTCCCTGCGGGG
AATGCTCTCGTGCGATCACGGAGTTTCTGTCGCGTTATCCGAACGTAACGCTTTTTATCTATATTGCTCGTCTTTACCACTTG
GCTAACCCTCGTAACCGCCAAGGACTTCGCGACCTGATTTCGAGTGGAGTAACCATCCAGATCATGACTGAGCAAGAATCTGG
ATACTGTTGGCACAATTTCGTAAACTACTCGCCATCGAATGAGAGCCATTGGCCTCGCTACCCCCATCTTTGGGTACGTTTGT
ATGTCTTGGAACTTTATTGTATCATTTTAGGGCTGCCACCTTGTCTTAATATTTTGCGCCGCAAACAGAGTCAGCTGACATCT
TTCACAATTGCTTTACAGTCATGCCACTACCAACGTCTTCCACCGCACATTCTGTGGCCACGGGCTTAAAATCGGGCGGTAG
CTCAGGCGGGAGTTCAGGCTCAGAAACGCCGGGAACTAGCGAATCCGCAACGCCAGAGTCTTCAGGTGGTTCGTCAGGTGGTT
CAGACAAGAAATATAGCATCGGCCTGGCCATCGGCACAAATAGCGTCGGATGGGCGGTGATCACTGATGAATATAAAGTTCCG
TCTAAAAAGTTCAAGGTACTGGGTAATACAGATCGCCATAGTATCAAAAAGAACTTAATCGGTGCGCTTCTGTTCGATTCCGG
CGAAACCGCAGAAGCAACACGTCTGAAACGCACCGCTCGTCGCCGTTACACCCGTCGTAAAAACCGCATCTGCTACCTGCAAG
AAATCTTCTCTAACGAAATGGCTAAAGTAGATGACAGCTTTTTTCACCGTCTGGAAGAATCATTTCTGGTGGAAGAAGATAAA
AAGCACGAACGTCATCCAATCTTCGGCAACATTGTGGACGAAGTAGCGTATCACGAAAAATACCCGACTATCTATCACCTGCG
CAAAAAGCTGGTCGATTCGACGGATAAGGCCGATCTGCGTCTGATCTATCTGGCCTTAGCGCATATGATTAAGTTCCGTGGTC
ATTTCCTGATCGAAGGCGACCTGAATCCAGACAACAGCGATGTAGACAAACTGTTCATCCAGCTGGTGCAAACCTATAACCAG
CTGTTTGAAGAAAACCCAATTAATGCTAGCGGTGTTGACGCGAAAGCGATCTTGTCCGCACGCCTGTCCAAATCCCGTCGTCT
GGAAAACTTAATTGCGCAACTGCCGGGTGAGAAGAAAAACGGACTGTTCGGCAATCTGATCGCTCTTAGCTTGGGACTGACCC
CGAACTTCAAAAGCAACTTCGATCTGGCAGAGGACGCAAAACTTCAACTTAGCAAAGATACGTATGACGATGACTTGGATAAC
TTACTGGCCCAGATCGGAGATCAGTACGCTGATCTGTTTCTGGCGGCAAAGAACTTATCAGACGCTATTCTCCTGTCTGATAT
TCTTCGTGTGAATACCGAAATCACCAAAGCACCGCTTTCTGCATCCATGATTAAACGCTATGACGAACATCACCAAGATCTGA
CTCTTCTGAAAGCGCTGGTACGGCAACAACTGCCGGAGAAGTACAAGGAGATCTTCTTTGACCAATCCAAAAACGGCTACGCG
GGTTATATTGACGGGGGTGCAAGCCAAGAGGAGTTCTACAAATTCATCAAGCCAATCTTAGAAAAAATGGATGGCACGGAAGA
ATTACTTGTTAAACTGAATCGTGAGGATCTGCTTCGTAAACAGCGTACCTTCGACAACGGTAGCATTCCGCACCAGATCCACT
TAGGTGAACTGCACGCTATCCTGCGTCGCCAAGAGGATTTTTACCCGTTCCTGAAAGATAATCGTGAAAAAATCGAAAAAATC
CTGACCTTTCGTATCCCGTATTATGTCGGCCCGCTGGCGCGTGGCAACTCCCGTTTCGCGTGGATGACTCGCAAATCCGAAGA
AACTATTACCCCGTGGAACTTCGAGGAAGTGGTTGACAAAGGCGCAAGCGCCCAATCCTTCATCGAGCGCATGACTAACTTTG
ATAAAAACCTGCCGAACGAAAAGGTACTGCCGAAACACTCCCTTCTGTACGAATACTTCACCGTGTACAACGAGCTGACTAAA
GTAAAGTATGTGACTGAGGGCATGCGTAAACCTGCATTCCTGAGCGGTGAACAGAAAAAAGCAATTGTTGATTTACTGTTTAA
AACCAACCGTAAAGTAACCGTTAAACAGCTGAAAGAGGACTACTTCAAGAAAATCGAATGCTTCGACTCCGTCGAGATTAGTG
GAGTTGAAGATCGTTTTAATGCAAGTTTAGGCACGTATCACGATTTATTAAAGATCATTAAAGACAAAGATTTCTTGGACAAC
GAAGAAAATGAGGACATCTTAGAGGACATCGTCCTGACCCTGACTCTGTTCGAAGATCGTGAAATGATTGAAGAACGCCTTAA
GACGTATGCTCACCTGTTTGACGATAAAGTAATGAAACAACTGAAACGTCGCCGTTATACTGGCTGGGGCCGTCTGAGCCGTA
AACTGATTAACGGTATCCGTGACAAACAGTCCGGTAAAACTATTCTGGACTTCCTGAAATCTGACGGCTTCGCAAACCGTAAC
TTCATGCAACTGATTCACGACGATTCCCTGACCTTCAAAGAGGACATCCAGAAAGCTCAGGTTTCTGGTCAAGGTGATTCTCT
GCACGAGCATATCGCCAATTTAGCAGGTAGTCCGGCGATCAAAAAAGGTATCCTGCAAACCGTGAAAGTGGTGGATGAGCTTG
TGAAAGTTATGGGTCGTCACAAACCGGAAAACATTGTTATCGAGATGGCTCGTGAAAACCAAACGACCCAGAAGGGACAGAAA
AACTCCCGCGAACGCATGAAACGTATCGAGGAGGGTATTAAAGAACTTGGCTCTCAGATTCTGAAAGAACACCCTGTTGAAAA
TACCCAACTGCAAAATGAAAAACTGTACCTGTACTACCTGCAAAATGGTCGTGACATGTATGTAGATCAGGAGCTGGACATCA
ACCGCCTCTCCGATTACGACGTTGACCACATTGTTCCGCAGTCTTTTCTGAAAGATGATTCCATTGATAACAAAGTACTCACC
CGTAGCGATAAAAACCGTGGGAAGAGTGACAACGTTCCATCGGAAGAAGTAGTTAAGAAAATGAAGAACTATTGGCGTCAACT
GCTTAACGCGAAACTGATTACTCAACGTAAATTTGATAACCTGACCAAAGCTGAACGTGGCGGTTTGTCTGAGCTGGATAAGG
CGGGTTTTATTAAACGTCAACTGGTAGAAACTCGCCAGATTACAAAACATGTTGCTCAGATTCTGGACTCTCGTATGAACACT
AAATACGATGAAAATGACAAACTGATCCGCGAAGTTAAGGTTATTACCCTGAAATCTAAGCTGGTTTCCGACTTCCGTAAAGA
TTTCCAATTCTATAAAGTGCGCGAGATTAACAACTATCACCACGCGCACGACGCATATCTGAATGCAGTTGTTGGCACGGCAC
TGATCAAAAAATATCCGAAACTGGAAAGCGAATTTGTGTACGGCGATTATAAAGTTTACGACGTGCGCAAAATGATCGCCAAA
TCTGAACAGGAAATTGGCAAAGCAACCGCTAAATACTTTTTCTACTCAAACATTATGAATTTCTTCAAAACCGAAATCACCTT
AGCGAATGGCGAAATTCGTAAACGCCCTCTGATCGAAACCAACGGCGAAACGGGTGAGATCGTGTGGGACAAAGGTCGTGATT
TCGCTACTGTCCGCAAAGTTCTGTCCATGCCTCAAGTAAACATCGTTAAAAAGACTGAGGTACAGACTGGCGGTTTCAGCAAG
GAATCCATTCTGCCGAAACGCAACTCCGACAAACTGATCGCGCGTAAGAAAGACTGGGATCCGAAGAAATACGGTGGCTTCGA
TTCTCCAACCGTGGCATACAGCGTTCTGGTAGTCGCCAAAGTCGAAAAGGGTAAATCAAAAAAACTGAAATCAGTGAAAGAAC
TTTTAGGCATCACCATTATGGAACGTAGCTCTTTCGAAAAAAACCCGATTGACTTCCTCGAAGCGAAGGGGTACAAGGAAGTA
AAGAAAGATCTGATTATCAAACTGCCGAAGTATTCCCTGTTCGAACTGGAAAATGGTCGTAAACGTATGTTAGCGTCTGCGGG
TGAACTGCAAAAAGGGAACGAATTGGCCCTTCCGTCCAAGTACGTGAACTTCCTGTATCTGGCCTCGCACTACGAGAAACTGA
AAGGTAGTCCGGAAGATAATGAGCAGAAACAGCTGTTCGTGGAACAGCACAAACACTATCTGGACGAGATTATTGAACAGATT
TCTGAGTTTAGCAAACGCGTAATTCTGGCGGACGCGAATCTGGATAAAGTCCTGAGCGCCTACAATAAACACCGTGATAAACC

GATCCGTGAACAGGCAGAAAACATCATTCACCTGTTCACGCTGACTAATCTTGGTGCTCCGGCAGCCTTCAAATACTTCGACA
CCACGATCGATCGTAAACGTTACACCTCCACTAAAGAAGTCTTAGATGCAACTCTTATTCACCAGAGCATCACTGGCCTGTAT
GAAACTCGTATTGATCTGAGTCAGTTGGGCGGTGACAGTGGGGGCAGCGGAGGTAGCGGAGGCAGCACTAATTTGTCGGATAT
TATTGAGAAGGAAACAGGAAAGCAATTGGTTATCCAGGAATCCATTTTAATGCTTCCGGAAGAAGTGGAAGAGGTGATCGGAA
ATAAGCCAGAGAGCGATATCTTAGTTCATACGGCCTATGATGAAAGCACTGACGAGAACGTTATGCTGTTAACATCGGACGCT
CCCGAGTACAAGCCGTGGGCCCTGGTTATCCAGGACAGTAATGGCGAAAATAAAATTAAAATGTTATAATGTATGCTTAAGCA
GCTCGGTACCAAAGACGAACAATAAGACGCTGAAAAGCGTCTTTTTTCGTTTTGGTCCTGTTGCGGCGCGATAGTGTGAACAT
GCTATAGACTTCTGGTGCTACCCGACTGACAATTAATCATCCGGCTCGTATAATGCTAGCCTCGTCAACAATTAATAGACGTT
TTAGAGCTAGAAATAGCAAGTTAAAATAAGGCTAGTCCGTTATCAACTTGAAAAAGTGGCACCGAGTCGGTGCTTTTTTTAAT
CCATTCGAATCACGTCCGACGAACAATAAGGCCTCCCTAACGGGGGGCCTTTTTTATTGATAACAAAAGTAACTTCGAGCTTG
TCTACCTCCTAGCACCATTATTGCAATTAATAAACAACTAACGGACAATTCTACCTAACAGTTTTCATATATGACGAGCAGTT
AAGTGATGAGTAAAGGTGAGGAATTATTTACTGGTGTTGTTCCGATCTTAGTTGAACTGGACGGCGATGTTAACGGTCATAAA
TTCAGTGTTCGTGGTGAAGGTGAAGGTGATGCAACCAACGGTAAGCTGACCCTGAAATTCATCTGCACTACTGGAAAATTACC
AGTACCGTGGCCTACTCTGGTGACTACCCTGACCTATGGTGTTCAGTGTTTTTCTCGTTACCCTGACCACATGAAGCAACATG
ATTTCTTCAAATCTGCAATGCCGGAAGGTTATGTACAGGAGCGCACCATTTCTTTCAAAGACGATGGCACGTATAAAACCCGT
GCAGAGGTTAAATTTGAAGGTGACACTCTGGTGAATCGTATTGAACTGAAAGGCATTGATTTCAAAGAGGACGGCAATATTTT
AGGCCACAAACTGGAATATAACTTCAACTCCCATAACGTTTACATCACCGCAGACAAACAGAAGAACGGTATCAAAGCTAACT
TCAAAATTCGCCATAACGTTGAAGATGGTAGCGTACAGCTGGCGGATCATTACCAACAGAACACTCCGATTGGAGATGCTCCT
GTTTTACTGCCGGATAACCACTACCTGTCCACCCAGTCTAAACTGTCGAAGGATCCGAACGAAAAGCGCGACCACATGGTGTT
ATTAGAGTTCGTTACCGCTAGTGGTATCACGCACGGTATGGATGAACTCTACAAATAAGACGAACAATAAGGGGAGCGGGAAA
CCGCTCCCCTTTTTTATTGATAACAAAAGTAAATTGCACGCTGATAGTCTCCCAATTGCGAAGGACCAAAACGAAAAAACACC
CTTTCGGGTGTCTTTTCTGGAATTTGGTACCGAGTACTAGGTATCGTGTAAGTAGCGAAGGCCCGTACGCGAGATAAACTGCT
AGGCAACCGCGACTCTACGACTGGTGCTCGATTTAATTTCGCTGACGTAAAGAAATTATCGGCAGTGCGTCAACTGCCGTATC
TTTATCTTAATTAGGTAGTTGGACAAGCCCTTGAAAGAAATAGCAAGAGCCTGCCTCTCTATTGAAGTCACGGCGAAAGTCGG
GTAGAAATCAAAGAAAGCAGAAATTAAATCGGAGTAACACTAAGGTGGGATAACTCCGTAACTGACTACGCCTTTCTCTAGAC
TTTACTTGACCAGATACACTGTCTTTGACACGTTGAAGGATTAGAGCAATCAAATCCAAGACTGGCTAAGCACGAAGCAACTC
TTGAGTGTTAAAAAGTTATCTCCTGTATTCGGGAAGCGGGTACTAGAAGATTGCAGGGACTCCGACGTTAAGTAAATTACAAA
GTAATAAGTATCGTTCAGGATCACGTTACCGCAATAAGAAGCGAGAATAATATAATTTCCGAAGTGCTTACCCCAGTAGTGAC
TATTCCTATAACCCTTCTGAGTGTCCGGAGGCGGAAATTTGCCACGAAAGAGAAAGTATTTCCCCGACAATAATAAAGGGGCG
CTCCTCAGCTTTTCCACTTGGTTGGGTAAGCTAGGCAACTCTGAAAGGAGTTTCGGCGAATTGAAGCCGACAGCTTTGAATTG
TTTTAGGGGCGTTATTCGAGGGCAATCGGAGCTAACTTCAAGACTACTTCTTTGTTGAATACTAAATAGTGCAAAGGTCGTGT
TTCCTCAAGGATACTCCGCTAACAATATAGGATTCCAATCAGATTCAGCACTGGCGGTACGGGTGTTGCGGTGAGGCGTTCGG
GTTTACGGCTCGAAGCTAGCACGGTAGG

p2328

CGCAGGATAAGTAAGGGGAGTAAGTGATCGAACGAATCAGAAGTGACAATATACTTAGGCTGGATCTCGTCCCGTGAATCCCA
ACCCTCACCAACTACGAGATAAGAGGTAAGCCAAAAATCGACTTGGTGGCGACCAACGACTGTTCCCCCCCTGTAACTAATCG
TTCCGTCAAAACCTGACTTACTTCAAGGCCAATTCCAAGCGCAAACAATACCGTCCTAGTTCTTCGGTTAAGTTTCCGAAGTA
GGAGTGAGCCTACCTCCGTTTGCGTCTTGTTACCACTGACCCAGCTATTTACTTTGTATTGCCTGCAATCGAATTTCTGAACT
CTCAGATAGTGGGGATAACGGGAAAGTTCCTATATTTGCGAACTAACTTAGCCGTCCACCTCGAAGCTACCTACTCACACCCA
CCCCGCGCGGGGTAAATAAGGCACTAATCCCAGCTGAGAGCTGGCGTAGCACTTAGCCACAAGTTAATTAACAGTTGTCTGGT
AGTTTGGCGGTATTAGGAAGATCCTAGAAGCAAGGCAGAGTTAGTTCTAACCTAAAGCCACAAATAAGACAGGTTGCCAAAGC
CCGCCGGAAATTAAATCTTGCTCAGTTCGGTAACGGAGTTTCCCTCCCGCGTACTTAATTCCCAATAAGAAACGCGCCCAAGT
CCTATCAGGCAAAATTCAGCCCCTTCCCGTGTTAGAACGAGGGTAAAAATACAAGCCGATTGAACAAGGGTTGGGGGCTTCAA
ATCGTCGTTTACCCCACTTTACAACGGAGATTAAGTAGTTCACCCTATAGTACGAAGCAGAACTATTTCGAGGGGCGTGCAAT
AATCGAATCTTCTGCGGTTGACTTAACACGCTAGGGACGTGCCCTCGATTCAATCGAAGGTACTCCTACTCAGACTGCCTCAC
ACCCAGCTAGTCACTGAGCGATAAAATTGACCCGCCCTCTAGGGAAGCGAGTACGTCCCAAAGGGCTCCGGACAGGGCTATAT
AGGAGAGTTTGATCTCGCCCCGACAACTGCAACCCTCAACTCCCTTAGATAATATTGTTAGCCGAAGTTGCACGACCCGCCGT
CCACGGACTGCTCTTAGGGTGTGGCTCCTTAATCTGACAACGTGCAACCCCTATCGAAGTCGATTGTTTCTGCGAAAGGTGTT
GTCCTAATAGTCCCGAAATTTGGCCCTTGTAGGTGTGAAACCACTTAGCTTCGCGCCGTAGTCCTAAAGGCCCACCTATTGAC
TTTGTTTCGGGTAGCACTAGGAATCTTAACAATTTGAATTTGGACGTGGAACGCGTACACCTTAATCTCCGAATAATTCTAGG
GATTTGGAAGTCCTCTACGTTGACACACCTACACTGCTCGAAGTAAATATACGAATAACGCGGGCCTCGCGGAGCCGTTCCGA
ATCGTCACGTGTTCGTTTACTGTTAATTGGTGGCAAATAAGCAATATCGTAGTCCGTCAGGCCCAGCCCTGTTATCCACGGCG
TTATTTGTCAAATTGCGTAGAACTGGATTGACTGCCTGACAATACCTAATTATCGGTACGAAGTCCCCGAATCTGTCGGGCTA
TTTCACTAATACTTTCCAAACGCCCCGTATCCAAGAAGAACGAATTTATCCACGCTCCCGTCTTTGGGACGAATACCGCTACA
AGTGGACAGAGGATCGGTACGGGCCTCTAATAAATCCAACACTCTACGCCCTCTTCAAGAGCTAGAAGAACAGGGTGCAGTTG
GAAAGGGAATTATTTCGTAAGGCGAGCCAATACCGTAATTAATTCGGAAGAGTTAACACGATTGGAAGTAGGAATAGTTTCTA
ACCACGGTTACTAATCCTAATAACGGAACGCTGTCTGATAGATTAGTGTCAGCGCTCGGTACCAAAGAAAAATAAAAAGACGC
TGAAAAGCGTCTTTTTATTTTTCGGTCCAGTGTAACTCAGGCAAAAGCACGTAATATTCGTACTTTCTTCCTCCGTAAGCGTC
ACCCACATTCCTTAAAGAGTGCATGTGCATATTTTGTTATCAATAAAAAAGGCCGCGATTTGCGGCCTTATTGTTCGTCTTGC
CGGATTACGCCCCGCCCTGCCACTCATCGCAGTATTGTTGTAATTCATTAAGCATTCTGCCGACATGGAAGCCATCACAAACG

GCATGATGAACTTGGATCGCCAGTGGCATTAACACCTTGTCGCCTTGCGTATAATATTTTCCCATAGTGAAAACGGGGGCGAA
GAAGTTGTCCATATTTGCTACGTTTAAATCAAAACTGGTGAAACTCACCCACGGATTGGCACTGACGAAAAACATATTTTCGA
TAAACCCTTTAGGGAAATATGCTAAGTTTTCACCGTAACACGCCACATCTTGACTATATATGTGTAGAAACTGCCGGAAATCG
TCGTGGTATTCTGACCAGAGCGATGAAAACGTTTCAGTTTGCTCATGGAAAACGGTGTAACAAGGGTGAACACTATCCCATAT
CACCAGCTCACCGTCTTTCATTGCCATACGAAACTCCGGATGTGCATTCATCAGGCGGGCAAGAATGTGAATAAAGGCCGGAT
AAAACTTGTGCTTATTTTTCTTTACGGTTTTTAAAAAGGCCGTAATATCCAGCTGAACGGTTTGGTTATAGGTGCACTGAGCA
ACTGACTGGAATGCCTCAAAATGTTCTTTACGATGCCATTGACTTATATCAACTGTAGTATATCCAGTGATTTTTTTCTCCAT
TTTAGCTTCCTTAGCTTGCGAAATCTCGATAACTCAAAAAATAGTAGTGATCTTTCGCAAAAAGTTGGCGCTTTATCAACACT
GTCGGAATGACAAATGGTTCCAATTATTGAACACCCTTCGGGGTGTTTTTTTGTTTCTGGTTTCCCGAGGCCGGCCTTTTGTT
GCAATGGCTGTCTACCCTGTCTACCTGAGTAAAGAAAAATACATTTAATTCAGTATATTAACTTGGGTAGACAGCCTTTTTTT
ACTGTCTACCTTCTGTCTACCCTCTCTACCTGATTTTACCTGAATCAGACAGGGAGGTAGACACGGGGTAGACAGTGGATAAA
AGCACTCTACCCCACTGAAAGCAGTGCCATTACTGGCATGGTTGCCAGTAAGGTTGATAAGGTAGACAAGGGGAGGGACAACT
CAAAACTTTTTAAACGAGGGGGTAAAACGCAGACCAAAACGATCTCAAGAAGATCATCTTATTAATCAGATAAAATATTTCTA
GATTTCAGTGCAATTTATCTCTTCAAATGTAGCACCGGCGCGCCGTGACCAATTATTGAAGGCCGCTAACGCGGCCTTTTTTT
GTTTCTGGTATCCCGAATGGAGCGACTTCTCCCCAAAAAGCCTCGCTTTCAGCACCTGTCGTTTCCTTTCTTTTCAGAGGGTA
TTTTAAATAAAAACATTAAGTTATGACGAAGAAGAACGGAAACGCCTTAAACCGGAAAATTTTCATAAATAGCGAAAACCCGC
GAGGTCGCCGCCCCGTAACCTGTCGGATCACCGGAAAGGACCCGTAAAGTGATAATGATTATCATCTACATATCACAACGTGC
GTAAAGGGTAAGTATGAAGGTCGTGTACTCCATCGCTACCAAATTCCAGAAAACAGACGCTTTCGAGCGTCTTTTTTCGTTTT
GGTCACGACGTACGGTGGAAGATTCGTTACCAATTGACAGCTAGCTCAGTCCTAGGTATATACATACATGCTTGTTTGTTTGT
AAACGGATAGAAATGGGAGATGAAGCGTACCGCCGATGGCAGCGAGTTTGAATCTCCGAAGAAAAAGCGTAAGGTCAGCGAAG
TTGAGTTCAGCCACGAATACTGGATGCGTCACGCTTTAACCCTGGCTAAACGCGCGCGCGACGAGCGCGAAGTACCAGTGGGG
GCGGTGCTGGTGTTAAACAACCGCGTAATCGGCGAAGGCTGGAACCGTGCAATCGGGTTACATGACCCGACCGCCCATGCCGA
GATCATGGCCCTGCGCCAGGGGGGGCTGGTCATGCAGAATTACCGTCTGATCGACGCGACGTTGTATGTCACATTCGAGCCAT
GCGTGATGTGTGCGGGGGCAATGATTCACTCTCGCATTGGTCGCGTCGTGTTTGGCGTTCGTAATAGTAAACGCGGCGCTGCT
GGCTCCTTAATGAATGTTCTGAATTATCCGGGTATGAACCACCGTGTCGAAATTACAGAAGGTATCTTAGCAGATGAATGTGC
CGCACTGCTGTGTGACTTCTACCGCATGCCGCGCCAAGTATTCAACGCCCAAAAAAAAGCTCAGTCCTCAATTAACTCTGGTG
GTAGTAGTGGCGGCTCTAGCGGCTCCGAGACGCCTGGTACGTCGGAATCGGCTACGCCTGAGTCGAGCGGTGGGTCCTCTGGC
GGCTCTGACAAGAAATATAGCATCGGCCTGGCCATCGGCACAAATAGCGTCGGATGGGCGGTGATCACTGATGAATATAAAGT
TCCGTCTAAAAAGTTCAAGGTACTGGGTAATACAGATCGCCATAGTATCAAAAAGAACTTAATCGGTGCGCTTCTGTTCGATT
CCGGCGAAACCGCAGAAGCAACACGTCTGAAACGCACCGCTCGTCGCCGTTACACCCGTCGTAAAAACCGCATCTGCTACCTG
CAAGAAATCTTCTCTAACGAAATGGCTAAAGTAGATGACAGCTTTTTTCACCGTCTGGAAGAATCATTTCTGGTGGAAGAAGA
TAAAAAGCACGAACGTCATCCAATCTTCGGCAACATTGTGGACGAAGTAGCGTATCACGAAAAATACCCGACTATCTATCACC
TGCGCAAAAAGCTGGTCGATTCGACGGATAAGGCCGATCTGCGTCTGATCTATCTGGCCTTAGCGCATATGATTAAGTTCCGT
GGTCATTTCCTGATCGAAGGCGACCTGAATCCAGACAACAGCGATGTAGACAAACTGTTCATCCAGCTGGTGCAAACCTATAA
CCAGCTGTTTGAAGAAAACCCAATTAATGCTAGCGGTGTTGACGCGAAAGCGATCTTGTCCGCACGCCTGTCCAAATCCCGTC
GTCTGGAAAACTTAATTGCGCAACTGCCGGGTGAGAAGAAAAACGGACTGTTCGGCAATCTGATCGCTCTTAGCTTGGGACTG
ACCCCGAACTTCAAAAGCAACTTCGATCTGGCAGAGGACGCAAAACTTCAACTTAGCAAAGATACGTATGACGATGACTTGGA
TAACTTACTGGCCCAGATCGGAGATCAGTACGCTGATCTGTTTCTGGCGGCAAAGAACTTATCAGACGCTATTCTCCTGTCTG
ATATTCTTCGTGTGAATACCGAAATCACCAAAGCACCGCTTTCTGCATCCATGATTAAACGCTATGACGAACATCACCAAGAT
CTGACTCTTCTGAAAGCGCTGGTACGGCAACAACTGCCGGAGAAGTACAAGGAGATCTTCTTTGACCAATCCAAAAACGGCTA
CGCGGGTTATATTGACGGGGGTGCAAGCCAAGAGGAGTTCTACAAATTCATCAAGCCAATCTTAGAAAAAATGGATGGCACGG
AAGAATTACTTGTTAAACTGAATCGTGAGGATCTGCTTCGTAAACAGCGTACCTTCGACAACGGTAGCATTCCGCACCAGATC
CACTTAGGTGAACTGCACGCTATCCTGCGTCGCCAAGAGGATTTTTACCCGTTCCTGAAAGATAATCGTGAAAAAATCGAAAA
AATCCTGACCTTTCGTATCCCGTATTATGTCGGCCCGCTGGCGCGTGGCAACTCCCGTTTCGCGTGGATGACTCGCAAATCCG
AAGAAACTATTACCCCGTGGAACTTCGAGGAAGTGGTTGACAAAGGCGCAAGCGCCCAATCCTTCATCGAGCGCATGACTAAC
TTTGATAAAAACCTGCCGAACGAAAAGGTACTGCCGAAACACTCCCTTCTGTACGAATACTTCACCGTGTACAACGAGCTGAC
TAAAGTAAAGTATGTGACTGAGGGCATGCGTAAACCTGCATTCCTGAGCGGTGAACAGAAAAAAGCAATTGTTGATTTACTGT
TTAAAACCAACCGTAAAGTAACCGTTAAACAGCTGAAAGAGGACTACTTCAAGAAAATCGAATGCTTCGACTCCGTCGAGATT
AGTGGAGTTGAAGATCGTTTTAATGCAAGTTTAGGCACGTATCACGATTTATTAAAGATCATTAAAGACAAAGATTTCTTGGA
CAACGAAGAAAATGAGGACATCTTAGAGGACATCGTCCTGACCCTGACTCTGTTCGAAGATCGTGAAATGATTGAAGAACGCC
TTAAGACGTATGCTCACCTGTTTGACGATAAAGTAATGAAACAACTGAAACGTCGCCGTTATACTGGCTGGGGCCGTCTGAGC
CGTAAACTGATTAACGGTATCCGTGACAAACAGTCCGGTAAAACTATTCTGGACTTCCTGAAATCTGACGGCTTCGCAAACCG
TAACTTCATGCAACTGATTCACGACGATTCCCTGACCTTCAAAGAGGACATCCAGAAAGCTCAGGTTTCTGGTCAAGGTGATT
CTCTGCACGAGCATATCGCCAATTTAGCAGGTAGTCCGGCGATCAAAAAAGGTATCCTGCAAACCGTGAAAGTGGTGGATGAG
CTTGTGAAAGTTATGGGTCGTCACAAACCGGAAAACATTGTTATCGAGATGGCTCGTGAAAACCAAACGACCCAGAAGGGACA
GAAAAACTCCCGCGAACGCATGAAACGTATCGAGGAGGGTATTAAAGAACTTGGCTCTCAGATTCTGAAAGAACACCCTGTTG
AAAATACCCAACTGCAAAATGAAAAACTGTACCTGTACTACCTGCAAAATGGTCGTGACATGTATGTAGATCAGGAGCTGGAC
ATCAACCGCCTCTCCGATTACGACGTTGACCACATTGTTCCGCAGTCTTTTCTGAAAGATGATTCCATTGATAACAAAGTACT
CACCCGTAGCGATAAAAACCGTGGGAAGAGTGACAACGTTCCATCGGAAGAAGTAGTTAAGAAAATGAAGAACTATTGGCGTC
AACTGCTTAACGCGAAACTGATTACTCAACGTAAATTTGATAACCTGACCAAAGCTGAACGTGGCGGTTTGTCTGAGCTGGAT
AAGGCGGGTTTTATTAAACGTCAACTGGTAGAAACTCGCCAGATTACAAAACATGTTGCTCAGATTCTGGACTCTCGTATGAA
CACTAAATACGATGAAAATGACAAACTGATCCGCGAAGTTAAGGTTATTACCCTGAAATCTAAGCTGGTTTCCGACTTCCGTA
AAGATTTCCAATTCTATAAAGTGCGCGAGATTAACAACTATCACCACGCGCACGACGCATATCTGAATGCAGTTGTTGGCACG

GCACTGATCAAAAAATATCCGAAACTGGAAAGCGAATTTGTGTACGGCGATTATAAAGTTTACGACGTGCGCAAAATGATCGC
CAAATCTGAACAGGAAATTGGCAAAGCAACCGCTAAATACTTTTTCTACTCAAACATTATGAATTTCTTCAAAACCGAAATCA
CCTTAGCGAATGGCGAAATTCGTAAACGCCCTCTGATCGAAACCAACGGCGAAACGGGTGAGATCGTGTGGGACAAAGGTCGT
GATTTCGCTACTGTCCGCAAAGTTCTGTCCATGCCTCAAGTAAACATCGTTAAAAAGACTGAGGTACAGACTGGCGGTTTCAG
CAAGGAATCCATTCTGCCGAAACGCAACTCCGACAAACTGATCGCGCGTAAGAAAGACTGGGATCCGAAGAAATACGGTGGCT
TCGATTCTCCAACCGTGGCATACAGCGTTCTGGTAGTCGCCAAAGTCGAAAAGGGTAAATCAAAAAAACTGAAATCAGTGAAA
GAACTTTTAGGCATCACCATTATGGAACGTAGCTCTTTCGAAAAAAACCCGATTGACTTCCTCGAAGCGAAGGGGTACAAGGA
AGTAAAGAAAGATCTGATTATCAAACTGCCGAAGTATTCCCTGTTCGAACTGGAAAATGGTCGTAAACGTATGTTAGCGTCTG
CGGGTGAACTGCAAAAAGGGAACGAATTGGCCCTTCCGTCCAAGTACGTGAACTTCCTGTATCTGGCCTCGCACTACGAGAAA
CTGAAAGGTAGTCCGGAAGATAATGAGCAGAAACAGCTGTTCGTGGAACAGCACAAACACTATCTGGACGAGATTATTGAACA
GATTTCTGAGTTTAGCAAACGCGTAATTCTGGCGGACGCGAATCTGGATAAAGTCCTGAGCGCCTACAATAAACACCGTGATA
AACCGATCCGTGAACAGGCAGAAAACATCATTCACCTGTTCACGCTGACTAATCTTGGTGCTCCGGCAGCCTTCAAATACTTC
GACACCACGATCGATCGTAAACGTTACACCTCCACTAAAGAAGTCTTAGATGCAACTCTTATTCACCAGAGCATCACTGGCCT
GTATGAAACTCGTATTGATCTGAGTCAGTTGGGCGGTGACTAATGTATGCTTAAGCAGCTCGGTACCAAAGACGAACAATAAG
ACGCTGAAAAGCGTCTTTTTTCGTTTTGGTCCTGTTGCGGCGCGATAGTGTGAACATGCTATAGACTTCTGGTGCTACCCGAC
TGACAATTAATCATCCGGCTCGTATAATGCTAGCACTTTTAAAGTTCTGCTATGGTTTTAGAGCTAGAAATAGCAAGTTAAAA
TAAGGCTAGTCCGTTATCAACTTGAAAAAGTGGCACCGAGTCGGTGCTTTTTTTAATCCATTCGAATCACGTCCGACGAACAA
TAAGGCCTCCCTAACGGGGGGCCTTTTTTATTGATAACAAAAGTAACTTCGAGCTTGTCTACCTCCTAGCACCATTATTGCAA
TTAATAAACAACTAACGGACAATTCTACCTAACAATGAGTAAAGGTGAGGAATTATTTACTGGTGTTGTTCCGATCTTAGTTG
AACTGGACGGCGATGTTAACGGTCATAAATTCAGTGTTCGTGGTGAAGGTGAAGGTGATGCAACCAACGGTAAGCTGACCCTG
AAATTCATCTGCACTACTGGAAAATTACCAGTACCGTGGCCTACTCTGGTGACTACCCTGACCTATGGTGTTCAGTGTTTTTC
TCGTTACCCTGACCACATGAAGCAACATGATTTCTTCAAATCTGCAATGCCGGAAGGTTATGTACAGGAGCGCACCATTTCTT
TCAAAGACGATGGCACGTATAAAACCCGTGCAGAGGTTAAATTTGAAGGTGACACTCTGGTGAATCGTATTGAACTGAAAGGC
ATTGATTTCAAAGAGGACGGCAATATTTTAGGCCACAAACTGGAATATAACTTCAACTCCCATAACGTTTACATCACCGCAGA
CAAACAGAAGAACGGTATCAAAGCTAACTTCAAAATTCGCCATAACGTTGAAGATGGTAGCGTACAGCTGGCGGATCATTACC
AACAGAACACTCCGATTGGAGATGCTCCTGTTTTACTGCCGGATAACCACTACCTGTCCACCCAGTCTAAACTGTCGAAGGAT
CCGAACGAAAAGCGCGACCACATGGTGTTATTAGAGTTCGTTACCGCTAGTGGTATCACGCACGGTATGGATGAACTCTACAA
ATAAGACGAACAATAAGGGGAGCGGGAAACCGCTCCCCTTTTTTATTGATAACAAAAGTAAATTGCACGCTGATAGTCTCCCA
ATTGCGAAGGACCAAAACGAAAAAACACCCTTTCGGGTGTCTTTTCTGGAATTTGGTACCGAGTACTAGGTATCGTGTAAGTA
GCGAAGGCCCGTACGCGAGATAAACTGCTAGGCAACCGCGACTCTACGACTGGTGCTCGATTTAATTTCGCTGACGTAAAGAA
ATTATCGGCAGTGCGTCAACTGCCGTATCTTTATCTTAATTAGGTAGTTGGACAAGCCCTTGAAAGAAATAGCAAGAGCCTGC
CTCTCTATTGAAGTCACGGCGAAAGTCGGGTAGAAATCAAAGAAAGCAGAAATTAAATCGGAGTAACACTAAGGTGGGATAAC
TCCGTAACTGACTACGCCTTTCTCTAGACTTTACTTGACCAGATACACTGTCTTTGACACGTTGAAGGATTAGAGCAATCAAA
TCCAAGACTGGCTAAGCACGAAGCAACTCTTGAGTGTTAAAAAGTTATCTCCTGTATTCGGGAAGCGGGTACTAGAAGATTGC
AGGGACTCCGACGTTAAGTAAATTACAAAGTAATAAGTATCGTTCAGGATCACGTTACCGCAATAAGAAGCGAGAATAATATA
ATTTCCGAAGTGCTTACCCCAGTAGTGACTATTCCTATAACCCTTCTGAGTGTCCGGAGGCGGAAATTTGCCACGAAAGAGAA
AGTATTTCCCCGACAATAATAAAGGGGCGCTCCTCAGCTTTTCCACTTGGTTGGGTAAGCTAGGCAACTCTGAAAGGAGTTTC
GGCGAATTGAAGCCGACAGCTTTGAATTGTTTTAGGGGCGTTATTCGAGGGCAATCGGAGCTAACTTCAAGACTACTTCTTTG
TTGAATACTAAATAGTGCAAAGGTCGTGTTTCCTCAAGGATACTCCGCTAACAATATAGGATTCCAATCAGATTCAGCACTGG
CGGTACGGGTGTTGCGGTGAGGCGTTCGGGTTTACGGCTCGAAGCTAGCACGGTAGG

p2515

CGCAGGATAAGTAAGGGGAGTAAGTGATCGAACGAATCAGAAGTGACAATATACTTAGGCTGGATCTCGTCCCGTGAATCCCA
ACCCTCACCAACTACGAGATAAGAGGTAAGCCAAAAATCGACTTGGTGGCGACCAACGACTGTTCCCCCCCTGTAACTAATCG
TTCCGTCAAAACCTGACTTACTTCAAGGCCAATTCCAAGCGCAAACAATACCGTCCTAGTTCTTCGGTTAAGTTTCCGAAGTA
GGAGTGAGCCTACCTCCGTTTGCGTCTTGTTACCACTGACCCAGCTATTTACTTTGTATTGCCTGCAATCGAATTTCTGAACT
CTCAGATAGTGGGGATAACGGGAAAGTTCCTATATTTGCGAACTAACTTAGCCGTCCACCTCGAAGCTACCTACTCACACCCA
CCCCGCGCGGGGTAAATAAGGCACTAATCCCAGCTGAGAGCTGGCGTAGCACTTAGCCACAAGTTAATTAACAGTTGTCTGGT
AGTTTGGCGGTATTAGGAAGATCCTAGAAGCAAGGCAGAGTTAGTTCTAACCTAAAGCCACAAATAAGACAGGTTGCCAAAGC
CCGCCGGAAATTAAATCTTGCTCAGTTCGGTAACGGAGTTTCCCTCCCGCGTACTTAATTCCCAATAAGAAACGCGCCCAAGT
CCTATCAGGCAAAATTCAGCCCCTTCCCGTGTTAGAACGAGGGTAAAAATACAAGCCGATTGAACAAGGGTTGGGGGCTTCAA
ATCGTCGTTTACCCCACTTTACAACGGAGATTAAGTAGTTCACCCTATAGTACGAAGCAGAACTATTTCGAGGGGCGTGCAAT
AATCGAATCTTCTGCGGTTGACTTAACACGCTAGGGACGTGCCCTCGATTCAATCGAAGGTACTCCTACTCAGACTGCCTCAC
ACCCAGCTAGTCACTGAGCGATAAAATTGACCCGCCCTCTAGGGAAGCGAGTACGTCCCAAAGGGCTCCGGACAGGGCTATAT
AGGAGAGTTTGATCTCGCCCCGACAACTGCAACCCTCAACTCCCTTAGATAATATTGTTAGCCGAAGTTGCACGACCCGCCGT
CCACGGACTGCTCTTAGGGTGTGGCTCCTTAATCTGACAACGTGCAACCCCTATCGAAGTCGATTGTTTCTGCGAAAGGTGTT
GTCCTAATAGTCCCGAAATTTGGCCCTTGTAGGTGTGAAACCACTTAGCTTCGCGCCGTAGTCCTAAAGGCCCACCTATTGAC
TTTGTTTCGGGTAGCACTAGGAATCTTAACAATTTGAATTTGGACGTGGAACGCGTACACCTTAATCTCCGAATAATTCTAGG
GATTTGGAAGTCCTCTACGTTGACACACCTACACTGCTCGAAGTAAATATACGAATAACGCGGGCCTCGCGGAGCCGTTCCGA
ATCGTCACGTGTTCGTTTACTGTTAATTGGTGGCAAATAAGCAATATCGTAGTCCGTCAGGCCCAGCCCTGTTATCCACGGCG
TTATTTGTCAAATTGCGTAGAACTGGATTGACTGCCTGACAATACCTAATTATCGGTACGAAGTCCCCGAATCTGTCGGGCTA

TTTCACTAATACTTTCCAAACGCCCCGTATCCAAGAAGAACGAATTTATCCACGCTCCCGTCTTTGGGACGAATACCGCTACA
AGTGGACAGAGGATCGGTACGGGCCTCTAATAAATCCAACACTCTACGCCCTCTTCAAGAGCTAGAAGAACAGGGTGCAGTTG
GAAAGGGAATTATTTCGTAAGGCGAGCCAATACCGTAATTAATTCGGAAGAGTTAACACGATTGGAAGTAGGAATAGTTTCTA
ACCACGGTTACTAATCCTAATAACGGAACGCTGTCTGATAGATTAGTGTCAGCGCTCGGTACCAAAGAAAAATAAAAAGACGC
TGAAAAGCGTCTTTTTATTTTTCGGTCCAGTGTAACTCAGGCAAAAGCACGTAATATTCGTACTTTCTTCCTCCGTAAGCGTC
ACCCACATTCCTTAAAGAGTGCATGTGCATATTTTGTTATCAATAAAAAAGGCCGCGATTTGCGGCCTTATTGTTCGTCTTGC
CGGATTACGCCCCGCCCTGCCACTCATCGCAGTATTGTTGTAATTCATTAAGCATTCTGCCGACATGGAAGCCATCACAAACG
GCATGATGAACTTGGATCGCCAGTGGCATTAACACCTTGTCGCCTTGCGTATAATATTTTCCCATAGTGAAAACGGGGGCGAA
GAAGTTGTCCATATTTGCTACGTTTAAATCAAAACTGGTGAAACTCACCCACGGATTGGCACTGACGAAAAACATATTTTCGA
TAAACCCTTTAGGGAAATATGCTAAGTTTTCACCGTAACACGCCACATCTTGACTATATATGTGTAGAAACTGCCGGAAATCG
TCGTGGTATTCTGACCAGAGCGATGAAAACGTTTCAGTTTGCTCATGGAAAACGGTGTAACAAGGGTGAACACTATCCCATAT
CACCAGCTCACCGTCTTTCATTGCCATACGAAACTCCGGATGTGCATTCATCAGGCGGGCAAGAATGTGAATAAAGGCCGGAT
AAAACTTGTGCTTATTTTTCTTTACGGTTTTTAAAAAGGCCGTAATATCCAGCTGAACGGTTTGGTTATAGGTGCACTGAGCA
ACTGACTGGAATGCCTCAAAATGTTCTTTACGATGCCATTGACTTATATCAACTGTAGTATATCCAGTGATTTTTTTCTCCAT
TTTAGCTTCCTTAGCTTGCGAAATCTCGATAACTCAAAAAATAGTAGTGATCTTATTTCATTATGGTGAAAGTTGTCTTACGT
GCAACATTTTCGCAAAAAGTTGGCGCTTTATCAACACTGTCGGAATGACAAATGGTTCCAATTATTGAACACCCTTCGGGGTG
TTTTTTTGTTTCTGGTTTCCCGAGGCCGGCCTTTTGTTGCAATGGCTGTCTACCCTGTCTACCTGAGTAAAGAAAAATACATT
TAATTCAGTATATTAACTTGGGTAGACAGCCTTTTTTTACTGTCTACCTTCTGTCTACCCTCTCTACCTGATTTTACCTGAAT
CAGACAGGGAGGTAGACACGGGGTAGACAGTGGATAAAAGCACTCTACCCCACTGAAAGCAGTGCCATTACTGGCATGGTTGC
CAGTAAGGTTGATAAGGTAGACAAGGGGAGGGACAACTCAAAACTTTTTAAACGAGGGGGTAAAACGCAGACCAAAACGATCT
CAAGAAGATCATCTTATTAATCAGATAAAATATTTCTAGATTTCAGTGCAATTTATCTCTTCAAATGTAGCACCGGCGCGCCG
TGACCAATTATTGAAGGCCGCTAACGCGGCCTTTTTTTGTTTCTGGTATCCCGAATGGAGCGACTTCTCCCCAAAAAGCCTCG
CTTTCAGCACCTGTCGTTTCCTTTCTTTTCAGAGGGTATTTTAAATAAAAACATTAAGTTATGACGAAGAAGAACGGAAACGC
CTTAAACCGGAAAATTTTCATAAATAGCGAAAACCCGCGAGGTCGCCGCCCCGTAACCTGTCGGATCACCGGAAAGGACCCGT
AAAGTGATAATGATTATCATCTACATATCACAACGTGCGTAAAGGGTAAGTATGAAGGTCGTGTACTCCATCGCTACCAAATT
CCAGAAAACAGACGCTTTCGAGCGTCTTTTTTCGTTTTGGTCACGACGTACGGTGGAAGATTCGTTACCAATTGACAGCTAGC
TCAGTCCTAGGTATATACATACATGCTTGTTTGTTTGTAAACGGAGATAAATGGTGAATGCGAGATGAAGCGTACCGCCGATG
GCAGCGAGTTTGAATCTCCGAAGAAAAAGCGTAAGGTCAGCGAAGTTGAGTTCAGCCACGAATACTGGATGCGTCACGCTTTA
ACCCTGGCTAAACGCGCGCGCGACGAGCGCGAAGTACCAGTGGGGGCGGTGCTGGTGTTAAACAACCGCGTAATCGGCGAAGG
CTGGAACCGTGCAATCGGGTTACATGACCCGACCGCCCATGCCGAGATCATGGCCCTGCGCCAGGGGGGGCTGGTCATGCAGA
ATTACCGTCTGATCGACGCGACGTTGTATGTCACATTCGAGCCATGCGTGATGTGTGCGGGGGCAATGATTCACTCTCGCATT
GGTCGCGTCGTGTTTGGCGTTCGTAATAGTAAACGCGGCGCTGCTGGCTCCTTAATGAATGTTCTGAATTATCCGGGTATGAA
CCACCGTGTCGAAATTACAGAAGGTATCTTAGCAGATGAATGTGCCGCACTGCTGTGTGACTTCTACCGCATGCCGCGCCAAG
TATTCAACGCCCAAAAAAAAGCTCAGTCCTCAATTAACTCTGGTGGTAGTAGTGGCGGCTCTAGCGGCTCCGAGACGCCTGGT
ACGTCGGAATCGGCTACGCCTGAGTCGAGCGGTGGGTCCTCTGGCGGCTCTGACAAGAAATATAGCATCGGCCTGGCCATCGG
CACAAATAGCGTCGGATGGGCGGTGATCACTGATGAATATAAAGTTCCGTCTAAAAAGTTCAAGGTACTGGGTAATACAGATC
GCCATAGTATCAAAAAGAACTTAATCGGTGCGCTTCTGTTCGATTCCGGCGAAACCGCAGAAGCAACACGTCTGAAACGCACC
GCTCGTCGCCGTTACACCCGTCGTAAAAACCGCATCTGCTACCTGCAAGAAATCTTCTCTAACGAAATGGCTAAAGTAGATGA
CAGCTTTTTTCACCGTCTGGAAGAATCATTTCTGGTGGAAGAAGATAAAAAGCACGAACGTCATCCAATCTTCGGCAACATTG
TGGACGAAGTAGCGTATCACGAAAAATACCCGACTATCTATCACCTGCGCAAAAAGCTGGTCGATTCGACGGATAAGGCCGAT
CTGCGTCTGATCTATCTGGCCTTAGCGCATATGATTAAGTTCCGTGGTCATTTCCTGATCGAAGGCGACCTGAATCCAGACAA
CAGCGATGTAGACAAACTGTTCATCCAGCTGGTGCAAACCTATAACCAGCTGTTTGAAGAAAACCCAATTAATGCTAGCGGTG
TTGACGCGAAAGCGATCTTGTCCGCACGCCTGTCCAAATCCCGTCGTCTGGAAAACTTAATTGCGCAACTGCCGGGTGAGAAG
AAAAACGGACTGTTCGGCAATCTGATCGCTCTTAGCTTGGGACTGACCCCGAACTTCAAAAGCAACTTCGATCTGGCAGAGGA
CGCAAAACTTCAACTTAGCAAAGATACGTATGACGATGACTTGGATAACTTACTGGCCCAGATCGGAGATCAGTACGCTGATC
TGTTTCTGGCGGCAAAGAACTTATCAGACGCTATTCTCCTGTCTGATATTCTTCGTGTGAATACCGAAATCACCAAAGCACCG
CTTTCTGCATCCATGATTAAACGCTATGACGAACATCACCAAGATCTGACTCTTCTGAAAGCGCTGGTACGGCAACAACTGCC
GGAGAAGTACAAGGAGATCTTCTTTGACCAATCCAAAAACGGCTACGCGGGTTATATTGACGGGGGTGCAAGCCAAGAGGAGT
TCTACAAATTCATCAAGCCAATCTTAGAAAAAATGGATGGCACGGAAGAATTACTTGTTAAACTGAATCGTGAGGATCTGCTT
CGTAAACAGCGTACCTTCGACAACGGTAGCATTCCGCACCAGATCCACTTAGGTGAACTGCACGCTATCCTGCGTCGCCAAGA
GGATTTTTACCCGTTCCTGAAAGATAATCGTGAAAAAATCGAAAAAATCCTGACCTTTCGTATCCCGTATTATGTCGGCCCGC
TGGCGCGTGGCAACTCCCGTTTCGCGTGGATGACTCGCAAATCCGAAGAAACTATTACCCCGTGGAACTTCGAGGAAGTGGTT
GACAAAGGCGCAAGCGCCCAATCCTTCATCGAGCGCATGACTAACTTTGATAAAAACCTGCCGAACGAAAAGGTACTGCCGAA
ACACTCCCTTCTGTACGAATACTTCACCGTGTACAACGAGCTGACTAAAGTAAAGTATGTGACTGAGGGCATGCGTAAACCTG
CATTCCTGAGCGGTGAACAGAAAAAAGCAATTGTTGATTTACTGTTTAAAACCAACCGTAAAGTAACCGTTAAACAGCTGAAA
GAGGACTACTTCAAGAAAATCGAATGCTTCGACTCCGTCGAGATTAGTGGAGTTGAAGATCGTTTTAATGCAAGTTTAGGCAC
GTATCACGATTTATTAAAGATCATTAAAGACAAAGATTTCTTGGACAACGAAGAAAATGAGGACATCTTAGAGGACATCGTCC
TGACCCTGACTCTGTTCGAAGATCGTGAAATGATTGAAGAACGCCTTAAGACGTATGCTCACCTGTTTGACGATAAAGTAATG
AAACAGCTGAAACGTCGCCGTTATACTGGCTGGGGCCGTCTGAGCCGTAAACTGATTAACGGTATCCGTGACAAACAGTCCGG
TAAAACTATTCTGGACTTCCTGAAATCTGACGGCTTCGCAAACCGTAACTTCATGCAACTGATTCACGACGATTCCCTGACCT
TCAAAGAGGACATCCAGAAAGCTCAGGTTTCTGGTCAAGGTGATTCTCTGCACGAGCATATCGCCAATTTAGCAGGTAGTCCG
GCGATCAAAAAAGGTATCCTGCAAACCGTGAAAGTGGTGGATGAGCTTGTGAAAGTTATGGGTCGTCACAAACCGGAAAACAT
TGTTATCGAGATGGCTCGTGAAAACCAAACGACCCAGAAGGGACAGAAAAACTCCCGCGAACGCATGAAACGTATCGAGGAGG

GTATTAAAGAACTTGGCTCTCAGATTCTGAAAGAACACCCTGTTGAAAATACCCAACTGCAAAATGAAAAACTGTACCTGTAC
TACCTGCAAAATGGTCGTGACATGTATGTAGATCAGGAGCTGGACATCAACCGCCTCTCCGATTACGACGTTGACCACATTGT
TCCGCAGTCTTTTCTGAAAGATGATTCCATTGATAACAAAGTACTCACCCGTAGCGATAAAAACCGTGGGAAGAGTGACAACG
TTCCATCGGAAGAAGTAGTTAAGAAAATGAAGAACTATTGGCGTCAACTGCTTAACGCGAAACTGATTACTCAACGTAAATTT
GATAACCTGACCAAAGCTGAACGTGGCGGTTTGTCTGAGCTGGATAAGGCGGGTTTTATTAAACGTCAACTGGTAGAAACTCG
CCAGATTACAAAACATGTTGCTCAGATTCTGGACTCTCGTATGAACACTAAATACGATGAAAATGACAAACTGATCCGCGAAG
TTAAGGTTATTACCCTGAAATCTAAGCTGGTTTCCGACTTCCGTAAAGATTTCCAATTCTATAAAGTGCGCGAGATTAACAAC
TATCACCACGCGCACGACGCATATCTGAATGCAGTTGTTGGCACGGCACTGATCAAAAAATATCCGAAACTGGAAAGCGAATT
TGTGTACGGCGATTATAAAGTTTACGACGTGCGCAAAATGATCGCCAAATCTGAACAGGAAATTGGCAAAGCAACCGCTAAAT
ACTTTTTCTACTCAAACATTATGAATTTCTTCAAAACCGAAATCACCTTAGCGAATGGCGAAATTCGTAAACGCCCTCTGATC
GAAACCAACGGCGAAACGGGTGAGATCGTGTGGGACAAAGGTCGTGATTTCGCTACTGTCCGCAAAGTTCTGTCCATGCCTCA
AGTAAACATCGTTAAAAAGACTGAGGTACAGACTGGCGGTTTCAGCAAGGAATCCATTCTGCCGAAACGCAACTCCGACAAAC
TGATCGCGCGTAAGAAAGACTGGGATCCGAAGAAATACGGTGGCTTCGATTCTCCAACCGTGGCATACAGCGTTCTGGTAGTC
GCCAAAGTCGAAAAGGGTAAATCAAAAAAACTGAAATCAGTGAAAGAACTTTTAGGCATCACCATTATGGAACGTAGCTCTTT
CGAAAAAAACCCGATTGACTTCCTCGAAGCGAAGGGGTACAAGGAAGTAAAGAAAGATCTGATTATCAAACTGCCGAAGTATT
CCCTGTTCGAACTGGAAAATGGTCGTAAACGTATGTTAGCGTCTGCGGGTGAACTGCAAAAAGGGAACGAATTGGCCCTTCCG
TCCAAGTACGTGAACTTCCTGTATCTGGCCTCGCACTACGAGAAACTGAAAGGTAGTCCGGAAGATAATGAGCAGAAACAGCT
GTTCGTGGAACAGCACAAACACTATCTGGACGAGATTATTGAACAGATTTCTGAGTTTAGCAAACGCGTAATTCTGGCGGACG
CGAATCTGGATAAAGTCCTGAGCGCCTACAATAAACACCGTGATAAACCGATCCGTGAACAGGCAGAAAACATCATTCACCTG
TTCACGCTGACTAATCTTGGTGCTCCGGCAGCCTTCAAATACTTCGACACCACGATCGATCGTAAACGTTACACCTCCACTAA
AGAAGTCTTAGATGCAACTCTTATTCACCAGAGCATCACTGGCCTGTATGAAACTCGTATTGATCTGAGTCAGTTGGGCGGTG
ACTAATGTATGCTTAAGCAGCTCGGTACCAAAGACGAACAATAAGACGCTGAAAAGCGTCTTTTTTCGTTTTGGTCCTGTTGC
GGCGCGATAGTGTGAACATGCTATAGACTTCTGGTGCTACCCGACTGACAATTAATCATCCGGCTCGTATAATGCTAGCGTTT
CATGTAAAACCCCCATGTTTTAGAGCTAGAAATAGCAAGTTAAAATAAGGCTAGTCCGTTATCAACTTGAAAAAGTGGCACCG
AGTCGGTGCTTTTTTTAATCCATTCGAATCACGTCCGACGAACAATAAGGCCTCCCTAACGGGGGGCCTTTTTTATTGATAAC
AAAAGTAACTTCGAGCTTGTCTACCTCCTAGCACCATTATTGCAATTAATAAACAACTAACGGACAATTCTACCTAACAGTTT
TCATATATGACGAGCAGTTAAGTGATGAGTAAAGGTGAGGAATTATTTACTGGTGTTGTTCCGATCTTAGTTGAACTGGACGG
CGATGTTAACGGTCATAAATTCAGTGTTCGTGGTGAAGGTGAAGGTGATGCAACCAACGGTAAGCTGACCCTGAAATTCATCT
GCACTACTGGAAAATTACCAGTACCGTGGCCTACTCTGGTGACTACCCTGACCTATGGTGTTCAGTGTTTTTCTCGTTACCCT
GACCACATGAAGCAACATGATTTCTTCAAATCTGCAATGCCGGAAGGTTATGTACAGGAGCGCACCATTTCTTTCAAAGACGA
TGGCACGTATAAAACCCGTGCAGAGGTTAAATTTGAAGGTGACACTCTGGTGAATCGTATTGAACTGAAAGGCATTGATTTCA
AAGAGGACGGCAATATTTTAGGCCACAAACTGGAATATAACTTCAACTCCCATAACGTTTACATCACCGCAGACAAACAGAAG
AACGGTATCAAAGCTAACTTCAAAATTCGCCATAACGTTGAAGATGGTAGCGTACAGCTGGCGGATCATTACCAACAGAACAC
TCCGATTGGAGATGCTCCTGTTTTACTGCCGGATAACCACTACCTGTCCACCCAGTCTAAACTGTCGAAGGATCCGAACGAAA
AGCGCGACCACATGGTGTTATTAGAGTTCGTTACCGCTAGTGGTATCACGCACGGTATGGATGAACTCTACAAATAAGACGAA
CAATAAGGGGAGCGGGAAACCGCTCCCCTTTTTTATTGATAACAAAAGTAAATTGCACGCTGATAGTCTCCCAATTGCGAAGG
ACCAAAACGAAAAAACACCCTTTCGGGTGTCTTTTCTGGAATTTGGTACCGAGTACTAGGTATCGTGTAAGTAGCGAAGGCCC
GTACGCGAGATAAACTGCTAGGCAACCGCGACTCTACGACTGGTGCTCGATTTAATTTCGCTGACGTAAAGAAATTATCGGCA
GTGCGTCAACTGCCGTATCTTTATCTTAATTAGGTAGTTGGACAAGCCCTTGAAAGAAATAGCAAGAGCCTGCCTCTCTATTG
AAGTCACGGCGAAAGTCGGGTAGAAATCAAAGAAAGCAGAAATTAAATCGGAGTAACACTAAGGTGGGATAACTCCGTAACTG
ACTACGCCTTTCTCTAGACTTTACTTGACCAGATACACTGTCTTTGACACGTTGAAGGATTAGAGCAATCAAATCCAAGACTG
GCTAAGCACGAAGCAACTCTTGAGTGTTAAAAAGTTATCTCCTGTATTCGGGAAGCGGGTACTAGAAGATTGCAGGGACTCCG
ACGTTAAGTAAATTACAAAGTAATAAGTATCGTTCAGGATCACGTTACCGCAATAAGAAGCGAGAATAATATAATTTCCGAAG
TGCTTACCCCAGTAGTGACTATTCCTATAACCCTTCTGAGTGTCCGGAGGCGGAAATTTGCCACGAAAGAGAAAGTATTTCCC
CGACAATAATAAAGGGGCGCTCCTCAGCTTTTCCACTTGGTTGGGTAAGCTAGGCAACTCTGAAAGGAGTTTCGGCGAATTGA
AGCCGACAGCTTTGAATTGTTTTAGGGGCGTTATTCGAGGGCAATCGGAGCTAACTTCAAGACTACTTCTTTGTTGAATACTA
AATAGTGCAAAGGTCGTGTTTCCTCAAGGATACTCCGCTAACAATATAGGATTCCAATCAGATTCAGCACTGGCGGTACGGGT
GTTGCGGTGAGGCGTTCGGGTTTACGGCTCGAAGCTAGCACGGTAGG

p2564

TCAGATCCTTCCGTATTTAGCCAGTATGTTCTCTAGTGTGGTTCGTTGTTTTTGCGTGAGCCATGAGAACGAACCATTGAGAT
CATACTTACTTTGCATGTCACTCAAAAATTTTGCCTCAAAACTGGTGAGCTGAATTTTTGCAGTTAAAGCATCGTGTAGTGTT
TTTCTTAGTCCGTTACGTAGGTAGGAATCTGATGTAATGGTTGTTGGTATTTTGTCACCATTCATTTTTATCTGGTTGTTCTC
AAGTTCGGTTACGAGATCCATTTGTCTATCTAGTTCAACTTGGAAAATCAACGTATCAGTCGGGCGGCCTCGCTTATCAACCA
CCAATTTCATATTGCTGTAAGTGTTTAAATCTTTACTTATTGGTTTCAAAACCCATTGGTTAAGCCTTTTAAACTCATGGTAG
TTATTTTCAAGCATTAACATGAACTTAAATTCATCAAGGCTAATCTCTATATTTGCCTTGTGAGTTTTCTTTTGTGTTAGTTC
TTTTAATAACCACTCATAAATCCTCATAGAGTATTTGTTTTCAAAAGACTTAACATGTTCCAGATTATATTTTATGAATTTTT
TTAACTGGAAAAGATAAGGCAATATCTCTTCACTAAAAACTAATTCTAATTTTTCGCTTGAGAACTTGGCATAGTTTGTCCAC
TGGAAAATCTCAAAGCCTTTAACCAAAGGATTCCTGATTTCCACAGTTCTCGTCATCAGCTCTCTGGTTGCTTTAGCTAATAC
ACCATAAGCATTTTCCCTACTGATGTTCATCATCTGAGCGTATTGGTTATAAGTGAACGATACCGTCCGTTCTTTCCTTGTAG
GGTTTTCAATCGTGGGGTTGAGTAGTGCCACACAGCATAAAATTAGCTTGGTTTCATGCTCCGTTAAGTCATAGCGACTAATC

GCTAGTTCATTTGCTTTGAAAACAACTAATTCAGACATACATCTCAATTGGTCTAGGTGATTTTAATCACTATACCAATTGAG
ATGGGCTAGTCAATGATAATTACTAGTCCTTTTCCTTTGAGTTGTGGGTATCTGTAAATTCTGCTAGACCTTTGCTGGAAAAC
TTGTAAATTCTGCTAGACCCTCTGTAAATTCCGCTAGACCTTTGTGTGTTTTTTTTGTTTATATTCAAGTGGTTATAATTTAT
AGAATAAAGAAAGAATAAAAAAAGATAAAAAGAATAGATCCCAGCCCTGTGTATAACTCACTACTTTAGTCAGTTCCGCAGTA
TTACAAAAGGATGTCGCAAACGCTGTTTGCTCCTCTACAAAACAGACCTTAAAACCCTAAAGGCTTAAGTAGCACCCTCGCAA
GCTCGGTTGCGGCCGCAATCGGGCAAATCGCTGAATATTCCTTTTGTCTCCGACCATCAGGCACCTGAGTCGCTGTCTTTTTC
GTGACATTCAGTTCGCTGCGCTCACGGCTCTGGCAGTGAATGGGGGTAAATGGCACTACAGGCGCCTTTTATGGATTCATGCA
AGGAAACTACCCATAATACAAGAAAAGCCCGTCACGGGCTTCTCAGGGCGTTTTATGGCGGGTCTGCTATGTGGTGCTATCTG
ACTTTTTGCTGTTCAGCAGTTCCTGCCCTCTGATTTTCCAGTCTGACCACTTCGGATTATCCCGTGACAGGTCATTCAGACTG
GCTAATGCACCCAGTAAGGCAGCGGTATCATCAACGGGGTCTGACGCTCAGTGGAACGAAAACTCACGTTAAGGGATTTTGGT
CATGAGATTATCAAAAAGGATCTTCACCTAGATCCTTTTAAATTAAAAATGAAGTTTTAAATCAATCTAAAGTATATATGAGT
AAACTTGGTCTGACAGTTACGTTTCCACAACCAATTAACCAATTCTGATTTAGAAAAACTCATCGAGCATCAAATGAAACTGC
AATTTATTCATATCAGGATTATCAATACCATATTTTTGAAAAAGCCGTTTCTGTAATGAAGGAGAAAACTCACCGAGGCAGTT
CCATAGGATGGCAAGATCCTGGTATCGGTCTGCGATTCCGACTCGTCCAACATCAATACAACCTATTAATTTCCCCTCGTCAA
AAATAAGGTTATCAAGTGAGAAATCACCATGAGTGACGACTGAATCCGGTGAGAATGGCAAAAGCTTATGCATTTCTTTCCAG
ACTTGTTCAACAGGCCAGCCATTACGCTCGTCATCAAAATCACTCGCATCAACCAAACCGTTATTCATTCGTGATTGCGCCTG
AGCGAGACGAAATACGCGATCGCTGTTAAAAGGACAATTACAAACAGGAATCGAATGCAACCGGCGCAGGAACACTGCCAGCG
CATCAACAATATTTTCACCTGAATCAGGATATTCTTCTAATACCTGGAATGCTGTTTTCCCGGGGATCGCAGTGGTGAGTAAC
CATGCATCATCAGGAGTACGGATAAAATGCTTGATGGTCGGAAGAGGCATAAATTCCGTCAGCCAGTTTAGTCTGACCATCTC
ATCTGTAACATCATTGGCAACGCTACCTTTGCCATGTTTCAGAAACAACTCTGGCGCATCGGGCTTCCCATACAATCGATAGA
TTGTCGCACCTGATTGCCCGACATTATCGCGAGCCCATTTATACCCATATAAATCAGCATCCATGTTGGAATTTAATCGCGGC
CTCGAGCAAGACGTTTCCCGTTGAATATGGCTCATAACACCCCTTGTATTACTGTTTATGTAAGCAGACAGTTTTATTGTTCA
TGATGATATATTTTTATCTTGTGCAATGTAACATCAGAGATTTTGAGACACAACGTGGCTTTCCCTGCAGGATTTCGGAGGCC
TGCGTTATCCCCTGATTCTGTGGATAACCGTATTACCGCCTTTGAGTGAGCTGATACCGCTCGCCGCAGCCGAACGCCGACTA
GTGGATTTTACGGCTAGCTCAGTCCTAGGTACAATGCTAGCGAATTCATTAAAGAGGAGAAAGGTACCATGAAACTGGCACCG
AACGTAAAACAGCAGTCACGCGGCATAAAACACAAAGAAACAGAAGTCATTATTTTTGCGGGTAGTGATGCCTGGTCACACGC
AAAACAATGGCAGGAACATGACGCGCGTATGGCCGGAGATAATGAGCCTCCTGTGTGGCTTGGGGAGCAGCAGTTATCCGAAC
TGGATAAGCTGCAAATTGTGCCGGAAGGCAGAAAATCCGTGCGCATATTCAGGGCCGGATATCTTGCGCCAGTAATGATAAAG
GCGATTGGTCAGAAGCTGGCGGCGGCAGGCGTACAGGATGCAAATTTTTACCCTGATGGTATGCACGGTCAGAAGGTGGAGAA
CTGGCGCGAATATCTGGCCCGTGAGCGCCAGAATCTTTCTGATGGTCTGGTCATTGAGCTTCCGGTAAAGCAAAAGGCGCAAC
TTTCGCAGATGGCGGACAGTGAGCGCGCGCAGCTGCTTGCCGATCGCTTTGATGGCGTTTGCGTACATCCTGAAAGTGAAATC
GTTCACGTATGGCGCGGCGGGGTATGGTGTCCGGTCAGCACAATGGAGCTGAGCCGCGAAATGGTGGCGATCTATTCAGAGCA
CAGGGCCACTTTCAGCAAGCGCGTAATCAATAACGCCGTGGAAGCGTTAAAAGTTATTGCCGAACCAATGGGCGAGCCGTCCG
GCGATTTGCTGCCGTTCGCCAATGGTGCGCTTGACCTGAAAACGGGGGAATTTTCCCCGCACACGCCGGAGAACTGGATCACC
ACGCACAACGGCATTGAGTACACGCCACCAGCACCCGGGGAGAACATCCGCGATAACGCGCCAAACTTTCATAAATGGCTTGA
GCACGCAGCCGGAAAAGACCCGCGCAAGATGATGCGTATATGTGCCGCGCTGTACATGATTATGGCGAACCGGTACGACTGGC
AGATGTTTATTGAGGCCACCGGAGACGGCGGGAGCGGTAAAAGTACATTCACACACATAGCCAGCCTTCTGGCAGGGAAACAA
AACACGGTAAGCGCTGAAATGACATCGCTTGATGATGCTGGTGGGCGTGCGCAGGTTGTCGGGAGTCGTCTTATCGTCCTGGC
AGACCAGCCGAAATATACAGGCGAAGGAACGGGCATCAAGAAAATCACGGGCGGCGACCCCGTGGAAATTAACCCGAAATATG
AAAAGCGTTTTACGGCGGTAATCAGGGCGGTGGTGCTGGCAACCAATAACAATCCGATGATATTCACCGAACGGGCCGGAGGT
GTGGCACGTCGTCGGGTGATATTCCGGTTCGATAACATCGTAAGCGAGGCAGAAAAAGACAGGGAGCTACCGGAAAAGATCGC
GGCTGAAATCCCTGTCATTATCCGCCGCTTGCTGGCGAACTTTGCCGACCCTGAAAAGGCACGGGCTTTACTCATTGAACAGC
GTGACGGTGATGAAGCACTGGCAATAAAGCAACAGACGGATCCGGTTATTGAGTTTTGCCAGTTCCTGAATTTTCTGGAGGAA
GCACGCGGCCTGATGATGGGCGGCGGTGGCGATTCAGTGAAGTACACGACCAGAAACAGCCTTTACCGCGTCTATCTGGCGTT
TATGGCGTACGCAGGCAGGAGCAAACCGCTAAACGTAAATGACTTTGGCAAGGCTATGAAGCCAGCCGCGAAAGTTTACGGAC
ATGAATATATTACGCGGAAAGTTAAAGGAGTAACGCAGACTAACGCAATAACAACAGACGATTGCGACGCGTTTTTATAATGA
CGCATCCTCACGATAATATCCGGGTAGGACGAACAATAAGGCCGCAAATCGCGGCCTTTTTTATTGATAACAAAAGGACAGTT
TTCCCTTTGATATGTAACGGTGAACAGTTGTTCTACTTTTGTTTGTTAGTCTTGATGCTTCACTGATAGATACAAGAGCCATA
AGAACC

p2770

CGCAGGATAAGTAAGGGGAGTAAGTGATCGAACGAATCAGAAGTGACAATATACTTAGGCTGGATCTCGTCCCGTGAATCCCA
ACCCTCACCAACTACGAGATAAGAGGTAAGCCAAAAATCGACTTGGTGGCGACCAACGACTGTTCCCCCCCTGTAACTAATCG
TTCCGTCAAAACCTGACTTACTTCAAGGCCAATTCCAAGCGCAAACAATACCGTCCTAGTTCTTCGGTTAAGTTTCCGAAGTA
GGAGTGAGCCTACCTCCGTTTGCGTCTTGTTACCACTGACCCAGCTATTTACTTTGTATTGCCTGCAATCGAATTTCTGAACT
CTCAGATAGTGGGGATAACGGGAAAGTTCCTATATTTGCGAACTAACTTAGCCGTCCACCTCGAAGCTACCTACTCACACCCA
CCCCGCGCGGGGTAAATAAGGCACTAATCCCAGCTGAGAGCTGGCGTAGCACTTAGCCACAAGTTAATTAACAGTTGTCTGGT
AGTTTGGCGGTATTAGGAAGATCCTAGAAGCAAGGCAGAGTTAGTTCTAACCTAAAGCCACAAATAAGACAGGTTGCCAAAGC

CCGCCGGAAATTAAATCTTGCTCAGTTCGGTAACGGAGTTTCCCTCCCGCGTACTTAATTCCCAATAAGAAACGCGCCCAAGT
CCTATCAGGCAAAATTCAGCCCCTTCCCGTGTTAGAACGAGGGTAAAAATACAAGCCGATTGAACAAGGGTTGGGGGCTTCAA
ATCGTCGTTTACCCCACTTTACAACGGAGATTAAGTAGTTCACCCTATAGTACGAAGCAGAACTATTTCGAGGGGCGTGCAAT
AATCGAATCTTCTGCGGTTGACTTAACACGCTAGGGACGTGCCCTCGATTCAATCGAAGGTACTCCTACTCAGACTGCCTCAC
ACCCAGCTAGTCACTGAGCGATAAAATTGACCCGCCCTCTAGGGAAGCGAGTACGTCCCAAAGGGCTCCGGACAGGGCTATAT
AGGAGAGTTTGATCTCGCCCCGACAACTGCAACCCTCAACTCCCTTAGATAATATTGTTAGCCGAAGTTGCACGACCCGCCGT
CCACGGACTGCTCTTAGGGTGTGGCTCCTTAATCTGACAACGTGCAACCCCTATCGAAGTCGATTGTTTCTGCGAAAGGTGTT
GTCCTAATAGTCCCGAAATTTGGCCCTTGTAGGTGTGAAACCACTTAGCTTCGCGCCGTAGTCCTAAAGGCCCACCTATTGAC
TTTGTTTCGGGTAGCACTAGGAATCTTAACAATTTGAATTTGGACGTGGAACGCGTACACCTTAATCTCCGAATAATTCTAGG
GATTTGGAAGTCCTCTACGTTGACACACCTACACTGCTCGAAGTAAATATACGAATAACGCGGGCCTCGCGGAGCCGTTCCGA
ATCGTCACGTGTTCGTTTACTGTTAATTGGTGGCAAATAAGCAATATCGTAGTCCGTCAGGCCCAGCCCTGTTATCCACGGCG
TTATTTGTCAAATTGCGTAGAACTGGATTGACTGCCTGACAATACCTAATTATCGGTACGAAGTCCCCGAATCTGTCGGGCTA
TTTCACTAATACTTTCCAAACGCCCCGTATCCAAGAAGAACGAATTTATCCACGCTCCCGTCTTTGGGACGAATACCGCTACA
AGTGGACAGAGGATCGGTACGGGCCTCTAATAAATCCAACACTCTACGCCCTCTTCAAGAGCTAGAAGAACAGGGTGCAGTTG
GAAAGGGAATTATTTCGTAAGGCGAGCCAATACCGTAATTAATTCGGAAGAGTTAACACGATTGGAAGTAGGAATAGTTTCTA
ACCACGGTTACTAATCCTAATAACGGAACGCTGTCTGATAGATTAGTGTCAGCGCTCGGTACCAAAGAAAAATAAAAAGACGC
TGAAAAGCGTCTTTTTATTTTTCGGTCCAGTGTAACTCAGGCAAAAGCACGTAATATTCGTACTTTCTTCCTCCGTAAGCGTC
ACCCACATTCCTTAAAGAGTGCATGTGCATATTTTGTTATCAATAAAAAAGGCCGCGATTTGCGGCCTTATTGTTCGTCTTGC
CGGATTACGCCCCGCCCTGCCACTCATCGCAGTATTGTTGTAATTCATTAAGCATTCTGCCGACATGGAAGCCATCACAAACG
GCATGATGAACTTGGATCGCCAGTGGCATTAACACCTTGTCGCCTTGCGTATAATATTTTCCCATAGTGAAAACGGGGGCGAA
GAAGTTGTCCATATTTGCTACGTTTAAATCAAAACTGGTGAAACTCACCCACGGATTGGCACTGACGAAAAACATATTTTCGA
TAAACCCTTTAGGGAAATATGCTAAGTTTTCACCGTAACACGCCACATCTTGACTATATATGTGTAGAAACTGCCGGAAATCG
TCGTGGTATTCTGACCAGAGCGATGAAAACGTTTCAGTTTGCTCATGGAAAACGGTGTAACAAGGGTGAACACTATCCCATAT
CACCAGCTCACCGTCTTTCATTGCCATACGAAACTCCGGATGTGCATTCATCAGGCGGGCAAGAATGTGAATAAAGGCCGGAT
AAAACTTGTGCTTATTTTTCTTTACGGTTTTTAAAAAGGCCGTAATATCCAGCTGAACGGTTTGGTTATAGGTGCACTGAGCA
ACTGACTGGAATGCCTCAAAATGTTCTTTACGATGCCATTGACTTATATCAACTGTAGTATATCCAGTGATTTTTTTCTCCAT
TTTAGCTTCCTTAGCTTGCGAAATCTCGATAACTCAAAAAATAGTAGTGATCTTATTTCATTATGGTGAAAGTTGTCTTACGT
GCAACATTTTCGCAAAAAGTTGGCGCTTTATCAACACTGTCGGAATGACAAATGGTTCCAATTATTGAACACCCTTCGGGGTG
TTTTTTTGTTTCTGGTTTCCCGAGGCCGGCCTGCGCTAGCGGAGTGTATACTGGCTTACTATGTTGGCACTGATGAGGGTGTA
AGTGAAGTGCTTCATGTGGCAGGAGAAAAAAGGCTGCATCGGTGCGTCAGCAGAATATGTGATACAGGATATATTCCGCTTCC
TCGCTCACTGACTCGCTACGCTCGGTCGTTCGACTGTGGCGAGCGGAAATGGCTTACGAACGGGGCGGAGATTTCCTGGAAGA
TGCCAGGAAGATACTTAACAGGGAAGTGAGAGGGTCGCGGCAAAGCCGTTTTTCCATAGGCTCCGCCCCCCTGACAAGCATCA
CGAAATCTGACGCTCAAATCAGTGGTGGCGAAACCTGACAGGACTATAAAGATACCAGGCGTTTCCCCCTGGCGGCTCCCTCG
TGCGCTCTCCTGTTCCTGCCTTTCGGTTTGCCGGTGTCATTCCTCTGTTACGGCCGAGTTTGTCTCATTCCACGCCTGACACT
CAGTTCCGGGTAGGCAGTTCGCTCCAAGCTGGACTGTATGCACGAACCCCCCGTTCAGTCCGACCGCTGCGCCTTATCCGGTA
ACTATCGTCTTGAGTCCAACCCGGAAAGACATGCAAAAGCACCACTGGCAGCAGCCACTGGTAATTGATTTAGAGGAGTTAGT
CTTGAAGTCATGCGCCGGATAAGGCTAAACTGAAAGGACAAGTTTTGGCGACTGCGCTCCTCCAAGCCAGTTACCTCGGTTCA
AAGAGTTGGTAGCTCAGAGAACCTTCGAAAAACCGCCCTGCAAGGCGGTTTTTTCGTTTTCAGAGCAAGAGATTACGCGCAGA
CCAAAACGATCTCAAGAAGATCATCTTATTAATCAGATAAAATATTTCTAGATTTCAGTGCAATTTATCTCTTCAAATGTAGC
ACCGGCGCGCCGTGACCAATTATTGAAGGCCGCTAACGCGGCCTTTTTTTGTTTCTGGTATCCCGAATGGAGCGACTTCTCCC
CAAAAAGCCTCGCTTTCAGCACCTGTCGTTTCCTTTCTTTTCAGAGGGTATTTTAAATAAAAACATTAAGTTATGACGAAGAA
GAACGGAAACGCCTTAAACCGGAAAATTTTCATAAATAGCGAAAACCCGCGAGGTCGCCGCCCCGTAACCTGTCGGATCACCG
GAAAGGACCCGTAAAGTGATAATGATTATCATCTACATATCACAACGTGCGTAAAGGGTAAGTATGAAGGTCGTGTACTCCAT
CGCTACCAAATTCCAGAAAACAGACGCTTTCGAGCGTCTTTTTTCGTTTTGGTCACGACGTACGGTGGAAGATTCGTTACCAA
TTGACAGCTAGCTCAGTCCTAGGTATATACATACATGCTTGTTTGTTTGTAAACCAGGATAAAGAGAGAGCATACTCGATGAA
ACGTACTGCCGACGGTTCAGAGTTCGAGTCGCCCAAGAAGAAACGTAAAGTGTCATCCAAAACCGGCCCAGTTGCGGTGGACC
CAACGCTGCGCCGTCGTATCGAACCGCACGAGTTCGAAGTATTTTTCGATCCTCGCGAACTGCGTAAAGAGACCTGTTTACTT
TACGAAATCAATTGGGGTGGCCGCCACTCGATTTGGCGCCACACGTCTCAGAATACAAACAAACATGTCGAAGTAAATTTCAT
CGAAAAATTTACAACGGAGCGTTATTTCTGTCCCAATACTCGTTGTTCTATTACATGGTTTTTATCATGGAGTCCCTGCGGGG
AATGCTCTCGTGCGATCACGGAGTTTCTGTCGCGTTATCCGAACGTAACGCTTTTTATCTATATTGCTCGTCTTTACCACTTG
GCTAACCCTCGTAACCGCCAAGGACTTCGCGACCTGATTTCGAGTGGAGTAACCATCCAGATCATGACTGAGCAAGAATCTGG
ATACTGTTGGCACAATTTCGTAAACTACTCGCCATCGAATGAGAGCCATTGGCCTCGCTACCCCCATCTTTGGGTACGTTTGT
ATGTCTTGGAACTTTATTGTATCATTTTAGGGCTGCCACCTTGTCTTAATATTTTGCGCCGCAAACAGAGTCAGCTGACATCT
TTCACAATTGCTTTACAGTCATGCCACTACCAACGTCTTCCACCGCACATTCTGTGGCCACGGGCTTAAAATCGGGCGGTAG
CTCAGGCGGGAGTTCAGGCTCAGAAACGCCGGGAACTAGCGAATCCGCAACGCCAGAGTCTTCAGGTGGTTCGTCAGGTGGTT
CAGACAAGAAATATAGCATCGGCCTGGCCATCGGCACAAATAGCGTCGGATGGGCGGTGATCACTGATGAATATAAAGTTCCG
TCTAAAAAGTTCAAGGTACTGGGTAATACAGATCGCCATAGTATCAAAAAGAACTTAATCGGTGCGCTTCTGTTCGATTCCGG
CGAAACCGCAGAAGCAACACGTCTGAAACGCACCGCTCGTCGCCGTTACACCCGTCGTAAAAACCGCATCTGCTACCTGCAAG
AAATCTTCTCTAACGAAATGGCTAAAGTAGATGACAGCTTTTTTCACCGTCTGGAAGAATCATTTCTGGTGGAAGAAGATAAA
AAGCACGAACGTCATCCAATCTTCGGCAACATTGTGGACGAAGTAGCGTATCACGAAAAATACCCGACTATCTATCACCTGCG
CAAAAAGCTGGTCGATTCGACGGATAAGGCCGATCTGCGTCTGATCTATCTGGCCTTAGCGCATATGATTAAGTTCCGTGGTC
ATTTCCTGATCGAAGGCGACCTGAATCCAGACAACAGCGATGTAGACAAACTGTTCATCCAGCTGGTGCAAACCTATAACCAG
CTGTTTGAAGAAAACCCAATTAATGCTAGCGGTGTTGACGCGAAAGCGATCTTGTCCGCACGCCTGTCCAAATCCCGTCGTCT

GGAAAACTTAATTGCGCAACTGCCGGGTGAGAAGAAAAACGGACTGTTCGGCAATCTGATCGCTCTTAGCTTGGGACTGACCC
CGAACTTCAAAAGCAACTTCGATCTGGCAGAGGACGCAAAACTTCAACTTAGCAAAGATACGTATGACGATGACTTGGATAAC
TTACTGGCCCAGATCGGAGATCAGTACGCTGATCTGTTTCTGGCGGCAAAGAACTTATCAGACGCTATTCTCCTGTCTGATAT
TCTTCGTGTGAATACCGAAATCACCAAAGCACCGCTTTCTGCATCCATGATTAAACGCTATGACGAACATCACCAAGATCTGA
CTCTTCTGAAAGCGCTGGTACGGCAACAACTGCCGGAGAAGTACAAGGAGATCTTCTTTGACCAATCCAAAAACGGCTACGCG
GGTTATATTGACGGGGGTGCAAGCCAAGAGGAGTTCTACAAATTCATCAAGCCAATCTTAGAAAAAATGGATGGCACGGAAGA
ATTACTTGTTAAACTGAATCGTGAGGATCTGCTTCGTAAACAGCGTACCTTCGACAACGGTAGCATTCCGCACCAGATCCACT
TAGGTGAACTGCACGCTATCCTGCGTCGCCAAGAGGATTTTTACCCGTTCCTGAAAGATAATCGTGAAAAAATCGAAAAAATC
CTGACCTTTCGTATCCCGTATTATGTCGGCCCGCTGGCGCGTGGCAACTCCCGTTTCGCGTGGATGACTCGCAAATCCGAAGA
AACTATTACCCCGTGGAACTTCGAGGAAGTGGTTGACAAAGGCGCAAGCGCCCAATCCTTCATCGAGCGCATGACTAACTTTG
ATAAAAACCTGCCGAACGAAAAGGTACTGCCGAAACACTCCCTTCTGTACGAATACTTCACCGTGTACAACGAGCTGACTAAA
GTAAAGTATGTGACTGAGGGCATGCGTAAACCTGCATTCCTGAGCGGTGAACAGAAAAAAGCAATTGTTGATTTACTGTTTAA
AACCAACCGTAAAGTAACCGTTAAACAGCTGAAAGAGGACTACTTCAAGAAAATCGAATGCTTCGACTCCGTCGAGATTAGTG
GAGTTGAAGATCGTTTTAATGCAAGTTTAGGCACGTATCACGATTTATTAAAGATCATTAAAGACAAAGATTTCTTGGACAAC
GAAGAAAATGAGGACATCTTAGAGGACATCGTCCTGACCCTGACTCTGTTCGAAGATCGTGAAATGATTGAAGAACGCCTTAA
GACGTATGCTCACCTGTTTGACGATAAAGTAATGAAACAACTGAAACGTCGCCGTTATACTGGCTGGGGCCGTCTGAGCCGTA
AACTGATTAACGGTATCCGTGACAAACAGTCCGGTAAAACTATTCTGGACTTCCTGAAATCTGACGGCTTCGCAAACCGTAAC
TTCATGCAACTGATTCACGACGATTCCCTGACCTTCAAAGAGGACATCCAGAAAGCTCAGGTTTCTGGTCAAGGTGATTCTCT
GCACGAGCATATCGCCAATTTAGCAGGTAGTCCGGCGATCAAAAAAGGTATCCTGCAAACCGTGAAAGTGGTGGATGAGCTTG
TGAAAGTTATGGGTCGTCACAAACCGGAAAACATTGTTATCGAGATGGCTCGTGAAAACCAAACGACCCAGAAGGGACAGAAA
AACTCCCGCGAACGCATGAAACGTATCGAGGAGGGTATTAAAGAACTTGGCTCTCAGATTCTGAAAGAACACCCTGTTGAAAA
TACCCAACTGCAAAATGAAAAACTGTACCTGTACTACCTGCAAAATGGTCGTGACATGTATGTAGATCAGGAGCTGGACATCA
ACCGCCTCTCCGATTACGACGTTGACCACATTGTTCCGCAGTCTTTTCTGAAAGATGATTCCATTGATAACAAAGTACTCACC
CGTAGCGATAAAAACCGTGGGAAGAGTGACAACGTTCCATCGGAAGAAGTAGTTAAGAAAATGAAGAACTATTGGCGTCAACT
GCTTAACGCGAAACTGATTACTCAACGTAAATTTGATAACCTGACCAAAGCTGAACGTGGCGGTTTGTCTGAGCTGGATAAGG
CGGGTTTTATTAAACGTCAACTGGTAGAAACTCGCCAGATTACAAAACATGTTGCTCAGATTCTGGACTCTCGTATGAACACT
AAATACGATGAAAATGACAAACTGATCCGCGAAGTTAAGGTTATTACCCTGAAATCTAAGCTGGTTTCCGACTTCCGTAAAGA
TTTCCAATTCTATAAAGTGCGCGAGATTAACAACTATCACCACGCGCACGACGCATATCTGAATGCAGTTGTTGGCACGGCAC
TGATCAAAAAATATCCGAAACTGGAAAGCGAATTTGTGTACGGCGATTATAAAGTTTACGACGTGCGCAAAATGATCGCCAAA
TCTGAACAGGAAATTGGCAAAGCAACCGCTAAATACTTTTTCTACTCAAACATTATGAATTTCTTCAAAACCGAAATCACCTT
AGCGAATGGCGAAATTCGTAAACGCCCTCTGATCGAAACCAACGGCGAAACGGGTGAGATCGTGTGGGACAAAGGTCGTGATT
TCGCTACTGTCCGCAAAGTTCTGTCCATGCCTCAAGTAAACATCGTTAAAAAGACTGAGGTACAGACTGGCGGTTTCAGCAAG
GAATCCATTCTGCCGAAACGCAACTCCGACAAACTGATCGCGCGTAAGAAAGACTGGGATCCGAAGAAATACGGTGGCTTCGA
TTCTCCAACCGTGGCATACAGCGTTCTGGTAGTCGCCAAAGTCGAAAAGGGTAAATCAAAAAAACTGAAATCAGTGAAAGAAC
TTTTAGGCATCACCATTATGGAACGTAGCTCTTTCGAAAAAAACCCGATTGACTTCCTCGAAGCGAAGGGGTACAAGGAAGTA
AAGAAAGATCTGATTATCAAACTGCCGAAGTATTCCCTGTTCGAACTGGAAAATGGTCGTAAACGTATGTTAGCGTCTGCGGG
TGAACTGCAAAAAGGGAACGAATTGGCCCTTCCGTCCAAGTACGTGAACTTCCTGTATCTGGCCTCGCACTACGAGAAACTGA
AAGGTAGTCCGGAAGATAATGAGCAGAAACAGCTGTTCGTGGAACAGCACAAACACTATCTGGACGAGATTATTGAACAGATT
TCTGAGTTTAGCAAACGCGTAATTCTGGCGGACGCGAATCTGGATAAAGTCCTGAGCGCCTACAATAAACACCGTGATAAACC
GATCCGTGAACAGGCAGAAAACATCATTCACCTGTTCACGCTGACTAATCTTGGTGCTCCGGCAGCCTTCAAATACTTCGACA
CCACGATCGATCGTAAACGTTACACCTCCACTAAAGAAGTCTTAGATGCAACTCTTATTCACCAGAGCATCACTGGCCTGTAT
GAAACTCGTATTGATCTGAGTCAGTTGGGCGGTGACAGTGGGGGCAGCGGAGGTAGCGGAGGCAGCACTAATTTGTCGGATAT
TATTGAGAAGGAAACAGGAAAGCAATTGGTTATCCAGGAATCCATTTTAATGCTTCCGGAAGAAGTGGAAGAGGTGATCGGAA
ATAAGCCAGAGAGCGATATCTTAGTTCATACGGCCTATGATGAAAGCACTGACGAGAACGTTATGCTGTTAACATCGGACGCT
CCCGAGTACAAGCCGTGGGCCCTGGTTATCCAGGACAGTAATGGCGAAAATAAAATTAAAATGTTATAATGTATGCTTAAGCA
GCTCGGTACCAAAGACGAACAATAAGACGCTGAAAAGCGTCTTTTTTCGTTTTGGTCCTGTTGCGGCGCGATAGTGTGAACAT
GCTATAGACTTCTGGTGCTACCCGACTGACAATTAATCATCCGGCTCGTATAATGCTAGCTGAAGAGCTTTCGCTCTTCAGTT
TTAGAGCTAGAAATAGCAAGTTAAAATAAGGCTAGTCCGTTATCAACTTGAAAAAGTGGCACCGAGTCGGTGCTTTTTTTAAT
CCATTCGAATCACGTCCGACGAACAATAAGGCCTCCCTAACGGGGGGCCTTTTTTATTGATAACAAAAGTAACTTCGAGCTTG
TCTACCTCCTAGCACCATTATTGCAATTAATAAACAACTAACGGACAATTCTACCTAACAGTTTTCATATATGACGAGCAGTT
AAGTGATGAGTAAAGGTGAGGAATTATTTACTGGTGTTGTTCCGATCTTAGTTGAACTGGACGGCGATGTTAACGGTCATAAA
TTCAGTGTTCGTGGTGAAGGTGAAGGTGATGCAACCAACGGTAAGCTGACCCTGAAATTCATCTGCACTACTGGAAAATTACC
AGTACCGTGGCCTACTCTGGTGACTACCCTGACCTATGGTGTTCAGTGTTTTTCTCGTTACCCTGACCACATGAAGCAACATG
ATTTCTTCAAATCTGCAATGCCGGAAGGTTATGTACAGGAGCGCACCATTTCTTTCAAAGACGATGGCACGTATAAAACCCGT
GCAGAGGTTAAATTTGAAGGTGACACTCTGGTGAATCGTATTGAACTGAAAGGCATTGATTTCAAAGAGGACGGCAATATTTT
AGGCCACAAACTGGAATATAACTTCAACTCCCATAACGTTTACATCACCGCAGACAAACAGAAGAACGGTATCAAAGCTAACT
TCAAAATTCGCCATAACGTTGAAGATGGTAGCGTACAGCTGGCGGATCATTACCAACAGAACACTCCGATTGGAGATGCTCCT
GTTTTACTGCCGGATAACCACTACCTGTCCACCCAGTCTAAACTGTCGAAGGATCCGAACGAAAAGCGCGACCACATGGTGTT
ATTAGAGTTCGTTACCGCTAGTGGTATCACGCACGGTATGGATGAACTCTACAAATAAGACGAACAATAAGGGGAGCGGGAAA
CCGCTCCCCTTTTTTATTGATAACAAAAGTAAATTGCACGCTGATAGTCTCCCAATTGCGAAGGACCAAAACGAAAAAACACC
CTTTCGGGTGTCTTTTCTGGAATTTGGTACCGAGTACTAGGTATCGTGTAAGTAGCGAAGGCCCGTACGCGAGATAAACTGCT
AGGCAACCGCGACTCTACGACTGGTGCTCGATTTAATTTCGCTGACGTAAAGAAATTATCGGCAGTGCGTCAACTGCCGTATC
TTTATCTTAATTAGGTAGTTGGACAAGCCCTTGAAAGAAATAGCAAGAGCCTGCCTCTCTATTGAAGTCACGGCGAAAGTCGG

GTAGAAATCAAAGAAAGCAGAAATTAAATCGGAGTAACACTAAGGTGGGATAACTCCGTAACTGACTACGCCTTTCTCTAGAC
TTTACTTGACCAGATACACTGTCTTTGACACGTTGAAGGATTAGAGCAATCAAATCCAAGACTGGCTAAGCACGAAGCAACTC
TTGAGTGTTAAAAAGTTATCTCCTGTATTCGGGAAGCGGGTACTAGAAGATTGCAGGGACTCCGACGTTAAGTAAATTACAAA
GTAATAAGTATCGTTCAGGATCACGTTACCGCAATAAGAAGCGAGAATAATATAATTTCCGAAGTGCTTACCCCAGTAGTGAC
TATTCCTATAACCCTTCTGAGTGTCCGGAGGCGGAAATTTGCCACGAAAGAGAAAGTATTTCCCCGACAATAATAAAGGGGCG
CTCCTCAGCTTTTCCACTTGGTTGGGTAAGCTAGGCAACTCTGAAAGGAGTTTCGGCGAATTGAAGCCGACAGCTTTGAATTG
TTTTAGGGGCGTTATTCGAGGGCAATCGGAGCTAACTTCAAGACTACTTCTTTGTTGAATACTAAATAGTGCAAAGGTCGTGT
TTCCTCAAGGATACTCCGCTAACAATATAGGATTCCAATCAGATTCAGCACTGGCGGTACGGGTGTTGCGGTGAGGCGTTCGG
GTTTACGGCTCGAAGCTAGCACGGTAGG

p2771

CGCAGGATAAGTAAGGGGAGTAAGTGATCGAACGAATCAGAAGTGACAATATACTTAGGCTGGATCTCGTCCCGTGAATCCCA
ACCCTCACCAACTACGAGATAAGAGGTAAGCCAAAAATCGACTTGGTGGCGACCAACGACTGTTCCCCCCCTGTAACTAATCG
TTCCGTCAAAACCTGACTTACTTCAAGGCCAATTCCAAGCGCAAACAATACCGTCCTAGTTCTTCGGTTAAGTTTCCGAAGTA
GGAGTGAGCCTACCTCCGTTTGCGTCTTGTTACCACTGACCCAGCTATTTACTTTGTATTGCCTGCAATCGAATTTCTGAACT
CTCAGATAGTGGGGATAACGGGAAAGTTCCTATATTTGCGAACTAACTTAGCCGTCCACCTCGAAGCTACCTACTCACACCCA
CCCCGCGCGGGGTAAATAAGGCACTAATCCCAGCTGAGAGCTGGCGTAGCACTTAGCCACAAGTTAATTAACAGTTGTCTGGT
AGTTTGGCGGTATTAGGAAGATCCTAGAAGCAAGGCAGAGTTAGTTCTAACCTAAAGCCACAAATAAGACAGGTTGCCAAAGC
CCGCCGGAAATTAAATCTTGCTCAGTTCGGTAACGGAGTTTCCCTCCCGCGTACTTAATTCCCAATAAGAAACGCGCCCAAGT
CCTATCAGGCAAAATTCAGCCCCTTCCCGTGTTAGAACGAGGGTAAAAATACAAGCCGATTGAACAAGGGTTGGGGGCTTCAA
ATCGTCGTTTACCCCACTTTACAACGGAGATTAAGTAGTTCACCCTATAGTACGAAGCAGAACTATTTCGAGGGGCGTGCAAT
AATCGAATCTTCTGCGGTTGACTTAACACGCTAGGGACGTGCCCTCGATTCAATCGAAGGTACTCCTACTCAGACTGCCTCAC
ACCCAGCTAGTCACTGAGCGATAAAATTGACCCGCCCTCTAGGGAAGCGAGTACGTCCCAAAGGGCTCCGGACAGGGCTATAT
AGGAGAGTTTGATCTCGCCCCGACAACTGCAACCCTCAACTCCCTTAGATAATATTGTTAGCCGAAGTTGCACGACCCGCCGT
CCACGGACTGCTCTTAGGGTGTGGCTCCTTAATCTGACAACGTGCAACCCCTATCGAAGTCGATTGTTTCTGCGAAAGGTGTT
GTCCTAATAGTCCCGAAATTTGGCCCTTGTAGGTGTGAAACCACTTAGCTTCGCGCCGTAGTCCTAAAGGCCCACCTATTGAC
TTTGTTTCGGGTAGCACTAGGAATCTTAACAATTTGAATTTGGACGTGGAACGCGTACACCTTAATCTCCGAATAATTCTAGG
GATTTGGAAGTCCTCTACGTTGACACACCTACACTGCTCGAAGTAAATATACGAATAACGCGGGCCTCGCGGAGCCGTTCCGA
ATCGTCACGTGTTCGTTTACTGTTAATTGGTGGCAAATAAGCAATATCGTAGTCCGTCAGGCCCAGCCCTGTTATCCACGGCG
TTATTTGTCAAATTGCGTAGAACTGGATTGACTGCCTGACAATACCTAATTATCGGTACGAAGTCCCCGAATCTGTCGGGCTA
TTTCACTAATACTTTCCAAACGCCCCGTATCCAAGAAGAACGAATTTATCCACGCTCCCGTCTTTGGGACGAATACCGCTACA
AGTGGACAGAGGATCGGTACGGGCCTCTAATAAATCCAACACTCTACGCCCTCTTCAAGAGCTAGAAGAACAGGGTGCAGTTG
GAAAGGGAATTATTTCGTAAGGCGAGCCAATACCGTAATTAATTCGGAAGAGTTAACACGATTGGAAGTAGGAATAGTTTCTA
ACCACGGTTACTAATCCTAATAACGGAACGCTGTCTGATAGATTAGTGTCAGCGCTCGGTACCAAAGAAAAATAAAAAGACGC
TGAAAAGCGTCTTTTTATTTTTCGGTCCAGTGTAACTCAGGCAAAAGCACGTAATATTCGTACTTTCTTCCTCCGTAAGCGTC
ACCCACATTCCTTAAAGAGTGCATGTGCATATTTTGTTATCAATAAAAAAGGCCGCGATTTGCGGCCTTATTGTTCGTCTTGC
CGGATTACGCCCCGCCCTGCCACTCATCGCAGTATTGTTGTAATTCATTAAGCATTCTGCCGACATGGAAGCCATCACAAACG
GCATGATGAACTTGGATCGCCAGTGGCATTAACACCTTGTCGCCTTGCGTATAATATTTTCCCATAGTGAAAACGGGGGCGAA
GAAGTTGTCCATATTTGCTACGTTTAAATCAAAACTGGTGAAACTCACCCACGGATTGGCACTGACGAAAAACATATTTTCGA
TAAACCCTTTAGGGAAATATGCTAAGTTTTCACCGTAACACGCCACATCTTGACTATATATGTGTAGAAACTGCCGGAAATCG
TCGTGGTATTCTGACCAGAGCGATGAAAACGTTTCAGTTTGCTCATGGAAAACGGTGTAACAAGGGTGAACACTATCCCATAT
CACCAGCTCACCGTCTTTCATTGCCATACGAAACTCCGGATGTGCATTCATCAGGCGGGCAAGAATGTGAATAAAGGCCGGAT
AAAACTTGTGCTTATTTTTCTTTACGGTTTTTAAAAAGGCCGTAATATCCAGCTGAACGGTTTGGTTATAGGTGCACTGAGCA
ACTGACTGGAATGCCTCAAAATGTTCTTTACGATGCCATTGACTTATATCAACTGTAGTATATCCAGTGATTTTTTTCTCCAT
TTTAGCTTCCTTAGCTTGCGAAATCTCGATAACTCAAAAAATAGTAGTGATCTTATTTCATTATGGTGAAAGTTGTCTTACGT
GCAACATTTTCGCAAAAAGTTGGCGCTTTATCAACACTGTCGGAATGACAAATGGTTCCAATTATTGAACACCCTTCGGGGTG
TTTTTTTGTTTCTGGTTTCCCGAGGCCGGCCTGCGCTAGCGGAGTGTATACTGGCTTACTATGTTGGCACTGATGAGGGTGTA
AGTGAAGTGCTTCATGTGGCAGGAGAAAAAAGGCTGCATCGGTGCGTCAGCAGAATATGTGATACAGGATATATTCCGCTTCC
TCGCTCACTGACTCGCTACGCTCGGTCGTTCGACTGTGGCGAGCGGAAATGGCTTACGAACGGGGCGGAGATTTCCTGGAAGA
TGCCAGGAAGATACTTAACAGGGAAGTGAGAGGGTCGCGGCAAAGCCGTTTTTCCATAGGCTCCGCCCCCCTGACAAGCATCA
CGAAATCTGACGCTCAAATCAGTGGTGGCGAAACCTGACAGGACTATAAAGATACCAGGCGTTTCCCCCTGGCGGCTCCCTCG
TGCGCTCTCCTGTTCCTGCCTTTCGGTTTGCCGGTGTCATTCCTCTGTTACGGCCGAGTTTGTCTCATTCCACGCCTGACACT
CAGTTCCGGGTAGGCAGTTCGCTCCAAGCTGGACTGTATGCACGAACCCCCCGTTCAGTCCGACCGCTGCGCCTTATCCGGTA
ACTATCGTCTTGAGTCCAACCCGGAAAGACATGCAAAAGCACCACTGGCAGCAGCCACTGGTAATTGATTTAGAGGAGTTAGT
CTTGAAGTCATGCGCCGGATAAGGCTAAACTGAAAGGACAAGTTTTGGCGACTGCGCTCCTCCAAGCCAGTTACCTCGGTTCA
AAGAGTTGGTAGCTCAGAGAACCTTCGAAAAACCGCCCTGCAAGGCGGTTTTTTCGTTTTCAGAGCAAGAGATTACGCGCAGA
CCAAAACGATCTCAAGAAGATCATCTTATTAATCAGATAAAATATTTCTAGATTTCAGTGCAATTTATCTCTTCAAATGTAGC
ACCGGCGCGCCGTGACCAATTATTGAAGGCCGCTAACGCGGCCTTTTTTTGTTTCTGGTATCCCGAATGGAGCGACTTCTCCC

CAAAAAGCCTCGCTTTCAGCACCTGTCGTTTCCTTTCTTTTCAGAGGGTATTTTAAATAAAAACATTAAGTTATGACGAAGAA
GAACGGAAACGCCTTAAACCGGAAAATTTTCATAAATAGCGAAAACCCGCGAGGTCGCCGCCCCGTAACCTGTCGGATCACCG
GAAAGGACCCGTAAAGTGATAATGATTATCATCTACATATCACAACGTGCGTAAAGGGTAAGTATGAAGGTCGTGTACTCCAT
CGCTACCAAATTCCAGAAAACAGACGCTTTCGAGCGTCTTTTTTCGTTTTGGTCACGACGTACGGTGGAAGATTCGTTACCAA
TTGACAGCTAGCTCAGTCCTAGGTATATACATACATGCTTGTTTGTTTGTAAACGGAGATAAATGGTGAATGCGAGATGAAGC
GTACCGCCGATGGCAGCGAGTTTGAATCTCCGAAGAAAAAGCGTAAGGTCAGCGAAGTTGAGTTCAGCCACGAATACTGGATG
CGTCACGCTTTAACCCTGGCTAAACGCGCGCGCGACGAGCGCGAAGTACCAGTGGGGGCGGTGCTGGTGTTAAACAACCGCGT
AATCGGCGAAGGCTGGAACCGTGCAATCGGGTTACATGACCCGACCGCCCATGCCGAGATCATGGCCCTGCGCCAGGGGGGGC
TGGTCATGCAGAATTACCGTCTGATCGACGCGACGTTGTATGTCACATTCGAGCCATGCGTGATGTGTGCGGGGGCAATGATT
CACTCTCGCATTGGTCGCGTCGTGTTTGGCGTTCGTAATAGTAAACGCGGCGCTGCTGGCTCCTTAATGAATGTTCTGAATTA
TCCGGGTATGAACCACCGTGTCGAAATTACAGAAGGTATCTTAGCAGATGAATGTGCCGCACTGCTGTGTGACTTCTACCGCA
TGCCGCGCCAAGTATTCAACGCCCAAAAAAAAGCTCAGTCCTCAATTAACTCTGGTGGTAGTAGTGGCGGCTCTAGCGGCTCC
GAGACGCCTGGTACGTCGGAATCGGCTACGCCTGAGTCGAGCGGTGGGTCCTCTGGCGGCTCTGACAAGAAATATAGCATCGG
CCTGGCCATCGGCACAAATAGCGTCGGATGGGCGGTGATCACTGATGAATATAAAGTTCCGTCTAAAAAGTTCAAGGTACTGG
GTAATACAGATCGCCATAGTATCAAAAAGAACTTAATCGGTGCGCTTCTGTTCGATTCCGGCGAAACCGCAGAAGCAACACGT
CTGAAACGCACCGCTCGTCGCCGTTACACCCGTCGTAAAAACCGCATCTGCTACCTGCAAGAAATCTTCTCTAACGAAATGGC
TAAAGTAGATGACAGCTTTTTTCACCGTCTGGAAGAATCATTTCTGGTGGAAGAAGATAAAAAGCACGAACGTCATCCAATCT
TCGGCAACATTGTGGACGAAGTAGCGTATCACGAAAAATACCCGACTATCTATCACCTGCGCAAAAAGCTGGTCGATTCGACG
GATAAGGCCGATCTGCGTCTGATCTATCTGGCCTTAGCGCATATGATTAAGTTCCGTGGTCATTTCCTGATCGAAGGCGACCT
GAATCCAGACAACAGCGATGTAGACAAACTGTTCATCCAGCTGGTGCAAACCTATAACCAGCTGTTTGAAGAAAACCCAATTA
ATGCTAGCGGTGTTGACGCGAAAGCGATCTTGTCCGCACGCCTGTCCAAATCCCGTCGTCTGGAAAACTTAATTGCGCAACTG
CCGGGTGAGAAGAAAAACGGACTGTTCGGCAATCTGATCGCTCTTAGCTTGGGACTGACCCCGAACTTCAAAAGCAACTTCGA
TCTGGCAGAGGACGCAAAACTTCAACTTAGCAAAGATACGTATGACGATGACTTGGATAACTTACTGGCCCAGATCGGAGATC
AGTACGCTGATCTGTTTCTGGCGGCAAAGAACTTATCAGACGCTATTCTCCTGTCTGATATTCTTCGTGTGAATACCGAAATC
ACCAAAGCACCGCTTTCTGCATCCATGATTAAACGCTATGACGAACATCACCAAGATCTGACTCTTCTGAAAGCGCTGGTACG
GCAACAACTGCCGGAGAAGTACAAGGAGATCTTCTTTGACCAATCCAAAAACGGCTACGCGGGTTATATTGACGGGGGTGCAA
GCCAAGAGGAGTTCTACAAATTCATCAAGCCAATCTTAGAAAAAATGGATGGCACGGAAGAATTACTTGTTAAACTGAATCGT
GAGGATCTGCTTCGTAAACAGCGTACCTTCGACAACGGTAGCATTCCGCACCAGATCCACTTAGGTGAACTGCACGCTATCCT
GCGTCGCCAAGAGGATTTTTACCCGTTCCTGAAAGATAATCGTGAAAAAATCGAAAAAATCCTGACCTTTCGTATCCCGTATT
ATGTCGGCCCGCTGGCGCGTGGCAACTCCCGTTTCGCGTGGATGACTCGCAAATCCGAAGAAACTATTACCCCGTGGAACTTC
GAGGAAGTGGTTGACAAAGGCGCAAGCGCCCAATCCTTCATCGAGCGCATGACTAACTTTGATAAAAACCTGCCGAACGAAAA
GGTACTGCCGAAACACTCCCTTCTGTACGAATACTTCACCGTGTACAACGAGCTGACTAAAGTAAAGTATGTGACTGAGGGCA
TGCGTAAACCTGCATTCCTGAGCGGTGAACAGAAAAAAGCAATTGTTGATTTACTGTTTAAAACCAACCGTAAAGTAACCGTT
AAACAGCTGAAAGAGGACTACTTCAAGAAAATCGAATGCTTCGACTCCGTCGAGATTAGTGGAGTTGAAGATCGTTTTAATGC
AAGTTTAGGCACGTATCACGATTTATTAAAGATCATTAAAGACAAAGATTTCTTGGACAACGAAGAAAATGAGGACATCTTAG
AGGACATCGTCCTGACCCTGACTCTGTTCGAAGATCGTGAAATGATTGAAGAACGCCTTAAGACGTATGCTCACCTGTTTGAC
GATAAAGTAATGAAACAACTGAAACGTCGCCGTTATACTGGCTGGGGCCGTCTGAGCCGTAAACTGATTAACGGTATCCGTGA
CAAACAGTCCGGTAAAACTATTCTGGACTTCCTGAAATCTGACGGCTTCGCAAACCGTAACTTCATGCAACTGATTCACGACG
ATTCCCTGACCTTCAAAGAGGACATCCAGAAAGCTCAGGTTTCTGGTCAAGGTGATTCTCTGCACGAGCATATCGCCAATTTA
GCAGGTAGTCCGGCGATCAAAAAAGGTATCCTGCAAACCGTGAAAGTGGTGGATGAGCTTGTGAAAGTTATGGGTCGTCACAA
ACCGGAAAACATTGTTATCGAGATGGCTCGTGAAAACCAAACGACCCAGAAGGGACAGAAAAACTCCCGCGAACGCATGAAAC
GTATCGAGGAGGGTATTAAAGAACTTGGCTCTCAGATTCTGAAAGAACACCCTGTTGAAAATACCCAACTGCAAAATGAAAAA
CTGTACCTGTACTACCTGCAAAATGGTCGTGACATGTATGTAGATCAGGAGCTGGACATCAACCGCCTCTCCGATTACGACGT
TGACCACATTGTTCCGCAGTCTTTTCTGAAAGATGATTCCATTGATAACAAAGTACTCACCCGTAGCGATAAAAACCGTGGGA
AGAGTGACAACGTTCCATCGGAAGAAGTAGTTAAGAAAATGAAGAACTATTGGCGTCAACTGCTTAACGCGAAACTGATTACT
CAACGTAAATTTGATAACCTGACCAAAGCTGAACGTGGCGGTTTGTCTGAGCTGGATAAGGCGGGTTTTATTAAACGTCAACT
GGTAGAAACTCGCCAGATTACAAAACATGTTGCTCAGATTCTGGACTCTCGTATGAACACTAAATACGATGAAAATGACAAAC
TGATCCGCGAAGTTAAGGTTATTACCCTGAAATCTAAGCTGGTTTCCGACTTCCGTAAAGATTTCCAATTCTATAAAGTGCGC
GAGATTAACAACTATCACCACGCGCACGACGCATATCTGAATGCAGTTGTTGGCACGGCACTGATCAAAAAATATCCGAAACT
GGAAAGCGAATTTGTGTACGGCGATTATAAAGTTTACGACGTGCGCAAAATGATCGCCAAATCTGAACAGGAAATTGGCAAAG
CAACCGCTAAATACTTTTTCTACTCAAACATTATGAATTTCTTCAAAACCGAAATCACCTTAGCGAATGGCGAAATTCGTAAA
CGCCCTCTGATCGAAACCAACGGCGAAACGGGTGAGATCGTGTGGGACAAAGGTCGTGATTTCGCTACTGTCCGCAAAGTTCT
GTCCATGCCTCAAGTAAACATCGTTAAAAAGACTGAGGTACAGACTGGCGGTTTCAGCAAGGAATCCATTCTGCCGAAACGCA
ACTCCGACAAACTGATCGCGCGTAAGAAAGACTGGGATCCGAAGAAATACGGTGGCTTCGATTCTCCAACCGTGGCATACAGC
GTTCTGGTAGTCGCCAAAGTCGAAAAGGGTAAATCAAAAAAACTGAAATCAGTGAAAGAACTTTTAGGCATCACCATTATGGA
ACGTAGCTCTTTCGAAAAAAACCCGATTGACTTCCTCGAAGCGAAGGGGTACAAGGAAGTAAAGAAAGATCTGATTATCAAAC
TGCCGAAGTATTCCCTGTTCGAACTGGAAAATGGTCGTAAACGTATGTTAGCGTCTGCGGGTGAACTGCAAAAAGGGAACGAA
TTGGCCCTTCCGTCCAAGTACGTGAACTTCCTGTATCTGGCCTCGCACTACGAGAAACTGAAAGGTAGTCCGGAAGATAATGA
GCAGAAACAGCTGTTCGTGGAACAGCACAAACACTATCTGGACGAGATTATTGAACAGATTTCTGAGTTTAGCAAACGCGTAA
TTCTGGCGGACGCGAATCTGGATAAAGTCCTGAGCGCCTACAATAAACACCGTGATAAACCGATCCGTGAACAGGCAGAAAAC
ATCATTCACCTGTTCACGCTGACTAATCTTGGTGCTCCGGCAGCCTTCAAATACTTCGACACCACGATCGATCGTAAACGTTA
CACCTCCACTAAAGAAGTCTTAGATGCAACTCTTATTCACCAGAGCATCACTGGCCTGTATGAAACTCGTATTGATCTGAGTC
AGTTGGGCGGTGACTAATGTATGCTTAAGCAGCTCGGTACCAAAGACGAACAATAAGACGCTGAAAAGCGTCTTTTTTCGTTT

TGGTCCTGTTGCGGCGCGATAGTGTGAACATGCTATAGACTTCTGGTGCTACCCGACTGACAATTAATCATCCGGCTCGTATA
ATGCTAGCTGAAGAGCTTTCGCTCTTCAGTTTTAGAGCTAGAAATAGCAAGTTAAAATAAGGCTAGTCCGTTATCAACTTGAA
AAAGTGGCACCGAGTCGGTGCTTTTTTTAATCCATTCGAATCACGTCCGACGAACAATAAGGCCTCCCTAACGGGGGGCCTTT
TTTATTGATAACAAAAGTAACTTCGAGCTTGTCTACCTCCTAGCACCATTATTGCAATTAATAAACAACTAACGGACAATTCT
ACCTAACAGTTTTCATATATGACGAGCAGTTAAGTGATGAGTAAAGGTGAGGAATTATTTACTGGTGTTGTTCCGATCTTAGT
TGAACTGGACGGCGATGTTAACGGTCATAAATTCAGTGTTCGTGGTGAAGGTGAAGGTGATGCAACCAACGGTAAGCTGACCC
TGAAATTCATCTGCACTACTGGAAAATTACCAGTACCGTGGCCTACTCTGGTGACTACCCTGACCTATGGTGTTCAGTGTTTT
TCTCGTTACCCTGACCACATGAAGCAACATGATTTCTTCAAATCTGCAATGCCGGAAGGTTATGTACAGGAGCGCACCATTTC
TTTCAAAGACGATGGCACGTATAAAACCCGTGCAGAGGTTAAATTTGAAGGTGACACTCTGGTGAATCGTATTGAACTGAAAG
GCATTGATTTCAAAGAGGACGGCAATATTTTAGGCCACAAACTGGAATATAACTTCAACTCCCATAACGTTTACATCACCGCA
GACAAACAGAAGAACGGTATCAAAGCTAACTTCAAAATTCGCCATAACGTTGAAGATGGTAGCGTACAGCTGGCGGATCATTA
CCAACAGAACACTCCGATTGGAGATGCTCCTGTTTTACTGCCGGATAACCACTACCTGTCCACCCAGTCTAAACTGTCGAAGG
ATCCGAACGAAAAGCGCGACCACATGGTGTTATTAGAGTTCGTTACCGCTAGTGGTATCACGCACGGTATGGATGAACTCTAC
AAATAAGACGAACAATAAGGGGAGCGGGAAACCGCTCCCCTTTTTTATTGATAACAAAAGTAAATTGCACGCTGATAGTCTCC
CAATTGCGAAGGACCAAAACGAAAAAACACCCTTTCGGGTGTCTTTTCTGGAATTTGGTACCGAGTACTAGGTATCGTGTAAG
TAGCGAAGGCCCGTACGCGAGATAAACTGCTAGGCAACCGCGACTCTACGACTGGTGCTCGATTTAATTTCGCTGACGTAAAG
AAATTATCGGCAGTGCGTCAACTGCCGTATCTTTATCTTAATTAGGTAGTTGGACAAGCCCTTGAAAGAAATAGCAAGAGCCT
GCCTCTCTATTGAAGTCACGGCGAAAGTCGGGTAGAAATCAAAGAAAGCAGAAATTAAATCGGAGTAACACTAAGGTGGATA
ACTCCGTAACTGACTACGCCTTTCTCTAGACTTTACTTGACCAGATACACTGTCTTTGACACGTTGAAGGATTAGAGCAATCA
AATCCAAGACTGGCTAAGCACGAAGCAACTCTTGAGTGTTAAAAAGTTATCTCCTGTATTCGGGAAGCGGGTACTAGAAGATT
GCAGGGACTCCGACGTTAAGTAAATTACAAAGTAATAAGTATCGTTCAGGATCACGTTACCGCAATAAGAAGCGAGAATAATA
TAATTTCCGAAGTGCTTACCCCAGTAGTGACTATTCCTATAACCCTTCTGAGTGTCCGGAGGCGGAAATTTGCCACGAAAGAG
AAAGTATTTCCCCGACAATAATAAAGGGGCGCTCCTCAGCTTTTCCACTTGGTTGGGTAAGCTAGGCAACTCTGAAAGGAGTT
TCGGCGAATTGAAGCCGACAGCTTTGAATTGTTTTAGGGGCGTTATTCGAGGGCAATCGGAGCTAACTTCAAGACTACTTCTT
TGTTGAATACTAAATAGTGCAAAGGTCGTGTTTCCTCAAGGATACTCCGCTAACAATATAGGATTCCAATCAGATTCAGCACT
GGCGGTACGGGTGTTGCGGTGAGGCGTTCGGGTTTACGGCTCGAAGCTAGCACGGTAGG

p2777

CGCAGGATAAGTAAGGGGAGTAAGTGATCGAACGAATCAGAAGTGACAATATACTTAGGCTGGATCTCGTCCCGTGAATCCCA
ACCCTCACCAACTACGAGATAAGAGGTAAGCCAAAAATCGACTTGGTGGCGACCAACGACTGTTCCCCCCCTGTAACTAATCG
TTCCGTCAAAACCTGACTTACTTCAAGGCCAATTCCAAGCGCAAACAATACCGTCCTAGTTCTTCGGTTAAGTTTCCGAAGTA
GGAGTGAGCCTACCTCCGTTTGCGTCTTGTTACCACTGACCCAGCTATTTACTTTGTATTGCCTGCAATCGAATTTCTGAACT
CTCAGATAGTGGGGATAACGGGAAAGTTCCTATATTTGCGAACTAACTTAGCCGTCCACCTCGAAGCTACCTACTCACACCCA
CCCCGCGCGGGGTAAATAAGGCACTAATCCCAGCTGAGAGCTGGCGTAGCACTTAGCCACAAGTTAATTAACAGTTGTCTGGT
AGTTTGGCGGTATTAGGAAGATCCTAGAAGCAAGGCAGAGTTAGTTCTAACCTAAAGCCACAAATAAGACAGGTTGCCAAAGC
CCGCCGGAAATTAAATCTTGCTCAGTTCGGTAACGGAGTTTCCCTCCCGCGTACTTAATTCCCAATAAGAAACGCGCCCAAGT
CCTATCAGGCAAAATTCAGCCCCTTCCCGTGTTAGAACGAGGGTAAAAATACAAGCCGATTGAACAAGGGTTGGGGGCTTCAA
ATCGTCGTTTACCCCACTTTACAACGGAGATTAAGTAGTTCACCCTATAGTACGAAGCAGAACTATTTCGAGGGGCGTGCAAT
AATCGAATCTTCTGCGGTTGACTTAACACGCTAGGGACGTGCCCTCGATTCAATCGAAGGTACTCCTACTCAGACTGCCTCAC
ACCCAGCTAGTCACTGAGCGATAAAATTGACCCGCCCTCTAGGGAAGCGAGTACGTCCCAAAGGGCTCCGGACAGGGCTATAT
AGGAGAGTTTGATCTCGCCCCGACAACTGCAACCCTCAACTCCCTTAGATAATATTGTTAGCCGAAGTTGCACGACCCGCCGT
CCACGGACTGCTCTTAGGGTGTGGCTCCTTAATCTGACAACGTGCAACCCCTATCGAAGTCGATTGTTTCTGCGAAAGGTGTT
GTCCTAATAGTCCCGAAATTTGGCCCTTGTAGGTGTGAAACCACTTAGCTTCGCGCCGTAGTCCTAAAGGCCCACCTATTGAC
TTTGTTTCGGGTAGCACTAGGAATCTTAACAATTTGAATTTGGACGTGGAACGCGTACACCTTAATCTCCGAATAATTCTAGG
GATTTGGAAGTCCTCTACGTTGACACACCTACACTGCTCGAAGTAAATATACGAATAACGCGGGCCTCGCGGAGCCGTTCCGA
ATCGTCACGTGTTCGTTTACTGTTAATTGGTGGCAAATAAGCAATATCGTAGTCCGTCAGGCCCAGCCCTGTTATCCACGGCG
TTATTTGTCAAATTGCGTAGAACTGGATTGACTGCCTGACAATACCTAATTATCGGTACGAAGTCCCCGAATCTGTCGGGCTA
TTTCACTAATACTTTCCAAACGCCCCGTATCCAAGAAGAACGAATTTATCCACGCTCCCGTCTTTGGGACGAATACCGCTACA
AGTGGACAGAGGATCGGTACGGGCCTCTAATAAATCCAACACTCTACGCCCTCTTCAAGAGCTAGAAGAACAGGGTGCAGTTG
GAAAGGGAATTATTTCGTAAGGCGAGCCAATACCGTAATTAATTCGGAAGAGTTAACACGATTGGAAGTAGGAATAGTTTCTA
ACCACGGTTACTAATCCTAATAACGGAACGCTGTCTGATAGATTAGTGTCAGCGCTCGGTACCAAAGAAAAATAAAAAGACGC
TGAAAAGCGTCTTTTTATTTTTCGGTCCAGTGTAACTCAGGCAAAAGCACGTAATATTCGTACTTTCTTCCTCCGTAAGCGTC
ACCCACATTCCTTAAAGAGTGCATGTGCATATTTTGTTATCAATAAAAAAGGCCGCGATTTGCGGCCTTATTGTTCGTCTTGC
CGGATTACGCCCCGCCCTGCCACTCATCGCAGTATTGTTGTAATTCATTAAGCATTCTGCCGACATGGAAGCCATCACAAACG
GCATGATGAACTTGGATCGCCAGTGGCATTAACACCTTGTCGCCTTGCGTATAATATTTTCCCATAGTGAAAACGGGGGCGAA
GAAGTTGTCCATATTTGCTACGTTTAAATCAAAACTGGTGAAACTCACCCACGGATTGGCACTGACGAAAAACATATTTTCGA
TAAACCCTTTAGGGAAATATGCTAAGTTTTCACCGTAACACGCCACATCTTGACTATATATGTGTAGAAACTGCCGGAAATCG
TCGTGGTATTCTGACCAGAGCGATGAAAACGTTTCAGTTTGCTCATGGAAAACGGTGTAACAAGGGTGAACACTATCCCATAT
CACCAGCTCACCGTCTTTCATTGCCATACGAAACTCCGGATGTGCATTCATCAGGCGGGCAAGAATGTGAATAAAGGCCGGAT

AAAACTTGTGCTTATTTTTCTTTACGGTTTTTAAAAAGGCCGTAATATCCAGCTGAACGGTTTGGTTATAGGTGCACTGAGCA
ACTGACTGGAATGCCTCAAAATGTTCTTTACGATGCCATTGACTTATATCAACTGTAGTATATCCAGTGATTTTTTTCTCCAT
TTTAGCTTCCTTAGCTTGCGAAATCTCGATAACTCAAAAAATAGTAGTGATCTTATTTCATTATGGTGAAAGTTGTCTTACGT
GCAACATTTTCGCAAAAAGTTGGCGCTTTATCAACACTGTCGGAATGACAAATGGTTCCAATTATTGAACACCCTTCGGGGTG
TTTTTTTGTTTCTGGTTTCCCGAGGCCGGCCTTTTGTTGCAATGGCTGTCTACCCTGTCTACCTGAGTAAAGAAAAATACATT
TAATTCAGTATATTAACTTGGGTAGACAGCCTTTTTTTACTGTCTACCTTCTGTCTACCCTCTCTACCTGATTTTACCTGAAT
CAGACAGGGAGGTAGACACGGGGTAGACAGTGGATAAAAGCACTCTACCCCACTGAAAGCAGTGCCATTACTGGCATGGTTGC
CAGTAAGGTTGATAAGGTAGACAAGGGGAGGGACAACTCAAAACTTTTTAAACGAGGGGGTAAAACGCAGACCAAAACGATCT
CAAGAAGATCATCTTATTAATCAGATAAAATATTTCTAGATTTCAGTGCAATTTATCTCTTCAAATGTAGCACCGGCGCGCCG
TGACCAATTATTGAAGGCCGCTAACGCGGCCTTTTTTTGTTTCTGGTATCCCGAATGGAGCGACTTCTCCCCAAAAAGCCTCG
CTTTCAGCACCTGTCGTTTCCTTTCTTTTCAGAGGGTATTTTAAATAAAAACATTAAGTTATGACGAAGAAGAACGGAAACGC
CTTAAACCGGAAAATTTTCATAAATAGCGAAAACCCGCGAGGTCGCCGCCCCGTAACCTGTCGGATCACCGGAAAGGACCCGT
AAAGTGATAATGATTATCATCTACATATCACAACGTGCGTAAAGGGTAAGTATGAAGGTCGTGTACTCCATCGCTACCAAATT
CCAGAAAACAGACGCTTTCGAGCGTCTTTTTTCGTTTTGGTCACGACGTACGGTGGAAGATTCGTTACCAATTGACAGCTAGC
TCAGTCCTAGGTATATACATACATGCTTGTTTGTTTGTAAACCAGGATAAAGAGAGAGCATACTCGATGAAACGTACTGCCGA
CGGTTCAGAGTTCGAGTCGCCCAAGAAGAAACGTAAAGTGTCATCCAAAACCGGCCCAGTTGCGGTGGACCCAACGCTGCGCC
GTCGTATCGAACCGCACGAGTTCGAAGTATTTTTCGATCCTCGCGAACTGCGTAAAGAGACCTGTTTACTTTACGAAATCAAT
TGGGGTGGCCGCCACTCGATTTGGCGCCACACGTCTCAGAATACAAACAAACATGTCGAAGTAAATTTCATCGAAAAATTTAC
AACGGAGCGTTATTTCTGTCCCAATACTCGTTGTTCTATTACATGGTTTTTATCATGGAGTCCCTGCGGGGAATGCTCTCGTG
CGATCACCGAGTTTCTGTCGCGTTATCCGAACGTAACGCTTTTTATCTATATTGCTCGTCTTTACCACTTGGCTAACCCTCGT
AACCGCCAAGGACTTCGCGACCTGATTTCGAGTGGAGTAACCATCCAGATCATGACTGAGCAAGAATCTGGATACTGTTGGCA
CAATTTCGTAAACTACTCGCCATCGAATGAGAGCCATTGGCCTCGCTACCCCCATCTTTGGGTACGTTTGTATGTCTTGGAAC
TTTATTGTATCATTTTAGGGCTGCCACCTTGTCTTAATATTTTGCGCCGCAAACAGAGTCAGCTGACATCTTTCACAATTGCT
TTACAGTCATGCCACTACCAACGTCTTCCACCGCACATTCTGTGGGCCACGGGCTTAAAATCGGGCGGTAGCTCAGGCGGGAG
TTCAGGCTCAGAAACGCCGGGAACTAGCGAATCCGCAACGCCAGAGTCTTCCGGTGGTTCGTCAGGTGGTTCAGACAAGAAAT
ATAGCATCGGCCTGGCCATCGGCACAAATAGCGTCGGATGGGCGGTGATCACTGATGAATATAAAGTTCCGTCTAAAAAGTTC
AAGGTACTGGGTAATACAGATCGCCATAGTATCAAAAAGAACTTAATCGGTGCGCTTCTGTTCGATTCCGGCGAAACCGCAGA
AGCAACACGTCTGAAACGCACCGCTCGTCGCCGTTACACCCGTCGTAAAAACCGTATCTGCTACCTGCAAGAAATCTTCTCTA
ACGAAATGGCTAAAGTAGATGACAGCTTTTTTCACCGTCTGGAAGAATCATTTCTGGTGGAAGAAGATAAAAAGCACGAACGT
CATCCAATCTTCGGCAACATTGTGGACGAAGTAGCGTATCACGAAAAATACCCGACTATCTATCACCTGCGCAAAAAGCTGGT
CGATTCGACGGATAAGGCCGATCTGCGTCTGATCTATCTGGCCTTAGCGCATATGATTAAGTTCCGTGGTCATTTCCTGATCG
AAGGCGACCTGAATCCAGACAACAGCGATGTAGACAAACTGTTCATCCAGCTGGTGCAAACCTATAACCAGCTGTTTGAAGAA
AACCCAATTAATGCTAGCGGTGTTGACGCGAAAGCGATCTTGTCCGCACGCCTGTCCAAATCCCGTCGTCTGGAAAACTTAAT
TGCGCAACTGCCGGGTGAGAAGAAAAACGGACTGTTCGGCAATCTGATCGCTCTTAGCTTGGGACTGACCCCGAACTTCAAAA
GCAACTTCGATCTGGCAGAGGACGCAAAACTTCAACTTAGCAAAGATACGTATGACGATGACTTGGATAACTTACTGGCCCAG
ATCGGAGATCAGTACGCTGATCTGTTTCTGGCGGCAAAGAACTTATCAGACGCTATTCTCCTGTCTGATATTCTTCGTGTGAA
TACCGAAATCACCAAAGCACCGCTTTCTGCATCCATGATTAAACGCTATGACGAACATCACCAAGATCTGACTCTTCTGAAAG
CGCTGGTACGGCAACAACTGCCGGAGAAGTACAAGGAGATCTTCTTTGACCAATCCAAAAACGGCTACGCGGGTTATATTGAC
GGGGGTGCAAGCCAAGAGGAGTTCTACAAATTCATCAAGCCAATCTTAGAAAAAATGGATGGCACGGAAGAATTACTTGTTAA
ACTGAATCGTGAGGATCTGCTTCGTAAACAGCGTACCTTCGACAACGGTAGCATTCCGCACCAGATCCACTTAGGTGAACTGC
ACGCTATCCTGCGTCGCCAAGAGGATTTTTACCCGTTCCTGAAAGATAATCGTGAAAAAATCGAAAAAATCCTGACCTTTCGT
ATCCCGTATTATGTCGGCCCGCTGGCGCGTGGCAACTCCCGTTTCGCGTGGATGACTCGCAAATCCGAAGAAACTATTACCCC
GTGGAACTTCGAGGAAGTGGTTGACAAAGGCGCAAGCGCCCAATCCTTCATCGAGCGCATGACTAACTTTGATAAAAACCTGC
CGAACGAAAAGGTACTGCCGAAACACTCCCTTCTGTACGAATACTTCACCGTGTACAACGAGCTGACTAAAGTAAAGTATGTG
ACTGAGGGCATGCGTAAACCTGCATTCCTGAGCGGTGAACAGAAAAAAGCAATTGTTGATTTACTGTTTAAAACCAACCGTAA
AGTAACCGTTAAACAGCTGAAAGAGGACTACTTCAAGAAAATCGAATGCTTCGACTCCGTCGAGATTAGTGGAGTTGAAGATC
GTTTTAATGCAAGTTTAGGCACGTATCACGATTTATTAAAGATCATTAAAGACAAAGATTTCTTGGACAACGAAGAAAATGAG
GACATCTTAGAGGACATCGTCCTGACCCTGACTCTGTTCGAAGATCGTGAAATGATTGAAGAACGCCTTAAGACGTATGCTCA
CCTGTTTGACGATAAAGTAATGAAACAACTGAAACGTCGCCGTTATACTGGCTGGGGCCGTCTGAGCCGTAAACTGATTAACG
GTATCCGTGACAAACAGTCCGGTAAAACTATTCTGGACTTCCTGAAATCTGACGGCTTCGCAAACCGTAACTTCATGCAACTG
ATTCACGACGATTCCCTGACCTTCAAAGAGGACATCCAGAAAGCTCAGGTTTCTGGTCAAGGTGATTCTCTGCACGAGCATAT
CGCCAATTTAGCAGGTAGTCCGGCGATCAAAAAAGGTATCCTGCAAACCGTGAAAGTGGTGGATGAGCTTGTGAAAGTTATGG
GTCGTCACAAACCGGAAAACATTGTTATCGAGATGGCTCGTGAAAACCAAACGACCCAGAAGGGACAGAAAAACTCCCGCGAA
CGCATGAAACGTATCGAGGAGGGTATTAAAGAACTTGGCTCTCAGATTCTGAAAGAACACCCTGTTGAAAATACCCAACTGCA
AAATGAAAAACTGTACCTGTACTACCTGCAAAATGGTCGTGACATGTATGTAGATCAGGAGCTGGACATCAACCGCCTCTCCG
ATTACGACGTTGACCACATTGTTCCGCAGTCTTTTCTGAAAGATGATTCCATTGATAACAAAGTACTCACCCGTAGCGATAAA
AACCGTGGGAAGAGTGACAACGTTCCATCGGAAGAAGTAGTTAAGAAAATGAAGAACTATTGGCGTCAACTGCTTAACGCGAA
ACTGATTACTCAACGTAAATTTGATAACCTGACCAAAGCTGAACGTGGCGGTTTGTCTGAGCTGGATAAGGCGGGTTTTATTA
AACGTCAACTGGTAGAAACTCGCCAGATTACAAAACATGTTGCTCAGATTCTGGACTCTCGTATGAACACTAAATACGATGAA
AATGACAAACTGATCCGCGAAGTTAAGGTTATTACCCTGAAATCTAAGCTGGTTTCCGACTTCCGTAAAGATTTCCAATTCTA
TAAAGTGCGCGAGATTAACAACTATCACCACGCGCACGACGCATATCTGAATGCAGTTGTTGGCACGGCACTGATCAAAAAAT
ATCCGAAACTGGAAAGCGAATTTGTGTACGGCGATTATAAAGTTTACGACGTGCGCAAAATGATCGCCAAATCTGAACAGGAA
ATTGGCAAAGCAACCGCTAAATACTTTTTCTACTCAAACATTATGAATTTCTTCAAAACCGAAATCACCTTAGCGAATGGCGA

AATTCGTAAACGCCCTCTGATCGAAACCAACGGCGAAACGGGTGAGATCGTGTGGGACAAAGGTCGTGATTTCGCTACTGTCC
GCAAAGTTCTGTCCATGCCTCAAGTAAACATCGTTAAAAAGACTGAGGTACAGACTGGCGGTTTCAGCAAGGAATCCATTCTG
CCGAAACGCAACTCCGACAAACTGATCGCGCGTAAGAAAGACTGGGATCCGAAGAAATACGGTGGCTTCGATTCTCCAACCGT
GGCATACAGCGTTCTGGTAGTCGCCAAAGTCGAAAAGGGTAAATCAAAAAAACTGAAATCAGTGAAAGAACTTTTAGGCATCA
CCATTATGGAACGTAGCTCTTTCGAAAAAAACCCGATTGACTTCCTCGAAGCGAAGGGGTACAAGGAAGTAAAGAAAGATCTG
ATTATCAAACTGCCGAAGTATTCCCTGTTCGAACTGGAAAATGGTCGTAAACGTATGTTAGCGTCTGCGGGTGAACTGCAAAA
AGGGAACGAATTGGCCCTTCCGTCCAAGTACGTGAACTTCCTGTATCTGGCCTCGCACTACGAGAAACTGAAAGGTAGTCCGG
AAGATAATGAGCAGAAACAGCTGTTCGTGGAACAGCACAAACACTATCTGGACGAGATTATTGAACAGATTTCTGAGTTTAGC
AAACGCGTAATTCTGGCGGACGCGAATCTGGATAAAGTCCTGAGCGCCTACAATAAACACCGTGATAAACCGATCCGTGAACA
GGCAGAAAACATCATTCACCTGTTCACGCTGACTAATCTTGGTGCTCCGGCAGCCTTCAAATACTTCGACACCACGATCGATC
GTAAACGTTACACCTCCACTAAAGAAGTCTTAGATGCAACTCTTATTCACCAGAGCATCACTGGCCTGTATGAAACTCGTATT
GATCTGAGTCAGTTGGGCGGTGACAGTGGGGGCAGCGGAGGTAGCGGAGGCAGCACTAATTTGTCGGATATTATTGAGAAGGA
AACAGGAAAGCAATTGGTTATCCAGGAATCCATTTTAATGCTTCCGGAAGAAGTGGAAGAGGTGATCGGAAATAAGCCAGAGA
GCGATATCTTAGTTCATACGGCCTATGATGAAAGCACTGACGAGAACGTTATGCTGTTAACATCGGACGCTCCCGAGTACAAG
CCGTGGGCCCTGGTTATCCAGGACAGTAATGGCGAAAATAAAATTAAAATGTTATAATGTATGCTTAAGCAGCTCGGTACCAA
AGACGAACAATAAGACGCTGAAAAGCGTCTTTTTTCGTTTTGGTCCTGTTGCGGCGCGATAGTGTGAACATGCTATAGACTTC
TGGTGCTACCCGACTGACAATTAATCATCCGGCTCGTATAATGCTAGCGCGCGCAAAGCCAGCAGCTGGTTTTAGAGCTAGAA
ATAGCAAGTTAAAATAAGGCTAGTCCGTTATCAACTTGAAAAAGTGGCACCGAGTCGGTGCTTTTTTTAATCCATTCGAATCA
CGTCCGACGAACAATAAGGCCTCCCTAACGGGGGGCCTTTTTTATTGATAACAAAAGTAACTTCGAGCTTGTCTACCTCCTAG
CACCATTATTGCAATTAATAAACAACTAACGGACAATTCTACCTAACAGTTTTCATATATGACGAGCAGTTAAGTGATGAGTA
AAGGTGAGGAATTATTTACTGGTGTTGTTCCGATCTTAGTTGAACTGGACGGCGATGTTAACGGTCATAAATTCAGTGTTCGT
GGTGAAGGTGAAGGTGATGCAACCAACGGTAAGCTGACCCTGAAATTCATCTGCACTACTGGAAAATTACCAGTACCGTGGCC
TACTCTGGTGACTACCCTGACCTATGGTGTTCAGTGTTTTTCTCGTTACCCTGACCACATGAAGCAACATGATTTCTTCAAAT
CTGCAATGCCGGAAGGTTATGTACAGGAGCGCACCATTTCTTTCAAAGACGATGGCACGTATAAAACCCGTGCAGAGGTTAAA
TTTGAAGGTGACACTCTGGTGAATCGTATTGAACTGAAAGGCATTGATTTCAAAGAGGACGGCAATATTTTAGGCCACAAACT
GGAATATAACTTCAACTCCCATAACGTTTACATCACCGCAGACAAACAGAAGAACGGTATCAAAGCTAACTTCAAAATTCGCC
ATAACGTTGAAGATGGTAGCGTACAGCTGGCGGATCATTACCAACAGAACACTCCGATTGGAGATGCTCCTGTTTTACTGCCG
GATAACCACTACCTGTCCACCCAGTCTAAACTGTCGAAGGATCCGAACGAAAAGCGCGACCACATGGTGTTATTAGAGTTCGT
TACCGCTAGTGGTATCACGCACGGTATGGATGAACTCTACAAATAAGACGAACAATAAGGGGAGCGGGAAACCGCTCCCCTTT
TTTATTGATAACAAAAGTAAATTGCACGCTGATAGTCTCCCAATTGCGAAGGACCAAAACGAAAAAACACCCTTTCGGGTGTC
TTTTCTGGAATTTGGTACCGAGTACTAGGTATCGTGTAAGTAGCGAAGGCCCGTACGCGAGATAAACTGCTAGGCAACCGCGA
CTCTACGACTGGTGCTCGATTTAATTTCGCTGACGTAAAGAAATTATCGGCAGTGCGTCAACTGCCGTATCTTTATCTTAATT
AGGTAGTTGGACAAGCCCTTGAAAGAAATAGCAAGAGCCTGCCTCTCTATTGAAGTCACGGCGAAAGTCGGGTAGAAATCAAA
GAAAGCAGAAATTAAATCGGAGTAACACTAAGGTGGGATAACTCCGTAACTGACTACGCCTTTCTCTAGACTTTACTTGACCA
GATACACTGTCTTTGACACGTTGAAGGATTAGAGCAATCAAATCCAAGACTGGCTAAGCACGAAGCAACTCTTGAGTGTTAAA
AAGTTATCTCCTGTATTCGGGAAGCGGGTACTAGAAGATTGCAGGGACTCCGACGTTAAGTAAATTACAAAGTAATAAGTATC
GTTCAGGATCACGTTACCGCAATAAGAAGCGAGAATAATATAATTTCCGAAGTGCTTACCCCAGTAGTGACTATTCCTATAAC
CCTTCTGAGTGTCCGGAGGCGGAAATTTGCCACGAAAGAGAAAGTATTTCCCCGACAATAATAAAGGGGCGCTCCTCAGCTTT
TCCACTTGGTTGGGTAAGCTAGGCAACTCTGAAAGGAGTTTCGGCGAATTGAAGCCGACAGCTTTGAATTGTTTTAGGGGCGT
TATTCGAGGGCAATCGGAGCTAACTTCAAGACTACTTCTTTGTTGAATACTAAATAGTGCAAAGGTCGTGTTTCCTCAAGGAT
ACTCCGCTAACAATATAGGATTCCAATCAGATTCAGCACTGGCGGTACGGGTGTTGCGGTGAGGCGTTCGGGTTTACGGCTCG
AAGCTAGCACGGTAGG

p2779

CGCAGGATAAGTAAGGGGAGTAAGTGATCGAACGAATCAGAAGTGACAATATACTTAGGCTGGATCTCGTCCCGTGAATCCCA
ACCCTCACCAACTACGAGATAAGAGGTAAGCCAAAAATCGACTTGGTGGCGACCAACGACTGTTCCCCCCCTGTAACTAATCG
TTCCGTCAAAACCTGACTTACTTCAAGGCCAATTCCAAGCGCAAACAATACCGTCCTAGTTCTTCGGTTAAGTTTCCGAAGTA
GGAGTGAGCCTACCTCCGTTTGCGTCTTGTTACCACTGACCCAGCTATTTACTTTGTATTGCCTGCAATCGAATTTCTGAACT
CTCAGATAGTGGGGATAACGGGAAAGTTCCTATATTTGCGAACTAACTTAGCCGTCCACCTCGAAGCTACCTACTCACACCCA
CCCCGCGCGGGGTAAATAAGGCACTAATCCCAGCTGAGAGCTGGCGTAGCACTTAGCCACAAGTTAATTAACAGTTGTCTGGT
AGTTTGGCGGTATTAGGAAGATCCTAGAAGCAAGGCAGAGTTAGTTCTAACCTAAAGCCACAAATAAGACAGGTTGCCAAAGC
CCGCCGGAAATTAAATCTTGCTCAGTTCGGTAACGGAGTTTCCCTCCCGCGTACTTAATTCCCAATAAGAAACGCGCCCAAGT
CCTATCAGGCAAAATTCAGCCCCTTCCCGTGTTAGAACGAGGGTAAAAATACAAGCCGATTGAACAAGGGTTGGGGGCTTCAA
ATCGTCGTTTACCCCACTTTACAACGGAGATTAAGTAGTTCACCCTATAGTACGAAGCAGAACTATTTCGAGGGGCGTGCAAT
AATCGAATCTTCTGCGGTTGACTTAACACGCTAGGGACGTGCCCTCGATTCAATCGAAGGTACTCCTACTCAGACTGCCTCAC
ACCCAGCTAGTCACTGAGCGATAAAATTGACCCGCCCTCTAGGGAAGCGAGTACGTCCCAAAGGGCTCCGGACAGGGCTATAT
AGGAGAGTTTGATCTCGCCCCGACAACTGCAACCCTCAACTCCCTTAGATAATATTGTTAGCCGAAGTTGCACGACCCGCCGT
CCACGGACTGCTCTTAGGGTGTGGCTCCTTAATCTGACAACGTGCAACCCCTATCGAAGTCGATTGTTTCTGCGAAAGGTGTT
GTCCTAATAGTCCCGAAATTTGGCCCTTGTAGGTGTGAAACCACTTAGCTTCGCGCCGTAGTCCTAAAGGCCCACCTATTGAC

TTTGTTTCGGGTAGCACTAGGAATCTTAACAATTTGAATTTGGACGTGGAACGCGTACACCTTAATCTCCGAATAATTCTAGG
GATTTGGAAGTCCTCTACGTTGACACACCTACACTGCTCGAAGTAAATATACGAATAACGCGGGCCTCGCGGAGCCGTTCCGA
ATCGTCACGTGTTCGTTTACTGTTAATTGGTGGCAAATAAGCAATATCGTAGTCCGTCAGGCCCAGCCCTGTTATCCACGGCG
TTATTTGTCAAATTGCGTAGAACTGGATTGACTGCCTGACAATACCTAATTATCGGTACGAAGTCCCCGAATCTGTCGGGCTA
TTTCACTAATACTTTCCAAACGCCCCGTATCCAAGAAGAACGAATTTATCCACGCTCCCGTCTTTGGGACGAATACCGCTACA
AGTGGACAGAGGATCGGTACGGGCCTCTAATAAATCCAACACTCTACGCCCTCTTCAAGAGCTAGAAGAACAGGGTGCAGTTG
GAAAGGGAATTATTTCGTAAGGCGAGCCAATACCGTAATTAATTCGGAAGAGTTAACACGATTGGAAGTAGGAATAGTTTCTA
ACCACGGTTACTAATCCTAATAACGGAACGCTGTCTGATAGATTAGTGTCAGCGCTCGGTACCAAAGAAAAATAAAAAGACGC
TGAAAAGCGTCTTTTTATTTTTCGGTCCAGTGTAACTCAGGCAAAAGCACGTAATATTCGTACTTTCTTCCTCCGTAAGCGTC
ACCCACATTCCTTAAAGAGTGCATGTGCATATTTTGTTATCAATAAAAAAGGCCGCGATTTGCGGCCTTATTGTTCGTCTTGC
CGGATTACGCCCCGCCCTGCCACTCATCGCAGTATTGTTGTAATTCATTAAGCATTCTGCCGACATGGAAGCCATCACAAACG
GCATGATGAACTTGGATCGCCAGTGGCATTAACACCTTGTCGCCTTGCGTATAATATTTTCCCATAGTGAAAACGGGGGCGAA
GAAGTTGTCCATATTTGCTACGTTTAAATCAAAACTGGTGAAACTCACCCACGGATTGGCACTGACGAAAAACATATTTTCGA
TAAACCCTTTAGGGAAATATGCTAAGTTTTCACCGTAACACGCCACATCTTGACTATATATGTGTAGAAACTGCCGGAAATCG
TCGTGGTATTCTGACCAGAGCGATGAAAACGTTTCAGTTTGCTCATGGAAAACGGTGTAACAAGGGTGAACACTATCCCATAT
CACCAGCTCACCGTCTTTCATTGCCATACGAAACTCCGGATGTGCATTCATCAGGCGGGCAAGAATGTGAATAAAGGCCGGAT
AAAACTTGTGCTTATTTTTCTTTACGGTTTTTAAAAAGGCCGTAATATCCAGCTGAACGGTTTGGTTATAGGTGCACTGAGCA
ACTGACTGGAATGCCTCAAAATGTTCTTTACGATGCCATTGACTTATATCAACTGTAGTATATCCAGTGATTTTTTTCTCCAT
TTTAGCTTCCTTAGCTTGCGAAATCTCGATAACTCAAAAAATAGTAGTGATCTTATTTCATTATGGTGAAAGTTGTCTTACGT
GCAACATTTTCGCAAAAAGTTGGCGCTTTATCAACACTGTCGGAATGACAAATGGTTCCAATTATTGAACACCCTTCGGGGTG
TTTTTTTGTTTCTGGTTTCCCGAGGCCGGCCTTTTGTTGCAATGGCTGTCTACCCTGTCTACCTGAGTAAAGAAAAATACATT
TAATTCAGTATATTAACTTGGGTAGACAGCCTTTTTTTACTGTCTACCTTCTGTCTACCCTCTCTACCTGATTTTACCTGAAT
CAGACAGGGAGGTAGACACGGGGTAGACAGTGGATAAAAGCACTCTACCCCACTGAAAGCAGTGCCATTACTGGCATGGTTGC
CAGTAAGGTTGATAAGGTAGACAAGGGGAGGGACAACTCAAAACTTTTTAAACGAGGGGGTAAAACGCAGACCAAAACGATCT
CAAGAAGATCATCTTATTAATCAGATAAAATATTTCTAGATTTCAGTGCAATTTATCTCTTCAAATGTAGCACCGGCGCGCCG
TGACCAATTATTGAAGGCCGCTAACGCGGCCTTTTTTTGTTTCTGGTATCCCGAATGGAGCGACTTCTCCCCAAAAAGCCTCG
CTTTCAGCACCTGTCGTTTCCTTTCTTTTCAGAGGGTATTTTAAATAAAAACATTAAGTTATGACGAAGAAGAACGGAAACGC
CTTAAACCGGAAAATTTTCATAAATAGCGAAAACCCGCGAGGTCGCCGCCCCGTAACCTGTCGGATCACCGGAAAGGACCCGT
AAAGTGATAATGATTATCATCTACATATCACAACGTGCGTAAAGGGTAAGTATGAAGGTCGTGTACTCCATCGCTACCAAATT
CCAGAAAACAGACGCTTTCGAGCGTCTTTTTTCGTTTTGGTCACGACGTACGGTGGAAGATTCGTTACCAATTGACAGCTAGC
TCAGTCCTAGGTATATACATACATGCTTGTTTGTTTGTAAACCAGGATAAAGAGAGAGCATACTCGATGAAACGTACTGCCGA
CGGTTCAGAGTTCGAGTCGCCCAAGAAGAAACGTAAAGTGTCATCCAAAACCGGCCCAGTTGCGGTGGACCCAACGCTGCGCC
GTCGTATCGAACCGCACGAGTTCGAAGTATTTTTCGATCCTCGCGAACTGCGTAAAGAGACCTGTTTACTTTACGAAATCAAT
TGGGGTGGCCGCCACTCGATTTGGCGCCACACGTCTCAGAATACAAACAAACATGTCGAAGTAAATTTCATCGAAAAATTTAC
AACGGAGCGTTATTTCTGTCCCAATACTCGTTGTTCTATTACATGGTTTTTATCATGGAGTCCCTGCGGGGAATGCTCTCGTG
CGATCACCGAGTTTCTGTCGCGTTATCCGAACGTAACGCTTTTTATCTATATTGCTCGTCTTTACCACTTGGCTAACCCTCGT
AACCGCCAAGGACTTCGCGACCTGATTTCGAGTGGAGTAACCATCCAGATCATGACTGAGCAAGAATCTGGATACTGTTGGCA
CAATTTCGTAAACTACTCGCCATCGAATGAGAGCCATTGGCCTCGCTACCCCCATCTTTGGGTACGTTTGTATGTCTTGGAAC
TTTATTGTATCATTTTAGGGCTGCCACCTTGTCTTAATATTTTGCGCCGCAAACAGAGTCAGCTGACATCTTTCACAATTGCT
TTACAGTCATGCCACTACCAACGTCTTCCACCGCACATTCTGTGGGCCACGGGCTTAAAATCGGGCGGTAGCTCAGGCGGGAG
TTCAGGCTCAGAAACGCCGGGAACTAGCGAATCCGCAACGCCAGAGTCTTCCGGTGGTTCGTCAGGTGGTTCAGACAAGAAAT
ATAGCATCGGCCTGGCCATCGGCACAAATAGCGTCGGATGGGCGGTGATCACTGATGAATATAAAGTTCCGTCTAAAAAGTTC
AAGGTACTGGGTAATACAGATCGCCATAGTATCAAAAAGAACTTAATCGGTGCGCTTCTGTTCGATTCCGGCGAAACCGCAGA
AGCAACACGTCTGAAACGCACCGCTCGTCGCCGTTACACCCGTCGTAAAAACCGTATCTGCTACCTGCAAGAAATCTTCTCTA
ACGAAATGGCTAAAGTAGATGACAGCTTTTTTCACCGTCTGGAAGAATCATTTCTGGTGGAAGAAGATAAAAAGCACGAACGT
CATCCAATCTTCGGCAACATTGTGGACGAAGTAGCGTATCACGAAAAATACCCGACTATCTATCACCTGCGCAAAAAGCTGGT
CGATTCGACGGATAAGGCCGATCTGCGTCTGATCTATCTGGCCTTAGCGCATATGATTAAGTTCCGTGGTCATTTCCTGATCG
AAGGCGACCTGAATCCAGACAACAGCGATGTAGACAAACTGTTCATCCAGCTGGTGCAAACCTATAACCAGCTGTTTGAAGAA
AACCCAATTAATGCTAGCGGTGTTGACGCGAAAGCGATCTTGTCCGCACGCCTGTCCAAATCCCGTCGTCTGGAAAACTTAAT
TGCGCAACTGCCGGGTGAGAAGAAAAACGGACTGTTCGGCAATCTGATCGCTCTTAGCTTGGGACTGACCCCGAACTTCAAAA
GCAACTTCGATCTGGCAGAGGACGCAAAACTTCAACTTAGCAAAGATACGTATGACGATGACTTGGATAACTTACTGGCCCAG
ATCGGAGATCAGTACGCTGATCTGTTTCTGGCGGCAAAGAACTTATCAGACGCTATTCTCCTGTCTGATATTCTTCGTGTGAA
TACCGAAATCACCAAAGCACCGCTTTCTGCATCCATGATTAAACGCTATGACGAACATCACCAAGATCTGACTCTTCTGAAAG
CGCTGGTACGGCAACAACTGCCGGAGAAGTACAAGGAGATCTTCTTTGACCAATCCAAAAACGGCTACGCGGGTTATATTGAC
GGGGGTGCAAGCCAAGAGGAGTTCTACAAATTCATCAAGCCAATCTTAGAAAAAATGGATGGCACGGAAGAATTACTTGTTAA
ACTGAATCGTGAGGATCTGCTTCGTAAACAGCGTACCTTCGACAACGGTAGCATTCCGCACCAGATCCACTTAGGTGAACTGC
ACGCTATCCTGCGTCGCCAAGAGGATTTTTACCCGTTCCTGAAAGATAATCGTGAAAAAATCGAAAAAATCCTGACCTTTCGT
ATCCCGTATTATGTCGGCCCGCTGGCGCGTGGCAACTCCCGTTTCGCGTGGATGACTCGCAAATCCGAAGAAACTATTACCCC
GTGGAACTTCGAGGAAGTGGTTGACAAAGGCGCAAGCGCCCAATCCTTCATCGAGCGCATGACTAACTTTGATAAAAACCTGC
CGAACGAAAAGGTACTGCCGAAACACTCCCTTCTGTACGAATACTTCACCGTGTACAACGAGCTGACTAAAGTAAAGTATGTG
ACTGAGGGCATGCGTAAACCTGCATTCCTGAGCGGTGAACAGAAAAAAGCAATTGTTGATTTACTGTTTAAAACCAACCGTAA
AGTAACCGTTAAACAGCTGAAAGAGGACTACTTCAAGAAAATCGAATGCTTCGACTCCGTCGAGATTAGTGGAGTTGAAGATC
GTTTTAATGCAAGTTTAGGCACGTATCACGATTTATTAAAGATCATTAAAGACAAAGATTTCTTGGACAACGAAGAAAATGAG

GACATCTTAGAGGACATCGTCCTGACCCTGACTCTGTTCGAAGATCGTGAAATGATTGAAGAACGCCTTAAGACGTATGCTCA
CCTGTTTGACGATAAAGTAATGAAACAACTGAAACGTCGCCGTTATACTGGCTGGGGCCGTCTGAGCCGTAAACTGATTAACG
GTATCCGTGACAAACAGTCCGGTAAAACTATTCTGGACTTCCTGAAATCTGACGGCTTCGCAAACCGTAACTTCATGCAACTG
ATTCACGACGATTCCCTGACCTTCAAAGAGGACATCCAGAAAGCTCAGGTTTCTGGTCAAGGTGATTCTCTGCACGAGCATAT
CGCCAATTTAGCAGGTAGTCCGGCGATCAAAAAAGGTATCCTGCAAACCGTGAAAGTGGTGGATGAGCTTGTGAAAGTTATGG
GTCGTCACAAACCGGAAAACATTGTTATCGAGATGGCTCGTGAAAACCAAACGACCCAGAAGGGACAGAAAAACTCCCGCGAA
CGCATGAAACGTATCGAGGAGGGTATTAAAGAACTTGGCTCTCAGATTCTGAAAGAACACCCTGTTGAAAATACCCAACTGCA
AAATGAAAAACTGTACCTGTACTACCTGCAAAATGGTCGTGACATGTATGTAGATCAGGAGCTGGACATCAACCGCCTCTCCG
ATTACGACGTTGACCACATTGTTCCGCAGTCTTTTCTGAAAGATGATTCCATTGATAACAAAGTACTCACCCGTAGCGATAAA
AACCGTGGGAAGAGTGACAACGTTCCATCGGAAGAAGTAGTTAAGAAAATGAAGAACTATTGGCGTCAACTGCTTAACGCGAA
ACTGATTACTCAACGTAAATTTGATAACCTGACCAAAGCTGAACGTGGCGGTTTGTCTGAGCTGGATAAGGCGGGTTTTATTA
AACGTCAACTGGTAGAAACTCGCCAGATTACAAAACATGTTGCTCAGATTCTGGACTCTCGTATGAACACTAAATACGATGAA
AATGACAAACTGATCCGCGAAGTTAAGGTTATTACCCTGAAATCTAAGCTGGTTTCCGACTTCCGTAAAGATTTCCAATTCTA
TAAAGTGCGCGAGATTAACAACTATCACCACGCGCACGACGCATATCTGAATGCAGTTGTTGGCACGGCACTGATCAAAAAAT
ATCCGAAACTGGAAAGCGAATTTGTGTACGGCGATTATAAAGTTTACGACGTGCGCAAAATGATCGCCAAATCTGAACAGGAA
ATTGGCAAAGCAACCGCTAAATACTTTTTCTACTCAAACATTATGAATTTCTTCAAAACCGAAATCACCTTAGCGAATGGCGA
AATTCGTAAACGCCCTCTGATCGAAACCAACGGCGAAACGGGTGAGATCGTGTGGGACAAAGGTCGTGATTTCGCTACTGTCC
GCAAAGTTCTGTCCATGCCTCAAGTAAACATCGTTAAAAAGACTGAGGTACAGACTGGCGGTTTCAGCAAGGAATCCATTCTG
CCGAAACGCAACTCCGACAAACTGATCGCGCGTAAGAAAGACTGGGATCCGAAGAAATACGGTGGCTTCGATTCTCCAACCGT
GGCATACAGCGTTCTGGTAGTCGCCAAAGTCGAAAAGGGTAAATCAAAAAAACTGAAATCAGTGAAAGAACTTTTAGGCATCA
CCATTATGGAACGTAGCTCTTTCGAAAAAAACCCGATTGACTTCCTCGAAGCGAAGGGGTACAAGGAAGTAAAGAAAGATCTG
ATTATCAAACTGCCGAAGTATTCCCTGTTCGAACTGGAAAATGGTCGTAAACGTATGTTAGCGTCTGCGGGTGAACTGCAAAA
AGGGAACGAATTGGCCCTTCCGTCCAAGTACGTGAACTTCCTGTATCTGGCCTCGCACTACGAGAAACTGAAAGGTAGTCCGG
AAGATAATGAGCAGAAACAGCTGTTCGTGGAACAGCACAAACACTATCTGGACGAGATTATTGAACAGATTTCTGAGTTTAGC
AAACGCGTAATTCTGGCGGACGCGAATCTGGATAAAGTCCTGAGCGCCTACAATAAACACCGTGATAAACCGATCCGTGAACA
GGCAGAAAACATCATTCACCTGTTCACGCTGACTAATCTTGGTGCTCCGGCAGCCTTCAAATACTTCGACACCACGATCGATC
GTAAACGTTACACCTCCACTAAAGAAGTCTTAGATGCAACTCTTATTCACCAGAGCATCACTGGCCTGTATGAAACTCGTATT
GATCTGAGTCAGTTGGGCGGTGACAGTGGGGGCAGCGGAGGTAGCGGAGGCAGCACTAATTTGTCGGATATTATTGAGAAGGA
AACAGGAAAGCAATTGGTTATCCAGGAATCCATTTTAATGCTTCCGGAAGAAGTGGAAGAGGTGATCGGAAATAAGCCAGAGA
GCGATATCTTAGTTCATACGGCCTATGATGAAAGCACTGACGAGAACGTTATGCTGTTAACATCGGACGCTCCCGAGTACAAG
CCGTGGGCCCTGGTTATCCAGGACAGTAATGGCGAAAATAAAATTAAAATGTTATAATGTATGCTTAAGCAGCTCGGTACCAA
AGACGAACAATAAGACGCTGAAAAGCGTCTTTTTTCGTTTTGGTCCTGTTGCGGCGCGATAGTGTGAACATGCTATAGACTTC
TGGTGCTACCCGACTGACAATTAATCATCCGGCTCGTATAATGCTAGCGCGCGCAAAGCCAGCAGCTGGTTTTAGAGCTAGAA
ATAGCAAGTTAAAATAAGGCTAGTCCGTTATCAACTTGAAAAAGTGGCACCGAGTCGGTGCTTTTTTTAATCCATTCGAATCA
CGTCCGACGAACAATAAGGCCTCCCTAACGGGGGGCCTTTTTTATTGATAACAAAAGTAACTTCGAGCTTGTCTACCTCCTAG
CACCATTATTGCAATTAATAAACAACTAACGGACAATTCTACCTAACAGTTTTCATATATGACGAGCAGTTAAGTGATGAGTA
AAGGTGAGGAATTATTTACTGGTGTTGTTCCGATCTTAGTTGAACTGGACGGCGATGTTAACGGTCATAAATTCAGTGTTCGT
GGTGAAGGTGAAGGTGATGCAACCAACGGTAAGCTGACCCTGAAATTCATCTGCACTACTGGAAAATTACCAGTACCGTGGCC
TACTCTGGTGACTACCCTGACCTATGGTGTTCAGTGTTTTTCTCGTTACCCTGACCACATGAAGCAACATGATTTCTTCAAAT
CTGCAATGCCGGAAGGTTATGTACAGGAGCGCACCATTTCTTTCAAAGACGATGGCACGTATAAAACCCGTGCAGAGGTTAAA
TTTGAAGGTGACACTCTGGTGAATCGTATTGAACTGAAAGGCATTGATTTCAAAGAGGACGGCAATATTTTAGGCCACAAACT
GGAATATAACTTCAACTCCCATAACGTTTACATCACCGCAGACAAACAGAAGAACGGTATCAAAGCTAACTTCAAAATTCGCC
ATAACGTTGAAGATGGTAGCGTACAGCTGGCGGATCATTACCAACAGAACACTCCGATTGGAGATGCTCCTGTTTTACTGCCG
GATAACCACTACCTGTCCACCCAGTCTAAACTGTCGAAGGATCCGAACGAAAAGCGCGACCACATGGTGTTATTAGAGTTCGT
TACCGCTAGTGGTATCACGCACGGTATGGATGAACTCTACAAATAAGACGAACAATAAGGGGAGCGGGAAACCGCTCCCCTTT
TTTATTGATAACAAAAGTAAATTGCACGCTGATAGTCTCCCAATTGCGAAGGACCAAAACGAAAAAACACCCTTTCGGGTGTC
TTTTCTGGAATTTGGTACCGAGTACTAGGTATCGTGTAAGTAGCGAAGGCCCGTACGCGAGATAAACTGCTAGGCAACCGCGA
CTCTACGACTGGTGCTCGATTTAATTTCGCTGACGTAAAGAAATTATCGGCAGTGCGTCAACTGCCGTATCTTTATCTTAATT
AGGTAGTTGGACAAGCCCTTGAAAGAAATAGCAAGAGCCTGCCTCTCTATTGAAGTCACGGCGAAAGTCGGGTAGAAATCAAA
GAAAGCAGAAATTAAATCGGAGTAACACTAAGGTGGGATAACTCCGTAACTGACTACGCCTTTCTCTAGACTTTACTTGACCA
GATACACTGTCTTTGACACGTTGAAGGATTAGAGCAATCAAATCCAAGACTGGCTAAGCACGAAGCAACTCTTGAGTGTTAAA
AAGTTATCTCCTGTATTCGGGAAGCGGGTACTAGAAGATTGCAGGGACTCCGACGTTAAGTAAATTACAAAGTAATAAGTATC
GTTCAGGATCACGTTACCGCAATAAGAAGCGAGAATAATATAATTTCCGAAGTGCTTACCCCAGTAGTGACTATTCCTATAAC
CCTTCTGAGTGTCCGGAGGCGGAAATTTGCCACGAAAGAGAAAGTATTTCCCCGACAATAATAAAGGGGCGCTCCTCAGCTTT
TCCACTTGGTTGGGTAAGCTAGGCAACTCTGAAAGGAGTTTCGGCGAATTGAAGCCGACAGCTTTGAATTGTTTTAGGGGCGT
TATTCGAGGGCAATCGGAGCTAACTTCAAGACTACTTCTTTGTTGAATACTAAATAGTGCAAAGGTCGTGTTTCCTCAAGGAT
ACTCCGCTAACAATATAGGATTCCAATCAGATTCAGCACTGGCGGTACGGGTGTTGCGGTGAGGCGTTCGGGTTTACGGCTCG
AAGCTAGCACGGTAGG

p2781

CGCAGGATAAGTAAGGGGAGTAAGTGATCGAACGAATCAGAAGTGACAATATACTTAGGCTGGATCTCGTCCCGTGAATCCCA
ACCCTCACCAACTACGAGATAAGAGGTAAGCCAAAAATCGACTTGGTGGCGACCAACGACTGTTCCCCCCCTGTAACTAATCG
TTCCGTCAAAACCTGACTTACTTCAAGGCCAATTCCAAGCGCAAACAATACCGTCCTAGTTCTTCGGTTAAGTTTCCGAAGTA
GGAGTGAGCCTACCTCCGTTTGCGTCTTGTTACCACTGACCCAGCTATTTACTTTGTATTGCCTGCAATCGAATTTCTGAACT
CTCAGATAGTGGGGATAACGGGAAAGTTCCTATATTTGCGAACTAACTTAGCCGTCCACCTCGAAGCTACCTACTCACACCCA
CCCCGCGCGGGGTAAATAAGGCACTAATCCCAGCTGAGAGCTGGCGTAGCACTTAGCCACAAGTTAATTAACAGTTGTCTGGT
AGTTTGGCGGTATTAGGAAGATCCTAGAAGCAAGGCAGAGTTAGTTCTAACCTAAAGCCACAAATAAGACAGGTTGCCAAAGC
CCGCCGGAAATTAAATCTTGCTCAGTTCGGTAACGGAGTTTCCCTCCCGCGTACTTAATTCCCAATAAGAAACGCGCCCAAGT
CCTATCAGGCAAAATTCAGCCCCTTCCCGTGTTAGAACGAGGGTAAAAATACAAGCCGATTGAACAAGGGTTGGGGGCTTCAA
ATCGTCGTTTACCCCACTTTACAACGGAGATTAAGTAGTTCACCCTATAGTACGAAGCAGAACTATTTCGAGGGGCGTGCAAT
AATCGAATCTTCTGCGGTTGACTTAACACGCTAGGGACGTGCCCTCGATTCAATCGAAGGTACTCCTACTCAGACTGCCTCAC
ACCCAGCTAGTCACTGAGCGATAAAATTGACCCGCCCTCTAGGGAAGCGAGTACGTCCCAAAGGGCTCCGGACAGGGCTATAT
AGGAGAGTTTGATCTCGCCCCGACAACTGCAACCCTCAACTCCCTTAGATAATATTGTTAGCCGAAGTTGCACGACCCGCCGT
CCACGGACTGCTCTTAGGGTGTGGCTCCTTAATCTGACAACGTGCAACCCCTATCGAAGTCGATTGTTTCTGCGAAAGGTGTT
GTCCTAATAGTCCCGAAATTTGGCCCTTGTAGGTGTGAAACCACTTAGCTTCGCGCCGTAGTCCTAAAGGCCCACCTATTGAC
TTTGTTTCGGGTAGCACTAGGAATCTTAACAATTTGAATTTGGACGTGGAACGCGTACACCTTAATCTCCGAATAATTCTAGG
GATTTGGAAGTCCTCTACGTTGACACACCTACACTGCTCGAAGTAAATATACGAATAACGCGGGCCTCGCGGAGCCGTTCCGA
ATCGTCACGTGTTCGTTTACTGTTAATTGGTGGCAAATAAGCAATATCGTAGTCCGTCAGGCCCAGCCCTGTTATCCACGGCG
TTATTTGTCAAATTGCGTAGAACTGGATTGACTGCCTGACAATACCTAATTATCGGTACGAAGTCCCCGAATCTGTCGGGCTA
TTTCACTAATACTTTCCAAACGCCCCGTATCCAAGAAGAACGAATTTATCCACGCTCCCGTCTTTGGGACGAATACCGCTACA
AGTGGACAGAGGATCGGTACGGGCCTCTAATAAATCCAACACTCTACGCCCTCTTCAAGAGCTAGAAGAACAGGGTGCAGTTG
GAAAGGGAATTATTTCGTAAGGCGAGCCAATACCGTAATTAATTCGGAAGAGTTAACACGATTGGAAGTAGGAATAGTTTCTA
ACCACGGTTACTAATCCTAATAACGGAACGCTGTCTGATAGATTAGTGTCAGCGCTCGGTACCAAAGAAAAATAAAAAGACGC
TGAAAAGCGTCTTTTTATTTTTCGGTCCAGTGTAACTCAGGCAAAAGCACGTAATATTCGTACTTTCTTCCTCCGTAAGCGTC
ACCCACATTCCTTAAAGAGTGCATGTGCATATTTTGTTATCAATAAAAAAGGCCGCGATTTGCGGCCTTATTGTTCGTCTTGC
CGGATTACGCCCCGCCCTGCCACTCATCGCAGTATTGTTGTAATTCATTAAGCATTCTGCCGACATGGAAGCCATCACAAACG
GCATGATGAACTTGGATCGCCAGTGGCATTAACACCTTGTCGCCTTGCGTATAATATTTTCCCATAGTGAAAACGGGGGCGAA
GAAGTTGTCCATATTTGCTACGTTTAAATCAAAACTGGTGAAACTCACCCACGGATTGGCACTGACGAAAAACATATTTTCGA
TAAACCCTTTAGGGAAATATGCTAAGTTTTCACCGTAACACGCCACATCTTGACTATATATGTGTAGAAACTGCCGGAAATCG
TCGTGGTATTCTGACCAGAGCGATGAAAACGTTTCAGTTTGCTCATGGAAAACGGTGTAACAAGGGTGAACACTATCCCATAT
CACCAGCTCACCGTCTTTCATTGCCATACGAAACTCCGGATGTGCATTCATCAGGCGGGCAAGAATGTGAATAAAGGCCGGAT
AAAACTTGTGCTTATTTTTCTTTACGGTTTTTAAAAAGGCCGTAATATCCAGCTGAACGGTTTGGTTATAGGTGCACTGAGCA
ACTGACTGGAATGCCTCAAAATGTTCTTTACGATGCCATTGACTTATATCAACTGTAGTATATCCAGTGATTTTTTTCTCCAT
TTTAGCTTCCTTAGCTTGCGAAATCTCGATAACTCAAAAAATAGTAGTGATCTTATTTCATTATGGTGAAAGTTGTCTTACGT
GCAACATTTTCGCAAAAAGTTGGCGCTTTATCAACACTGTCGGAATGACAAATGGTTCCAATTATTGAACACCCTTCGGGGTG
TTTTTTTGTTTCTGGTTTCCCGAGGCCGGCCTTTTGTTGCAATGGCTGTCTACCCTGTCTACCTGAGTAAAGAAAAATACATT
TAATTCAGTATATTAACTTGGGTAGACAGCCTTTTTTTACTGTCTACCTTCTGTCTACCCTCTCTACCTGATTTTACCTGAAT
CAGACAGGGAGGTAGACACGGGGTAGACAGTGGATAAAAGCACTCTACCCCACTGAAAGCAGTGCCATTACTGGCATGGTTGC
CAGTAAGGTTGATAAGGTAGACAAGGGGAGGGACAACTCAAAACTTTTTAAACGAGGGGGTAAAACGCAGACCAAAACGATCT
CAAGAAGATCATCTTATTAATCAGATAAAATATTTCTAGATTTCAGTGCAATTTATCTCTTCAAATGTAGCACCGGCGCGCCG
TGACCAATTATTGAAGGCCGCTAACGCGGCCTTTTTTTGTTTCTGGTATCCCGAATGGAGCGACTTCTCCCCAAAAAGCCTCG
CTTTCAGCACCTGTCGTTTCCTTTCTTTTCAGAGGGTATTTTAAATAAAAACATTAAGTTATGACGAAGAAGAACGGAAACGC
CTTAAACCGGAAAATTTTCATAAATAGCGAAAACCCGCGAGGTCGCCGCCCCGTAACCTGTCGGATCACCGGAAAGGACCCGT
AAAGTGATAATGATTATCATCTACATATCACAACGTGCGTAAAGGGTAAGTATGAAGGTCGTGTACTCCATCGCTACCAAATT
CCAGAAAACAGACGCTTTCGAGCGTCTTTTTTCGTTTTGGTCACGACGTACGGTGGAAGATTCGTTACCAATTGACAGCTAGC
TCAGTCCTAGGTATATACATACATGCTTGTTTGTTTGTAAACCAGGATAAAGAGAGAGCATACTCGATGAAACGTACTGCCGA
CGGTTCAGAGTTCGAGTCGCCCAAGAAGAAACGTAAAGTGTCATCCAAAACCGGCCCAGTTGCGGTGGACCCAACGCTGCGCC
GTCGTATCGAACCGCACGAGTTCGAAGTATTTTTCGATCCTCGCGAACTGCGTAAAGAGACCTGTTTACTTTACGAAATCAAT
TGGGGTGGCCGCCACTCGATTTGGCGCCACACGTCTCAGAATACAAACAAACATGTCGAAGTAAATTTCATCGAAAAATTTAC
AACGGAGCGTTATTTCTGTCCCAATACTCGTTGTTCTATTACATGGTTTTTATCATGGAGTCCCTGCGGGGAATGCTCTCGTG
CGATCACCGAGTTTCTGTCGCGTTATCCGAACGTAACGCTTTTTATCTATATTGCTCGTCTTTACCACTTGGCTAACCCTCGT
AACCGCCAAGGACTTCGCGACCTGATTTCGAGTGGAGTAACCATCCAGATCATGACTGAGCAAGAATCTGGATACTGTTGGCA
CAATTTCGTAAACTACTCGCCATCGAATGAGAGCCATTGGCCTCGCTACCCCCATCTTTGGGTACGTTTGTATGTCTTGGAAC
TTTATTGTATCATTTTAGGGCTGCCACCTTGTCTTAATATTTTGCGCCGCAAACAGAGTCAGCTGACATCTTTCACAATTGCT
TTACAGTCATGCCACTACCAACGTCTTCCACCGCACATTCTGTGGGCCACGGGCTTAAAATCGGGCGGTAGCTCAGGCGGGAG
TTCAGGCTCAGAAACGCCGGGAACTAGCGAATCCGCAACGCCAGAGTCTTCCGGTGGTTCGTCAGGTGGTTCAGACAAGAAAT
ATAGCATCGGCCTGGCCATCGGCACAAATAGCGTCGGATGGGCGGTGATCACTGATGAATATAAAGTTCCGTCTAAAAAGTTC
AAGGTACTGGGTAATACAGATCGCCATAGTATCAAAAAGAACTTAATCGGTGCGCTTCTGTTCGATTCCGGCGAAACCGCAGA
AGCAACACGTCTGAAACGCACCGCTCGTCGCCGTTACACCCGTCGTAAAAACCGTATCTGCTACCTGCAAGAAATCTTCTCTA
ACGAAATGGCTAAAGTAGATGACAGCTTTTTTCACCGTCTGGAAGAATCATTTCTGGTGGAAGAAGATAAAAAGCACGAACGT
CATCCAATCTTCGGCAACATTGTGGACGAAGTAGCGTATCACGAAAAATACCCGACTATCTATCACCTGCGCAAAAAGCTGGT

CGATTCGACGGATAAGGCCGATCTGCGTCTGATCTATCTGGCCTTAGCGCATATGATTAAGTTCCGTGGTCATTTCCTGATCG
AAGGCGACCTGAATCCAGACAACAGCGATGTAGACAAACTGTTCATCCAGCTGGTGCAAACCTATAACCAGCTGTTTGAAGAA
AACCCAATTAATGCTAGCGGTGTTGACGCGAAAGCGATCTTGTCCGCACGCCTGTCCAAATCCCGTCGTCTGGAAAACTTAAT
TGCGCAACTGCCGGGTGAGAAGAAAAACGGACTGTTCGGCAATCTGATCGCTCTTAGCTTGGGACTGACCCCGAACTTCAAAA
GCAACTTCGATCTGGCAGAGGACGCAAAACTTCAACTTAGCAAAGATACGTATGACGATGACTTGGATAACTTACTGGCCCAG
ATCGGAGATCAGTACGCTGATCTGTTTCTGGCGGCAAAGAACTTATCAGACGCTATTCTCCTGTCTGATATTCTTCGTGTGAA
TACCGAAATCACCAAAGCACCGCTTTCTGCATCCATGATTAAACGCTATGACGAACATCACCAAGATCTGACTCTTCTGAAAG
CGCTGGTACGGCAACAACTGCCGGAGAAGTACAAGGAGATCTTCTTTGACCAATCCAAAAACGGCTACGCGGGTTATATTGAC
GGGGGTGCAAGCCAAGAGGAGTTCTACAAATTCATCAAGCCAATCTTAGAAAAAATGGATGGCACGGAAGAATTACTTGTTAA
ACTGAATCGTGAGGATCTGCTTCGTAAACAGCGTACCTTCGACAACGGTAGCATTCCGCACCAGATCCACTTAGGTGAACTGC
ACGCTATCCTGCGTCGCCAAGAGGATTTTTACCCGTTCCTGAAAGATAATCGTGAAAAAATCGAAAAAATCCTGACCTTTCGT
ATCCCGTATTATGTCGGCCCGCTGGCGCGTGGCAACTCCCGTTTCGCGTGGATGACTCGCAAATCCGAAGAAACTATTACCCC
GTGGAACTTCGAGGAAGTGGTTGACAAAGGCGCAAGCGCCCAATCCTTCATCGAGCGCATGACTAACTTTGATAAAAACCTGC
CGAACGAAAAGGTACTGCCGAAACACTCCCTTCTGTACGAATACTTCACCGTGTACAACGAGCTGACTAAAGTAAAGTATGTG
ACTGAGGGCATGCGTAAACCTGCATTCCTGAGCGGTGAACAGAAAAAAGCAATTGTTGATTTACTGTTTAAAACCAACCGTAA
AGTAACCGTTAAACAGCTGAAAGAGGACTACTTCAAGAAAATCGAATGCTTCGACTCCGTCGAGATTAGTGGAGTTGAAGATC
GTTTTAATGCAAGTTTAGGCACGTATCACGATTTATTAAAGATCATTAAAGACAAAGATTTCTTGGACAACGAAGAAAATGAG
GACATCTTAGAGGACATCGTCCTGACCCTGACTCTGTTCGAAGATCGTGAAATGATTGAAGAACGCCTTAAGACGTATGCTCA
CCTGTTTGACGATAAAGTAATGAAACAACTGAAACGTCGCCGTTATACTGGCTGGGGCCGTCTGAGCCGTAAACTGATTAACG
GTATCCGTGACAAACAGTCCGGTAAAACTATTCTGGACTTCCTGAAATCTGACGGCTTCGCAAACCGTAACTTCATGCAACTG
ATTCACGACGATTCCCTGACCTTCAAAGAGGACATCCAGAAAGCTCAGGTTTCTGGTCAAGGTGATTCTCTGCACGAGCATAT
CGCCAATTTAGCAGGTAGTCCGGCGATCAAAAAAGGTATCCTGCAAACCGTGAAAGTGGTGGATGAGCTTGTGAAAGTTATGG
GTCGTCACAAACCGGAAAACATTGTTATCGAGATGGCTCGTGAAAACCAAACGACCCAGAAGGGACAGAAAAACTCCCGCGAA
CGCATGAAACGTATCGAGGAGGGTATTAAAGAACTTGGCTCTCAGATTCTGAAAGAACACCCTGTTGAAAATACCCAACTGCA
AAATGAAAAACTGTACCTGTACTACCTGCAAAATGGTCGTGACATGTATGTAGATCAGGAGCTGGACATCAACCGCCTCTCCG
ATTACGACGTTGACCACATTGTTCCGCAGTCTTTTCTGAAAGATGATTCCATTGATAACAAAGTACTCACCCGTAGCGATAAA
AACCGTGGGAAGAGTGACAACGTTCCATCGGAAGAAGTAGTTAAGAAAATGAAGAACTATTGGCGTCAACTGCTTAACGCGAA
ACTGATTACTCAACGTAAATTTGATAACCTGACCAAAGCTGAACGTGGCGGTTTGTCTGAGCTGGATAAGGCGGGTTTTATTA
AACGTCAACTGGTAGAAACTCGCCAGATTACAAAACATGTTGCTCAGATTCTGGACTCTCGTATGAACACTAAATACGATGAA
AATGACAAACTGATCCGCGAAGTTAAGGTTATTACCCTGAAATCTAAGCTGGTTTCCGACTTCCGTAAAGATTTCCAATTCTA
TAAAGTGCGCGAGATTAACAACTATCACCACGCGCACGACGCATATCTGAATGCAGTTGTTGGCACGGCACTGATCAAAAAAT
ATCCGAAACTGGAAAGCGAATTTGTGTACGGCGATTATAAAGTTTACGACGTGCGCAAAATGATCGCCAAATCTGAACAGGAA
ATTGGCAAAGCAACCGCTAAATACTTTTTCTACTCAAACATTATGAATTTCTTCAAAACCGAAATCACCTTAGCGAATGGCGA
AATTCGTAAACGCCCTCTGATCGAAACCAACGGCGAAACGGGTGAGATCGTGTGGGACAAAGGTCGTGATTTCGCTACTGTCC
GCAAAGTTCTGTCCATGCCTCAAGTAAACATCGTTAAAAAGACTGAGGTACAGACTGGCGGTTTCAGCAAGGAATCCATTCTG
CCGAAACGCAACTCCGACAAACTGATCGCGCGTAAGAAAGACTGGGATCCGAAGAAATACGGTGGCTTCGATTCTCCAACCGT
GGCATACAGCGTTCTGGTAGTCGCCAAAGTCGAAAAGGGTAAATCAAAAAAACTGAAATCAGTGAAAGAACTTTTAGGCATCA
CCATTATGGAACGTAGCTCTTTCGAAAAAAACCCGATTGACTTCCTCGAAGCGAAGGGGTACAAGGAAGTAAAGAAAGATCTG
ATTATCAAACTGCCGAAGTATTCCCTGTTCGAACTGGAAAATGGTCGTAAACGTATGTTAGCGTCTGCGGGTGAACTGCAAAA
AGGGAACGAATTGGCCCTTCCGTCCAAGTACGTGAACTTCCTGTATCTGGCCTCGCACTACGAGAAACTGAAAGGTAGTCCGG
AAGATAATGAGCAGAAACAGCTGTTCGTGGAACAGCACAAACACTATCTGGACGAGATTATTGAACAGATTTCTGAGTTTAGC
AAACGCGTAATTCTGGCGGACGCGAATCTGGATAAAGTCCTGAGCGCCTACAATAAACACCGTGATAAACCGATCCGTGAACA
GGCAGAAAACATCATTCACCTGTTCACGCTGACTAATCTTGGTGCTCCGGCAGCCTTCAAATACTTCGACACCACGATCGATC
GTAAACGTTACACCTCCACTAAAGAAGTCTTAGATGCAACTCTTATTCACCAGAGCATCACTGGCCTGTATGAAACTCGTATT
GATCTGAGTCAGTTGGGCGGTGACAGTGGGGGCAGCGGAGGTAGCGGAGGCAGCACTAATTTGTCGGATATTATTGAGAAGGA
AACAGGAAAGCAATTGGTTATCCAGGAATCCATTTTAATGCTTCCGGAAGAAGTGGAAGAGGTGATCGGAAATAAGCCAGAGA
GCGATATCTTAGTTCATACGGCCTATGATGAAAGCACTGACGAGAACGTTATGCTGTTAACATCGGACGCTCCCGAGTACAAG
CCGTGGGCCCTGGTTATCCAGGACAGTAATGGCGAAAATAAAATTAAAATGTTATAATGTATGCTTAAGCAGCTCGGTACCAA
AGACGAACAATAAGACGCTGAAAAGCGTCTTTTTTCGTTTTGGTCCTGTTGCGGCGCGATAGTGTGAACATGCTATAGACTTC
TGGTGCTACCCGACTGACAATTAATCATCCGGCTCGTATAATGCTAGCGCCGCGATTAAATTCCAACAGTTTTAGAGCTAGAA
ATAGCAAGTTAAAATAAGGCTAGTCCGTTATCAACTTGAAAAAGTGGCACCGAGTCGGTGCTTTTTTTAATCCATTCGAATCA
CGTCCGACGAACAATAAGGCCTCCCTAACGGGGGGCCTTTTTTATTGATAACAAAAGTAACTTCGAGCTTGTCTACCTCCTAG
CACCATTATTGCAATTAATAAACAACTAACGGACAATTCTACCTAACAGTTTTCATATATGACGAGCAGTTAAGTGATGAGTA
AAGGTGAGGAATTATTTACTGGTGTTGTTCCGATCTTAGTTGAACTGGACGGCGATGTTAACGGTCATAAATTCAGTGTTCGT
GGTGAAGGTGAAGGTGATGCAACCAACGGTAAGCTGACCCTGAAATTCATCTGCACTACTGGAAAATTACCAGTACCGTGGCC
TACTCTGGTGACTACCCTGACCTATGGTGTTCAGTGTTTTTCTCGTTACCCTGACCACATGAAGCAACATGATTTCTTCAAAT
CTGCAATGCCGGAAGGTTATGTACAGGAGCGCACCATTTCTTTCAAAGACGATGGCACGTATAAAACCCGTGCAGAGGTTAAA
TTTGAAGGTGACACTCTGGTGAATCGTATTGAACTGAAAGGCATTGATTTCAAAGAGGACGGCAATATTTTAGGCCACAAACT
GGAATATAACTTCAACTCCCATAACGTTTACATCACCGCAGACAAACAGAAGAACGGTATCAAAGCTAACTTCAAAATTCGCC
ATAACGTTGAAGATGGTAGCGTACAGCTGGCGGATCATTACCAACAGAACACTCCGATTGGAGATGCTCCTGTTTTACTGCCG
GATAACCACTACCTGTCCACCCAGTCTAAACTGTCGAAGGATCCGAACGAAAAGCGCGACCACATGGTGTTATTAGAGTTCGT
TACCGCTAGTGGTATCACGCACGGTATGGATGAACTCTACAAATAAGACGAACAATAAGGGGAGCGGAAACCGCTCCCCTTT
TTTATTGATAACAAAAGTAAATTGCACGCTGATAGTCTCCCAATTGCGAAGGACCAAAACGAAAAAACACCCTTTCGGGTGTC

TTTTCTGGAATTTGGTACCGAGTACTAGGTATCGTGTAAGTAGCGAAGGCCCGTACGCGAGATAAACTGCTAGGCAACCGCGA
CTCTACGACTGGTGCTCGATTTAATTTCGCTGACGTAAAGAAATTATCGGCAGTGCGTCAACTGCCGTATCTTTATCTTAATT
AGGTAGTTGGACAAGCCCTTGAAAGAAATAGCAAGAGCCTGCCTCTCTATTGAAGTCACGGCGAAAGTCGGGTAGAAATCAAA
GAAAGCAGAAATTAAATCGGAGTAACACTAAGGTGGGATAACTCCGTAACTGACTACGCCTTTCTCTAGACTTTACTTGACCA
GATACACTGTCTTTGACACGTTGAAGGATTAGAGCAATCAAATCCAAGACTGGCTAAGCACGAAGCAACTCTTGAGTGTTAAA
AAGTTATCTCCTGTATTCGGGAAGCGGGTACTAGAAGATTGCAGGGACTCCGACGTTAAGTAAATTACAAAGTAATAAGTATC
GTTCAGGATCACGTTACCGCAATAAGAAGCGAGAATAATATAATTTCCGAAGTGCTTACCCCAGTAGTGACTATTCCTATAAC
CCTTCTGAGTGTCCGGAGGCGGAAATTTGCCACGAAAGAGAAAGTATTTCCCCGACAATAATAAAGGGGCGCTCCTCAGCTTT
TCCACTTGGTTGGGTAAGCTAGGCAACTCTGAAAGGAGTTTCGGCGAATTGAAGCCGACAGCTTTGAATTGTTTTAGGGGCGT
TATTCGAGGGCAATCGGAGCTAACTTCAAGACTACTTCTTTGTTGAATACTAAATAGTGCAAAGGTCGTGTTTCCTCAAGGAT
ACTCCGCTAACAATATAGGATTCCAATCAGATTCAGCACTGGCGGTACGGGTGTTGCGGTGAGGCGTTCGGGTTTACGGCTCG
AAGCTAGCACGGTAGG

p2798

CGCAGGATAAGTAAGGGGAGTAAGTGATCGAACGAATCAGAAGTGACAATATACTTAGGCTGGATCTCGTCCCGTGAATCCCA
ACCCTCACCAACTACGAGATAAGAGGTAAGCCAAAAATCGACTTGGTGGCGACCAACGACTGTTCCCCCCCTGTAACTAATCG
TTCCGTCAAAACCTGACTTACTTCAAGGCCAATTCCAAGCGCAAACAATACCGTCCTAGTTCTTCGGTTAAGTTTCCGAAGTA
GGAGTGAGCCTACCTCCGTTTGCGTCTTGTTACCACTGACCCAGCTATTTACTTTGTATTGCCTGCAATCGAATTTCTGAACT
CTCAGATAGTGGGGATAACGGGAAAGTTCCTATATTTGCGAACTAACTTAGCCGTCCACCTCGAAGCTACCTACTCACACCCA
CCCCGCGCGGGGTAAATAAGGCACTAATCCCAGCTGAGAGCTGGCGTAGCACTTAGCCACAAGTTAATTAACAGTTGTCTGGT
AGTTTGGCGGTATTAGGAAGATCCTAGAAGCAAGGCAGAGTTAGTTCTAACCTAAAGCCACAAATAAGACAGGTTGCCAAAGC
CCGCCGGAAATTAAATCTTGCTCAGTTCGGTAACGGAGTTTCCCTCCCGCGTACTTAATTCCCAATAAGAAACGCGCCCAAGT
CCTATCAGGCAAAATTCAGCCCCTTCCCGTGTTAGAACGAGGGTAAAAATACAAGCCGATTGAACAAGGGTTGGGGGCTTCAA
ATCGTCGTTTACCCCACTTTACAACGGAGATTAAGTAGTTCACCCTATAGTACGAAGCAGAACTATTTCGAGGGGCGTGCAAT
AATCGAATCTTCTGCGGTTGACTTAACACGCTAGGGACGTGCCCTCGATTCAATCGAAGGTACTCCTACTCAGACTGCCTCAC
ACCCAGCTAGTCACTGAGCGATAAAATTGACCCGCCCTCTAGGGAAGCGAGTACGTCCCAAAGGGCTCCGGACAGGGCTATAT
AGGAGAGTTTGATCTCGCCCCGACAACTGCAACCCTCAACTCCCTTAGATAATATTGTTAGCCGAAGTTGCACGACCCGCCGT
CCACGGACTGCTCTTAGGGTGTGGCTCCTTAATCTGACAACGTGCAACCCCTATCGAAGTCGATTGTTTCTGCGAAAGGTGTT
GTCCTAATAGTCCCGAAATTTGGCCCTTGTAGGTGTGAAACCACTTAGCTTCGCGCCGTAGTCCTAAAGGCCCACCTATTGAC
TTTGTTTCGGGTAGCACTAGGAATCTTAACAATTTGAATTTGGACGTGGAACGCGTACACCTTAATCTCCGAATAATTCTAGG
GATTTGGAAGTCCTCTACGTTGACACACCTACACTGCTCGAAGTAAATATACGAATAACGCGGGCCTCGCGGAGCCGTTCCGA
ATCGTCACGTGTTCGTTTACTGTTAATTGGTGGCAAATAAGCAATATCGTAGTCCGTCAGGCCCAGCCCTGTTATCCACGGCG
TTATTTGTCAAATTGCGTAGAACTGGATTGACTGCCTGACAATACCTAATTATCGGTACGAAGTCCCCGAATCTGTCGGGCTA
TTTCACTAATACTTTCCAAACGCCCCGTATCCAAGAAGAACGAATTTATCCACGCTCCCGTCTTTGGGACGAATACCGCTACA
AGTGGACAGAGGATCGGTACGGGCCTCTAATAAATCCAACACTCTACGCCCTCTTCAAGAGCTAGAAGAACAGGGTGCAGTTG
GAAAGGGAATTATTTCGTAAGGCGAGCCAATACCGTAATTAATTCGGAAGAGTTAACACGATTGGAAGTAGGAATAGTTTCTA
ACCACGGTTACTAATCCTAATAACGGAACGCTGTCTGATAGATTAGTGTCAGCGCTCGGTACCAAAGAAAAATAAAAAGACGC
TGAAAAGCGTCTTTTTATTTTTCGGTCCAGTGTAACTCAGGCAAAAGCACGTAATATTCGTACTTTCTTCCTCCGTAAGCGTC
ACCCACATTCCTTAAAGAGTGCATGTGCATATTTTGTTATCAATAAAAAAGGCCGCGATTTGCGGCCTTATTGTTCGTCTTGC
CGGATTACGCCCCGCCCTGCCACTCATCGCAGTATTGTTGTAATTCATTAAGCATTCTGCCGACATGGAAGCCATCACAAACG
GCATGATGAACTTGGATCGCCAGTGGCATTAACACCTTGTCGCCTTGCGTATAATATTTCCCATAGTGAAAACGGGGGCGAA
GAAGTTGTCCATATTTGCTACGTTTAAATCAAAACTGGTGAAACTCACCCACGGATTGGCACTGACGAAAAACATATTTTCGA
TAAACCCTTTAGGGAAATATGCTAAGTTTTCACCGTAACACGCCACATCTTGACTATATATGTGTAGAAACTGCCGGAAATCG
TCGTGGTATTCTGACCAGAGCGATGAAAACGTTTCAGTTTGCTCATGGAAAACGGTGTAACAAGGGTGAACACTATCCCATAT
CACCAGCTCACCGTCTTTCATTGCCATACGAAACTCCGGATGTGCATTCATCAGGCGGGCAAGAATGTGAATAAAGGCCGGAT
AAAACTTGTGCTTATTTTTCTTTACGGTTTTTAAAAAGGCCGTAATATCCAGCTGAACGGTTTGGTTATAGGTGCACTGAGCA
ACTGACTGGAATGCCTCAAAATGTTCTTTACGATGCCATTGACTTATATCAACTGTAGTATATCCAGTGATTTTTTTCTCCAT
TTTAGCTTCCTTAGCTTGCGAAATCTCGATAACTCAAAAAATAGTAGTGATCTTATTTCATTATGGTGAAAGTTGTCTTACGT
GCAACATTTTCGCAAAAAGTTGGCGCTTTATCAACACTGTCGGAATGACAAATGGTTCCAATTATTGAACACCCTTCGGGGTG
TTTTTTTGTTTCTGGTTTCCCGAGGCCGGCCTTTTGTTGCAATGGCTGTCTACCCTGTCTACCTGAGTAAAGAAAAATACATT
TAATTCAGTATATTAACTTGGGTAGACAGCCTTTTTTTACTGTCTACCTTCTGTCTACCCTCTCTACCTGATTTTACCTGAAT
CAGACAGGGAGGTAGACACGGGGTAGACAGTGGATAAAAGCACTCTACCCCACTGAAAGCAGTGCCATTACTGGCATGGTTGC
CAGTAAGGTTGATAAGGTAGACAAGGGGAGGGACAACTCAAAACTTTTTAAACGAGGGGGTAAAACGCAGACCAAAACGATCT
CAAGAAGATCATCTTATTAATCAGATAAAATATTTCTAGATTTCAGTGCAATTTATCTCTTCAAATGTAGCACCGGCGCGCCG
TGACCAATTATTGAAGGCCGCTAACGCGGCCTTTTTTTGTTTCTGGTATCCCGAATGGAGCGACTTCTCCCCAAAAAGCCTCG
CTTTCAGCACCTGTCGTTTCCTTTCTTTTCAGAGGGTATTTTAAATAAAAACATTAAGTTATGACGAAGAAGAACGGAAACGC
CTTAAACCGGAAAATTTTCATAAATAGCGAAAACCCGCGAGGTCGCCGCCCCGTAACCTGTCGGATCACCGGAAAGGACCCGT
AAAGTGATAATGATTATCATCTACATATCACAACGTGCGTAAAGGGTAAGTATGAAGGTCGTGTACTCCATCGCTACCAAATT
CCAGAAAACAGACGCTTTCGAGCGTCTTTTTTCGTTTTGGTCACGACGTACGGTGGAAGATTCGTTACCAATTGACAGCTAGC
TCAGTCCTAGGTATATACATACATGCTTGTTTGTTTGTAAACCAGGATAAAGAGAGAGCATACTCGATGAAACGTACTGCCGA
CGGTTCAGAGTTCGAGTCGCCCAAGAAGAAACGTAAAGTGTCATCCAAAACCGGCCCAGTTGCGGTGGACCCAACGCTGCGCC

GTCGTATCGAACCGCACGAGTTCGAAGTATTTTTCGATCCTCGCGAACTGCGTAAAGAGACCTGTTTACTTTACGAAATCAAT
TGGGGTGGCCGCCACTCGATTTGGCGCCACACGTCTCAGAATACAAACAAACATGTCGAAGTAAATTTCATCGAAAAATTTAC
AACGGAGCGTTATTTCTGTCCCAATACTCGTTGTTCTATTACATGGTTTTTATCATGGAGTCCCTGCGGGGAATGCTCTCGTG
CGATCACGGAGTTTCTGTCGCGTTATCCGAACGTAACGCTTTTTATCTATATTGCTCGTCTTTACCACTTGGCTAACCCTCGT
AACCGCCAAGGACTTCGCGACCTGATTTCGAGTGGAGTAACCATCCAGATCATGACTGAGCAAGAATCTGGATACTGTTGGCA
CAATTTCGTAAACTACTCGCCATCGAATGAGAGCCATTGGCCTCGCTACCCCCATCTTTGGGTACGTTTGTATGTCTTGGAAC
TTTATTGTATCATTTTAGGGCTGCCACCTTGTCTTAATATTTTGCGCCGCAAACAGAGTCAGCTGACATCTTTCACAATTGCT
TTACAGTCATGCCACTACCAACGTCTTCCACCGCACATTCTGTGGGCCACGGGCTTAAAATCGGGCGGTAGCTCAGGCGGGAG
TTCAGGCTCAGAAACGCCGGGAACTAGCGAATCCGCAACGCCAGAGTCTTCAGGTGGTTCGTCAGGTGGTTCAGACAAGAAAT
ATAGCATCGGCCTGGCCATCGGCACAAATAGCGTCGGATGGGCGGTGATCACTGATGAATATAAAGTTCCGTCTAAAAAGTTC
AAGGTACTGGGTAATACAGATCGCCATAGTATCAAAAAGAACTTAATCGGTGCGCTTCTGTTCGATTCCGGCGAAACCGCAGA
AGCAACACGTCTGAAACGCACCGCTCGTCGCCGTTACACCCGTCGTAAAAACCGCATCTGCTACCTGCAAGAAATCTTCTCTA
ACGAAATGGCTAAAGTAGATGACAGCTTTTTTCACCGTCTGGAAGAATCATTTCTGGTGGAAGAAGATAAAAAGCACGAACGT
CATCCAATCTTCGGCAACATTGTGGACGAAGTAGCGTATCACGAAAAATACCCGACTATCTATCACCTGCGCAAAAAGCTGGT
CGATTCGACGGATAAGGCCGATCTGCGTCTGATCTATCTGGCCTTAGCGCATATGATTAAGTTCCGTGGTCATTTCCTGATCG
AAGGCGACCTGAATCCAGACAACAGCGATGTAGACAAACTGTTCATCCAGCTGGTGCAAACCTATAACCAGCTGTTTGAAGAA
AACCCAATTAATGCTAGCGGTGTTGACGCGAAAGCGATCTTGTCCGCACGCCTGTCCAAATCCCGTCGTCTGGAAAACTTAAT
TGCGCAACTGCCGGGTGAGAAGAAAAACGGACTGTTCGGCAATCTGATCGCTCTTAGCTTGGGACTGACCCCGAACTTCAAAA
GCAACTTCGATCTGGCAGAGGACGCAAAACTTCAACTTAGCAAAGATACGTATGACGATGACTTGGATAACTTACTGGCCCAG
ATCGGAGATCAGTACGCTGATCTGTTTCTGGCGGCAAAGAACTTATCAGACGCTATTCTCCTGTCTGATATTCTTCGTGTGAA
TACCGAAATCACCAAAGCACCGCTTTCTGCATCCATGATTAAACGCTATGACGAACATCACCAAGATCTGACTCTTCTGAAAG
CGCTGGTACGGCAACAACTGCCGGAGAAGTACAAGGAGATCTTCTTTGACCAATCCAAAAACGGCTACGCGGGTTATATTGAC
GGGGGTGCAAGCCAAGAGGAGTTCTACAAATTCATCAAGCCAATCTTAGAAAAAATGGATGGCACGGAAGAATTACTTGTTAA
ACTGAATCGTGAGGATCTGCTTCGTAAACAGCGTACCTTCGACAACGGTAGCATTCCGCACCAGATCCACTTAGGTGAACTGC
ACGCTATCCTGCGTCGCCAAGAGGATTTTTACCCGTTCCTGAAAGATAATCGTGAAAAAATCGAAAAAATCCTGACCTTTCGT
ATCCCGTATTATGTCGGCCCGCTGGCGCGTGGCAACTCCCGTTTCGCGTGGATGACTCGCAAATCCGAAGAAACTATTACCCC
GTGGAACTTCGAGGAAGTGGTTGACAAAGGCGCAAGCGCCCAATCCTTCATCGAGCGCATGACTAACTTTGATAAAAACCTGC
CGAACGAAAAGGTACTGCCGAAACACTCCCTTCTGTACGAATACTTCACCGTGTACAACGAGCTGACTAAAGTAAAGTATGTG
ACTGAGGGCATGCGTAAACCTGCATTCCTGAGCGGTGAACAGAAAAAAGCAATTGTTGATTTACTGTTTAAAACCAACCGTAA
AGTAACCGTTAAACAGCTGAAAGAGGACTACTTCAAGAAAATCGAATGCTTCGACTCCGTCGAGATTAGTGGAGTTGAAGATC
GTTTTAATGCAAGTTTAGGCACGTATCACGATTTATTAAAGATCATTAAAGACAAAGATTTCTTGGACAACGAAGAAAATGAG
GACATCTTAGAGGACATCGTCCTGACCCTGACTCTGTTCGAAGATCGTGAAATGATTGAAGAACGCCTTAAGACGTATGCTCA
CCTGTTTGACGATAAAGTAATGAAACAACTGAAACGTCGCCGTTATACTGGCTGGGGCCGTCTGAGCCGTAAACTGATTAACG
GTATCCGTGACAAACAGTCCGGTAAAACTATTCTGGACTTCCTGAAATCTGACGGCTTCGCAAACCGTAACTTCATGCAACTG
ATTCACGACGATTCCCTGACCTTCAAAGAGGACATCCAGAAAGCTCAGGTTTCTGGTCAAGGTGATTCTCTGCACGAGCATAT
CGCCAATTTAGCAGGTAGTCCGGCGATCAAAAAAGGTATCCTGCAAACCGTGAAAGTGGTGGATGAGCTTGTGAAAGTTATGG
GTCGTCACAAACCGGAAAACATTGTTATCGAGATGGCTCGTGAAAACCAAACGACCCAGAAGGGACAGAAAAACTCCCGCGAA
CGCATGAAACGTATCGAGGAGGGTATTAAAGAACTTGGCTCTCAGATTCTGAAAGAACACCCTGTTGAAAATACCCAACTGCA
AAATGAAAAACTGTACCTGTACTACCTGCAAAATGGTCGTGACATGTATGTAGATCAGGAGCTGGACATCAACCGCCTCTCCG
ATTACGACGTTGACCACATTGTTCCGCAGTCTTTTCTGAAAGATGATTCCATTGATAACAAAGTACTCACCCGTAGCGATAAA
AACCGTGGGAAGAGTGACAACGTTCCATCGGAAGAAGTAGTTAAGAAAATGAAGAACTATTGGCGTCAACTGCTTAACGCGAA
ACTGATTACTCAACGTAAATTTGATAACCTGACCAAAGCTGAACGTGGCGGTTTGTCTGAGCTGGATAAGGCGGGTTTTATTA
AACGTCAACTGGTAGAAACTCGCCAGATTACAAAACATGTTGCTCAGATTCTGGACTCTCGTATGAACACTAAATACGATGAA
AATGACAAACTGATCCGCGAAGTTAAGGTTATTACCCTGAAATCTAAGCTGGTTTCCGACTTCCGTAAAGATTTCCAATTCTA
TAAAGTGCGCGAGATTAACAACTATCACCACGCGCACGACGCATATCTGAATGCAGTTGTTGGCACGGCACTGATCAAAAAAT
ATCCGAAACTGGAAAGCGAATTTGTGTACGGCGATTATAAAGTTTACGACGTGCGCAAAATGATCGCCAAATCTGAACAGGAA
ATTGGCAAAGCAACCGCTAAATACTTTTTCTACTCAAACATTATGAATTTCTTCAAAACCGAAATCACCTTAGCGAATGGCGA
AATTCGTAAACGCCCTCTGATCGAAACCAACGGCGAAACGGGTGAGATCGTGTGGGACAAAGGTCGTGATTTCGCTACTGTCC
GCAAAGTTCTGTCCATGCCTCAAGTAAACATCGTTAAAAAGACTGAGGTACAGACTGGCGGTTTCAGCAAGGAATCCATTCTG
CCGAAACGCAACTCCGACAAACTGATCGCGCGTAAGAAAGACTGGGATCCGAAGAAATACGGTGGCTTCGATTCTCCAACCGT
GGCATACAGCGTTCTGGTAGTCGCCAAAGTCGAAAAGGGTAAATCAAAAAAACTGAAATCAGTGAAAGAACTTTTAGGCATCA
CCATTATGGAACGTAGCTCTTTCGAAAAAAACCCGATTGACTTCCTCGAAGCGAAGGGGTACAAGGAAGTAAAGAAAGATCTG
ATTATCAAACTGCCGAAGTATTCCCTGTTCGAACTGGAAAATGGTCGTAAACGTATGTTAGCGTCTGCGGGTGAACTGCAAAA
AGGGAACGAATTGGCCCTTCCGTCCAAGTACGTGAACTTCCTGTATCTGGCCTCGCACTACGAGAAACTGAAAGGTAGTCCGG
AAGATAATGAGCAGAAACAGCTGTTCGTGGAACAGCACAAACACTATCTGGACGAGATTATTGAACAGATTTCTGAGTTTAGC
AAACGCGTAATTCTGGCGGACGCGAATCTGGATAAAGTCCTGAGCGCCTACAATAAACACCGTGATAAACCGATCCGTGAACA
GGCAGAAAACATCATTCACCTGTTCACGCTGACTAATCTTGGTGCTCCGGCAGCCTTCAAATACTTCGACACCACGATCGATC
GTAAACGTTACACCTCCACTAAAGAAGTCTTAGATGCAACTCTTATTCACCAGAGCATCACTGGCCTGTATGAAACTCGTATT
GATCTGAGTCAGTTGGGCGGTGACAGTGGGGGCAGCGGAGGTAGCGGAGGCAGCACTAATTTGTCGGATATTATTGAGAAGGA
AACAGGAAAGCAATTGGTTATCCAGGAATCCATTTTAATGCTTCCGGAAGAAGTGGAAGAGGTGATCGGAAATAAGCCAGAGA
GCGATATCTTAGTTCATACGGCCTATGATGAAAGCACTGACGAGAACGTTATGCTGTTAACATCGGACGCTCCCGAGTACAAG
CCGTGGGCCCTGGTTATCCAGGACAGTAATGGCGAAAATAAAATTAAAATGTTATAATGTATGCTTAAGCAGCTCGGTACCAA
AGACGAACAATAAGACGCTGAAAAGCGTCTTTTTTCGTTTTGGTCCTGTTGCGGCGCGATAGTGTGAACATGCTATAGACTTC

TGGTGCTACCCGACTGACAATTAATCATCCGGCTCGTATAATGCTAGCATTTTCAGATTAAAATTAGAGTTTTAGAGCTAGAA
ATAGCAAGTTAAAATAAGGCTAGTCCGTTATCAACTTGAAAAAGTGGCACCGAGTCGGTGCTTTTTTTAATCCATTCGAATCA
CGTCCGACGAACAATAAGGCCTCCCTAACGGGGGGCCTTTTTTATTGATAACAAAAGTAACTTCGAGCTTGTCTACCTCCTAG
CACCATTATTGCAATTAATAAACAACTAACGGACAATTCTACCTAACAGTTTTCATATATGACGAGCAGTTAAGTGATGAGTA
AAGGTGAGGAATTATTTACTGGTGTTGTTCCGATCTTAGTTGAACTGGACGGCGATGTTAACGGTCATAAATTCAGTGTTCGT
GGTGAAGGTGAAGGTGATGCAACCAACGGTAAGCTGACCCTGAAATTCATCTGCACTACTGGAAAATTACCAGTACCGTGGCC
TACTCTGGTGACTACCCTGACCTATGGTGTTCAGTGTTTTTCTCGTTACCCTGACCACATGAAGCAACATGATTTCTTCAAAT
CTGCAATGCCGGAAGGTTATGTACAGGAGCGCACCATTTCTTTCAAAGACGATGGCACGTATAAAACCCGTGCAGAGGTTAAA
TTTGAAGGTGACACTCTGGTGAATCGTATTGAACTGAAAGGCATTGATTTCAAAGAGGACGGCAATATTTTAGGCCACAAACT
GGAATATAACTTCAACTCCCATAACGTTTACATCACCGCAGACAAACAGAAGAACGGTATCAAAGCTAACTTCAAAATTCGCC
ATAACGTTGAAGATGGTAGCGTACAGCTGGCGGATCATTACCAACAGAACACTCCGATTGGAGATGCTCCTGTTTTACTGCCG
GATAACCACTACCTGTCCACCCAGTCTAAACTGTCGAAGGATCCGAACGAAAAGCGCGACCACATGGTGTTATTAGAGTTCGT
TACCGCTAGTGGTATCACGCACGGTATGGATGAACTCTACAAATAAGACGAACAATAAGGGGAGCGGGAAACCGCTCCCCTTT
TTTATTGATAACAAAAGTAAATTGCACGCTGATAGTCTCCCAATTGCGAAGGACCAAAACGAAAAAACACCCTTTCGGGTGTC
TTTTCTGGAATTTGGTACCGAGTACTAGGTATCGTGTAAGTAGCGAAGGCCCGTACGCGAGATAAACTGCTAGGCAACCGCGA
CTCTACGACTGGTGCTCGATTTAATTTCGCTGACGTAAAGAAATTATCGGCAGTGCGTCAACTGCCGTATCTTTATCTTAATT
AGGTAGTTGGACAAGCCCTTGAAAGAAATAGCAAGAGCCTGCCTCTCTATTGAAGTCACGGCGAAAGTCGGGTAGAAATCAAA
GAAAGCAGAAATTAAATCGGAGTAACACTAAGGTGGGATAACTCCGTAACTGACTACGCCTTTCTCTAGACTTTACTTGACCA
GATACACTGTCTTTGACACGTTGAAGGATTAGAGCAATCAAATCCAAGACTGGCTAAGCACGAAGCAACTCTTGAGTGTTAAA
AAGTTATCTCCTGTATTCGGGAAGCGGGTACTAGAAGATTGCAGGGACTCCGACGTTAAGTAAATTACAAAGTAATAAGTATC
GTTCAGGATCACGTTACCGCAATAAGAAGCGAGAATAATATAATTTCCGAAGTGCTTACCCCAGTAGTGACTATTCCTATAAC
CCTTCTGAGTGTCCGGAGGCGGAAATTTGCCACGAAAGAGAAAGTATTTCCCCGACAATAATAAAGGGGCGCTCCTCAGCTTT
TCCACTTGGTTGGGTAAGCTAGGCAACTCTGAAAGGAGTTTCGGCGAATTGAAGCCGACAGCTTTGAATTGTTTTAGGGGCGT
TATTCGAGGGCAATCGGAGCTAACTTCAAGACTACTTCTTTGTTGAATACTAAATAGTGCAAAGGTCGTGTTTCCTCAAGGAT
ACTCCGCTAACAATATAGGATTCCAATCAGATTCAGCACTGGCGGTACGGGTGTTGCGGTGAGGCGTTCGGGTTTACGGCTCG
AAGCTAGCACGGTAGG

p2800

CGCAGGATAAGTAAGGGGAGTAAGTGATCGAACGAATCAGAAGTGACAATATACTTAGGCTGGATCTCGTCCCGTGAATCCCA
ACCCTCACCAACTACGAGATAAGAGGTAAGCCAAAAATCGACTTGGTGGCGACCAACGACTGTTCCCCCCCTGTAACTAATCG
TTCCGTCAAAACCTGACTTACTTCAAGGCCAATTCCAAGCGCAAACAATACCGTCCTAGTTCTTCGGTTAAGTTTCCGAAGTA
GGAGTGAGCCTACCTCCGTTTGCGTCTTGTTACCACTGACCCAGCTATTTACTTTGTATTGCCTGCAATCGAATTTCTGAACT
CTCAGATAGTGGGGATAACGGGAAAGTTCCTATATTTGCGAACTAACTTAGCCGTCCACCTCGAAGCTACCTACTCACACCCA
CCCCGCGCGGGGTAAATAAGGCACTAATCCCAGCTGAGAGCTGGCGTAGCACTTAGCCACAAGTTAATTAACAGTTGTCTGGT
AGTTTGGCGGTATTAGGAAGATCCTAGAAGCAAGGCAGAGTTAGTTCTAACCTAAAGCCACAAATAAGACAGGTTGCCAAAGC
CCGCCGGAAATTAAATCTTGCTCAGTTCGGTAACGGAGTTTCCCTCCCGCGTACTTAATTCCCAATAAGAAACGCGCCCAAGT
CCTATCAGGCAAAATTCAGCCCCTTCCCGTGTTAGAACGAGGGTAAAAATACAAGCCGATTGAACAAGGGTTGGGGGCTTCAA
ATCGTCGTTTACCCCACTTTACAACGGAGATTAAGTAGTTCACCCTATAGTACGAAGCAGAACTATTTCGAGGGGCGTGCAAT
AATCGAATCTTCTGCGGTTGACTTAACACGCTAGGGACGTGCCCTCGATTCAATCGAAGGTACTCCTACTCAGACTGCCTCAC
ACCCAGCTAGTCACTGAGCGATAAAATTGACCCGCCCTCTAGGGAAGCGAGTACGTCCCAAAGGGCTCCGGACAGGGCTATAT
AGGAGAGTTTGATCTCGCCCCGACAACTGCAACCCTCAACTCCCTTAGATAATATTGTTAGCCGAAGTTGCACGACCCGCCGT
CCACGGACTGCTCTTAGGGTGTGGCTCCTTAATCTGACAACGTGCAACCCCTATCGAAGTCGATTGTTTCTGCGAAAGGTGTT
GTCCTAATAGTCCCGAAATTTGGCCCTTGTAGGTGTGAAACCACTTAGCTTCGCGCCGTAGTCCTAAAGGCCCACCTATTGAC
TTTGTTTCGGGTAGCACTAGGAATCTTAACAATTTGAATTTGGACGTGGAACGCGTACACCTTAATCTCCGAATAATTCTAGG
GATTTGGAAGTCCTCTACGTTGACACACCTACACTGCTCGAAGTAAATATACGAATAACGCGGGCCTCGCGGAGCCGTTCCGA
ATCGTCACGTGTTCGTTTACTGTTAATTGGTGGCAAATAAGCAATATCGTAGTCCGTCAGGCCCAGCCCTGTTATCCACGGCG
TTATTTGTCAAATTGCGTAGAACTGGATTGACTGCCTGACAATACCTAATTATCGGTACGAAGTCCCCGAATCTGTCGGGCTA
TTTCACTAATACTTTCCAAACGCCCCGTATCCAAGAAGAACGAATTTATCCACGCTCCCGTCTTTGGGACGAATACCGCTACA
AGTGGACAGAGGATCGGTACGGGCCTCTAATAAATCCAACACTCTACGCCCTCTTCAAGAGCTAGAAGAACAGGGTGCAGTTG
GAAAGGGAATTATTTCGTAAGGCGAGCCAATACCGTAATTAATTCGGAAGAGTTAACACGATTGGAAGTAGGAATAGTTTCTA
ACCACGGTTACTAATCCTAATAACGGAACGCTGTCTGATAGATTAGTGTCAGCGCTCGGTACCAAAGAAAAATAAAAAGACGC
TGAAAAGCGTCTTTTTATTTTTCGGTCCAGTGTAACTCAGGCAAAAGCACGTAATATTCGTACTTTCTTCCTCCGTAAGCGTC
ACCCACATTCCTTAAAGAGTGCATGTGCATATTTTGTTATCAATAAAAAAGGCCGCGATTTGCGGCCTTATTGTTCGTCTTGC
CGGATTACGCCCCGCCCTGCCACTCATCGCAGTATTGTTGTAATTCATTAAGCATTCTGCCGACATGGAAGCCATCACAAACG
GCATGATGAACTTGGATCGCCAGTGGCATTAACACCTTGTCGCCTTGCGTATAATATTTTCCCATAGTGAAAACGGGGGCGAA
GAAGTTGTCCATATTTGCTACGTTTAAATCAAAACTGGTGAAACTCACCCACGGATTGGCACTGACGAAAAACATATTTTCGA
TAAACCCTTTAGGGAAATATGCTAAGTTTTCACCGTAACACGCCACATCTTGACTATATATGTGTAGAAACTGCCGGAAATCG
TCGTGGTATTCTGACCAGAGCGATGAAAACGTTTCAGTTTGCTCATGGAAAACGGTGTAACAAGGGTGAACACTATCCCATAT
CACCAGCTCACCGTCTTTCATTGCCATACGAAACTCCGGATGTGCATTCATCAGGCGGGCAAGAATGTGAATAAAGGCCGGAT
AAAACTTGTGCTTATTTTTCTTTACGGTTTTTAAAAAGGCCGTAATATCCAGCTGAACGGTTTGGTTATAGGTGCACTGAGCA
ACTGACTGGAATGCCTCAAAATGTTCTTTACGATGCCATTGACTTATATCAACTGTAGTATATCCAGTGATTTTTTTCTCCAT

TTTAGCTTCCTTAGCTTGCGAAATCTCGATAACTCAAAAAATAGTAGTGATCTTATTTCATTATGGTGAAAGTTGTCTTACGT
GCAACATTTTCGCAAAAAGTTGGCGCTTTATCAACACTGTCGGAATGACAAATGGTTCCAATTATTGAACACCCTTCGGGGTG
TTTTTTTGTTTCTGGTTTCCCGAGGCCGGCCTTTTGTTGCAATGGCTGTCTACCCTGTCTACCTGAGTAAAGAAAAATACATT
TAATTCAGTATATTAACTTGGGTAGACAGCCTTTTTTTACTGTCTACCTTCTGTCTACCCTCTCTACCTGATTTTACCTGAAT
CAGACAGGGAGGTAGACACGGGGTAGACAGTGGATAAAAGCACTCTACCCCACTGAAAGCAGTGCCATTACTGGCATGGTTGC
CAGTAAGGTTGATAAGGTAGACAAGGGGAGGGACAACTCAAAACTTTTTAAACGAGGGGGTAAAACGCAGACCAAAACGATCT
CAAGAAGATCATCTTATTAATCAGATAAAATATTTCTAGATTTCAGTGCAATTTATCTCTTCAAATGTAGCACCGGCGCGCCG
TGACCAATTATTGAAGGCCGCTAACGCGGCCTTTTTTTGTTTCTGGTATCCCGAATGGAGCGACTTCTCCCCAAAAAGCCTCG
CTTTCAGCACCTGTCGTTTCCTTTCTTTTCAGAGGGTATTTTAAATAAAAACATTAAGTTATGACGAAGAAGAACGGAAACGC
CTTAAACCGGAAAATTTTCATAAATAGCGAAAACCCGCGAGGTCGCCGCCCCGTAACCTGTCGGATCACCGGAAAGGACCCGT
AAAGTGATAATGATTATCATCTACATATCACAACGTGCGTAAAGGGTAAGTATGAAGGTCGTGTACTCCATCGCTACCAAATT
CCAGAAAACAGACGCTTTCGAGCGTCTTTTTTCGTTTTGGTCACGACGTACGGTGGAAGATTCGTTACCAATTGACAGCTAGC
TCAGTCCTAGGTATATACATACATGCTTGTTTGTTTGTAAACCAGGATAAAGAGAGAGCATACTCGATGAAACGTACTGCCGA
CGGTTCAGAGTTCGAGTCGCCCAAGAAGAAACGTAAAGTGTCATCCAAAACCGGCCCAGTTGCGGTGGACCCAACGCTGCGCC
GTCGTATCGAACCGCACGAGTTCGAAGTATTTTTCGATCCTCGCGAACTGCGTAAAGAGACCTGTTTACTTTACGAAATCAAT
TGGGGTGGCCGCCACTCGATTTGGCGCCACACGTCTCAGAATACAAACAAACATGTCGAAGTAAATTTCATCGAAAAATTTAC
AACGGAGCGTTATTTCTGTCCCAATACTCGTTGTTCTATTACATGGTTTTTATCATGGAGTCCCTGCGGGGAATGCTCTCGTG
CGATCACGGAGTTTCTGTCGCGTTATCCGAACGTAACGCTTTTTATCTATATTGCTCGTCTTTACCACTTGGCTAACCCTCGT
AACCGCCAAGGACTTCGCGACCTGATTTCGAGTGGAGTAACCATCCAGATCATGACTGAGCAAGAATCTGGATACTGTTGGCA
CAATTTCGTAAACTACTCGCCATCGAATGAGAGCCATTGGCCTCGCTACCCCCATCTTTGGGTACGTTTGTATGTCTTGGAAC
TTTATTGTATCATTTTAGGGCTGCCACCTTGTCTTAATATTTTGCGCCGCAAACAGAGTCAGCTGACATCTTTCACAATTGCT
TTACAGTCATGCCACTACCAACGTCTTCCACCGCACATTCTGTGGGCCACGGGCTTAAAATCGGGCGGTAGCTCAGGCGGGAG
TTCAGGCTCAGAAACGCCGGGAACTAGCGAATCCGCAACGCCAGAGTCTTCAGGTGGTTCGTCAGGTGGTTCAGACAAGAAAT
ATAGCATCGGCCTGGCCATCGGCACAAATAGCGTCGGATGGGCGGTGATCACTGATGAATATAAAGTTCCGTCTAAAAAGTTC
AAGGTACTGGGTAATACAGATCGCCATAGTATCAAAAAGAACTTAATCGGTGCGCTTCTGTTCGATTCCGGCGAAACCGCAGA
AGCAACACGTCTGAAACGCACCGCTCGTCGCCGTTACACCCGTCGTAAAAACCGCATCTGCTACCTGCAAGAAATCTTCTCTA
ACGAAATGGCTAAAGTAGATGACAGCTTTTTTCACCGTCTGGAAGAATCATTTCTGGTGGAAGAAGATAAAAAGCACGAACGT
CATCCAATCTTCGGCAACATTGTGGACGAAGTAGCGTATCACGAAAAATACCCGACTATCTATCACCTGCGCAAAAAGCTGGT
CGATTCGACGGATAAGGCCGATCTGCGTCTGATCTATCTGGCCTTAGCGCATATGATTAAGTTCCGTGGTCATTTCCTGATCG
AAGGCGACCTGAATCCAGACAACAGCGATGTAGACAAACTGTTCATCCAGCTGGTGCAAACCTATAACCAGCTGTTTGAAGAA
AACCCAATTAATGCTAGCGGTGTTGACGCGAAAGCGATCTTGTCCGCACGCCTGTCCAAATCCCGTCGTCTGGAAAACTTAAT
TGCGCAACTGCCGGGTGAGAAGAAAAACGGACTGTTCGGCAATCTGATCGCTCTTAGCTTGGGACTGACCCCGAACTTCAAAA
GCAACTTCGATCTGGCAGAGGACGCAAAACTTCAACTTAGCAAAGATACGTATGACGATGACTTGGATAACTTACTGGCCCAG
ATCGGAGATCAGTACGCTGATCTGTTTCTGGCGGCAAAGAACTTATCAGACGCTATTCTCCTGTCTGATATTCTTCGTGTGAA
TACCGAAATCACCAAAGCACCGCTTTCTGCATCCATGATTAAACGCTATGACGAACATCACCAAGATCTGACTCTTCTGAAAG
CGCTGGTACGGCAACAACTGCCGGAGAAGTACAAGGAGATCTTCTTTGACCAATCCAAAAACGGCTACGCGGGTTATATTGAC
GGGGGTGCAAGCCAAGAGGAGTTCTACAAATTCATCAAGCCAATCTTAGAAAAAATGGATGGCACGGAAGAATTACTTGTTAA
ACTGAATCGTGAGGATCTGCTTCGTAAACAGCGTACCTTCGACAACGGTAGCATTCCGCACCAGATCCACTTAGGTGAACTGC
ACGCTATCCTGCGTCGCCAAGAGGATTTTTACCCGTTCCTGAAAGATAATCGTGAAAAAATCGAAAAAATCCTGACCTTTCGT
ATCCCGTATTATGTCGGCCCGCTGGCGCGTGGCAACTCCCGTTTCGCGTGGATGACTCGCAAATCCGAAGAAACTATTACCCC
GTGGAACTTCGAGGAAGTGGTTGACAAAGGCGCAAGCGCCCAATCCTTCATCGAGCGCATGACTAACTTTGATAAAAACCTGC
CGAACGAAAAGGTACTGCCGAAACACTCCCTTCTGTACGAATACTTCACCGTGTACAACGAGCTGACTAAAGTAAAGTATGTG
ACTGAGGGCATGCGTAAACCTGCATTCCTGAGCGGTGAACAGAAAAAAGCAATTGTTGATTTACTGTTTAAAACCAACCGTAA
AGTAACCGTTAAACAGCTGAAAGAGGACTACTTCAAGAAAATCGAATGCTTCGACTCCGTCGAGATTAGTGGAGTTGAAGATC
GTTTTAATGCAAGTTTAGGCACGTATCACGATTTATTAAAGATCATTAAAGACAAAGATTTCTTGGACAACGAAGAAAATGAG
GACATCTTAGAGGACATCGTCCTGACCCTGACTCTGTTCGAAGATCGTGAAATGATTGAAGAACGCCTTAAGACGTATGCTCA
CCTGTTTGACGATAAAGTAATGAAACAACTGAAACGTCGCCGTTATACTGGCTGGGGCCGTCTGAGCCGTAAACTGATTAACG
GTATCCGTGACAAACAGTCCGGTAAAACTATTCTGGACTTCCTGAAATCTGACGGCTTCGCAAACCGTAACTTCATGCAACTG
ATTCACGACGATTCCCTGACCTTCAAAGAGGACATCCAGAAAGCTCAGGTTTCTGGTCAAGGTGATTCTCTGCACGAGCATAT
CGCCAATTTAGCAGGTAGTCCGGCGATCAAAAAAGGTATCCTGCAAACCGTGAAAGTGGTGGATGAGCTTGTGAAAGTTATGG
GTCGTCACAAACCGGAAAACATTGTTATCGAGATGGCTCGTGAAAACCAAACGACCCAGAAGGGACAGAAAAACTCCCGCGAA
CGCATGAAACGTATCGAGGAGGGTATTAAAGAACTTGGCTCTCAGATTCTGAAAGAACACCCTGTTGAAAATACCCAACTGCA
AAATGAAAAACTGTACCTGTACTACCTGCAAAATGGTCGTGACATGTATGTAGATCAGGAGCTGGACATCAACCGCCTCTCCG
ATTACGACGTTGACCACATTGTTCCGCAGTCTTTTCTGAAAGATGATTCCATTGATAACAAAGTACTCACCCGTAGCGATAAA
AACCGTGGGAAGAGTGACAACGTTCCATCGGAAGAAGTAGTTAAGAAAATGAAGAACTATTGGCGTCAACTGCTTAACGCGAA
ACTGATTACTCAACGTAAATTTGATAACCTGACCAAAGCTGAACGTGGCGGTTTGTCTGAGCTGGATAAGGCGGGTTTTATTA
AACGTCAACTGGTAGAAACTCGCCAGATTACAAAACATGTTGCTCAGATTCTGGACTCTCGTATGAACACTAAATACGATGAA
AATGACAAACTGATCCGCGAAGTTAAGGTTATTACCCTGAAATCTAAGCTGGTTTCCGACTTCCGTAAAGATTTCCAATTCTA
TAAAGTGCGCGAGATTAACAACTATCACCACGCGCACGACGCATATCTGAATGCAGTTGTTGGCACGGCACTGATCAAAAAAT
ATCCGAAACTGGAAAGCGAATTTGTGTACGGCGATTATAAAGTTTACGACGTGCGCAAAATGATCGCCAAATCTGAACAGGAA
ATTGGCAAAGCAACCGCTAAATACTTTTTCTACTCAAACATTATGAATTTCTTCAAAACCGAAATCACCTTAGCGAATGGCGA
AATTCGTAAACGCCCTCTGATCGAAACCAACGGCGAAACGGGTGAGATCGTGTGGGACAAAGGTCGTGATTTCGCTACTGTCC
GCAAAGTTCTGTCCATGCCTCAAGTAAACATCGTTAAAAAGACTGAGGTACAGACTGGCGGTTTCAGCAAGGAATCCATTCTG

CCGAAACGCAACTCCGACAAACTGATCGCGCGTAAGAAAGACTGGGATCCGAAGAAATACGGTGGCTTCGATTCTCCAACCGT
GGCATACAGCGTTCTGGTAGTCGCCAAAGTCGAAAAGGGTAAATCAAAAAAACTGAAATCAGTGAAAGAACTTTTAGGCATCA
CCATTATGGAACGTAGCTCTTTCGAAAAAAACCCGATTGACTTCCTCGAAGCGAAGGGGTACAAGGAAGTAAAGAAAGATCTG
ATTATCAAACTGCCGAAGTATTCCCTGTTCGAACTGGAAAATGGTCGTAAACGTATGTTAGCGTCTGCGGGTGAACTGCAAAA
AGGGAACGAATTGGCCCTTCCGTCCAAGTACGTGAACTTCCTGTATCTGGCCTCGCACTACGAGAAACTGAAAGGTAGTCCGG
AAGATAATGAGCAGAAACAGCTGTTCGTGGAACAGCACAAACACTATCTGGACGAGATTATTGAACAGATTTCTGAGTTTAGC
AAACGCGTAATTCTGGCGGACGCGAATCTGGATAAAGTCCTGAGCGCCTACAATAAACACCGTGATAAACCGATCCGTGAACA
GGCAGAAAACATCATTCACCTGTTCACGCTGACTAATCTTGGTGCTCCGGCAGCCTTCAAATACTTCGACACCACGATCGATC
GTAAACGTTACACCTCCACTAAAGAAGTCTTAGATGCAACTCTTATTCACCAGAGCATCACTGGCCTGTATGAAACTCGTATT
GATCTGAGTCAGTTGGGCGGTGACAGTGGGGGCAGCGGAGGTAGCGGAGGCAGCACTAATTTGTCGGATATTATTGAGAAGGA
AACAGGAAAGCAATTGGTTATCCAGGAATCCATTTTAATGCTTCCGGAAGAAGTGGAAGAGGTGATCGGAAATAAGCCAGAGA
GCGATATCTTAGTTCATACGGCCTATGATGAAAGCACTGACGAGAACGTTATGCTGTTAACATCGGACGCTCCCGAGTACAAG
CCGTGGGCCCTGGTTATCCAGGACAGTAATGGCGAAAATAAAATTAAAATGTTATAATGTATGCTTAAGCAGCTCGGTACCAA
AGACGAACAATAAGACGCTGAAAAGCGTCTTTTTTCGTTTTGGTCCTGTTGCGGCGCGATAGTGTGAACATGCTATAGACTTC
TGGTGCTACCCGACTGACAATTAATCATCCGGCTCGTATAATGCTAGCTTCAGCAGGTGATAAACACCGTTTTAGAGCTAGAA
ATAGCAAGTTAAAATAAGGCTAGTCCGTTATCAACTTGAAAAAGTGGCACCGAGTCGGTGCTTTTTTTAATCCATTCGAATCA
CGTCCGACGAACAATAAGGCCTCCCTAACGGGGGGCCTTTTTTATTGATAACAAAAGTAACTTCGAGCTTGTCTACCTCCTAG
CACCATTATTGCAATTAATAAACAACTAACGGACAATTCTACCTAACAGTTTTCATATATGACGAGCAGTTAAGTGATGAGTA
AAGGTGAGGAATTATTTACTGGTGTTGTTCCGATCTTAGTTGAACTGGACGGCGATGTTAACGGTCATAAATTCAGTGTTCGT
GGTGAAGGTGAAGGTGATGCAACCAACGGTAAGCTGACCCTGAAATTCATCTGCACTACTGGAAAATTACCAGTACCGTGGCC
TACTCTGGTGACTACCCTGACCTATGGTGTTCAGTGTTTTTCTCGTTACCCTGACCACATGAAGCAACATGATTTCTTCAAAT
CTGCAATGCCGGAAGGTTATGTACAGGAGCGCACCATTTCTTTCAAAGACGATGGCACGTATAAAACCCGTGCAGAGGTTAAA
TTTGAAGGTGACACTCTGGTGAATCGTATTGAACTGAAAGGCATTGATTTCAAAGAGGACGGCAATATTTTAGGCCACAAACT
GGAATATAACTTCAACTCCCATAACGTTTACATCACCGCAGACAAACAGAAGAACGGTATCAAAGCTAACTTCAAAATTCGCC
ATAACGTTGAAGATGGTAGCGTACAGCTGGCGGATCATTACCAACAGAACACTCCGATTGGAGATGCTCCTGTTTTACTGCCG
GATAACCACTACCTGTCCACCCAGTCTAAACTGTCGAAGGATCCGAACGAAAAGCGCGACCACATGGTGTTATTAGAGTTCGT
TACCGCTAGTGGTATCACGCACGGTATGGATGAACTCTACAAATAAGACGAACAATAAGGGGAGCGGGAAACCGCTCCCCTTT
TTTATTGATAACAAAAGTAAATTGCACGCTGATAGTCTCCCAATTGCGAAGGACCAAAACGAAAAAACACCCTTTCGGGTGTC
TTTTCTGGAATTTGGTACCGAGTACTAGGTATCGTGTAAGTAGCGAAGGCCCGTACGCGAGATAAACTGCTAGGCAACCGCGA
CTCTACGACTGGTGCTCGATTTAATTTCGCTGACGTAAAGAAATTATCGGCAGTGCGTCAACTGCCGTATCTTTATCTTAATT
AGGTAGTTGGACAAGCCCTTGAAAGAAATAGCAAGAGCCTGCCTCTCTATTGAAGTCACGGCGAAAGTCGGGTAGAAATCAAA
GAAAGCAGAAATTAAATCGGAGTAACACTAAGGTGGGATAACTCCGTAACTGACTACGCCTTTCTCTAGACTTTACTTGACCA
GATACACTGTCTTTGACACGTTGAAGGATTAGAGCAATCAAATCCAAGACTGGCTAAGCACGAAGCAACTCTTGAGTGTTAAA
AAGTTATCTCCTGTATTCGGGAAGCGGGTACTAGAAGATTGCAGGGACTCCGACGTTAAGTAAATTACAAAGTAATAAGTATC
GTTCAGGATCACGTTACCGCAATAAGAAGCGAGAATAATATAATTTCCGAAGTGCTTACCCCAGTAGTGACTATTCCTATAAC
CCTTCTGAGTGTCCGGAGGCGGAAATTTGCCACGAAAGAGAAAGTATTTCCCCGACAATAATAAAGGGGCGCTCCTCAGCTTT
TCCACTTGGTTGGGTAAGCTAGGCAACTCTGAAAGGAGTTTCGGCGAATTGAAGCCGACAGCTTTGAATTGTTTTAGGGGCGT
TATTCGAGGGCAATCGGAGCTAACTTCAAGACTACTTCTTTGTTGAATACTAAATAGTGCAAAGGTCGTGTTTCCTCAAGGAT
ACTCCGCTAACAATATAGGATTCCAATCAGATTCAGCACTGGCGGTACGGGTGTTGCGGTGAGGCGTTCGGGTTTACGGCTCG
AAGCTAGCACGGTAGG

p2802

CGCAGGATAAGTAAGGGGAGTAAGTGATCGAACGAATCAGAAGTGACAATATACTTAGGCTGGATCTCGTCCCGTGAATCCCA
ACCCTCACCAACTACGAGATAAGAGGTAAGCCAAAAATCGACTTGGTGGCGACCAACGACTGTTCCCCCCCTGTAACTAATCG
TTCCGTCAAAACCTGACTTACTTCAAGGCCAATTCCAAGCGCAAACAATACCGTCCTAGTTCTTCGGTTAAGTTTCCGAAGTA
GGAGTGAGCCTACCTCCGTTTGCGTCTTGTTACCACTGACCCAGCTATTTACTTTGTATTGCCTGCAATCGAATTTCTGAACT
CTCAGATAGTGGGGATAACGGGAAAGTTCCTATATTTGCGAACTAACTTAGCCGTCCACCTCGAAGCTACCTACTCACACCCA
CCCCGCGCGGGGTAAATAAGGCACTAATCCCAGCTGAGAGCTGGCGTAGCACTTAGCCACAAGTTAATTAACAGTTGTCTGGT
AGTTTGGCGGTATTAGGAAGATCCTAGAAGCAAGGCAGAGTTAGTTCTAACCTAAAGCCACAAATAAGACAGGTTGCCAAAGC
CCGCCGGAAATTAAATCTTGCTCAGTTCGGTAACGGAGTTTCCCTCCCGCGTACTTAATTCCCAATAAGAAACGCGCCCAAGT
CCTATCAGGCAAAATTCAGCCCCTTCCCGTGTTAGAACGAGGGTAAAAATACAAGCCGATTGAACAAGGGTTGGGGGCTTCAA
ATCGTCGTTTACCCCACTTTACAACGGAGATTAAGTAGTTCACCCTATAGTACGAAGCAGAACTATTTCGAGGGGCGTGCAAT
AATCGAATCTTCTGCGGTTGACTTAACACGCTAGGGACGTGCCCTCGATTCAATCGAAGGTACTCCTACTCAGACTGCCTCAC
ACCCAGCTAGTCACTGAGCGATAAAATTGACCCGCCCTCTAGGGAAGCGAGTACGTCCCAAAGGGCTCCGGACAGGGCTATAT
AGGAGAGTTTGATCTCGCCCCGACAACTGCAACCCTCAACTCCCTTAGATAATATTGTTAGCCGAAGTTGCACGACCCGCCGT
CCACGGACTGCTCTTAGGGTGTGGCTCCTTAATCTGACAACGTGCAACCCCTATCGAAGTCGATTGTTTCTGCGAAAGGTGTT
GTCCTAATAGTCCCGAAATTTGGCCCTTGTAGGTGTGAAACCACTTAGCTTCGCGCCGTAGTCCTAAAGGCCCACCTATTGAC
TTTGTTTCGGGTAGCACTAGGAATCTTAACAATTTGAATTTGGACGTGGAACGCGTACACCTTAATCTCCGAATAATTCTAGG
GATTTGGAAGTCCTCTACGTTGACACACCTACACTGCTCGAAGTAAATATACGAATAACGCGGGCCTCGCGGAGCCGTTCCGA
ATCGTCACGTGTTCGTTTACTGTTAATTGGTGGCAAATAAGCAATATCGTAGTCCGTCAGGCCCAGCCCTGTTATCCACGGCG
TTATTTGTCAAATTGCGTAGAACTGGATTGACTGCCTGACAATACCTAATTATCGGTACGAAGTCCCCGAATCTGTCGGGCTA

TTTCACTAATACTTTCCAAACGCCCCGTATCCAAGAAGAACGAATTTATCCACGCTCCCGTCTTTGGGACGAATACCGCTACA
AGTGGACAGAGGATCGGTACGGGCCTCTAATAAATCCAACACTCTACGCCCTCTTCAAGAGCTAGAAGAACAGGGTGCAGTTG
GAAAGGGAATTATTTCGTAAGGCGAGCCAATACCGTAATTAATTCGGAAGAGTTAACACGATTGGAAGTAGGAATAGTTTCTA
ACCACGGTTACTAATCCTAATAACGGAACGCTGTCTGATAGATTAGTGTCAGCGCTCGGTACCAAAGAAAAATAAAAAGACGC
TGAAAAGCGTCTTTTTATTTTTCGGTCCAGTGTAACTCAGGCAAAAGCACGTAATATTCGTACTTTCTTCCTCCGTAAGCGTC
ACCCACATTCCTTAAAGAGTGCATGTGCATATTTTGTTATCAATAAAAAAGGCCGCGATTTGCGGCCTTATTGTTCGTCTTGC
CGGATTACGCCCCGCCCTGCCACTCATCGCAGTATTGTTGTAATTCATTAAGCATTCTGCCGACATGGAAGCCATCACAAACG
GCATGATGAACTTGGATCGCCAGTGGCATTAACACCTTGTCGCCTTGCGTATAATATTTTCCCATAGTGAAAACGGGGGCGAA
GAAGTTGTCCATATTTGCTACGTTTAAATCAAAACTGGTGAAACTCACCCACGGATTGGCACTGACGAAAAACATATTTTCGA
TAAACCCTTTAGGGAAATATGCTAAGTTTTCACCGTAACACGCCACATCTTGACTATATATGTGTAGAAACTGCCGGAAATCG
TCGTGGTATTCTGACCAGAGCGATGAAAACGTTTCAGTTTGCTCATGGAAAACGGTGTAACAAGGGTGAACACTATCCCATAT
CACCAGCTCACCGTCTTTCATTGCCATACGAAACTCCGGATGTGCATTCATCAGGCGGGCAAGAATGTGAATAAAGGCCGGAT
AAAACTTGTGCTTATTTTTCTTTACGGTTTTTAAAAAGGCCGTAATATCCAGCTGAACGGTTTGGTTATAGGTGCACTGAGCA
ACTGACTGGAATGCCTCAAAATGTTCTTTACGATGCCATTGACTTATATCAACTGTAGTATATCCAGTGATTTTTTTCTCCAT
TTTAGCTTCCTTAGCTTGCGAAATCTCGATAACTCAAAAAATAGTAGTGATCTTATTTCATTATGGTGAAAGTTGTCTTACGT
GCAACATTTTCGCAAAAAGTTGGCGCTTTATCAACACTGTCGGAATGACAAATGGTTCCAATTATTGAACACCCTTCGGGGTG
TTTTTTTGTTTCTGGTTTCCCGAGGCCGGCCTTTTGTTGCAATGGCTGTCTACCCTGTCTACCTGAGTAAAGAAAAATACATT
TAATTCAGTATATTAACTTGGGTAGACAGCCTTTTTTTACTGTCTACCTTCTGTCTACCCTCTCTACCTGATTTTACCTGAAT
CAGACAGGGAGGTAGACACGGGGTAGACAGTGGATAAAAGCACTCTACCCCACTGAAAGCAGTGCCATTACTGGCATGGTTGC
CAGTAAGGTTGATAAGGTAGACAAGGGGAGGGACAACTCAAAACTTTTTAAACGAGGGGGTAAAACGCAGACCAAAACGATCT
CAAGAAGATCATCTTATTAATCAGATAAAATATTTCTAGATTTCAGTGCAATTTATCTCTTCAAATGTAGCACCGGCGCGCCG
TGACCAATTATTGAAGGCCGCTAACGCGGCCTTTTTTTGTTTCTGGTATCCCGAATGGAGCGACTTCTCCCCAAAAAGCCTCG
CTTTCAGCACCTGTCGTTTCCTTTCTTTTCAGAGGGTATTTTAAATAAAAACATTAAGTTATGACGAAGAAGAACGGAAACGC
CTTAAACCGGAAAATTTTCATAAATAGCGAAAACCCGCGAGGTCGCCGCCCCGTAACCTGTCGGATCACCGGAAAGGACCCGT
AAAGTGATAATGATTATCATCTACATATCACAACGTGCGTAAAGGGTAAGTATGAAGGTCGTGTACTCCATCGCTACCAAATT
CCAGAAAACAGACGCTTTCGAGCGTCTTTTTTCGTTTTGGTCACGACGTACGGTGGAAGATTCGTTACCAATTGACAGCTAGC
TCAGTCCTAGGTATATACATACATGCTTGTTTGTTTGTAAACCAGGATAAAGAGAGAGCATACTCGATGAAACGTACTGCCGA
CGGTTCAGAGTTCGAGTCGCCCAAGAAGAAACGTAAAGTGTCATCCAAAACCGGCCCAGTTGCGGTGGACCCAACGCTGCGCC
GTCGTATCGAACCGCACGAGTTCGAAGTATTTTTCGATCCTCGCGAACTGCGTAAAGAGACCTGTTTACTTTACGAAATCAAT
TGGGGTGGCCGCCACTCGATTTGGCGCCACACGTCTCAGAATACAAACAAACATGTCGAAGTAAATTTCATCGAAAAATTTAC
AACGGAGCGTTATTTCTGTCCCAATACTCGTTGTTCTATTACATGGTTTTTATCATGGAGTCCCTGCGGGGAATGCTCTCGTG
CGATCACGGAGTTTCTGTCGCGTTATCCGAACGTAACGCTTTTTATCTATATTGCTCGTCTTTACCACTTGGCTAACCCTCGT
AACCGCCAAGGACTTCGCGACCTGATTTCGAGTGGAGTAACCATCCAGATCATGACTGAGCAAGAATCTGGATACTGTTGGCA
CAATTTCGTAAACTACTCGCCATCGAATGAGAGCCATTGGCCTCGCTACCCCCATCTTTGGGTACGTTTGTATGTCTTGGAAC
TTTATTGTATCATTTTAGGGCTGCCACCTTGTCTTAATATTTTGCGCCGCAAACAGAGTCAGCTGACATCTTTCACAATTGCT
TTACAGTCATGCCACTACCAACGTCTTCCACCGCACATTCTGTGGGCCACGGGCTTAAAATCGGGCGGTAGCTCAGGCGGGAG
TTCAGGCTCAGAAACGCCGGGAACTAGCGAATCCGCAACGCCAGAGTCTTCAGGTGGTTCGTCAGGTGGTTCAGACAAGAAAT
ATAGCATCGGCCTGGCCATCGGCACAAATAGCGTCGGATGGGCGGTGATCACTGATGAATATAAAGTTCCGTCTAAAAAGTTC
AAGGTACTGGGTAATACAGATCGCCATAGTATCAAAAAGAACTTAATCGGTGCGCTTCTGTTCGATTCCGGCGAAACCGCAGA
AGCAACACGTCTGAAACGCACCGCTCGTCGCCGTTACACCCGTCGTAAAAACCGCATCTGCTACCTGCAAGAAATCTTCTCTA
ACGAAATGGCTAAAGTAGATGACAGCTTTTTTCACCGTCTGGAAGAATCATTTCTGGTGGAAGAAGATAAAAAGCACGAACGT
CATCCAATCTTCGGCAACATTGTGGACGAAGTAGCGTATCACGAAAAATACCCGACTATCTATCACCTGCGCAAAAAGCTGGT
CGATTCGACGGATAAGGCCGATCTGCGTCTGATCTATCTGGCCTTAGCGCATATGATTAAGTTCCGTGGTCATTTCCTGATCG
AAGGCGACCTGAATCCAGACAACAGCGATGTAGACAAACTGTTCATCCAGCTGGTGCAAACCTATAACCAGCTGTTTGAAGAA
AACCCAATTAATGCTAGCGGTGTTGACGCGAAAGCGATCTTGTCCGCACGCCTGTCCAAATCCCGTCGTCTGGAAAACTTAAT
TGCGCAACTGCCGGGTGAGAAGAAAAACGGACTGTTCGGCAATCTGATCGCTCTTAGCTTGGGACTGACCCCGAACTTCAAAA
GCAACTTCGATCTGGCAGAGGACGCAAAACTTCAACTTAGCAAAGATACGTATGACGATGACTTGGATAACTTACTGGCCCAG
ATCGGAGATCAGTACGCTGATCTGTTTCTGGCGGCAAAGAACTTATCAGACGCTATTCTCCTGTCTGATATTCTTCGTGTGAA
TACCGAAATCACCAAAGCACCGCTTTCTGCATCCATGATTAAACGCTATGACGAACATCACCAAGATCTGACTCTTCTGAAAG
CGCTGGTACGGCAACAACTGCCGGAGAAGTACAAGGAGATCTTCTTTGACCAATCCAAAAACGGCTACGCGGGTTATATTGAC
GGGGGTGCAAGCCAAGAGGAGTTCTACAAATTCATCAAGCCAATCTTAGAAAAAATGGATGGCACGGAAGAATTACTTGTTAA
ACTGAATCGTGAGGATCTGCTTCGTAAACAGCGTACCTTCGACAACGGTAGCATTCCGCACCAGATCCACTTAGGTGAACTGC
ACGCTATCCTGCGTCGCCAAGAGGATTTTTACCCGTTCCTGAAAGATAATCGTGAAAAAATCGAAAAAATCCTGACCTTTCGT
ATCCCGTATTATGTCGGCCCGCTGGCGCGTGGCAACTCCCGTTTCGCGTGGATGACTCGCAAATCCGAAGAAACTATTACCCC
GTGGAACTTCGAGGAAGTGGTTGACAAAGGCGCAAGCGCCCAATCCTTCATCGAGCGCATGACTAACTTTGATAAAAACCTGC
CGAACGAAAAGGTACTGCCGAAACACTCCCTTCTGTACGAATACTTCACCGTGTACAACGAGCTGACTAAAGTAAAGTATGTG
ACTGAGGGCATGCGTAAACCTGCATTCCTGAGCGGTGAACAGAAAAAAGCAATTGTTGATTTACTGTTTAAAACCAACCGTAA
AGTAACCGTTAAACAGCTGAAAGAGGACTACTTCAAGAAAATCGAATGCTTCGACTCCGTCGAGATTAGTGGAGTTGAAGATC
GTTTTAATGCAAGTTTAGGCACGTATCACGATTTATTAAAGATCATTAAAGACAAAGATTTCTTGGACAACGAAGAAAATGAG
GACATCTTAGAGGACATCGTCCTGACCCTGACTCTGTTCGAAGATCGTGAAATGATTGAAGAACGCCTTAAGACGTATGCTCA
CCTGTTTGACGATAAAGTAATGAAACAACTGAAACGTCGCCGTTATACTGGCTGGGGCCGTCTGAGCCGTAAACTGATTAACG
GTATCCGTGACAAACAGTCCGGTAAAACTATTCTGGACTTCCTGAAATCTGACGGCTTCGCAAACCGTAACTTCATGCAACTG
ATTCACGACGATTCCCTGACCTTCAAAGAGGACATCCAGAAAGCTCAGGTTTCTGGTCAAGGTGATTCTCTGCACGAGCATAT

CGCCAATTTAGCAGGTAGTCCGGCGATCAAAAAAGGTATCCTGCAAACCGTGAAAGTGGTGGATGAGCTTGTGAAAGTTATGG
GTCGTCACAAACCGGAAAACATTGTTATCGAGATGGCTCGTGAAAACCAAACGACCCAGAAGGGACAGAAAAACTCCCGCGAA
CGCATGAAACGTATCGAGGAGGGTATTAAAGAACTTGGCTCTCAGATTCTGAAAGAACACCCTGTTGAAAATACCCAACTGCA
AAATGAAAAACTGTACCTGTACTACCTGCAAAATGGTCGTGACATGTATGTAGATCAGGAGCTGGACATCAACCGCCTCTCCG
ATTACGACGTTGACCACATTGTTCCGCAGTCTTTTCTGAAAGATGATTCCATTGATAACAAAGTACTCACCCGTAGCGATAAA
AACCGTGGGAAGAGTGACAACGTTCCATCGGAAGAAGTAGTTAAGAAAATGAAGAACTATTGGCGTCAACTGCTTAACGCGAA
ACTGATTACTCAACGTAAATTTGATAACCTGACCAAAGCTGAACGTGGCGGTTTGTCTGAGCTGGATAAGGCGGGTTTTATTA
AACGTCAACTGGTAGAAACTCGCCAGATTACAAAACATGTTGCTCAGATTCTGGACTCTCGTATGAACACTAAATACGATGAA
AATGACAAACTGATCCGCGAAGTTAAGGTTATTACCCTGAAATCTAAGCTGGTTTCCGACTTCCGTAAAGATTTCCAATTCTA
TAAAGTGCGCGAGATTAACAACTATCACCACGCGCACGACGCATATCTGAATGCAGTTGTTGGCACGGCACTGATCAAAAAAT
ATCCGAAACTGGAAAGCGAATTTGTGTACGGCGATTATAAAGTTTACGACGTGCGCAAAATGATCGCCAAATCTGAACAGGAA
ATTGGCAAAGCAACCGCTAAATACTTTTTCTACTCAAACATTATGAATTTCTTCAAAACCGAAATCACCTTAGCGAATGGCGA
AATTCGTAAACGCCCTCTGATCGAAACCAACGGCGAAACGGGTGAGATCGTGTGGGACAAAGGTCGTGATTTCGCTACTGTCC
GCAAAGTTCTGTCCATGCCTCAAGTAAACATCGTTAAAAAGACTGAGGTACAGACTGGCGGTTTCAGCAAGGAATCCATTCTG
CCGAAACGCAACTCCGACAAACTGATCGCGCGTAAGAAAGACTGGGATCCGAAGAAATACGGTGGCTTCGATTCTCCAACCGT
GGCATACAGCGTTCTGGTAGTCGCCAAAGTCGAAAAGGGTAAATCAAAAAAACTGAAATCAGTGAAAGAACTTTTAGGCATCA
CCATTATGGAACGTAGCTCTTTCGAAAAAAACCCGATTGACTTCCTCGAAGCGAAGGGGTACAAGGAAGTAAAGAAAGATCTG
ATTATCAAACTGCCGAAGTATTCCCTGTTCGAACTGGAAAATGGTCGTAAACGTATGTTAGCGTCTGCGGGTGAACTGCAAAA
AGGGAACGAATTGGCCCTTCCGTCCAAGTACGTGAACTTCCTGTATCTGGCCTCGCACTACGAGAAACTGAAAGGTAGTCCGG
AAGATAATGAGCAGAAACAGCTGTTCGTGGAACAGCACAAACACTATCTGGACGAGATTATTGAACAGATTTCTGAGTTTAGC
AAACGCGTAATTCTGGCGGACGCGAATCTGGATAAAGTCCTGAGCGCCTACAATAAACACCGTGATAAACCGATCCGTGAACA
GGCAGAAAACATCATTCACCTGTTCACGCTGACTAATCTTGGTGCTCCGGCAGCCTTCAAATACTTCGACACCACGATCGATC
GTAAACGTTACACCTCCACTAAAGAAGTCTTAGATGCAACTCTTATTCACCAGAGCATCACTGGCCTGTATGAAACTCGTATT
GATCTGAGTCAGTTGGGCGGTGACAGTGGGGGCAGCGGAGGTAGCGGAGGCAGCACTAATTTGTCGGATATTATTGAGAAGGA
AACAGGAAAGCAATTGGTTATCCAGGAATCCATTTTAATGCTTCCGGAAGAAGTGGAAGAGGTGATCGGAAATAAGCCAGAGA
GCGATATCTTAGTTCATACGGCCTATGATGAAAGCACTGACGAGAACGTTATGCTGTTAACATCGGACGCTCCCGAGTACAAG
CCGTGGGCCCTGGTTATCCAGGACAGTAATGGCGAAAATAAAATTAAAATGTTATAATGTATGCTTAAGCAGCTCGGTACCAA
AGACGAACAATAAGACGCTGAAAAGCGTCTTTTTTCGTTTTGGTCCTGTTGCGGCGCGATAGTGTGAACATGCTATAGACTTC
TGGTGCTACCCGACTGACAATTAATCATCCGGCTCGTATAATGCTAGCAACTTTCAGAAATTAAGTCGGTTTTAGAGCTAGAA
ATAGCAAGTTAAAATAAGGCTAGTCCGTTATCAACTTGAAAAAGTGGCACCGAGTCGGTGCTTTTTTTAATCCATTCGAATCA
CGTCCGACGAACAATAAGGCCTCCCTAACGGGGGGCCTTTTTTATTGATAACAAAAGTAACTTCGAGCTTGTCTACCTCCTAG
CACCATTATTGCAATTAATAAACAACTAACGGACAATTCTACCTAACAGTTTTCATATATGACGAGCAGTTAAGTGATGAGTA
AAGGTGAGGAATTATTTACTGGTGTTGTTCCGATCTTAGTTGAACTGGACGGCGATGTTAACGGTCATAAATTCAGTGTTCGT
GGTGAAGGTGAAGGTGATGCAACCAACGGTAAGCTGACCCTGAAATTCATCTGCACTACTGGAAAATTACCAGTACCGTGGCC
TACTCTGGTGACTACCCTGACCTATGGTGTTCAGTGTTTTTCTCGTTACCCTGACCACATGAAGCAACATGATTTCTTCAAAT
CTGCAATGCCGGAAGGTTATGTACAGGAGCGCACCATTTCTTTCAAAGACGATGGCACGTATAAAACCCGTGCAGAGGTTAAA
TTTGAAGGTGACACTCTGGTGAATCGTATTGAACTGAAAGGCATTGATTTCAAAGAGGACGGCAATATTTTAGGCCACAAACT
GGAATATAACTTCAACTCCCATAACGTTTACATCACCGCAGACAAACAGAAGAACGGTATCAAAGCTAACTTCAAAATTCGCC
ATAACGTTGAAGATGGTAGCGTACAGCTGGCGGATCATTACCAACAGAACACTCCGATTGGAGATGCTCCTGTTTTACTGCCG
GATAACCACTACCTGTCCACCCAGTCTAAACTGTCGAAGGATCCGAACGAAAAGCGCGACCACATGGTGTTATTAGAGTTCGT
TACCGCTAGTGGTATCACGCACGGTATGGATGAACTCTACAAATAAGACGAACAATAAGGGGAGCGGGAAACCGCTCCCCTTT
TTTATTGATAACAAAAGTAAATTGCACGCTGATAGTCTCCCAATTGCGAAGGACCAAAACGAAAAAACACCCTTTCGGGTGTC
TTTTCTGGAATTTGGTACCGAGTACTAGGTATCGTGTAAGTAGCGAAGGCCCGTACGCGAGATAAACTGCTAGGCAACCGCGA
CTCTACGACTGGTGCTCGATTTAATTTCGCTGACGTAAAGAAATTATCGGCAGTGCGTCAACTGCCGTATCTTTATCTTAATT
AGGTAGTTGGACAAGCCCTTGAAAGAAATAGCAAGAGCCTGCCTCTCTATTGAAGTCACGGCGAAAGTCGGGTAGAAATCAAA
GAAAGCAGAAATTAAATCGGAGTAACACTAAGGTGGGATAACTCCGTAACTGACTACGCCTTTCTCTAGACTTTACTTGACCA
GATACACTGTCTTTGACACGTTGAAGGATTAGAGCAATCAAATCCAAGACTGGCTAAGCACGAAGCAACTCTTGAGTGTTAAA
AAGTTATCTCCTGTATTCGGGAAGCGGGTACTAGAAGATTGCAGGGACTCCGACGTTAAGTAAATTACAAAGTAATAAGTATC
GTTCAGGATCACGTTACCGCAATAAGAAGCGAGAATAATATAATTTCCGAAGTGCTTACCCCAGTAGTGACTATTCCTATAAC
CCTTCTGAGTGTCCGGAGGCGGAAATTTGCCACGAAAGAGAAAGTATTTCCCCGACAATAATAAAGGGGCGCTCCTCAGCTTT
TCCACTTGGTTGGGTAAGCTAGGCAACTCTGAAAGGAGTTTCGGCGAATTGAAGCCGACAGCTTTGAATTGTTTTAGGGGCGT
TATTCGAGGGCAATCGGAGCTAACTTCAAGACTACTTCTTTGTTGAATACTAAATAGTGCAAAGGTCGTGTTTCCTCAAGGAT
ACTCCGCTAACAATATAGGATTCCAATCAGATTCAGCACTGGCGGTACGGGTGTTGCGGTGAGGCGTTCGGGTTTACGGCTCG
AAGCTAGCACGGTAGG

p2826

CATATTTGCTACGTTTAAATCAAAACTGGTGAAACTCACCCACGGATTGGCACTGACGAAAAACATATTTTCGATAAACCCTT
TAGGGAAATATGCTAAGTTTTCACCGTAACACGCCACATCTTGACTATATATGTGTAGAAACTGCCGGAAATCGTCGTGGTAT
TCTGACCAGAGCGATGAAAACGTTTCAGTTTGCTCATGGAAAACGGTGTAACAAGGGTGAACACTATCCCATATCACCAGCTC

ACCGTCTTTCATTGCCATACGAAACTCCGGATGTGCATTCATCAGGCGGGCAAGAATGTGAATAAAGGCCGGATAAAACTTGT
GCTTATTTTTCTTTACGGTTTTTAAAAAGGCCGTAATATCCAGCTGAACGGTTTGGTTATAGGTGCACTGAGCAACTGACTGG
AATGCCTCAAAATGTTCTTTACGATGCCATTGACTTATATCAACTGTAGTATATCCAGTGATTTTTTTCTCCATTTTAGCTTC
CTTAGCTTGCGAAATCTCGATAACTCAAAAAATAGTAGTGATCTTATTTCATTATGGTGAAAGTTGTCTTACGTGCAACATTT
TCGCAAAAAGTTGGCGCTTTATCAACACTGTCCCTCCTGTTCAGCTACTGACGGTACTGCGGAACTGACTAAAGTAGTgCGTA
ACGGCAAAAGCACCGCCGGACATCAGCGCTAGCGGAGTGTATACTGGCTTACTATGTTGGCACTGATGAGGGTGTCAGTGAAG
TGCTTCATGTGGCAGGAGAAAAAAGGCTGCACCGGTGCGTCAGCAGAATATGTGATACAGGATATATTCCGCTTCCTCGCTCA
CTGACTCGCTACGCTCGGTCGTTCGACTGCGGCGAGCGGAAATGGCTTACGAACGGGGCGGAGATTTCCTGAAGATGCCAGG
AAGATACTTAACAGGGAAGTGAGAGGGCCGCGGCAAAGCCGTTTTTCCATAGGCTCCGCCCCCCTGACAAGCATCACGAAATC
TGACGCTCAAATCAGTGGTGGCGAAACCtGACAGGACTATAAAGATACCAGGCGTTTCCCCCTGGCGGCTCCCTCGTGCGCTC
TCCTGTTCCTGCCTTTCGGTTTACCGGTGTCATTCCGCTGTTATGGCCGCGTTTGTCTCATTCCACGCCTGACACTCAGTTCC
GGGTAGGCAGTTCGCTCCAAGCTGGACTGTATGCACGAACCCCCCGTTCAGTCCGACCGCTGCGCCTTATCCGGTAACTATCG
TCTTGAGTCCAACCCGGAAAGACATGCAAAAGCACCACTGGCAGCAGCCACTGGTAATTGATTTAGAGGAGTTAGTCTTGAAG
TCATGCGCCGGTTAAGGCTAAACTGAAAGGACAAGTTTTGGTGACTGCGCTCCTCCAAGCCAGTTACCTCGGTTCAAAGAGTT
GGTAGCTCAGAGAACCTTCGAAAAACCGCCCTGCAAGGCGGTTTTTTCGTTTTCAGAGCAAGAGATTACGCGCAGACCAAAAC
GATCTCAAGAAGATCATCTTATTAATCAGATAAAATATTTCTAGATTTCAGTGCAATTTATCTCTTCAAATGTAGCACgattt
tacggctagctcagtcctaggtaTaatgctagcgaatcattaaagaggagaaaggtactATGGCACGTACCCCGTCACGTAGT
AGCATTGGTAGCCTGCGTAGTCCGCATACCCATAAAGCAATTCTGACCAGTACCATCGAGATCCTGAAAGAATGTGGTTATAG
CGGACTGAGCATTGAAAGCGTTGCACGTCGTGCCGGAGCAAGCAAACCGACCATTTATCGTTGGTGGACGAATAAAGCAGCAC
TGATTGCCGAAGTGTATGAAAATGAAAGCGAACAGGTGCGTAAATTTCCGGATCTGGGTAGCTTTAAAGCAGATCTGGATTTT
TTACTGCGTAATTTATGGAAAGTTTGGCGTGAAACTATTTGCGGTGAAGCATTTCGTTGTGTTATTGCAGAAGCTCAGCTGGA
TCCTGCAACCCTGACCCAGTTAAAGGATCAATTTATGGAACGTCGTCGTGAGATGCCGAAAAAACTGGTTGAAAATGCCATTA
GCAATGGTGAACTGCCGAAAGATACCAATCGTGAACTTCTTCTGGATATGATTTTTGGTTTTTGTTGGTATCGCCTGTTAACC
GAACAGCTGACCGTTGAACAGGATATTGAAGAATTTACCTTCCTTCTGATTAATGGTGTTTGTCCGGGTACTCAGCGTTAACT
AGGCCATAATCGCTACCAAATTCCAGAAAACAGACGCTTTCGAGCGTCTTTTTTCGTTTTGGTCACGACGTACTGAATCTGAT
TCGTTACCAATTGACATGATACGAAACGTACCGTATCGTTAAGGTTACTGGAGCTTAAAAAGGAGAAAGTTTCTATGGATAAG
AAATACAGCATAGGCTTAGACATCGGCACAAATAGCGTCGGATGGGCGGTGATCACTGATGAATATAAAGTTCCGTCTAAAAA
GTTCAAGGTACTGGGTAATACAGATCGCCATAGTATCAAAAAGAACTTAATCGGTGCGCTTCTGTTCGATTCCGGCGAAACCG
CAGAAGCAACACGTCTGAAACGCACCGCTCGTCGCCGTTACACCCGTCGTAAAAACCGCATCTGCTACCTGCAAGAAATCTTC
TCTAACGAAATGGCTAAAGTAGATGACAGCTTTTTTCACCGTCTGGAAGAATCATTTCTGGTGGAAGAAGATAAAAAGCACGA
ACGTCATCCAATCTTCGGCAACATTGTGGACGAAGTAGCGTATCACGAAAAATACCCGACTATCTATCACCTGCGCAAAAAGC
TGGTCGATTCGACGGATAAGGCCGATCTGCGTCTGATCTATCTGGCCTTAGCGCATATGATTAAGTTCCGTGGTCATTTCCTG
ATCGAAGGCGACCTGAATCCAGACAACAGCGATGTAGACAAACTGTTCATCCAGCTGGTGCAAACCTATAACCAGCTGTTTGA
AGAAAACCCAATTAATGCTAGCGGTGTTGACGCGAAAGCGATCTTGTCCGCACGCCTGTCCAAATCCCGTCGTCTGGAAAACT
TAATTGCGCAACTGCCGGGTGAGAAGAAAAACGGACTGTTCGGCAATCTGATCGCTCTTAGCTTGGGACTGACCCCGAACTTC
AAAAGCAACTTCGATCTGGCAGAGGACGCAAAACTTCAACTTAGCAAAGATACGTATGACGATGACTTGGATAACTTACTGGC
CCAGATCGGAGATCAGTACGCTGATCTGTTTCTGGCGGCAAAGAACTTATCAGACGCTATTCTCCTGTCTGATATTCTTCGTG
TGAATACCGAAATCACCAAAGCACCGCTTTCTGCATCCATGATTAAACGCTATGACGAACATCACCAAGATCTGACTCTTCTG
AAAGCGCTGGTACGGCAACAACTGCCGGAGAAGTACAAGGAGATCTTCTTTGACCAATCCAAAAACGGCTACGCGGGTTATAT
TGACGGGGGTGCAAGCCAAGAGGAGTTCTACAAATTCATCAAGCCAATCTTAGAAAAAATGGATGGCACGGAAGAATTACTTG
TTAAACTGAATCGTGAGGATCTGCTTCGTAAACAGCGTACCTTCGACAACGGTAGCATTCCGCACCAGATCCACTTAGGTGAA
CTGCACGCTATCCTGCGTCGCCAAGAGGATTTTTACCCGTTCCTGAAAGATAATCGTGAAAAAATCGAAAAAATCCTGACCTT
TCGTATCCCGTATTATGTCGGCCCGCTGGCGCGTGGCAACTCCCGTTTCGCGTGGATGACTCGCAAATCCGAAGAAACTATTA
CCCCGTGGAACTTCGAGGAAGTGGTTGACAAAGGCGCAAGCGCCCAATCCTTCATCGAGCGCATGACTAACTTTGATAAAAAC
CTGCCGAACGAAAAGGTACTGCCGAAACACTCCCTTCTGTACGAATACTTCACCGTGTACAACGAGCTGACTAAAGTAAAGTA
TGTGACTGAGGGCATGCGTAAACCTGCATTCCTGAGCGGTGAACAGAAAAAAGCAATTGTTGATTTACTGTTTAAAACCAACC
GTAAAGTAACCGTTAAACAGCTGAAAGAGGACTACTTCAAGAAAATCGAATGCTTCGACTCCGTCGAGATTAGTGGAGTTGAA
GATCGTTTTAATGCAAGTTTAGGCACGTATCACGATTTATTAAAGATCATTAAAGACAAAGATTTCTTGGACAACGAAGAAAA
TGAGGACATCTTAGAGGACATCGTCCTGACCCTGACTCTGTTCGAAGATCGTGAAATGATTGAAGAACGCTTGAAGACGTATG
CTCACCTGTTTGACGATAAAGTAATGAAACAgCTGAAACGTCGCCGTTATACTGGCTGGGGCCGTCTGAGCCGTAAACTGATT
AACGGTATCCGTGACAAACAGTCCGGTAAAACTATTCTGGACTTCCTGAAATCTGACGGCTTCGCAAACCGTAACTTCATGCA
ACTGATTCACGACGATTCCCTGACCTTCAAAGAGGACATCCAGAAAGCTCAGGTTTCTGGTCAAGGTGATTCTCTGCACGAGC
ATATCGCCAATTTAGCAGGTAGTCCGGCGATCAAAAAAGGTATCCTGCAAACCGTGAAAGTGGTGGATGAGCTTGTGAAAGTT
ATGGGTCGTCACAAACCGGAAAACATTGTTATCGAGATGGCTCGTGAAAACCAAACGACCCAGAAGGGACAGAAAAACTCCCG
CGAACGCATGAAACGTATCGAGGAGGGTATTAAAGAACTTGGCTCTCAGATTCTGAAAGAACACCCTGTTGAAAATACCCAAC
TGCAAAATGAAAAACTGTACCTGTACTACCTGCAAAATGGTCGTGACATGTATGTAGATCAGGAGCTGGACATCAACCGCCTC
TCCGATTACGACGTTGACCACATTGTTCCGCAGTCTTTTCTGAAAGATGATTCCATTGATAACAAAGTACTCACCCGTAGCGA
TAAAAACCGTGGGAAGAGTGACAACGTTCCATCGGAAGAAGTAGTTAAGAAAATGAAGAACTATTGGCGTCAACTGCTTAACG
CGAAACTGATTACTCAACGTAAATTTGATAACCTGACCAAAGCTGAACGTGGCGGTTTGTCTGAGCTGGATAAGGCGGGTTTT
ATTAAACGTCAACTGGTAGAAACTCGCCAGATTACAAAACATGTTGCTCAGATTCTGGACTCTCGTATGAACACTAAATACGA
TGAAAATGACAAACTGATCCGCGAAGTTAAGGTTATTACCCTGAAATCTAAGCTGGTTTCCGACTTCCGTAAAGATTTCCAAT
TCTATAAAGTGCGCGAGATTAACAACTATCACCACGCGCACGACGCATATCTGAATGCAGTTGTTGGCACGGCACTGATCAAA
AAATATCCGAAACTGGAAAGCGAATTTGTGTACGGCGATTATAAAGTTTACGACGTGCGCAAAATGATCGCCAAATCTGAACA

GGAAATTGGCAAAGCAACCGCTAAATACTTTTTCTACTCAAACATTATGAATTTCTTCAAAACCGAAATCACCTTAGCGAATG
GCGAAATTCGTAAACGCCCTCTGATCGAAACCAACGGCGAAACGGGTGAGATCGTGTGGGACAAAGGTCGTGATTTCGCTACT
GTCCGCAAAGTTCTGTCCATGCCTCAAGTAAACATCGTTAAAAAGACTGAGGTACAGACTGGCGGTTTCAGCAAGGAATCCAT
TCTGCCGAAACGCAACTCCGACAAACTGATCGCGCGTAAGAAAGACTGGGATCCGAAGAAATACGGTGGCTTCGATTCTCCAA
CCGTGGCATACAGCGTTCTGGTAGTCGCCAAAGTCGAAAAGGGTAAATCAAAAAAACTGAAATCAGTGAAAGAACTTTTAGGC
ATCACCATTATGGAACGTAGCTCTTTCGAAAAAAACCCGATTGACTTCCTCGAAGCGAAGGGGTACAAGGAAGTAAAGAAAGA
TCTGATTATCAAACTGCCGAAGTATTCCCTGTTCGAACTGGAAAATGGTCGTAAACGTATGTTAGCGTCTGCGGGTGAACTGC
AAAAAGGGAACGAATTGGCCCTTCCGTCCAAGTACGTGAACTTCCTGTATCTGGCCTCGCACTACGAGAAACTGAAAGGTAGT
CCGGAAGATAATGAGCAGAAACAGCTGTTCGTGGAACAGCACAAACACTATCTGGACGAGATTATTGAACAGATTTCTGAGTT
TAGCAAACGCGTAATTCTGGCGGACGCGAATCTGGATAAAGTCCTGAGCGCCTACAATAAACACCGTGATAAACCGATCCGTG
AACAGGCAGAAAACATCATTCACCTGTTCACGCTGACTAATCTTGGTGCTCCGGCAGCCTTCAAATACTTCGACACCACGATC
GATCGTAAACGTTACACCTCCACTAAAGAAGTCTTAGATGCAACTCTTATTCACCAGAGCATCACTGGCCTGTATGAAACTCG
TATTGATCTGAGtCAGTTGGGCGGTGACTAATAACGTTAAAGTCAGTTTCACCTGTTTTACGTTAAAACCCGCTTCGGCGGGT
TTTTACTTTTGGGtttAGCCGAACGCCCCAAAAAGCCTCGCTTTCAGCACCTGTCGTTTCCTTTCTTTTCAGAGGGTATTTTA
AATAAAAACATTAAGTTATGACGAAGAAGAACGGAAACGCCTTAAACCGGAAAATTTTCATAAATAGCGAAAACCCGCGAGGT
CGCCGCCCCGTAACCTGTCGGATCACCGGAAAGGACCCGTAAAGTGATAATGATTATCATCTACATATCACAACGTGCGTAAA
GGGactagtggatGtttCCAAGGTTCTGATGACAGCTCAATCGATCTGACTCAGCGTGGTTTCGGCAACAGCGCTACTCTTGA
TCAGTGGAATGGTAAAGATTCTACTATGACTGTTAAACAGTTCGGTGGCGGTAACGGTGCTGCTGTTGACCAGACTGCATCTA
ACTCCAGCGTTAACGTCACTCAGGTTGGCTTTGGTAACAACGCGACCGCTCATCAGTACGGTGGCAGCGGCAACTGGAGCCAC
CCGCAGTTCGAAAAGtaaAGGGCGTAAGCCCTGTTTTTTTTTCGGGAGAAGAATATGAATGCGTTATTACTCCTTGCGGCACT
TTCCAGTCAGATAACCTTTAATACGACCCAGCAAGGGGATATGTACACCATTATTCCTGAAGTCACTCTTACTCAATCTTGTC
TGTGCAGAGTACAAATATTGTCCCTGCGCGAAGGCAGTTCAGGGCAAAGTCAGACGAAGCAAGAAAAGACCCTCTCATTGCCT
GCTAATCAACCCATTGCTTGATCTCAAAAAAAGCACCGACTCGGTGCCACTTTTTCAAGTTGATAACGGACTAGCCTTATTTT
AACTTGCTATTTCTAGCTCTAAAACTGTTTCTTTCATACAGATGAgctagcattatacctaggactgagctagctgtcagTCG
GGTAGCACCAGAAGTCTATAGCATGtgcataCCTTTGGTCGAAAAAAAAAGCCCGCACTGTCAGGTGCGGGCTTTTTTCaGTG
TTTCCttgccggaTTACGCCCCGCCCTGCCACTCATCGCAGTATTGTTGTAATTCATTAAGCATTCTGCCGACATGGAAGCCA
TCACAAACGGCATGATGAACTTGGATCGCCAGTGGCATTAACACCTTGTCGCCTTGCGTATAATATTTTCCCATAGTGAAAAC
GGGGGCGAAGAAGTTGTC
