# Supplementary material for: In situ targeted base editing of bacteria in the mouse gut
Source: Nature. 2024 Jul 10;632(8026):877–84. doi: 10.1038/s41586-024-07681-w (PMC11338833; doi:10.1038/s41586-024-07681-w)
Supplement: Supplementary file 2 — Reporting Summary [file 41586_2024_7681_MOESM2_ESM.pdf]

nature portfolio

Corresponding author(s): David Bikard
Last updated by author(s): May 15, 2024

Reporting Summary

Nature Portfolio wishes to improve the reproducibility of the work that we publish. This form provides structure for consistency and transparency in reporting. For further information on Nature Portfolio policies, see our Editorial Policies and the Editorial Policy Checklist.

Please do not complete any field with "not applicable" or n/a. Refer to the help text for what text to use if an item is not relevant to your study.
For final submission: please carefully check your responses for accuracy; you will not be able to make changes later.

Statistics

For all statistical analyses, confirm that the following items are present in the figure legend, table legend, main text, or Methods section.

n/a	Confirmed	
☐	☒	The exact sample size (*n*) for each experimental group/condition, given as a discrete number and unit of measurement
☐	☒	A statement on whether measurements were taken from distinct samples or whether the same sample was measured repeatedly
☐	☒	The statistical test(s) used AND whether they are one- or two-sided<br>*Only common tests should be described solely by name; describe more complex techniques in the Methods section.*
☐	☒	A description of all covariates tested
☐	☒	A description of any assumptions or corrections, such as tests of normality and adjustment for multiple comparisons
☐	☒	A full description of the statistical parameters including central tendency (e.g. means) or other basic estimates (e.g. regression coefficient) AND variation (e.g. standard deviation) or associated estimates of uncertainty (e.g. confidence intervals)
☐	☒	For null hypothesis testing, the test statistic (e.g. *F*, *t*, *r*) with confidence intervals, effect sizes, degrees of freedom and *P* value noted<br>*Give P values as exact values whenever suitable.*
☒	☐	For Bayesian analysis, information on the choice of priors and Markov chain Monte Carlo settings
☒	☐	For hierarchical and complex designs, identification of the appropriate level for tests and full reporting of outcomes
☒	☐	Estimates of effect sizes (e.g. Cohen's *d*, Pearson's *r*), indicating how they were calculated

Our web collection on statistics for biologists contains articles on many of the points above.

Software and code

Policy information about availability of computer code

Data collection

No software was used for data collection

Data analysis

Microbiome analysis:
Obtained paired-end reads were filtered by length (cutadapt v3.357; --minimum-length parameter set to 20), merged (FLASH v1.2.1158, parameters --min-overlap 10 --max-mismatch-density 0.2) and filtered by quality (fastp v0.23.159; parameters -q 19 -u 15). Subsequently, chimeric sequences were removed using vsearch v2.16.060 with Silva reference database61. At this point, one sample (D5 M36) had to be excluded from the subsequent analysis due to low sequencing depth (11499 non-chimeric reads). Sequences were clustered into operational taxonomic units (OTUs) at a 97% similarity threshold with Uparse v7.0.100162 and annotated with taxonomy information using QIIME v1.9.163 and Silva database. The QIIME toolkit was also used for taxonomic profiling of the samples and for obtaining the beta-diversity values. The phylogenetic tree supplied to the UniFrac distance computation64 was generated using MUSCLE 3.8.155165 (-maxiters parameter set to 2).

Off-target analysis:
The program fastp v0.23.259 was used for quality control (QC) of the raw reads. Since multiple libraries were sequenced to obtain the desired depth, all QC-passing reads from the same sample were merged into a single file. The MG1655 complete reference genome was downloaded from NCBI (nuccore accession number: NC_000913.3), manually modified to reflect the genetic modifications to the wild-type strain, and further corrected using breseq with the reads from the control sample S1. For TN03 (NCBI assembly accession number: GCA_015186165.1), the complete reference genome obtained from a previous PacBio sequencing run of the same strain was used. The versions of both reference genomes used in the following analyses are available at https://github.com/Eligo-Bioscience/in-situ-targeted-base-editing-of-gut-bacteria-in-mice.

The four readsets (two repeats for base-edited, ABE S1 and ABE S2, and two repeats for controls, Control S1 and Control S2) corresponding to each of the two strains were aligned against their respective reference genome using bwa v0.7.17-r118868 (mem algorithm, default parameters). For each position of the reference genome, the frequency of each nucleotide was computed with a custom Python script using the pileup function of pysam v0.20.069. The "read mismatch frequency" was calculated as the ratio between the number of aligned reads differing from the reference and the total alignment coverage at each position.

Flow cytometry:
Flow cytometry data was collected on the Attune Nxt Thermo Scientific Instrument and analysed with v4.2.0 of the Attune Nxt software.

The code used for the analysis and the generation of the figures is available at https://github.com/Eligo-Bioscience/in-situ-targeted-base-editing-of-gut-bacteria-in-mice and was deposited on Zenodo: https://doi.org/10.5281/zenodo.11198996

For manuscripts utilizing custom algorithms or software that are central to the research but not yet described in published literature, software must be made available to editors and reviewers. We strongly encourage code deposition in a community repository (e.g. GitHub). See the Nature Portfolio guidelines for submitting code & software for further information.

Data

Policy information about availability of data

All manuscripts must include a data availability statement. This statement should provide the following information, where applicable:

- Accession codes, unique identifiers, or web links for publicly available datasets
- A description of any restrictions on data availability
- For clinical datasets or third party data, please ensure that the statement adheres to our policy

Sequencing reads are available on NCBI SRA (Bioproject PRJNA944658)
https://www.ncbi.nlm.nih.gov/bioproject/PRJNA944658

Reference genome for E. coli strains were downloaded from NCBI: MG1655 (nuccore accession number: NC_000913.3),TN03 (NCBI assembly accession number: GCA_015186165.1)

Human research participants

Policy information about studies involving human research participants and Sex and Gender in Research.

Reporting on sex and gender	N/A
Population characteristics	N/A
Recruitment	N/A
Ethics oversight	N/A

Note that full information on the approval of the study protocol must also be provided in the manuscript.

Field-specific reporting

Please select the one below that is the best fit for your research. If you are not sure, read the appropriate sections before making your selection.

☒ Life sciences ☐ Behavioural & social sciences ☐ Ecological, evolutionary & environmental sciences

For a reference copy of the document with all sections, see nature.com/documents/nr-reporting-summary-flat.pdf

Life sciences study design

All studies must disclose on these points even when the disclosure is negative.

Sample size

We selected groups of 10 animals which should enable to measure a difference in the proportion of edited bacterial in animals between 40% and 90%, given the following formula:

n = (Zα+Zβ)^2 * (p1(1-p1)+p2(1-p2)) / (p1-p2)^2,

where Zα is the critical value of the Normal distribution at α/2 (α is 0.05 and the critical value is 1.96), Zβ is the critical value of the Normal distribution at β (for a power of 80%, β is 0.2 and the critical value is 0.84) and p1 and p2 are the expected sample proportions of the two groups.

Wang, H. and Chow, S.-C. 2007. Sample Size Calculation for Comparing Proportions. Wiley Encyclopedia of Clinical Trials.

	After observing a higher variability of editing efficiencies in experiments performed with strain TN03 we increased the sample size to 15 mouse per group for the experiment in which mice were treated with multiple doses.
Data exclusions	Occasionally, a particular mouse did not yield any stool sample at a given timepoint and no data could be gathered (ãround 10% of animals at a given time point).<br><br>Specifically:<br>Targeted adenine base editing on the E. coli genome in gut of BALB/c mice after packaged λ cosmid treatment using a non-replicative payload<br><br>- In the 4x10^10tu/dose single dose group , we excluded one mouse from any subsequent analysis because it was not properly treated.<br>- In the 4x10^10tu/dose single dose group, we excluded one other mouse from the D7 time point because of a lack of feces.<br>- In the 1x10^10tu/dose multidose group (1 dose per day for 3 days) , we excluded one mouse from the D7 time point for editing efficacy and payload shedding because of a lack of feces.<br><br>Targeted adenine base editing of the csgA gene on the E. coli TN03 genome in the gut of BALB/c mice after packaged λ cosmid treatment using a non-replicative payload.<br>- In the 1x10^10tu/dose multidose group one animal died before the beginning of treatment. Data for 14/15 animals is thus reported.
Replication	In vitro experiments were typically performed in triplicate or as indicated in the figure legend. Animal experiments were not replicated to limit the number of animals used and the cost of the experiments. Each animal experiment did however include multiple animals in each treatment group as indicated.
Randomization	Animals for randomly assigned into cages upon reception. After acclimatation, cages where randomly assigned to treatment groups.
Blinding	Samples processing and data analysis (including reading experimental results) were performed by different investigators for all animal experiments.

Reporting for specific materials, systems and methods

We require information from authors about some types of materials, experimental systems and methods used in many studies. Here, indicate whether each material, system or method listed is relevant to your study. If you are not sure if a list item applies to your research, read the appropriate section before selecting a response.

Materials & experimental systems

n/a	Involved in the study
☒	☐ Antibodies
☒	☐ Eukaryotic cell lines
☒	☐ Palaeontology and archaeology
☐	☒ Animals and other organisms
☒	☐ Clinical data
☒	☐ Dual use research of concern

Methods

n/a	Involved in the study
☒	☐ ChIP-seq
☐	☒ Flow cytometry
☒	☐ MRI-based neuroimaging

Animals and other research organisms

Policy information about studies involving animals; ARRIVE guidelines recommended for reporting animal research, and Sex and Gender in Research

Laboratory animals	Specific pathogen-free 5 to 9 week old female BALB/cYJ mice were supplied by Charles River Laboratories and housed in an animal facility in accordance with Institut Pasteur's guidelines and European recommendations.
Wild animals	no wild animals were used in the study
Reporting on sex	To minimize variability and facilitate randomisation, only females were used in the findings reported in this paper. Plans will be made to confirm these results in males.
Field-collected samples	no field collected samples were used in the study
Ethics oversight	Animal procedures were approved by the Institut Pasteur (approval ID: 20040) and the French Research Ministry (APAFIS ID: 28717) and animal experiments were performed in compliance with applicable ethical regulations.

Note that full information on the approval of the study protocol must also be provided in the manuscript.

Flow Cytometry

Plots

Confirm that:

☒ The axis labels state the marker and fluorochrome used (e.g. CD4-FITC).

☒ The axis scales are clearly visible. Include numbers along axes only for bottom left plot of group (a 'group' is an analysis of identical markers).

☒ All plots are contour plots with outliers or pseudocolor plots.

☒ A numerical value for number of cells or percentage (with statistics) is provided.

Methodology

Sample preparation	8 µl of sample were added to 250 µl ice-cold PBS plus 1 mg ml-1 kanamycin<br>prior to analysis (see Materials and Methods)
Instrument	Attune NxT Thermo Scientific
Software	Attune NxT Software v4.2.0
Cell population abundance	the initial R1 gate (SSC-H/FSC-H) for bacterial cells corresponds to >= 80% of total events
Gating strategy	R1 gate on dot density plot of all events, SSC-H/FSC-H<br>R2 gate on dot density plot of [R1], SSC-A/SSC-H (single cells)<br>R3 gate on histogram plot of [R2] (Count/BL1-H for GFP or Count/YL2-H for mCherry). GFP+ or mCherry+ population gated and its percentage plotted.

☒ Tick this box to confirm that a figure exemplifying the gating strategy is provided in the Supplementary Information.

This checklist template is licensed under a Creative Commons Attribution 4.0 International License, which permits use, sharing, adaptation, distribution and reproduction in any medium or format, as long as you give appropriate credit to the original author(s) and the source, provide a link to the Creative Commons license, and indicate if changes were made. The images or other third party material in this article are included in the article's Creative Commons license, unless indicated otherwise in a credit line to the material. If material is not included in the article's Creative Commons license and your intended use is not permitted by statutory regulation or exceeds the permitted use, you will need to obtain permission directly from the copyright holder. To view a copy of this license, visit http://creativecommons.org/licenses/by/4.0/
